# Supplementary material for: Deciphering minimal antigenic epitopes associated with Burkholderia pseudomallei and Burkholderia mallei lipopolysaccharide O-antigens
Source: Nat Commun. 2017 Jul 24;8:115. doi: 10.1038/s41467-017-00173-8 (PMC5524647; doi:10.1038/s41467-017-00173-8)
Supplement: Supplementary file 1 — Supplementary Information [file 41467_2017_173_MOESM1_ESM.pdf]

File Name: Supplementary Information
Description: Supplementary Figures, Supplementary Tables, Supplementary Methods and Supplementary References

File Name: Peer Review File
Description:

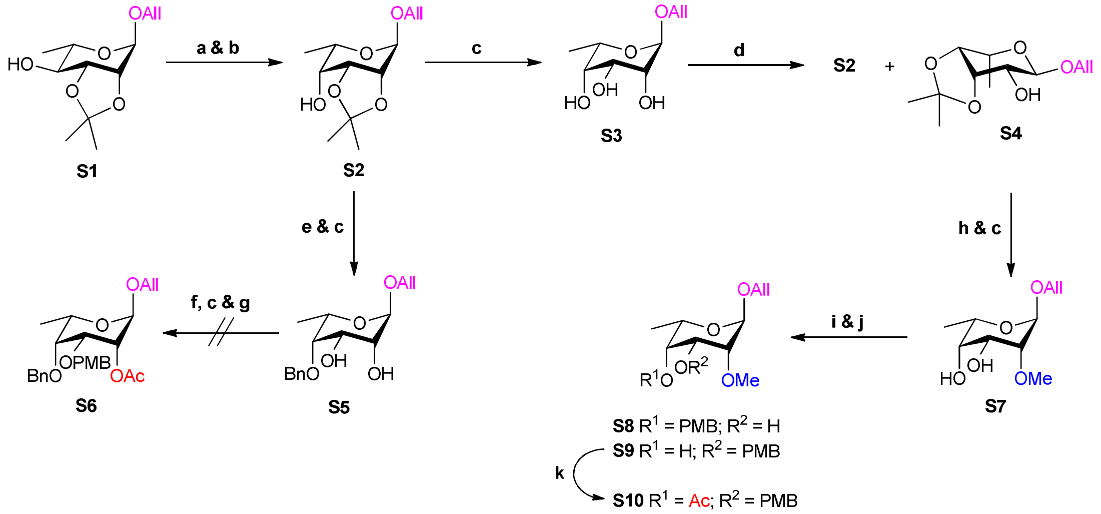

Supplementary Figure 1 │ Synthesis of 6-deoxy-L-talose derivatives. Reagents and conditions: (**a**) PDCP, DMSO, Et_3N, DCM, –10 °C to RT, 40 min; or Dess-Martin periodinane, DCE, reflux, 1 h; (**b**) $NaBH_4$, DCM/MeOH, –10 to 0 °C, 1 h, 65–70% (over two steps); (**c**) 80% HOAc, 60 °C, 3 h, 96% (for **S3**); 99% (for **S5**, over two steps); 92% (for **S7**, over two steps); (**d**) 2,2-DMP, PTSA, Me_2CO, RT, 2 h, 83% (for **S4**); 14% (for **S2**); (**e**) BnBr, NaH, DMF, 0 °C to RT, 2 h; (**f**) $MeC(OMe)_3$, PTSA, CH_3CN; (**g**) PMBCl, Ag_2CO_3, tol, 60 °C; or PMBCl, Ag_2O, Me_2S, TBAI, CH_3CN; or PMBTCA, Et_2O, TfOH; (**h**) MeI, NaH, TBAI, DMF, 0 °C to RT, 5 h; (**i**) Bu_2SnO, tol, reflux, 3 h; (**j**) PMBCl, CsF, TBAI, tol, 50 °C, overnight, 13% (for **S8**, over two steps); 60% (for **S9**, over two steps); (**k**) Ac_2O, py, DMAP, RT, overnight, 84%. Ac, acetyl; Ac_2O, acetic anhydride; All, allyl; Bn, benzyl; BnBr, benzyl bromide; Bu_2SnO, dibutyltin oxide; DCE, 1,2-dichloroethane; DCM, dichloromethane; DMAP, 4-(dimethylamino)pyridine; DMF, *N*,*N*-dimethylformamide; 2,2-DMP, 2,2-dimethoxypropane; DMSO, dimethylsulfoxide; Et_2O, diethyl ether; HOAc, acetic acid; $MeC(OMe)_3$, trimethyl orthoacetate; PDCP, phenyl dichlorophosphate; PMB, *para*-methoxybenzyl; PMBCl, *para*-methoxybenzyl chloride; PMBTCA, *para*-methoxybenzyl trichloroacetimidate; PTSA, *para*-toluenesulfonic acid; py, pyridine; RT, room temperature; TBAI, tetrabutylammonium iodide; TfOH, trifluoromethanesulfonic acid; tol, toluene.

Supplementary Table 1 | Regioselective protection of diols via stannylene acetal.

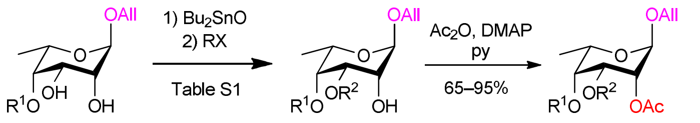

Entry	Compd	Reagents and conditions Step 1)[a]	Step 2)[a]	Product	Yield[b] (%)
1	**S3**	tol	PMBCl, TBAI, tol	**S11**	43
2	**S3**	tol	PMBCl, CsF, tol	**S11**	trace
3	**S3**	tol	PMBCl, TBAI, CsF, tol	**S11**	31
4	**S3**	MeOH	PMBCl, TBAI, CsF, tol	**S11**	65
5	**S3**	MeOH	MeI, CsF, tol[c]	**S12**	28
6	**S3**	MeOH	MeI, CsF, DMF[c]	**S12**	38
7	**S5**	tol	MeI, CsF, tol	**S14**	85
8	**S5**	tol	PMBCl, TBAI, tol	**S13**	56[d]

[a]The reaction was performed in refluxing toluene or MeOH.
[b]Isolated yield.
[c]The reaction was performed at 80 °C.
[d]The 2-*O*-PMB regioisomer was isolated as a minor compound (32% yield).

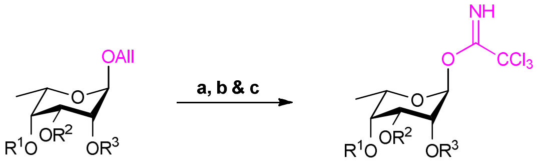

S6 R1 = Bn; R2 = PMB; R3 = Ac
S10 R1 = Ac; R2 = PMB; R3 = Me
S15 R1 = Ac; R2 = PMB; R3 = Ac
S17 R1 = Bn; R2 = Me; R3 = Ac
S16 R1 = Ac; R2 = Me; R3 = Ac

8 R1 = Bn; R2 = PMB; R3 = Ac
9 R1 = Ac; R2 = PMB; R3 = Me
10 R1 = Ac; R2 = PMB; R3 = Ac
11 R1 = Bn; R2 = Me; R3 = Ac
12 R1 = Ac; R2 = Me; R3 = Ac

Supplementary Figure 2 │ Synthesis of 6-deoxy-L-talopyranosyl trichloroacetimidate donors. Reagents and conditions: (**a**) [Ir(COD){PMe(C_6H_5)$_2$}$_2$]$^+$.PF_6^-, H_2, THF, RT, 1 h; (**b**) I_2, THF, H_2O, RT, 2 h, 66–89% (over two steps); (**c**) CCl_3CN, Cs_2CO_3, DCM/Me_2CO or DBU, DCM/Me_2CO, RT, 2–4 h, 58–91% (over two steps). CCl_3CN, trichloroacetonitrile; COD, cyclooctadienyl; DBU, 1,8-diazabicyclo[5.4.0]undec-7-ene; THF, tetrahydrofuran.

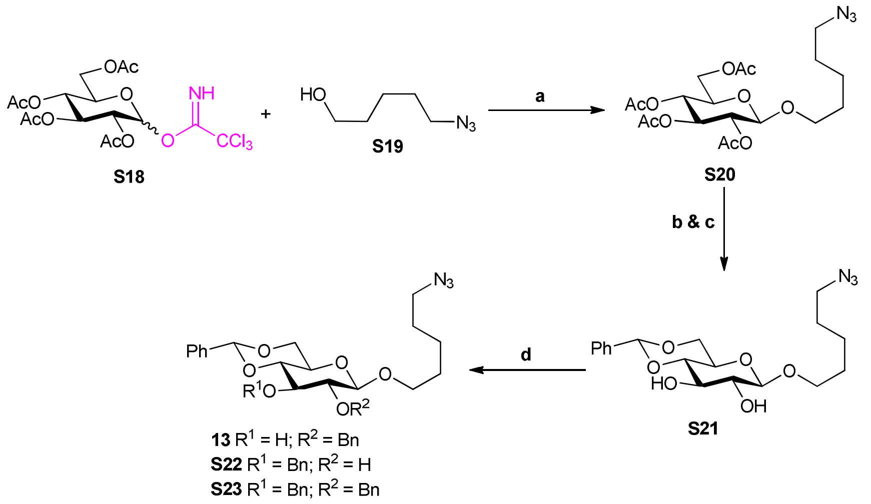

Supplementary Figure 3 | Synthesis of glucoside acceptor 13. Reagents and conditions: (**a**) TMSOTf, DCE, 4 Å MS, –10 °C to RT, overnight, 50%; (**b**) Et_3N, MeOH, RT, 48 h; (**c**) BDMA, CSA, CH_3CN, RT, 8 h, 78% (over two steps); (**d**) BnBr, TBAHS, 5% NaOH, DCM, reflux, 16 h, 55% (for **13**); 28% (for **S22**); 9% (for **S23**). BDMA, benzaldehyde dimethyl acetal; CSA, camphorsulfonic acid; Ph, phenyl; TBAHS, tetrabutylammonium hydrogenosulfonate; TMSOTf, trimethylsilyl trifluoromethanesulfonate.

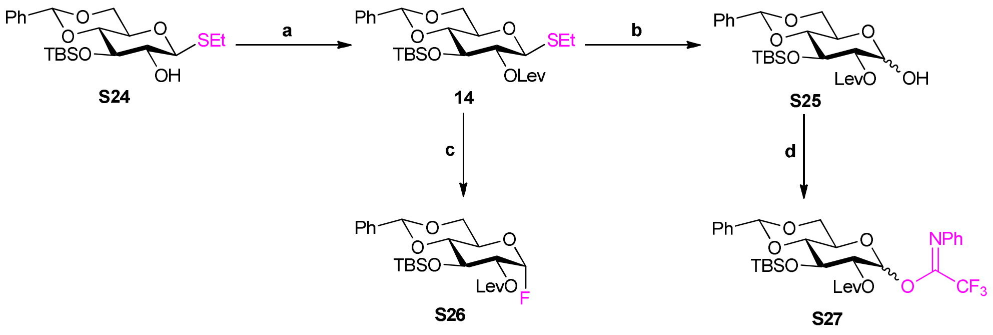

Supplementary Figure 4 │ Synthesis of glucosydonors 14, S26, and S27. Reagents and conditions: (**a**) Lev_2O, py, DMAP, 50 °C, 6 h, 83%; (**b**) NBS, DCM, H_2O, 0 °C to RT, 2 h, 66%; (**c**) NBS, DAST, DCM, –10 °C to RT, 2 h, 73%; (**d**) PTFACl, K_2CO_3, Me_2CO, RT, 7 h, 58%. DAST, diethylaminosulfur trifluoride; Lev, levulinoyl; Lev_2O, levulinic anhydride; NBS, *N*-bromosuccinimide; PTFACl, *N*-phenyl-2,2,2-trifluoroacetimidoyl chloride; SEt, thioethyl; TBS, *tert*-butyldimethylsilyl.

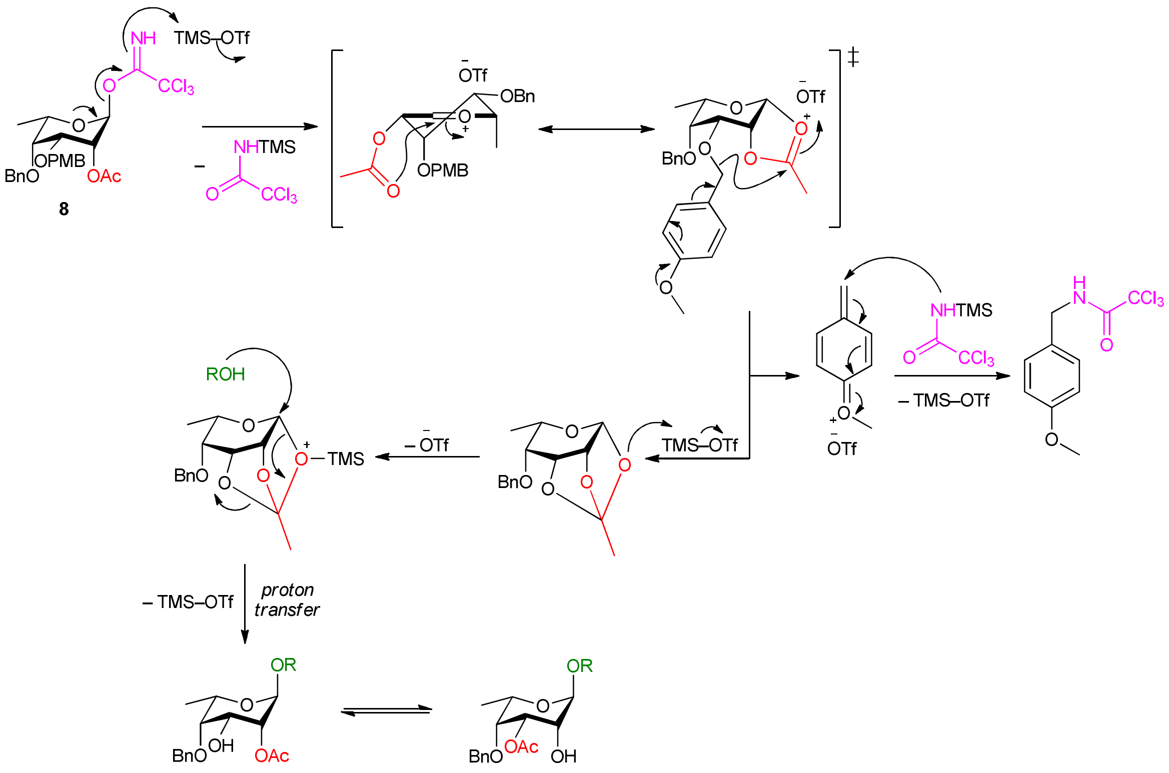

Supplementary Figure 5 | Proposed mechanism for the cleavage of PMB group during glycosylation. Formation of derivative **20** can be tentatively explained via a mechanism in which a transient tricylic orthoester intermediate is attacked by the alcohol acceptor on the α-side. The resulting kinetic product **20** can then be transformed into the more stable derivative **27** by migration of the acetyl group from the C2 to C3 position.

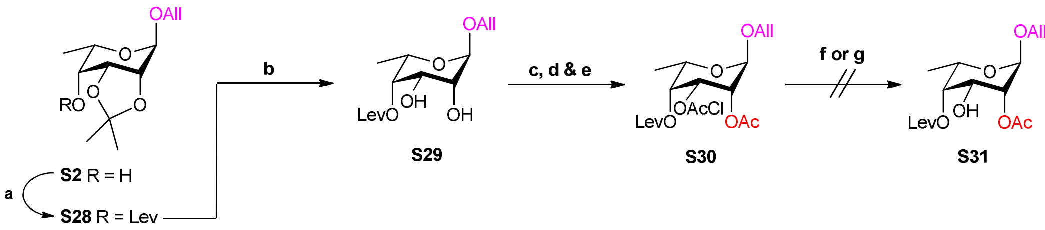

Supplementary Figure 6 │ Attempts to synthesize taloside acceptor S31. Reagents and conditions: (**a**) Lev_2O, py, DMAP, 50 °C, 2 h, 95%; (**b**) 80% HOAc, 60 °C, 1 h; (**c**) $MeC(OMe)_3$, PTSA, CH_3CN, RT, 2 h; (**d**) 80% HOAc, 0 °C to RT, 2 h; (**e**) $(ClAc)_2O$, py, DMAP, RT, 10 min, 83% (over four steps from **S28**); (**f**) DABCO, 55 °C, EtOH/py 5:1; (**g**) TBAF, THF, RT. $(ClAc)_2O$, chloroacetic anhydride; DABCO, 1,4-diazabicyclo[2.2.2]octane; TBAF, tetrabutylammonium fluoride.

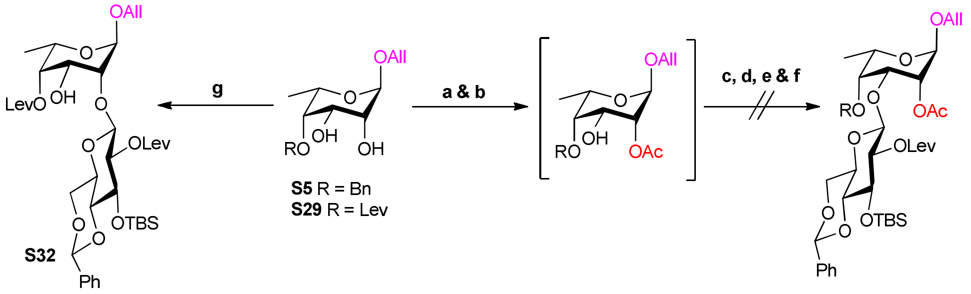

Supplementary Figure 7 │ Attempts to synthesize (1→3)-linked disaccharides. Reagents and conditions: (**a**) MeC(OMe)$_3$, PTSA, CH_3CN, RT, 2 h; (**b**) 80% HOAc, 0 °C to RT, 2 h; (**c**) donor **14**, NIS, AgOTf, 4 Å MS, Et_2O, –10 °C; (**d**) donor **14**, DMTST, DTBMP, 4 Å MS, Et_2O, –10 to 40 °C; (**e**) donor **S26**, Cp_2ZrCl_2, AgOTf, 4 Å MS, Et_2O, –10 °C; (**f**) donor **S27**, TMSOTf, DCE, 4 Å MS, –10 °C; (**g**) donor **14**, DMTST, DTBMP, 4 Å MS, DCE, RT, 2 h, 43%. AgOTf, silver(I) trifluoromethanesulfonate; Cp_2ZrCl_2, bis(cyclopentadienyl)zirconium(IV) dichloride; DMTST, dimethyl(methylthio)sulfonium trifluoromethanesulfonate; DTBMP, 2,6-di-*tert*-butyl-4-methylpyridine; MS, molecular sieves; NIS, *N*-iodosuccinimide.

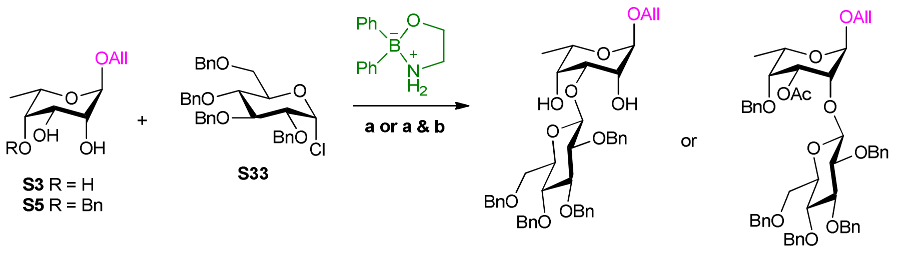

Supplementary Figure 8 │ Glucosylation of diol S5 and triol S3 using Taylor catalyst. Reagents and conditions: (**a**) 2-aminoethyl diphenylborinate (0.25 equiv), Ag_2O, CH_3CN, 60 °C, 16–48 h, 25% (for **S34**); (**b**) Ac_2O, py, DMAP, RT, overnight, 58% (for **S35** over two steps).

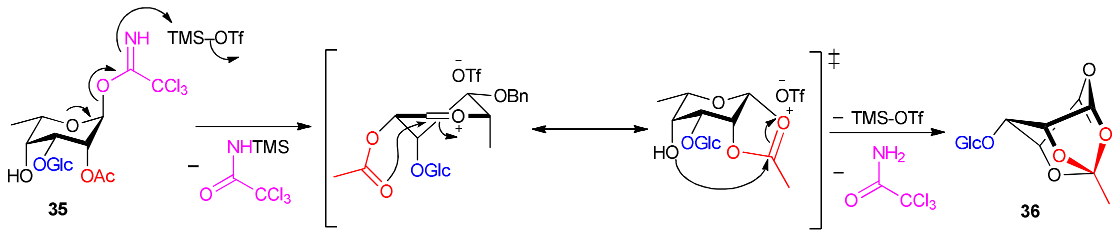

Supplementary Figure 9 │ Proposed mechanism for the formation of tricyclic orthoester 36. Formation of derivative **36** can be tentatively explained by the attack of free alcohol C4 to the carbonyl group of the dioxalenium intermediate.

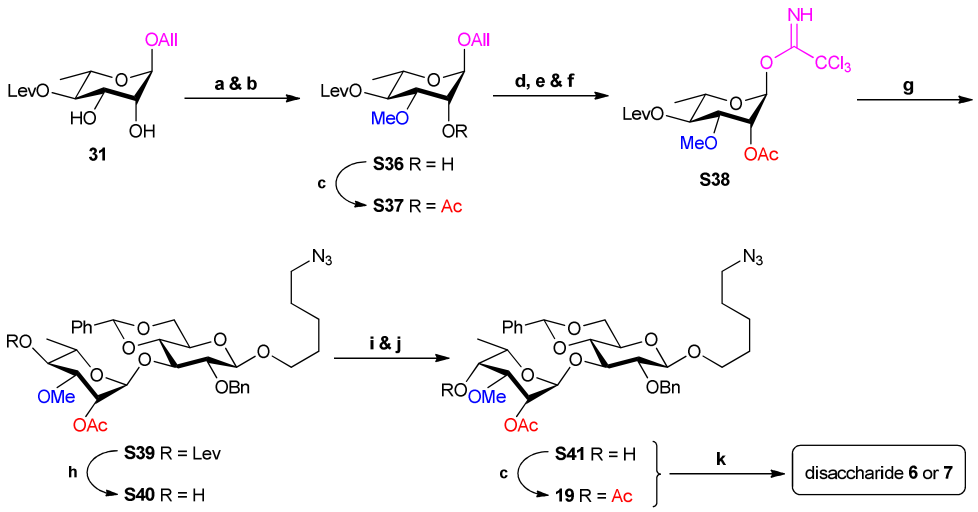

Supplementary Figure 10 │ Second generation synthesis of target disaccharides 6 and 7. Reagents and conditions: (**a**) Bu_2SnO, tol, reflux, 5 h; (**b**) MeI, CsF, tol, 80 °C, overnight, 96% (over two steps); (**c**) Ac_2O, py, DMAP, RT, 16 h, 88% (for **S37**); 65% (for **19**); (**d**) $[Ir(COD)\{PMe(C_6H_5)_2\}_2]^+.PF_6^-$, H_2, THF; (**e**) I_2, THF, H_2O; (**f**) CCl_3CN, Cs_2CO_3, Me_2CO, 87% (over three steps); (**g**) acceptor **13**, TMSOTf (0.1 equiv), 4 Å MS, Et_2O/DCE 5:1, –10 °C to RT, 30 min, 92%; (**h**) $H_2NNH_2.H_2O$, py, HOAc, 0 °C to RT, overnight, 99%; (**i**) PDCP, DMSO, Et_3N, DCM, –10 °C to RT, 1 h; (**j**) $NaBH_4$, MeOH/DCM 3:1, –10 °C to RT, 1 h, 66% (over two steps); (**k**) Pd black, H_2, HCl (1.0 equiv), MeOH, DCM, 40 °C, quant. (for **6** and **7**).

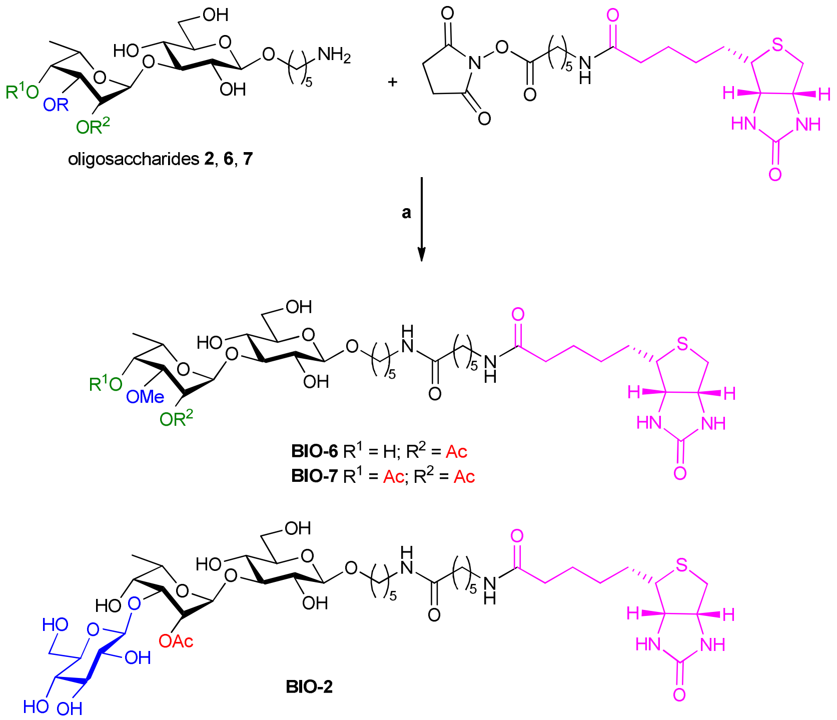

Supplementary Figure 11 │ Synthesis of biotinylated oligosaccharides. Reagents and conditions: (**a**) Et_3N, DMF, H_2O, rt, 1 h, 69% (for **BIO-6**); 69% (for **BIO-7**); 55% (for **BIO-2**).

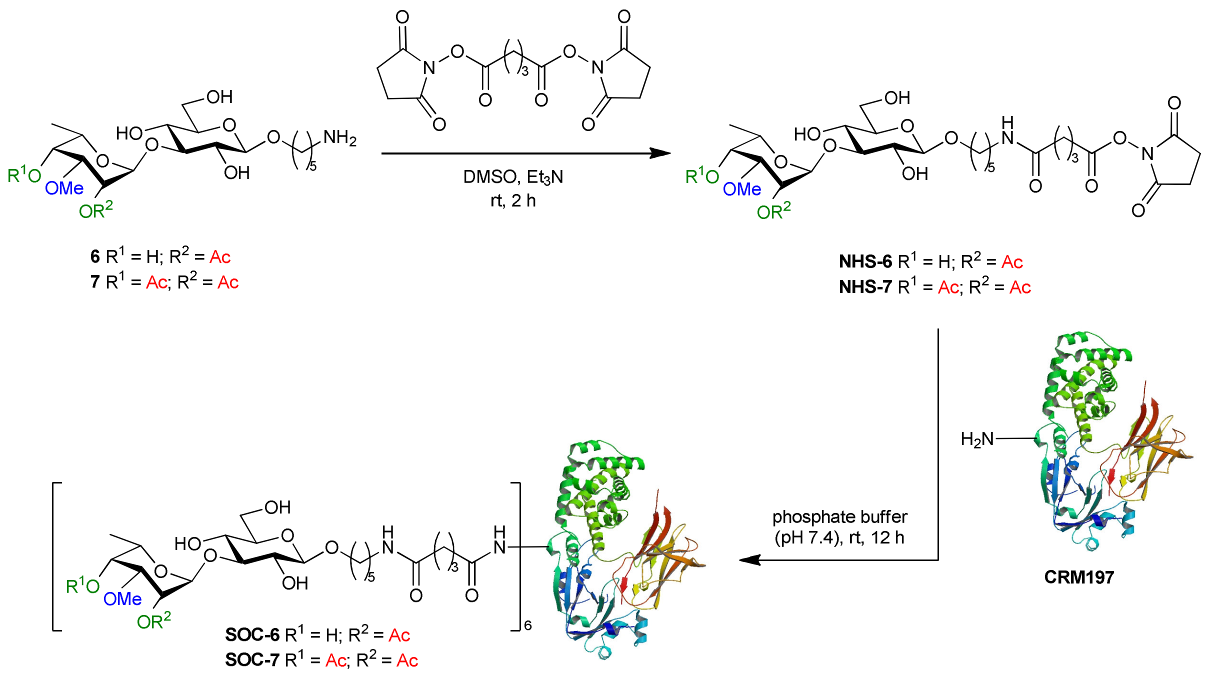

Supplementary Figure 12 │ Synthesis of disaccharide:CRM197 conjugates SOC-6 and SOC-7. Disaccharide **6** or **7** was reacted with disuccinimidyl glutarate to generate derivative **NHS-6** or **NHS-7**, respectively, which upon reaction with CRM197 led to the formation of glycoconjugate vaccine **SOC-6** or **SOC-7**, respectively.

Supplementary Figure 13 | ^{1}H NMR spectra ($CDCl_3$, 400 MHz) of compound S2.

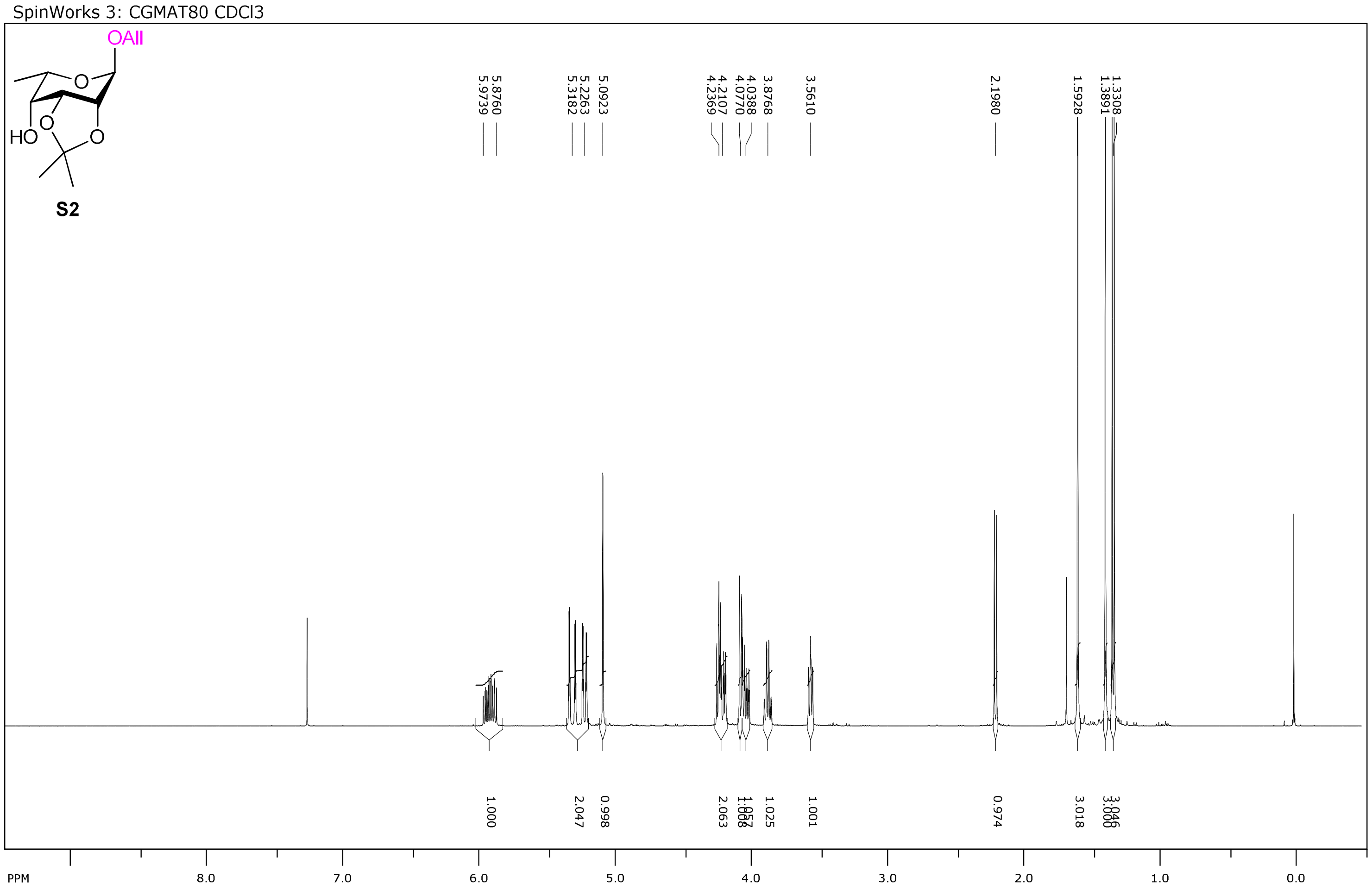

file: D:\RMN LPS\CGMAT80\1\fid expt: <zg30>
transmitter freq.: 400.132001 MHz
time domain size: 32768 points
width: 4807.69 Hz = 12.0153 ppm = 0.146719 Hz/pt
number of scans: 16

freq. of 0 ppm: 400.130006 MHz
processed size: 32768 complex points
LB: 0.300 GF: 0.0000
Hz/cm: 160.052 ppm/cm: 0.40000

Supplementary Figure 14 │ ^{13}C NMR spectra ($CDCl_3$, 100 MHz) of compound S2.

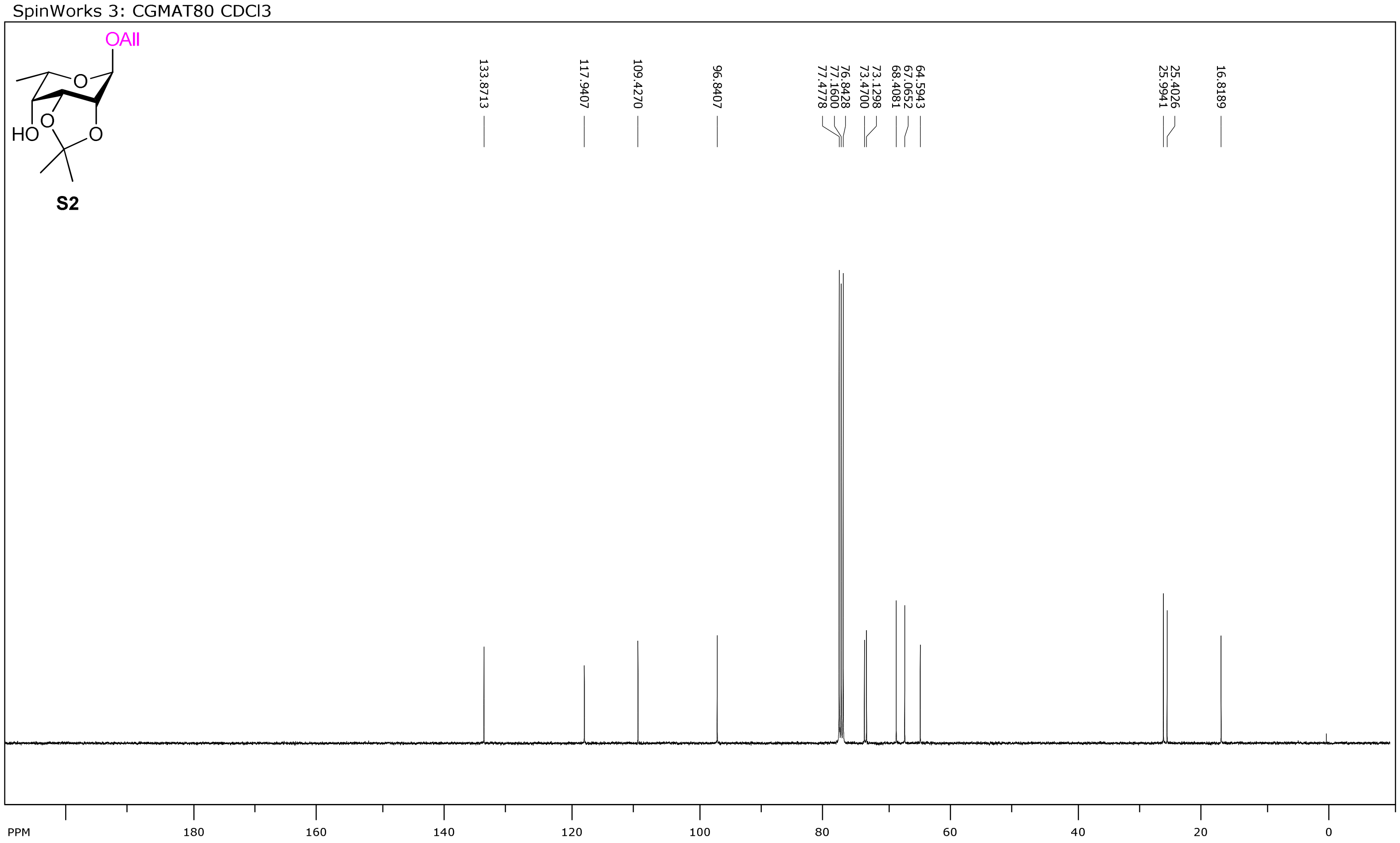

file: D:\RMN LPS\CGMAT80\4\fid expt: <zgpg30>
transmitter freq.: 100.622830 MHz
time domain size: 65536 points
width: 24038.46 Hz = 238.8967 ppm = 0.366798 Hz/pt
number of scans: 1024

freq. of 0 ppm: 100.612754 MHz
processed size: 32768 complex points
LB: 1.000 GF: 0.0000
Hz/cm: 885.392 ppm/cm: 8.79912

Supplementary Figure 15 │ ^{1}H NMR spectra ($CDCl_3$, 400 MHz) of compound S3.

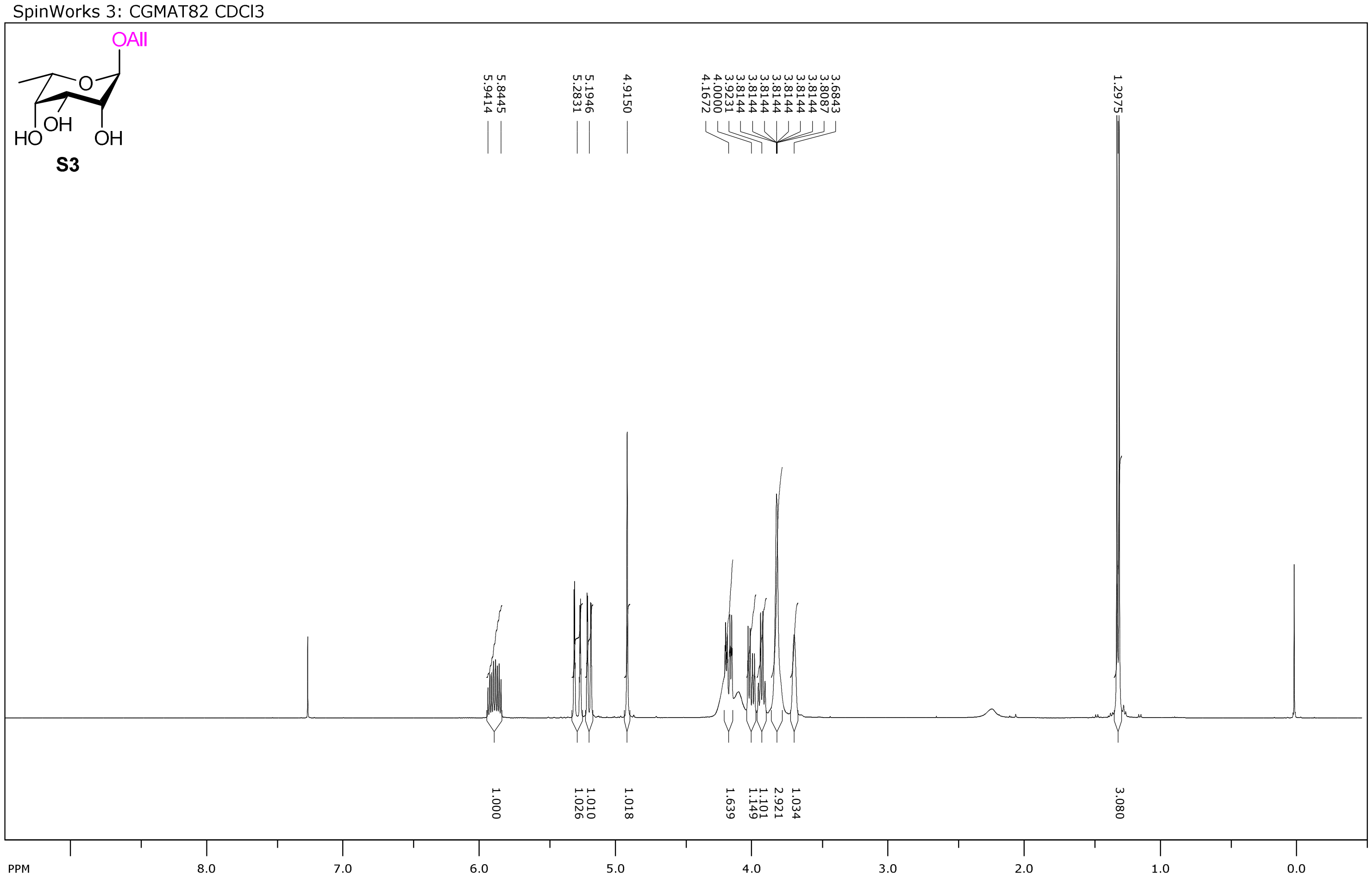

file: D:\RMN LPS\CGMAT82\2\fid expt: <zg30>
transmitter freq.: 400.132001 MHz
time domain size: 32768 points
width: 4807.69 Hz = 12.0153 ppm = 0.146719 Hz/pt
number of scans: 64

freq. of 0 ppm: 400.130006 MHz
processed size: 32768 complex points
LB: 0.300 GF: 0.0000
Hz/cm: 160.052 ppm/cm: 0.40000

Supplementary Figure 16 | ^{13}C NMR spectra ($CDCl_3$, 100 MHz) of compound S3.

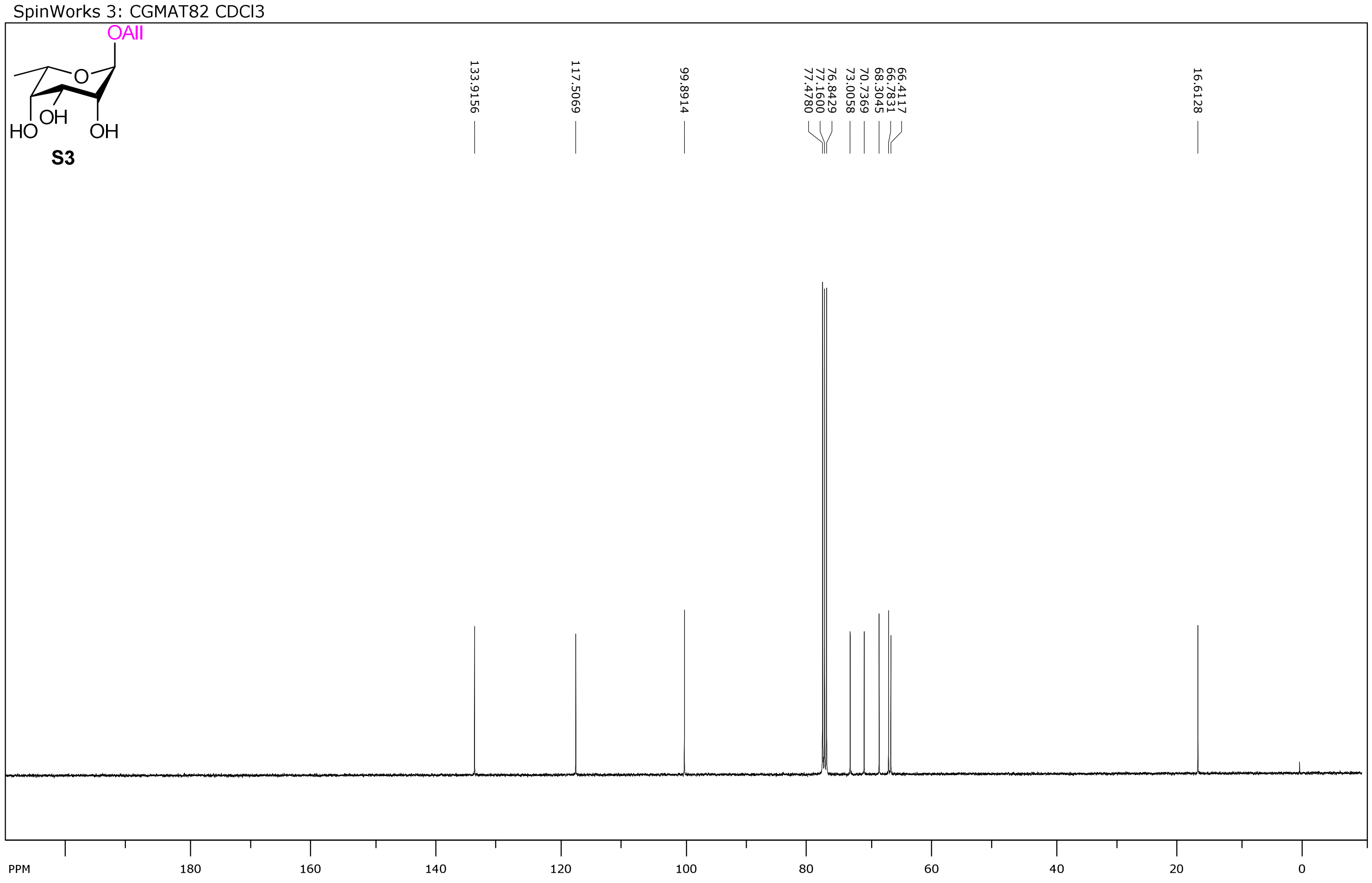

file: D:\RMN LPS\CGMAT82\4\fid expt: <zgpg30>
transmitter freq.: 100.622830 MHz
time domain size: 65536 points
width: 24038.46 Hz = 238.8967 ppm = 0.366798 Hz/pt
number of scans: 1024

freq. of 0 ppm: 100.612754 MHz
processed size: 32768 complex points
LB: 1.000 GF: 0.0000
Hz/cm: 885.392 ppm/cm: 8.79912

Supplementary Figure 17 │ ^{1}H NMR spectra ($CDCl_3$, 400 MHz) of compound S4.

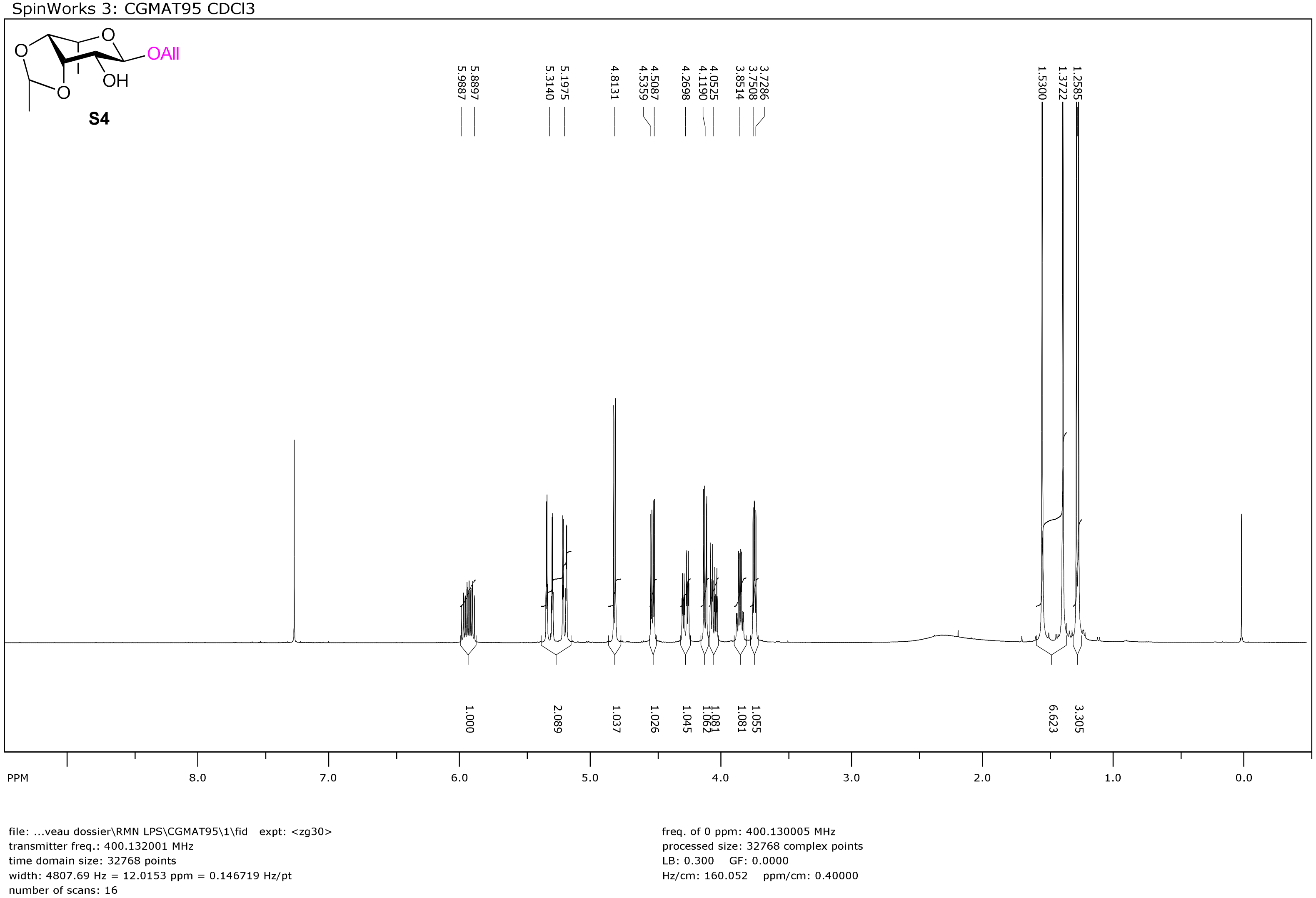

Supplementary Figure 18 │ ^{13}C NMR spectra ($CDCl_3$, 100 MHz) of compound S4.

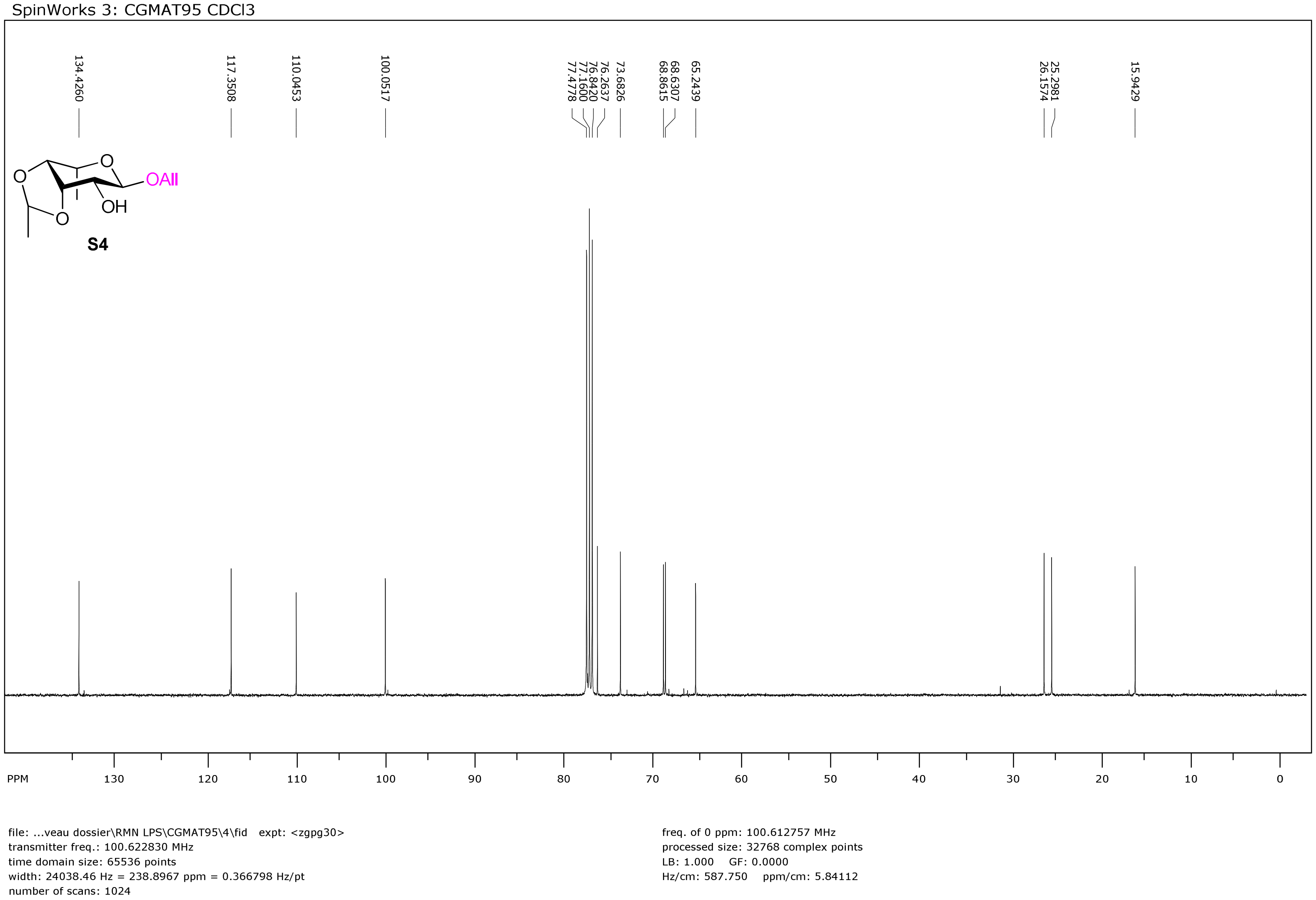

Supplementary Figure 19 | ^{1}H NMR spectra ($CDCl_3$, 400 MHz) of compound S5.

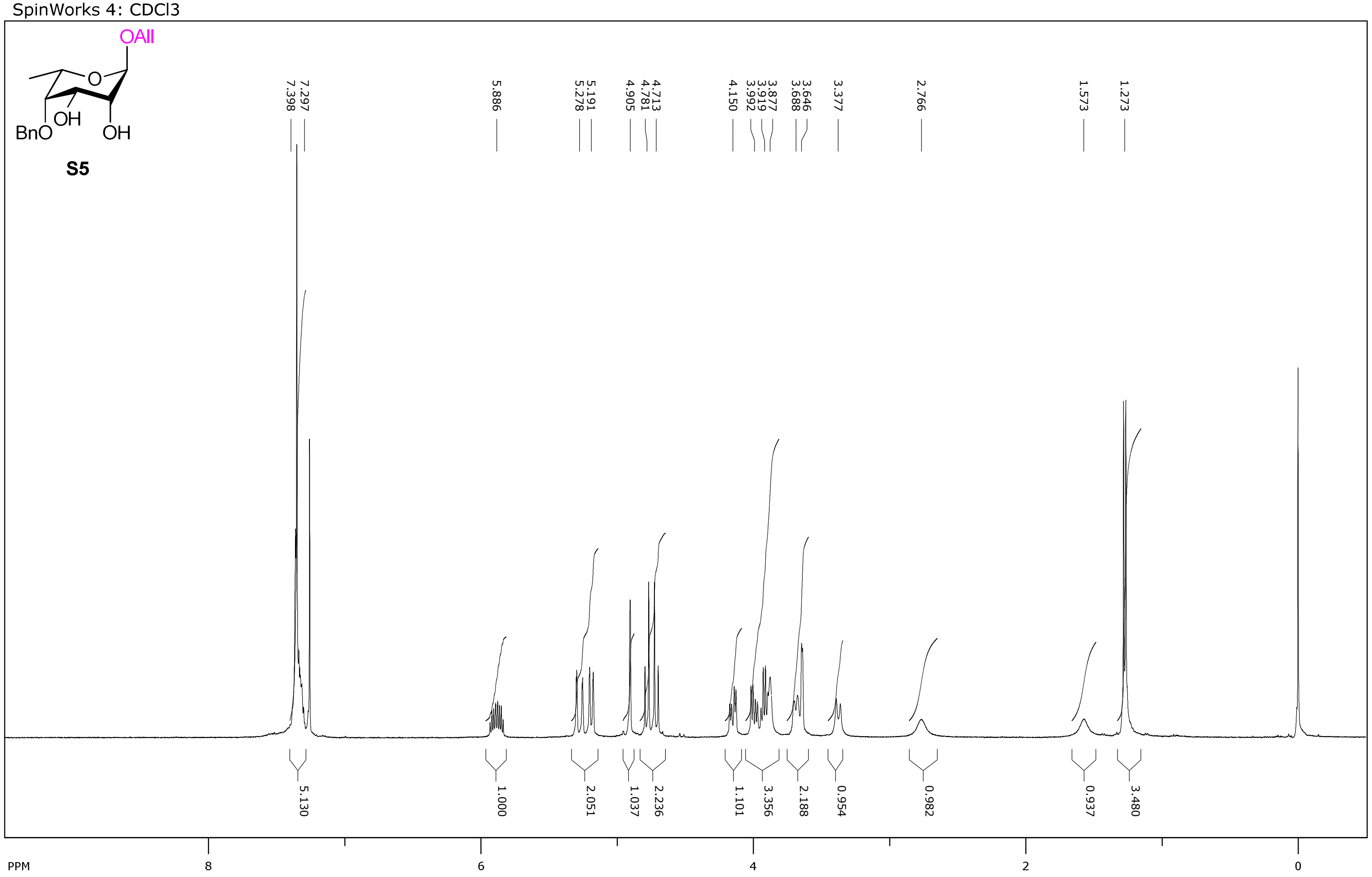

file: ... LPS Marielle\RMN LPS\CGAB28\1\fid expt: <zg30>
transmitter freq.: 400.132001 MHz
time domain size: 32768 points
width: 4807.69 Hz = 12.0153 ppm = 0.146719 Hz/pt
number of scans: 16

freq. of 0 ppm: 400.130010 MHz
processed size: 32768 complex points
LB: 0.300 GF: 0.0000

Supplementary Figure 20 | ^{13}C NMR spectra ($CDCl_3$, 100 MHz) of compound S5.

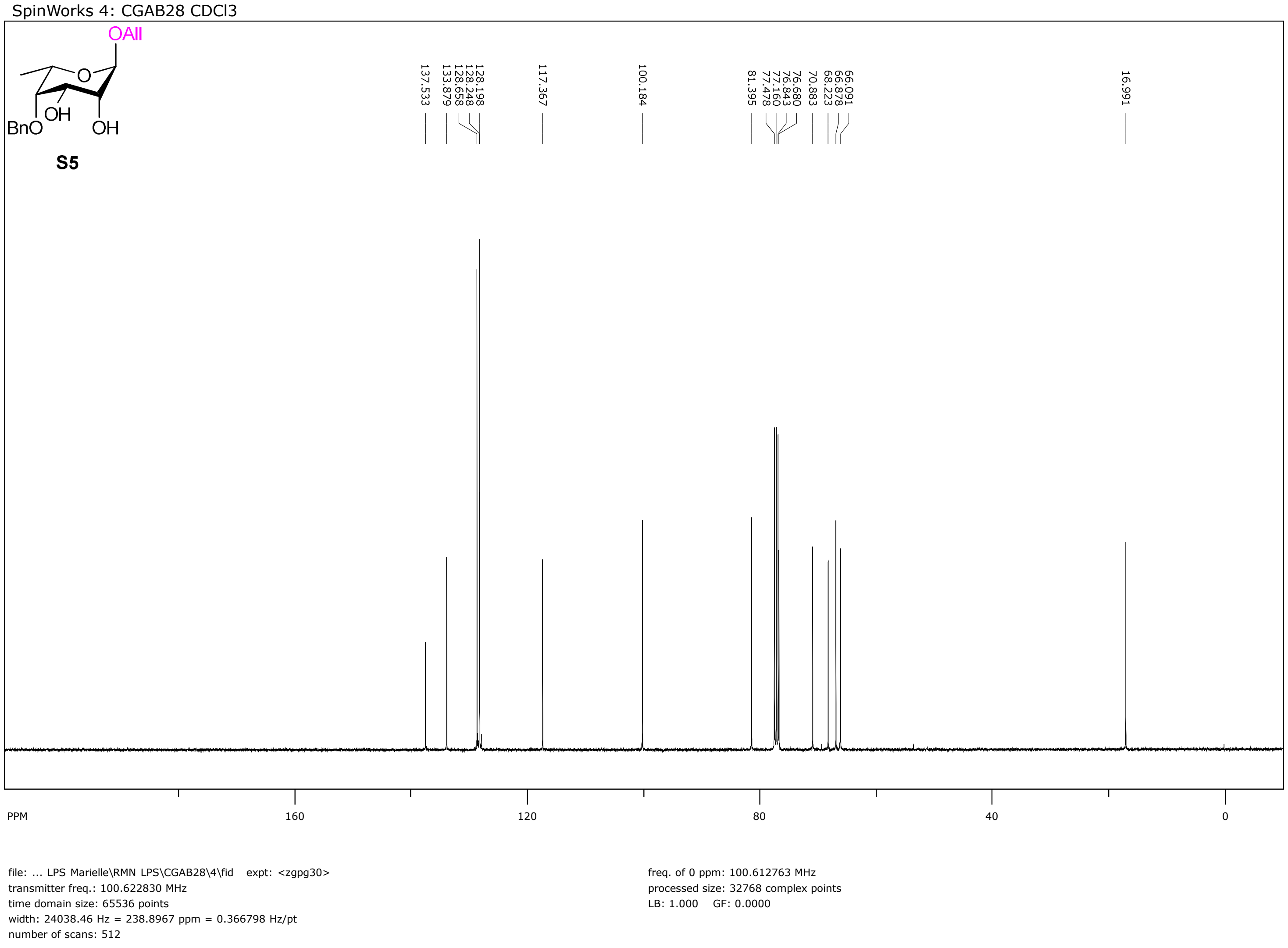

Supplementary Figure 21 | ^{1}H NMR spectra ($CDCl_3$, 400 MHz) of compound S7.

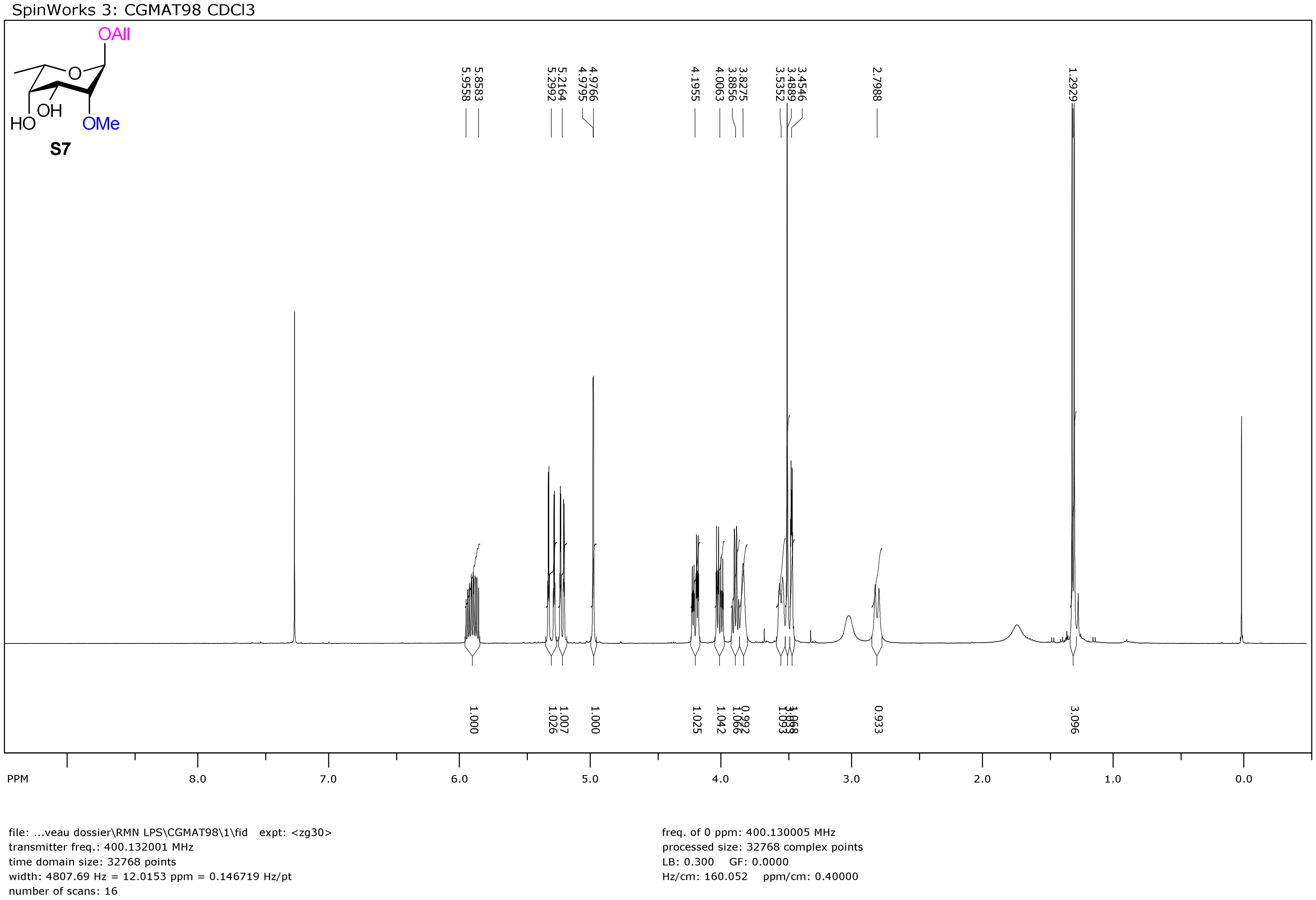

Supplementary Figure 22 | ^{13}C NMR spectra ($CDCl_3$, 100 MHz) of compound S7.

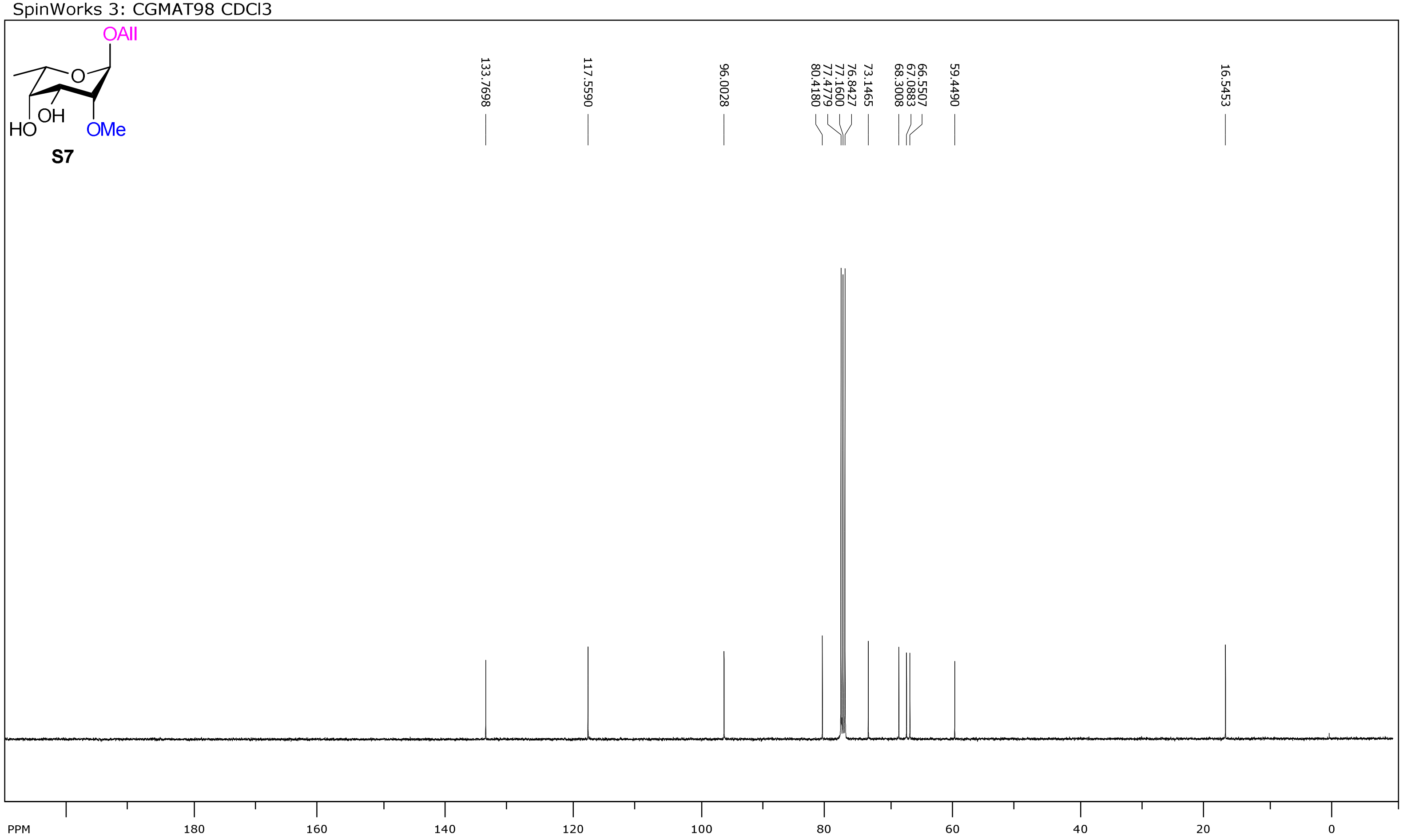

file: ...veau dossier\RMN LPS\CGMAT98\4\fid expt: <zgpg30>
transmitter freq.: 100.622830 MHz
time domain size: 65536 points
width: 24038.46 Hz = 238.8967 ppm = 0.366798 Hz/pt
number of scans: 1024

freq. of 0 ppm: 100.612755 MHz
processed size: 32768 complex points
LB: 1.000 GF: 0.0000
Hz/cm: 885.392 ppm/cm: 8.79912

Supplementary Figure 23 | ^{1}H NMR spectra ($CDCl_3$, 400 MHz) of compound S8.

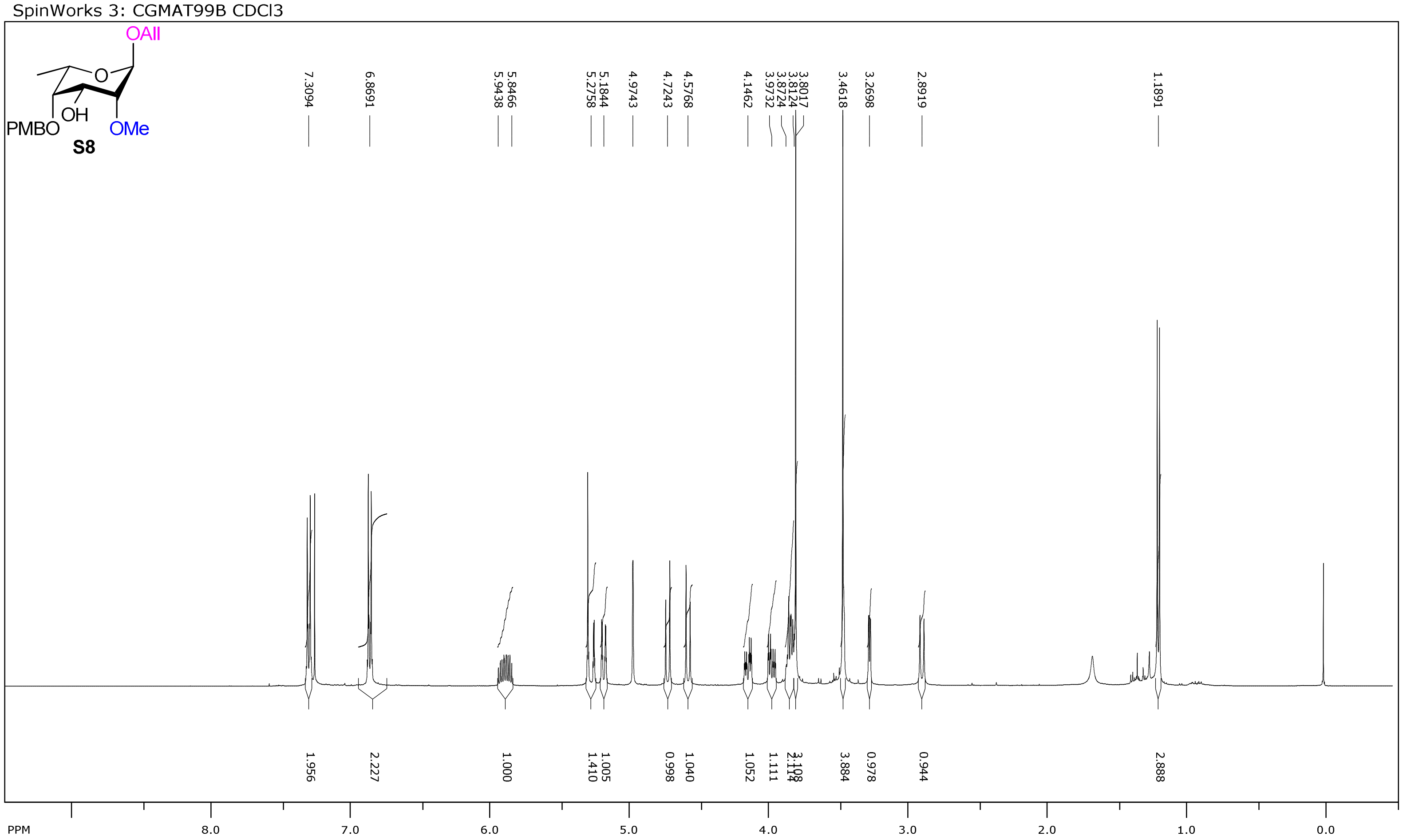

file: ...eau dossier\RMN LPS\CGMAT99B\1\fid expt: <zg30>
transmitter freq.: 400.132001 MHz
time domain size: 32768 points
width: 4807.69 Hz = 12.0153 ppm = 0.146719 Hz/pt
number of scans: 64

freq. of 0 ppm: 400.130007 MHz
processed size: 32768 complex points
LB: 0.300 GF: 0.0000
Hz/cm: 160.052 ppm/cm: 0.40000

Supplementary Figure 24 | ^{13}C NMR spectra ($CDCl_3$, 100 MHz) of compound S8.

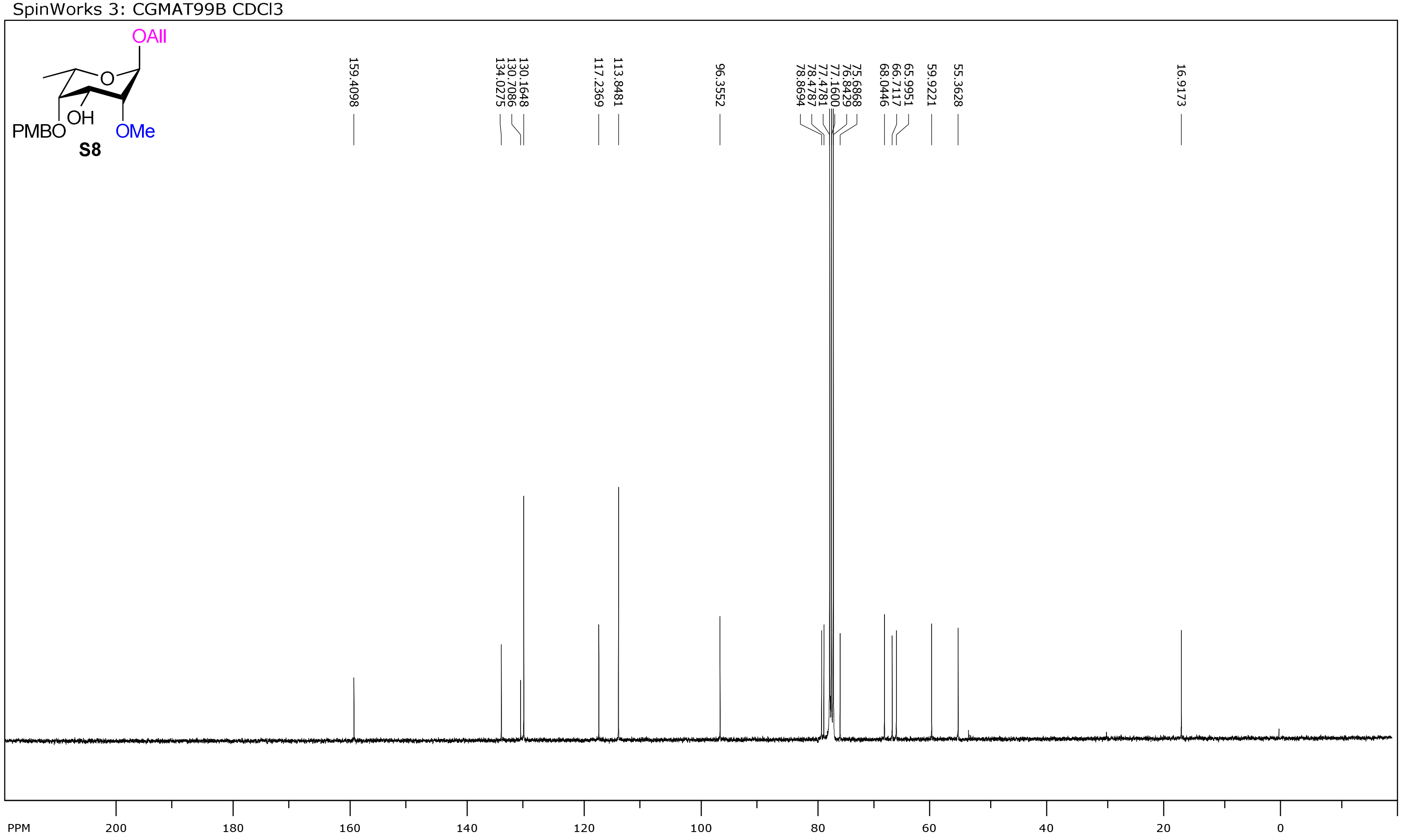

file: ...eau dossier\RMN LPS\CGMAT99B\3\fid expt: <zgpg30>
transmitter freq.: 100.622830 MHz
time domain size: 65536 points
width: 24038.46 Hz = 238.8967 ppm = 0.366798 Hz/pt
number of scans: 1024

freq. of 0 ppm: 100.612757 MHz
processed size: 32768 complex points
LB: 1.000 GF: 0.0000
Hz/cm: 961.538 ppm/cm: 9.55587

Supplementary Figure 25 │ ^{1}H NMR spectra ($CDCl_3$, 400 MHz) of compound S9.

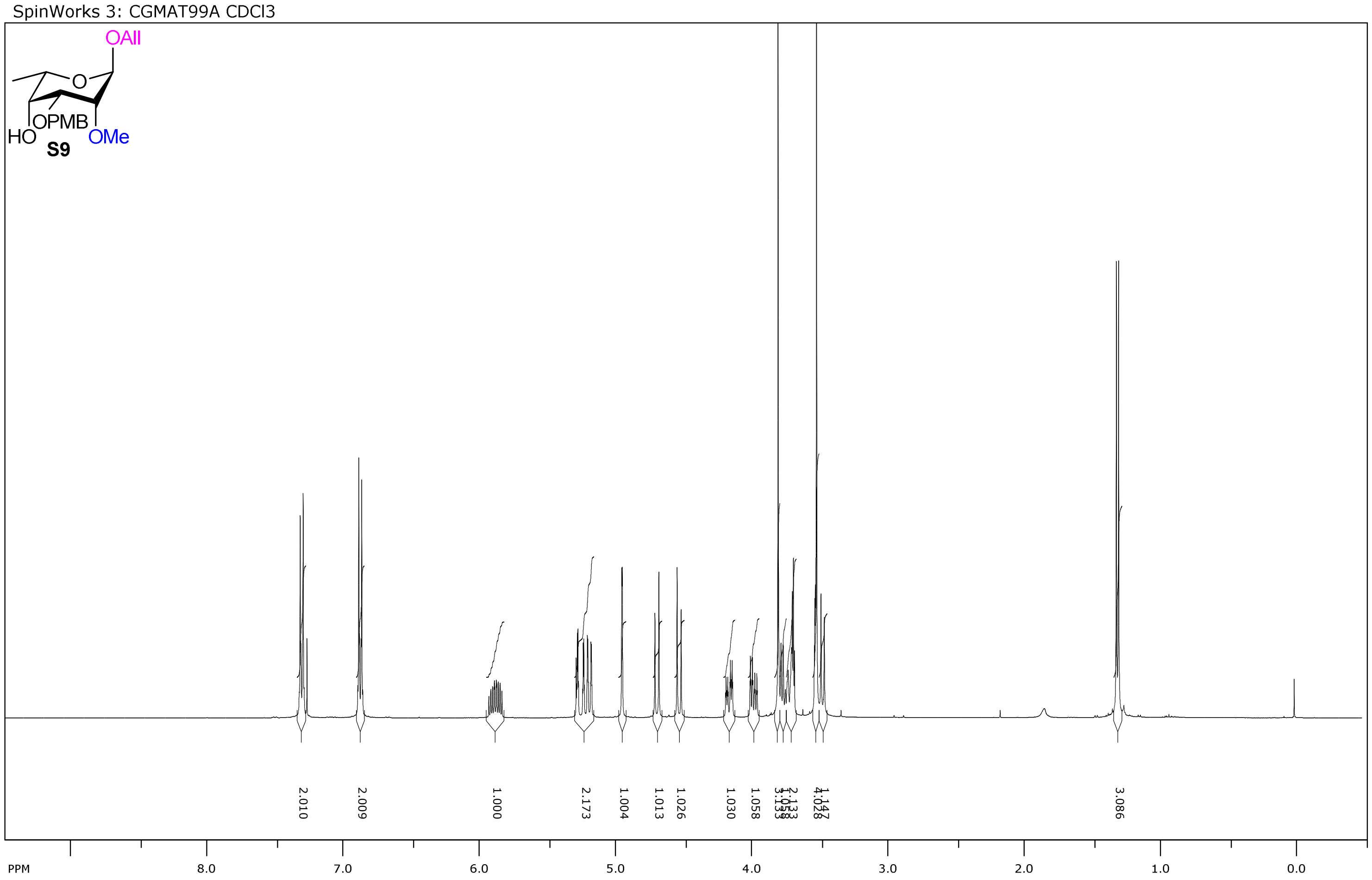

file: F:\CGMAT99A\1\fid expt: <zg30>
transmitter freq.: 400.132001 MHz
time domain size: 32768 points
width: 4807.69 Hz = 12.0153 ppm = 0.146719 Hz/pt
number of scans: 64

freq. of 0 ppm: 400.130004 MHz
processed size: 32768 complex points
LB: 0.300 GF: 0.0000
Hz/cm: 160.052 ppm/cm: 0.40000

Supplementary Figure 26 | ^{13}C NMR spectra ($CDCl_3$, 100 MHz) of compound S9.

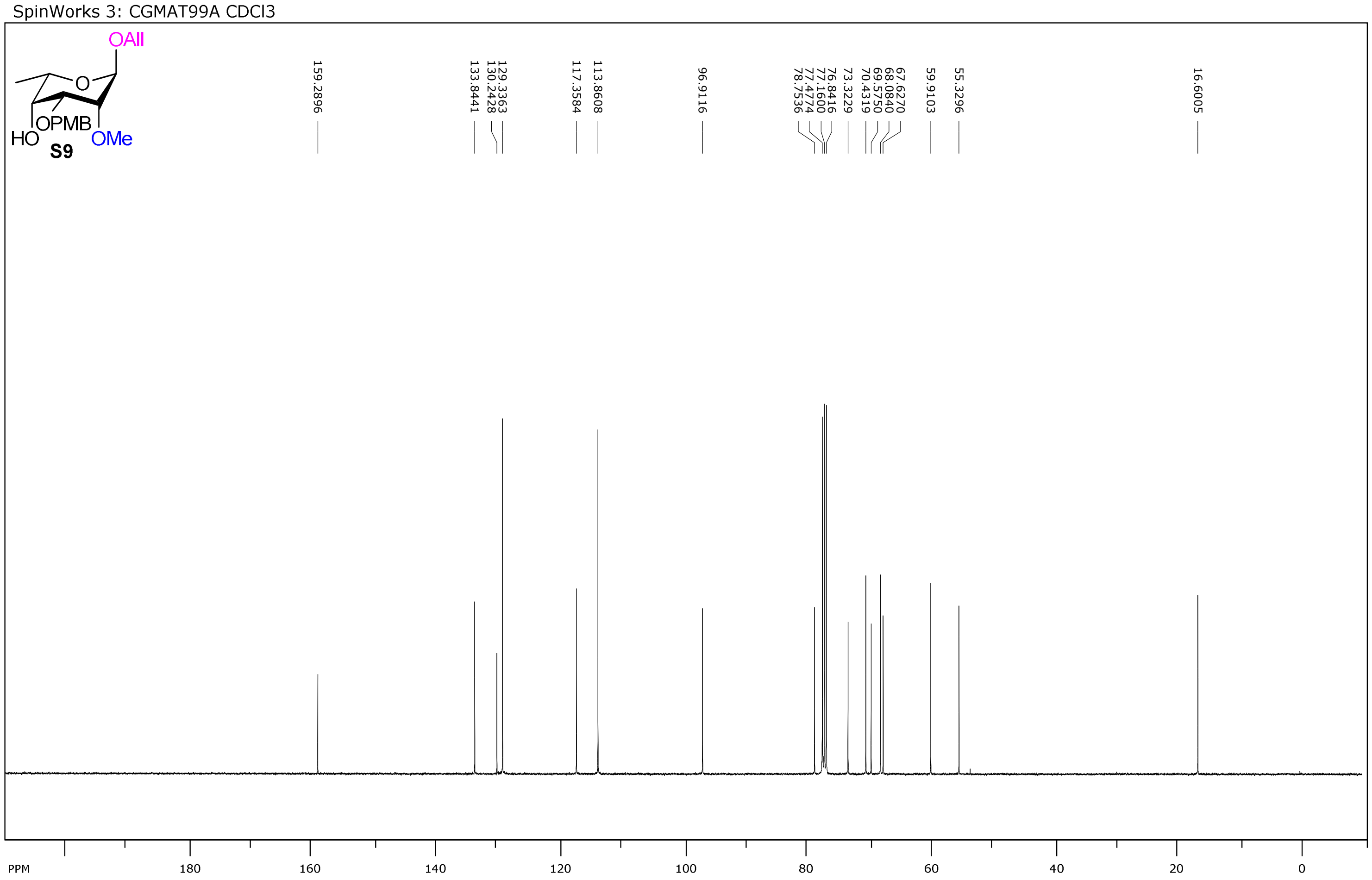

file: F:\CGMAT99A\3\fid expt: <zgpg30>
transmitter freq.: 100.622830 MHz
time domain size: 65536 points
width: 24038.46 Hz = 238.8967 ppm = 0.366798 Hz/pt
number of scans: 1024

freq. of 0 ppm: 100.612762 MHz
processed size: 32768 complex points
LB: 1.000 GF: 0.0000
Hz/cm: 885.392 ppm/cm: 8.79912

Supplementary Figure 27 | ^{1}H NMR spectra ($CDCl_3$, 400 MHz) of compound S10.

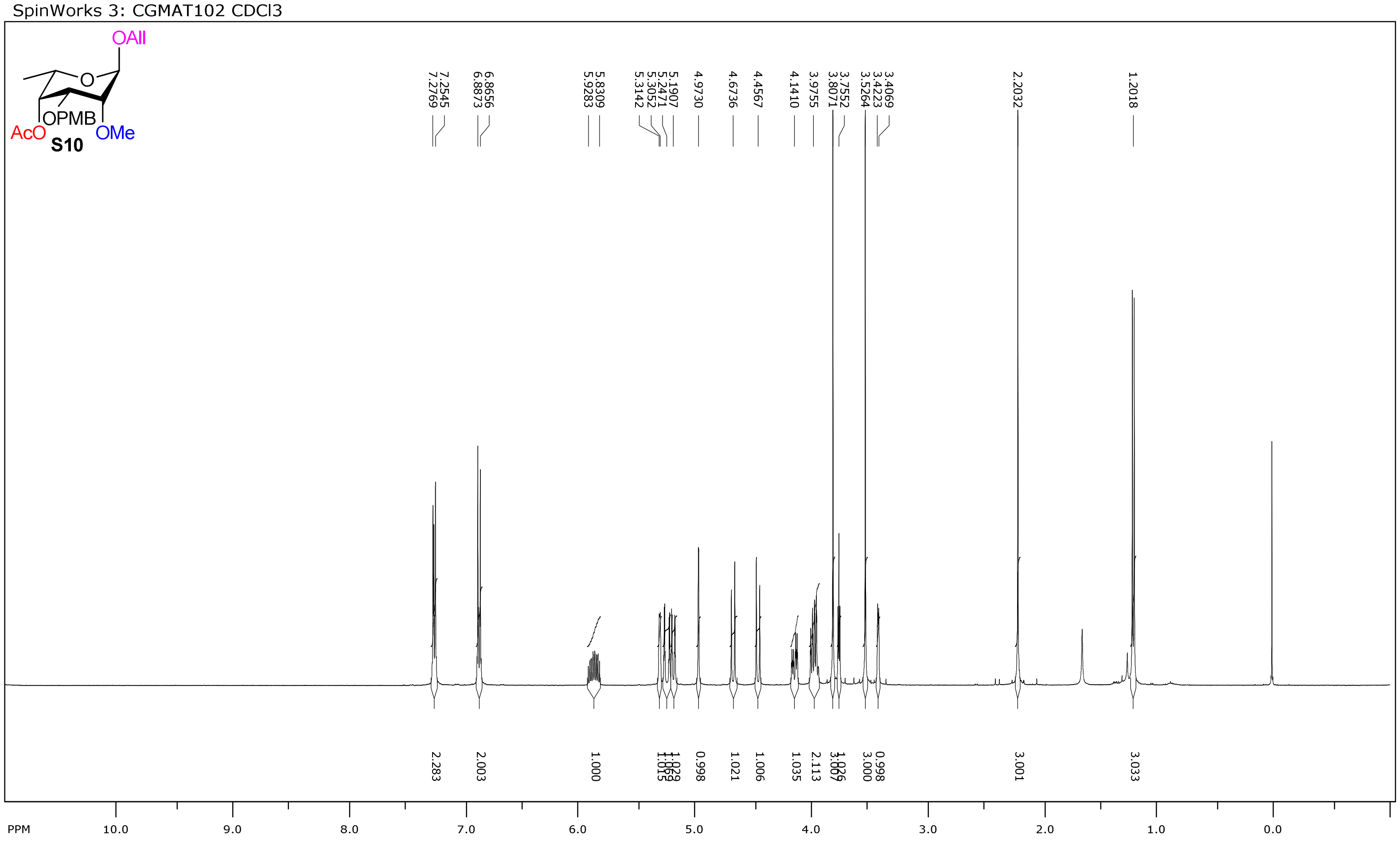

file: ...eau dossier\RMN LPS\CGMAT102\1\fid expt: <zg30>
transmitter freq.: 400.132001 MHz
time domain size: 32768 points
width: 4807.69 Hz = 12.0153 ppm = 0.146719 Hz/pt
number of scans: 4

freq. of 0 ppm: 400.130007 MHz
processed size: 32768 complex points
LB: 0.300 GF: 0.0000
Hz/cm: 192.308 ppm/cm: 0.48061

Supplementary Figure 28 | ^{13}C NMR spectra ($CDCl_3$, 100 MHz) of compound S10.

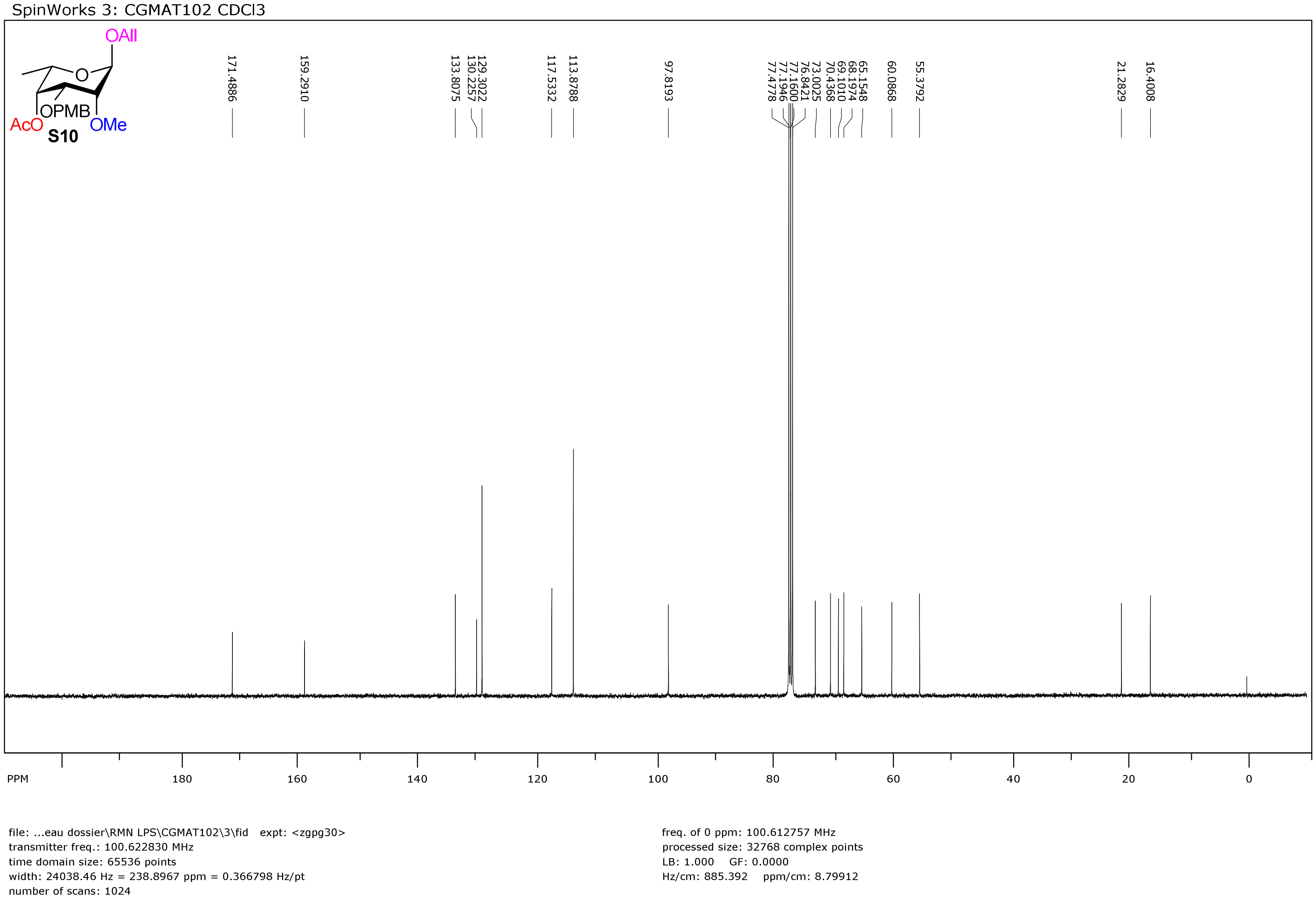

Supplementary Figure 29 | ^{1}H NMR spectra ($CDCl_3$, 400 MHz) of compound S11.

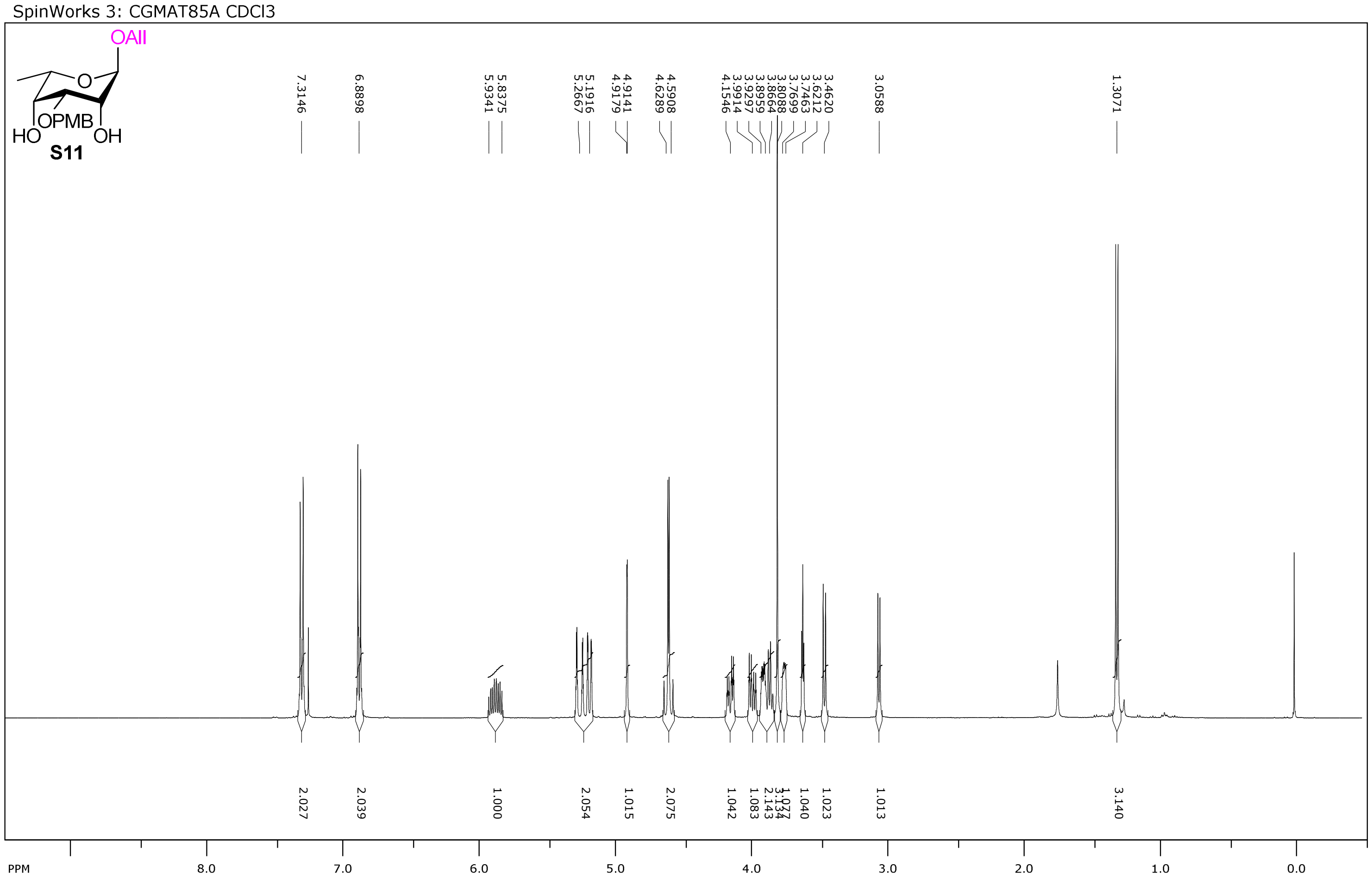

file: D:\RMN LPS\CGMAT85A\1\fid expt: <zg30>
transmitter freq.: 400.132001 MHz
time domain size: 32768 points
width: 4807.69 Hz = 12.0153 ppm = 0.146719 Hz/pt
number of scans: 16

freq. of 0 ppm: 400.130009 MHz
processed size: 32768 complex points
LB: 0.300 GF: 0.0000
Hz/cm: 160.052 ppm/cm: 0.40000

Supplementary Figure 30 | ^{13}C NMR spectra ($CDCl_3$, 100 MHz) of compound S11.

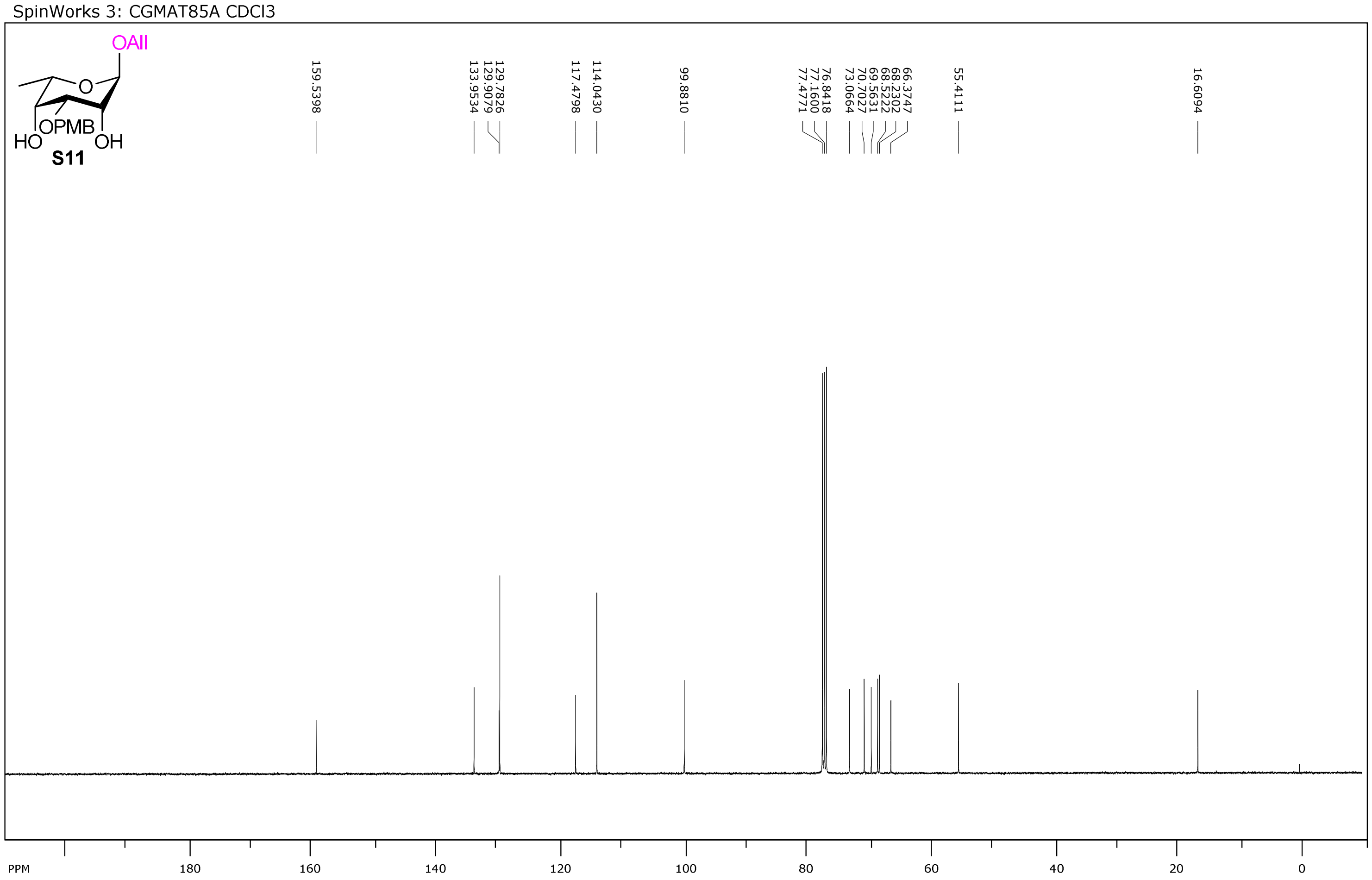

file: D:\RMN LPS\CGMAT85A\4\fid expt: <zgpg30>
transmitter freq.: 100.622830 MHz
time domain size: 65536 points
width: 24038.46 Hz = 238.8967 ppm = 0.366798 Hz/pt
number of scans: 1024

freq. of 0 ppm: 100.612757 MHz
processed size: 32768 complex points
LB: 1.000 GF: 0.0000
Hz/cm: 885.392 ppm/cm: 8.79912

Supplementary Figure 31 | ^{1}H NMR spectra ($CDCl_3$, 400 MHz) of compound S12.

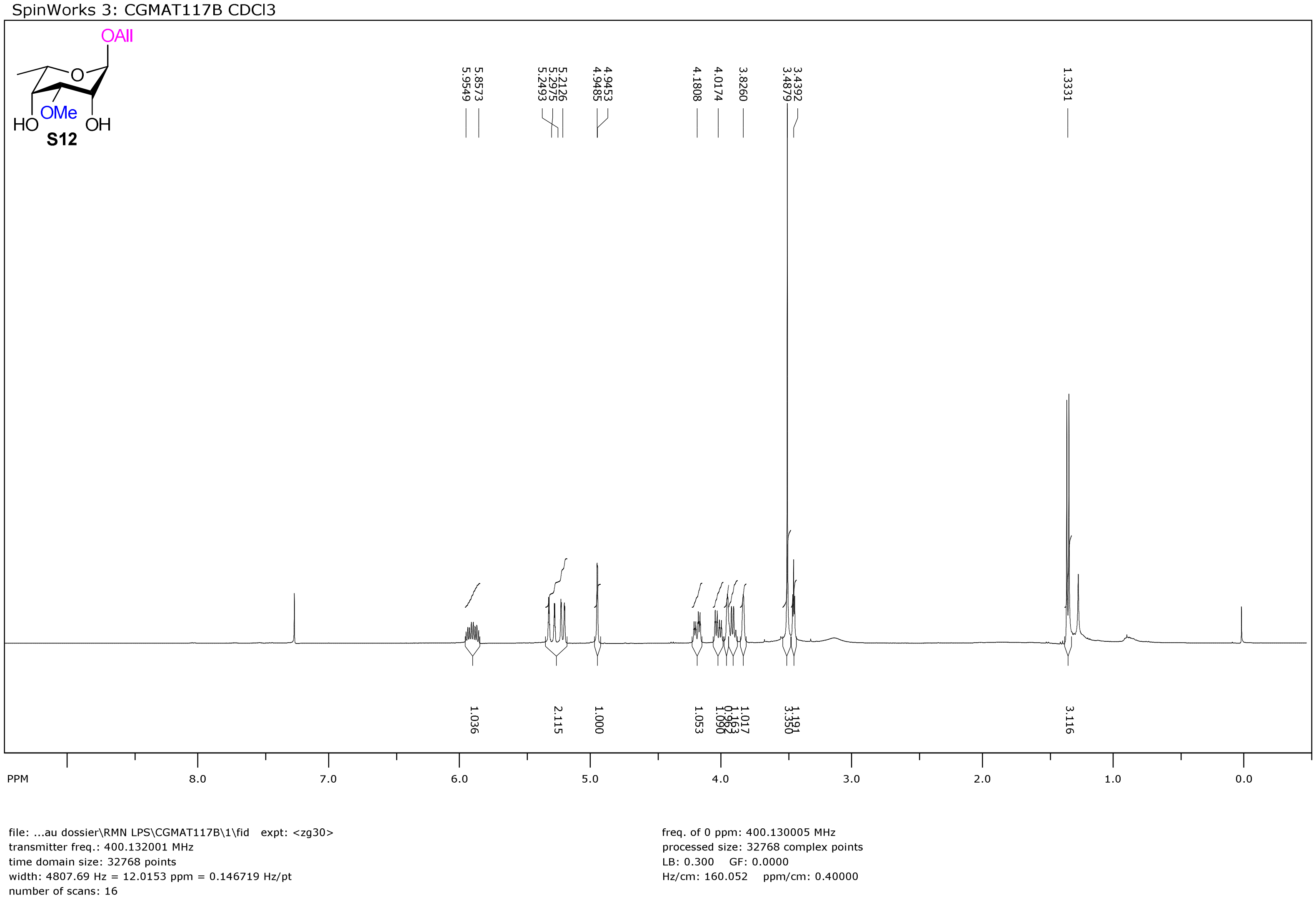

Supplementary Figure 32 | ^{13}C NMR spectra ($CDCl_3$, 100 MHz) of compound S12.

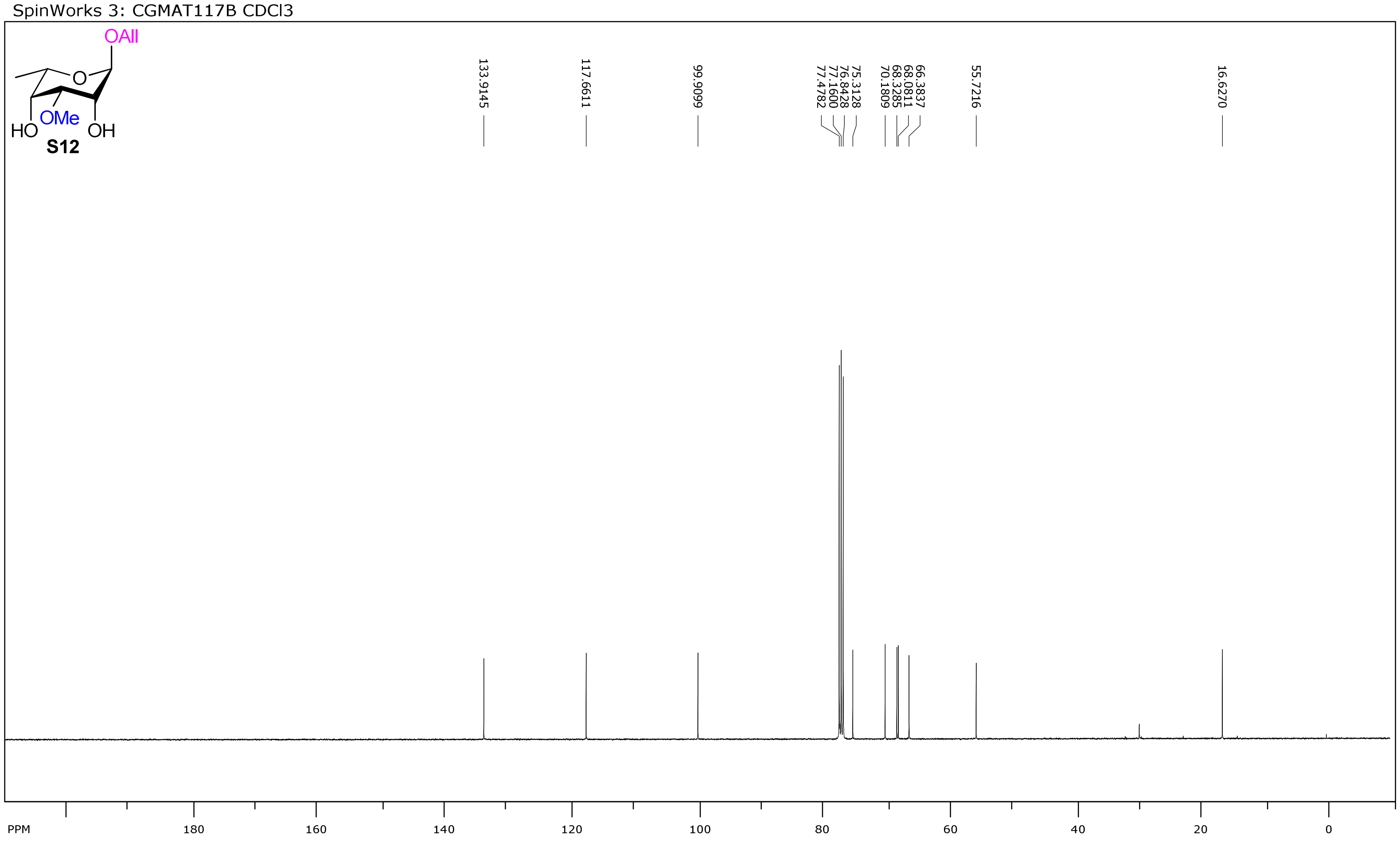

file: ...au dossier\RMN LPS\CGMAT117B\4\fid expt: <zgpg30>
transmitter freq.: 100.622830 MHz
time domain size: 65536 points
width: 24038.46 Hz = 238.8967 ppm = 0.366798 Hz/pt
number of scans: 2048

freq. of 0 ppm: 100.612756 MHz
processed size: 32768 complex points
LB: 1.000 GF: 0.0000
Hz/cm: 885.392 ppm/cm: 8.79912

Supplementary Figure 33 | ^{1}H NMR spectra ($CDCl_3$, 400 MHz) of compound S13.

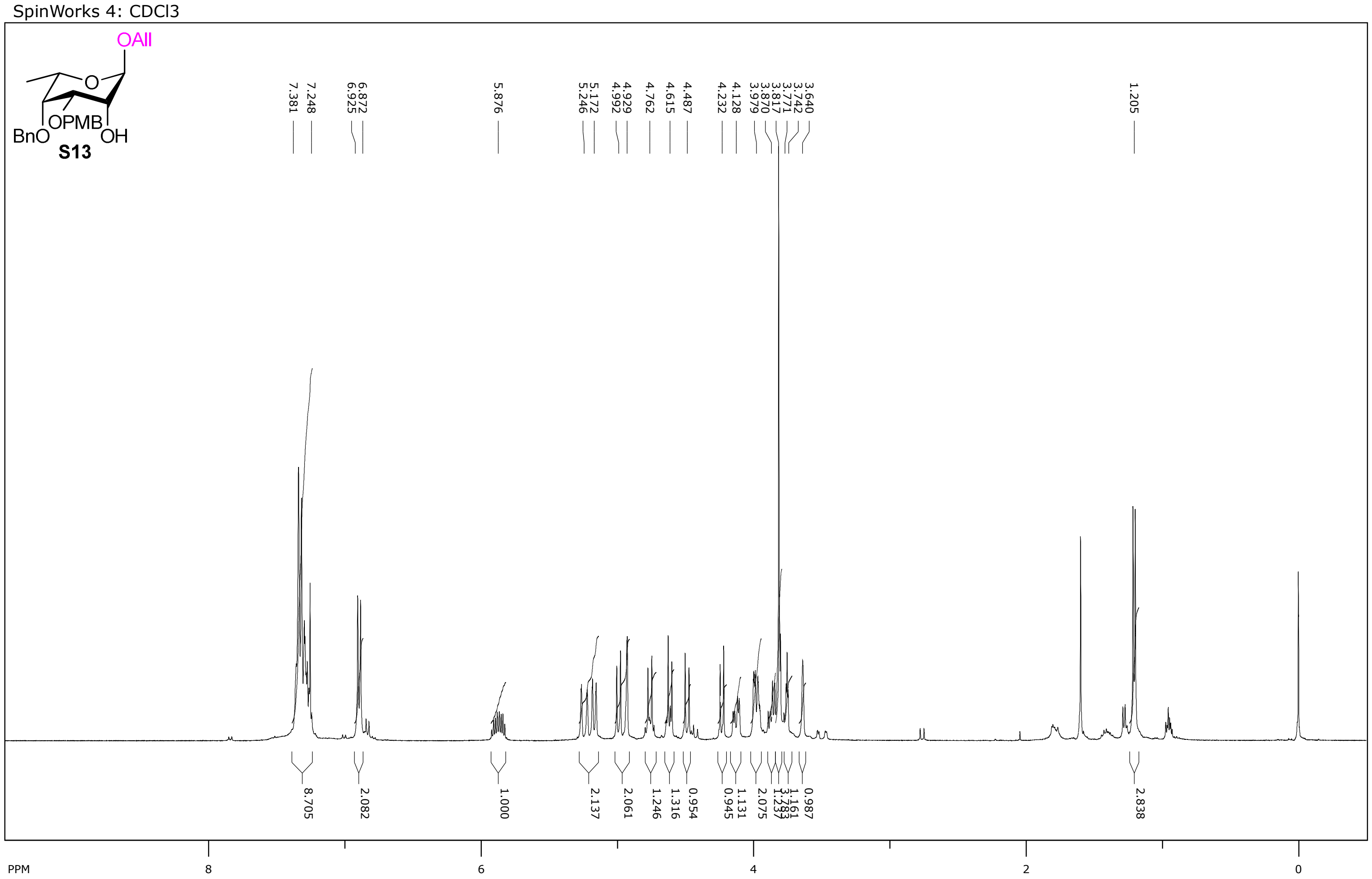

file: ...LPS Marielle\RMN LPS\CGAB29A\1\fid expt: <zg30>
transmitter freq.: 400.132001 MHz
time domain size: 32768 points
width: 4807.69 Hz = 12.0153 ppm = 0.146719 Hz/pt
number of scans: 16

freq. of 0 ppm: 400.130011 MHz
processed size: 32768 complex points
LB: 0.300 GF: 0.0000

Supplementary Figure 34 | ^{13}C NMR spectra ($CDCl_3$, 100 MHz) of compound S13.

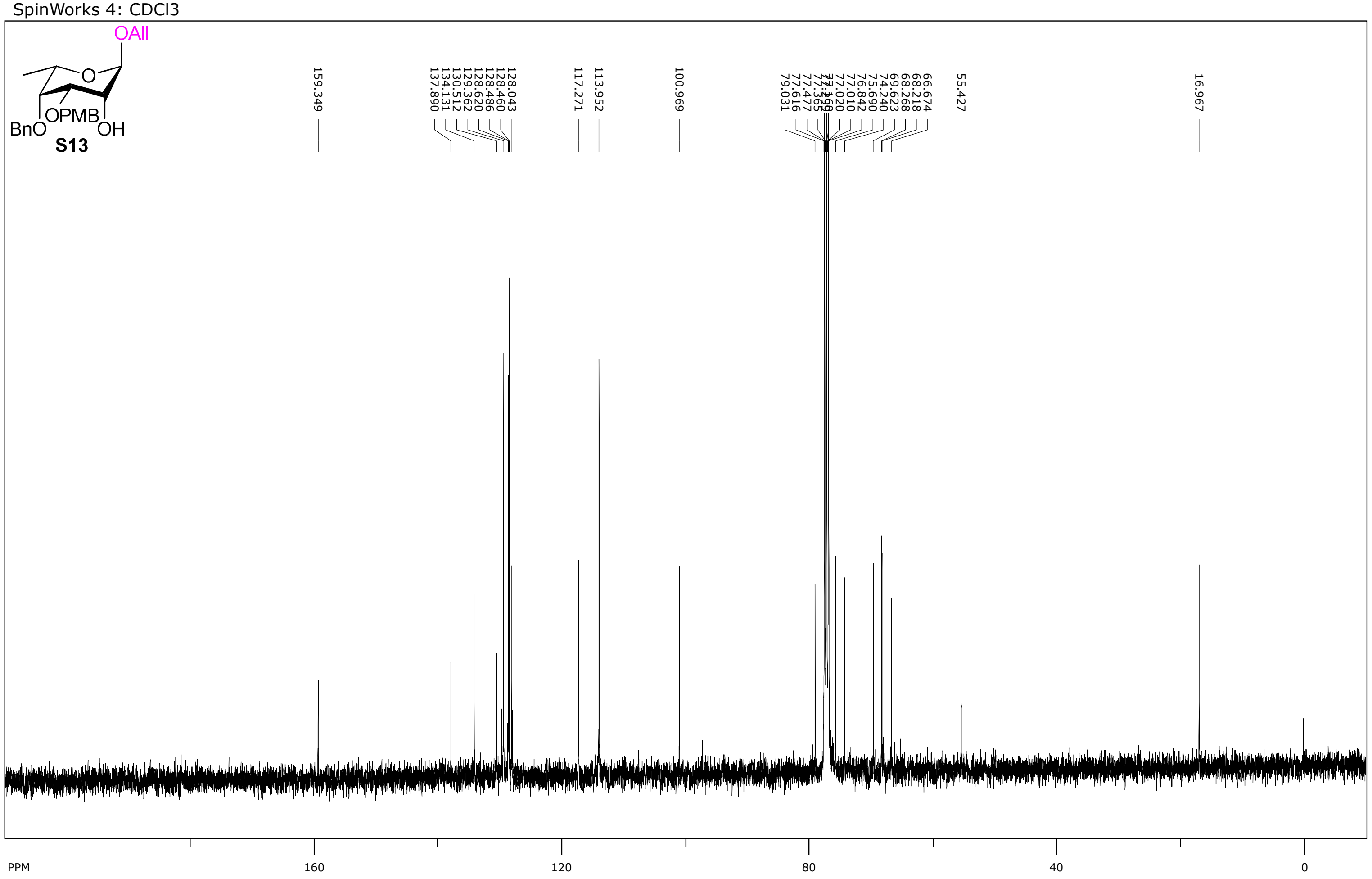

file: ...LPS Marielle\RMN LPS\CGAB29A\3\fid expt: <zgpg30>
transmitter freq.: 100.622830 MHz
time domain size: 65536 points
width: 24038.46 Hz = 238.8967 ppm = 0.366798 Hz/pt
number of scans: 512

freq. of 0 ppm: 100.612755 MHz
processed size: 32768 complex points
LB: 1.000 GF: 0.0000

Supplementary Figure 35 | ^{1}H NMR spectra ($CDCl_3$, 400 MHz) of compound S14.

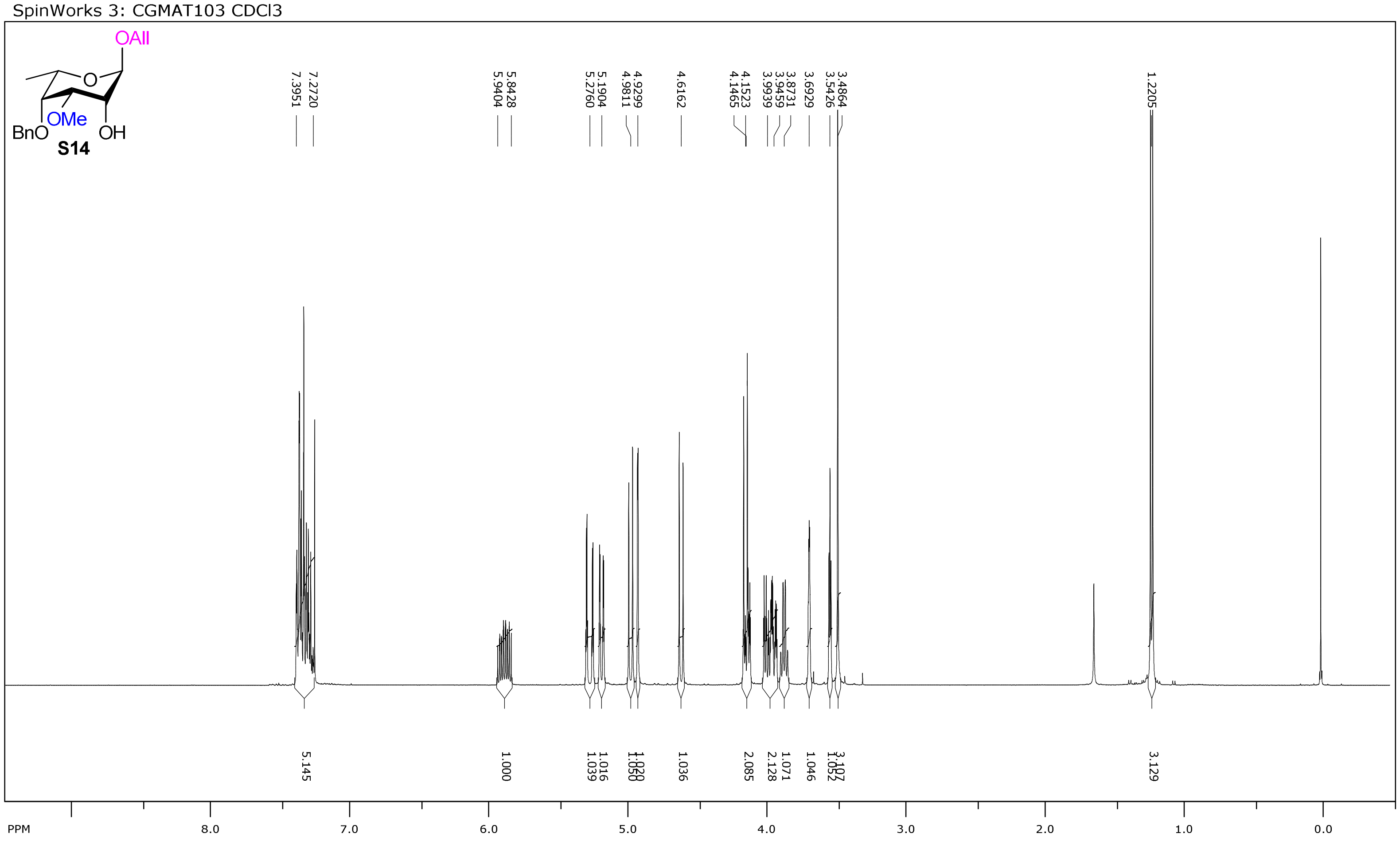

file: ...eau dossier\RMN LPS\CGMAT103\1\fid expt: <zg30>
transmitter freq.: 400.132001 MHz
time domain size: 32768 points
width: 4807.69 Hz = 12.0153 ppm = 0.146719 Hz/pt
number of scans: 16

freq. of 0 ppm: 400.130008 MHz
processed size: 32768 complex points
LB: 0.300 GF: 0.0000
Hz/cm: 160.052 ppm/cm: 0.40000

Supplementary Figure 36 | ^{13}C NMR spectra ($CDCl_3$, 100 MHz) of compound S14.

SpinWorks 3: CGMAT103 CDCl3

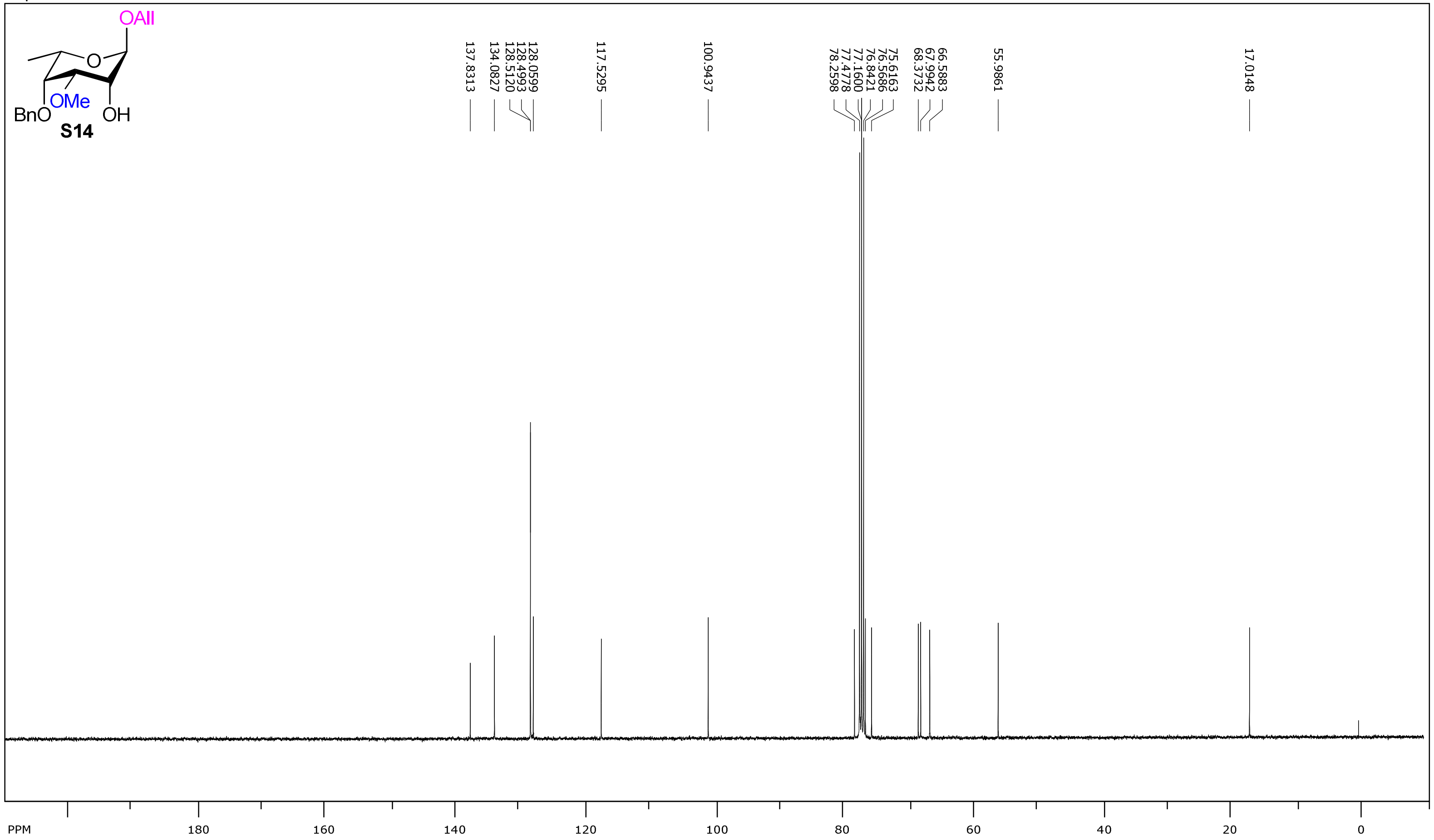

file: ...eau dossier\RMN LPS\CGMAT103\4\fid expt: <zgpg30>
transmitter freq.: 100.622830 MHz
time domain size: 65536 points
width: 24038.46 Hz = 238.8967 ppm = 0.366798 Hz/pt
number of scans: 1024

freq. of 0 ppm: 100.612756 MHz
processed size: 32768 complex points
LB: 1.000 GF: 0.0000
Hz/cm: 885.392 ppm/cm: 8.79912

Supplementary Figure 37 | ^{1}H NMR spectra ($CDCl_3$, 400 MHz) of compound S15.

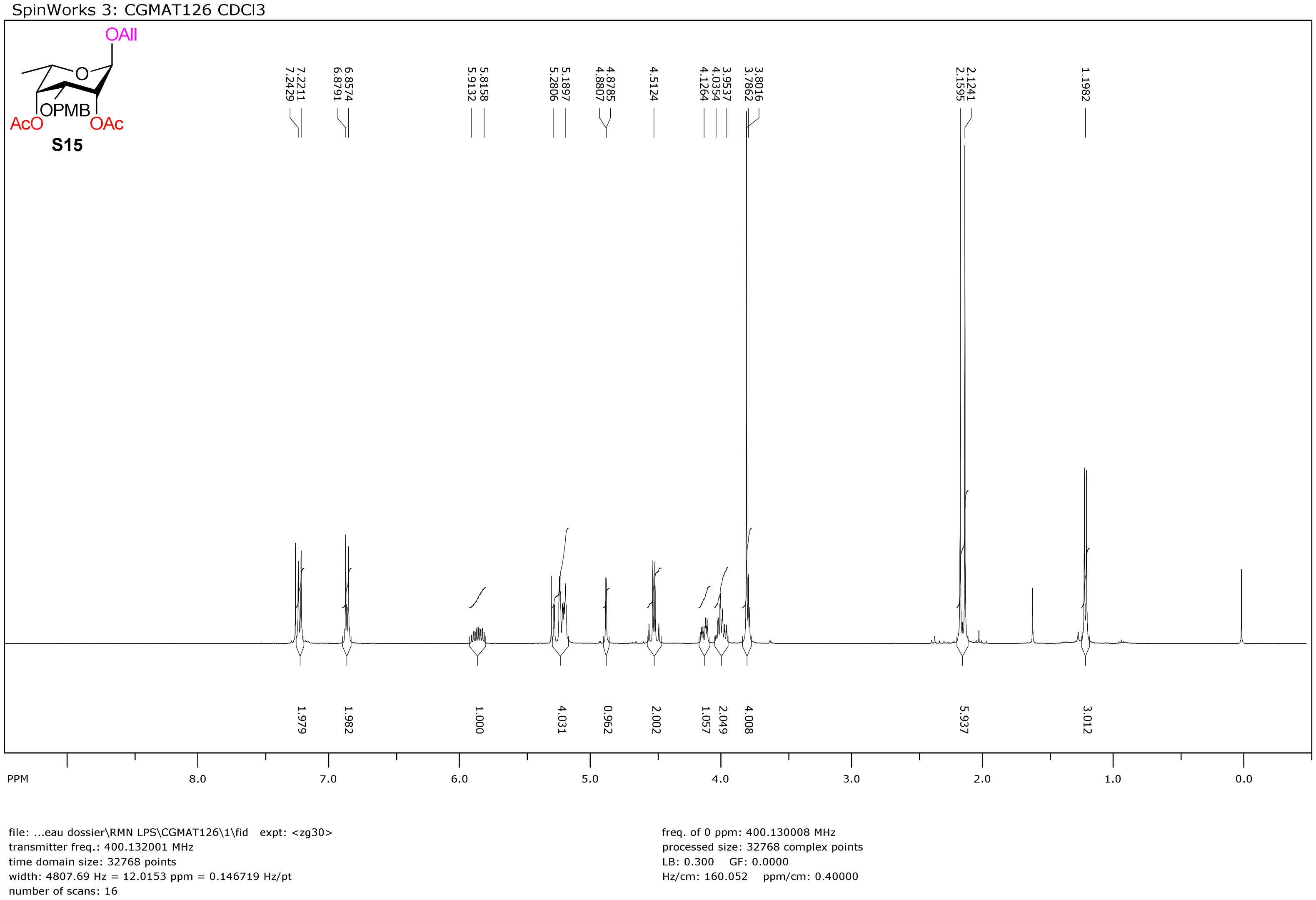

Supplementary Figure 38 | ^{13}C NMR spectra ($CDCl_3$, 100 MHz) of compound S15.

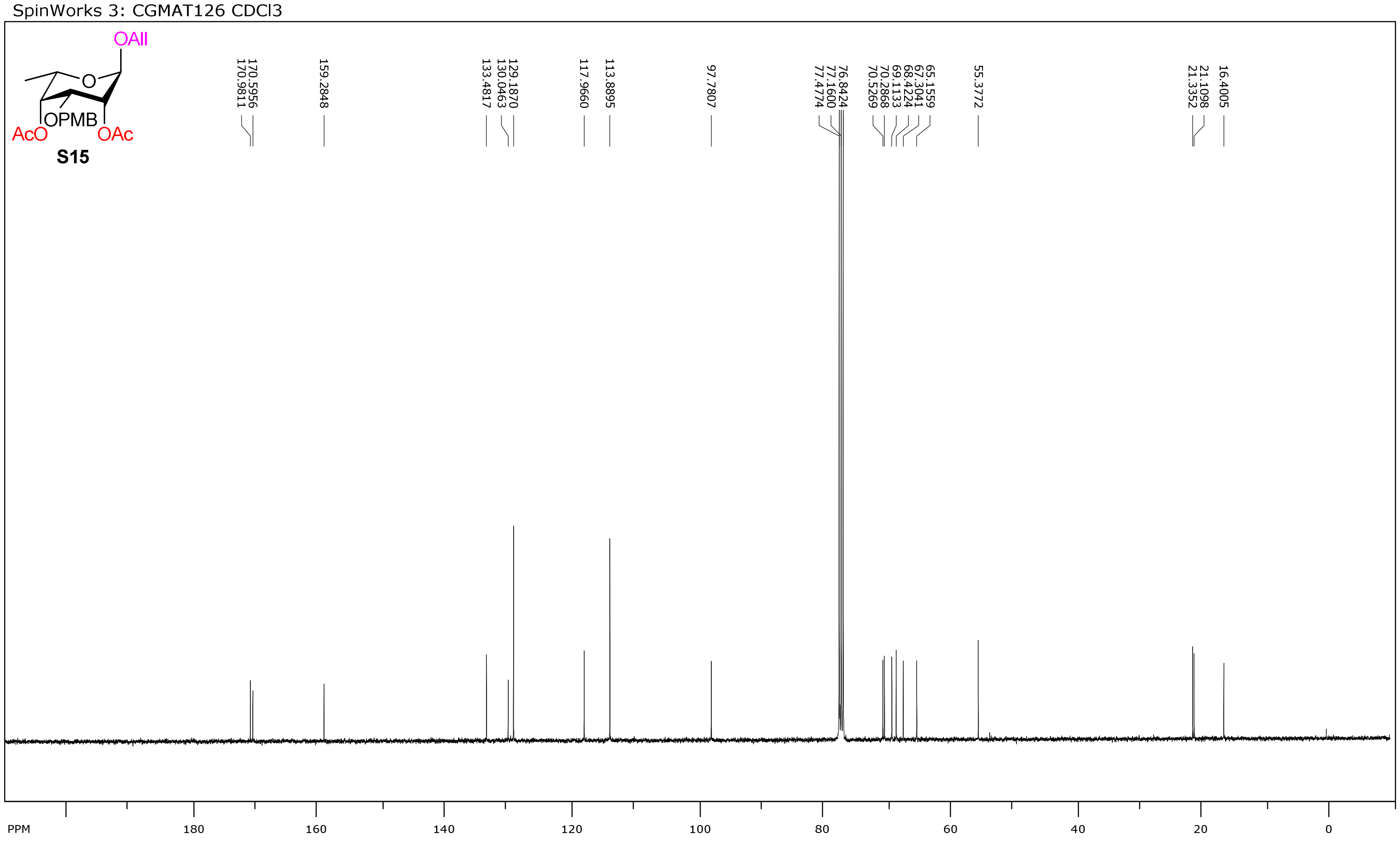

file: ...eau dossier\RMN LPS\CGMAT126\4\fid expt: <zgpg30>
transmitter freq.: 100.622830 MHz
time domain size: 65536 points
width: 24038.46 Hz = 238.8967 ppm = 0.366798 Hz/pt
number of scans: 1024

freq. of 0 ppm: 100.612756 MHz
processed size: 32768 complex points
LB: 1.000 GF: 0.0000
Hz/cm: 885.392 ppm/cm: 8.79912

Supplementary Figure 39 | ^{1}H NMR spectra ($CDCl_3$, 400 MHz) of compound S16.

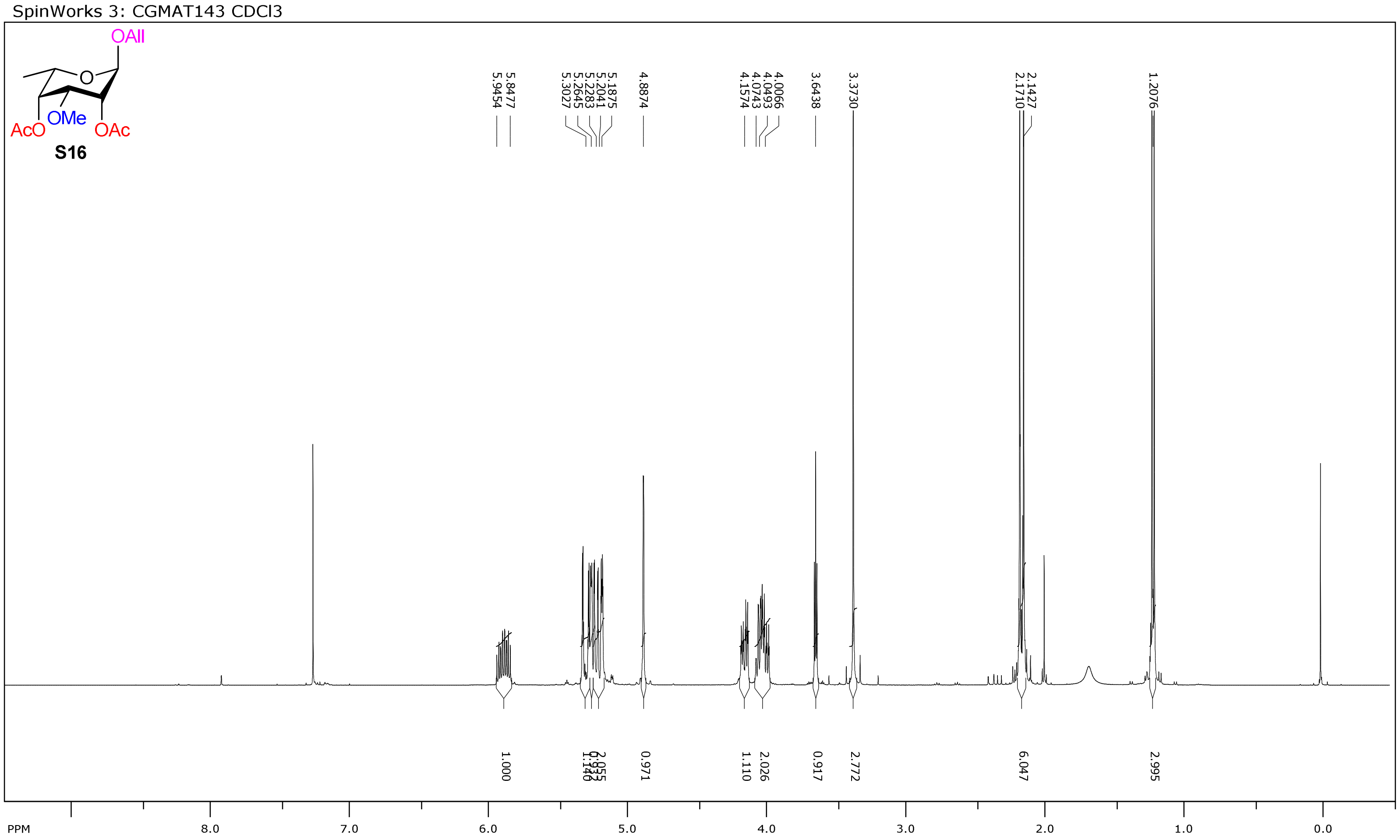

file: ...eau dossier\RMN LPS\CGMAT143\1\fid expt: <zg30>
transmitter freq.: 400.132001 MHz
time domain size: 32768 points
width: 4807.69 Hz = 12.0153 ppm = 0.146719 Hz/pt
number of scans: 32

freq. of 0 ppm: 400.130005 MHz
processed size: 32768 complex points
LB: 0.300 GF: 0.0000
Hz/cm: 160.052 ppm/cm: 0.40000

Supplementary Figure 40 | ^{13}C NMR spectra ($CDCl_3$, 100 MHz) of compound S16.

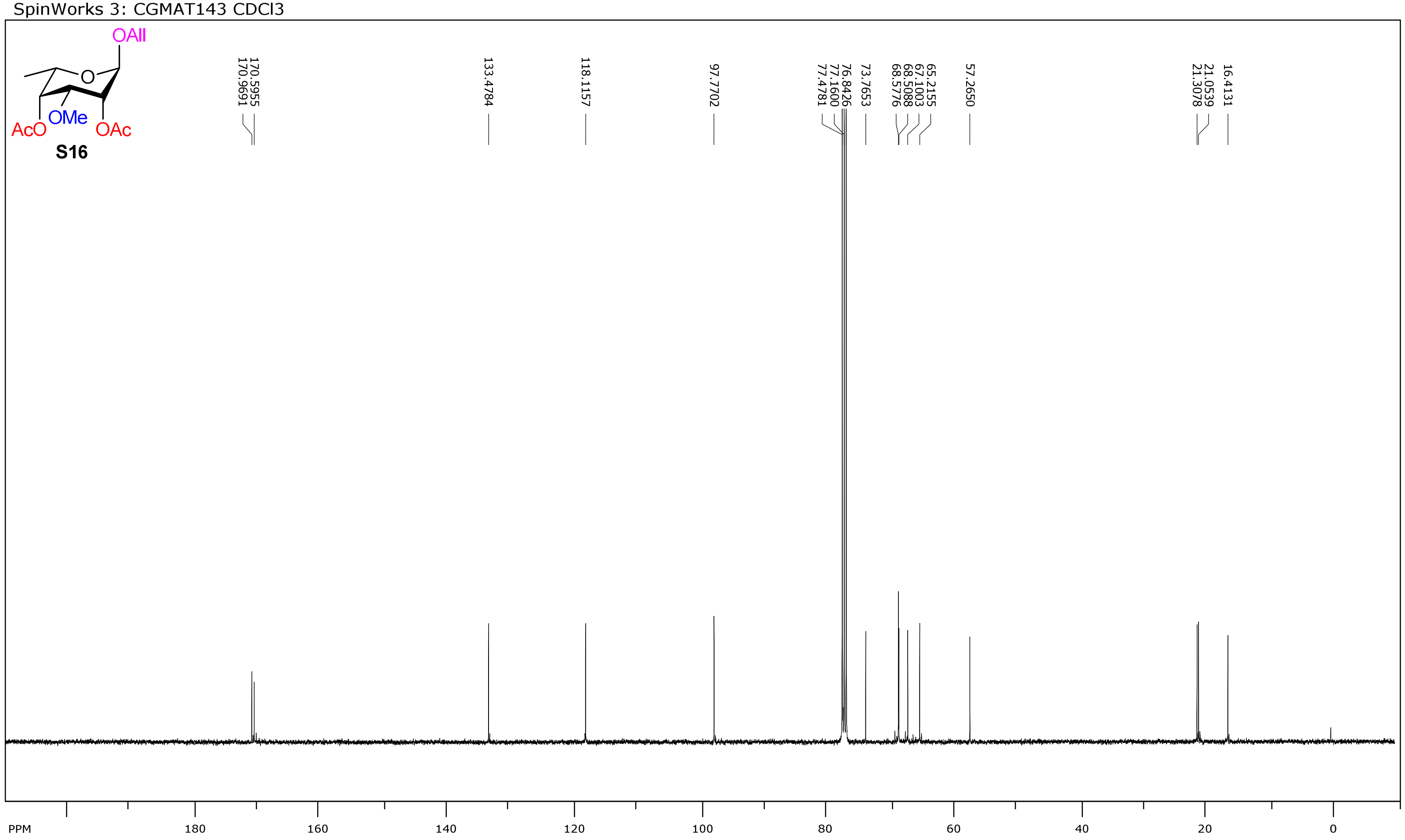

file: ...eau dossier\RMN LPS\CGMAT143\3\fid expt: <zgpg30>
transmitter freq.: 100.622830 MHz
time domain size: 65536 points
width: 24038.46 Hz = 238.8967 ppm = 0.366798 Hz/pt
number of scans: 1024

freq. of 0 ppm: 100.612755 MHz
processed size: 32768 complex points
LB: 1.000 GF: 0.0000
Hz/cm: 885.392 ppm/cm: 8.79912

Supplementary Figure 41 | ^{1}H NMR spectra ($CDCl_3$, 400 MHz) of compound S6.

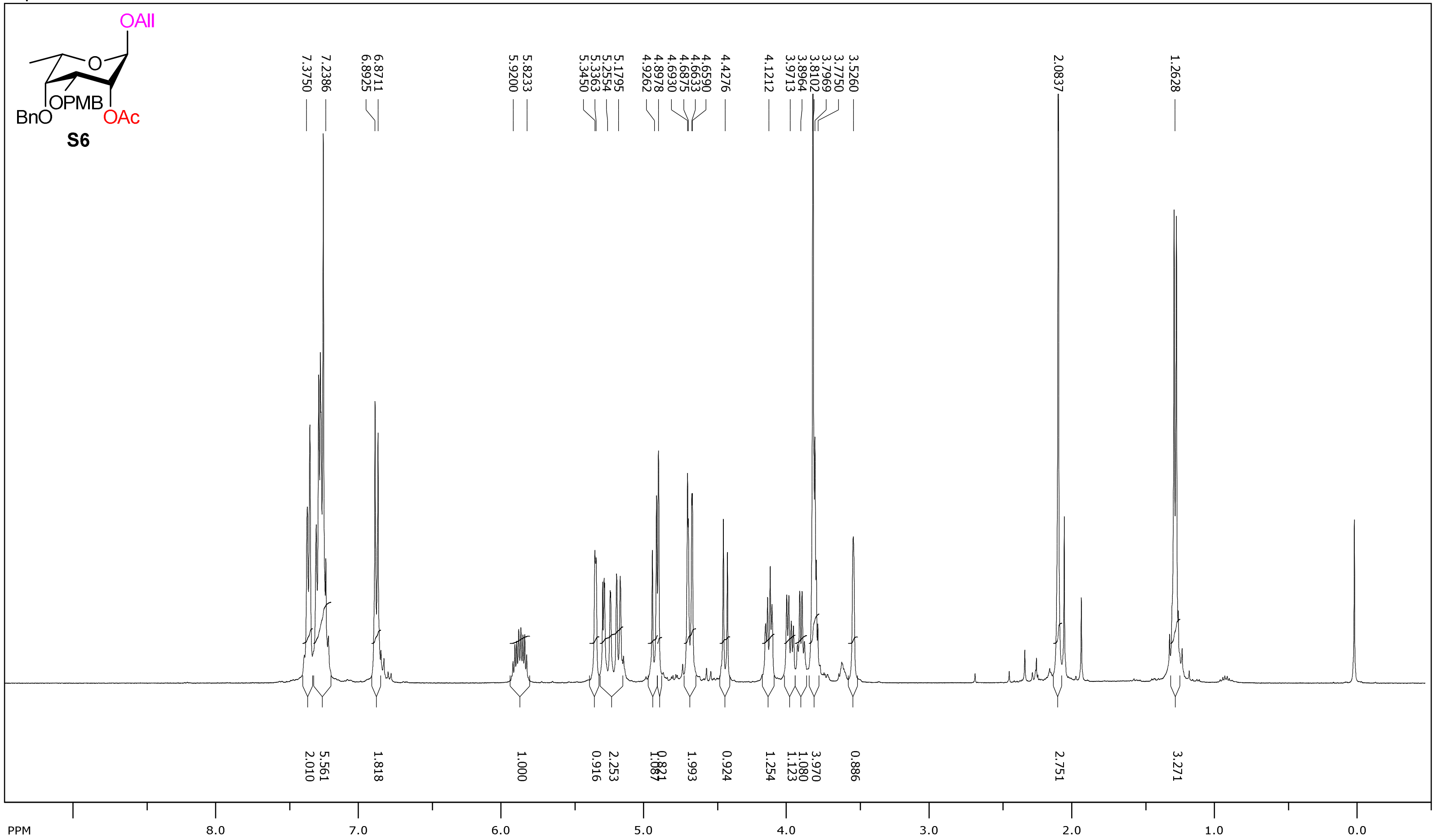

file: ...veau dossier\RMN LPS\CGMAT32\1\fid expt: <zg30>
transmitter freq.: 400.132001 MHz
time domain size: 32768 points
width: 4807.69 Hz = 12.0153 ppm = 0.146719 Hz/pt
number of scans: 16

freq. of 0 ppm: 400.130011 MHz
processed size: 32768 complex points
LB: 0.300 GF: 0.0000
Hz/cm: 160.052 ppm/cm: 0.40000

Supplementary Figure 42 | ^{13}C NMR spectra ($CDCl_3$, 100 MHz) of compound S6.

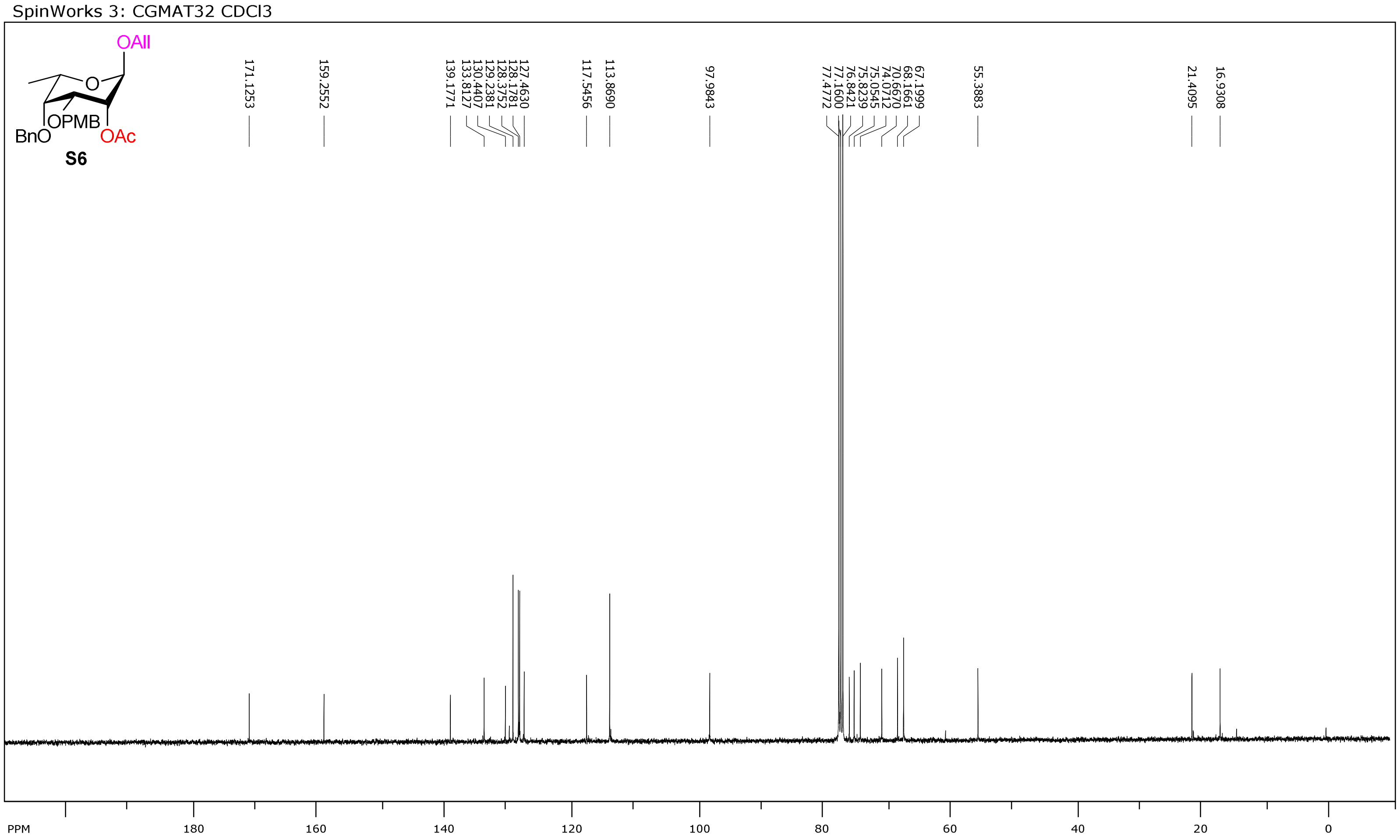

file: ...veau dossier\RMN LPS\CGMAT32\4\fid expt: <zgpg30>
transmitter freq.: 100.622830 MHz
time domain size: 65536 points
width: 24038.46 Hz = 238.8967 ppm = 0.366798 Hz/pt
number of scans: 512

freq. of 0 ppm: 100.612758 MHz
processed size: 32768 complex points
LB: 1.000 GF: 0.0000
Hz/cm: 885.392 ppm/cm: 8.79912

Supplementary Figure 43 | ^{1}H NMR spectra ($CDCl_3$, 400 MHz) of compound S17.

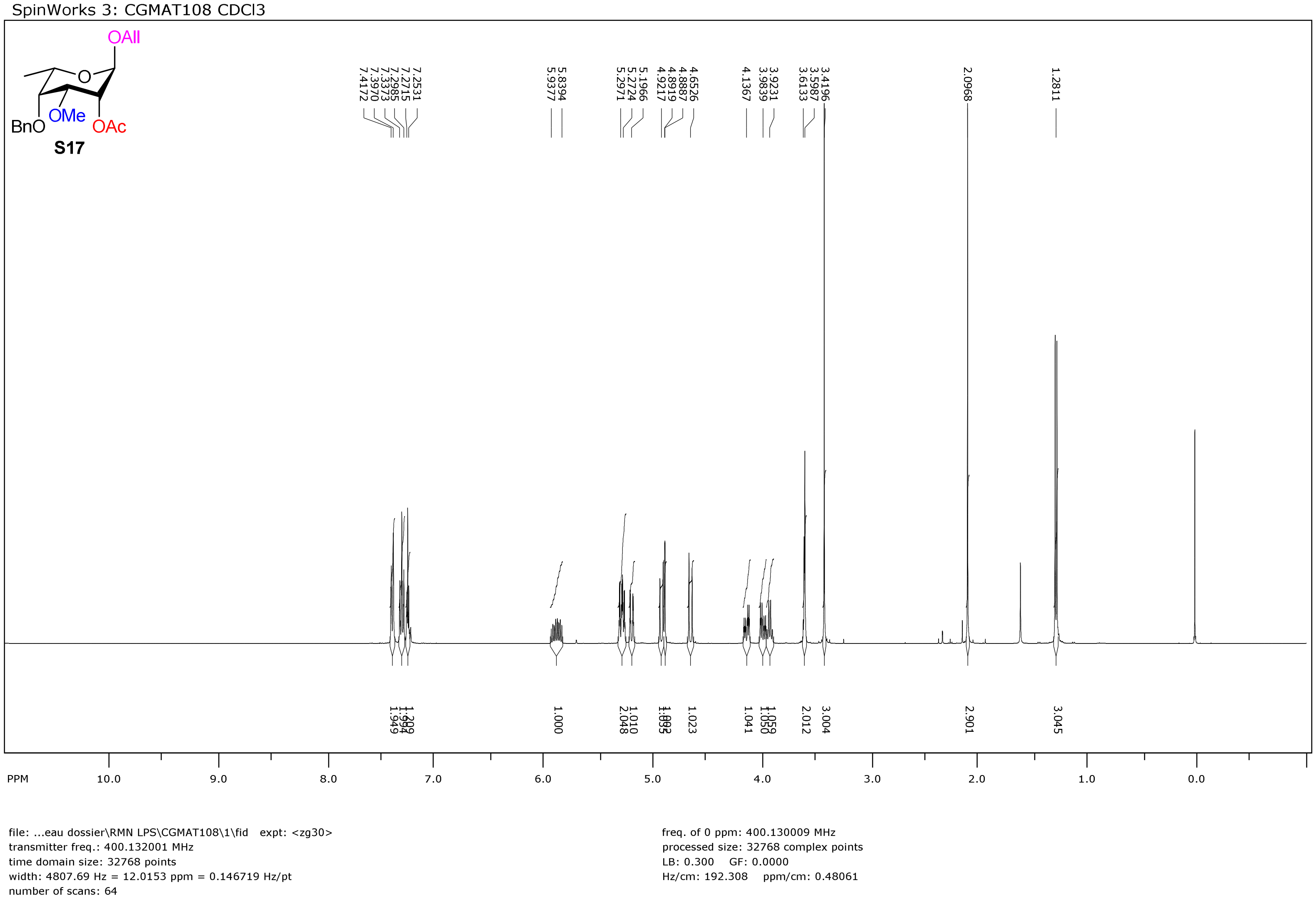

Supplementary Figure 44 | ^{13}C NMR spectra ($CDCl_3$, 100 MHz) of compound S17.

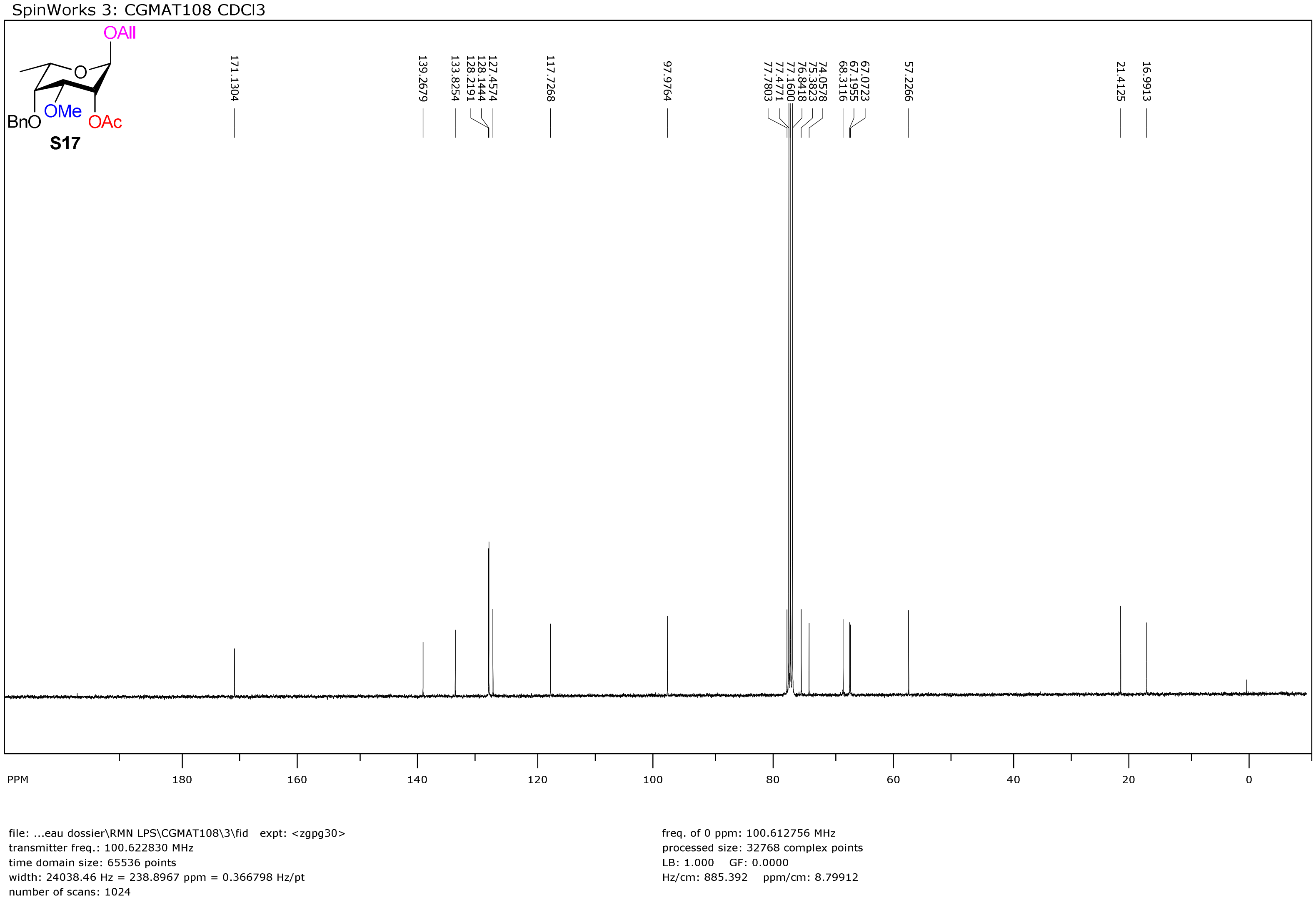

Supplementary Figure 45 | ^{1}H NMR spectra (py-d_5, 400 MHz) of compound 8.

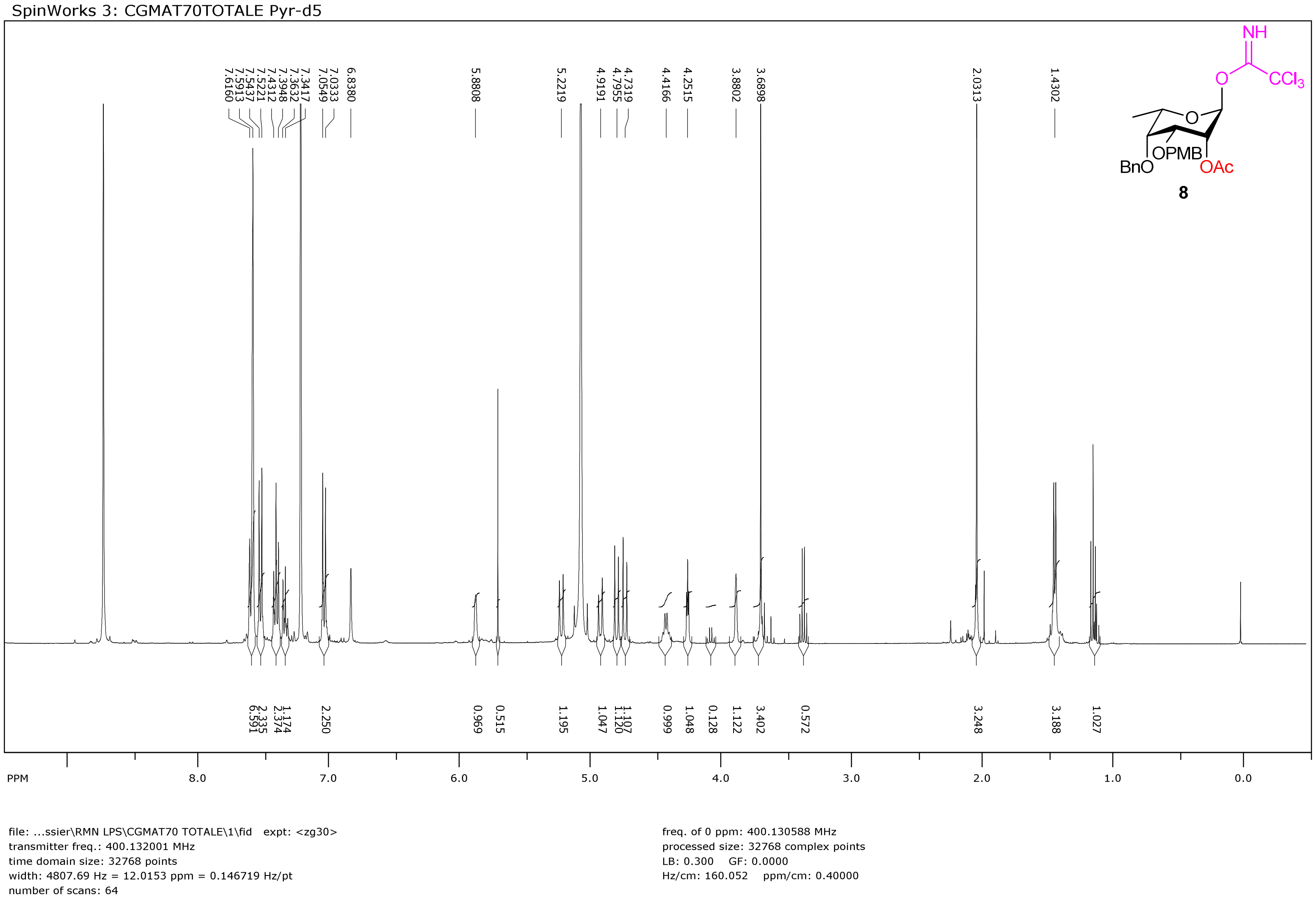

Supplementary Figure 46 | ^{13}C NMR spectra (py-d_5, 100 MHz) of compound 8.

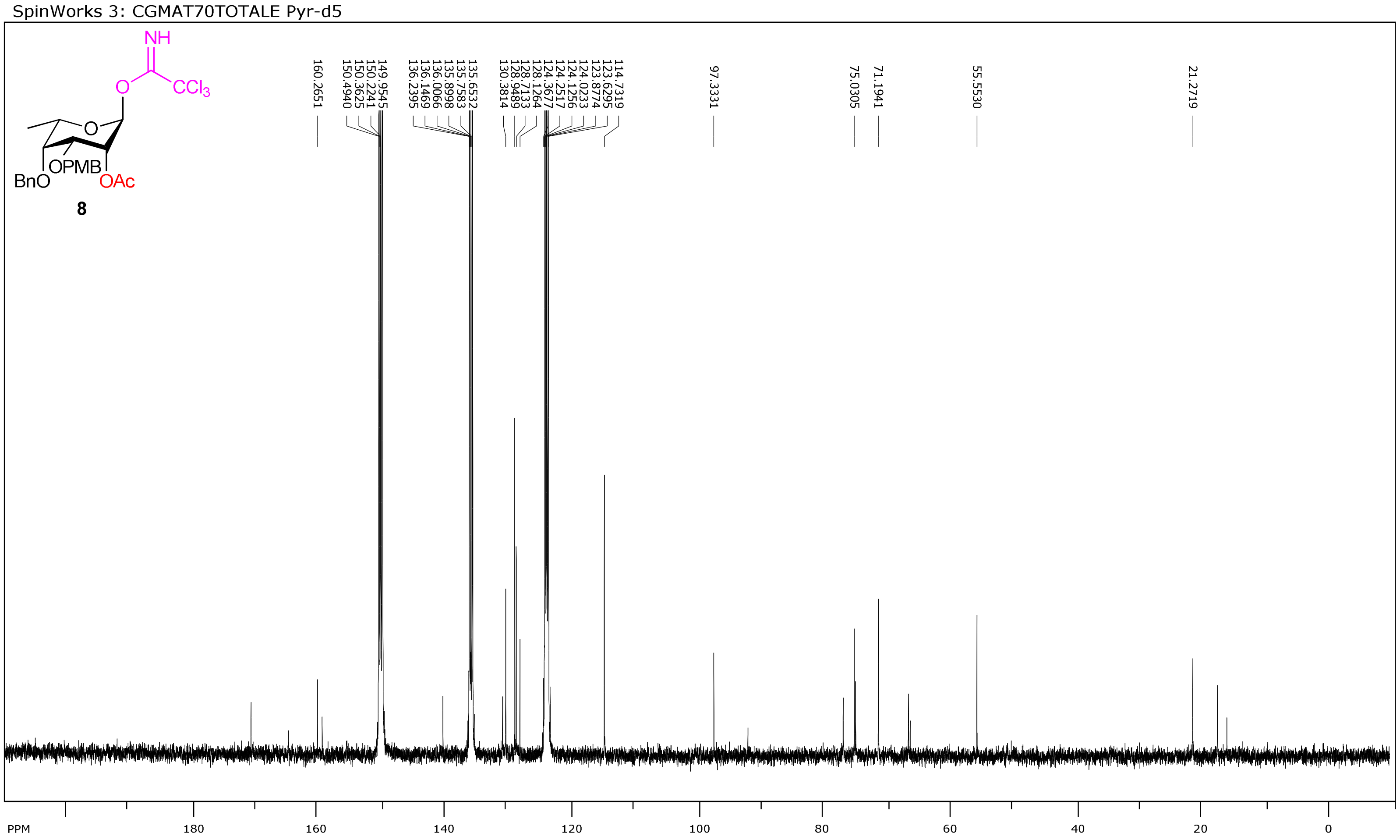

file: ...ssier\RMN LPS\CGMAT70 TOTALE\3\fid expt: <zgpg30>
transmitter freq.: 100.622830 MHz
time domain size: 65536 points
width: 24038.46 Hz = 238.8967 ppm = 0.366798 Hz/pt
number of scans: 1024

freq. of 0 ppm: 100.612860 MHz
processed size: 32768 complex points
LB: 1.000 GF: 0.0000
Hz/cm: 885.393 ppm/cm: 8.79913

Supplementary Figure 47 | ^{1}H NMR spectra (py-d_5, 400 MHz) of compound 9.

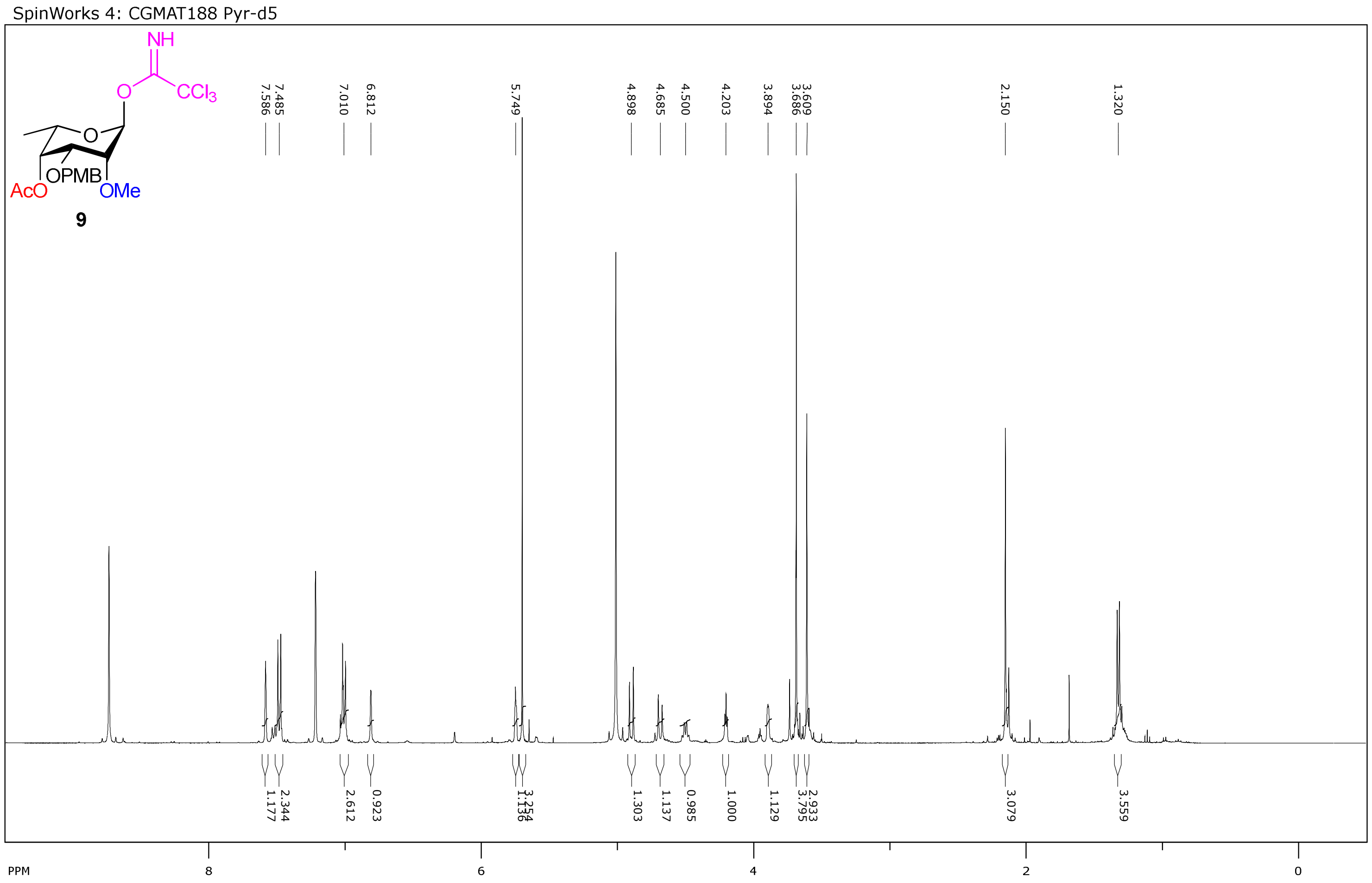

file: ...eau dossier\RMN LPS\CGMAT188\1\fid expt: <zg30>
transmitter freq.: 400.132001 MHz
time domain size: 32768 points
width: 4807.69 Hz = 12.0153 ppm = 0.146719 Hz/pt
number of scans: 32

freq. of 0 ppm: 400.130591 MHz
processed size: 32768 complex points
LB: 0.300 GF: 0.0000

Supplementary Figure 48 | ^{13}C NMR spectra (py-d_5, 100 MHz) of compound 9.

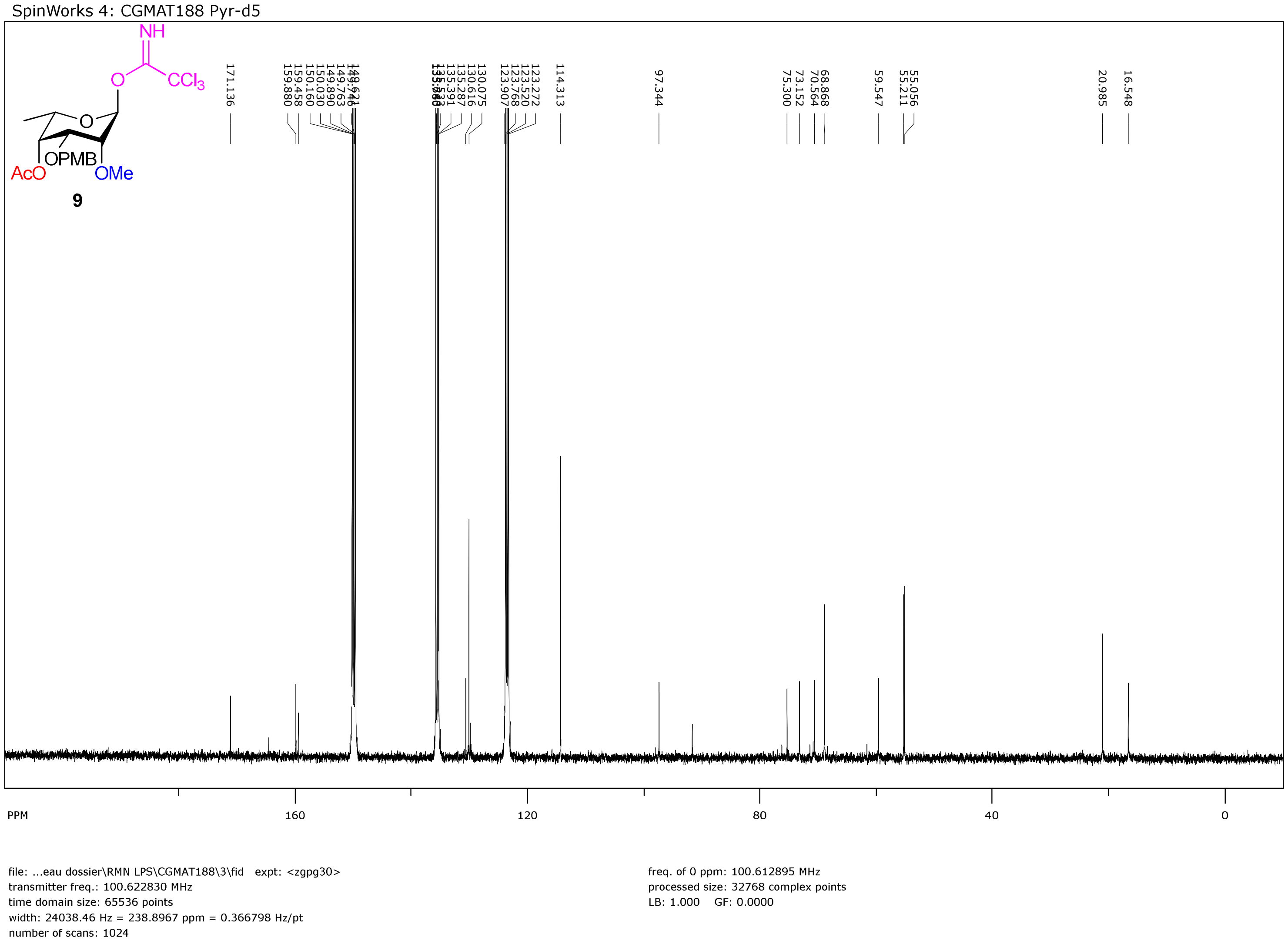

Supplementary Figure 49 | ^{1}H NMR spectra (py-d_5, 400 MHz) of compound 10.

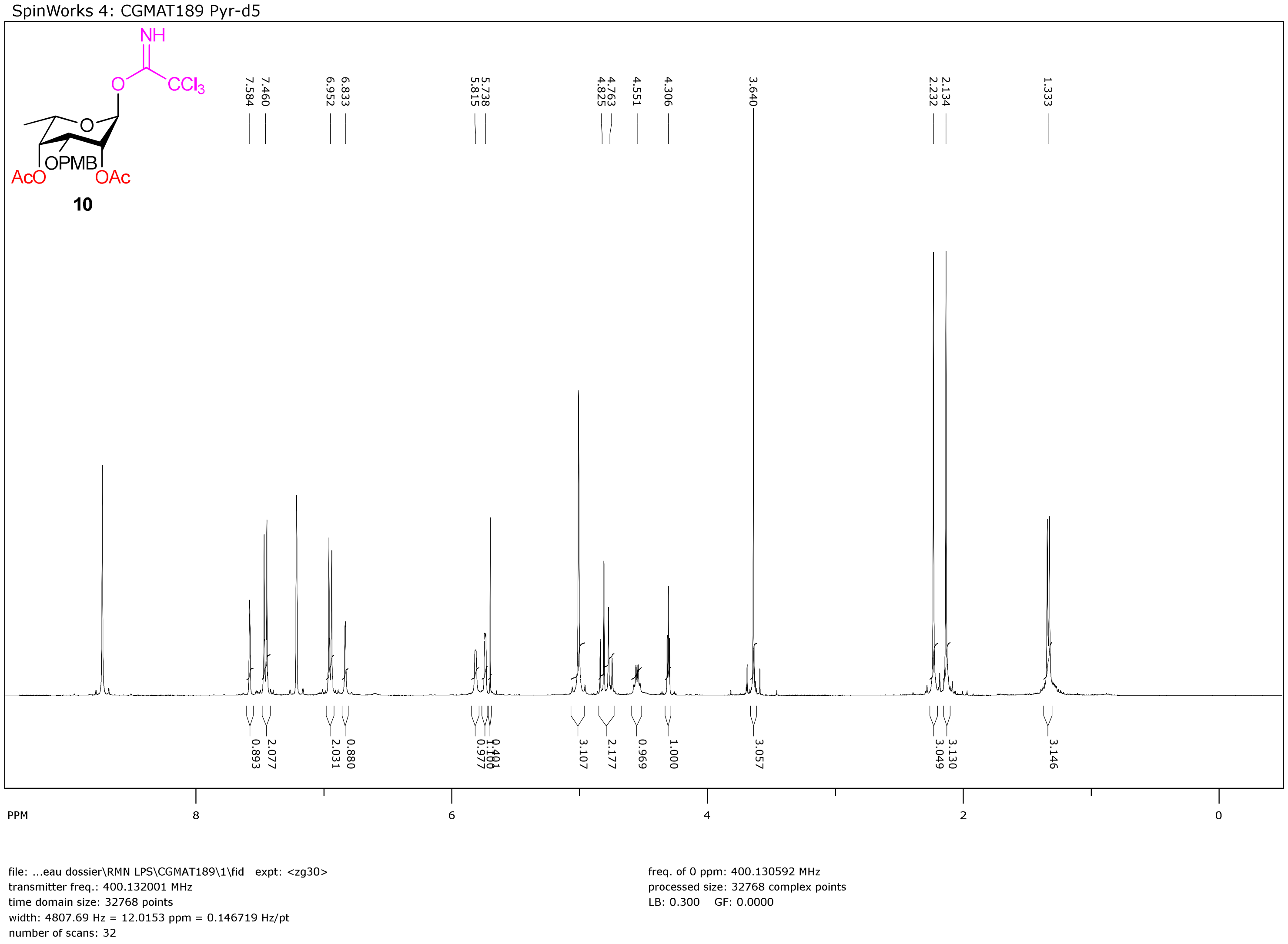

Supplementary Figure 50 | ^{13}C NMR spectra (py-d_5, 100 MHz) of compound 10.

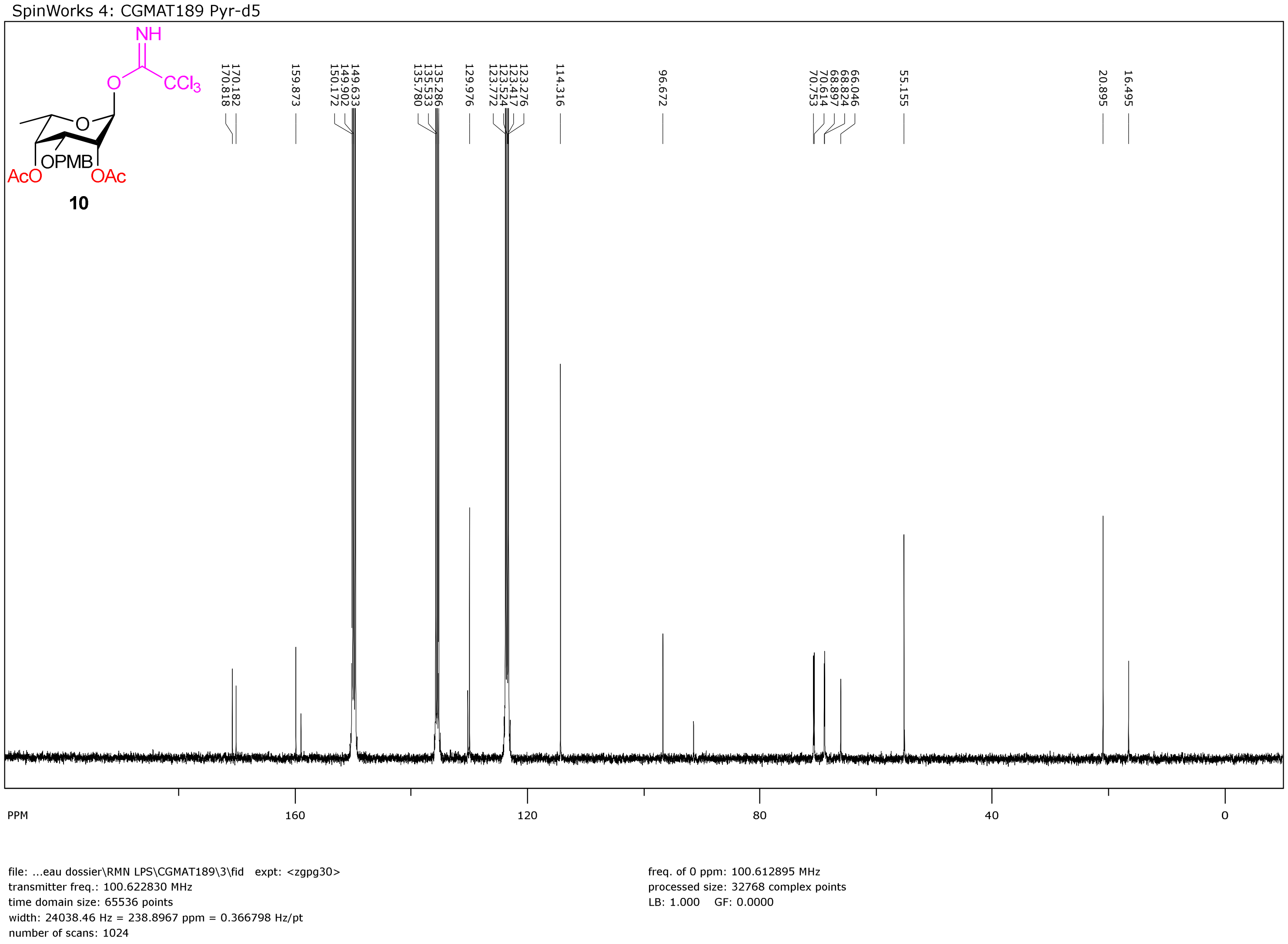

Supplementary Figure 51 | ^{1}H NMR spectra (py-d_5, 400 MHz) of compound 11.

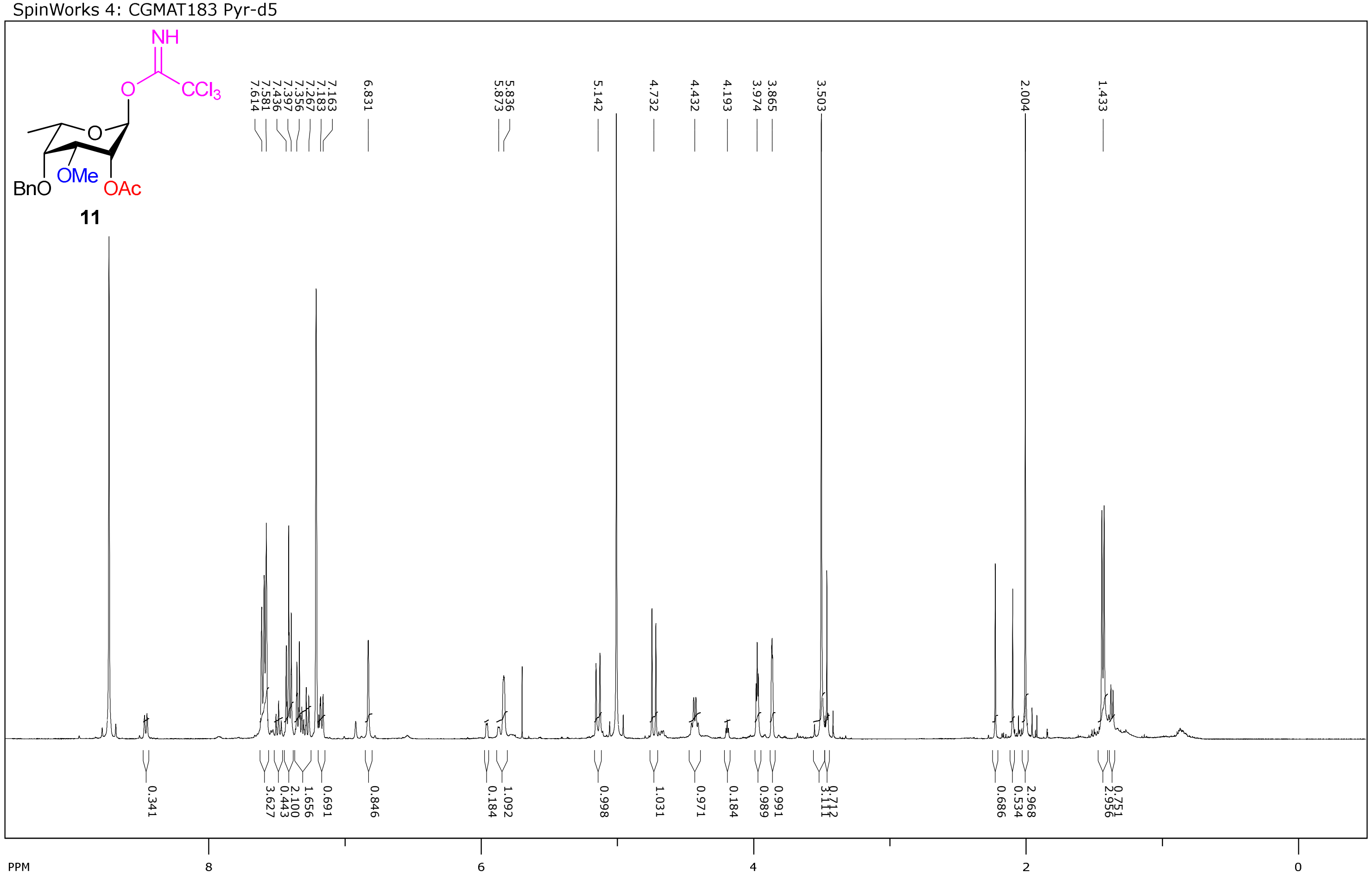

file: ...eau dossier\RMN LPS\CGMAT183\1\fid expt: <zg30>
transmitter freq.: 400.132001 MHz
time domain size: 32768 points
width: 4807.69 Hz = 12.0153 ppm = 0.146719 Hz/pt
number of scans: 32

freq. of 0 ppm: 400.130593 MHz
processed size: 32768 complex points
LB: 0.300 GF: 0.0000

Supplementary Figure 52 | ^{13}C NMR spectra (py-d_5, 100 MHz) of compound 11.

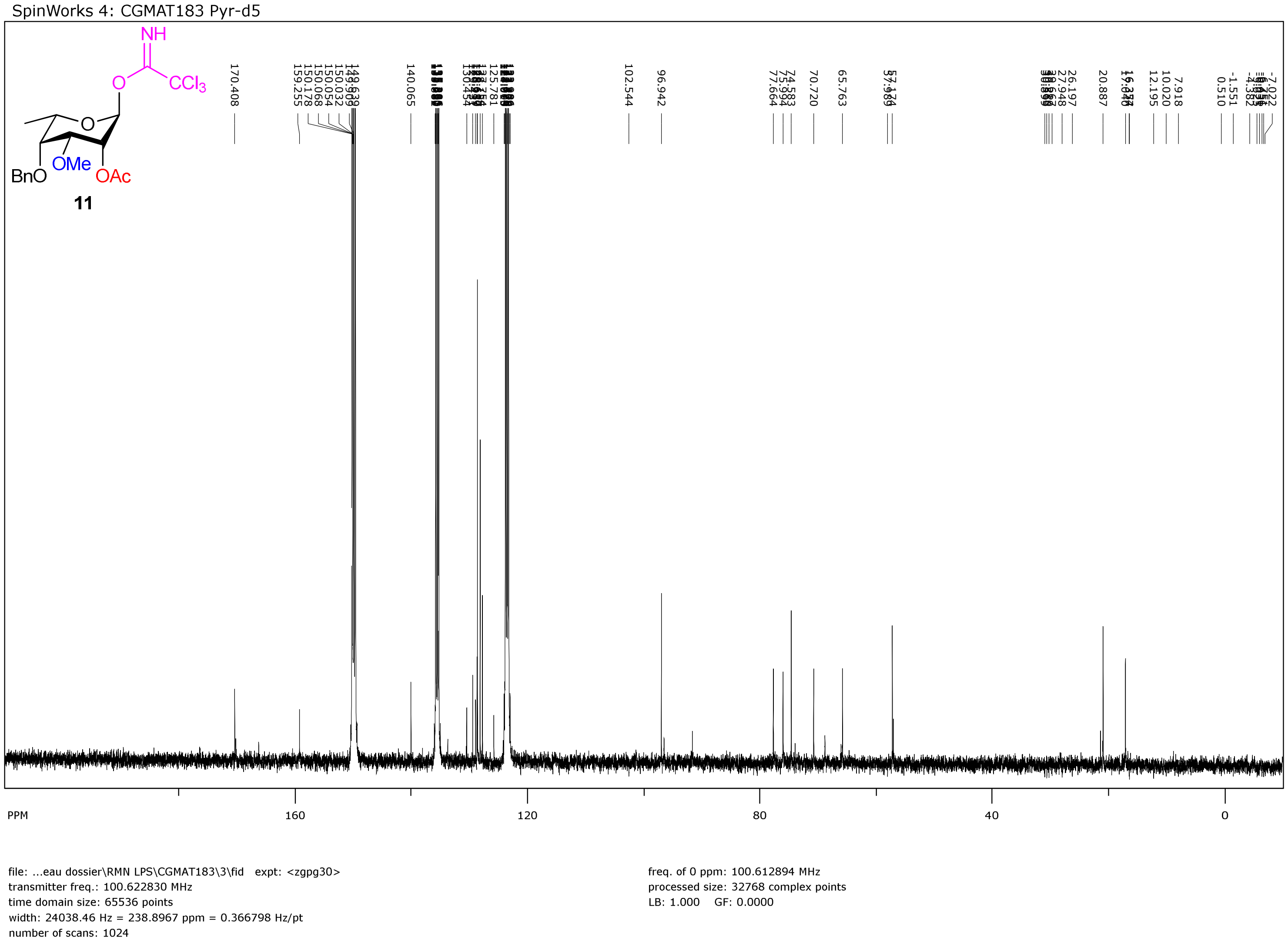

Supplementary Figure 53 │ ^{1}H NMR spectra (py-d_5, 400 MHz) of compound 12.

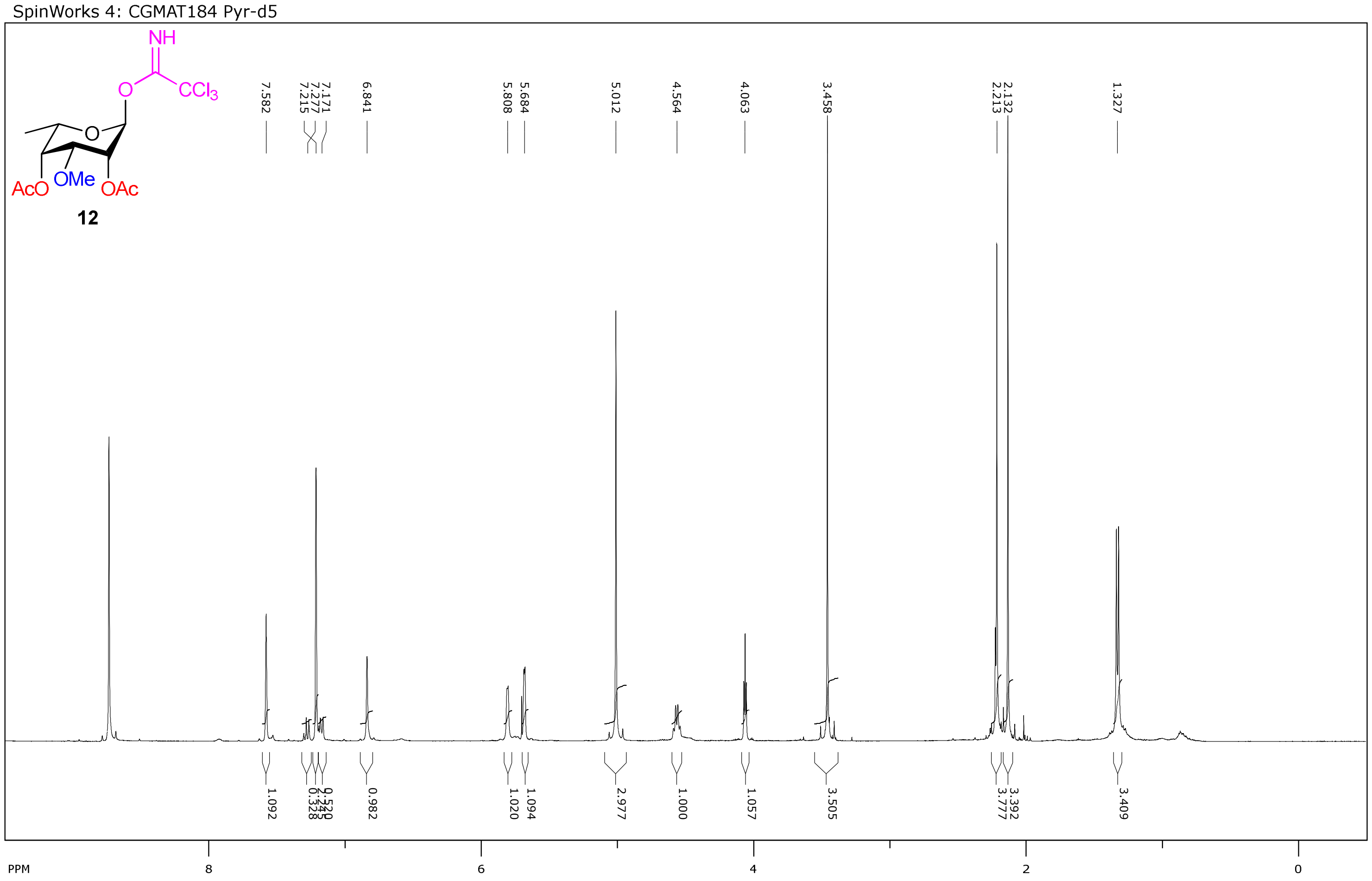

file: ...eau dossier\RMN LPS\CGMAT184\1\fid expt: <zg30>
transmitter freq.: 400.132001 MHz
time domain size: 32768 points
width: 4807.69 Hz = 12.0153 ppm = 0.146719 Hz/pt
number of scans: 32

freq. of 0 ppm: 400.130592 MHz
processed size: 32768 complex points
LB: 0.300 GF: 0.0000

Supplementary Figure 54 | ^{13}C NMR spectra (py-d_5, 100 MHz) of compound 12.

SpinWorks 4: CGMAT184 Pyr-d5

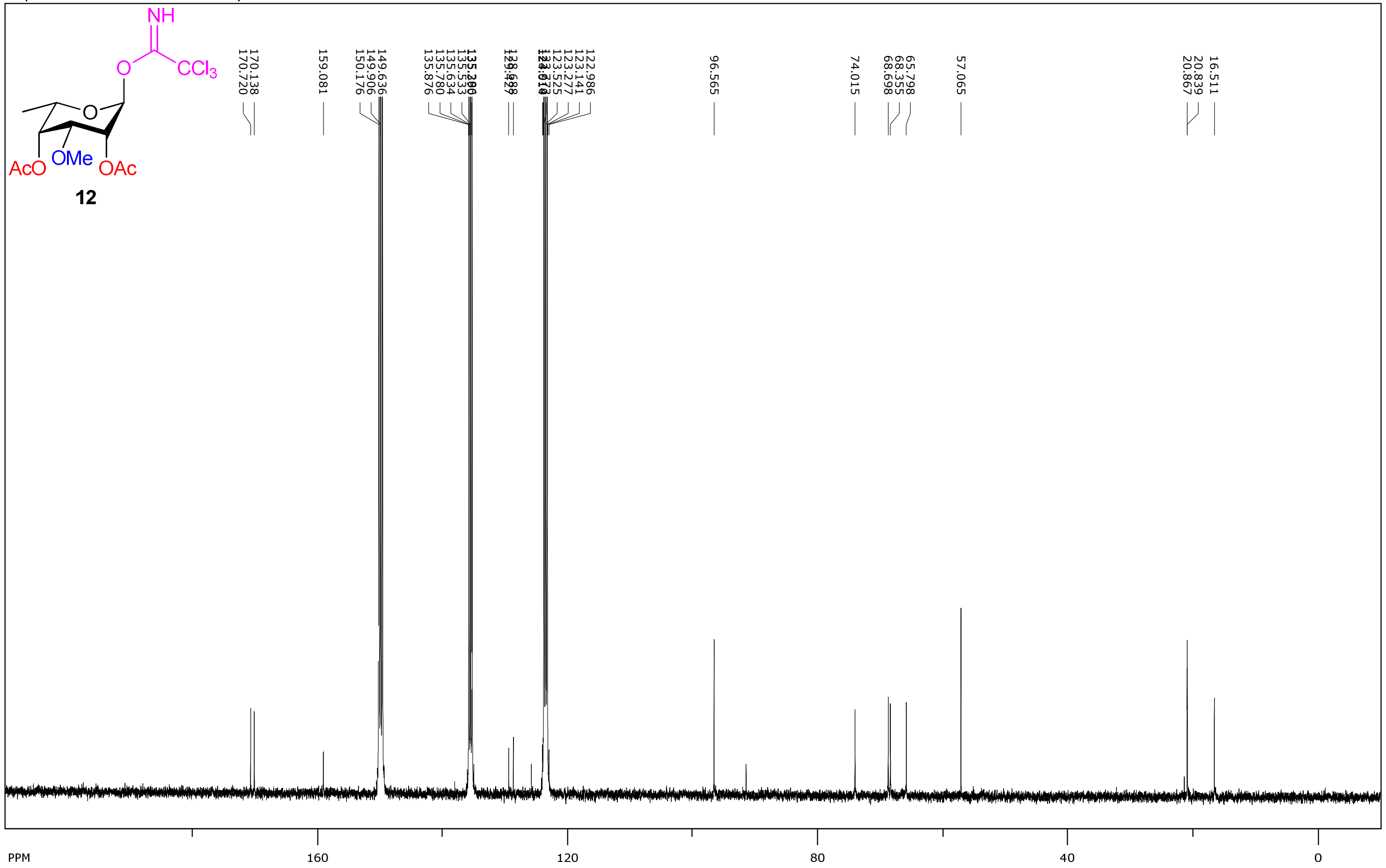

file: ...eau dossier\RMN LPS\CGMAT184\3\fid expt: <zgpg30>
transmitter freq.: 100.622830 MHz
time domain size: 65536 points
width: 24038.46 Hz = 238.8967 ppm = 0.366798 Hz/pt
number of scans: 1024

freq. of 0 ppm: 100.612895 MHz
processed size: 32768 complex points
LB: 1.000 GF: 0.0000

Supplementary Figure 55 │ ¹H NMR spectra ($CDCl_3$, 400 MHz) of compound S20.

SpinWorks 4: CGMAT34 CDCl3

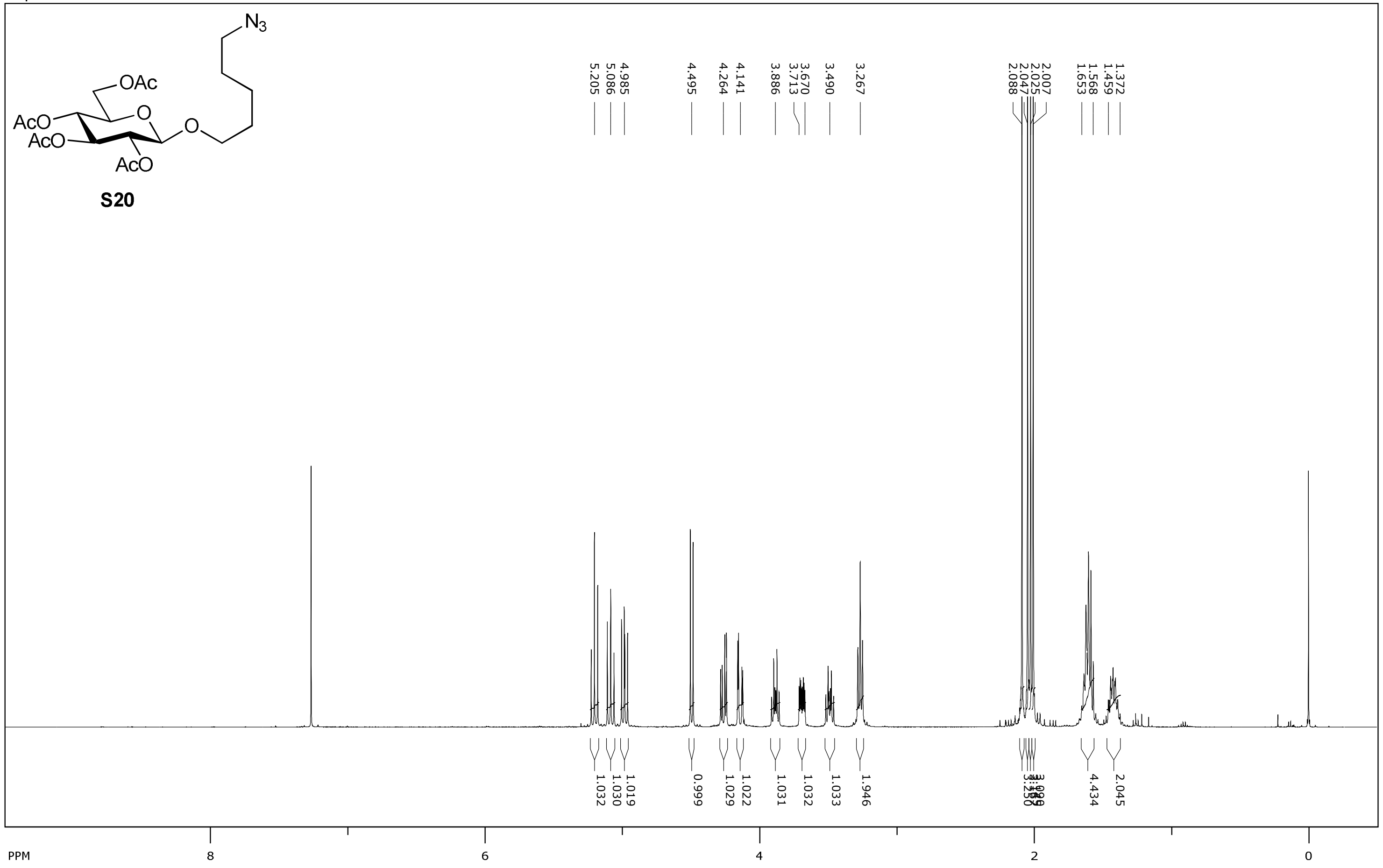

file: ...veau dossier\RMN LPS\CGMAT34\1\fid expt: <zg30>
transmitter freq.: 400.132001 MHz
time domain size: 32768 points
width: 4807.69 Hz = 12.0153 ppm = 0.146719 Hz/pt
number of scans: 16

freq. of 0 ppm: 400.130006 MHz
processed size: 32768 complex points
LB: 0.300 GF: 0.0000

Supplementary Figure 56 | ^{13}C NMR spectra ($CDCl_3$, 100 MHz) of compound S20.

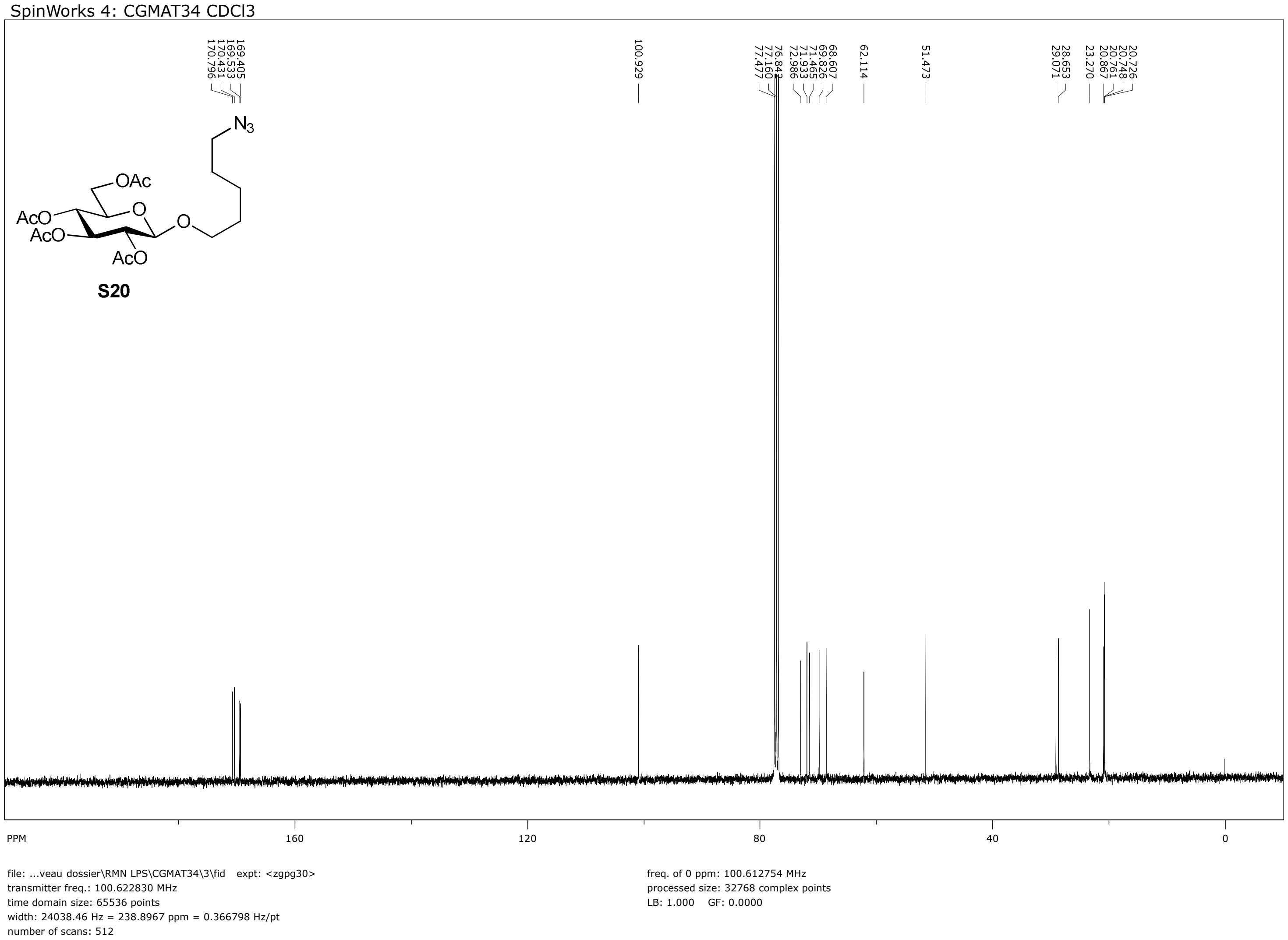

Supplementary Figure 57 │ ^{1}H NMR spectra ($CDCl_3$, 400 MHz) of compound S21.

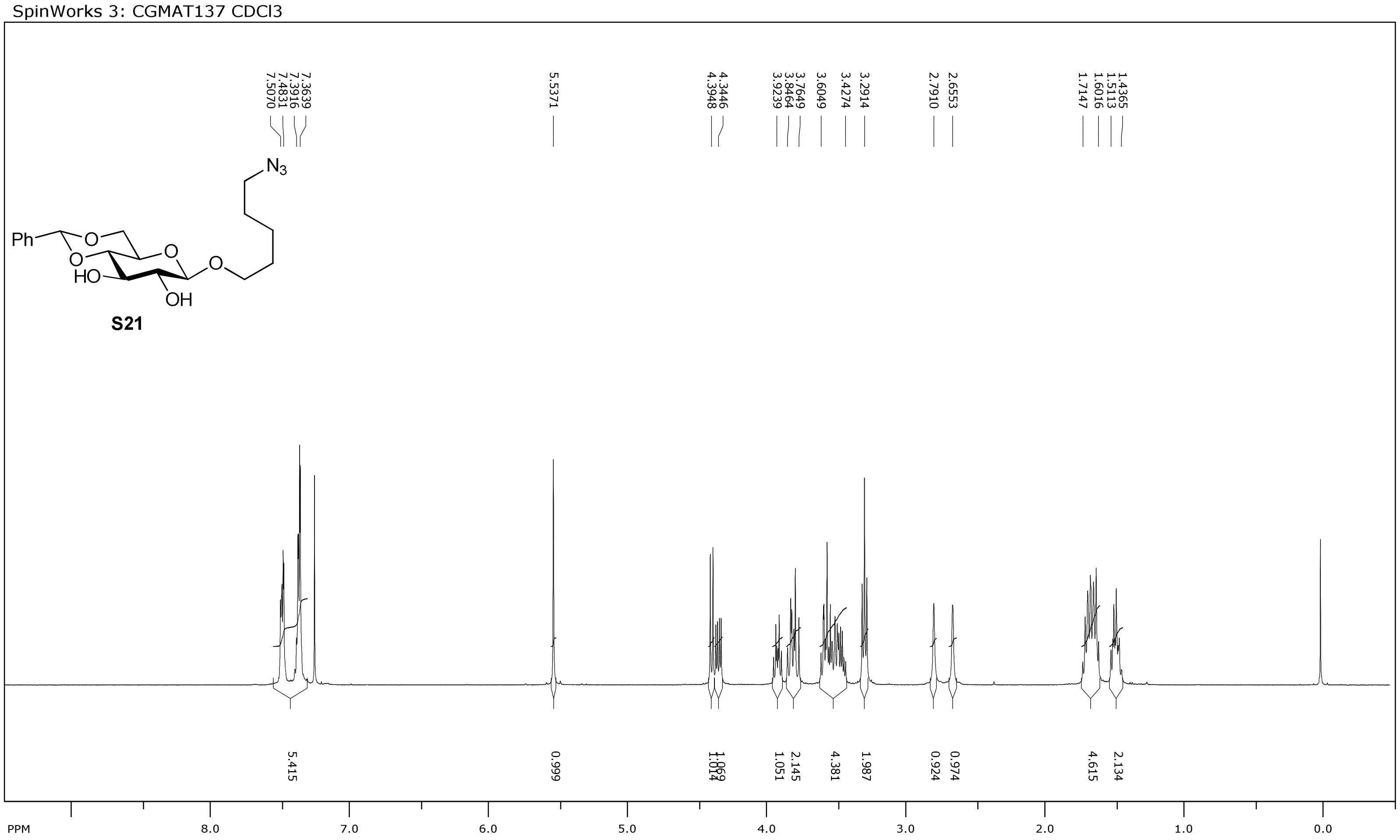

file: ...eau dossier\RMN LPS\CGMAT137\1\fid expt: <zg30>
transmitter freq.: 400.132001 MHz
time domain size: 32768 points
width: 4807.69 Hz = 12.0153 ppm = 0.146719 Hz/pt
number of scans: 16

freq. of 0 ppm: 400.130009 MHz
processed size: 32768 complex points
LB: 0.300 GF: 0.0000
Hz/cm: 160.052 ppm/cm: 0.40000

Supplementary Figure 58 │ ^{13}C NMR spectra ($CDCl_3$, 100 MHz) of compound S21.

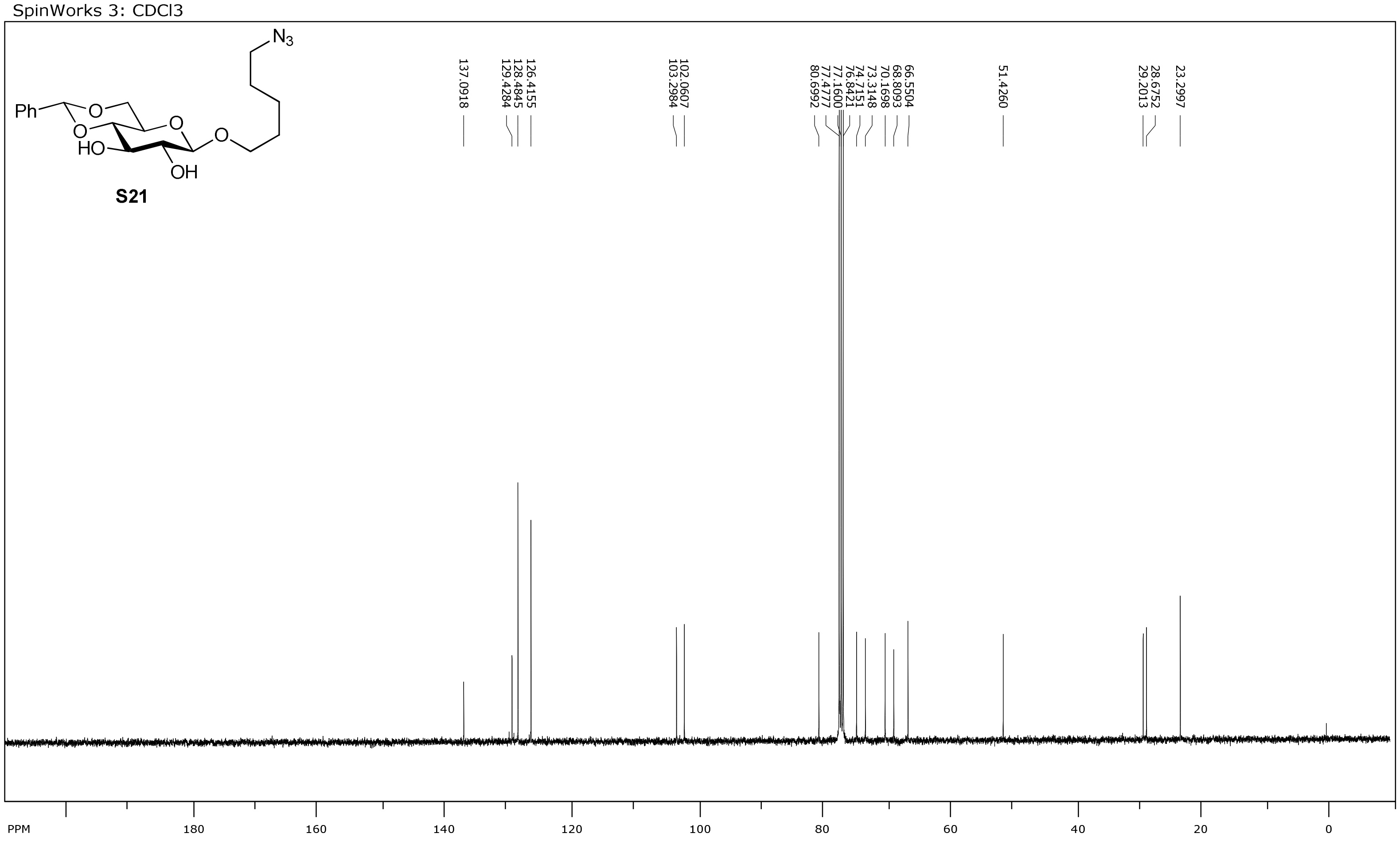

file: ...veau dossier\RMN LPS\CGMAT42\3\fid expt: <zgpg30>
transmitter freq.: 100.622830 MHz
time domain size: 65536 points
width: 24038.46 Hz = 238.8967 ppm = 0.366798 Hz/pt
number of scans: 512

freq. of 0 ppm: 100.612755 MHz
processed size: 32768 complex points
LB: 1.000 GF: 0.0000
Hz/cm: 885.392 ppm/cm: 8.79912

Supplementary Figure 59 │ ^{1}H NMR spectra ($CDCl_3$, 400 MHz) of compound 13.

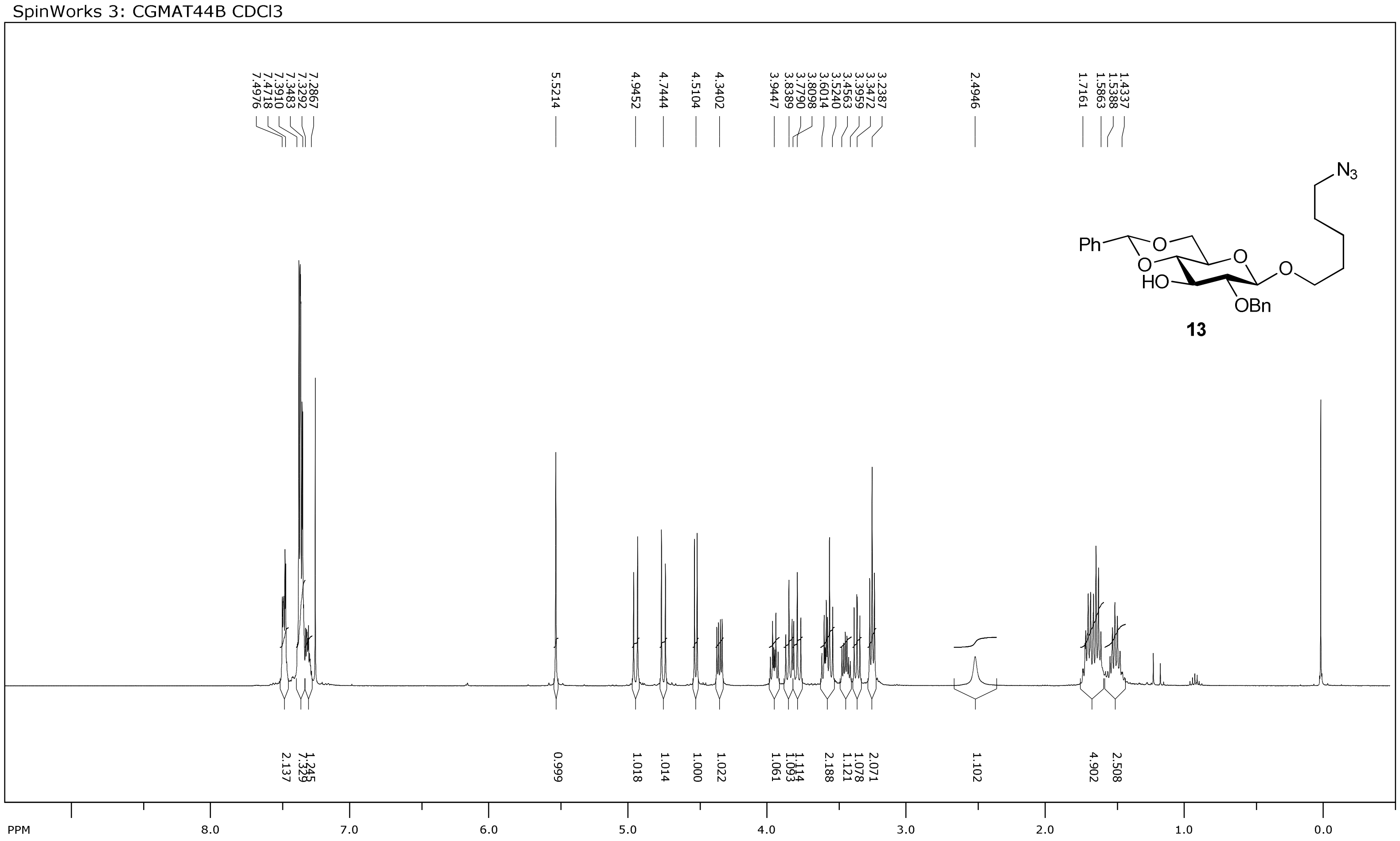

file: ...eau dossier\RMN LPS\CGMAT44B\1\fid expt: <zg30>
transmitter freq.: 400.132001 MHz
time domain size: 32768 points
width: 4807.69 Hz = 12.0153 ppm = 0.146719 Hz/pt
number of scans: 16

freq. of 0 ppm: 400.130011 MHz
processed size: 32768 complex points
LB: 0.300 GF: 0.0000
Hz/cm: 160.052 ppm/cm: 0.40000

Supplementary Figure 60 │ ^{13}C NMR spectra ($CDCl_3$, 100 MHz) of compound 13.

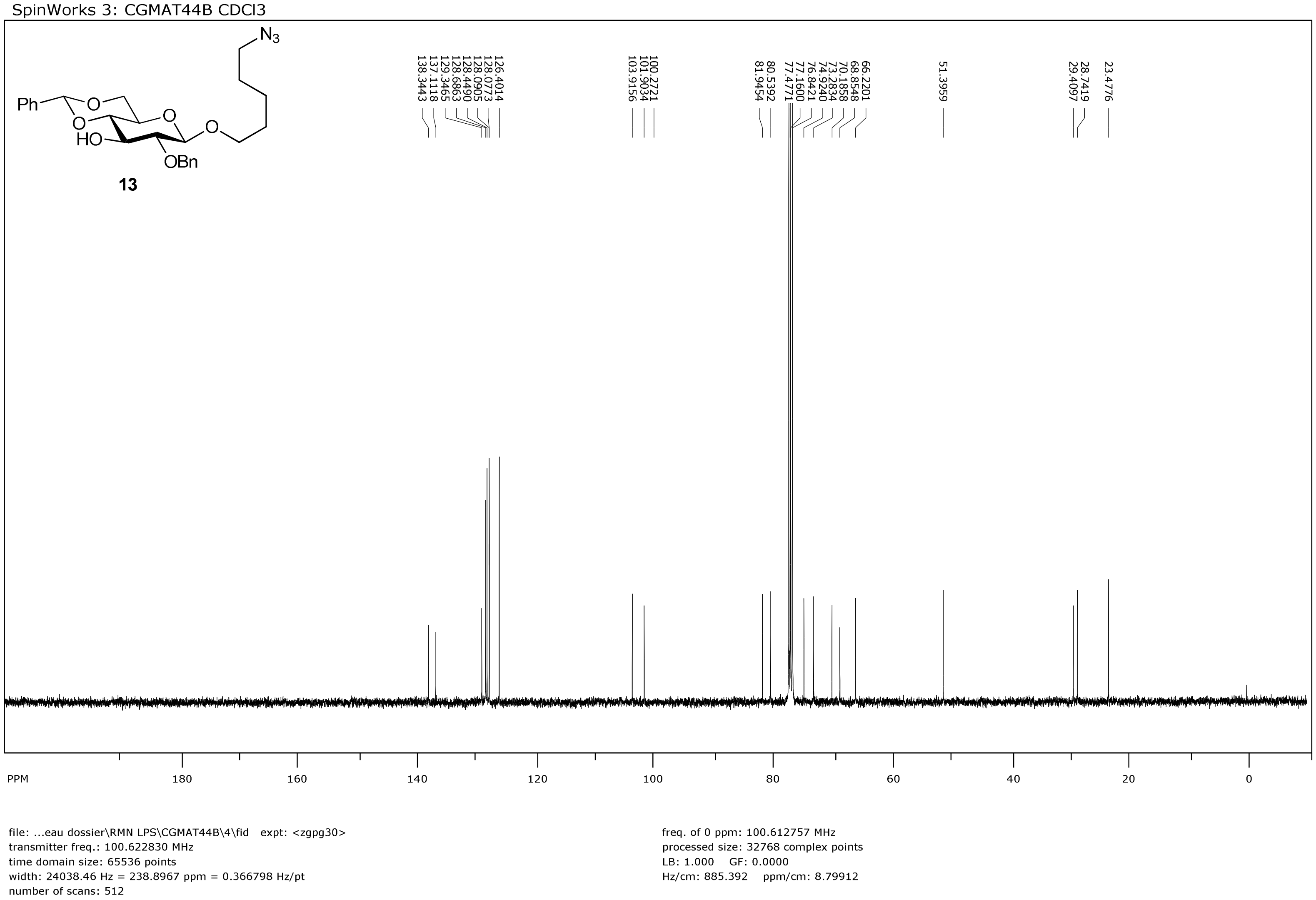

Supplementary Figure 61 │ ^{1}H NMR spectra ($CDCl_3$, 400 MHz) of compound S22.

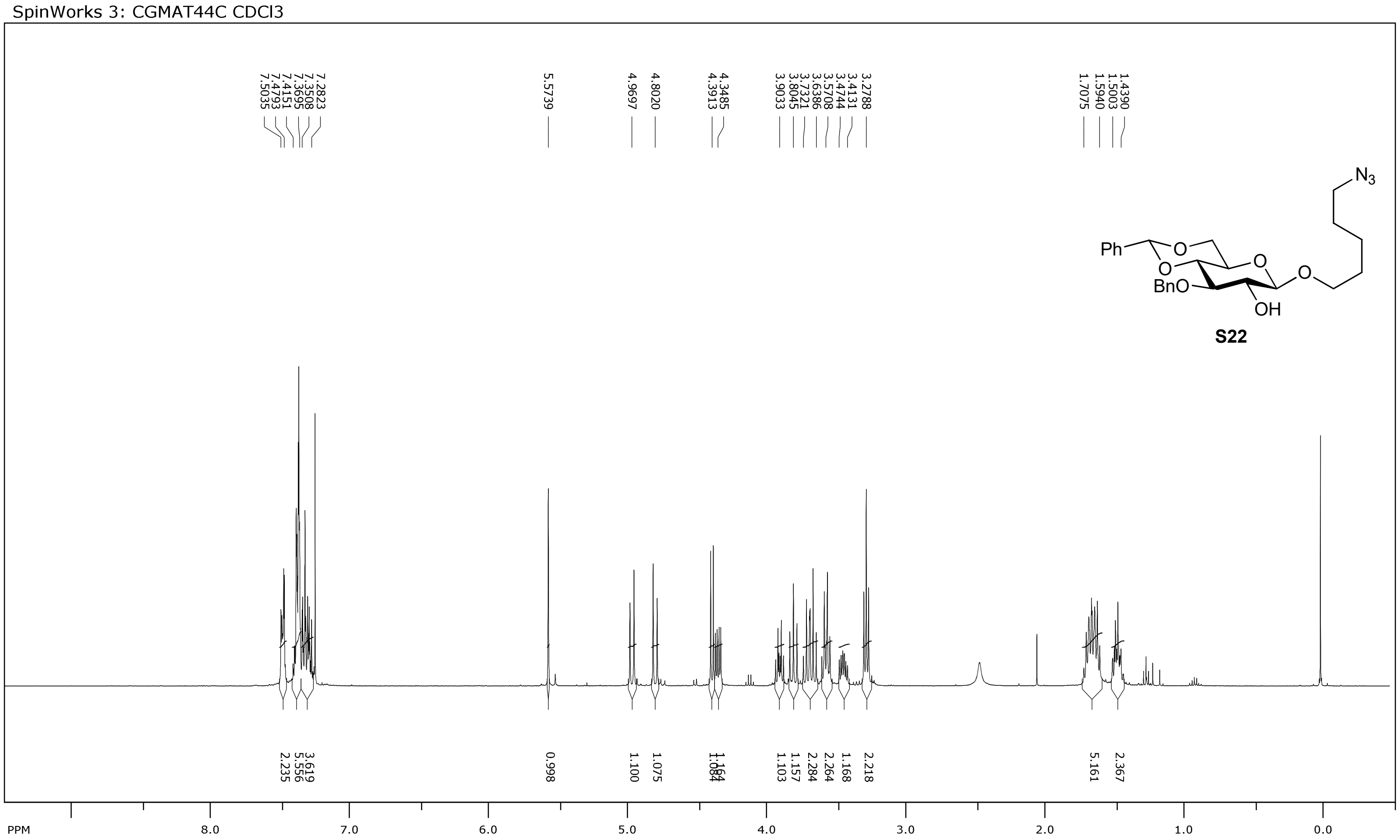

file: ...eau dossier\RMN LPS\CGMAT44C\1\fid expt: <zg30>
transmitter freq.: 400.132001 MHz
time domain size: 32768 points
width: 4807.69 Hz = 12.0153 ppm = 0.146719 Hz/pt
number of scans: 16

freq. of 0 ppm: 400.130011 MHz
processed size: 32768 complex points
LB: 0.300 GF: 0.0000
Hz/cm: 160.052 ppm/cm: 0.40000

Supplementary Figure 62 │ ^{13}C NMR spectra ($CDCl_3$, 100 MHz) of compound S22.

SpinWorks 3: CGMAT44C CDCl3

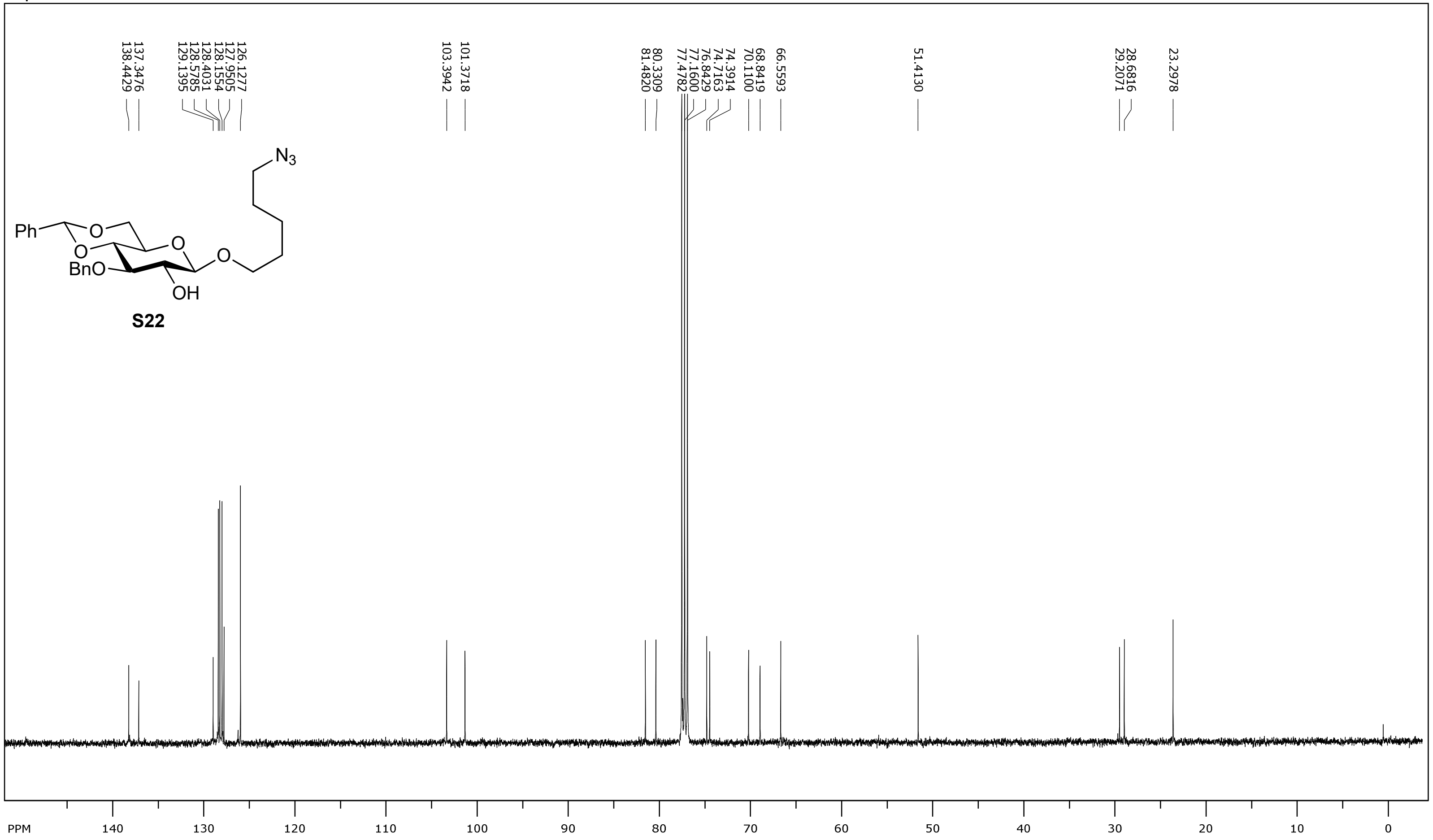

file: ...eau dossier\RMN LPS\CGMAT44C\4\fid expt: <zgpg30>
transmitter freq.: 100.622830 MHz
time domain size: 65536 points
width: 24038.46 Hz = 238.8967 ppm = 0.366798 Hz/pt
number of scans: 512

freq. of 0 ppm: 100.612757 MHz
processed size: 32768 complex points
LB: 1.000 GF: 0.0000
Hz/cm: 629.194 ppm/cm: 6.25299

Supplementary Figure 63 │ ^{1}H NMR spectra ($CDCl_3$, 400 MHz) of compound S23.

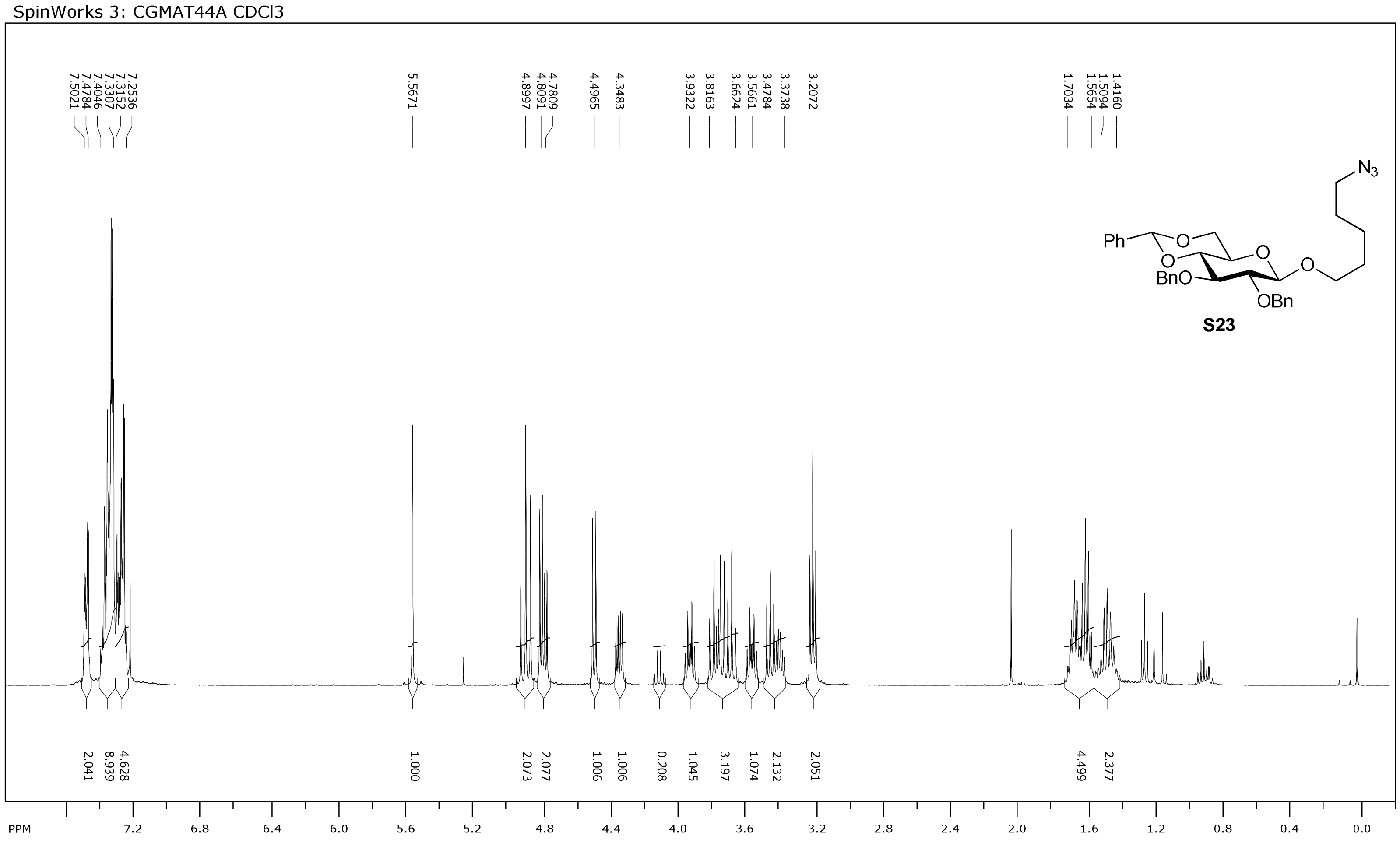

file: ...eau dossier\RMN LPS\CGMAT44A\1\fid expt: <zg30>
transmitter freq.: 400.132001 MHz
time domain size: 32768 points
width: 4807.69 Hz = 12.0153 ppm = 0.146719 Hz/pt
number of scans: 16

freq. of 0 ppm: 400.130021 MHz
processed size: 32768 complex points
LB: 0.300 GF: 0.0000
Hz/cm: 130.630 ppm/cm: 0.32647

Supplementary Figure 64 │ ^{13}C NMR spectra ($CDCl_3$, 100 MHz) of compound S23.

SpinWorks 3: CGMAT44A CDCl3

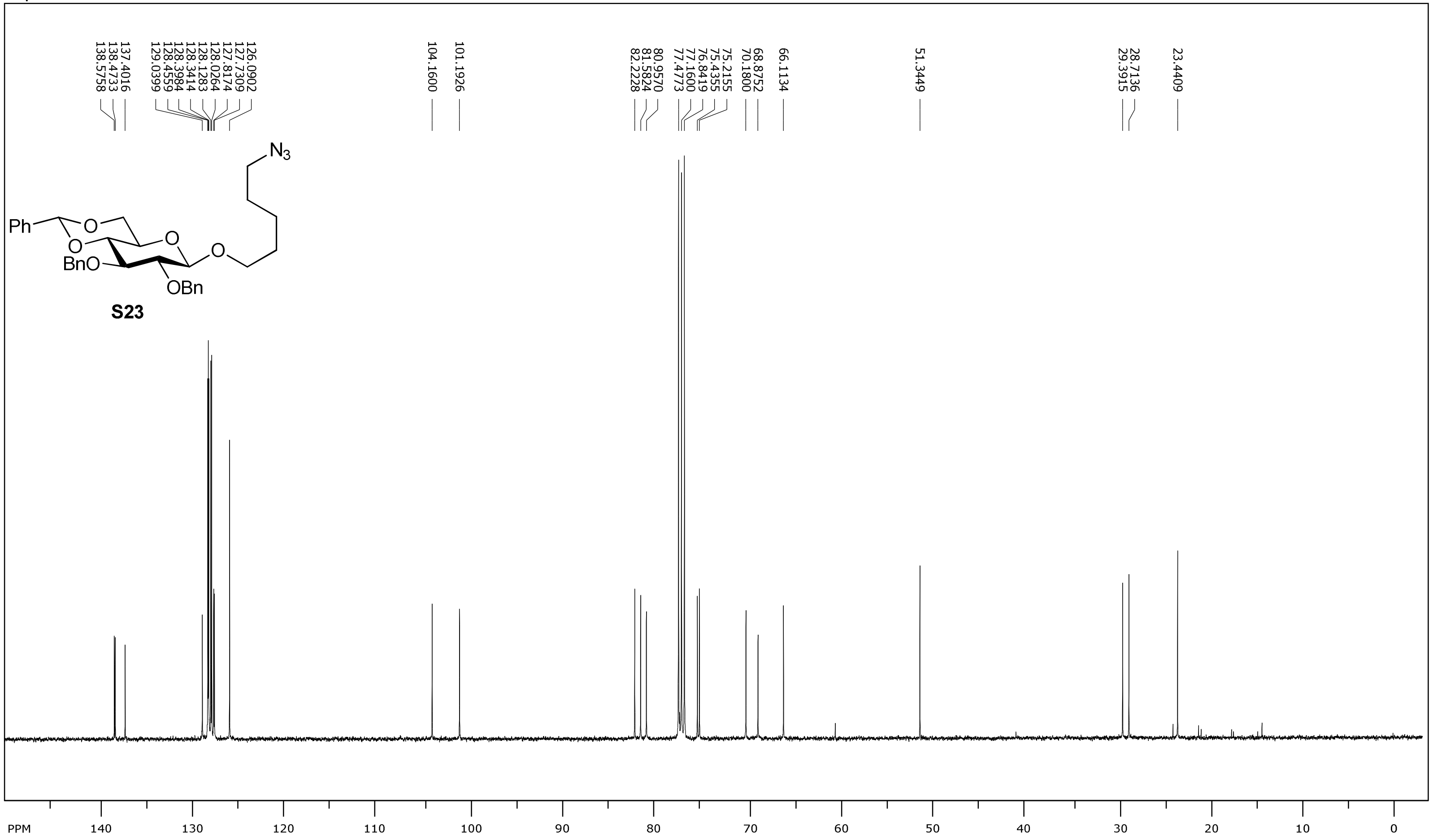

file: ...eau dossier\RMN LPS\CGMAT44A\4\fid expt: <zgpg30>
transmitter freq.: 100.622830 MHz
time domain size: 65536 points
width: 24038.46 Hz = 238.8967 ppm = 0.366798 Hz/pt
number of scans: 512

freq. of 0 ppm: 100.612763 MHz
processed size: 32768 complex points
LB: 1.000 GF: 0.0000
Hz/cm: 617.891 ppm/cm: 6.14066

Supplementary Figure 65 │ ^{1}H NMR spectra ($CDCl_3$, 400 MHz) of compound 14.

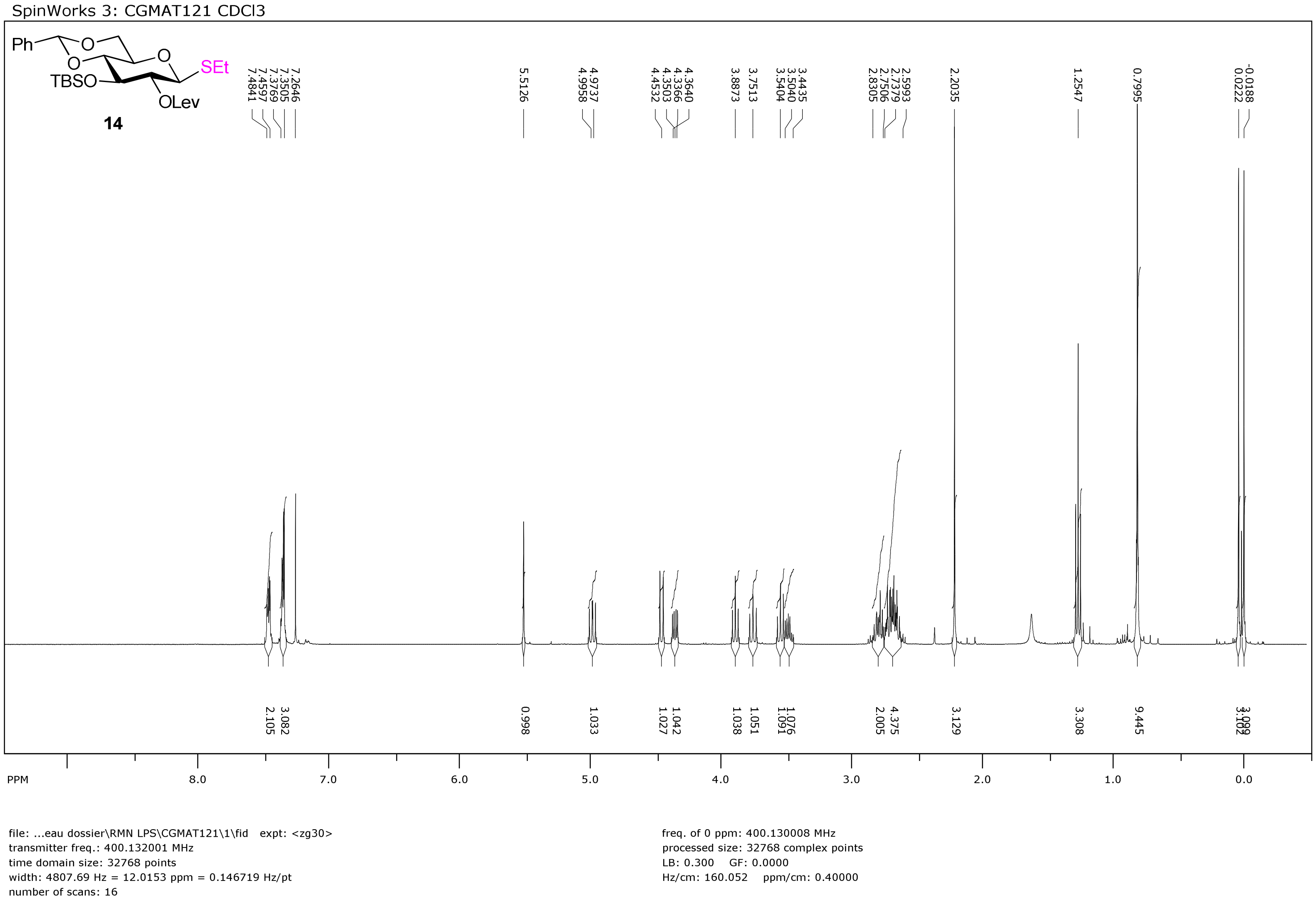

Supplementary Figure 66 | ^{13}C NMR spectra ($CDCl_3$, 100 MHz) of compound 14.

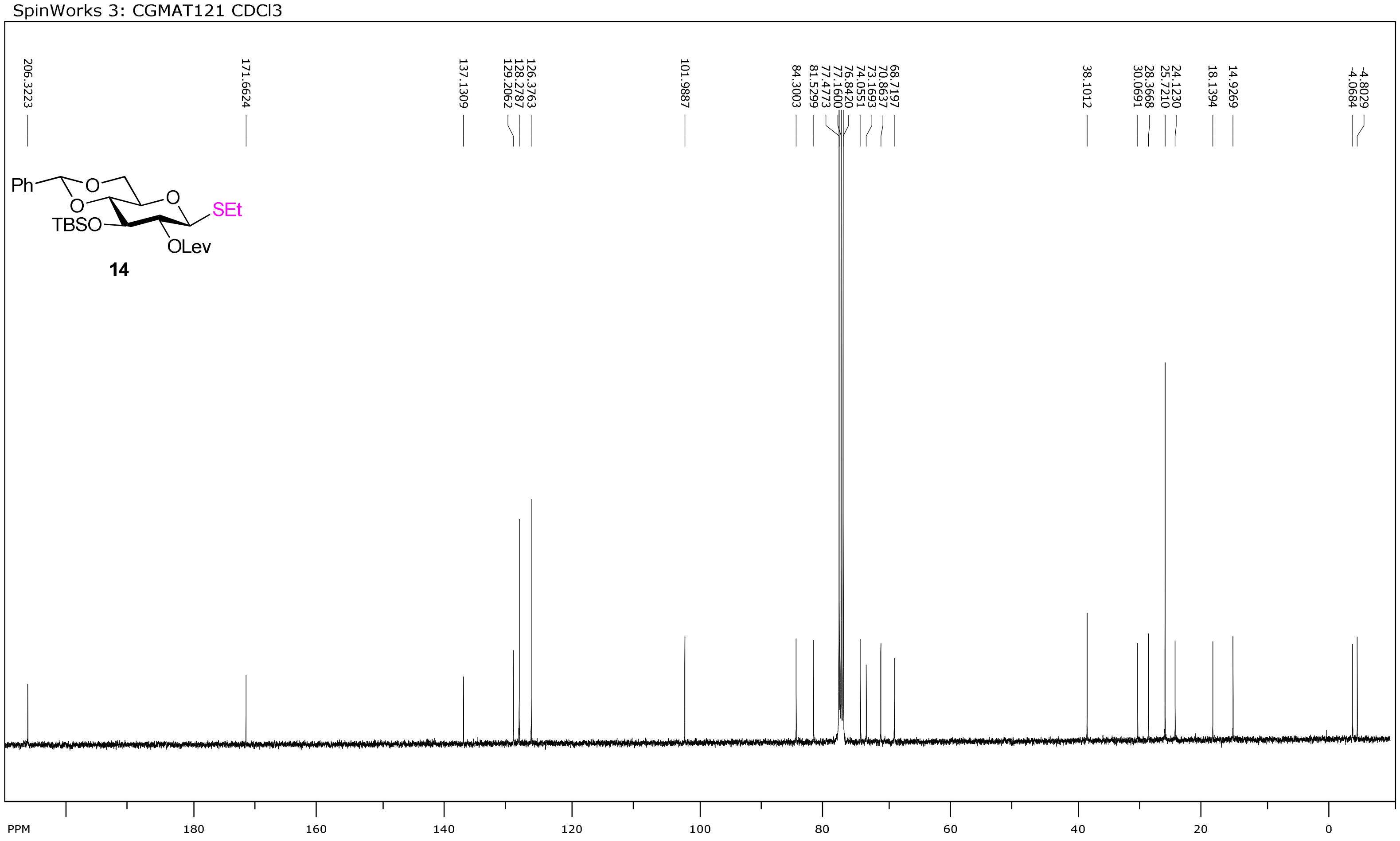

file: ...eau dossier\RMN LPS\CGMAT121\4\fid expt: <zgpg30>
transmitter freq.: 100.622830 MHz
time domain size: 65536 points
width: 24038.46 Hz = 238.8967 ppm = 0.366798 Hz/pt
number of scans: 1024

freq. of 0 ppm: 100.612756 MHz
processed size: 32768 complex points
LB: 1.000 GF: 0.0000
Hz/cm: 885.392 ppm/cm: 8.79912

Supplementary Figure 67 │ ¹H NMR spectra ($CDCl_3$, 400 MHz) of compound S25.

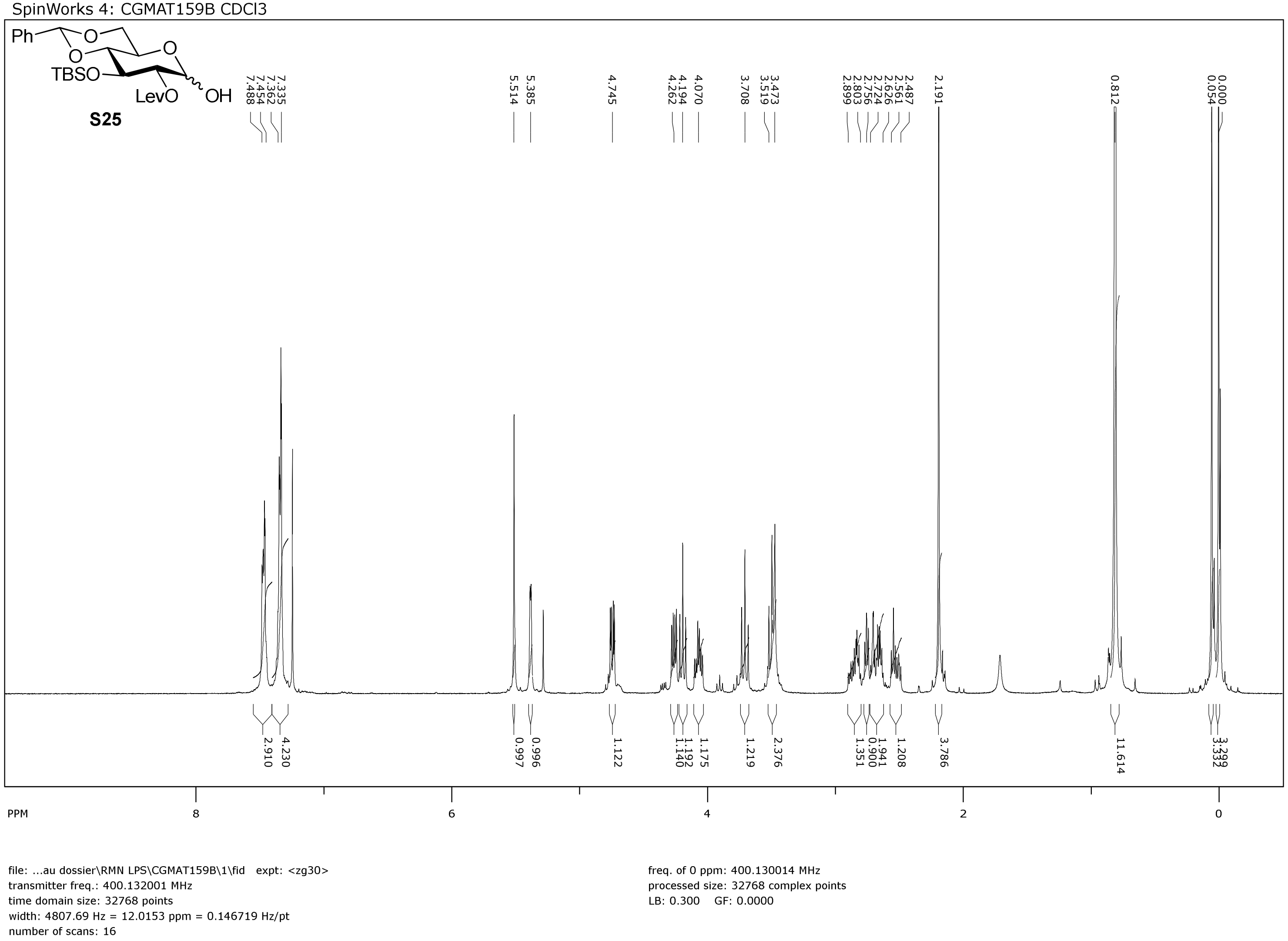

Supplementary Figure 68 │ ^{13}C NMR spectra ($CDCl_3$, 100 MHz) of compound S25.

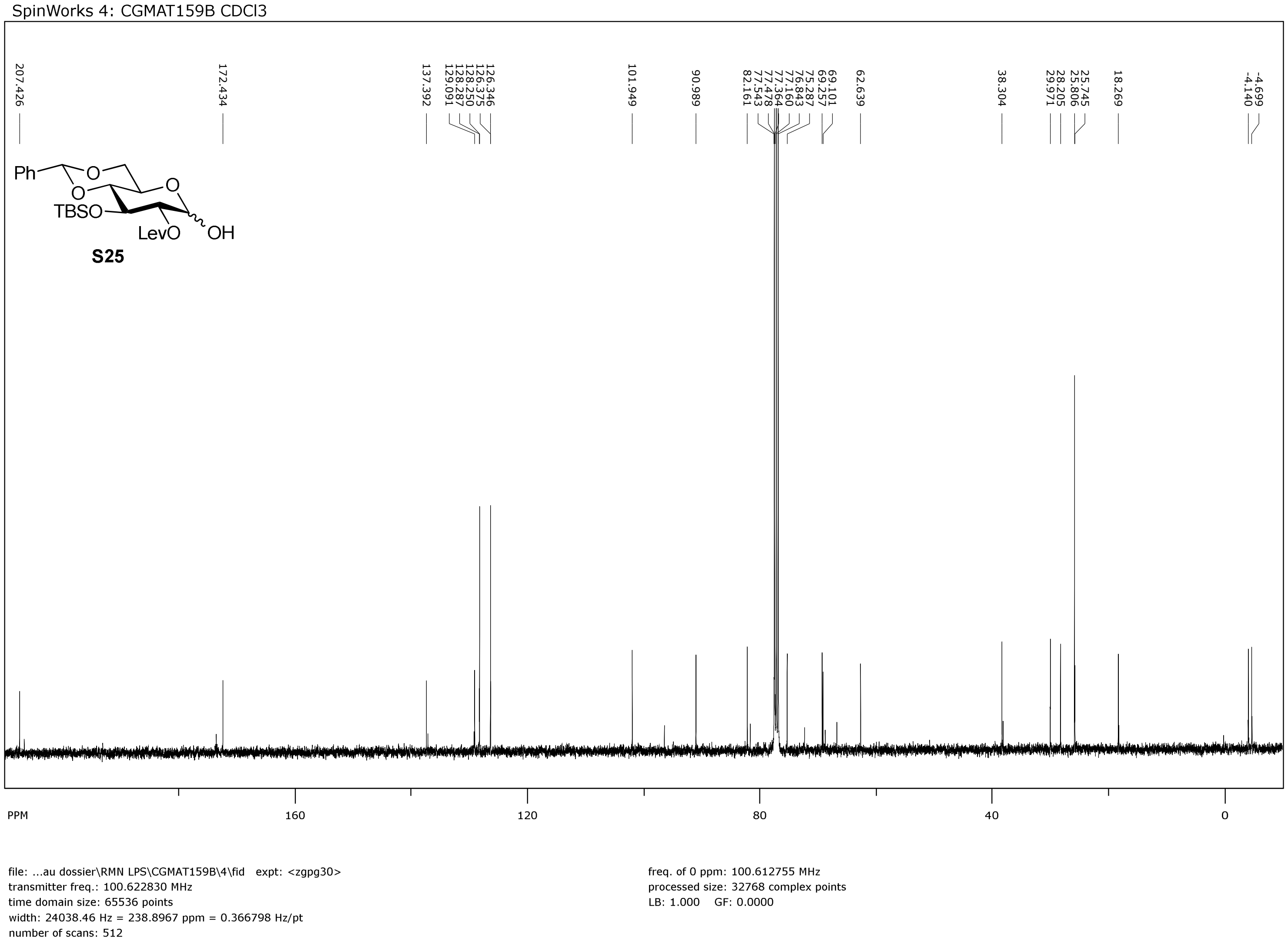

Supplementary Figure 69 │ ^{1}H NMR spectra ($CDCl_3$, 400 MHz) of compound S26.

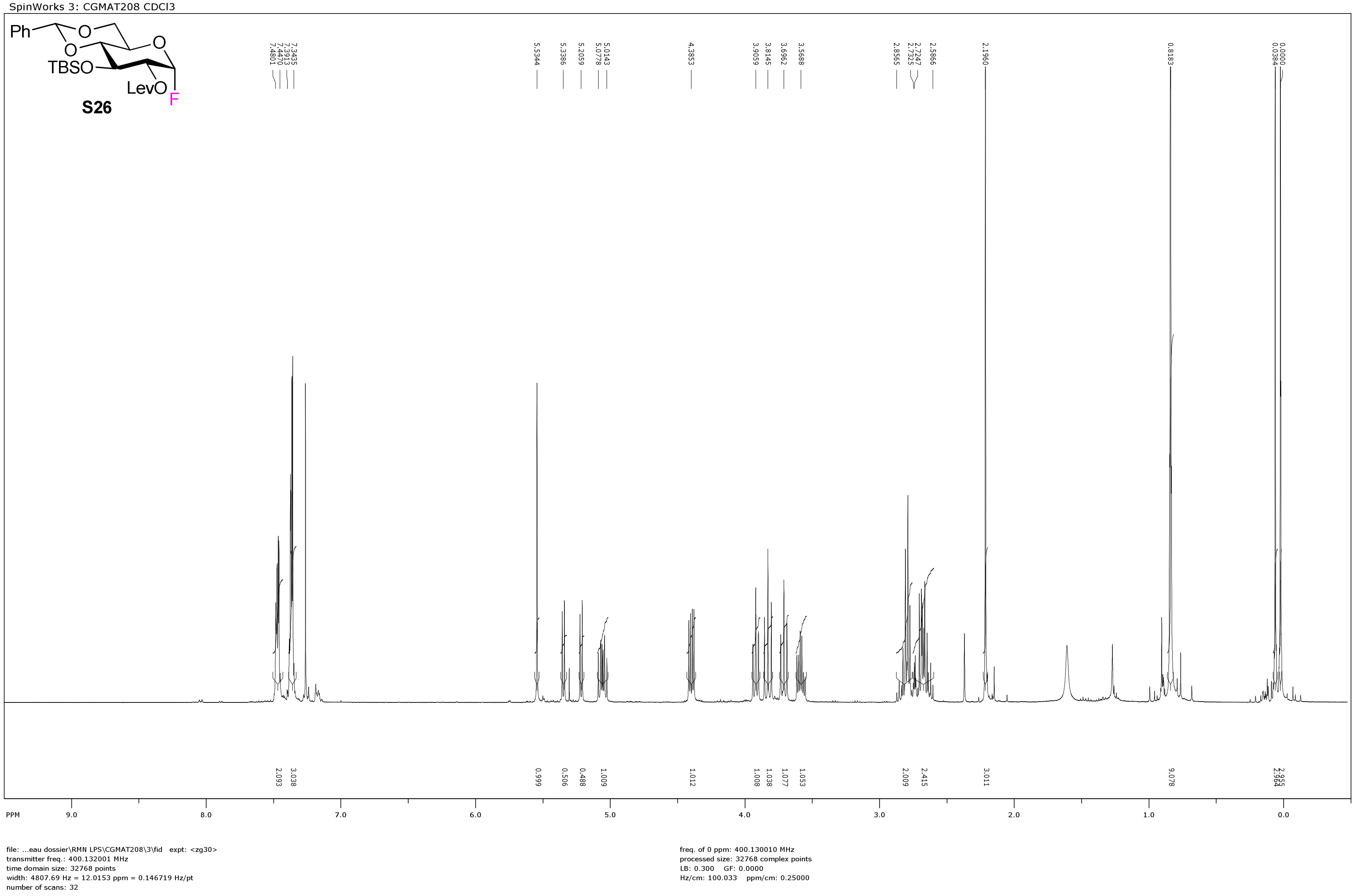

Supplementary Figure 70 | ^{13}C NMR spectra ($CDCl_3$, 100 MHz) of compound S26.

SpinWorks 3: CGMAT208 CDCl3

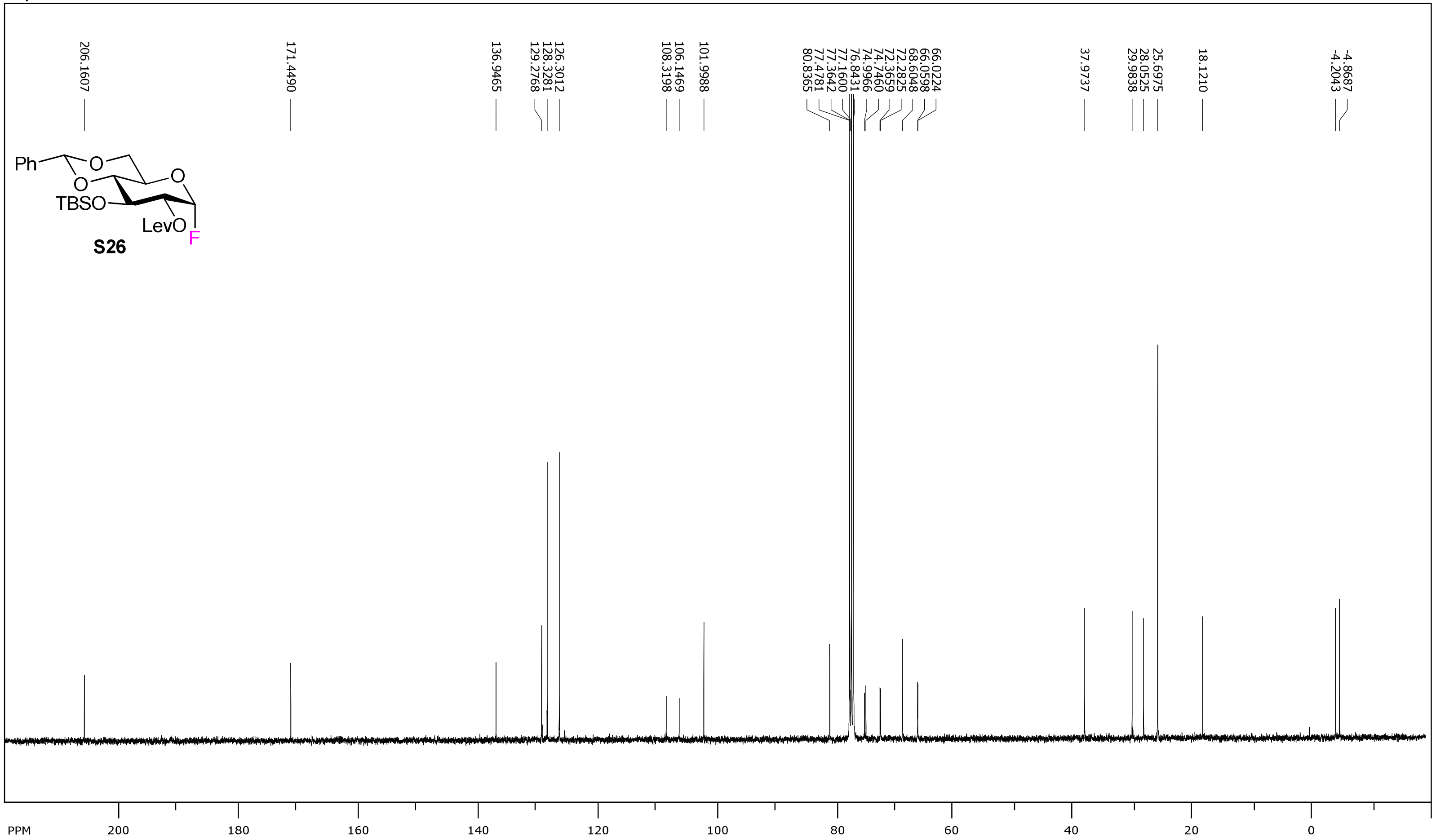

file: ...eau dossier\RMN LPS\CGMAT208\5\fid   expt: <zgpg30>
transmitter freq.: 100.622830 MHz
time domain size: 65536 points
width: 24038.46 Hz = 238.8967 ppm = 0.366798 Hz/pt
number of scans: 1024

freq. of 0 ppm: 100.612755 MHz
processed size: 32768 complex points
LB: 1.000   GF: 0.0000
Hz/cm: 961.538   ppm/cm: 9.55587

Supplementary Figure 71 | ^{1}H NMR spectra ($CDCl_3$, 400 MHz) of compound S27.

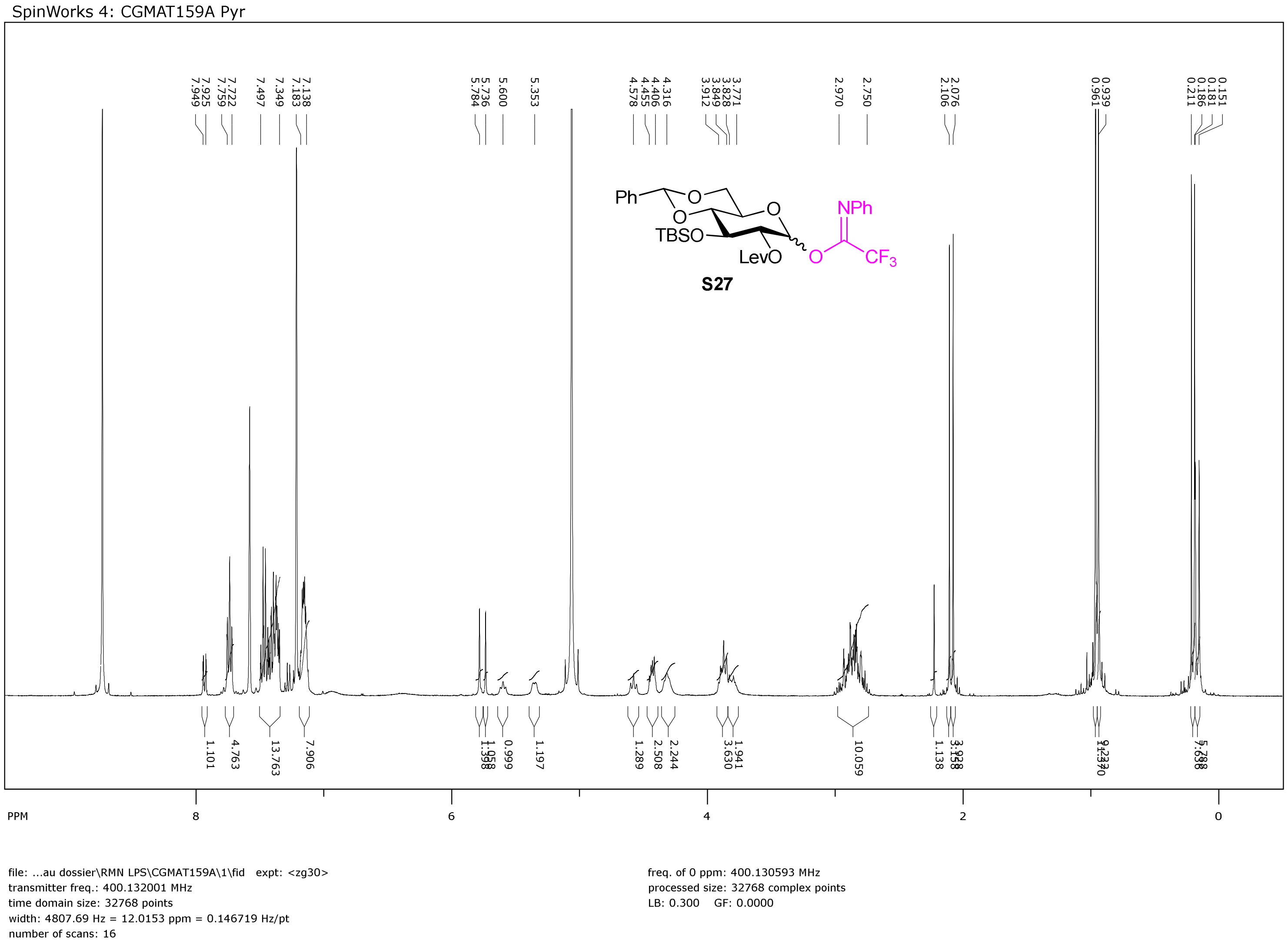

Supplementary Figure 72 | ^{13}C NMR spectra ($CDCl_3$, 100 MHz) of compound S27.

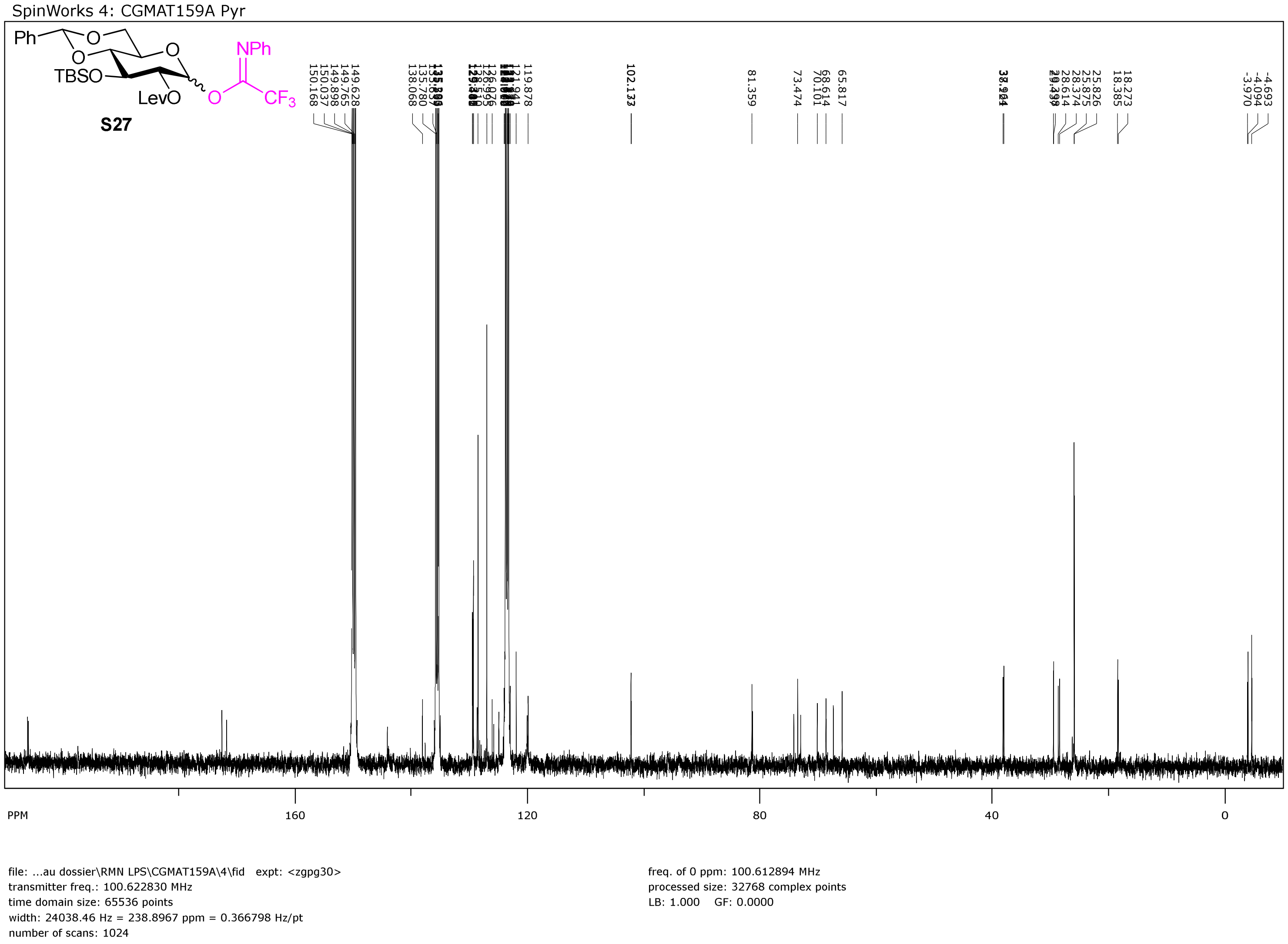

Supplementary Figure 73 | ^{1}H NMR spectra ($CDCl_3$, 400 MHz) of compound 15.

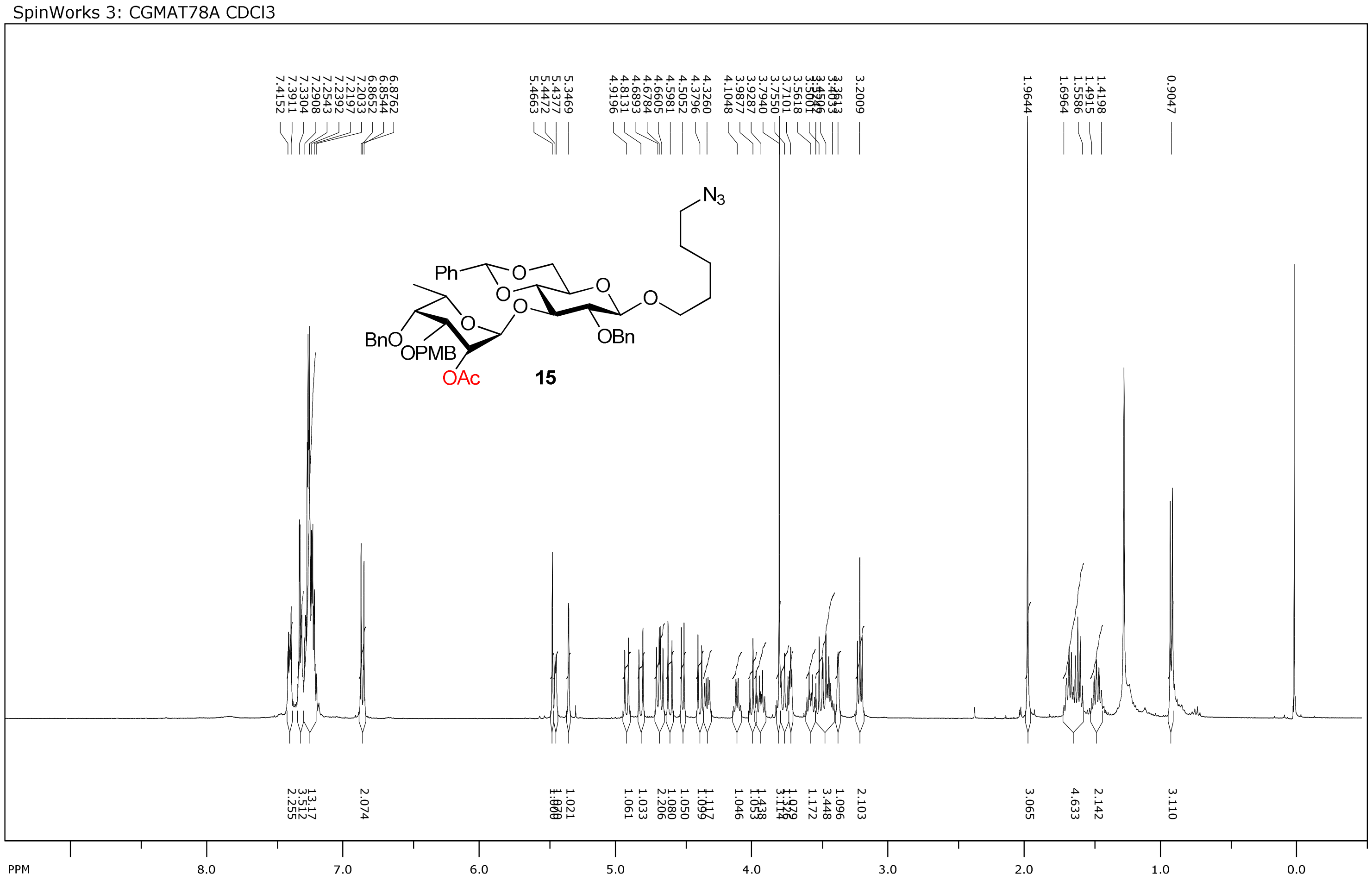

file: D:\RMN LPS\CGMAT78A\1\fid expt: <zg30>
transmitter freq.: 400.132001 MHz
time domain size: 32768 points
width: 4807.69 Hz = 12.0153 ppm = 0.146719 Hz/pt
number of scans: 16

freq. of 0 ppm: 400.130012 MHz
processed size: 32768 complex points
LB: 0.300 GF: 0.0000
Hz/cm: 160.052 ppm/cm: 0.40000

Supplementary Figure 74 | ^{13}C NMR spectra ($CDCl_3$, 100 MHz) of compound 15.

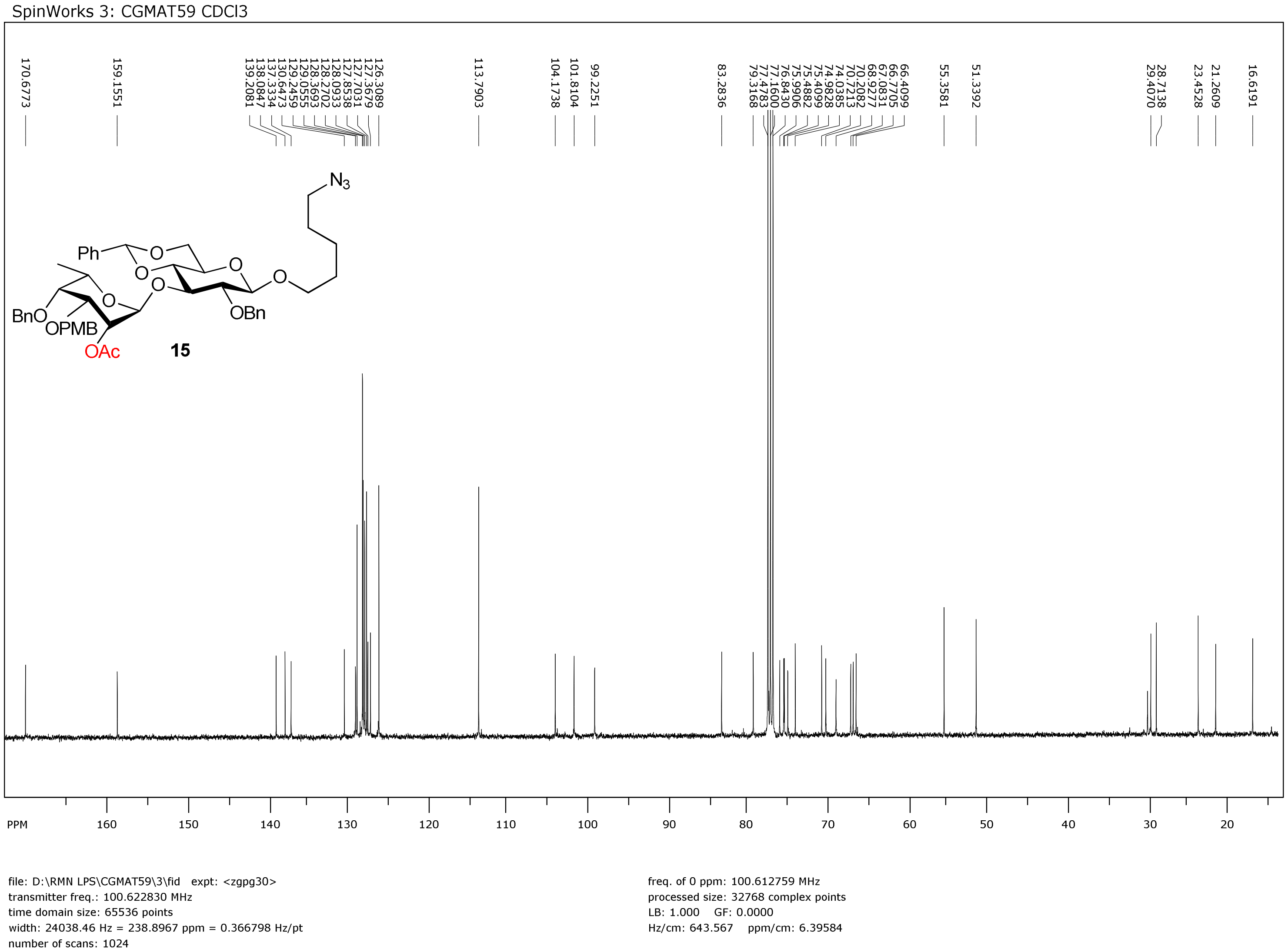

Supplementary Figure 75 | ^{1}H NMR spectra ($CDCl_3$, 400 MHz) of compound 16.

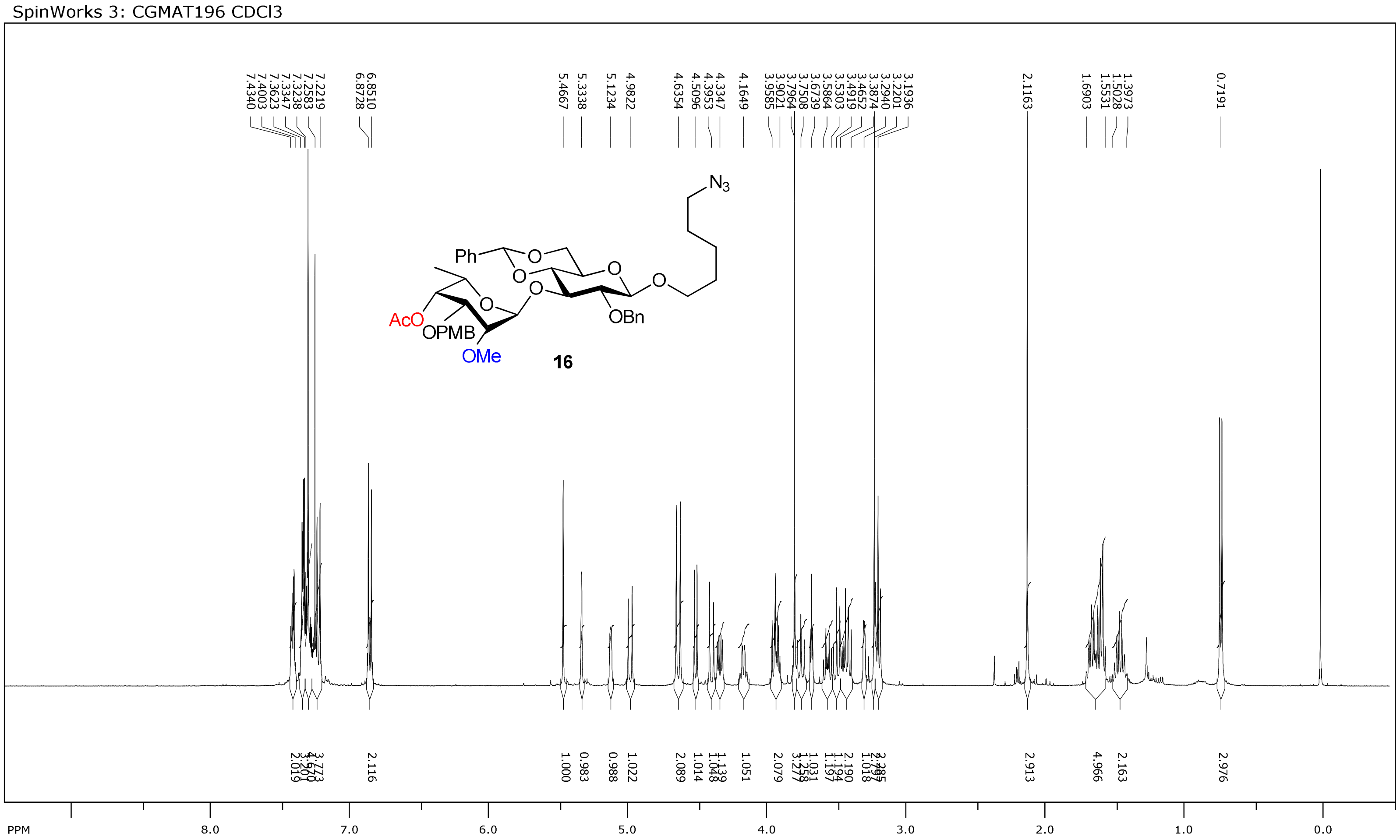

file: ...eau dossier\RMN LPS\CGMAT196\1\fid expt: <zg30>
transmitter freq.: 400.132001 MHz
time domain size: 32768 points
width: 4807.69 Hz = 12.0153 ppm = 0.146719 Hz/pt
number of scans: 32

freq. of 0 ppm: 400.130010 MHz
processed size: 32768 complex points
LB: 0.300 GF: 0.0000
Hz/cm: 160.052 ppm/cm: 0.40000

Supplementary Figure 76 | ^{13}C NMR spectra ($CDCl_3$, 100 MHz) of compound 16.

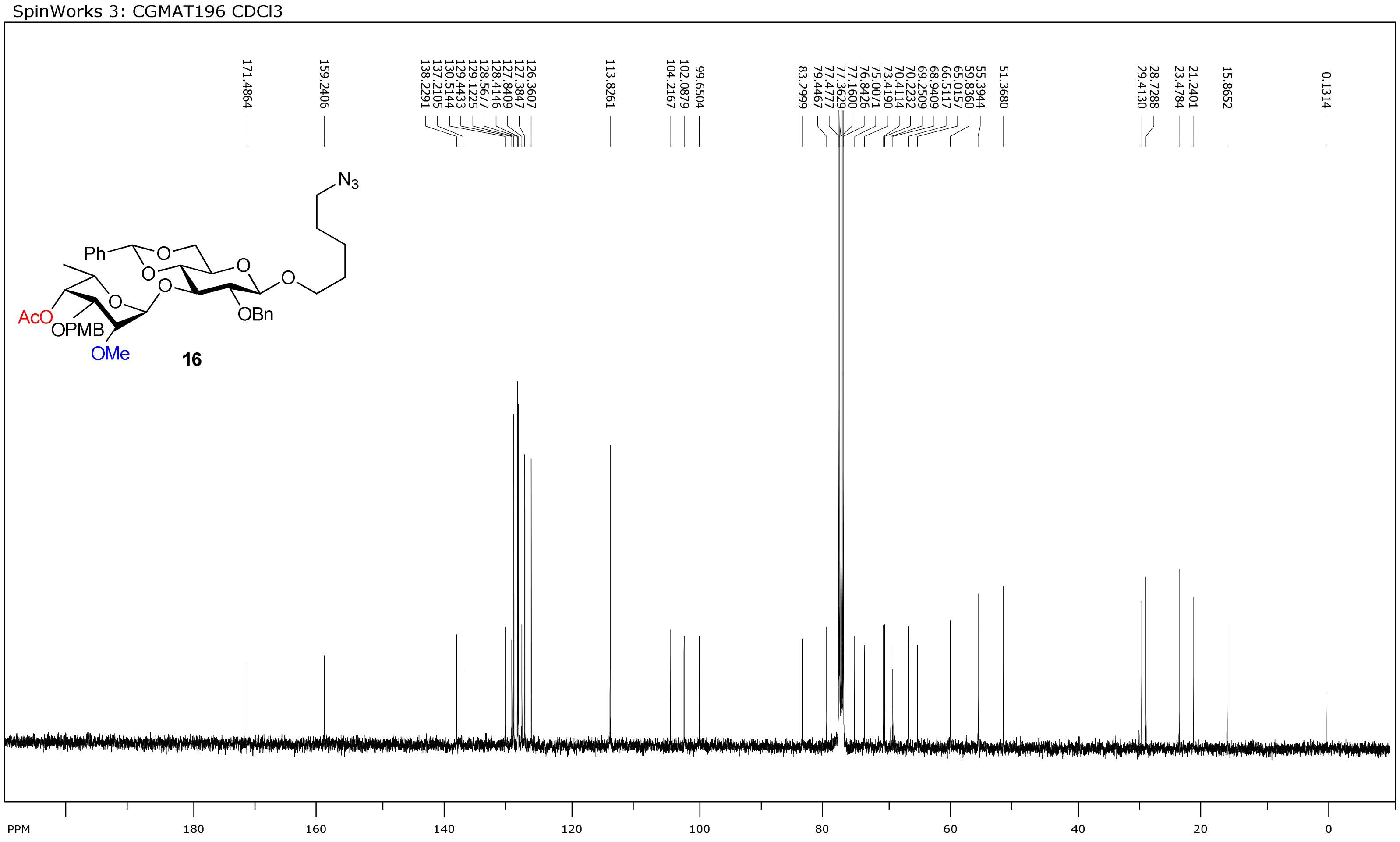

file: ...eau dossier\RMN LPS\CGMAT196\3\fid expt: <zgpg30>
transmitter freq.: 100.622830 MHz
time domain size: 65536 points
width: 24038.46 Hz = 238.8967 ppm = 0.366798 Hz/pt
number of scans: 1024

freq. of 0 ppm: 100.612755 MHz
processed size: 32768 complex points
LB: 1.000 GF: 0.0000
Hz/cm: 885.392 ppm/cm: 8.79912

Supplementary Figure 77 | ^{1}H NMR spectra ($CDCl_3$, 400 MHz) of compound 17.

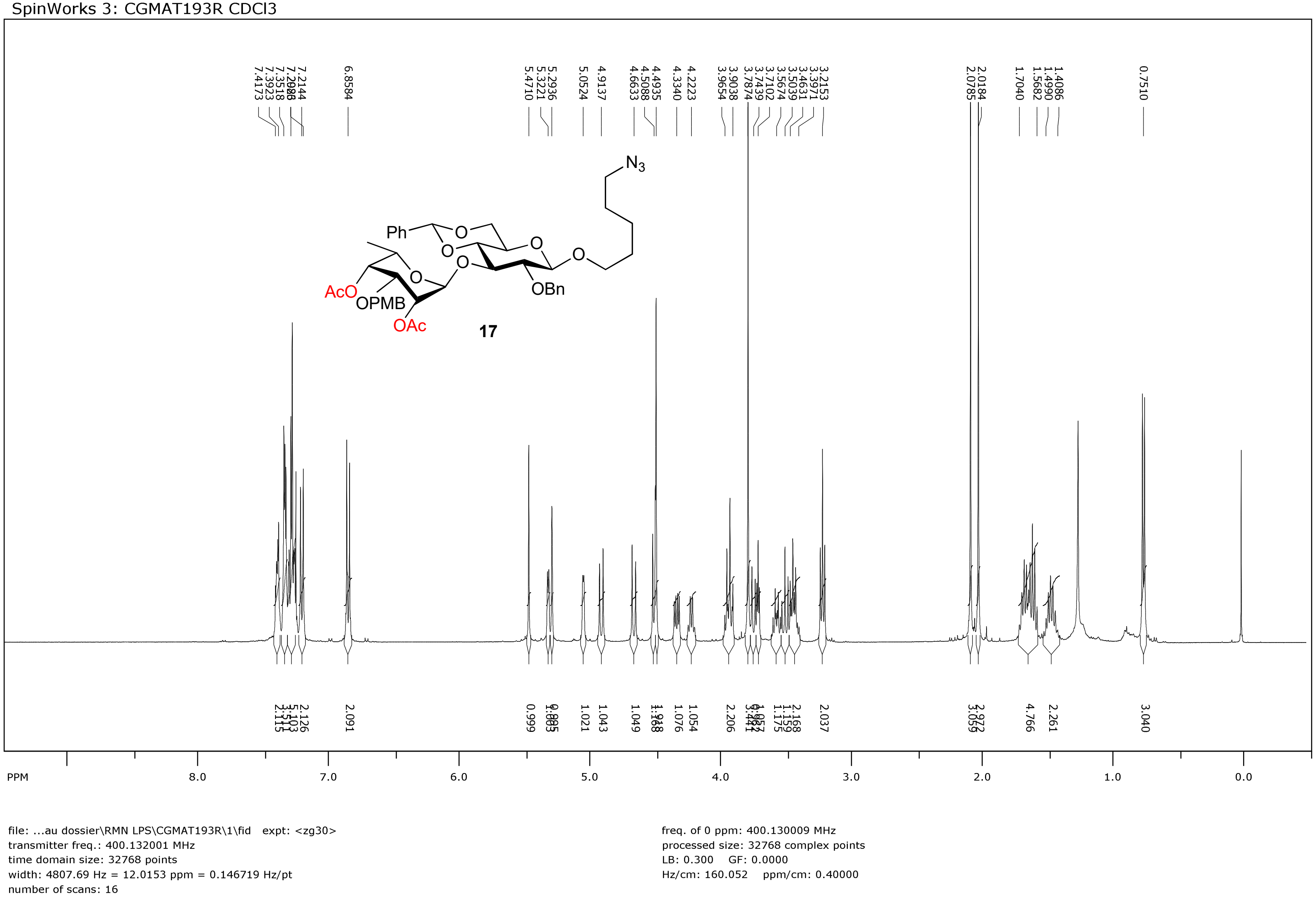

Supplementary Figure 78 | ^{13}C NMR spectra ($CDCl_3$, 100 MHz) of compound 17.

SpinWorks 3: CGMAT193R CDCl3

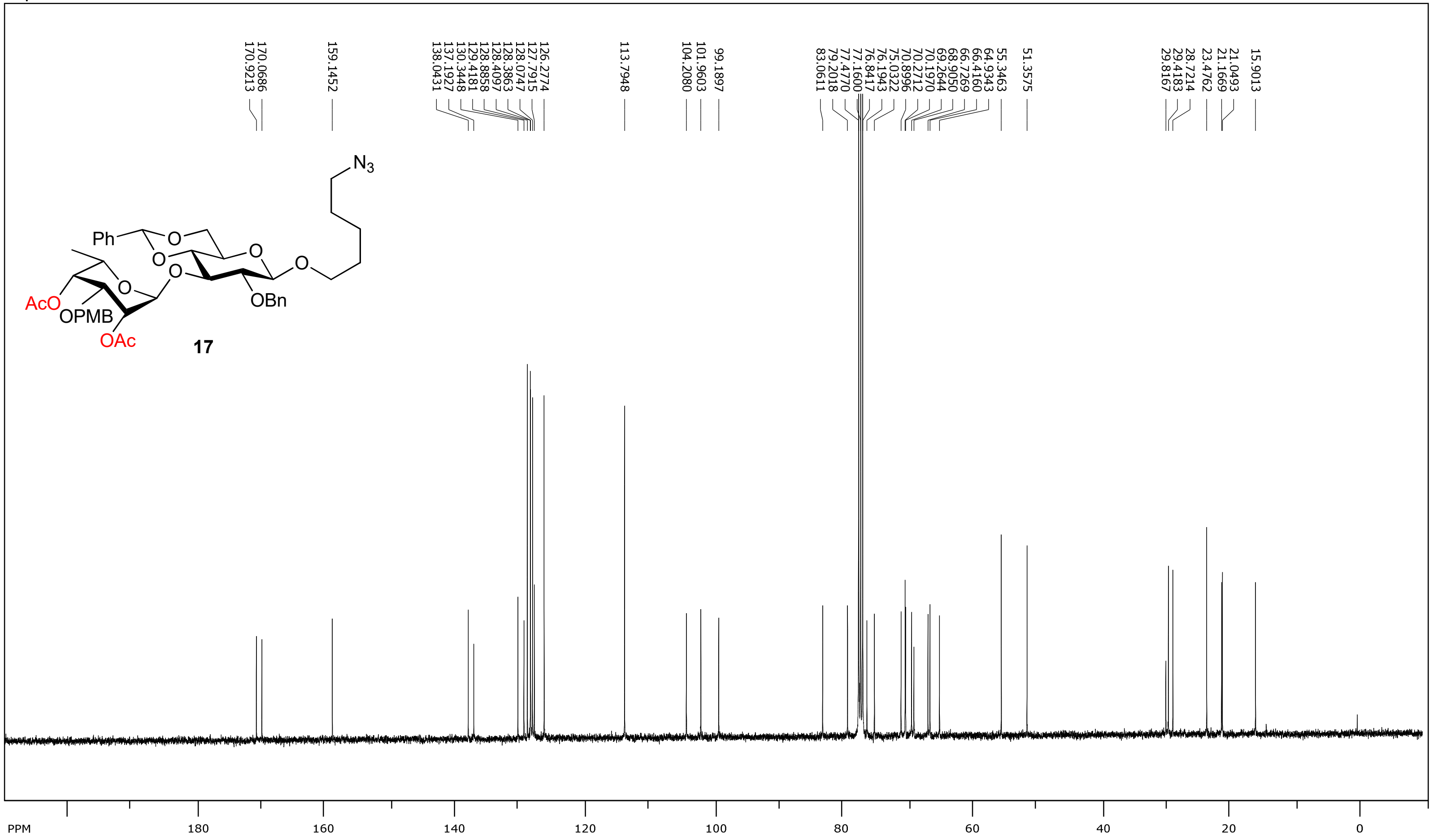

file: ...au dossier\RMN LPS\CGMAT193R\4\fid expt: <zgpg30>
transmitter freq.: 100.622830 MHz
time domain size: 65536 points
width: 24038.46 Hz = 238.8967 ppm = 0.366798 Hz/pt
number of scans: 1024

freq. of 0 ppm: 100.612757 MHz
processed size: 32768 complex points
LB: 1.000 GF: 0.0000
Hz/cm: 885.392 ppm/cm: 8.79912

Supplementary Figure 79 | ^{1}H NMR spectra ($CDCl_3$, 400 MHz) of compound 18.

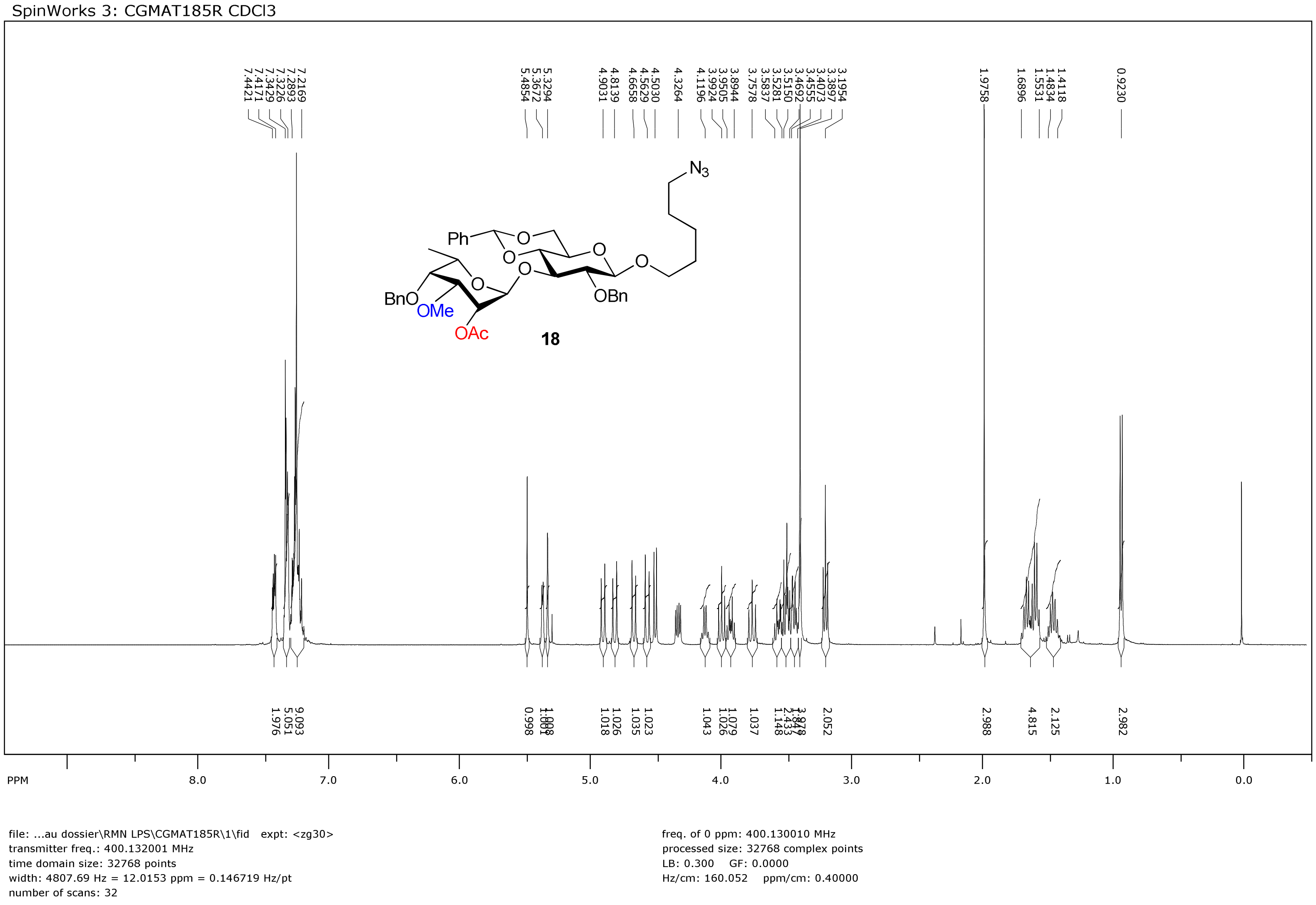

Supplementary Figure 80 | ^{13}C NMR spectra ($CDCl_3$, 100 MHz) of compound 18.

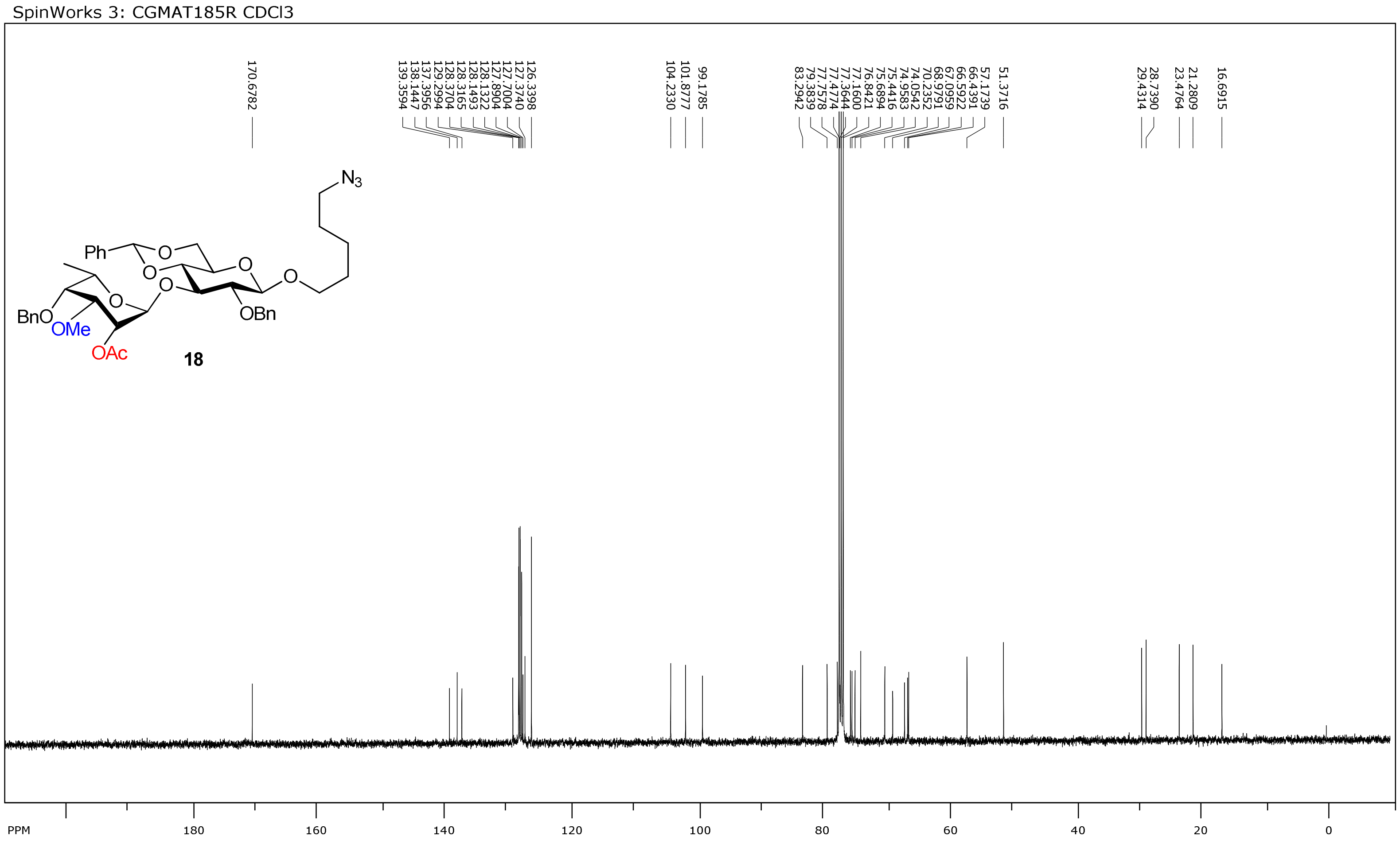

file: ...au dossier\RMN LPS\CGMAT185R\3\fid expt: <zgpg30>
transmitter freq.: 100.622830 MHz
time domain size: 65536 points
width: 24038.46 Hz = 238.8967 ppm = 0.366798 Hz/pt
number of scans: 1024

freq. of 0 ppm: 100.612755 MHz
processed size: 32768 complex points
LB: 1.000 GF: 0.0000
Hz/cm: 885.392 ppm/cm: 8.79912

Supplementary Figure 81 | ^{1}H NMR spectra ($CDCl_3$, 400 MHz) of compound 19.

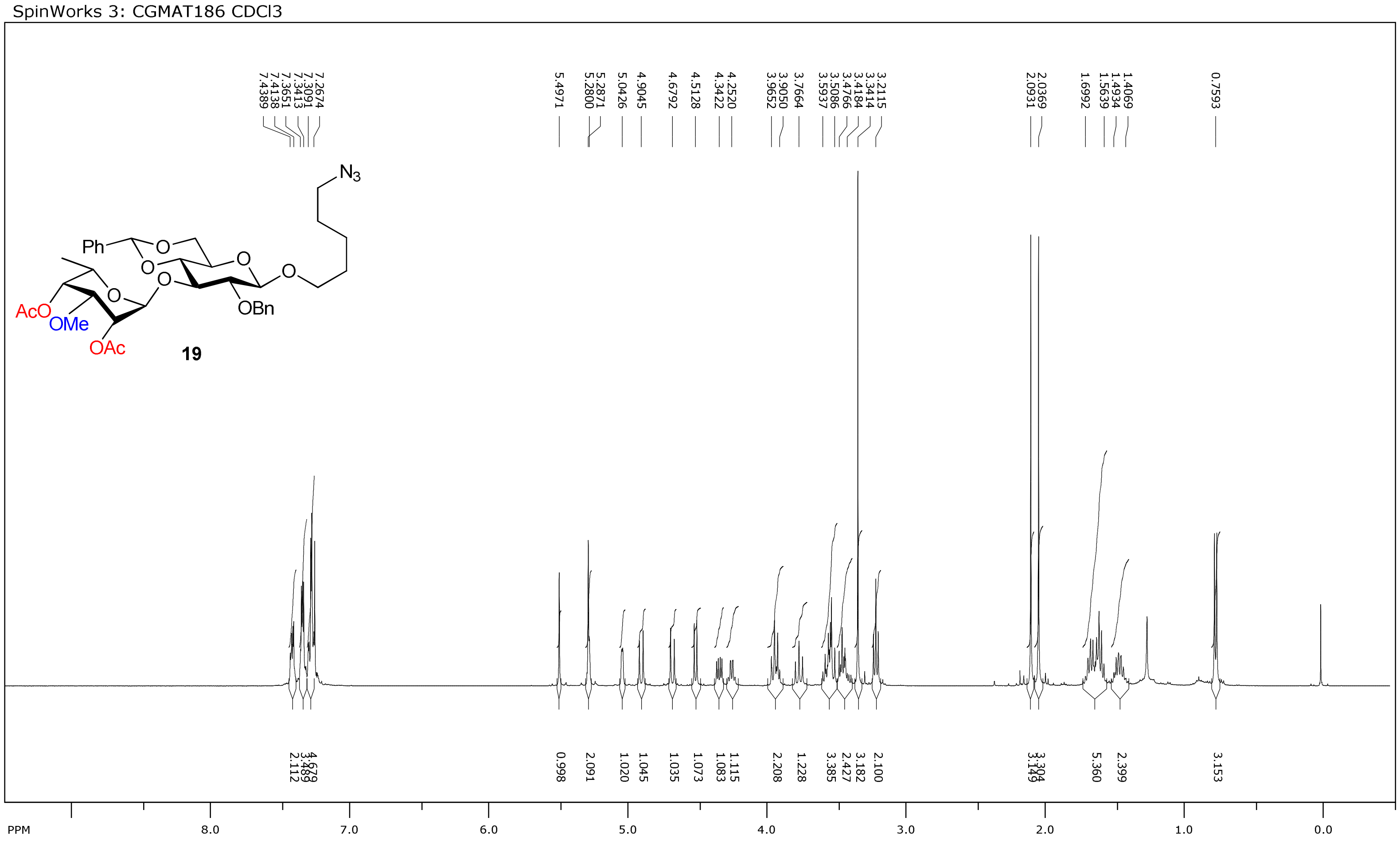

file: ...eau dossier\RMN LPS\CGMAT186\1\fid expt: <zg30>
transmitter freq.: 400.132001 MHz
time domain size: 32768 points
width: 4807.69 Hz = 12.0153 ppm = 0.146719 Hz/pt
number of scans: 16

freq. of 0 ppm: 400.130009 MHz
processed size: 32768 complex points
LB: 0.300 GF: 0.0000
Hz/cm: 160.052 ppm/cm: 0.40000

Supplementary Figure 82 | ^{13}C NMR spectra ($CDCl_3$, 100 MHz) of compound 19.

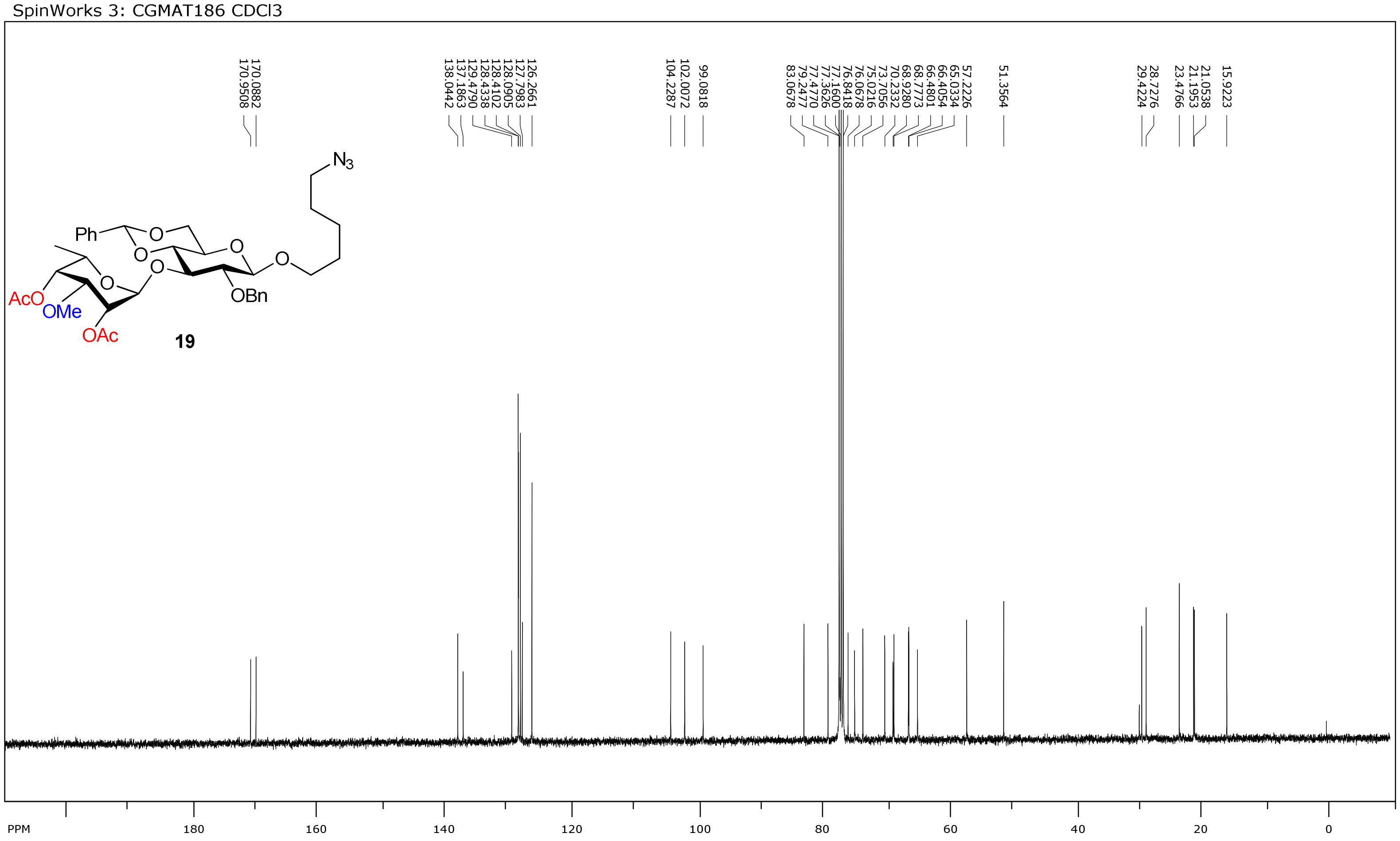

file: ...eau dossier\RMN LPS\CGMAT186\4\fid expt: <zgpg30>
transmitter freq.: 100.622830 MHz
time domain size: 65536 points
width: 24038.46 Hz = 238.8967 ppm = 0.366798 Hz/pt
number of scans: 1024

freq. of 0 ppm: 100.612757 MHz
processed size: 32768 complex points
LB: 1.000 GF: 0.0000
Hz/cm: 885.392 ppm/cm: 8.79912

Supplementary Figure 83 | ^{1}H NMR spectra ($CDCl_3$, 400 MHz) of compound 20.

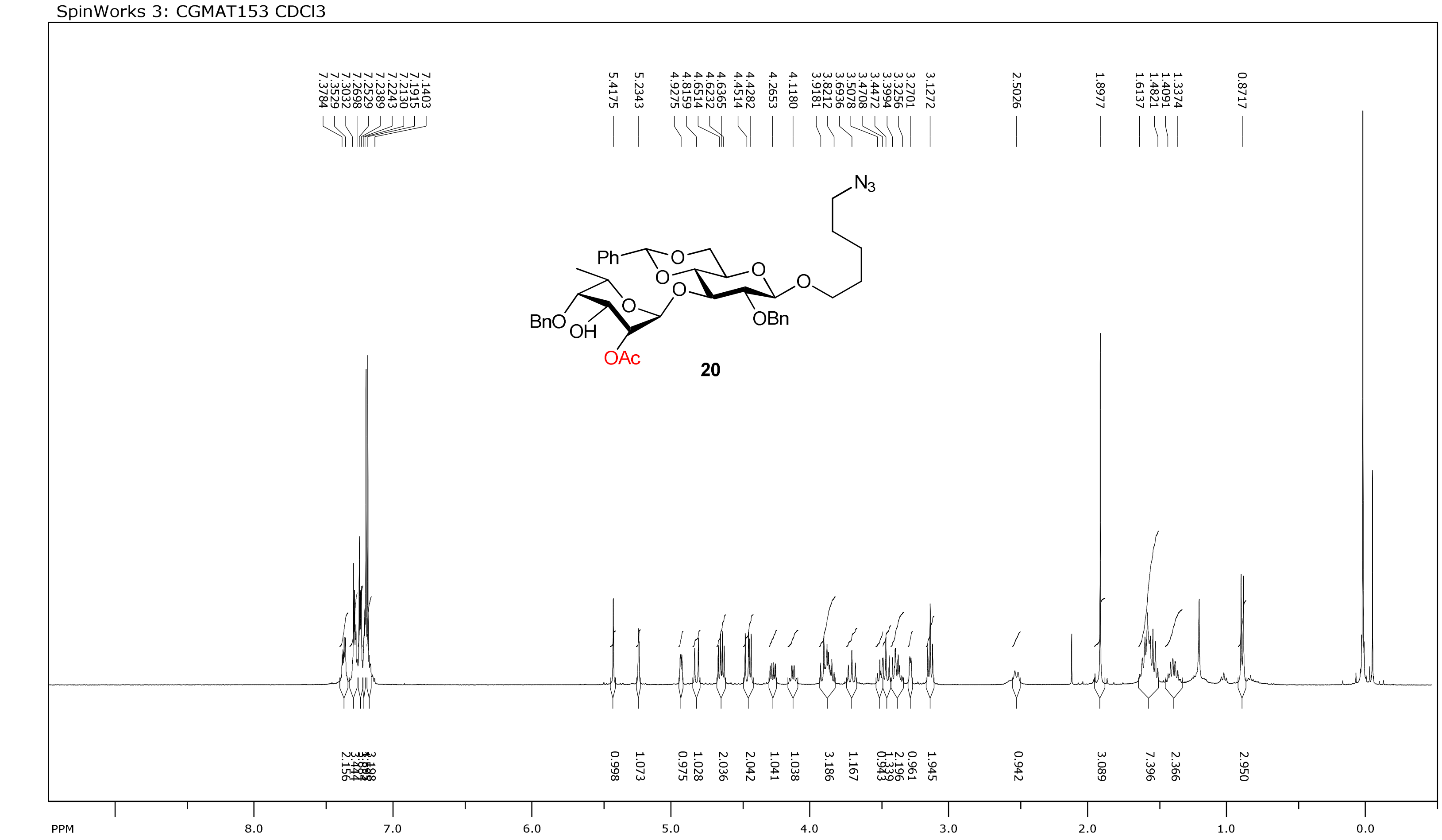

file: ...eau dossier\RMN LPS\CGMAT153\1\fid expt: <zg30>
transmitter freq.: 400.132001 MHz
time domain size: 32768 points
width: 4807.69 Hz = 12.0153 ppm = 0.146719 Hz/pt
number of scans: 32

freq. of 0 ppm: 400.130038 MHz
processed size: 32768 complex points
LB: 0.300 GF: 0.0000
Hz/cm: 160.052 ppm/cm: 0.40000

Supplementary Figure 84 | ^{13}C NMR spectra ($CDCl_3$, 100 MHz) of compound 20.

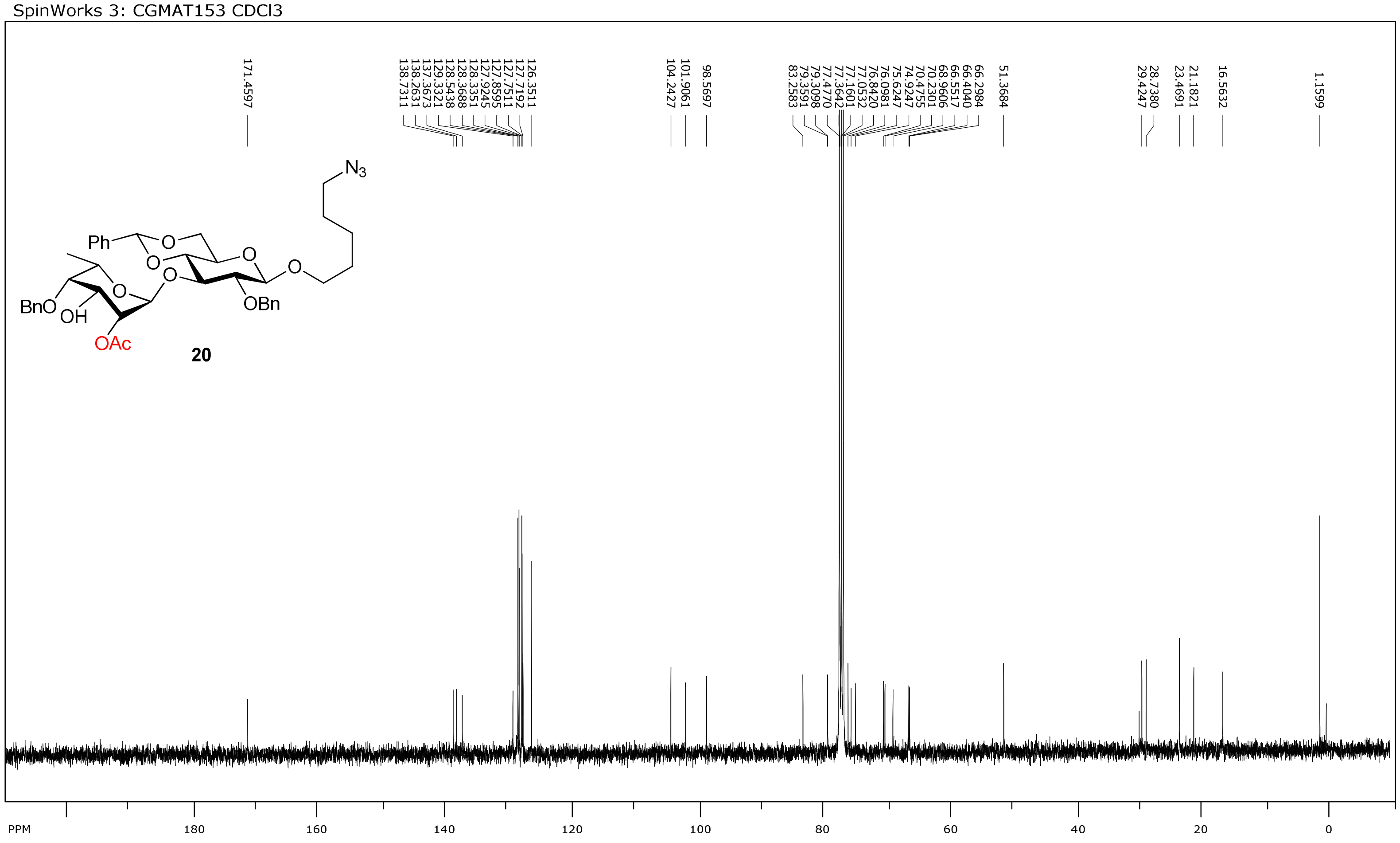

file: ...eau dossier\RMN LPS\CGMAT153\3\fid expt: <zgpg30>
transmitter freq.: 100.622830 MHz
time domain size: 65536 points
width: 24038.46 Hz = 238.8967 ppm = 0.366798 Hz/pt
number of scans: 1024

freq. of 0 ppm: 100.612755 MHz
processed size: 32768 complex points
LB: 1.000 GF: 0.0000
Hz/cm: 885.392 ppm/cm: 8.79912

Supplementary Figure 85 │ ^{1}H NMR spectra ($CDCl_3$, 400 MHz) of compound 21.

SpinWorks 3: CGMAT62A CDCl3

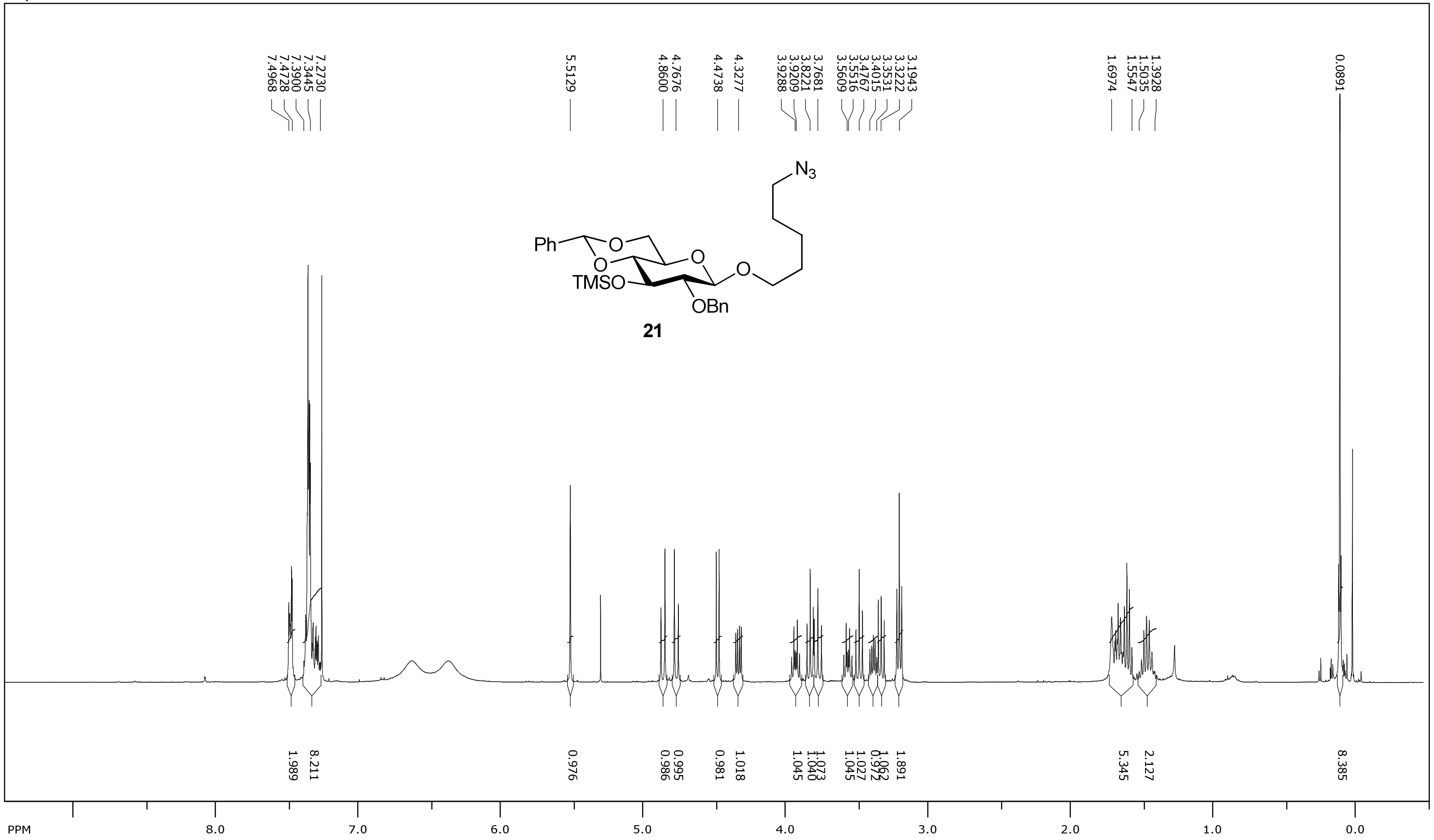

file: ...eau dossier\RMN LPS\CGMAT62A\1\fid expt: <zg30>
transmitter freq.: 400.132001 MHz
time domain size: 32768 points
width: 4807.69 Hz = 12.0153 ppm = 0.146719 Hz/pt
number of scans: 64

freq. of 0 ppm: 400.130008 MHz
processed size: 32768 complex points
LB: 0.300 GF: 0.0000
Hz/cm: 160.052 ppm/cm: 0.40000

Supplementary Figure 86 | ^{13}C NMR spectra ($CDCl_3$, 100 MHz) of compound 21.

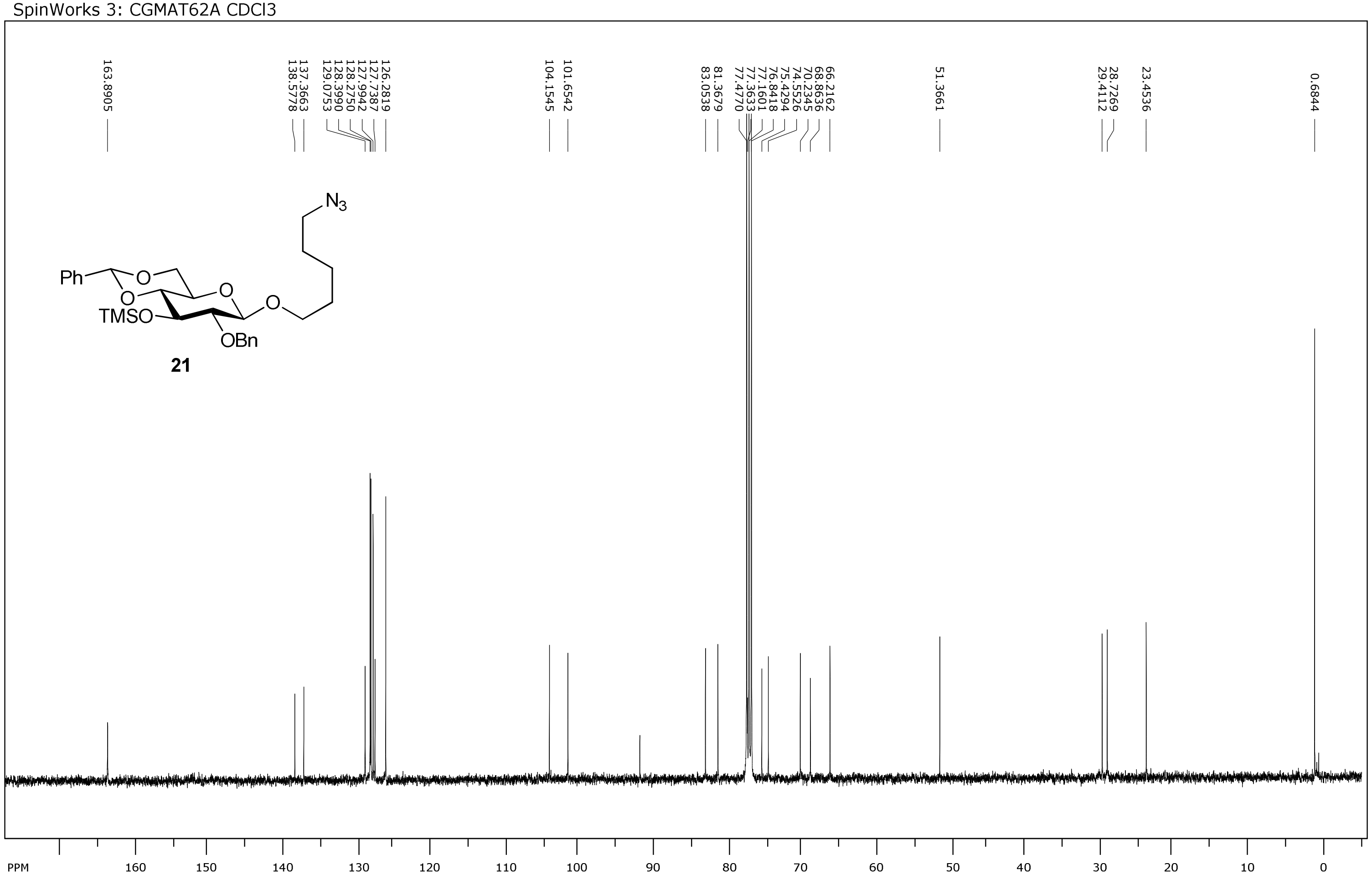

21

file: ...eau dossier\RMN LPS\CGMAT62A\3\fid expt: <zgpg30>
transmitter freq.: 100.622830 MHz
time domain size: 65536 points
width: 24038.46 Hz = 238.8967 ppm = 0.366798 Hz/pt
number of scans: 1024

freq. of 0 ppm: 100.612757 MHz
processed size: 32768 complex points
LB: 1.000 GF: 0.0000
Hz/cm: 738.455 ppm/cm: 7.33884

Supplementary Figure 87 │ ^{1}H NMR spectra ($CDCl_3$, 400 MHz) of compound 22.

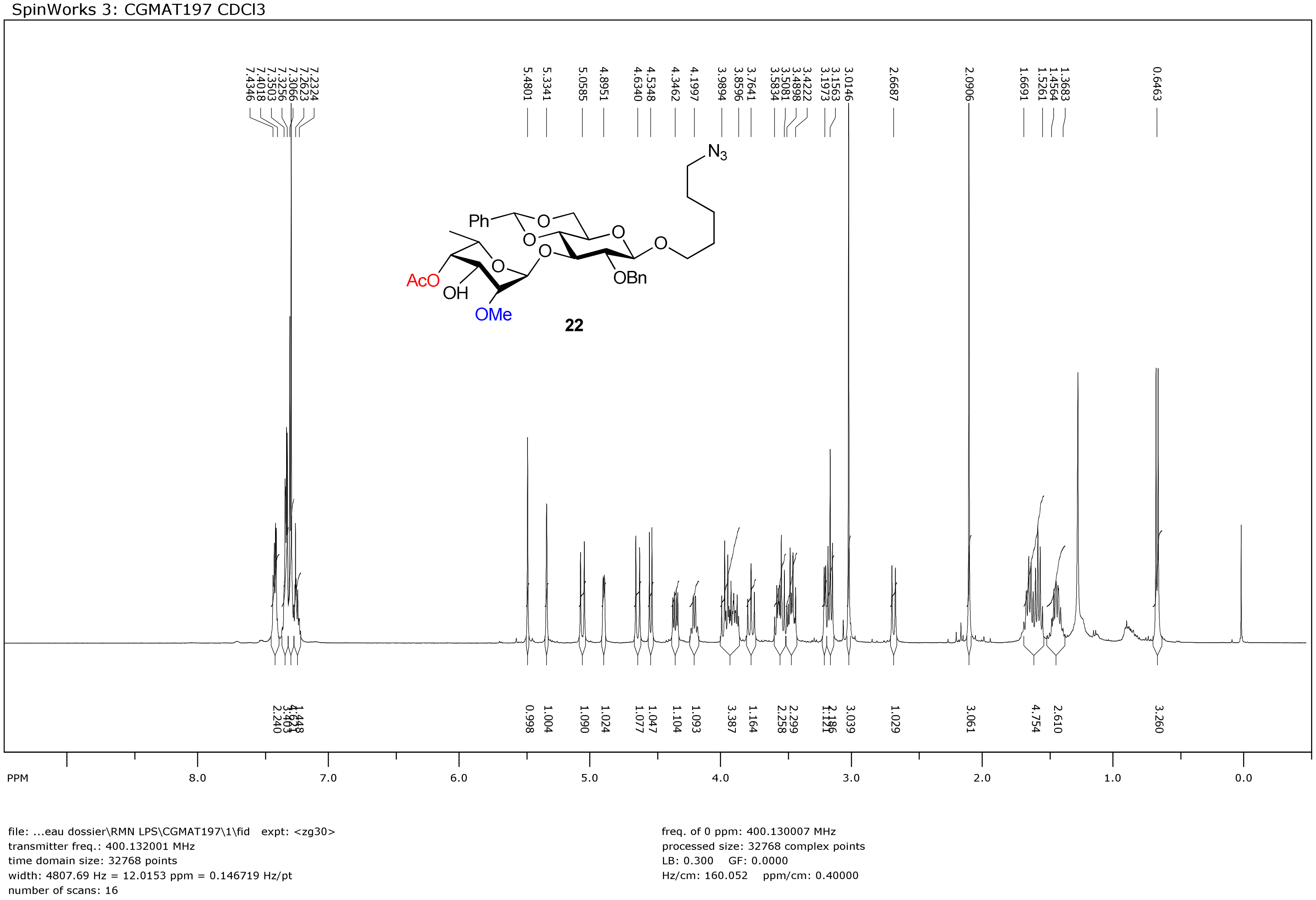

Supplementary Figure 88 │ ^{13}C NMR spectra ($CDCl_3$, 100 MHz) of compound 22.

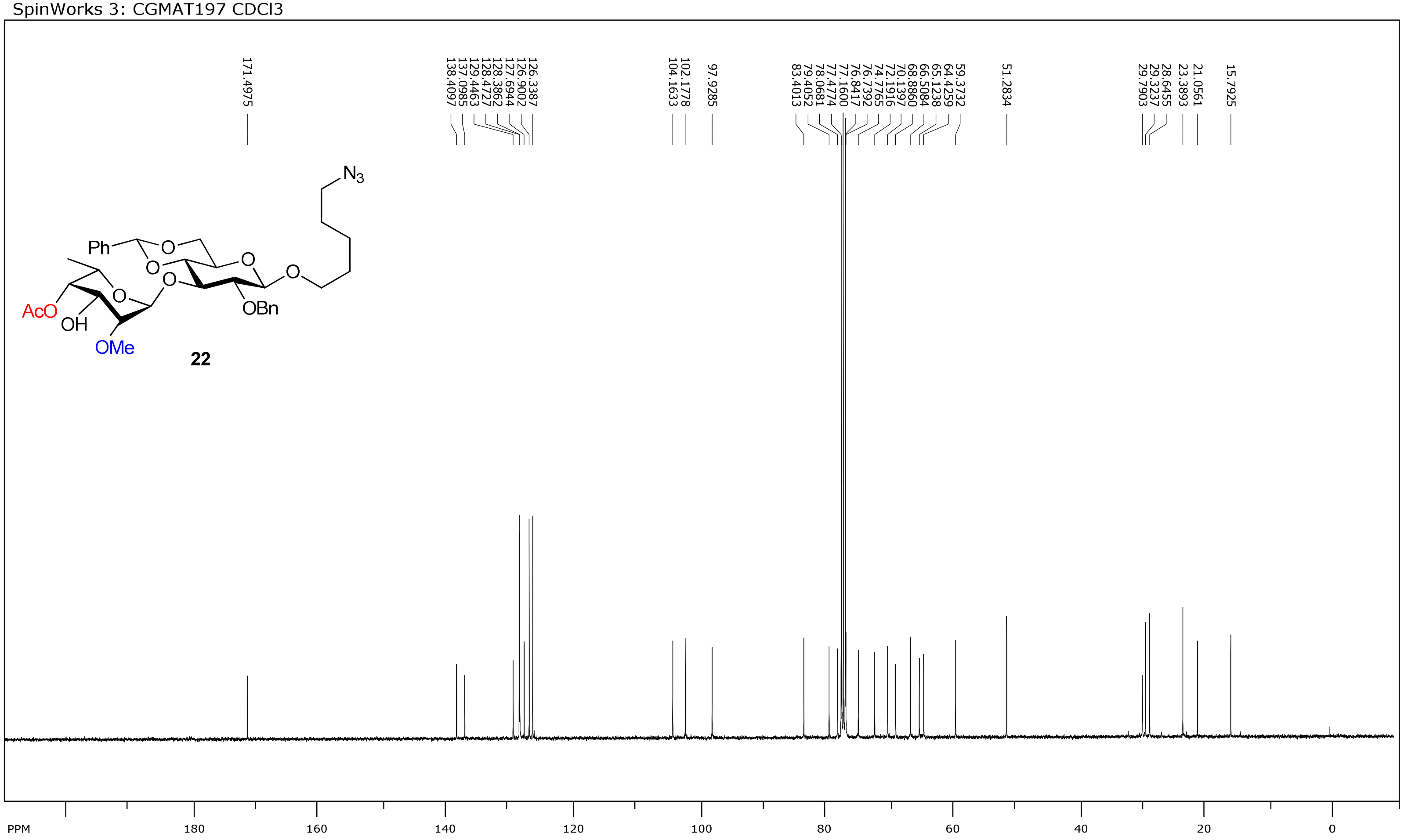

file: ...eau dossier\RMN LPS\CGMAT197\4\fid expt: <zgpg30>
transmitter freq.: 100.622830 MHz
time domain size: 65536 points
width: 24038.46 Hz = 238.8967 ppm = 0.366798 Hz/pt
number of scans: 1024

freq. of 0 ppm: 100.612759 MHz
processed size: 32768 complex points
LB: 1.000 GF: 0.0000
Hz/cm: 885.392 ppm/cm: 8.79912

Supplementary Figure 89 | ^{1}H NMR spectra ($CDCl_3$, 400 MHz) of compound 23.

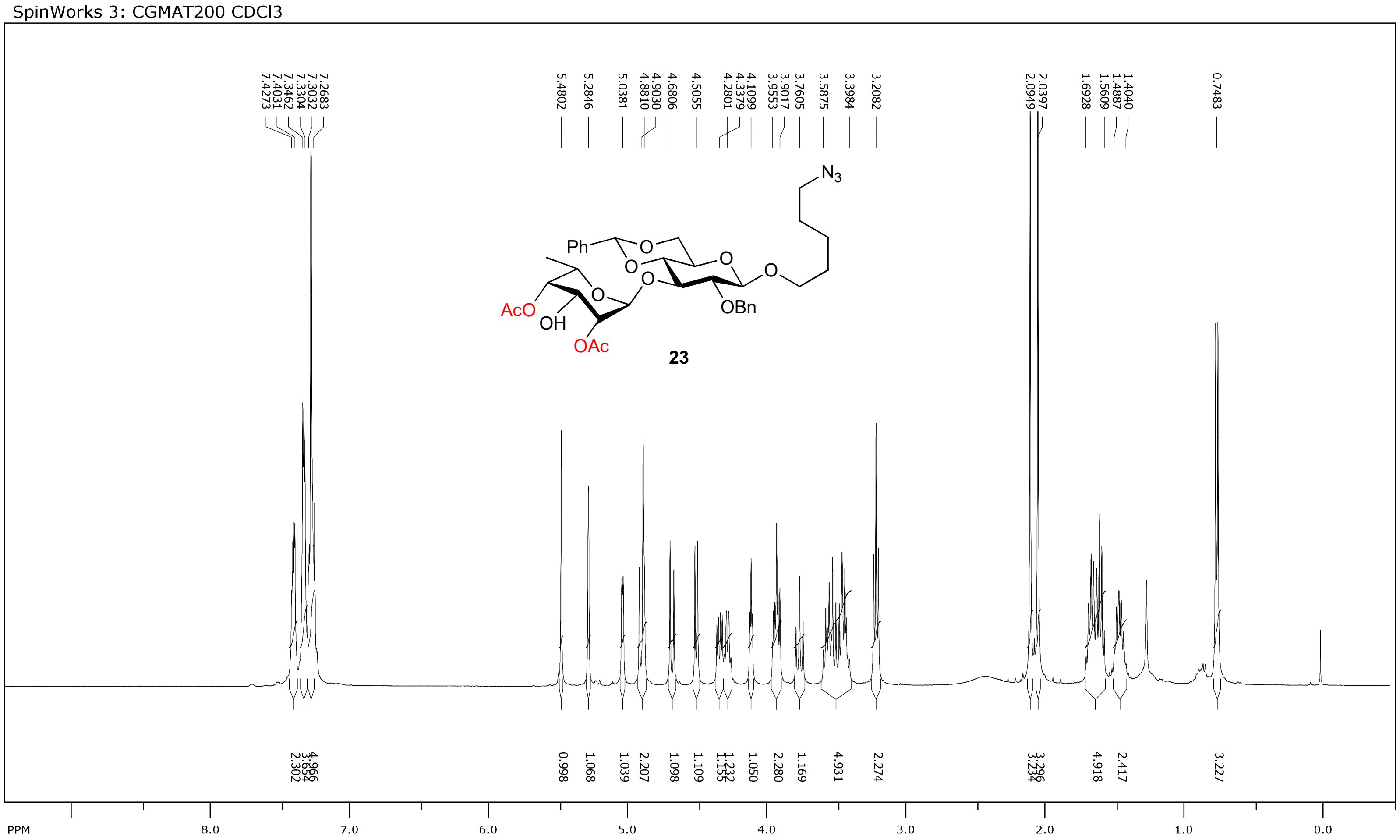

file: ...eau dossier\RMN LPS\CGMAT200\1\fid expt: <zg30>
transmitter freq.: 400.132001 MHz
time domain size: 32768 points
width: 4807.69 Hz = 12.0153 ppm = 0.146719 Hz/pt
number of scans: 32

freq. of 0 ppm: 400.130009 MHz
processed size: 32768 complex points
LB: 0.300 GF: 0.0000
Hz/cm: 160.052 ppm/cm: 0.40000

Supplementary Figure 90 | ^{13}C NMR spectra ($CDCl_3$, 100 MHz) of compound 23.

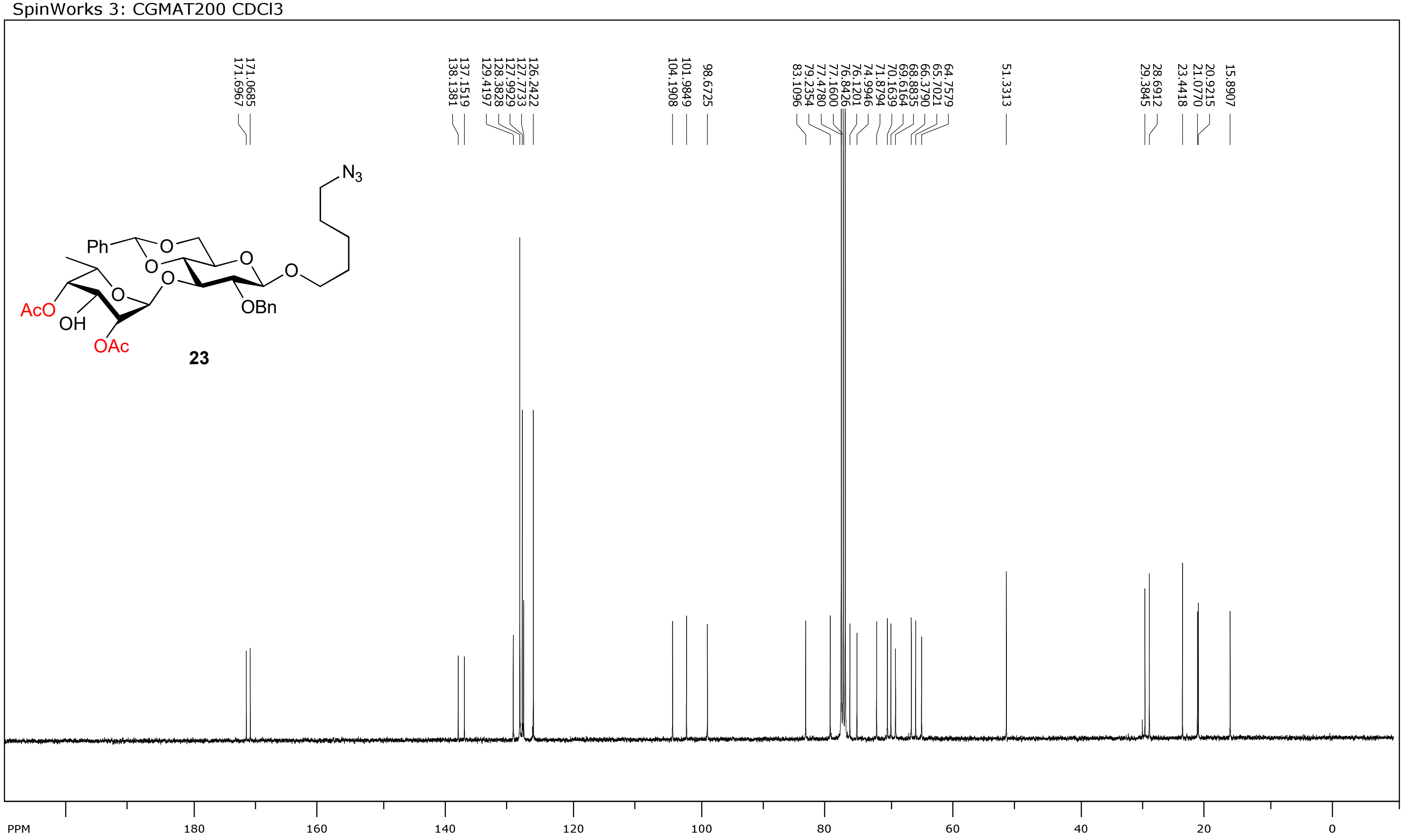

file: ...eau dossier\RMN LPS\CGMAT200\3\fid expt: <zgpg30>
transmitter freq.: 100.622830 MHz
time domain size: 65536 points
width: 24038.46 Hz = 238.8967 ppm = 0.366798 Hz/pt
number of scans: 1024

freq. of 0 ppm: 100.612759 MHz
processed size: 32768 complex points
LB: 1.000 GF: 0.0000
Hz/cm: 885.392 ppm/cm: 8.79912

Supplementary Figure 91 │ ^{1}H NMR spectra ($CDCl_3$, 400 MHz) of compound 25.

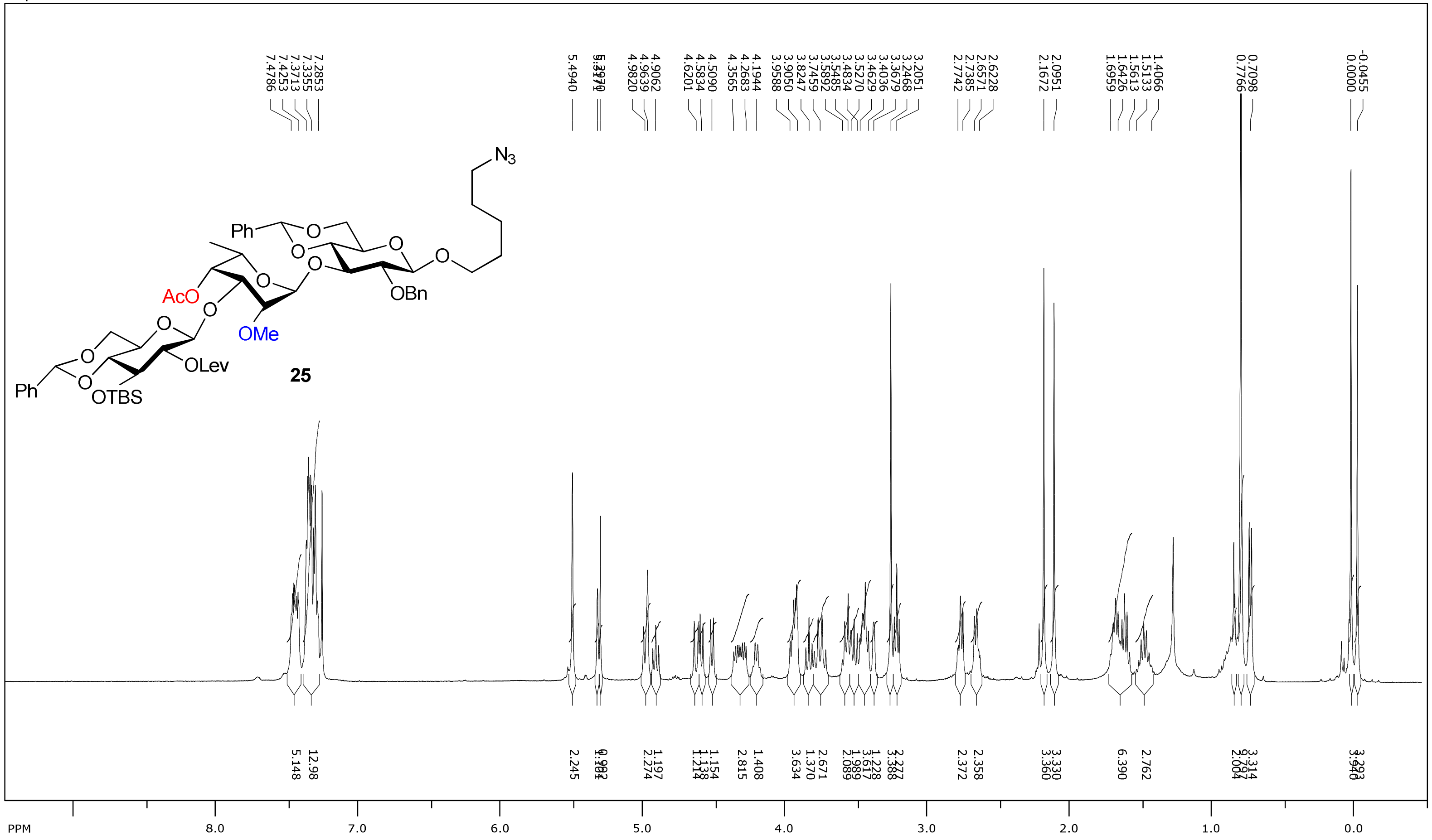

file: ...eau dossier\RMN LPS\CGMAT199\1\fid expt: <zg30>
transmitter freq.: 400.132001 MHz
time domain size: 32768 points
width: 4807.69 Hz = 12.0153 ppm = 0.146719 Hz/pt
number of scans: 16

freq. of 0 ppm: 400.130009 MHz
processed size: 32768 complex points
LB: 0.300 GF: 0.0000
Hz/cm: 160.052 ppm/cm: 0.40000

Supplementary Figure 92 | ^{13}C NMR spectra ($CDCl_3$, 100 MHz) of compound 25.

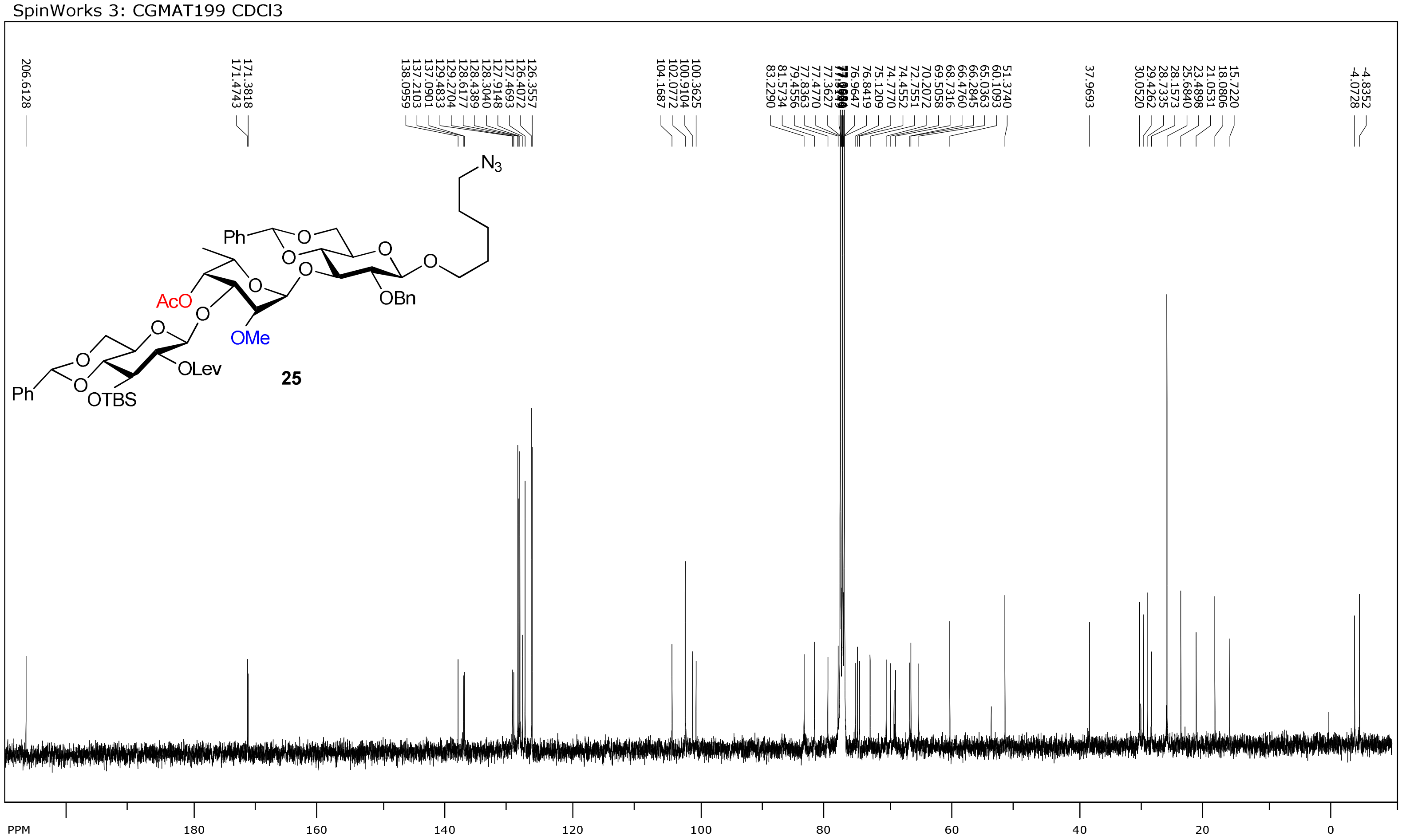

file: ...eau dossier\RMN LPS\CGMAT199\4\fid expt: <zgpg30>
transmitter freq.: 100.622830 MHz
time domain size: 65536 points
width: 24038.46 Hz = 238.8967 ppm = 0.366798 Hz/pt
number of scans: 1024

freq. of 0 ppm: 100.612755 MHz
processed size: 32768 complex points
LB: 1.000 GF: 0.0000
Hz/cm: 885.392 ppm/cm: 8.79912

Supplementary Figure 93 │ ^{1}H NMR spectra ($CDCl_3$, 400 MHz) of compound 26.

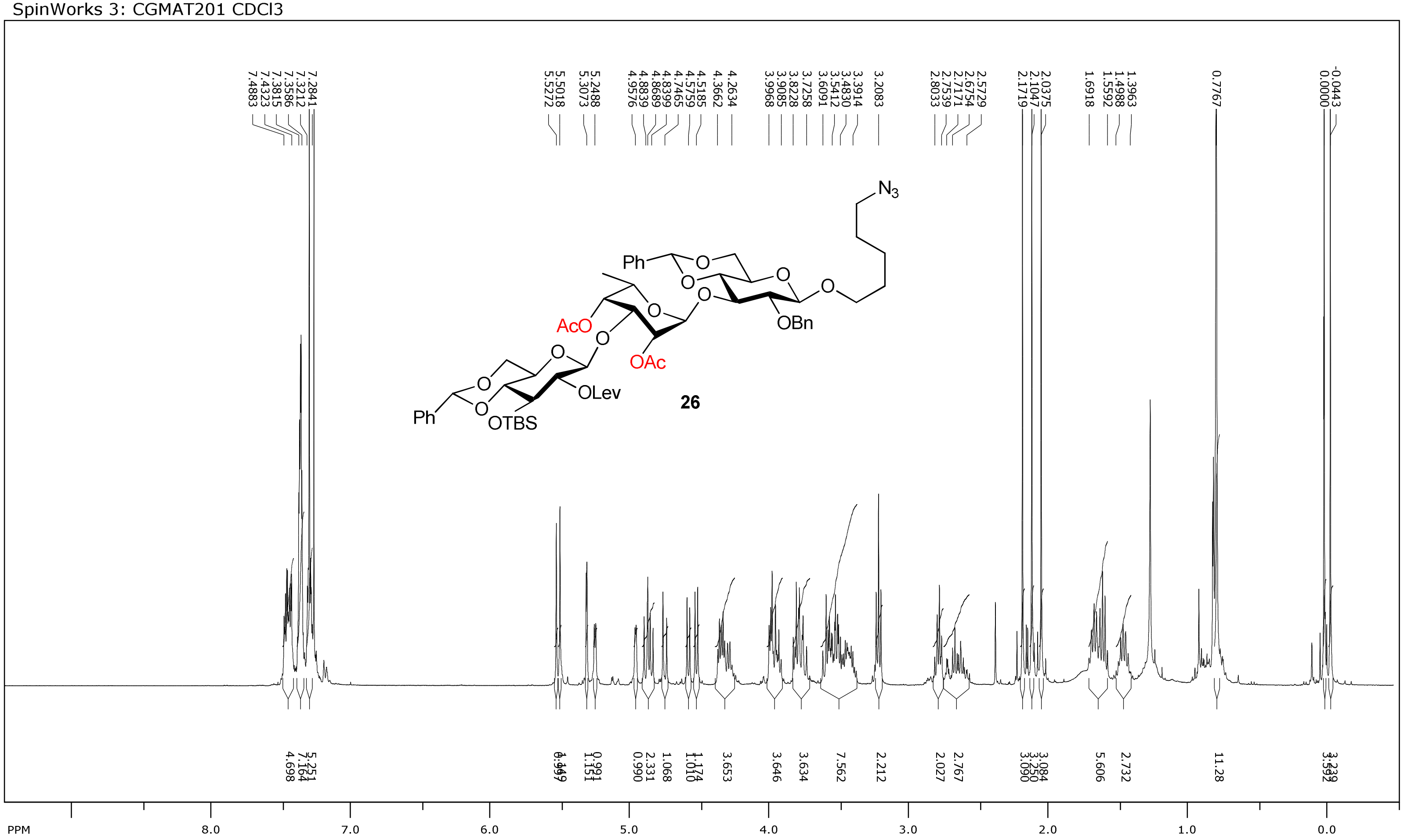

file: ...eau dossier\RMN LPS\CGMAT201\1\fid expt: <zg30>
transmitter freq.: 400.132001 MHz
time domain size: 32768 points
width: 4807.69 Hz = 12.0153 ppm = 0.146719 Hz/pt
number of scans: 32

freq. of 0 ppm: 400.130006 MHz
processed size: 32768 complex points
LB: 0.300 GF: 0.0000
Hz/cm: 160.052 ppm/cm: 0.40000

Supplementary Figure 94 | ^{13}C NMR spectra ($CDCl_3$, 100 MHz) of compound 26.

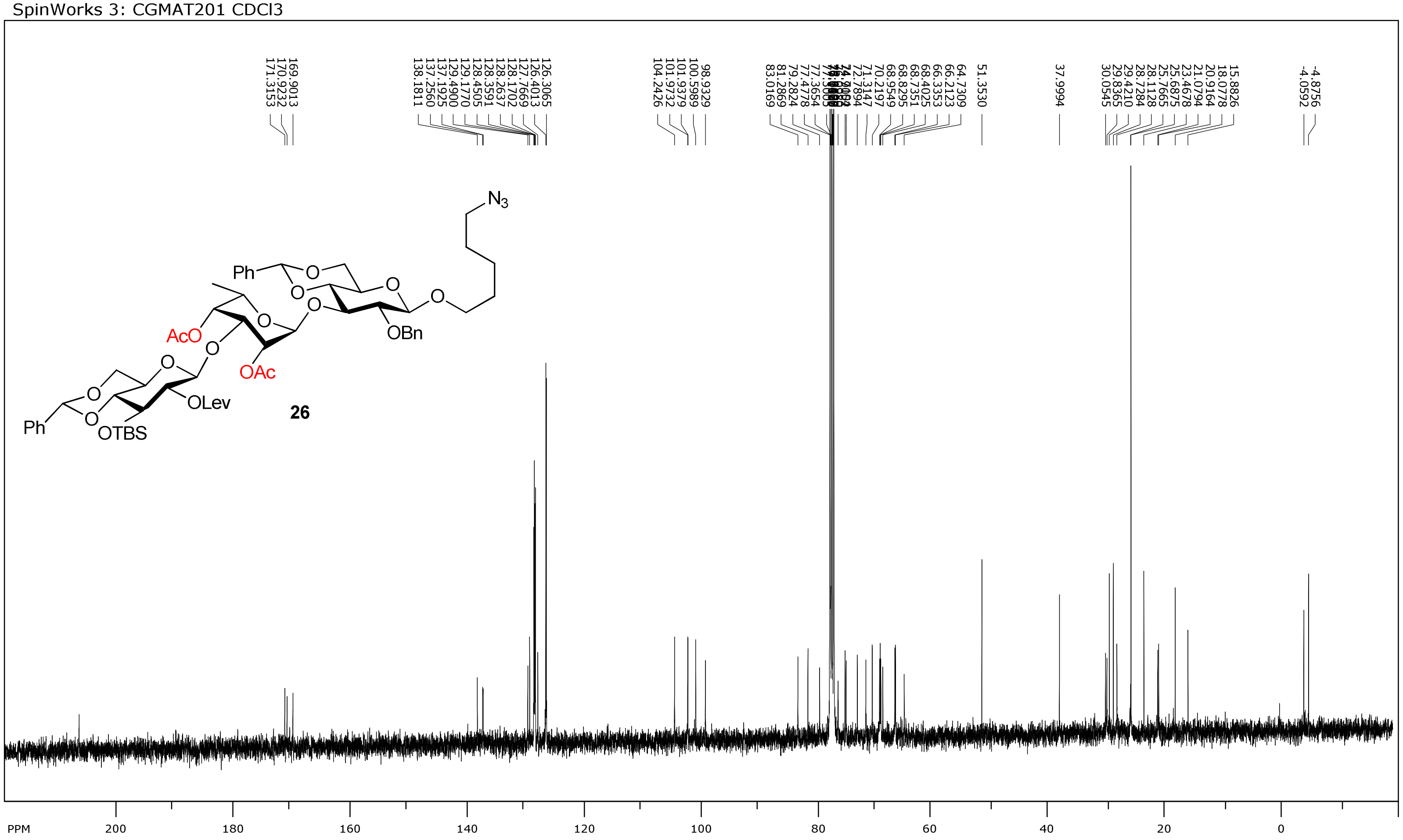

file: ...eau dossier\RMN LPS\CGMAT201\3\fid expt: <zgpg30>
transmitter freq.: 100.622830 MHz
time domain size: 65536 points
width: 24038.46 Hz = 238.8967 ppm = 0.366798 Hz/pt
number of scans: 1024

freq. of 0 ppm: 100.612756 MHz
processed size: 32768 complex points
LB: 1.000 GF: 0.0000
Hz/cm: 961.538 ppm/cm: 9.55587

Supplementary Figure 95 │ ^{1}H NMR spectra ($CDCl_3$, 400 MHz) of compound 27.

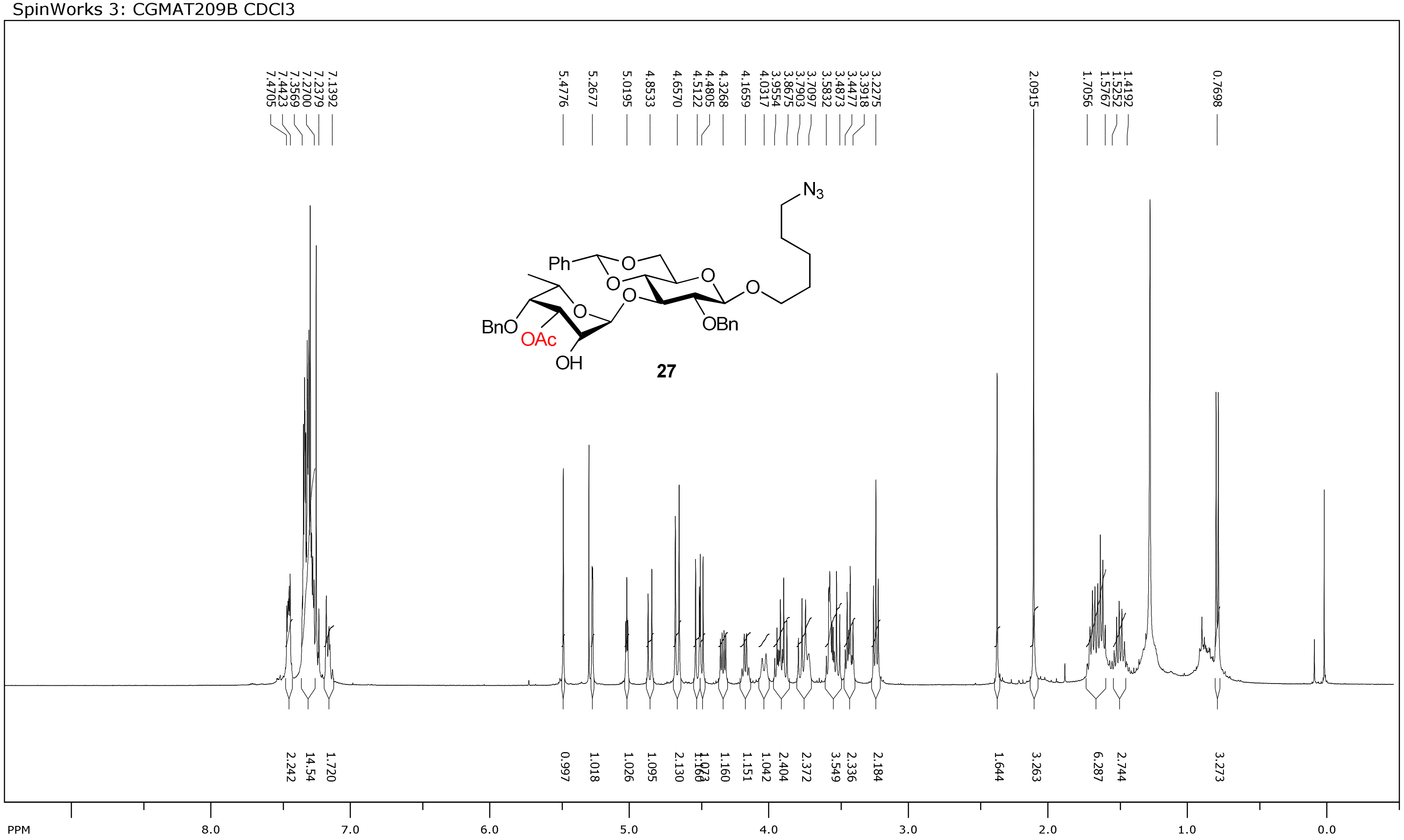

file: ...au dossier\RMN LPS\CGMAT209B\1\fid expt: <zg30>
transmitter freq.: 400.132001 MHz
time domain size: 32768 points
width: 4807.69 Hz = 12.0153 ppm = 0.146719 Hz/pt
number of scans: 32

freq. of 0 ppm: 400.130011 MHz
processed size: 32768 complex points
LB: 0.300 GF: 0.0000
Hz/cm: 160.052 ppm/cm: 0.40000

Supplementary Figure 96 | ^{13}C NMR spectra ($CDCl_3$, 100 MHz) of compound 27.

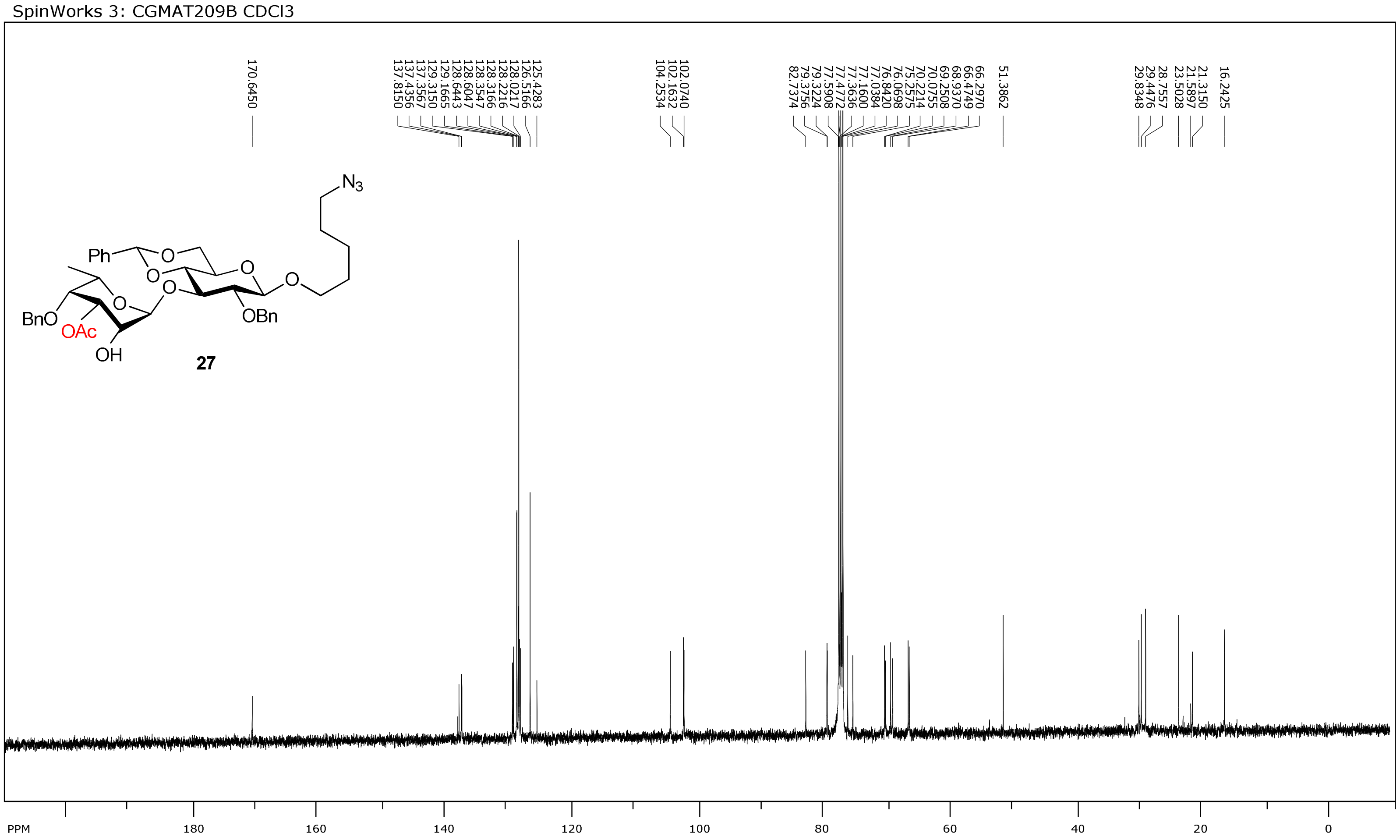

file: ...au dossier\RMN LPS\CGMAT209B\4\fid expt: <zgpg30>
transmitter freq.: 100.622830 MHz
time domain size: 65536 points
width: 24038.46 Hz = 238.8967 ppm = 0.366798 Hz/pt
number of scans: 1024

freq. of 0 ppm: 100.612756 MHz
processed size: 32768 complex points
LB: 1.000 GF: 0.0000
Hz/cm: 885.392 ppm/cm: 8.79912

Supplementary Figure 97 | ^{1}H NMR spectra ($CDCl_3$, 400 MHz) of compound 28.

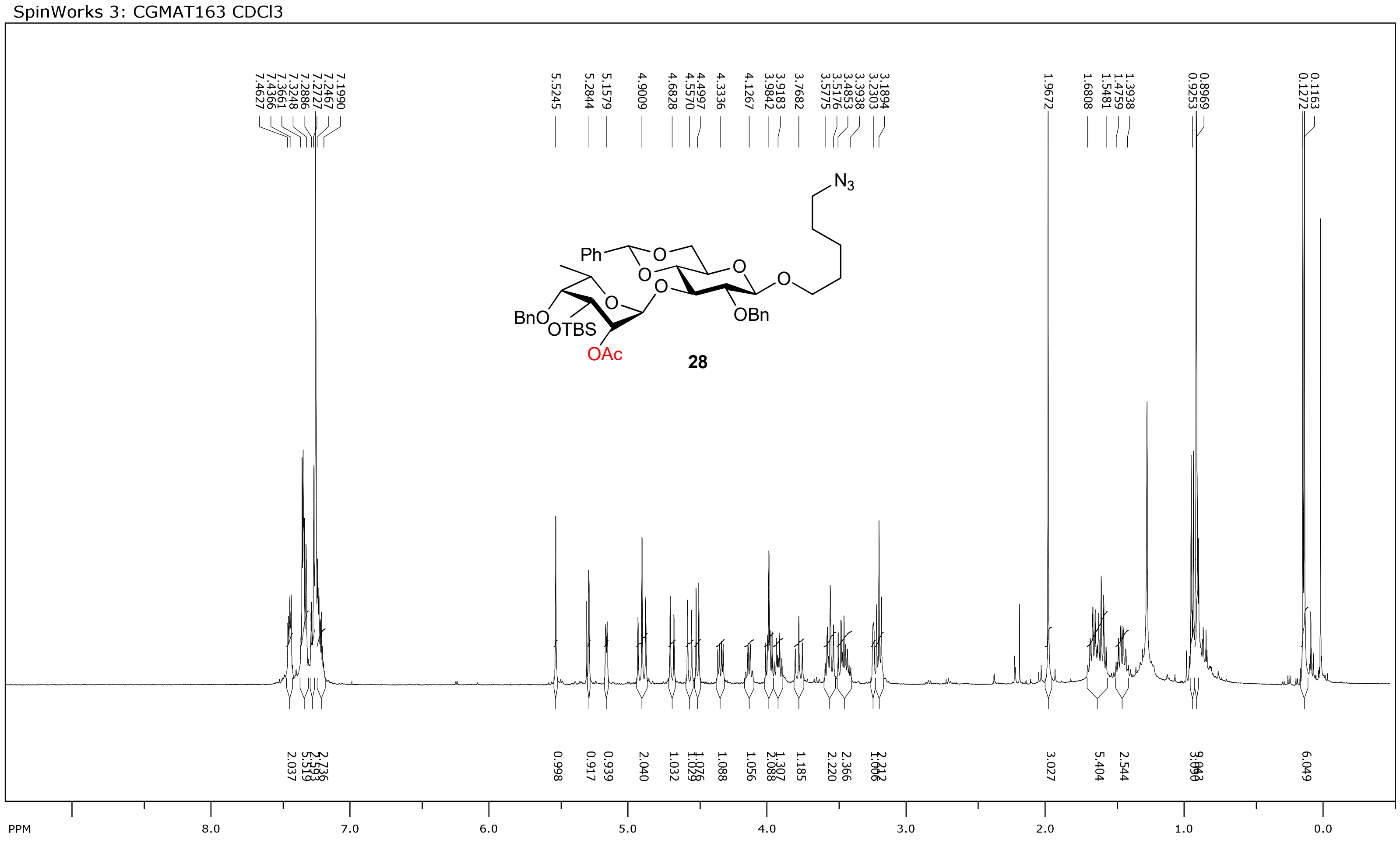

file: ...eau dossier\RMN LPS\CGMAT163\1\fid expt: <zg30>
transmitter freq.: 400.132001 MHz
time domain size: 32768 points
width: 4807.69 Hz = 12.0153 ppm = 0.146719 Hz/pt
number of scans: 32

freq. of 0 ppm: 400.130010 MHz
processed size: 32768 complex points
LB: 0.300 GF: 0.0000
Hz/cm: 160.052 ppm/cm: 0.40000

Supplementary Figure 98 | ^{13}C NMR spectra ($CDCl_3$, 100 MHz) of compound 28.

SpinWorks 3: CGMAT163 CDCl3

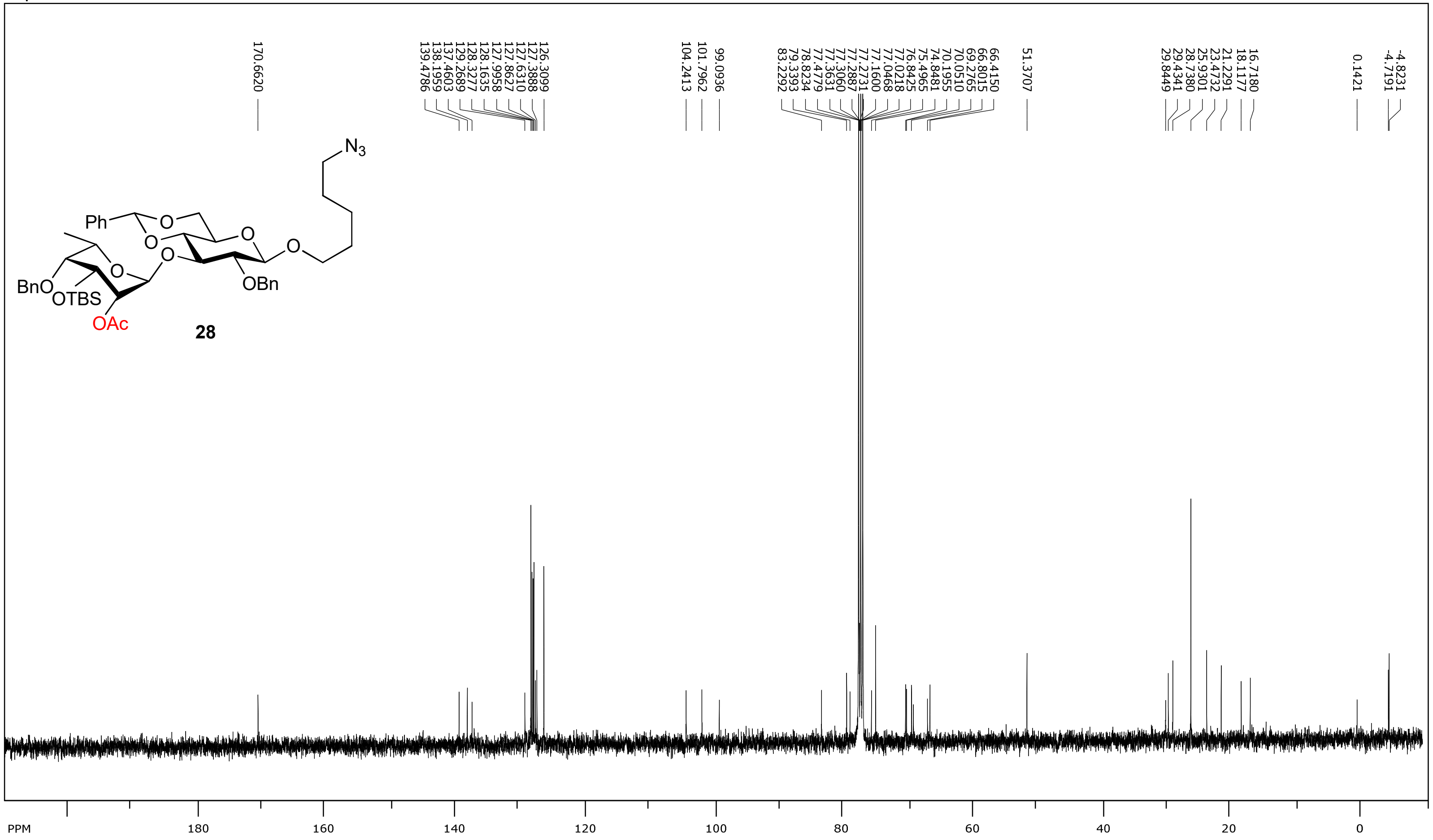

file: ...eau dossier\RMN LPS\CGMAT163\4\fid expt: <zgpg30>
transmitter freq.: 100.622830 MHz
time domain size: 65536 points
width: 24038.46 Hz = 238.8967 ppm = 0.366798 Hz/pt
number of scans: 512

freq. of 0 ppm: 100.612755 MHz
processed size: 32768 complex points
LB: 1.000 GF: 0.0000
Hz/cm: 885.392 ppm/cm: 8.79912

Supplementary Figure 99 │ ^{1}H NMR spectra ($CDCl_3$, 400 MHz) of compound 29.

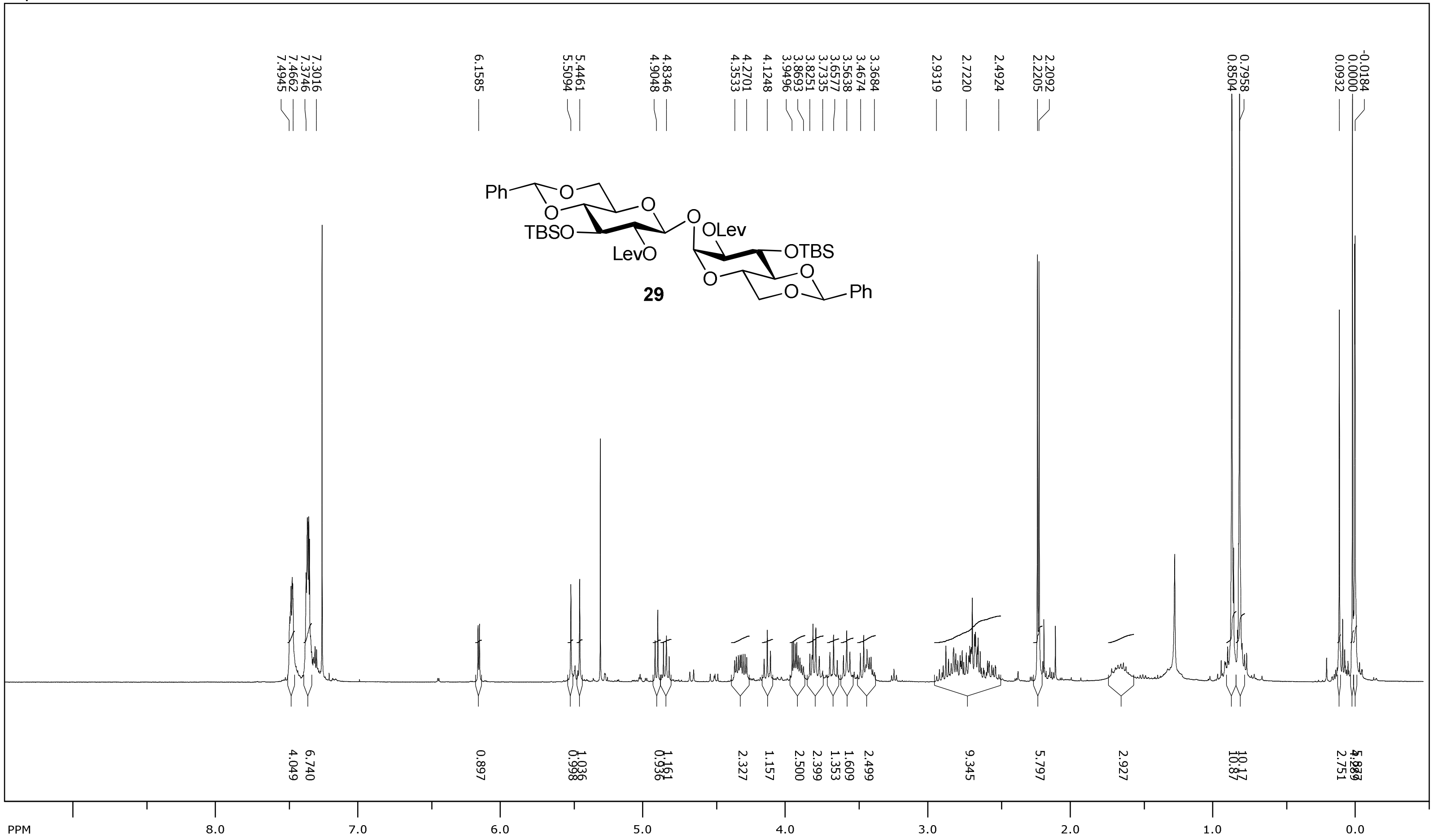

file: ...eau dossier\RMN LPS\CGMAT157\1\fid expt: <zg30>
transmitter freq.: 400.132001 MHz
time domain size: 32768 points
width: 4807.69 Hz = 12.0153 ppm = 0.146719 Hz/pt
number of scans: 64

freq. of 0 ppm: 400.130010 MHz
processed size: 32768 complex points
LB: 0.300 GF: 0.0000
Hz/cm: 160.052 ppm/cm: 0.40000

Supplementary Figure 100 │ ^{13}C NMR spectra ($CDCl_3$, 100 MHz) of compound 29.

SpinWorks 3: CGMAT157 CDCl3

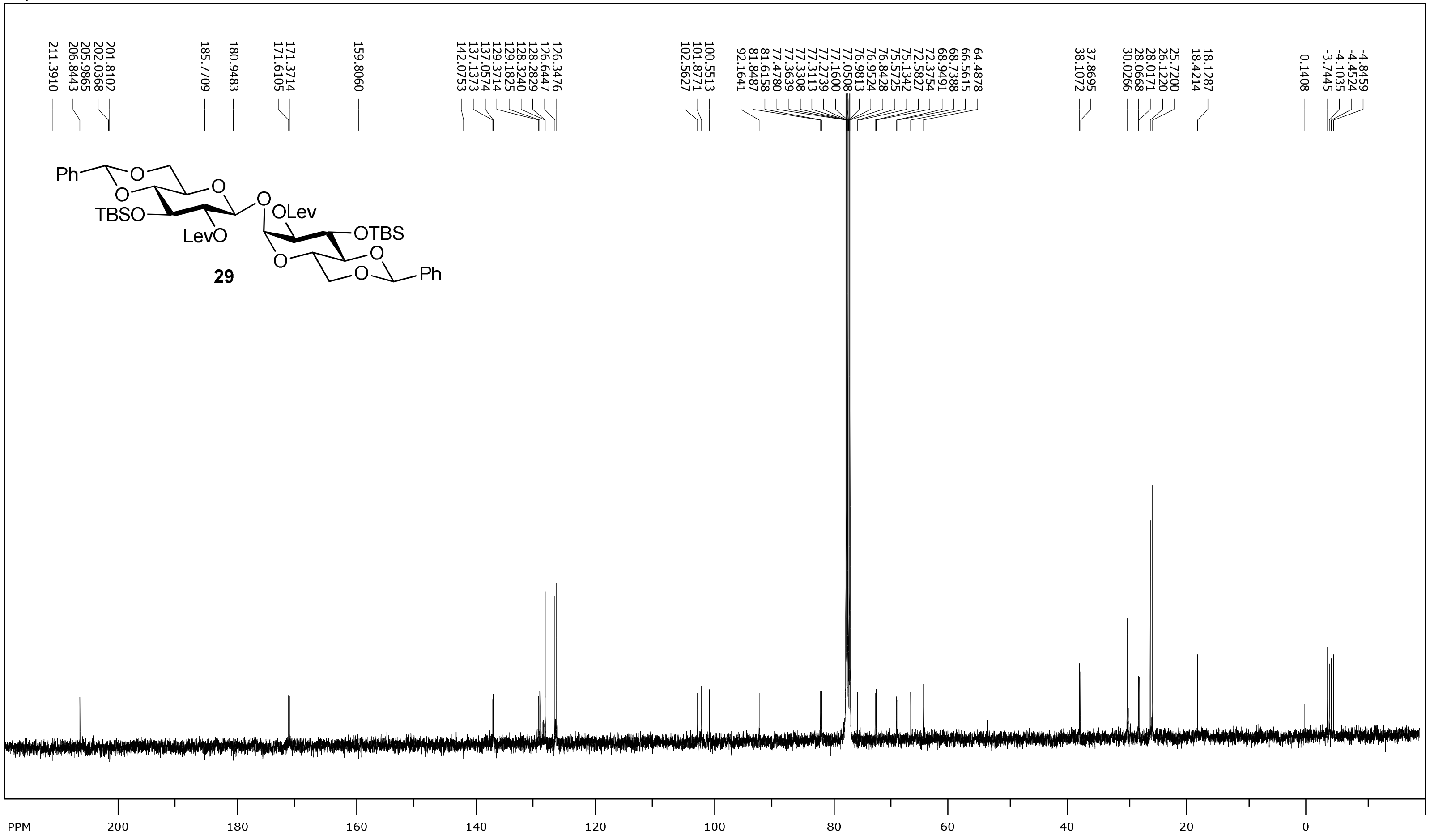

file: ...eau dossier\RMN LPS\CGMAT157\4\fid expt: <zgpg30>
transmitter freq.: 100.622830 MHz
time domain size: 65536 points
width: 24038.46 Hz = 238.8967 ppm = 0.366798 Hz/pt
number of scans: 1024

freq. of 0 ppm: 100.612755 MHz
processed size: 32768 complex points
LB: 1.000 GF: 0.0000
Hz/cm: 961.538 ppm/cm: 9.55587

Supplementary Figure 101 │ ^{1}H NMR spectra ($CDCl_3$, 400 MHz) of compound S28.

SpinWorks 3: CGMAT231 CDCl3

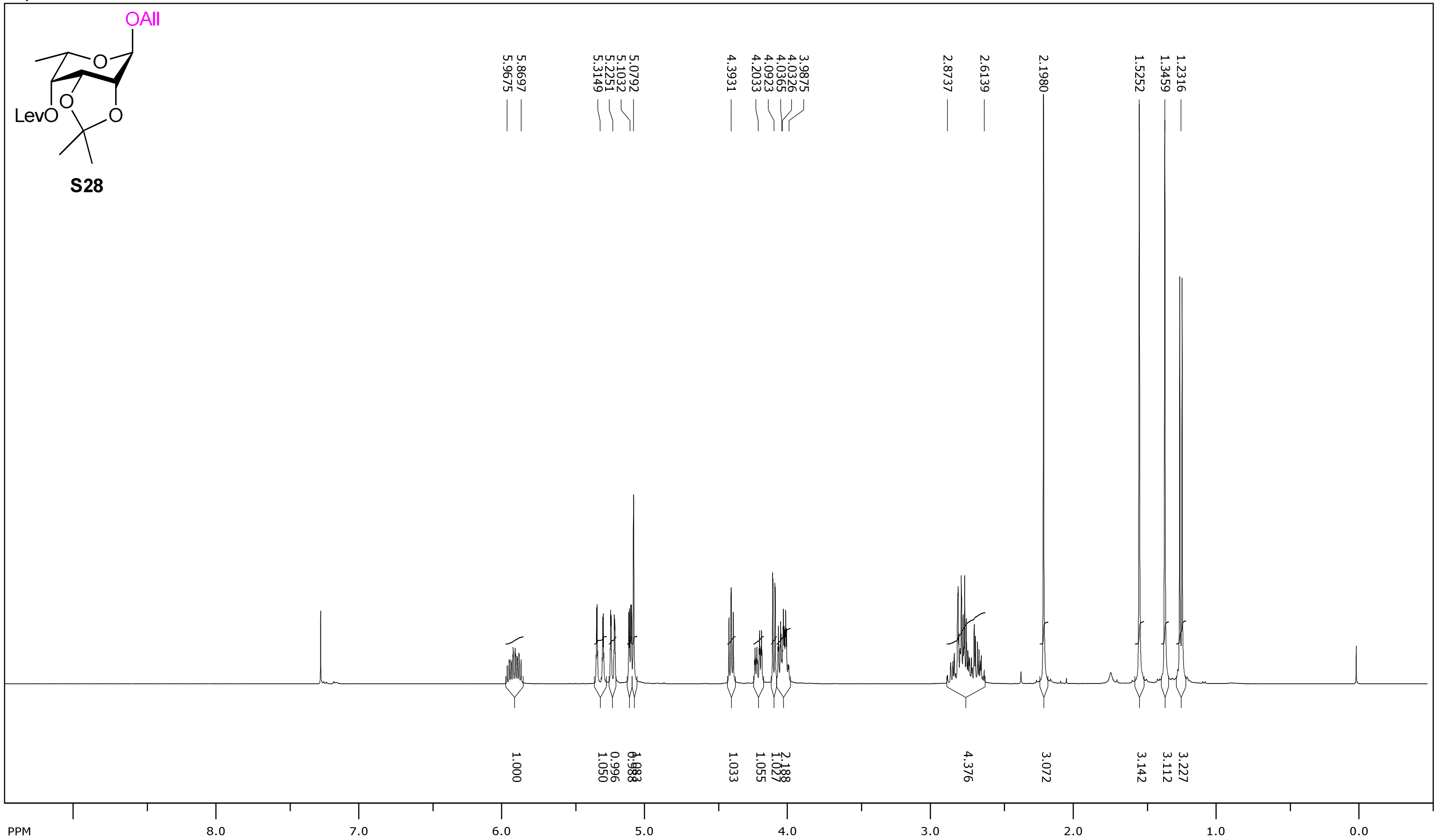

file: ...eau dossier\RMN LPS\CGMAT231\1\fid expt: <zg30>
transmitter freq.: 400.132001 MHz
time domain size: 32768 points
width: 4807.69 Hz = 12.0153 ppm = 0.146719 Hz/pt
number of scans: 16

freq. of 0 ppm: 400.130002 MHz
processed size: 32768 complex points
LB: 0.300 GF: 0.0000
Hz/cm: 160.052 ppm/cm: 0.40000

Supplementary Figure 102 | ^{13}C NMR spectra ($CDCl_3$, 100 MHz) of compound S28.

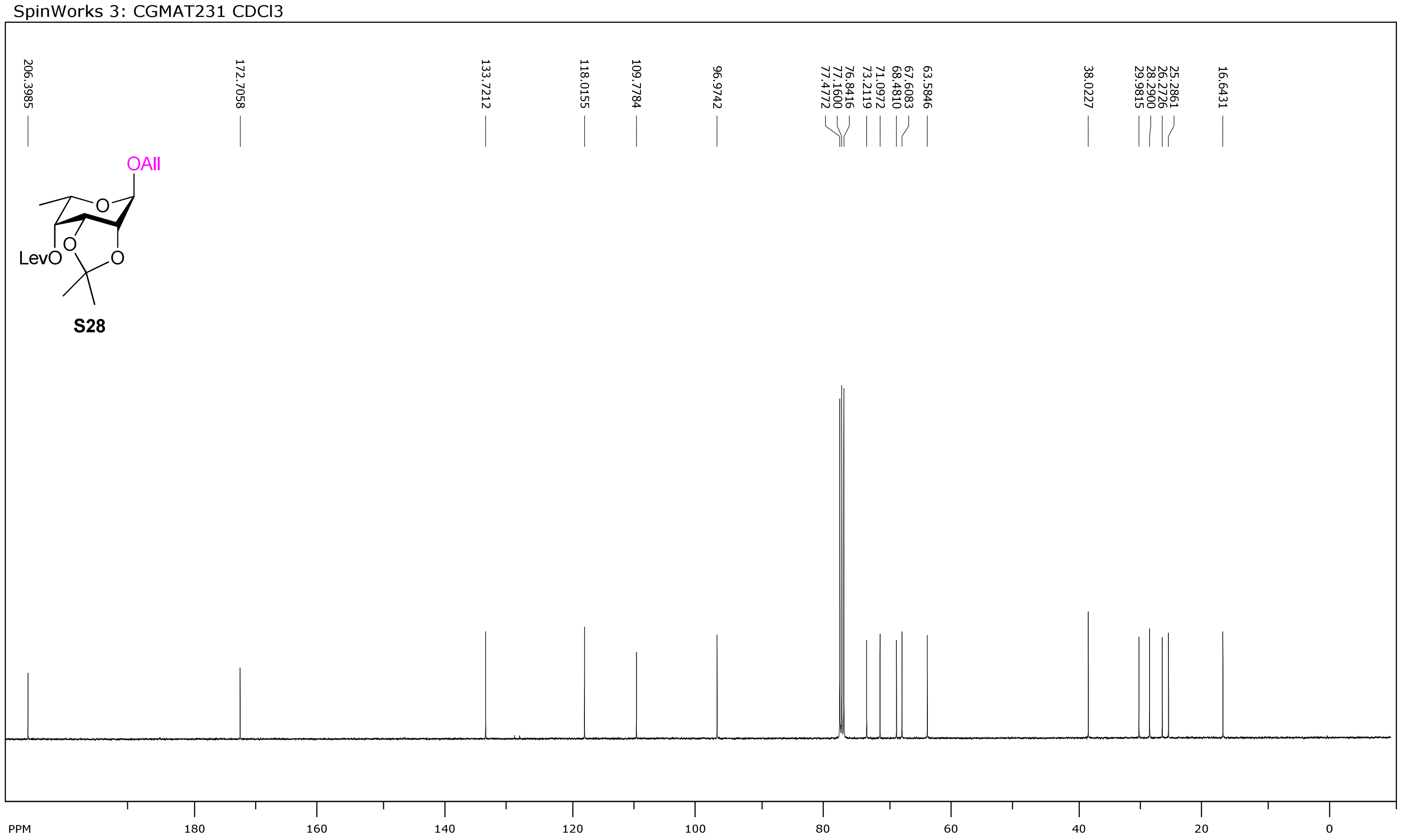

file: ...eau dossier\RMN LPS\CGMAT231\4\fid expt: <zgpg30>
transmitter freq.: 100.622830 MHz
time domain size: 65536 points
width: 24038.46 Hz = 238.8967 ppm = 0.366798 Hz/pt
number of scans: 1024

freq. of 0 ppm: 100.612757 MHz
processed size: 32768 complex points
LB: 1.000 GF: 0.0000
Hz/cm: 885.392 ppm/cm: 8.79912

Supplementary Figure 103 | ^{1}H NMR spectra ($CDCl_3$, 400 MHz) of compound S30.

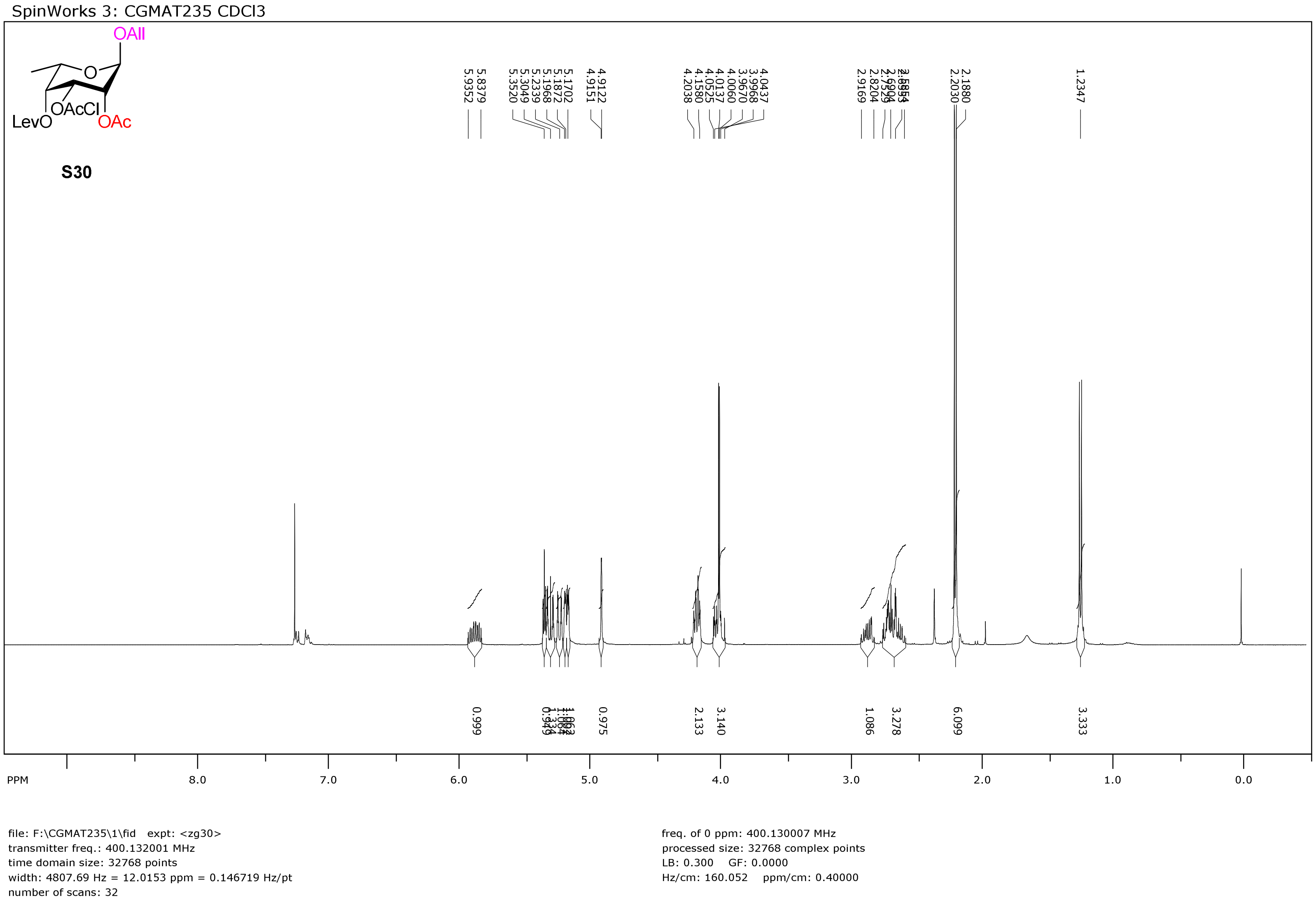

Supplementary Figure 104 | ^{13}C NMR spectra ($CDCl_3$, 100 MHz) of compound S30.

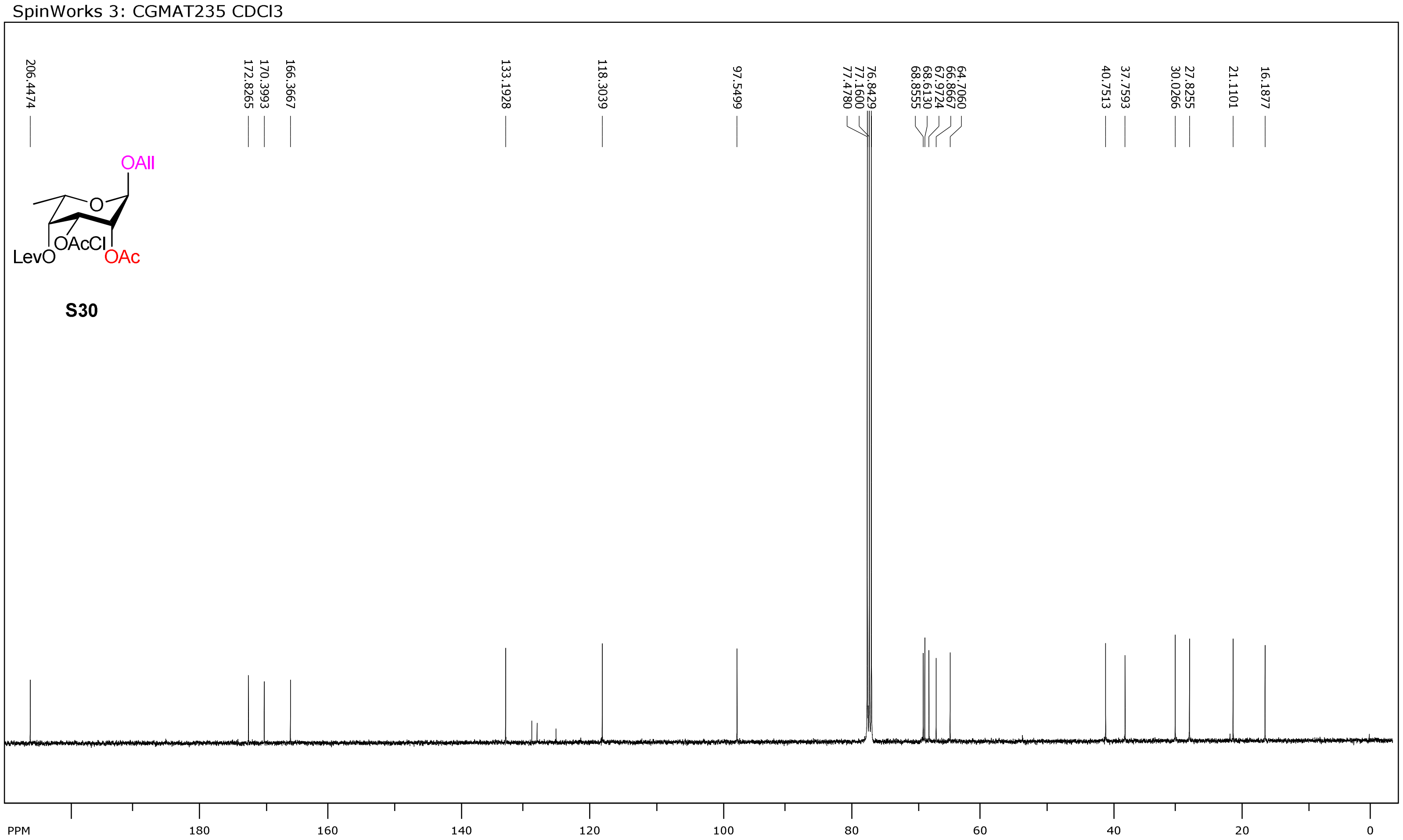

file: F:\CGMAT235\3\fid expt: <zgpg30>
transmitter freq.: 100.622830 MHz
time domain size: 65536 points
width: 24038.46 Hz = 238.8967 ppm = 0.366798 Hz/pt
number of scans: 1024

freq. of 0 ppm: 100.612755 MHz
processed size: 32768 complex points
LB: 1.000 GF: 0.0000
Hz/cm: 860.884 ppm/cm: 8.55555

Supplementary Figure 105 │ ^{1}H NMR spectra ($CDCl_3$, 400 MHz) of compound S32.

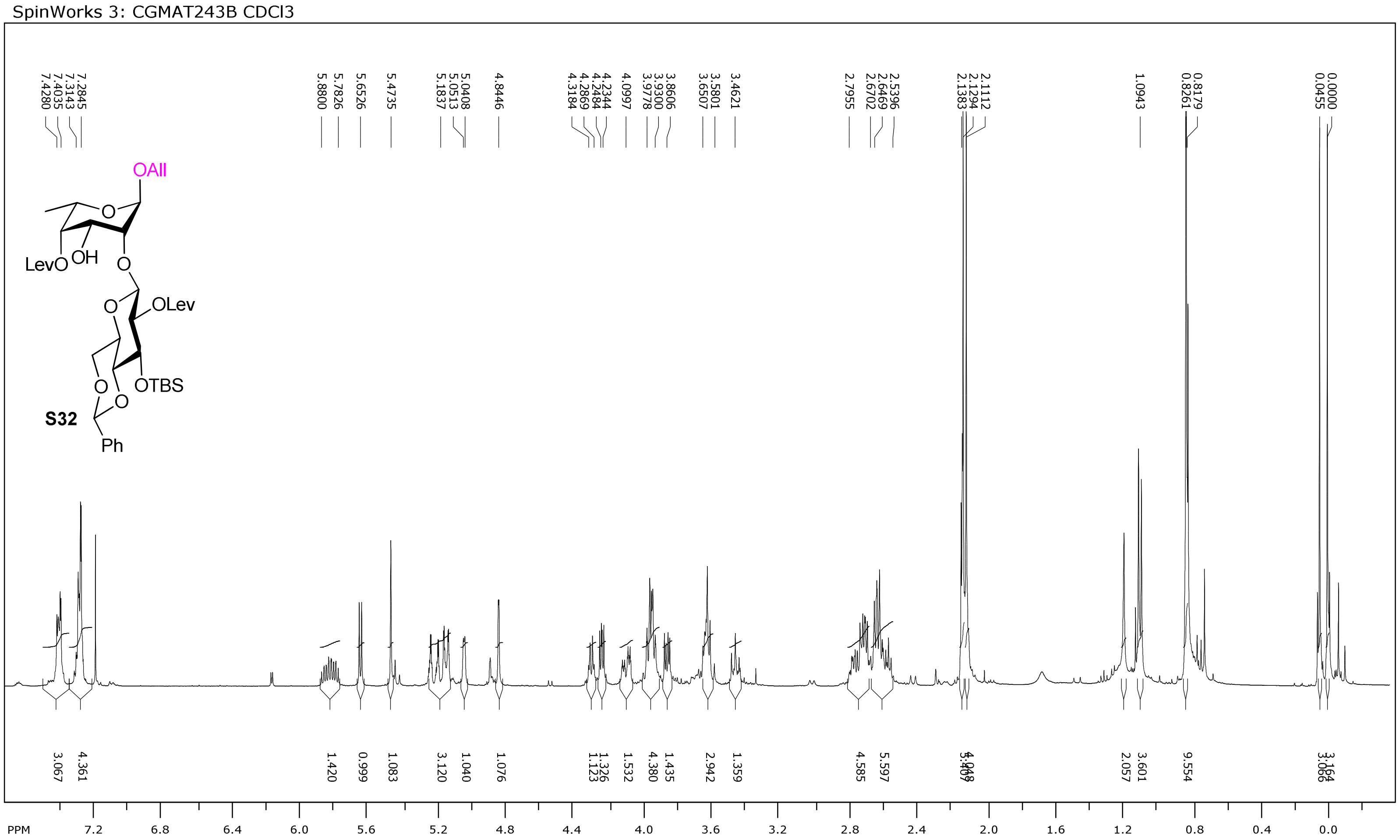

file: ...au dossier\RMN LPS\CGMAT243B\1\fid expt: <zg30>
transmitter freq.: 400.132001 MHz
time domain size: 32768 points
width: 4807.69 Hz = 12.0153 ppm = 0.146719 Hz/pt
number of scans: 32

freq. of 0 ppm: 400.130033 MHz
processed size: 32768 complex points
LB: 0.300 GF: 0.0000
Hz/cm: 129.624 ppm/cm: 0.32395

Supplementary Figure 106 | ^{13}C NMR spectra ($CDCl_3$, 100 MHz) of compound S32.

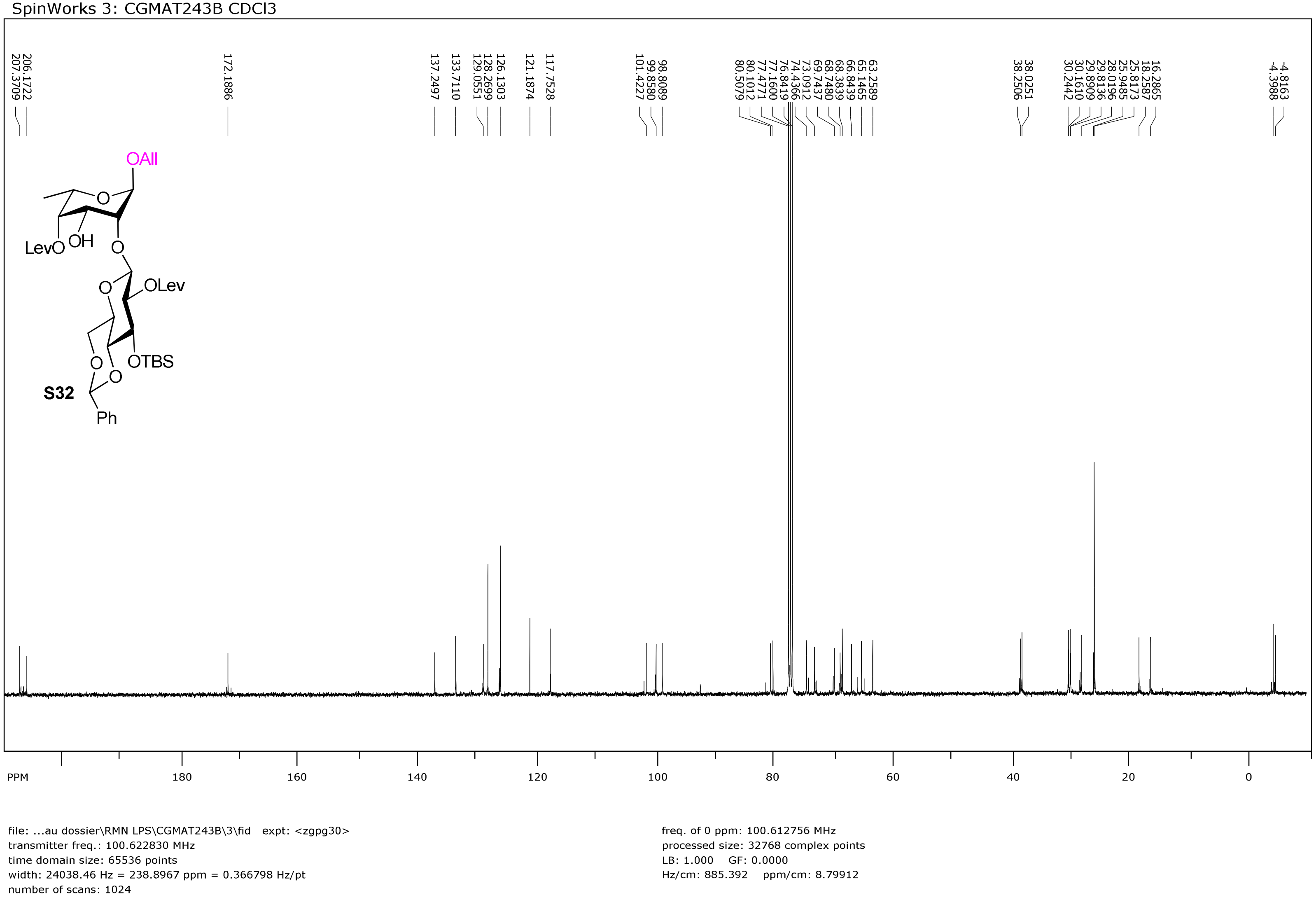

Supplementary Figure 107 │ ¹H NMR spectra ($CDCl_3$, 400 MHz) of compound S34.

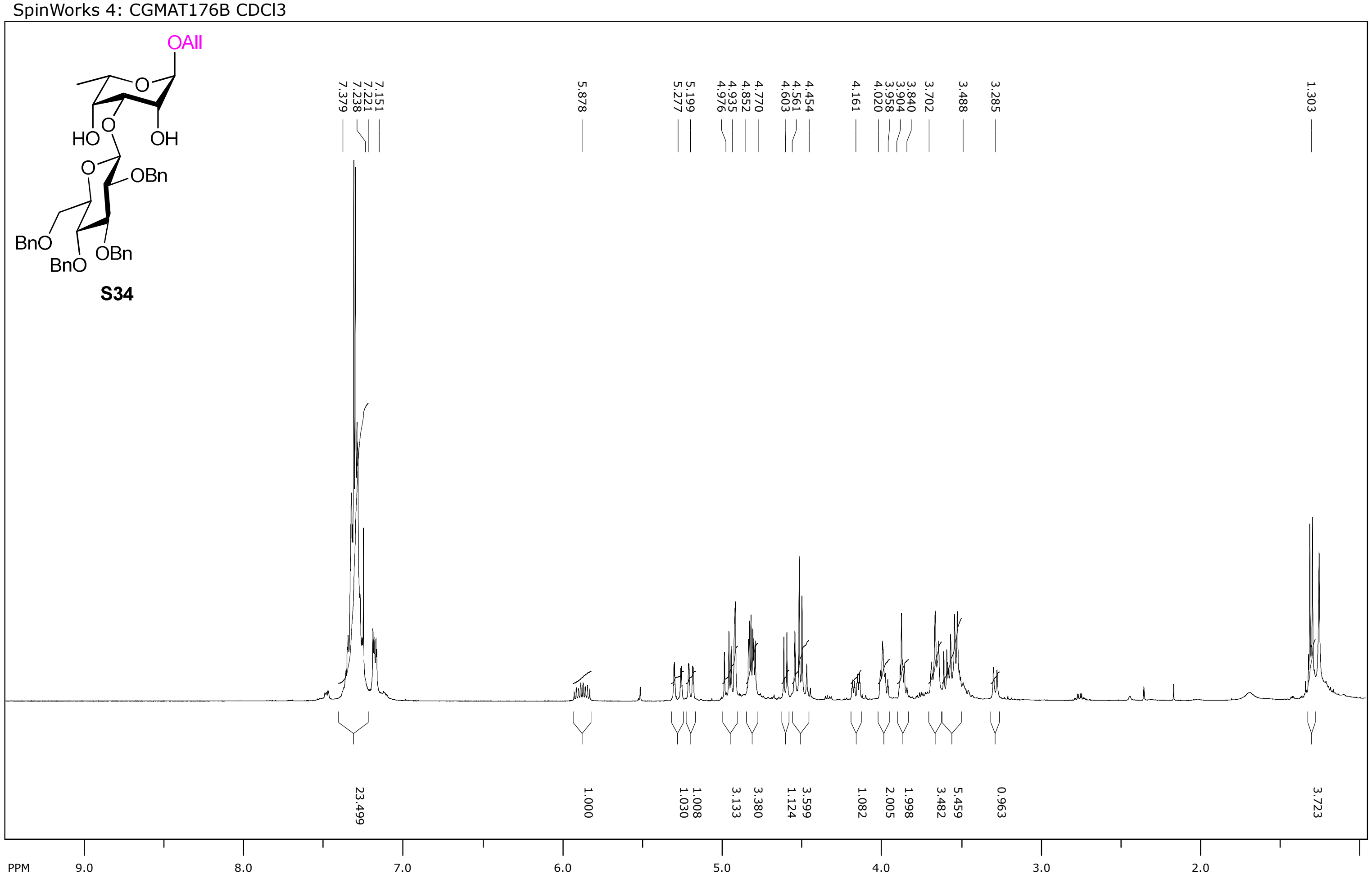

file: ...S Marielle\RMN LPS\CGMAT176B\1\fid expt: <zg30>
transmitter freq.: 400.132001 MHz
time domain size: 32768 points
width: 4807.69 Hz = 12.0153 ppm = 0.146719 Hz/pt
number of scans: 32

freq. of 0 ppm: 400.130014 MHz
processed size: 32768 complex points
LB: 0.300 GF: 0.0000

Supplementary Figure 108 | ^{13}C NMR spectra ($CDCl_3$, 100 MHz) of compound S34.

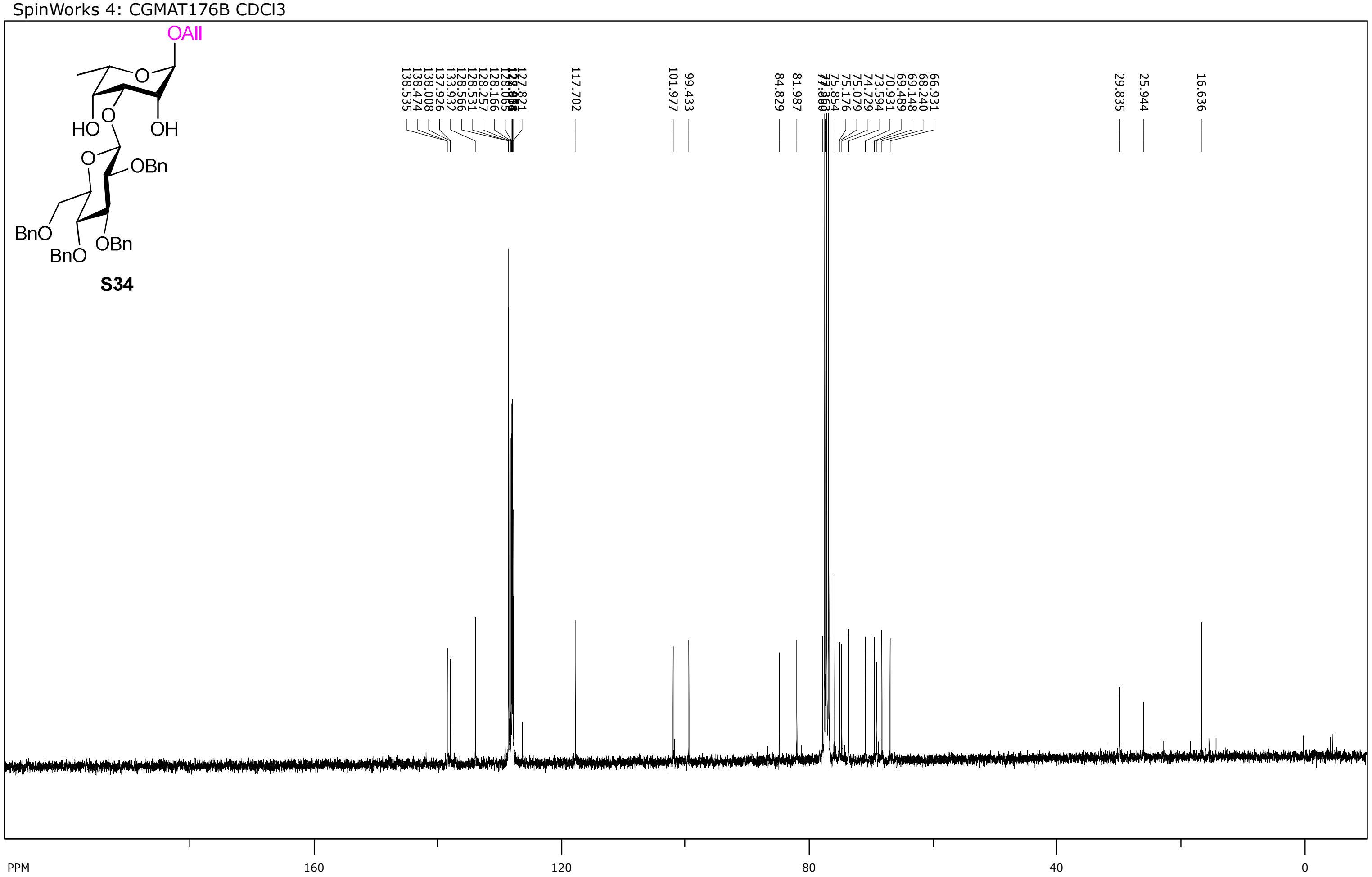

file: ...S Marielle\RMN LPS\CGMAT176B\3\fid expt: <zgpg30>
transmitter freq.: 100.622830 MHz
time domain size: 65536 points
width: 24038.46 Hz = 238.8967 ppm = 0.366798 Hz/pt
number of scans: 1024

freq. of 0 ppm: 100.612757 MHz
processed size: 32768 complex points
LB: 1.000 GF: 0.0000

Supplementary Figure 109 │ ^{1}H NMR spectra ($CDCl_3$, 400 MHz) of compound S35.

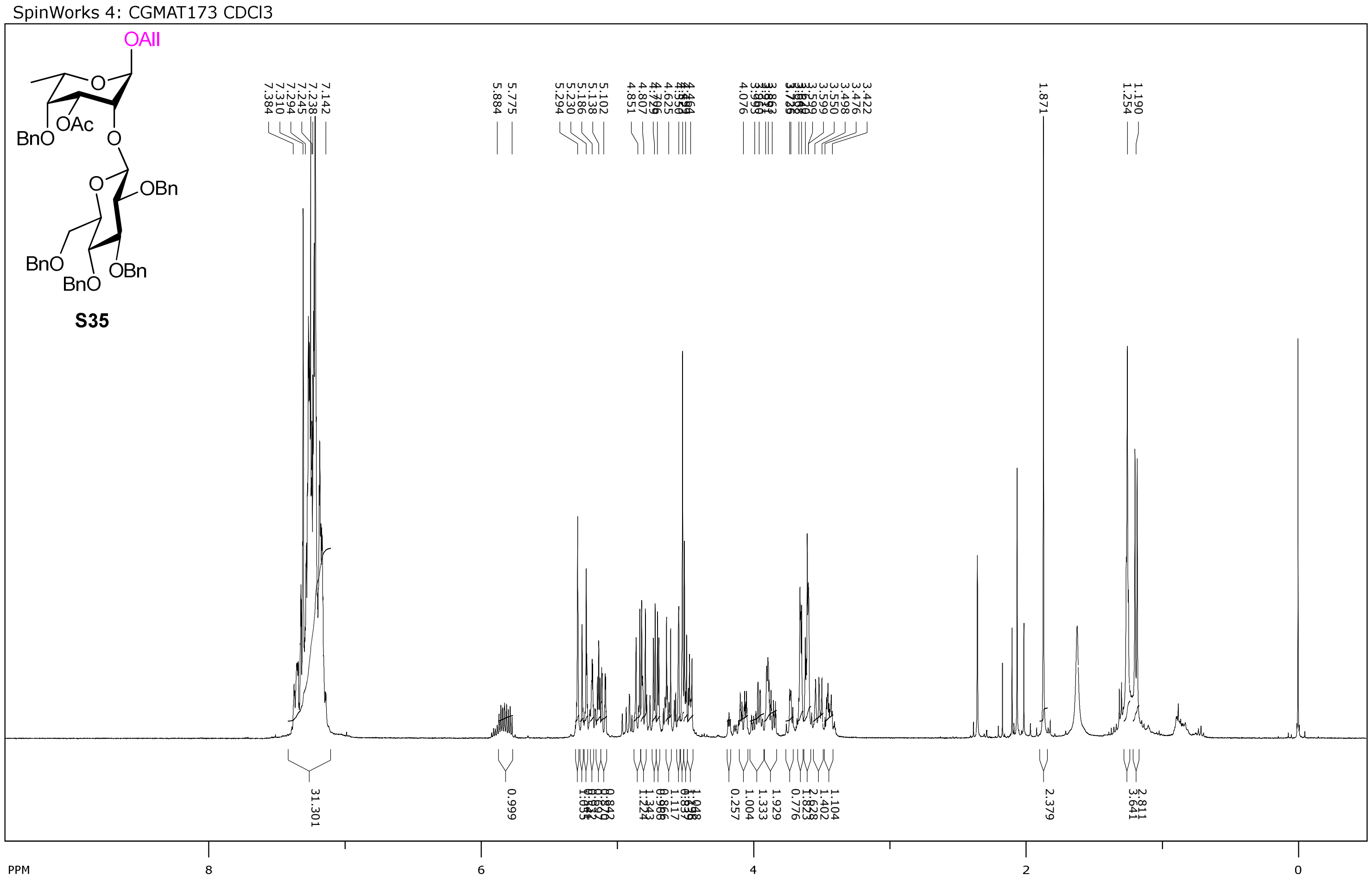

file: ...eau dossier\RMN LPS\CGMAT173\1\fid expt: <zg30>
transmitter freq.: 400.132001 MHz
time domain size: 32768 points
width: 4807.69 Hz = 12.0153 ppm = 0.146719 Hz/pt
number of scans: 16

freq. of 0 ppm: 400.130012 MHz
processed size: 32768 complex points
LB: 0.300 GF: 0.0000

Supplementary Figure 110 | ^{13}C NMR spectra ($CDCl_3$, 100 MHz) of compound S35.

SpinWorks 4: CGMAT173 CDCl3

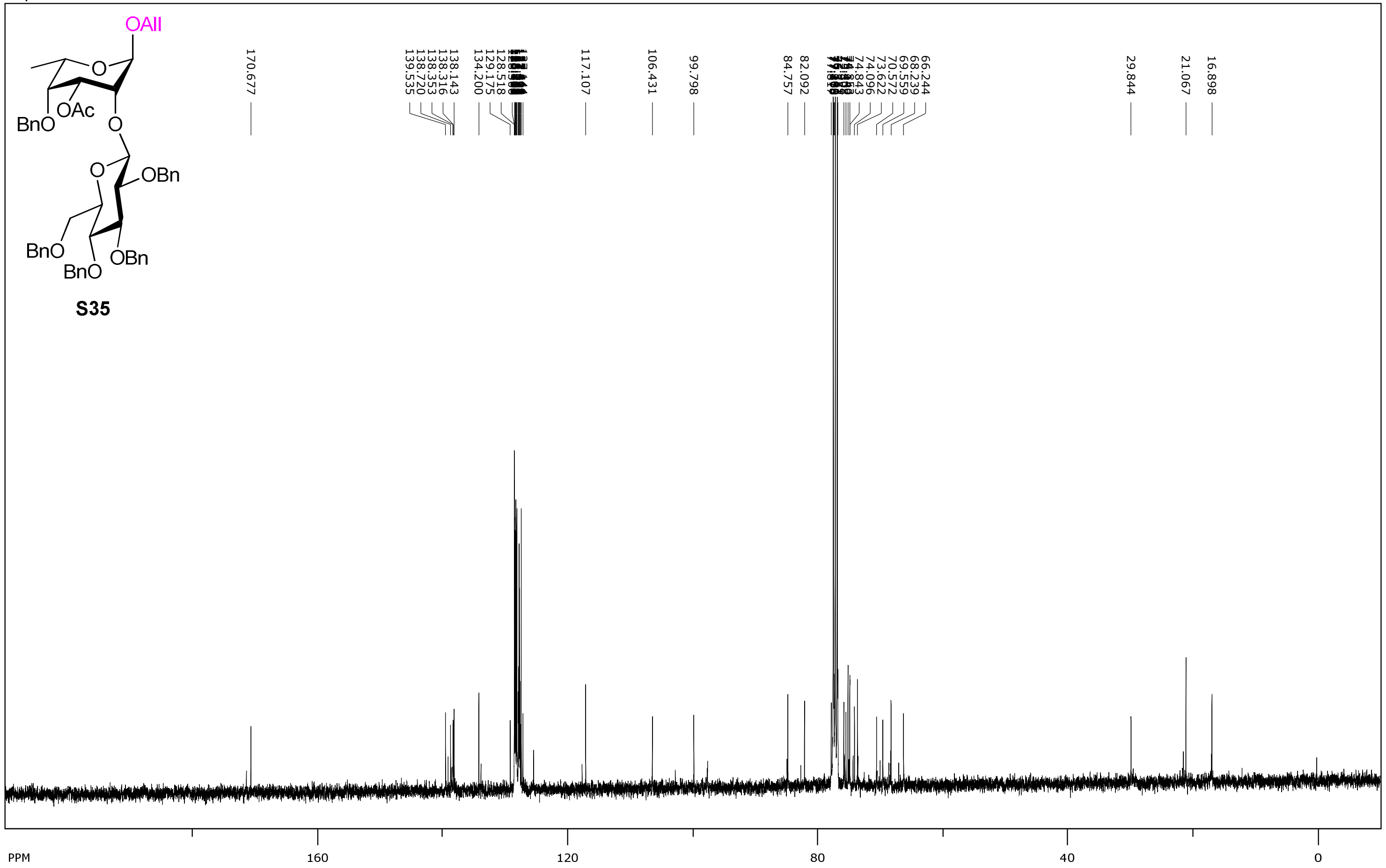

file: ...eau dossier\RMN LPS\CGMAT173\4\fid expt: <zgpg30>
transmitter freq.: 100.622830 MHz
time domain size: 65536 points
width: 24038.46 Hz = 238.8967 ppm = 0.366798 Hz/pt
number of scans: 1024

freq. of 0 ppm: 100.612756 MHz
processed size: 32768 complex points
LB: 1.000 GF: 0.0000

Supplementary Figure 111 | ^{1}H NMR spectra ($CDCl_3$, 400 MHz) of compound 30.

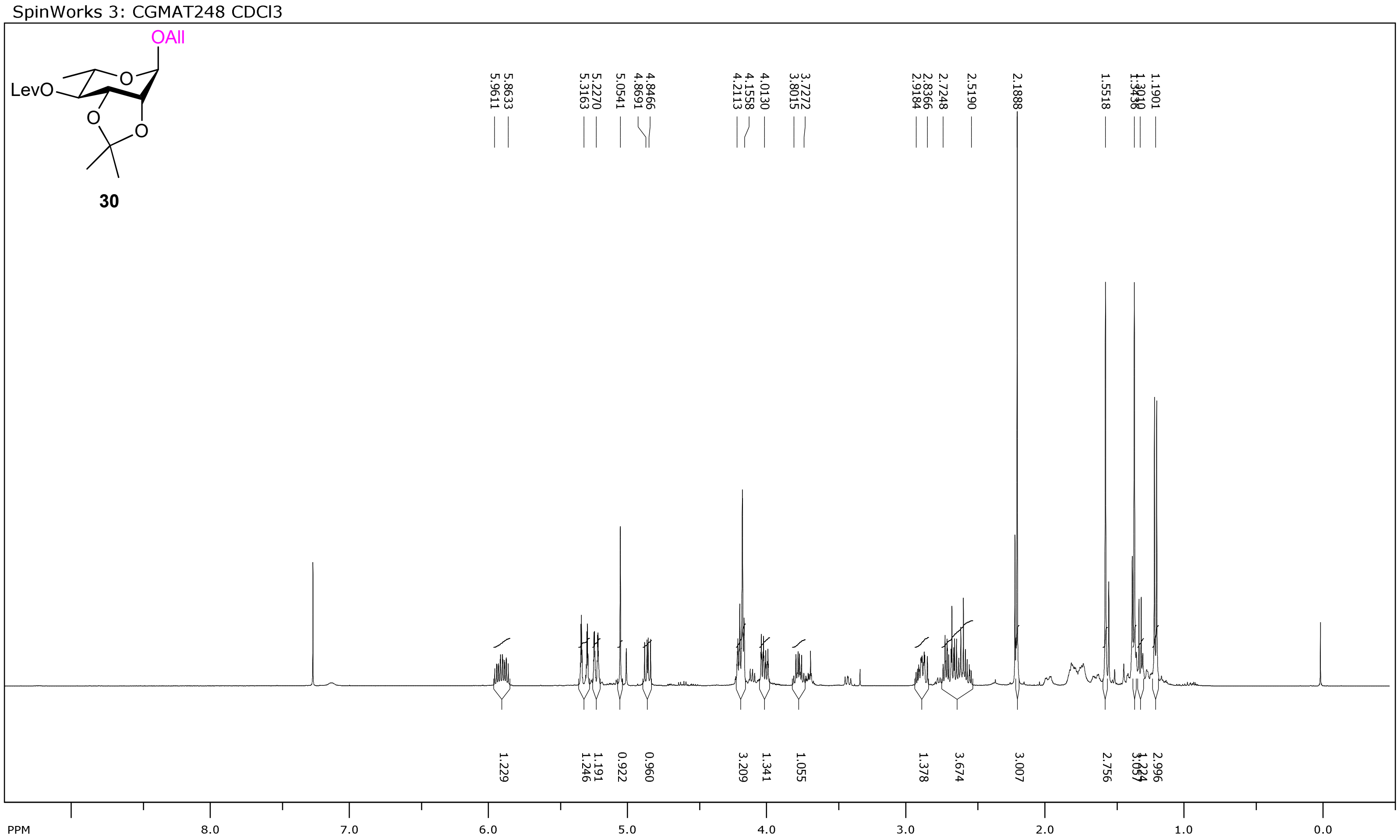

file: ...eau dossier\RMN LPS\CGMAT248\1\fid expt: <zg30>
transmitter freq.: 400.132001 MHz
time domain size: 32768 points
width: 4807.69 Hz = 12.0153 ppm = 0.146719 Hz/pt
number of scans: 32

freq. of 0 ppm: 400.130005 MHz
processed size: 32768 complex points
LB: 0.300 GF: 0.0000
Hz/cm: 160.052 ppm/cm: 0.40000

Supplementary Figure 112 | ^{13}C NMR spectra ($CDCl_3$, 100 MHz) of compound 30.

SpinWorks 3: CGMAT248 CDCl3

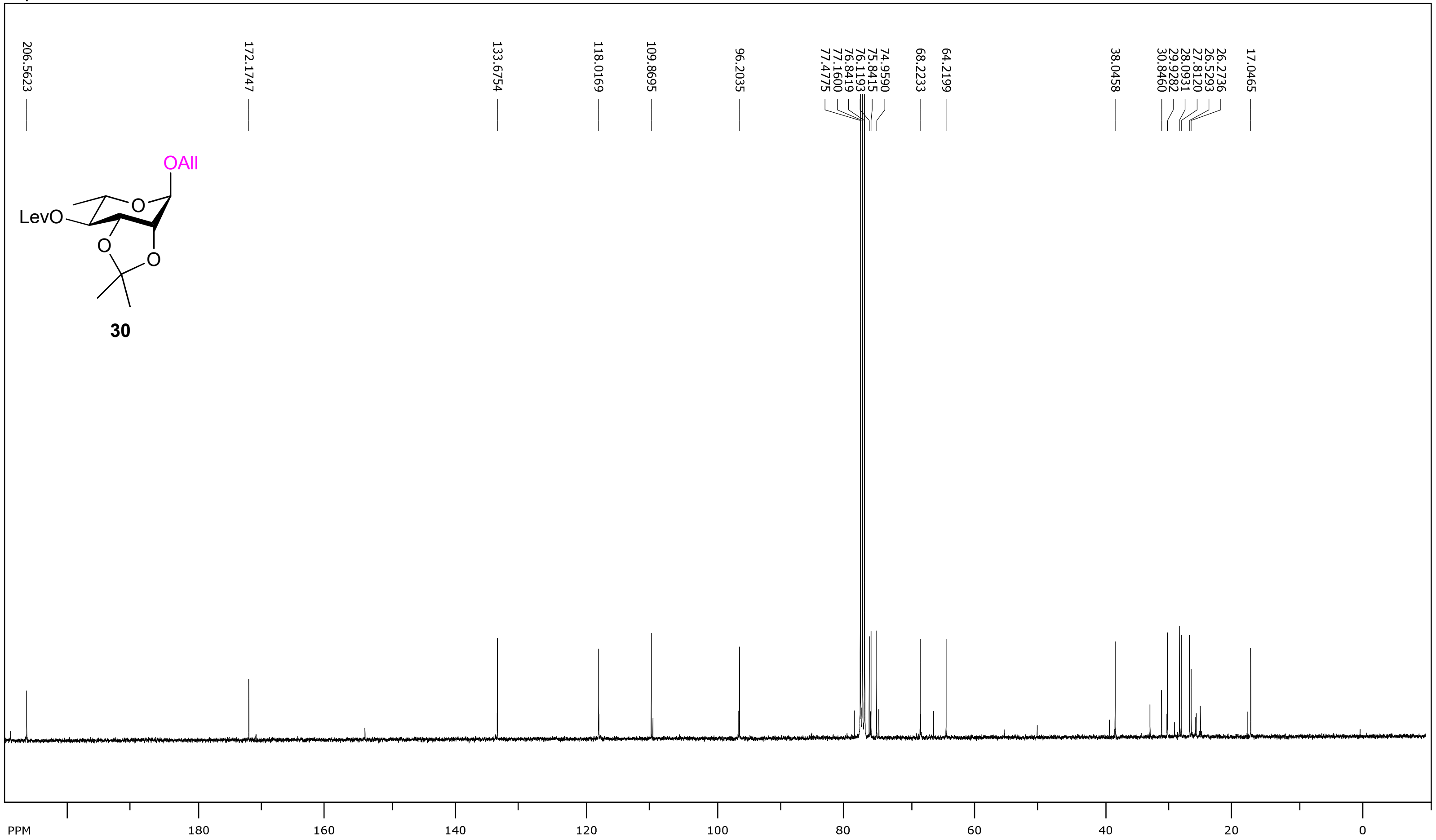

file: ...eau dossier\RMN LPS\CGMAT248\3\fid expt: <zgpg30>
transmitter freq.: 100.622830 MHz
time domain size: 65536 points
width: 24038.46 Hz = 238.8967 ppm = 0.366798 Hz/pt
number of scans: 1024

freq. of 0 ppm: 100.612755 MHz
processed size: 32768 complex points
LB: 1.000 GF: 0.0000
Hz/cm: 885.392 ppm/cm: 8.79912

Supplementary Figure 113 | ^{1}H NMR spectra ($CDCl_3$, 400 MHz) of compound 31.

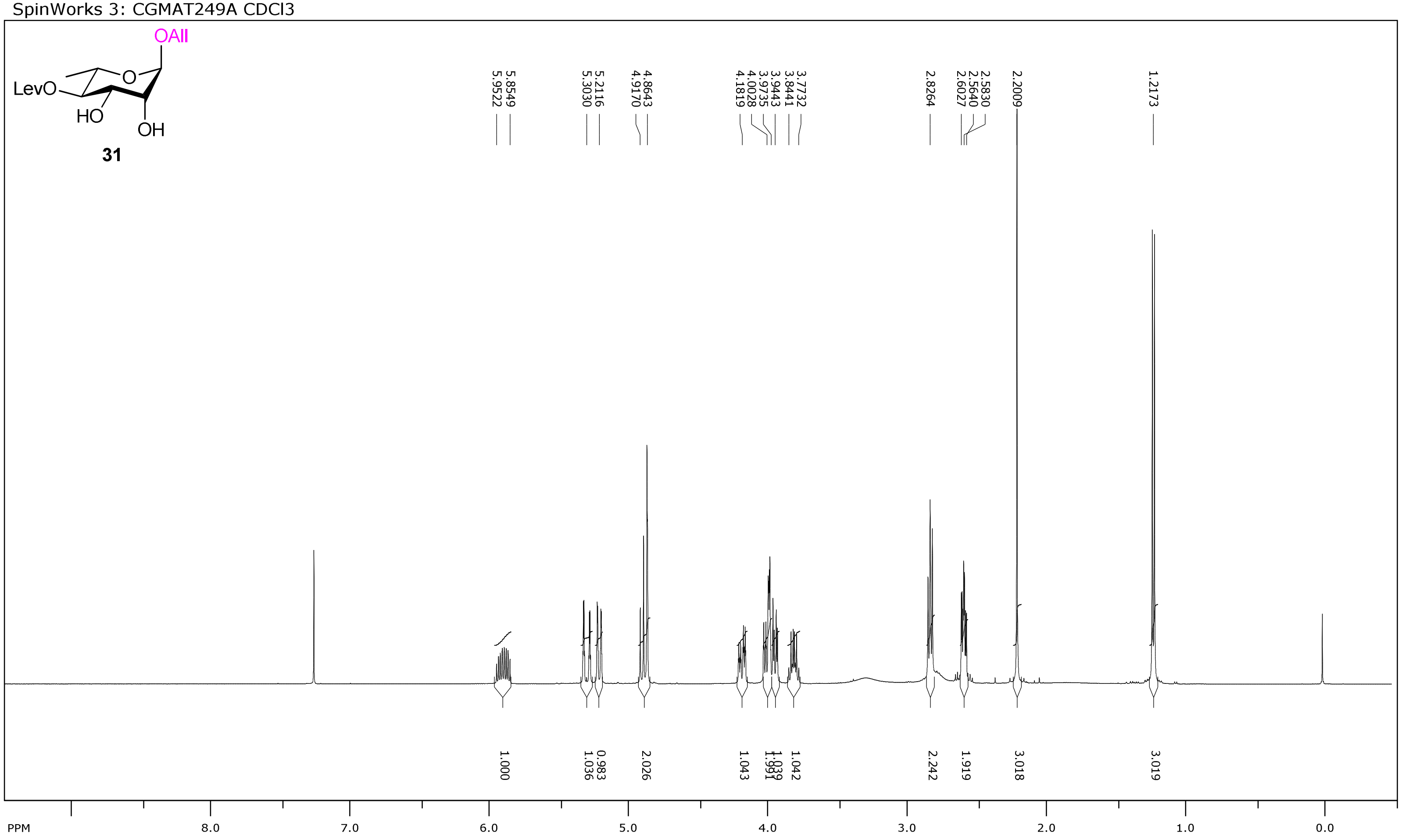

file: ...au dossier\RMN LPS\CGMAT249A\1\fid expt: <zg30>
transmitter freq.: 400.132001 MHz
time domain size: 32768 points
width: 4807.69 Hz = 12.0153 ppm = 0.146719 Hz/pt
number of scans: 32

freq. of 0 ppm: 400.130007 MHz
processed size: 32768 complex points
LB: 0.300 GF: 0.0000
Hz/cm: 160.052 ppm/cm: 0.40000

Supplementary Figure 114 | ^{13}C NMR spectra ($CDCl_3$, 100 MHz) of compound 31.

SpinWorks 3: CGMAT249A CDCl3

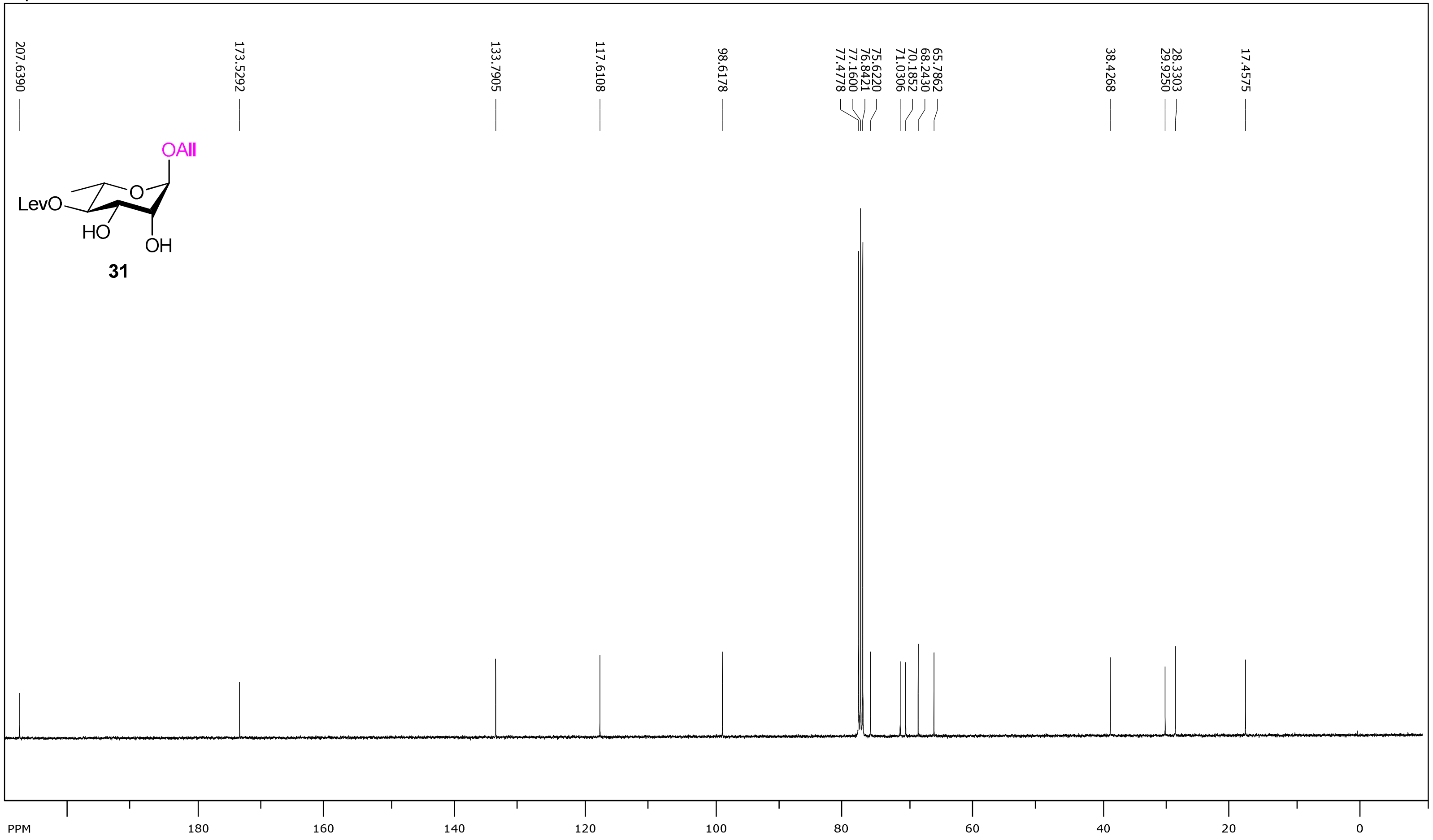

file: ...au dossier\RMN LPS\CGMAT249A\3\fid expt: <zgpg30>
transmitter freq.: 100.622830 MHz
time domain size: 65536 points
width: 24038.46 Hz = 238.8967 ppm = 0.366798 Hz/pt
number of scans: 1024

freq. of 0 ppm: 100.612755 MHz
processed size: 32768 complex points
LB: 1.000 GF: 0.0000
Hz/cm: 885.392 ppm/cm: 8.79912

Supplementary Figure 115 | ^{1}H NMR spectra ($CDCl_3$, 400 MHz) of compound 32.

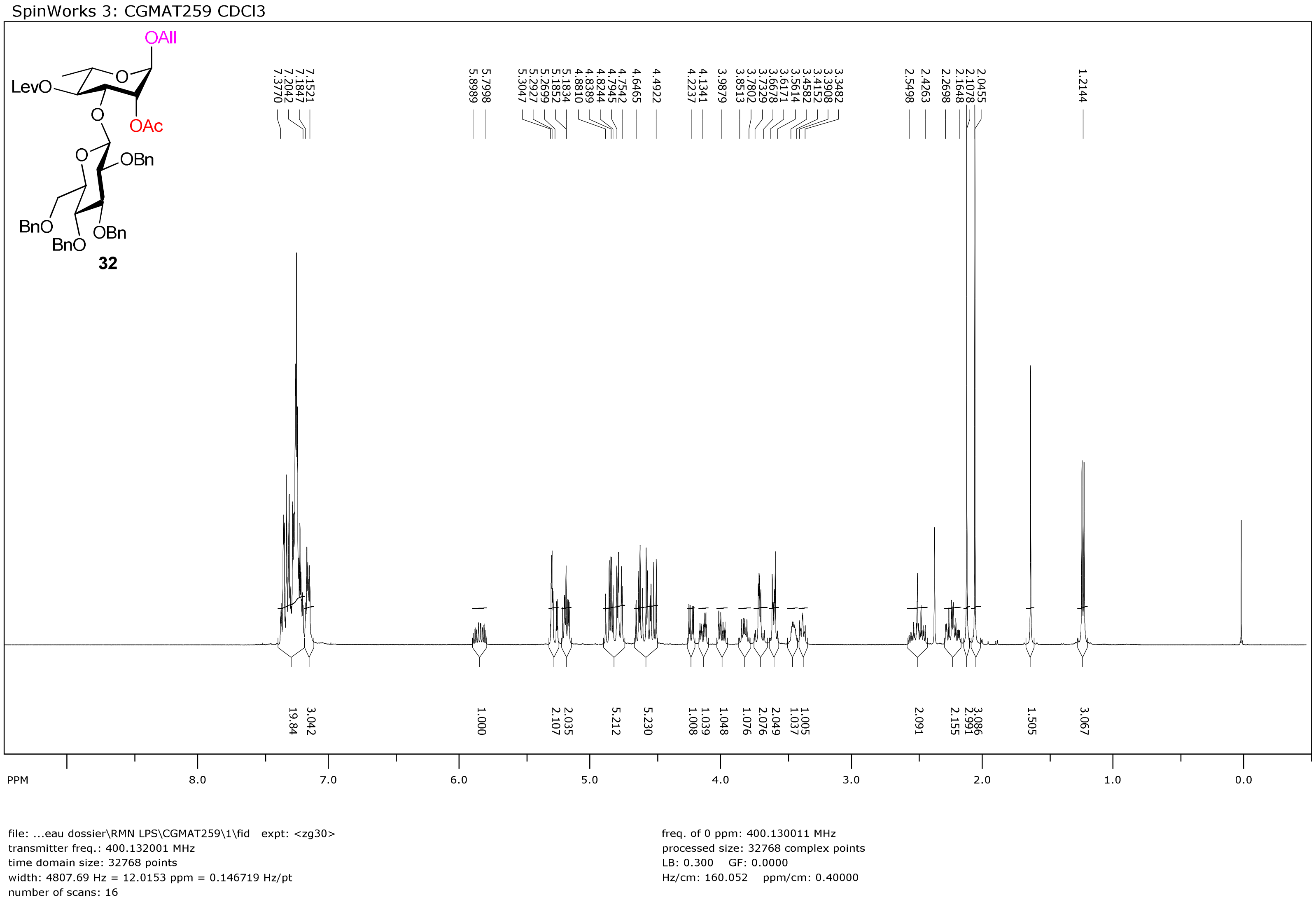

Supplementary Figure 116 | ^{13}C NMR spectra ($CDCl_3$, 100 MHz) of compound 32.

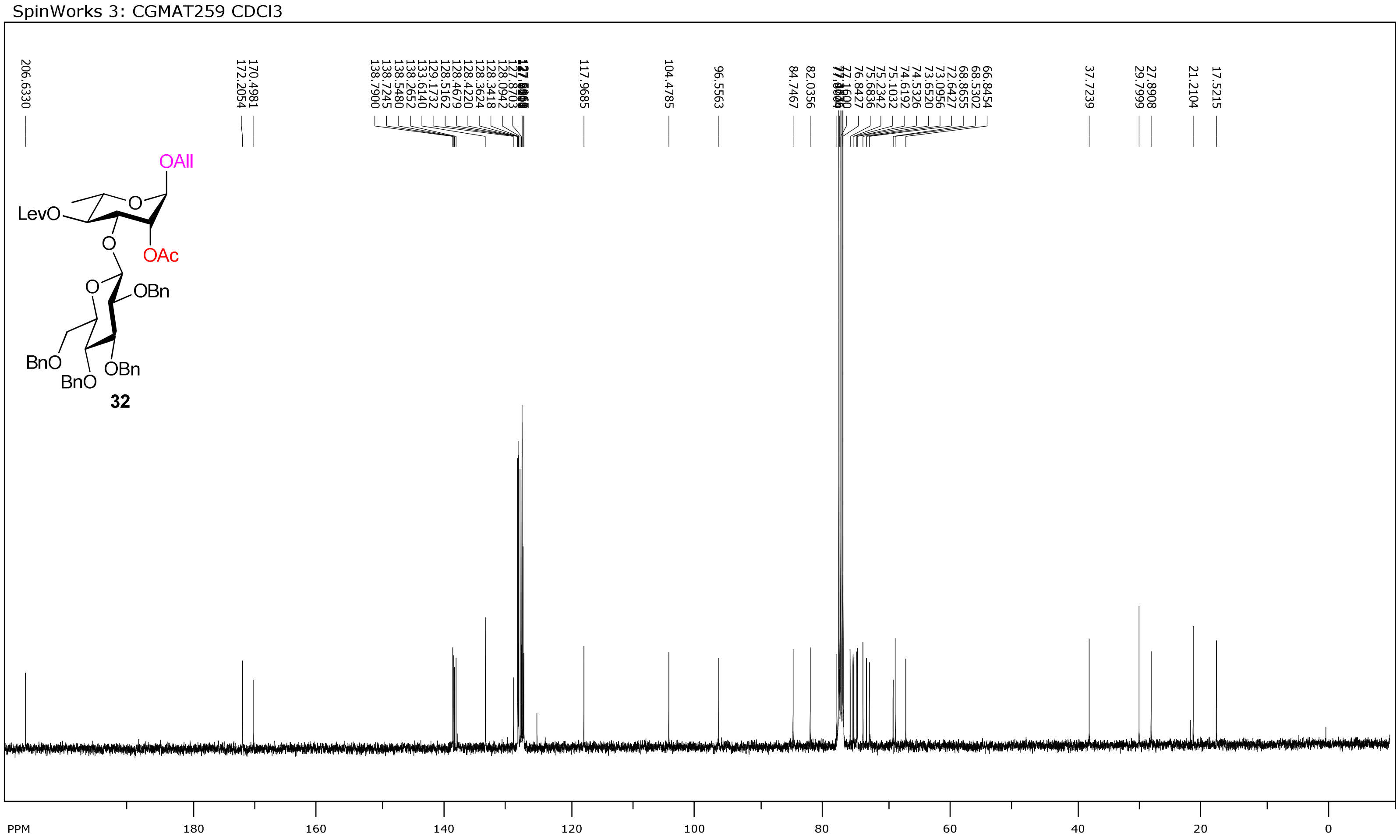

file: ...eau dossier\RMN LPS\CGMAT259\4\fid expt: <zgpg30>
transmitter freq.: 100.622830 MHz
time domain size: 65536 points
width: 24038.46 Hz = 238.8967 ppm = 0.366798 Hz/pt
number of scans: 1024

freq. of 0 ppm: 100.612756 MHz
processed size: 32768 complex points
LB: 1.000 GF: 0.0000
Hz/cm: 885.392 ppm/cm: 8.79912

Supplementary Figure 117 │ ^{1}H NMR spectra ($CDCl_3$, 400 MHz) of compound 33.

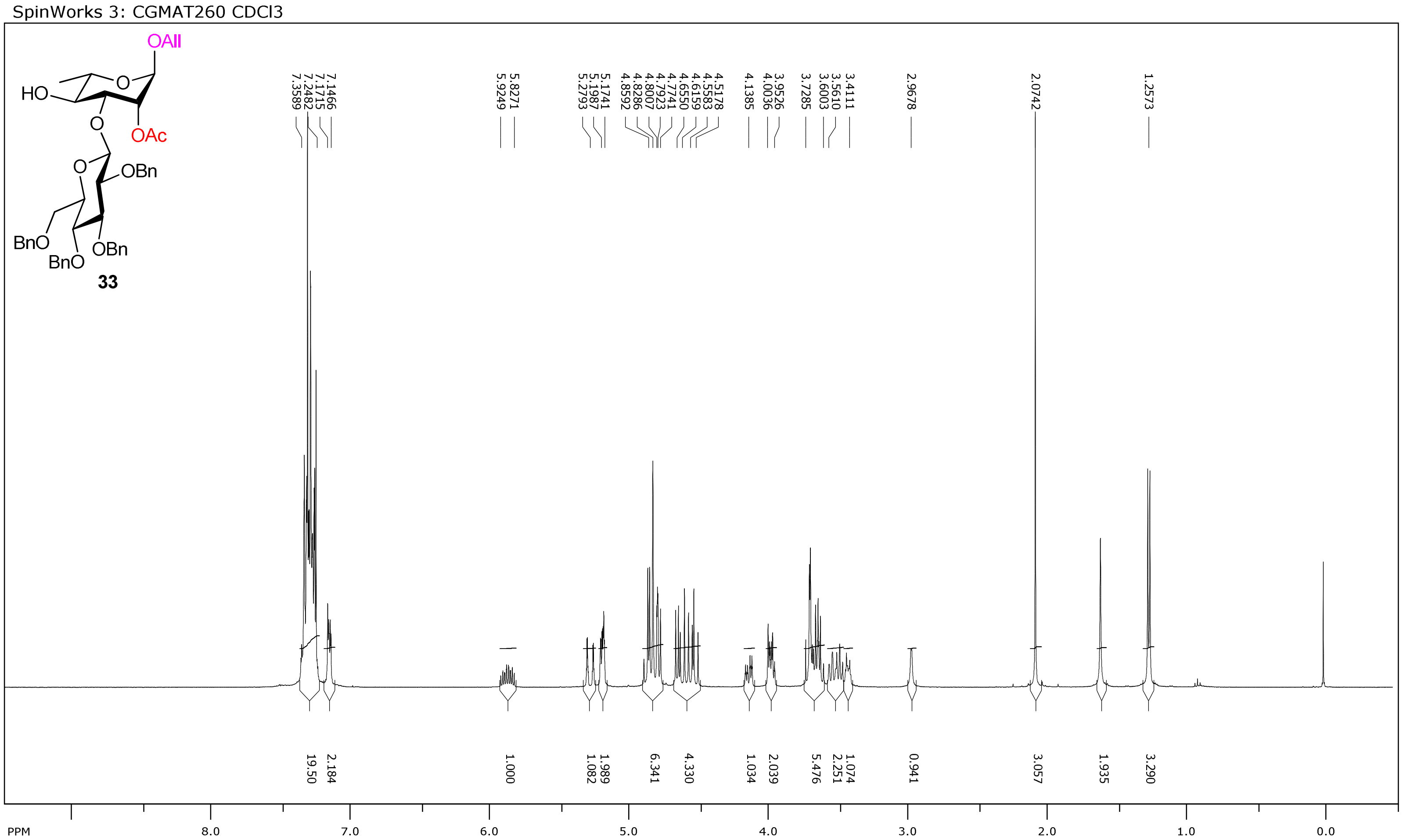

file: ...eau dossier\RMN LPS\CGMAT260\1\fid expt: <zg30>
transmitter freq.: 400.132001 MHz
time domain size: 32768 points
width: 4807.69 Hz = 12.0153 ppm = 0.146719 Hz/pt
number of scans: 16

freq. of 0 ppm: 400.130011 MHz
processed size: 32768 complex points
LB: 0.300 GF: 0.0000
Hz/cm: 160.052 ppm/cm: 0.40000

Supplementary Figure 118 | ^{13}C NMR spectra ($CDCl_3$, 100 MHz) of compound 33.

SpinWorks 3: CGMAT260 CDCl3

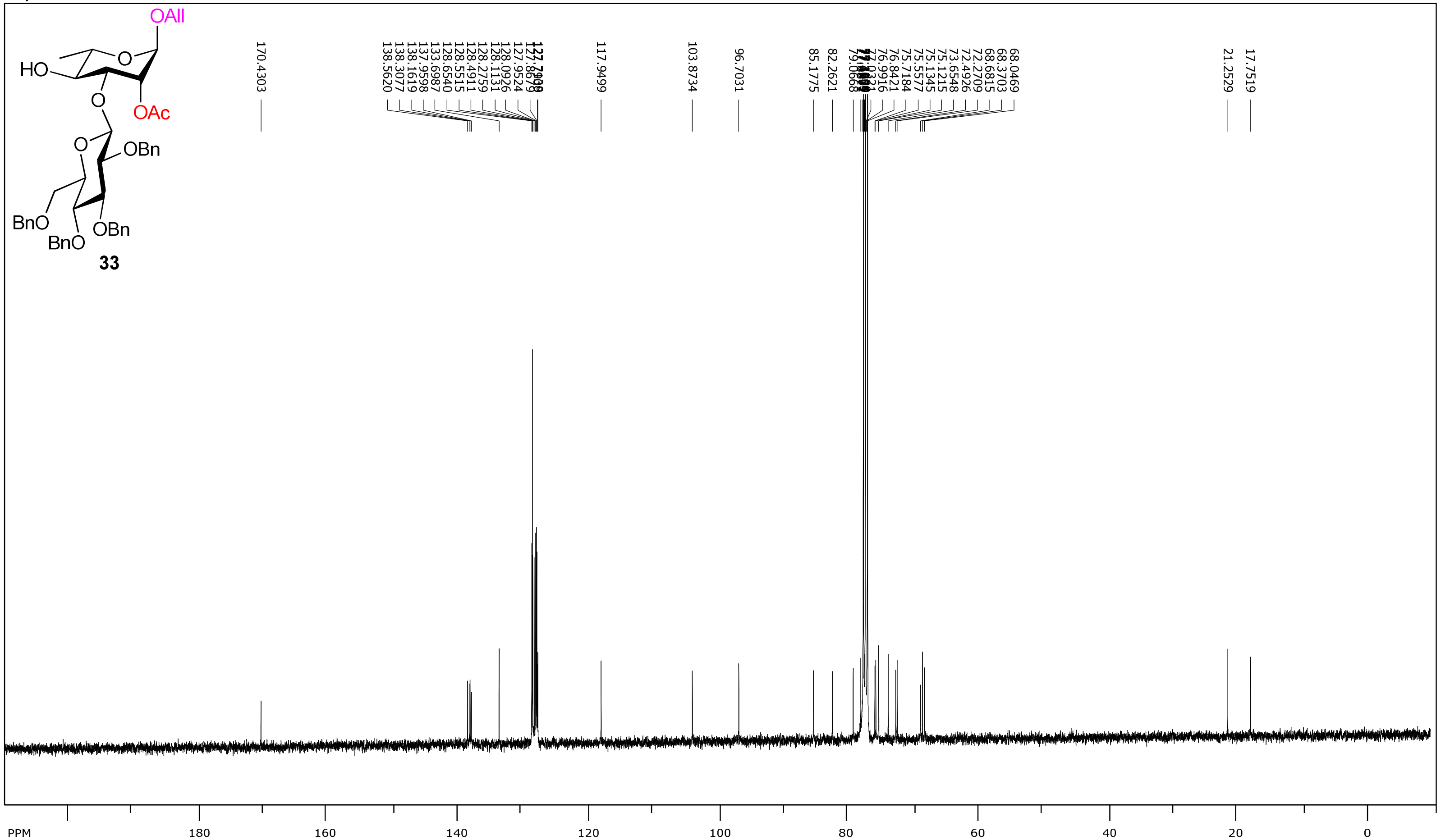

file: ...eau dossier\RMN LPS\CGMAT260\3\fid expt: <zgpg30>
transmitter freq.: 100.622830 MHz
time domain size: 65536 points
width: 24038.46 Hz = 238.8967 ppm = 0.366798 Hz/pt
number of scans: 1024

freq. of 0 ppm: 100.612755 MHz
processed size: 32768 complex points
LB: 1.000 GF: 0.0000
Hz/cm: 885.392 ppm/cm: 8.79912

Supplementary Figure 119 │ ^{1}H NMR spectra ($CDCl_3$, 400 MHz) of compound 34.

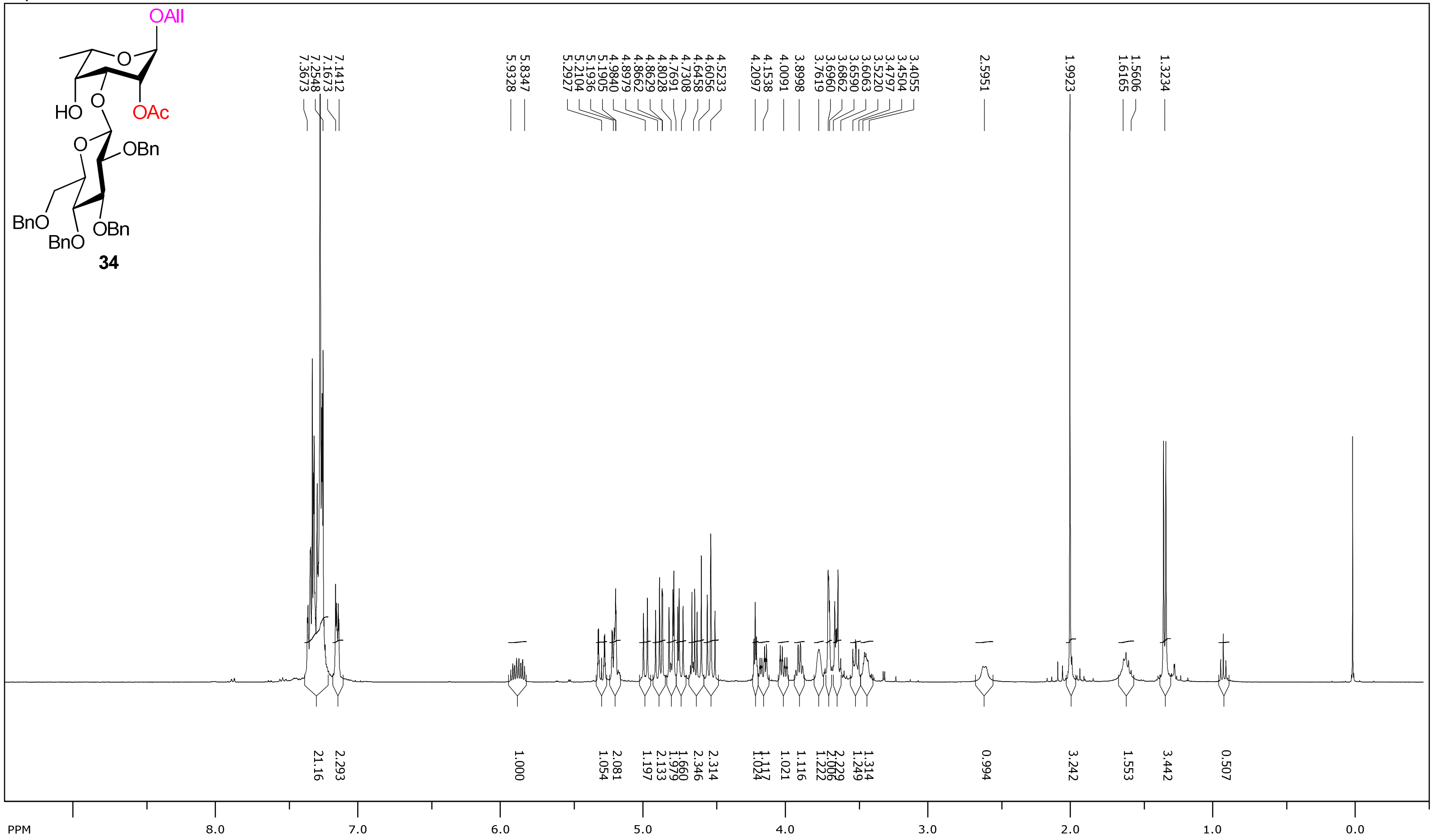

file: ...eau dossier\RMN LPS\CGMAT261\1\fid expt: <zg30>
transmitter freq.: 400.132001 MHz
time domain size: 32768 points
width: 4807.69 Hz = 12.0153 ppm = 0.146719 Hz/pt
number of scans: 32

freq. of 0 ppm: 400.130012 MHz
processed size: 32768 complex points
LB: 0.300 GF: 0.0000
Hz/cm: 160.052 ppm/cm: 0.40000

Supplementary Figure 120 │ ^{13}C NMR spectra ($CDCl_3$, 100 MHz) of compound 34.

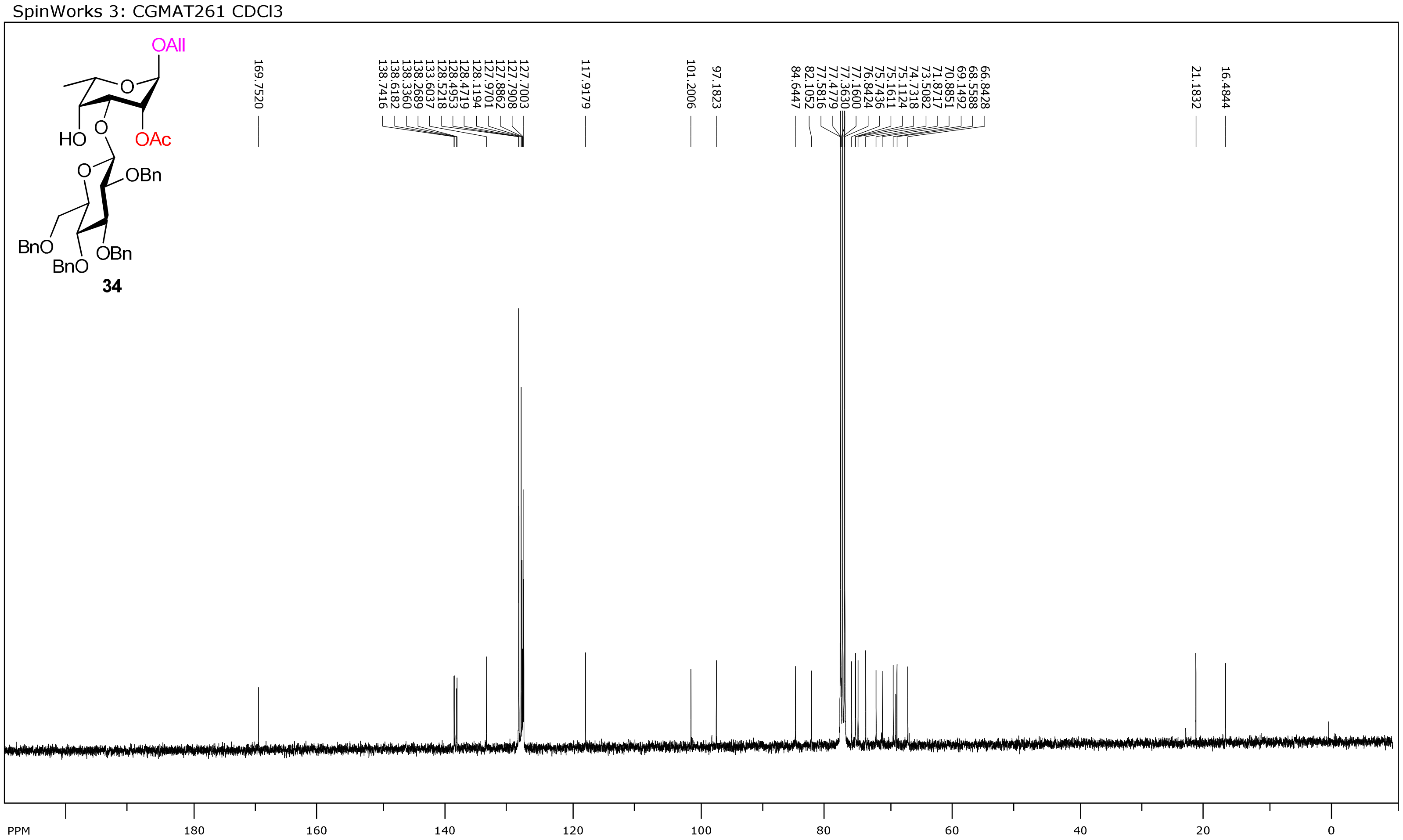

file: ...eau dossier\RMN LPS\CGMAT261\3\fid expt: <zgpg30>
transmitter freq.: 100.622830 MHz
time domain size: 65536 points
width: 24038.46 Hz = 238.8967 ppm = 0.366798 Hz/pt
number of scans: 1024

freq. of 0 ppm: 100.612755 MHz
processed size: 32768 complex points
LB: 1.000 GF: 0.0000
Hz/cm: 885.392 ppm/cm: 8.79912

Supplementary Figure 121 │ ^{1}H NMR spectra (py-d_5, 400 MHz) of compound 35.

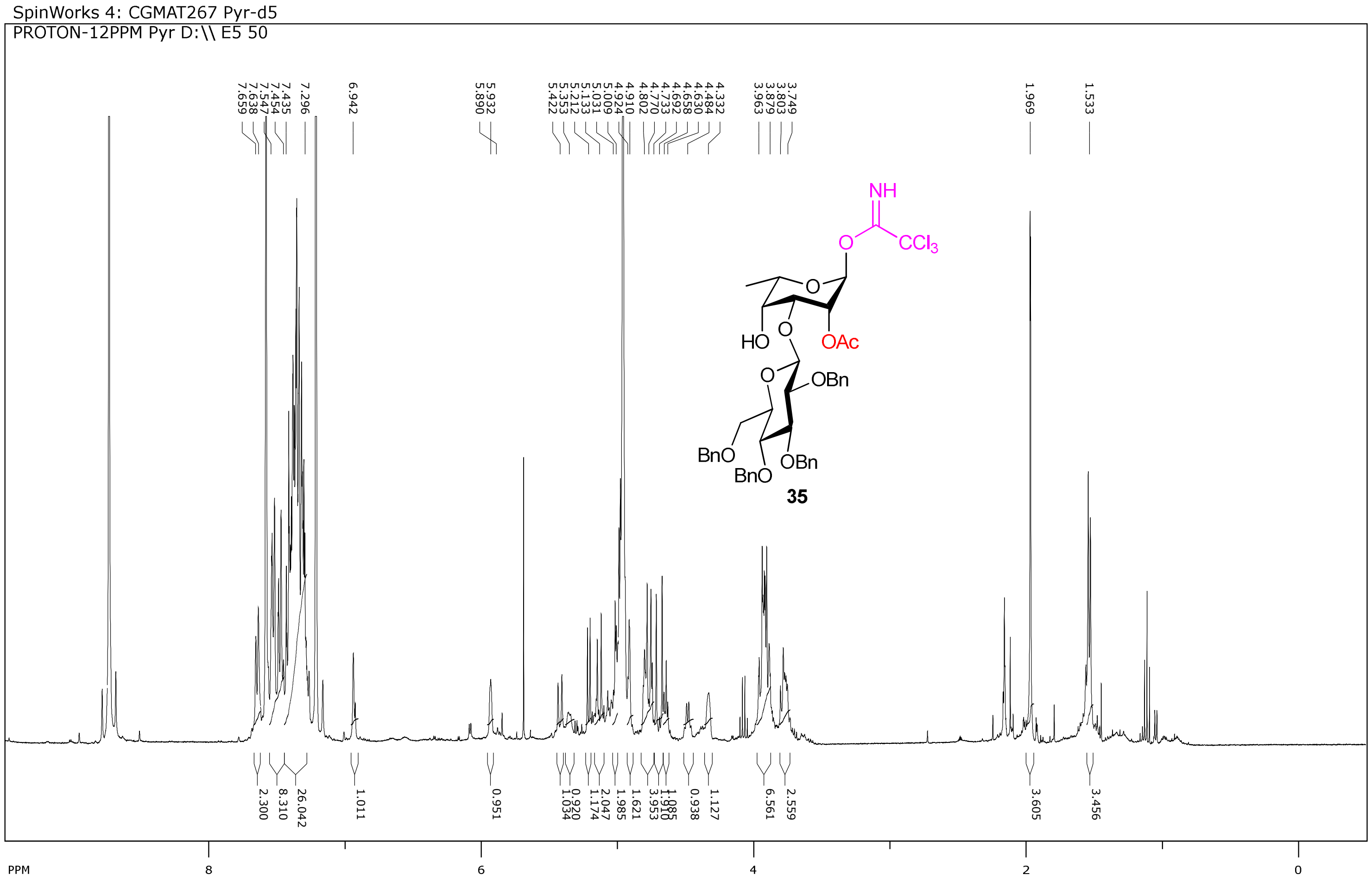

file: ...au dossier\RMN LPS\CCGMAT267\1\fid expt: <zg30>
transmitter freq.: 400.132001 MHz
time domain size: 32768 points
width: 4807.69 Hz = 12.0153 ppm = 0.146719 Hz/pt
number of scans: 32

freq. of 0 ppm: 400.130592 MHz
processed size: 32768 complex points
LB: 0.300 GF: 0.0000

Supplementary Figure 122 | ^{13}C NMR spectra (py-d_5, 100 MHz) of compound 35.

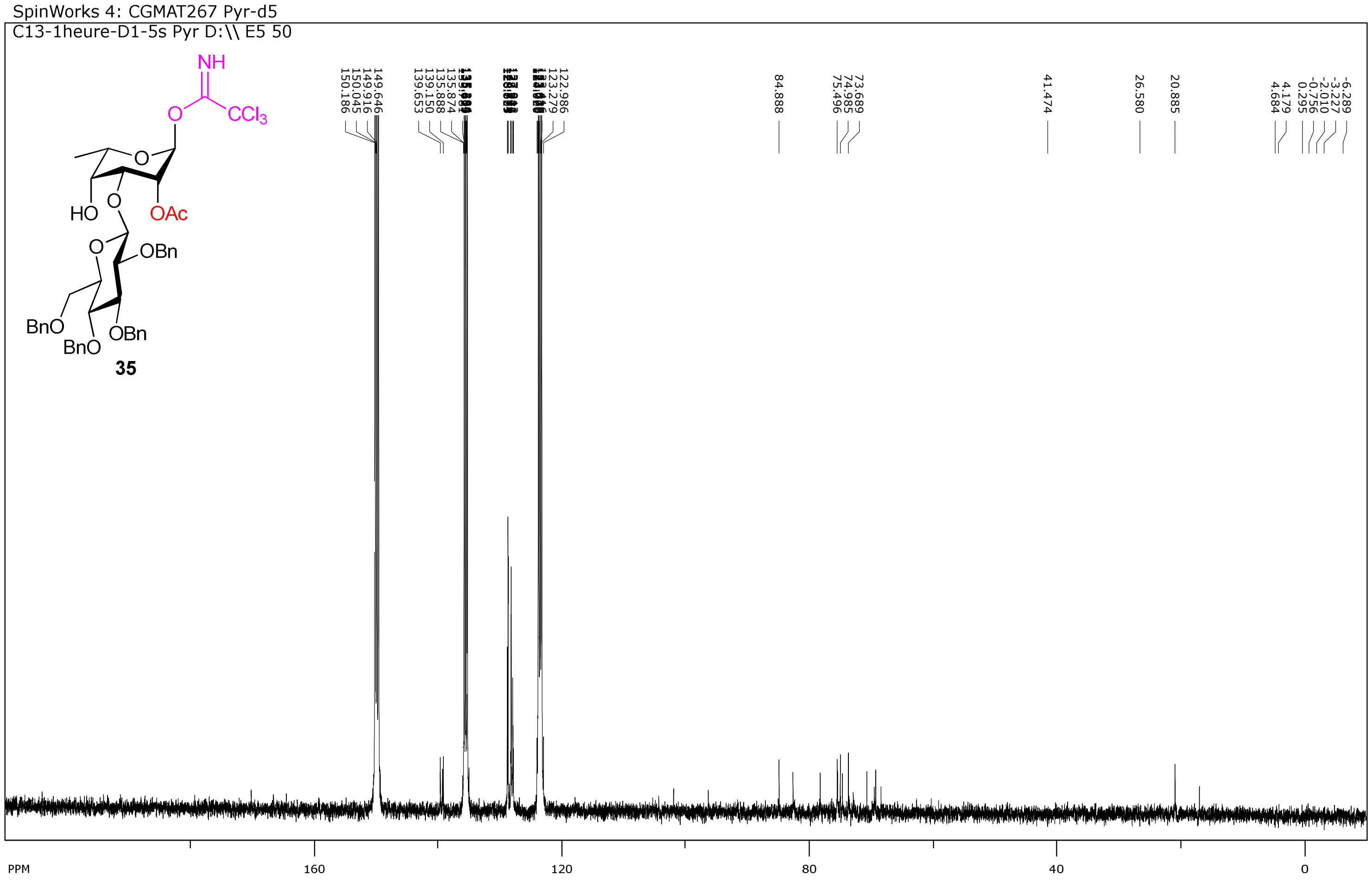

file: ...au dossier\RMN LPS\CCGMAT267\3\fid expt: <zgpg30>
transmitter freq.: 100.622830 MHz
time domain size: 65536 points
width: 24038.46 Hz = 238.8967 ppm = 0.366798 Hz/pt
number of scans: 1024

freq. of 0 ppm: 100.612893 MHz
processed size: 32768 complex points
LB: 1.000 GF: 0.0000

Supplementary Figure 123 │ ^{1}H NMR spectra ($CDCl_3$, 400 MHz) of compound 36.

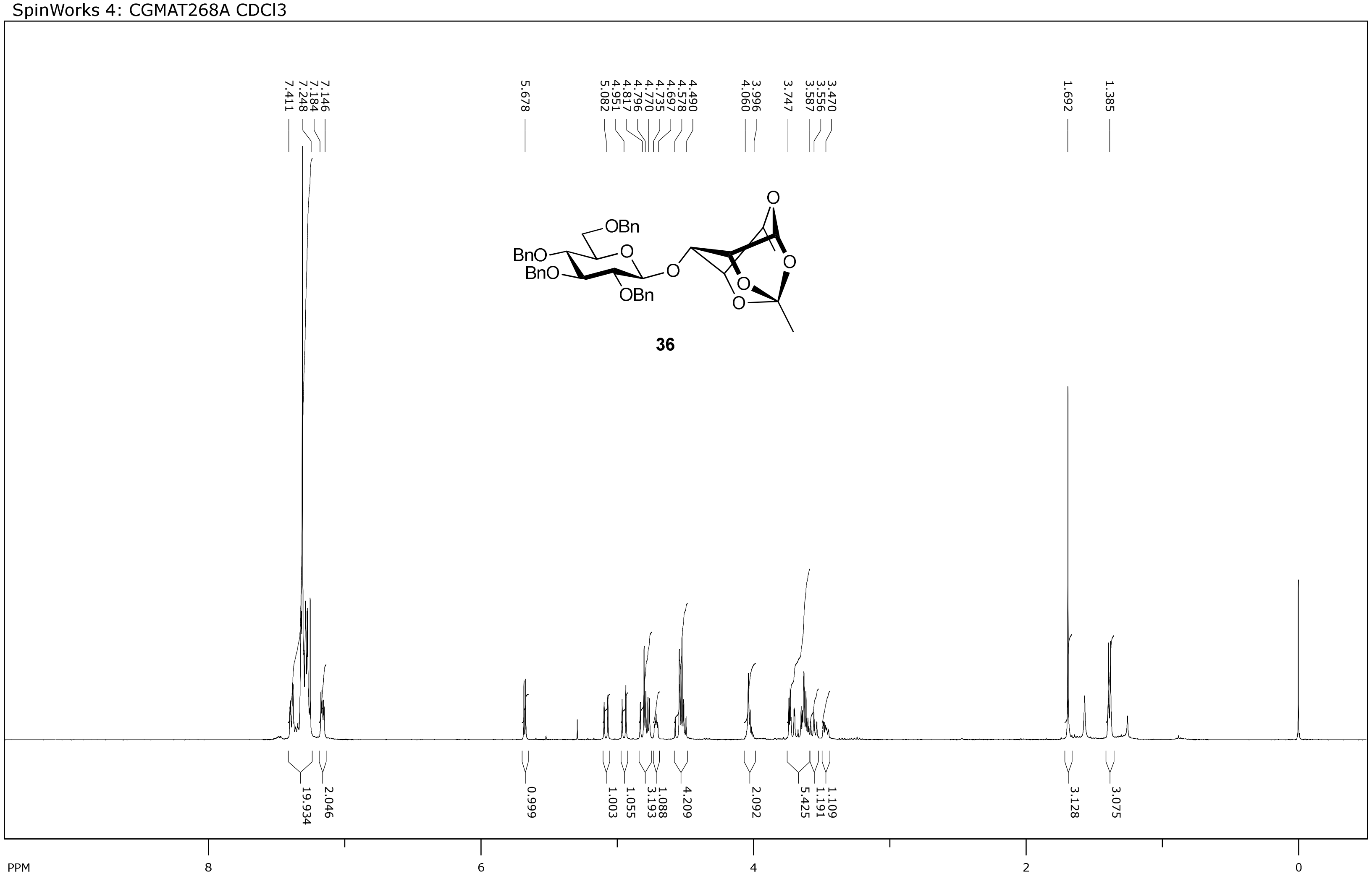

Supplementary Figure 124 | ^{13}C NMR spectra ($CDCl_3$, 100 MHz) of compound 36.

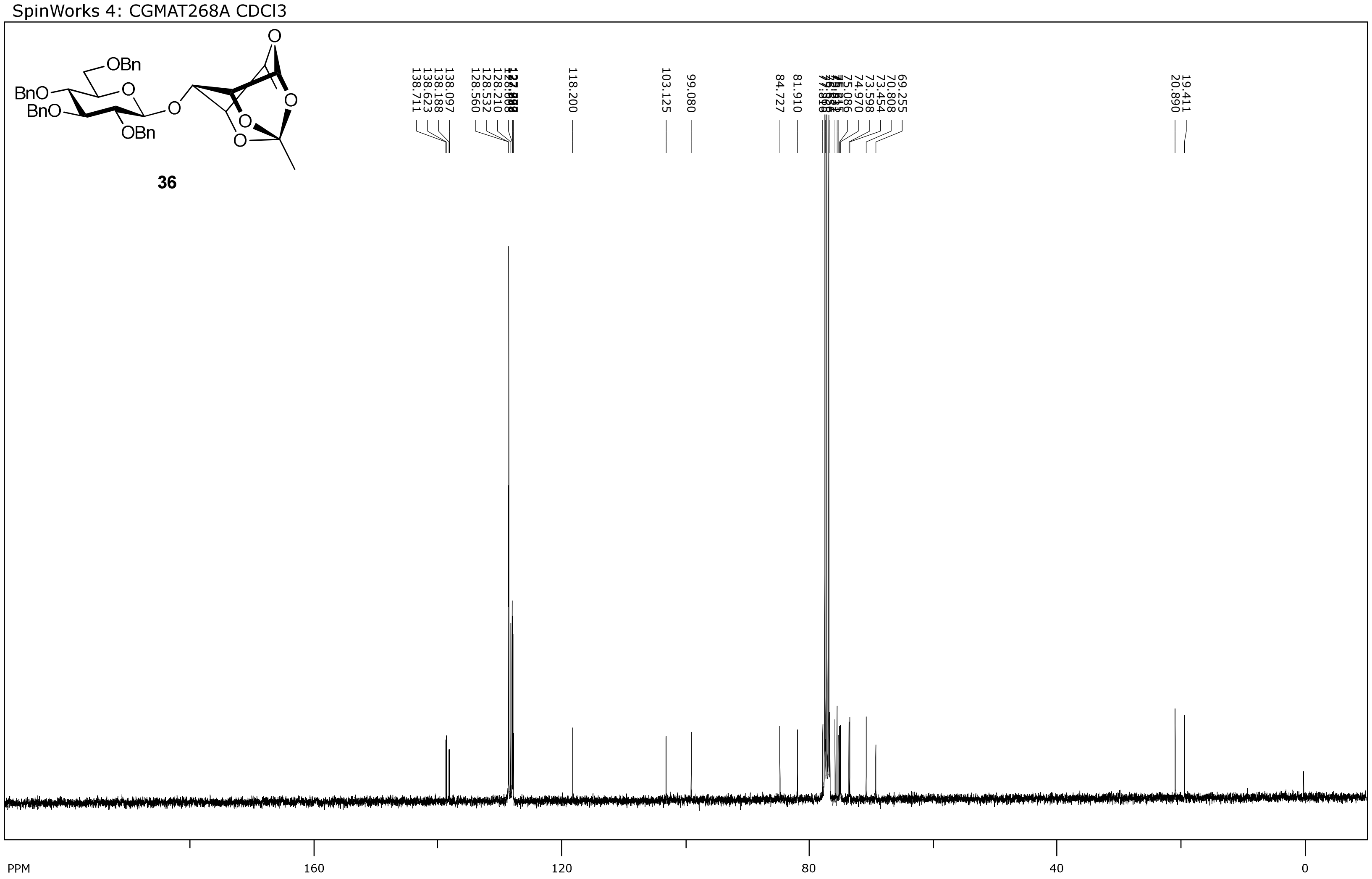

Supplementary Figure 125 │ ^{1}H NMR spectra ($CDCl_3$, 400 MHz) of compound 37.

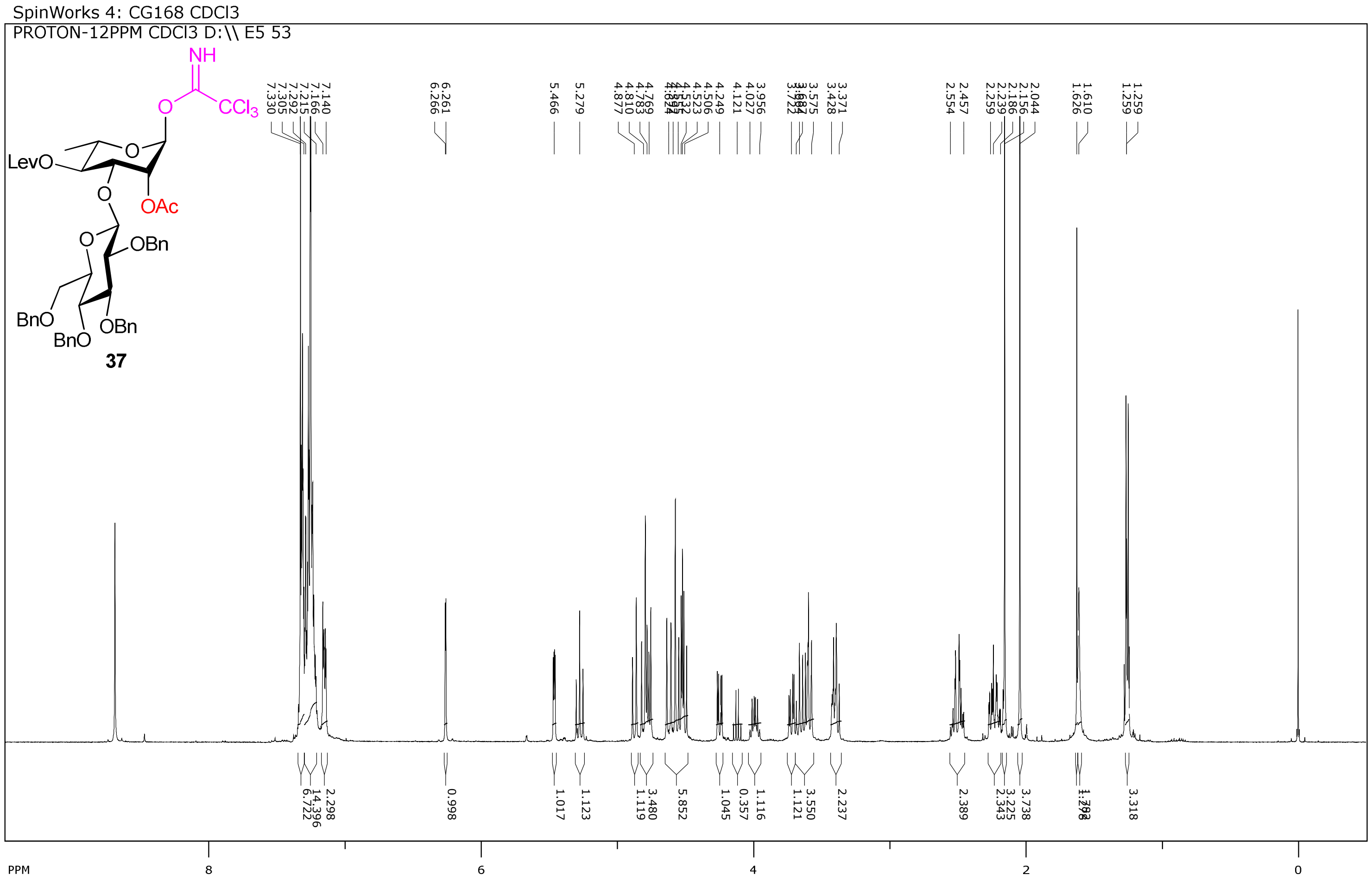

file: ...amigne\Desktop\RMN LPS\CG168\1\fid expt: <zg30>
transmitter freq.: 400.132001 MHz
time domain size: 32768 points
width: 4807.69 Hz = 12.0153 ppm = 0.146719 Hz/pt
number of scans: 32

freq. of 0 ppm: 400.130010 MHz
processed size: 32768 complex points
LB: 0.300 GF: 0.0000

Supplementary Figure 126 | ^{13}C NMR spectra ($CDCl_3$, 100 MHz) of compound 37.

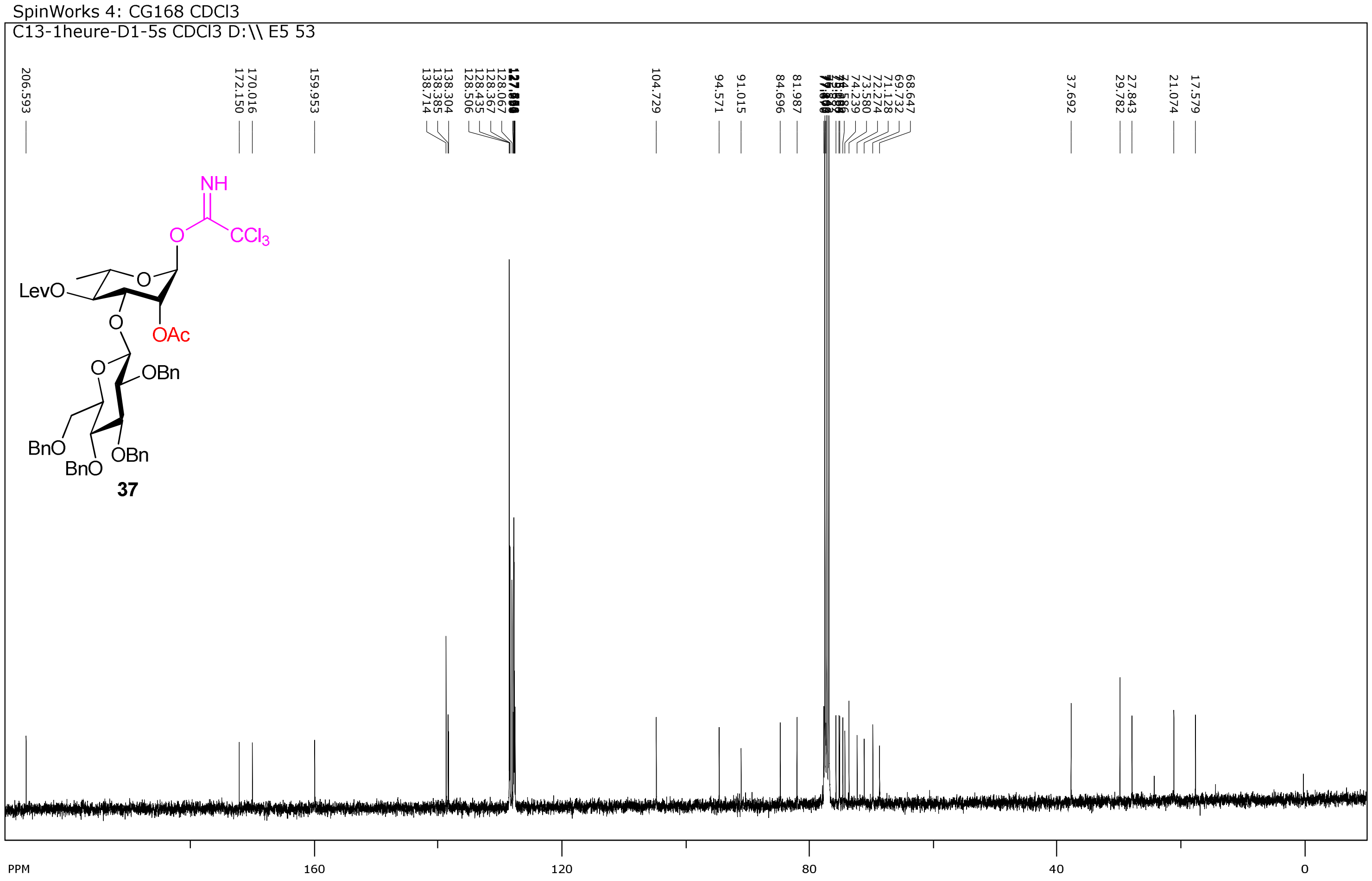

file: ...amigne\Desktop\RMN LPS\CG168\3\fid expt: <zgpg30>
transmitter freq.: 100.622830 MHz
time domain size: 65536 points
width: 24038.46 Hz = 238.8967 ppm = 0.366798 Hz/pt
number of scans: 1024

freq. of 0 ppm: 100.612755 MHz
processed size: 32768 complex points
LB: 1.000 GF: 0.0000

Supplementary Figure 127 │ ^{1}H NMR spectra ($CDCl_3$, 400 MHz) of compound 38.

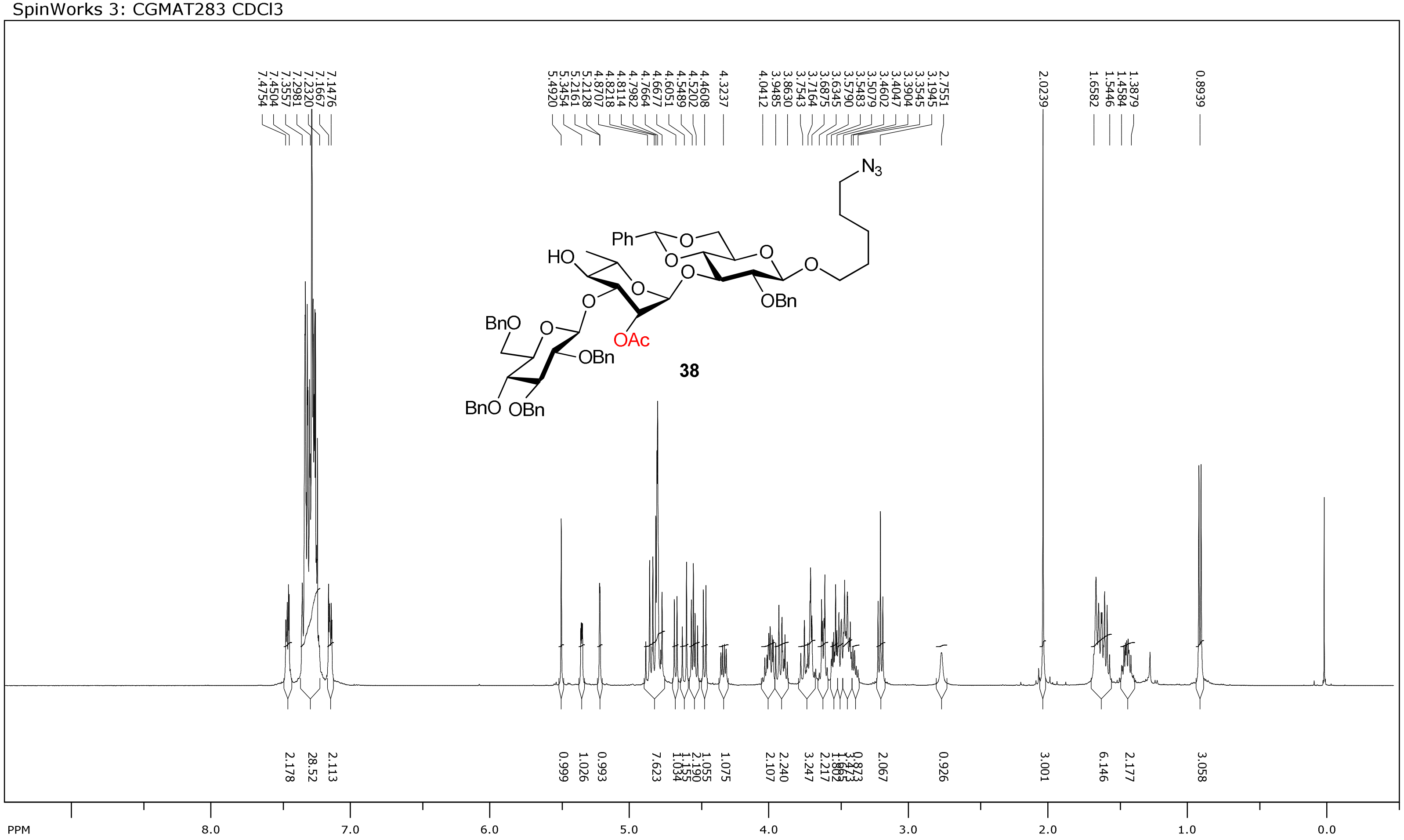

file: ...eau dossier\RMN LPS\CGMAT283\1\fid   expt: <zg30>
transmitter freq.: 400.132001 MHz
time domain size: 32768 points
width: 4807.69 Hz = 12.0153 ppm = 0.146719 Hz/pt
number of scans: 32

freq. of 0 ppm: 400.130016 MHz
processed size: 32768 complex points
LB: 0.300   GF: 0.0000
Hz/cm: 160.052   ppm/cm: 0.40000

Supplementary Figure 128 │ ^{13}C NMR spectra ($CDCl_3$, 100 MHz) of compound 38.

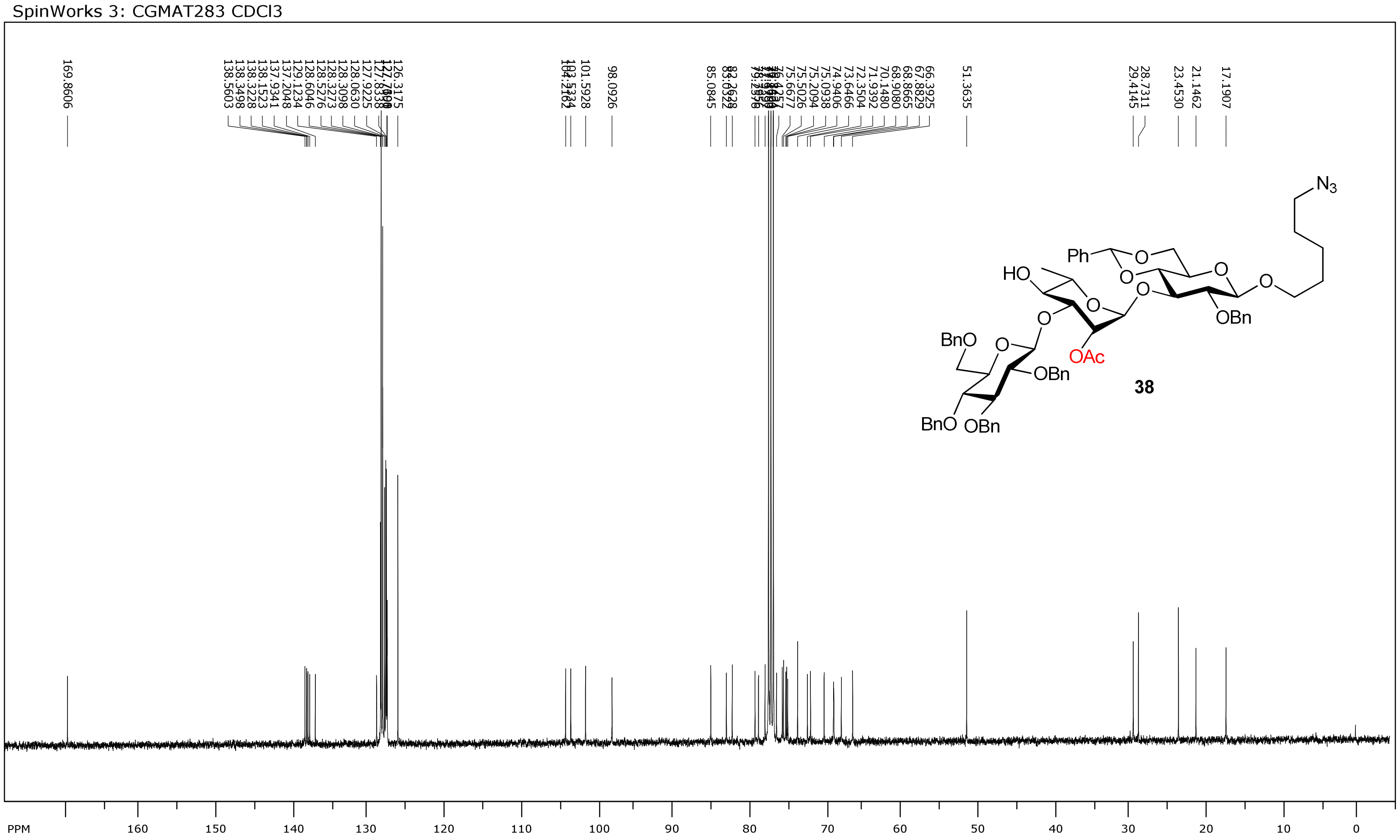

file: ...eau dossier\RMN LPS\CGMAT283\3\fid expt: <zgpg30>
transmitter freq.: 100.622830 MHz
time domain size: 65536 points
width: 24038.46 Hz = 238.8967 ppm = 0.366798 Hz/pt
number of scans: 1024

freq. of 0 ppm: 100.612757 MHz
processed size: 32768 complex points
LB: 1.000 GF: 0.0000
Hz/cm: 734.687 ppm/cm: 7.30140

Supplementary Figure 129 │ ^{1}H NMR spectra ($CDCl_3$, 400 MHz) of compound 39.

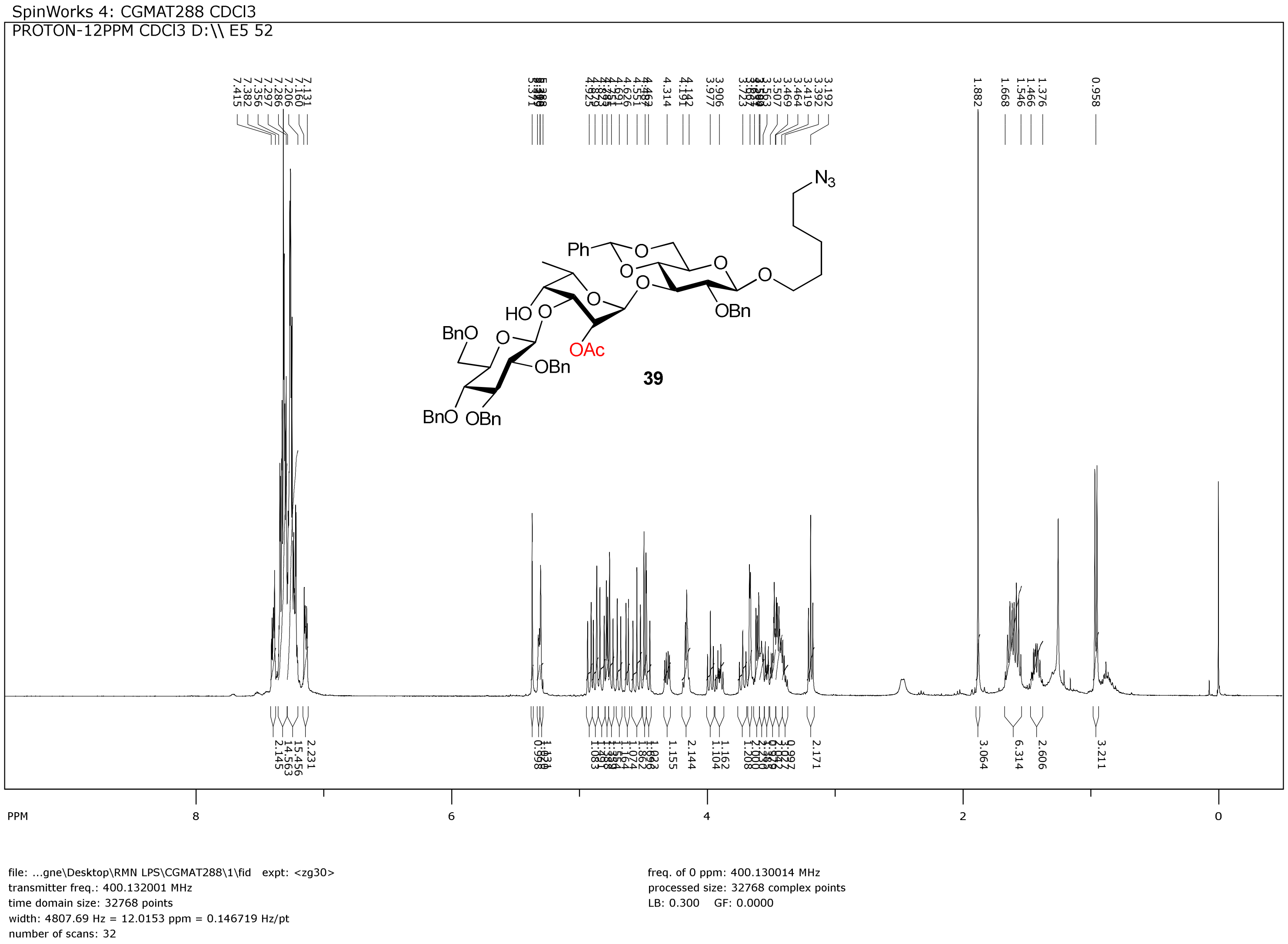

Supplementary Figure 130 | ^{13}C NMR spectra ($CDCl_3$, 100 MHz) of compound 39.

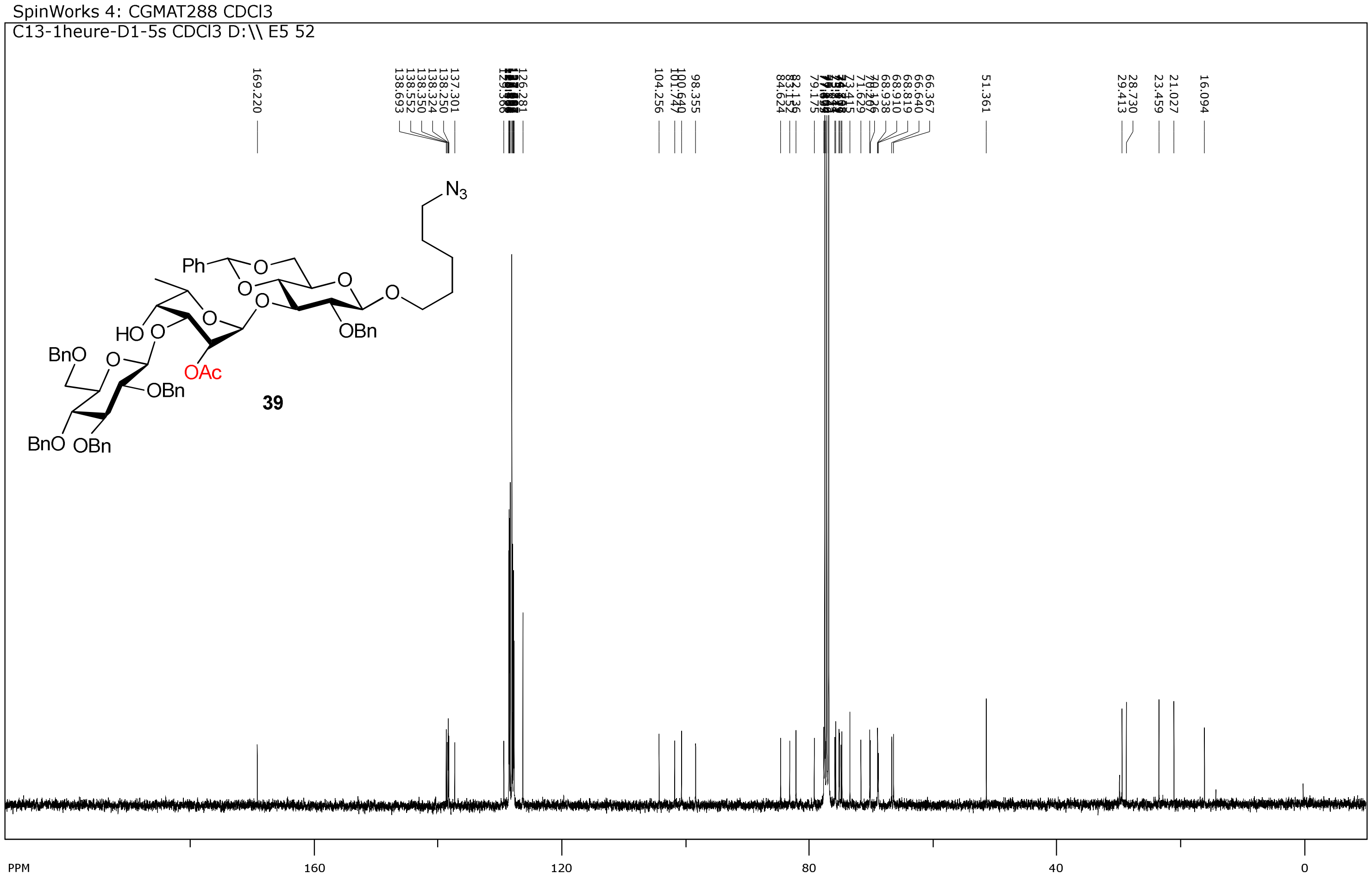

file: ...gne\Desktop\RMN LPS\CGMAT288\3\fid expt: <zgpg30>
transmitter freq.: 100.622830 MHz
time domain size: 65536 points
width: 24038.46 Hz = 238.8967 ppm = 0.366798 Hz/pt
number of scans: 1024

freq. of 0 ppm: 100.612756 MHz
processed size: 32768 complex points
LB: 1.000 GF: 0.0000

Supplementary Figure 131 │ ^{1}H NMR spectra ($CDCl_3$, 400 MHz) of compound 40.

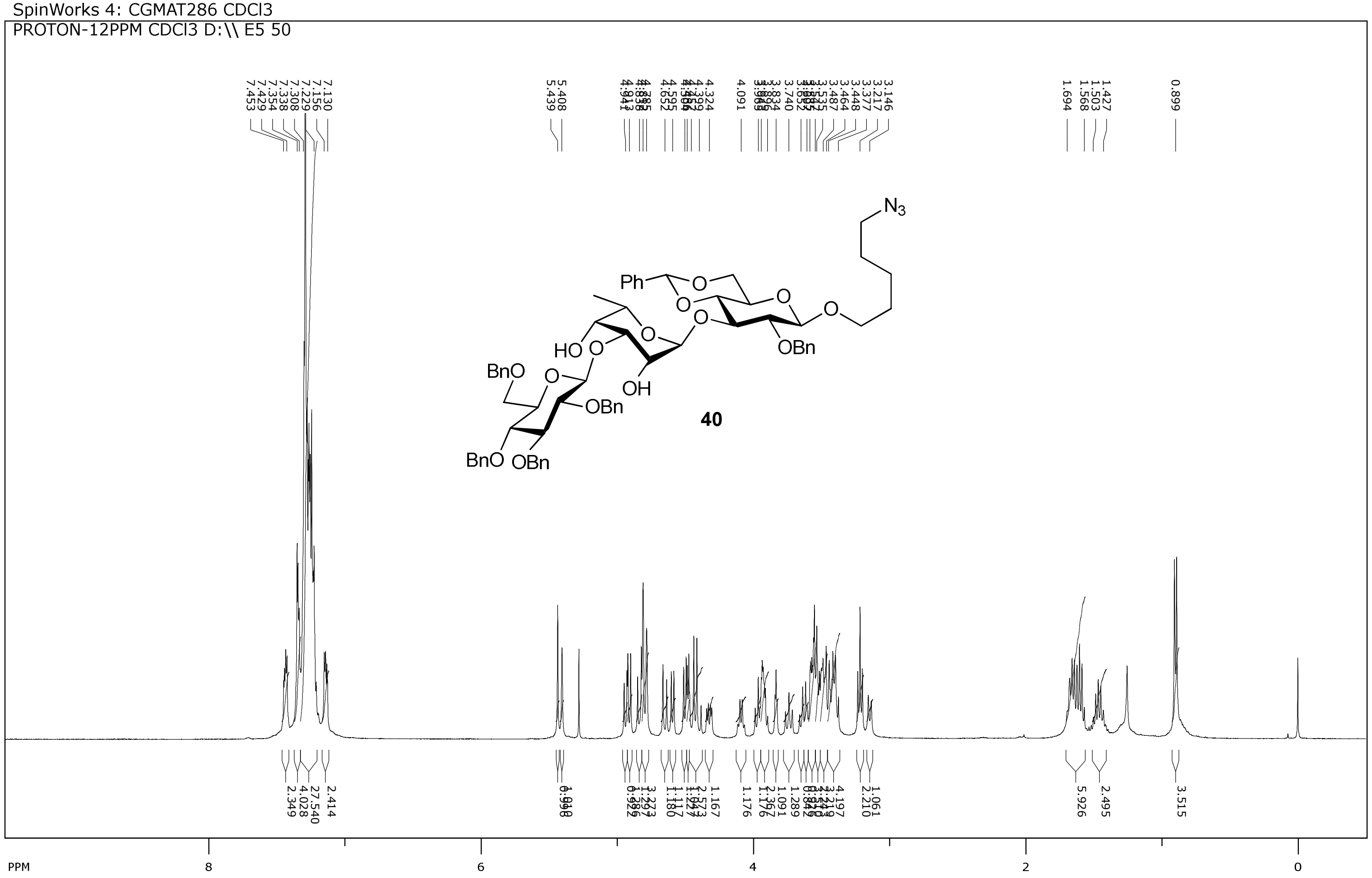

file: ...gne\Desktop\RMN LPS\CGMAT286\1\fid expt: <zg30>
transmitter freq.: 400.132001 MHz
time domain size: 32768 points
width: 4807.69 Hz = 12.0153 ppm = 0.146719 Hz/pt
number of scans: 32

freq. of 0 ppm: 400.130013 MHz
processed size: 32768 complex points
LB: 0.300 GF: 0.0000

Supplementary Figure 132 │ ^{13}C NMR spectra ($CDCl_3$, 100 MHz) of compound 40.

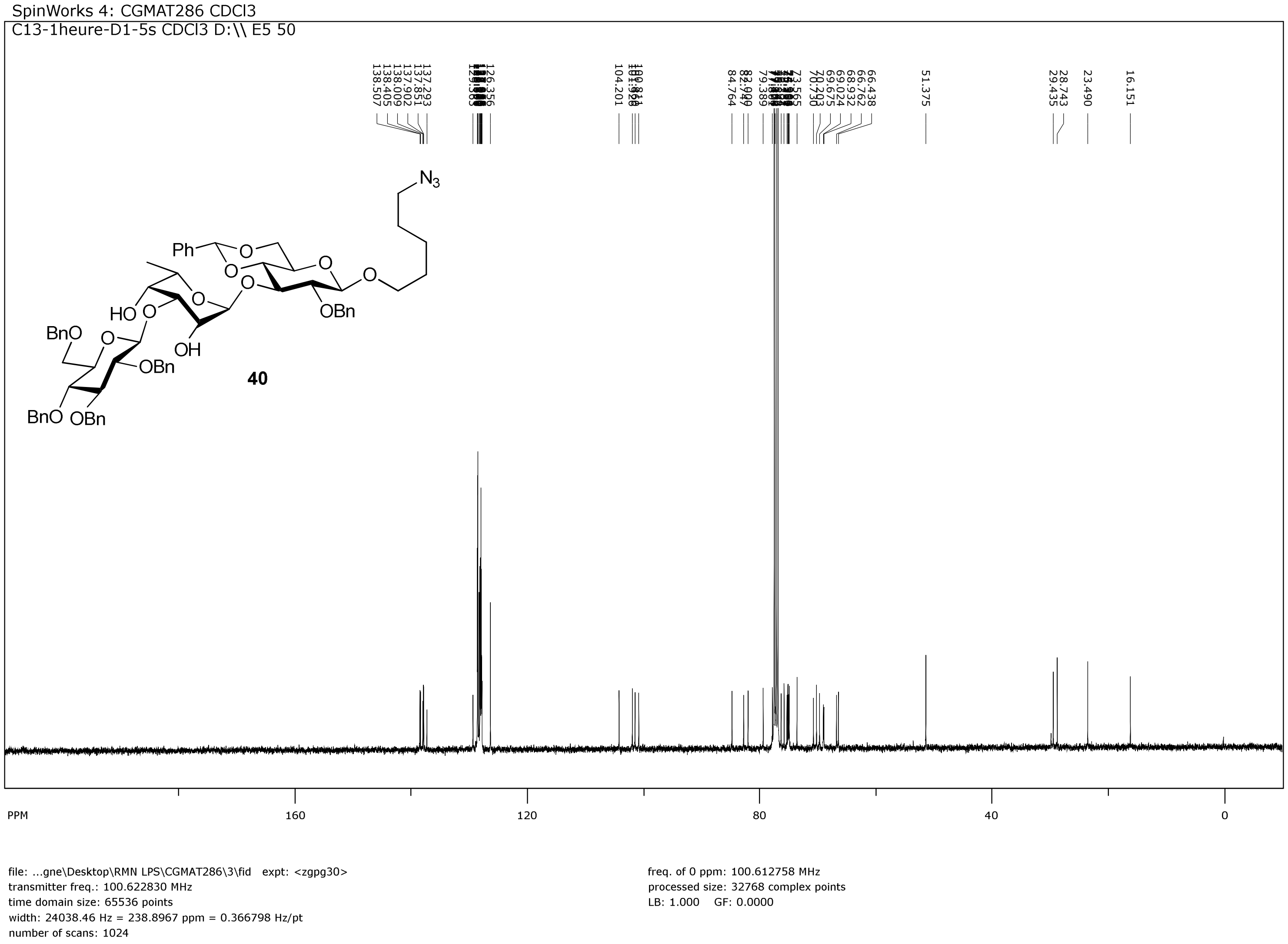

Supplementary Figure 133 │ ^{1}H NMR spectra ($CDCl_3$, 400 MHz) of compound 41.

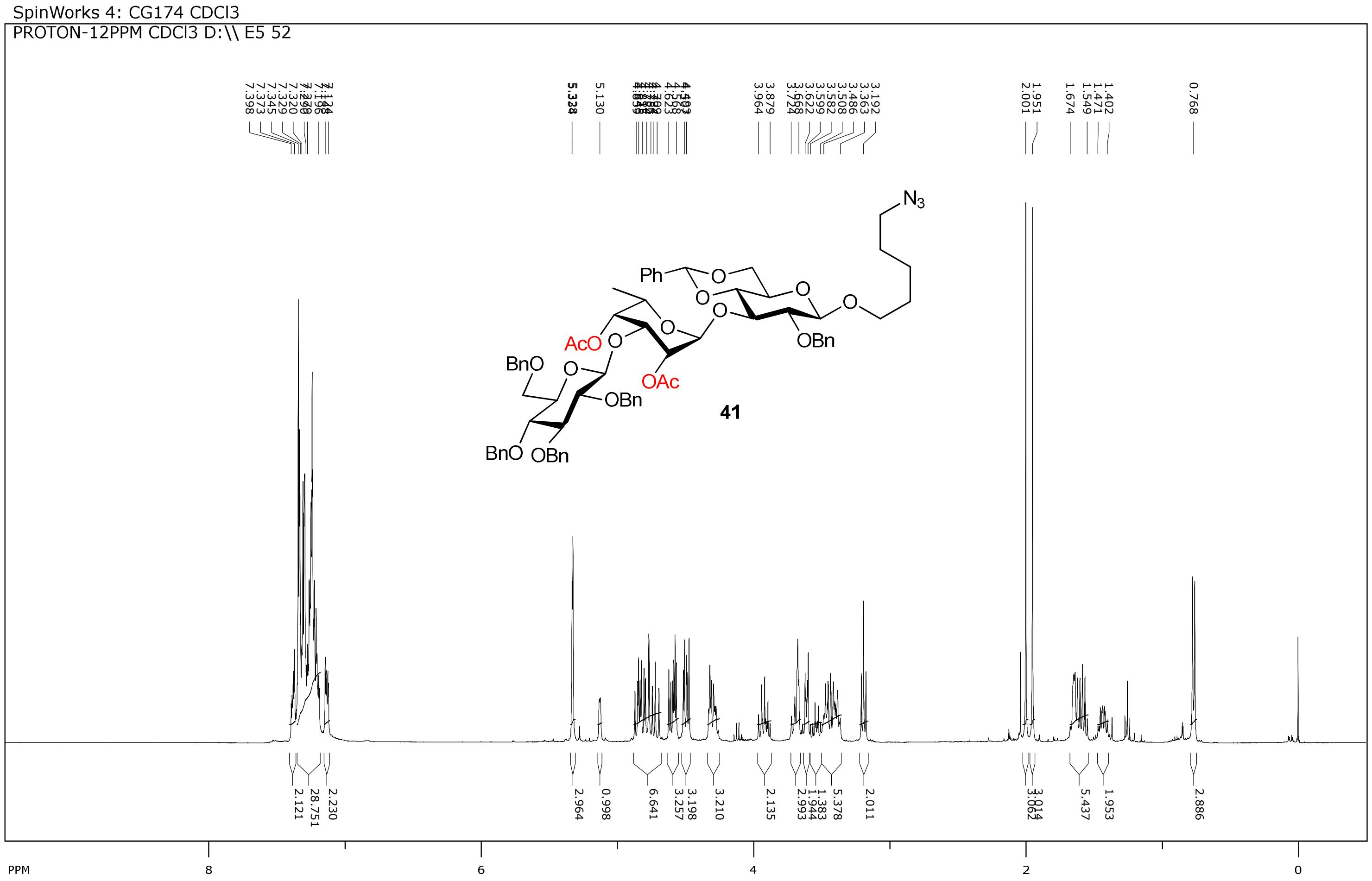

file: ...amigne\Desktop\RMN LPS\CG174\2\fid expt: <zg30>
transmitter freq.: 400.132001 MHz
time domain size: 32768 points
width: 4807.69 Hz = 12.0153 ppm = 0.146719 Hz/pt
number of scans: 32

freq. of 0 ppm: 400.130016 MHz
processed size: 32768 complex points
LB: 0.300 GF: 0.0000

Supplementary Figure 134 | ^{13}C NMR spectra ($CDCl_3$, 100 MHz) of compound 41.

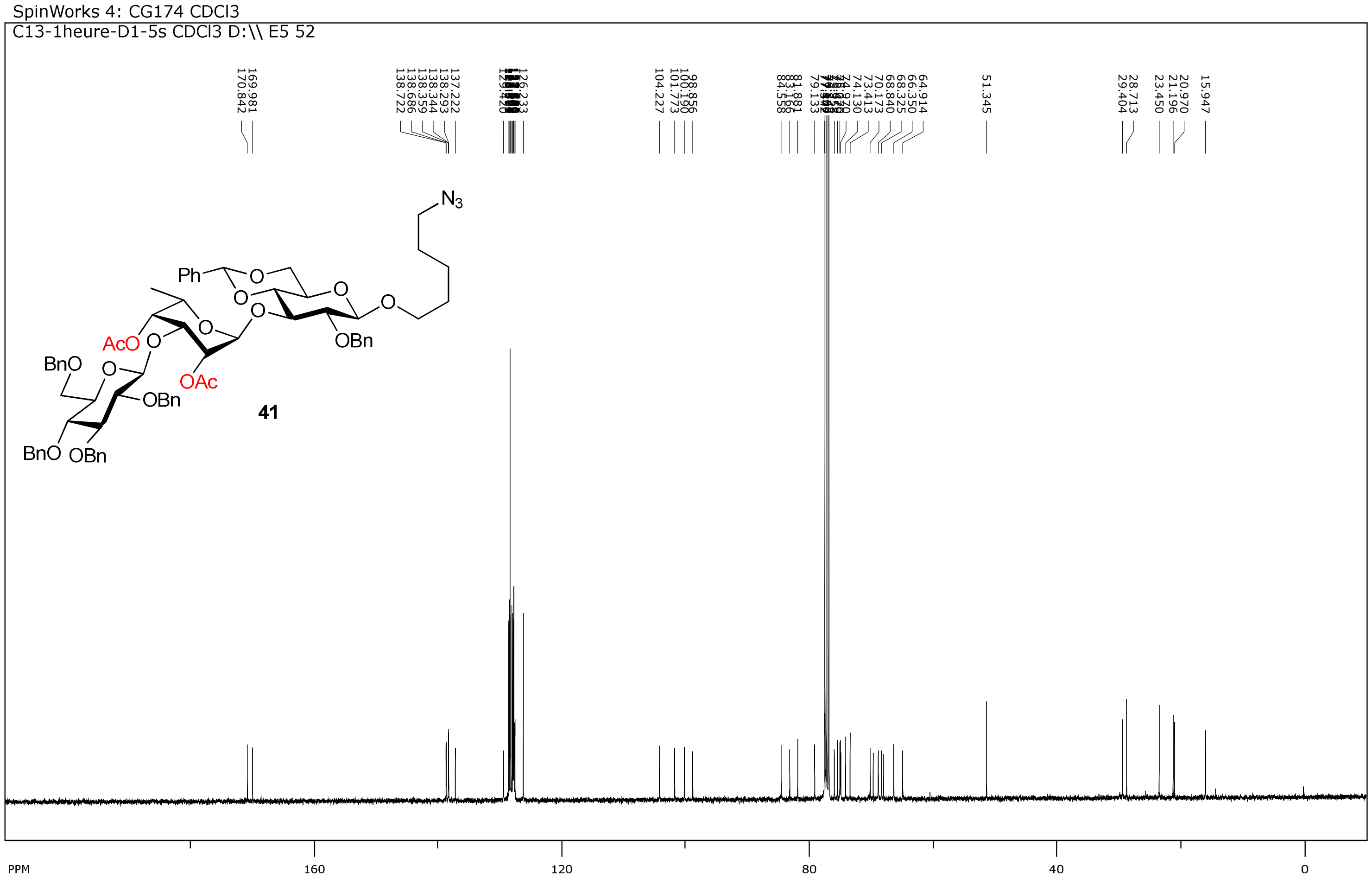

file: ...amigne\Desktop\RMN LPS\CG174\4\fid expt: <zgpg30>
transmitter freq.: 100.622830 MHz
time domain size: 65536 points
width: 24038.46 Hz = 238.8967 ppm = 0.366798 Hz/pt
number of scans: 1024

freq. of 0 ppm: 100.612759 MHz
processed size: 32768 complex points
LB: 1.000 GF: 0.0000

Supplementary Figure 135 │ ^{1}H NMR spectra ($CDCl_3$, 400 MHz) of compound S36.

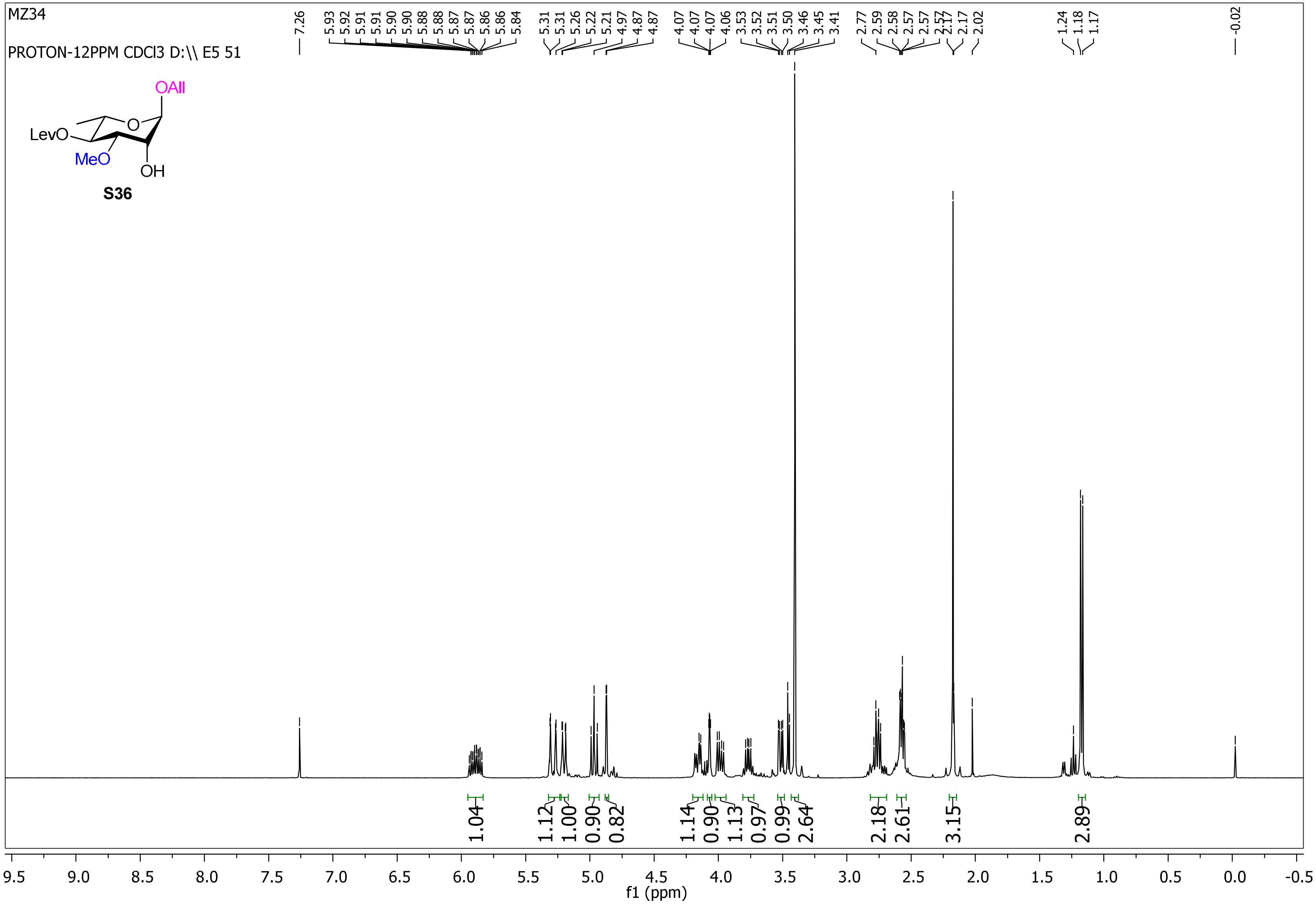

Supplementary Figure 136 │ ^{13}C NMR spectra ($CDCl_3$, 100 MHz) of compound S36.

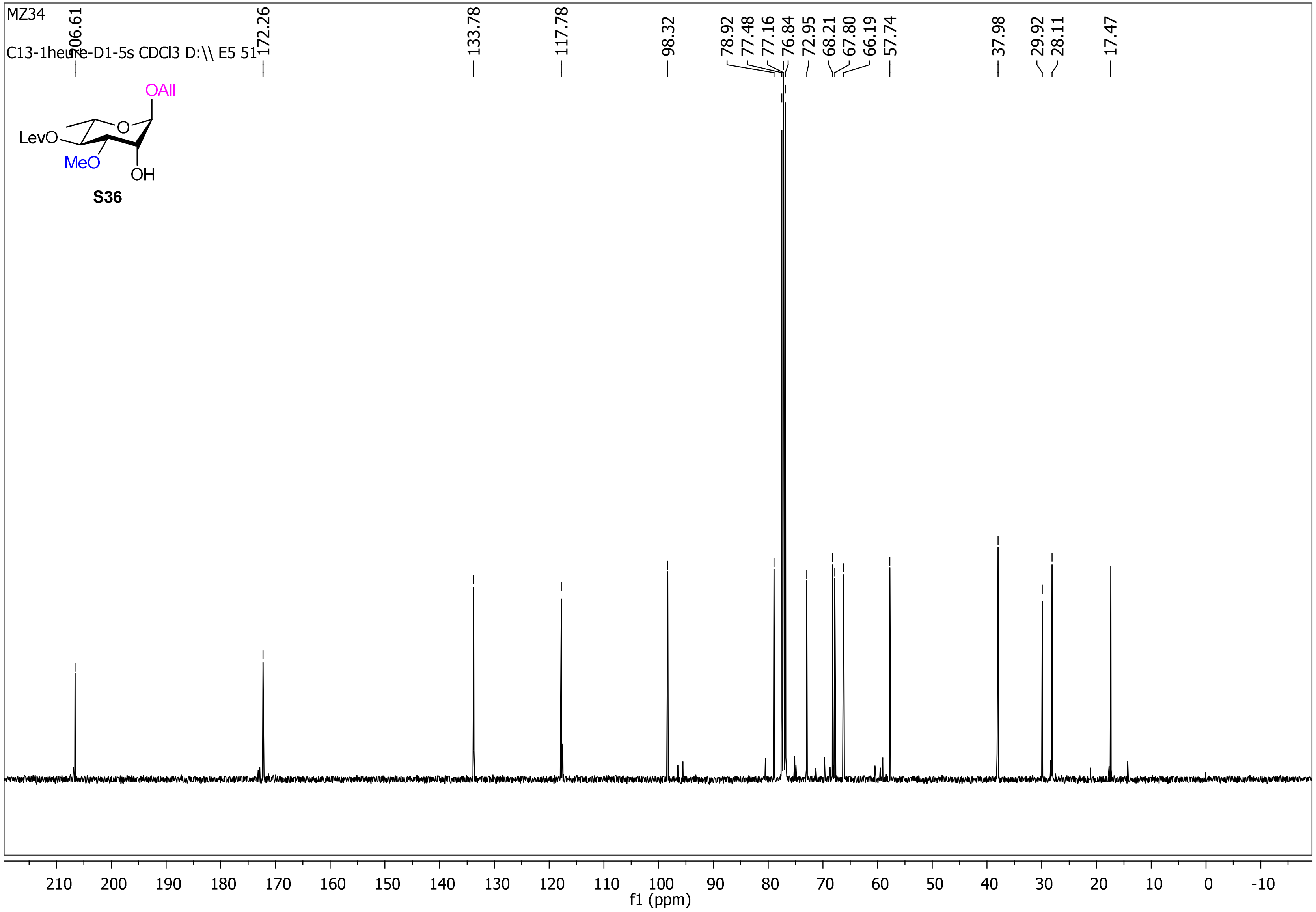

Supplementary Figure 137 | ^{1}H NMR spectra ($CDCl_3$, 400 MHz) of compound S37.

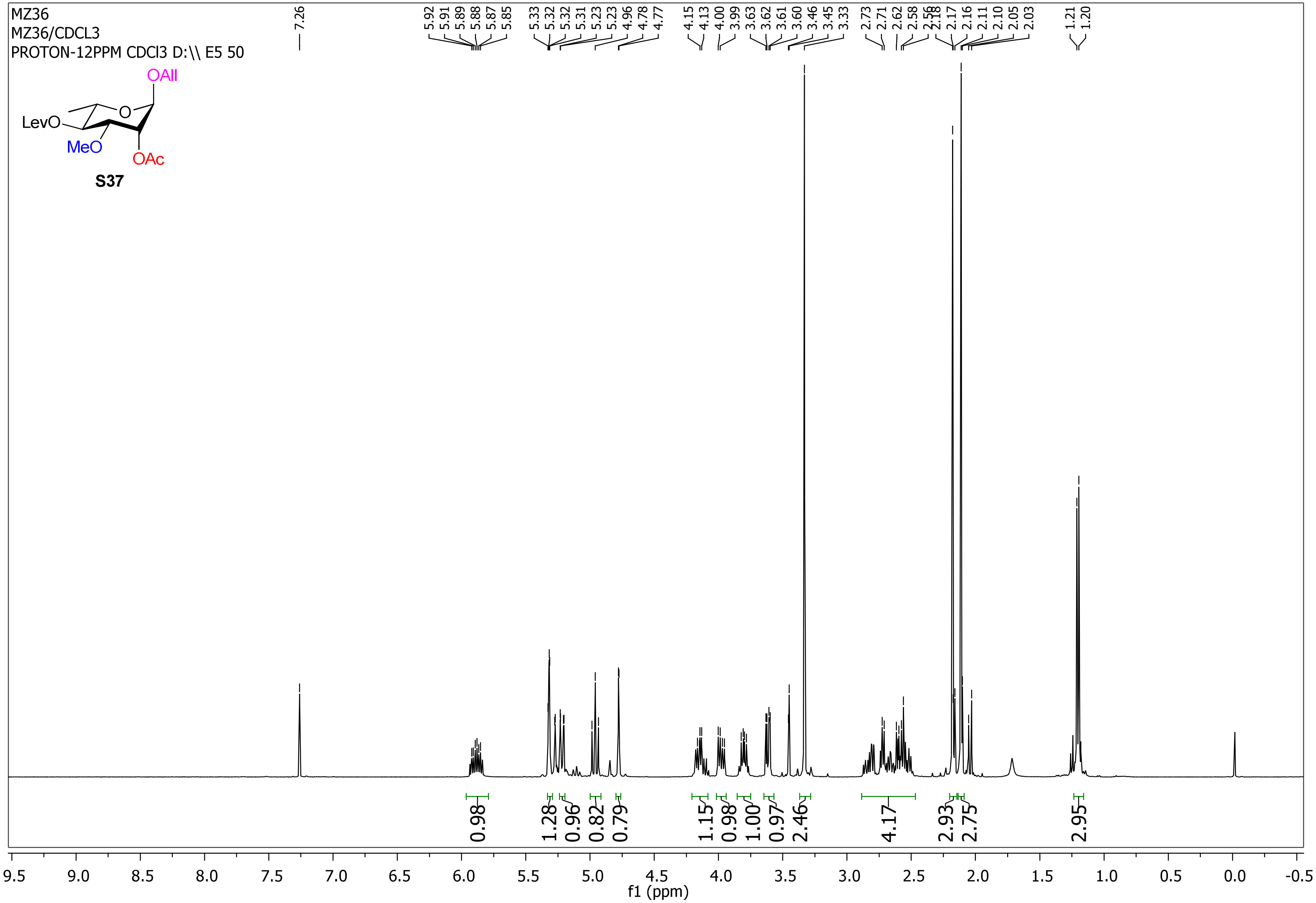

Supplementary Figure 138 | ^{13}C NMR spectra ($CDCl_3$, 100 MHz) of compound S37.

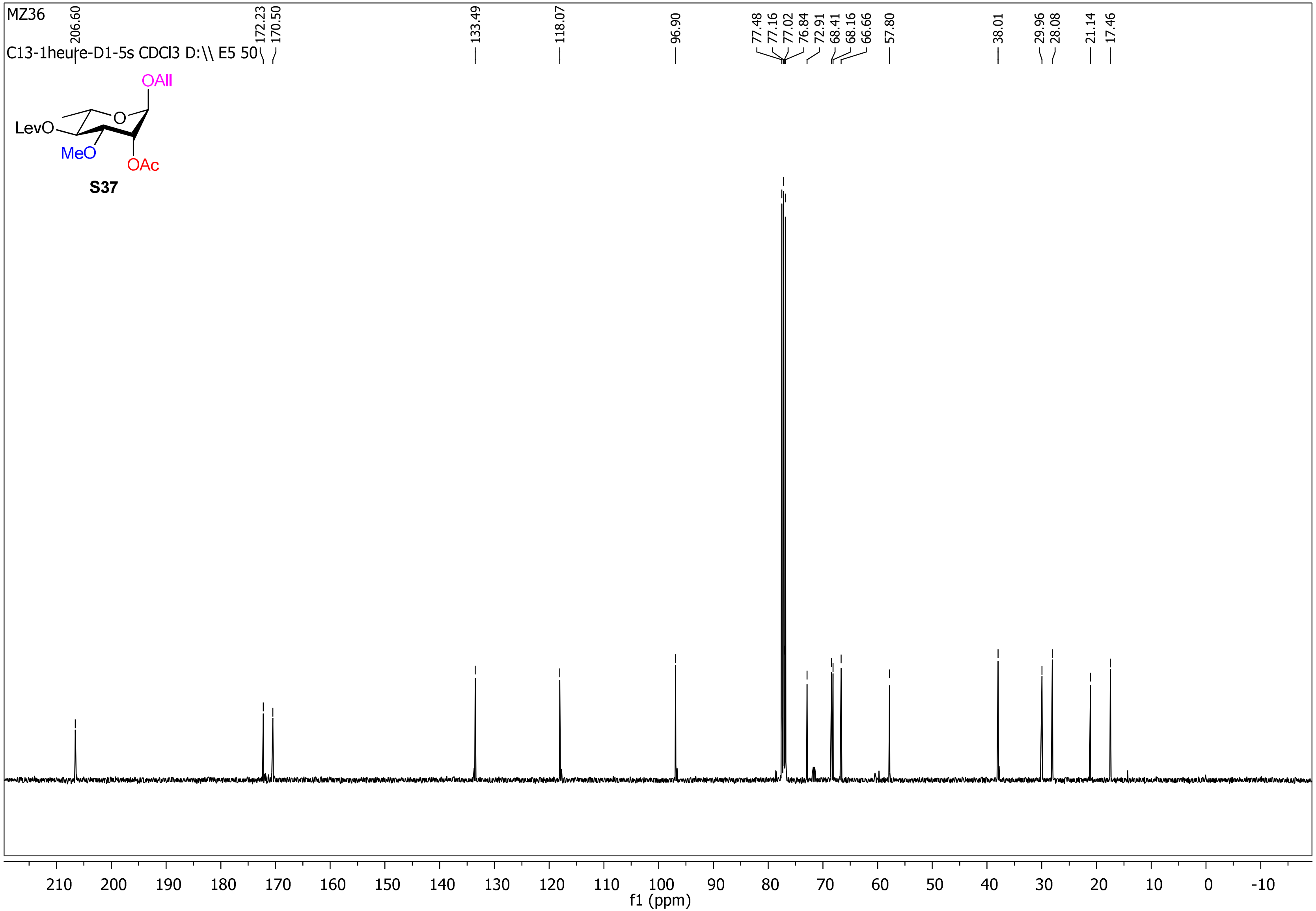

Supplementary Figure 139 | ^{1}H NMR spectra ($CDCl_3$, 400 MHz) of compound S38.

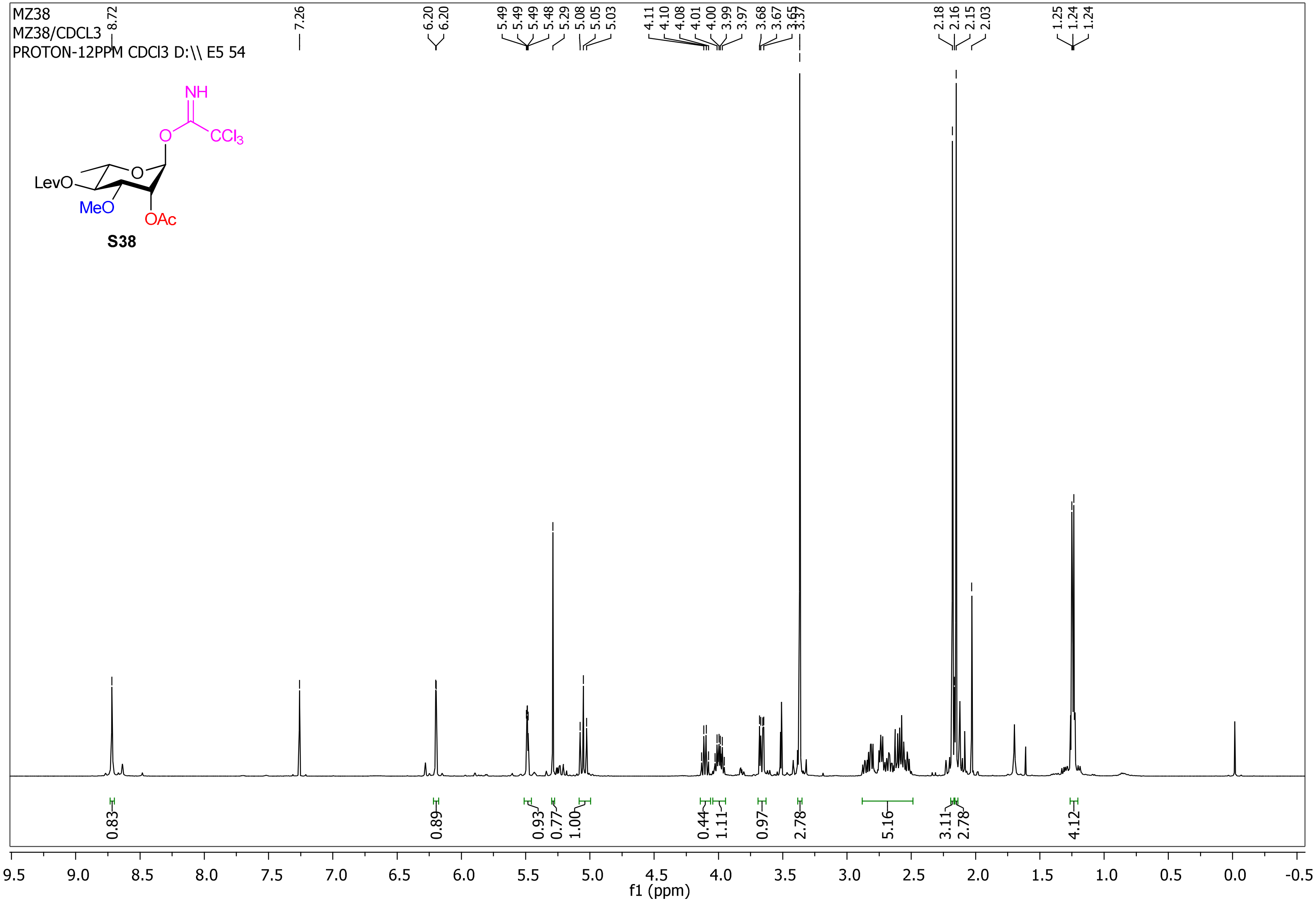

Supplementary Figure 140 | ^{13}C NMR spectra ($CDCl_3$, 100 MHz) of compound S38.

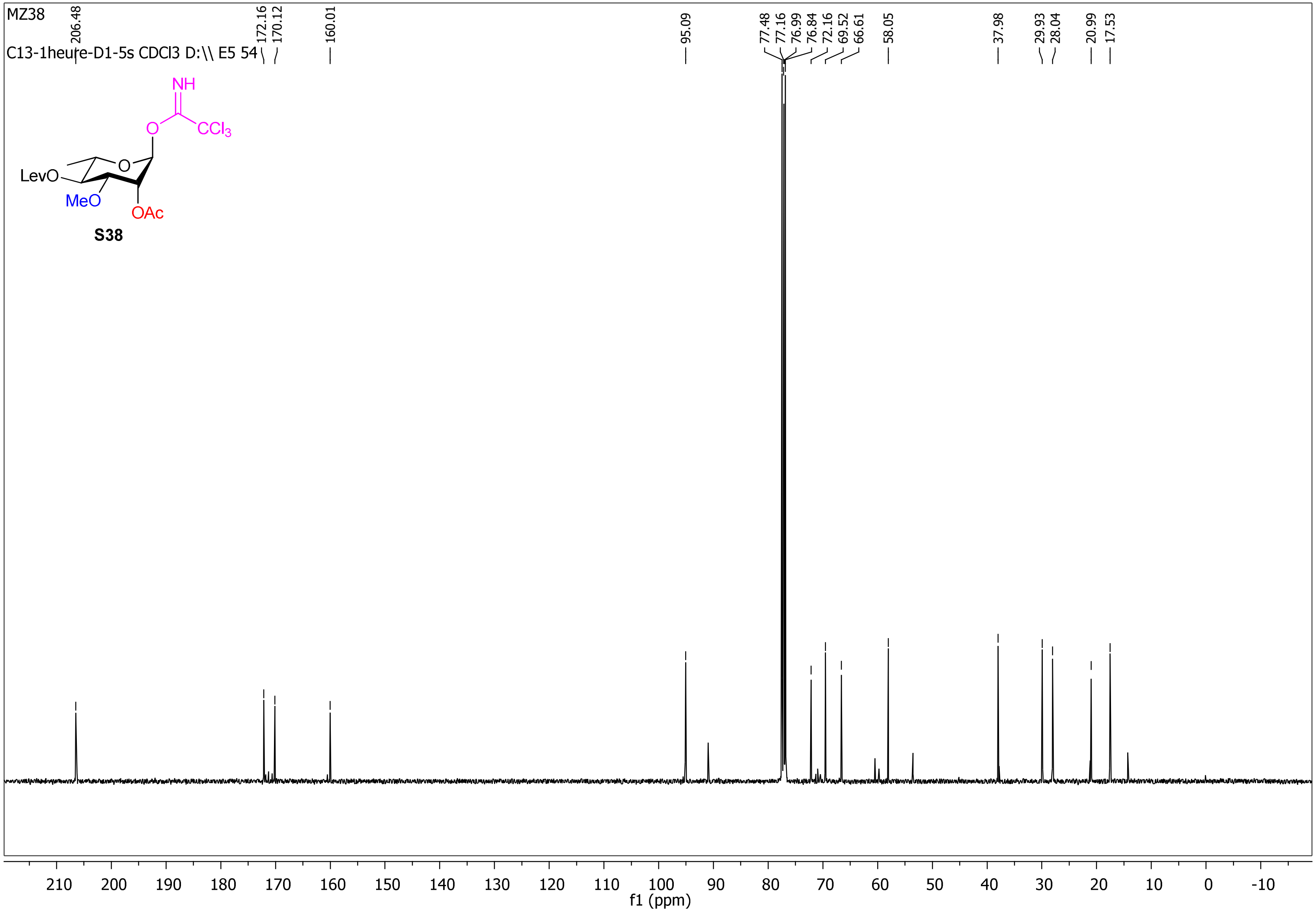

Supplementary Figure 141 │ ^{1}H NMR spectra ($CDCl_3$, 400 MHz) of compound S39.

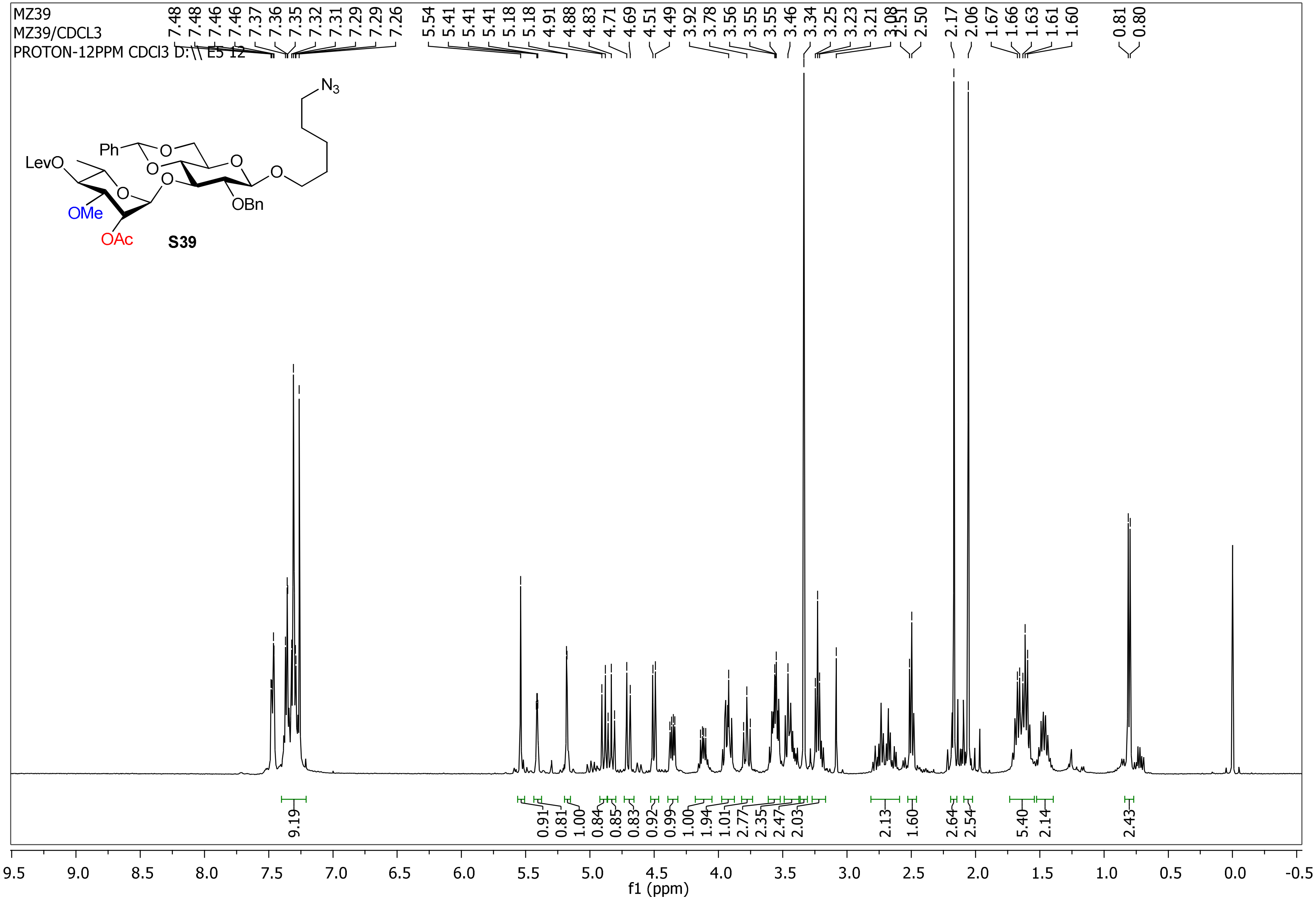

Supplementary Figure 142 │ ^{13}C NMR spectra ($CDCl_3$, 100 MHz) of compound S39.

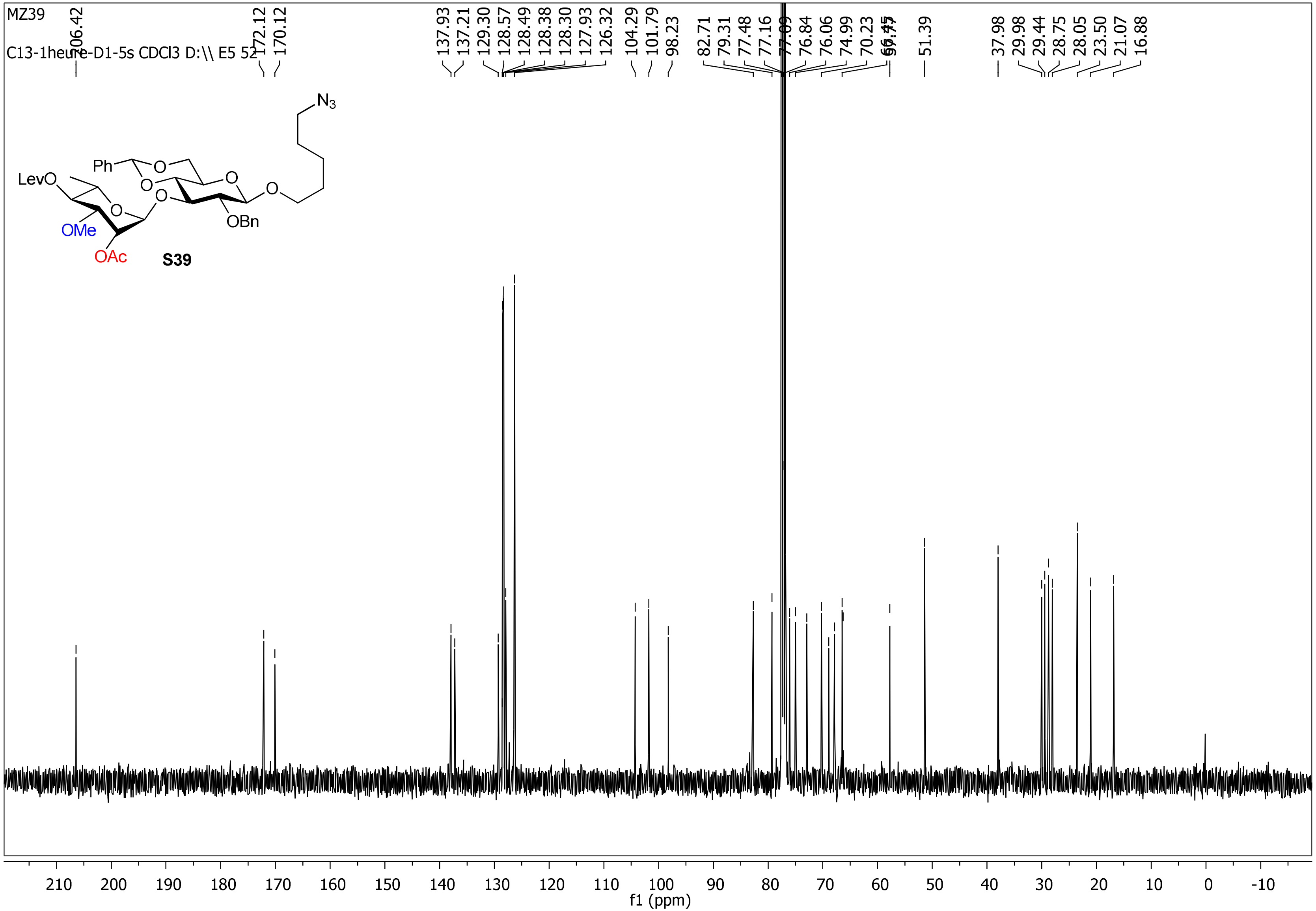

Supplementary Figure 143 │ ^{1}H NMR spectra (CDCl3, 400 MHz) of compound S40.

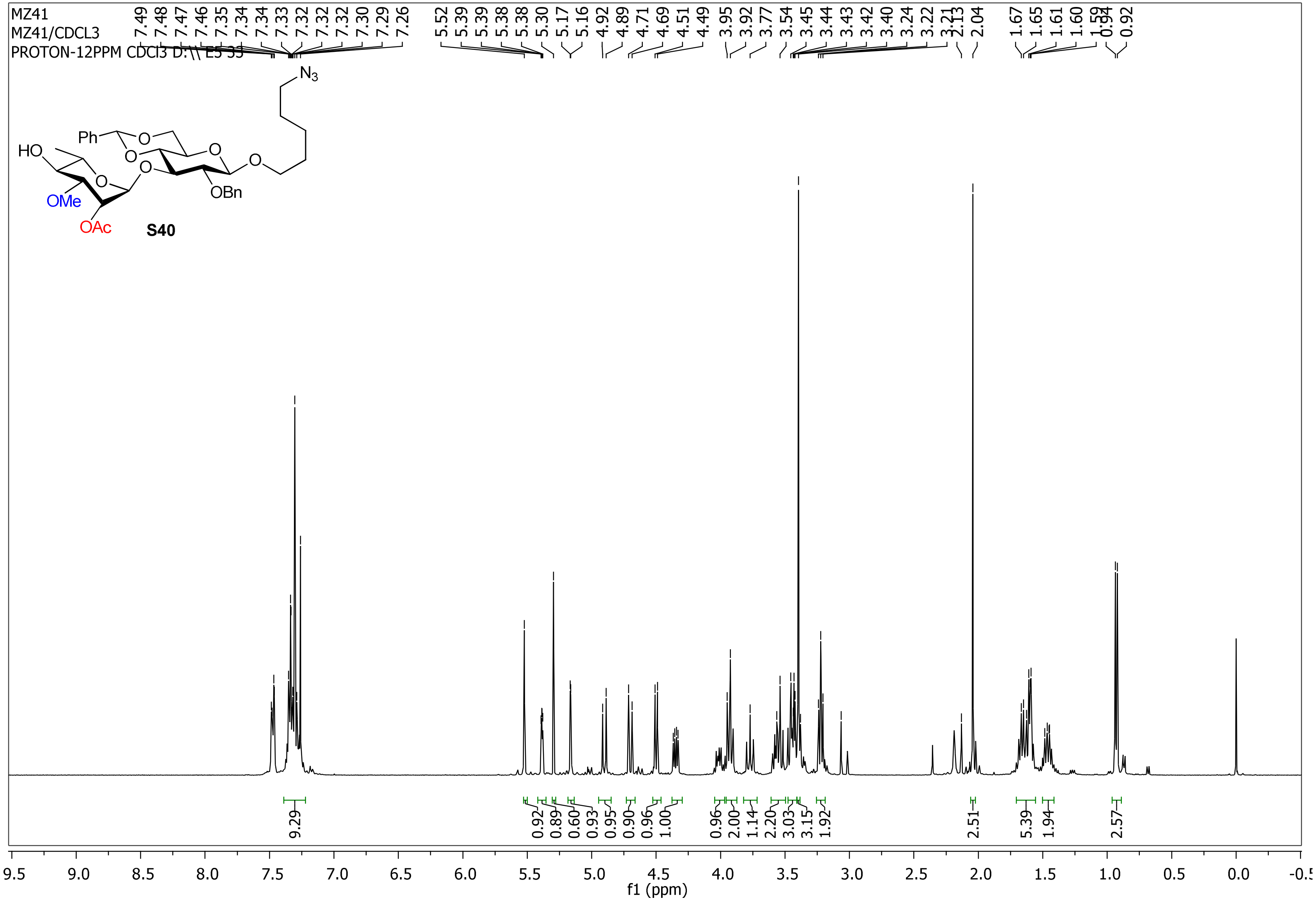

Supplementary Figure 144 | ^{13}C NMR spectra ($CDCl_3$, 100 MHz) of compound S40.

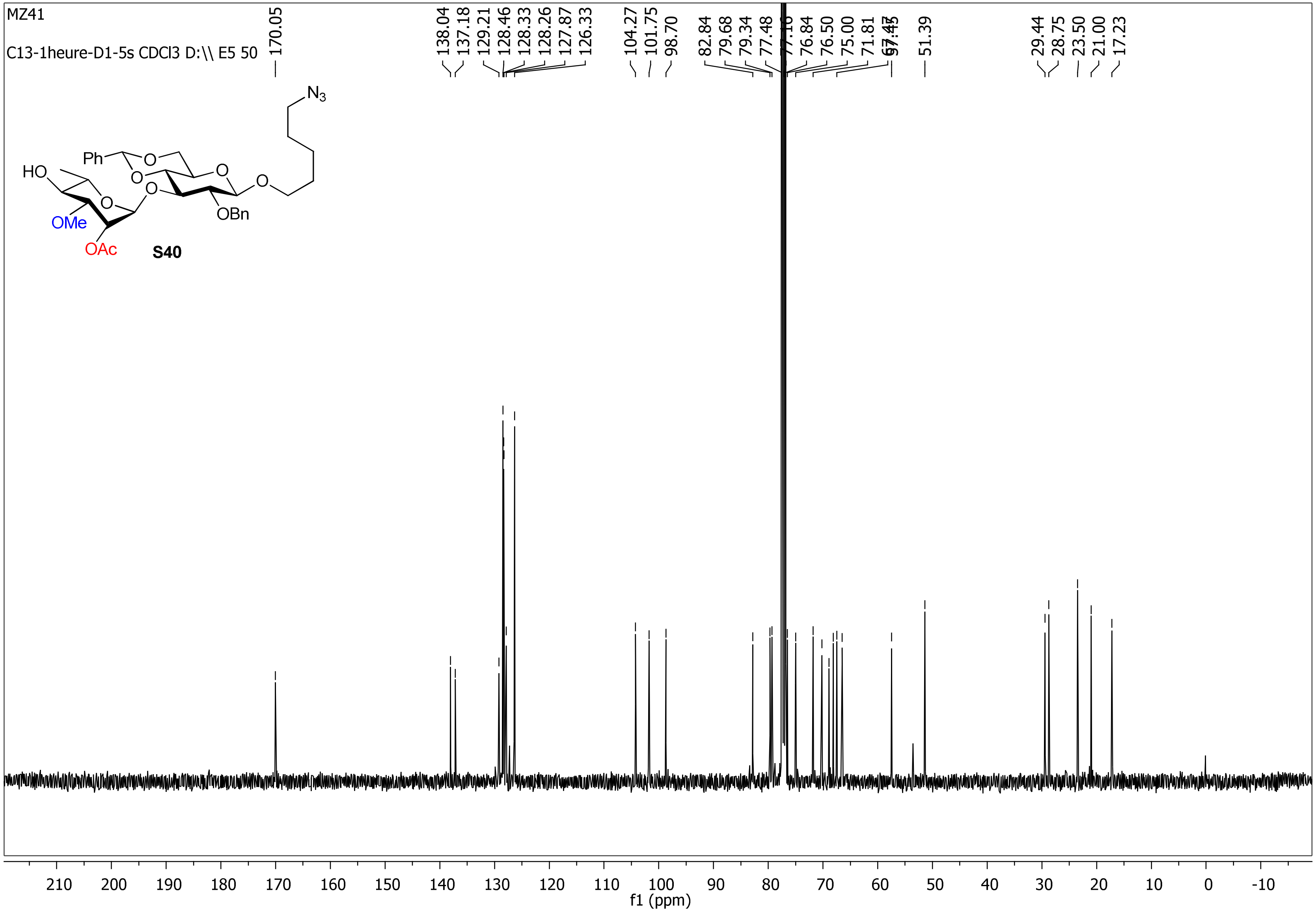

Supplementary Figure 145 │ ^{1}H NMR spectra (CDCl$_3$, 400 MHz) of compound S41.

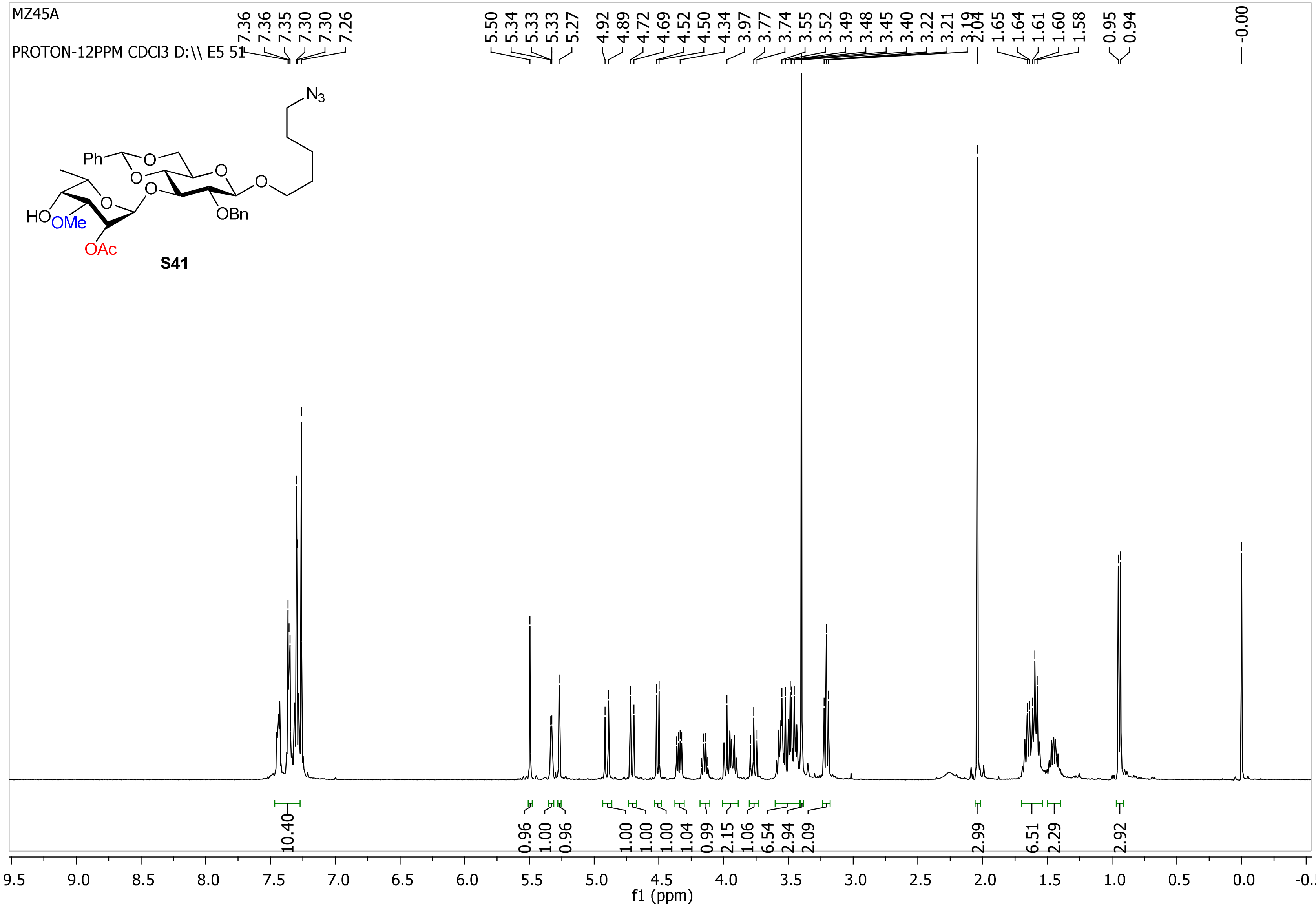

Supplementary Figure 146 | ^{13}C NMR spectra ($CDCl_3$, 100 MHz) of compound S41.

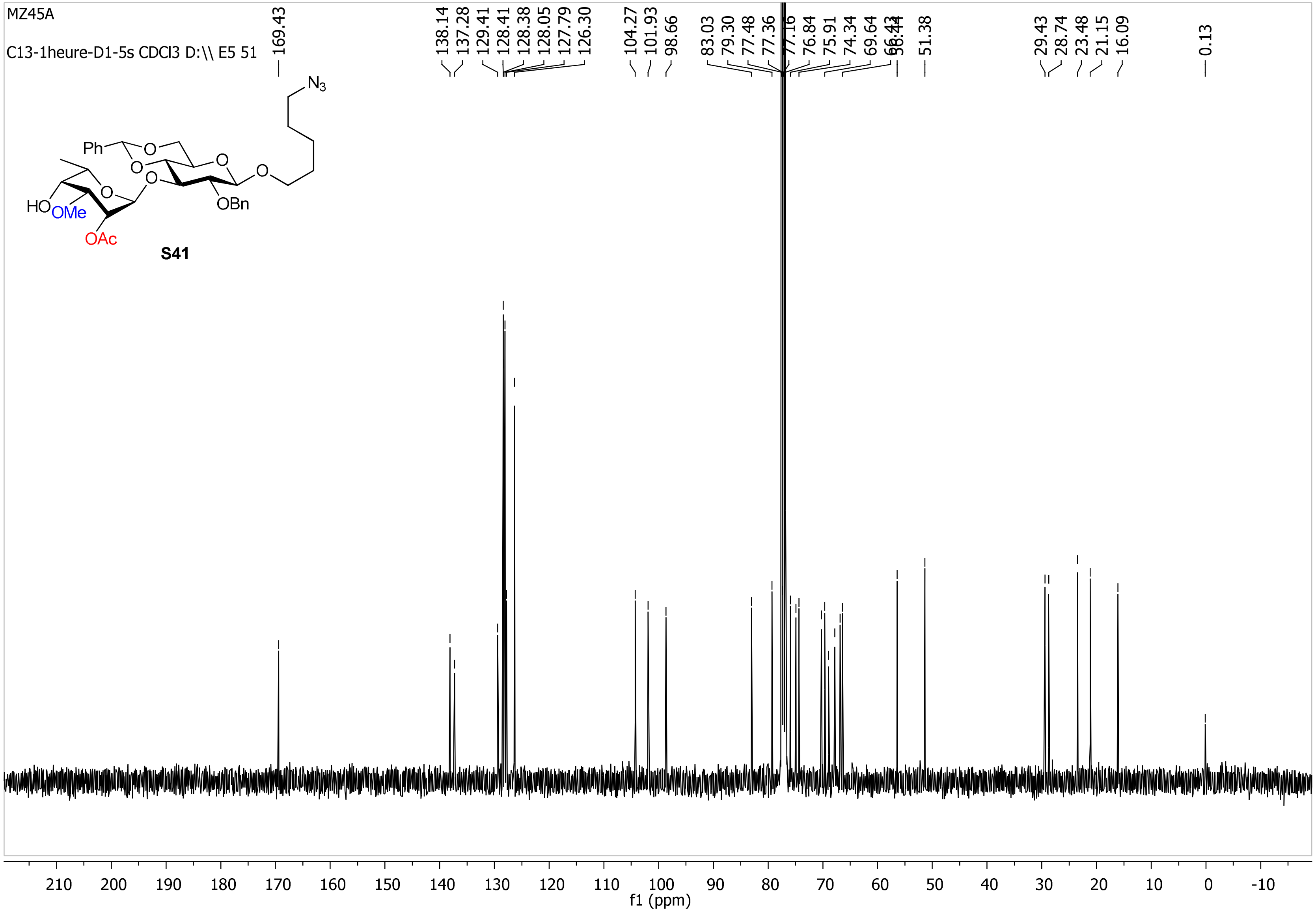

Supplementary Figure 147 │ ^{1}H NMR spectra (D_2O + acetone, 400 MHz) of compound 1.

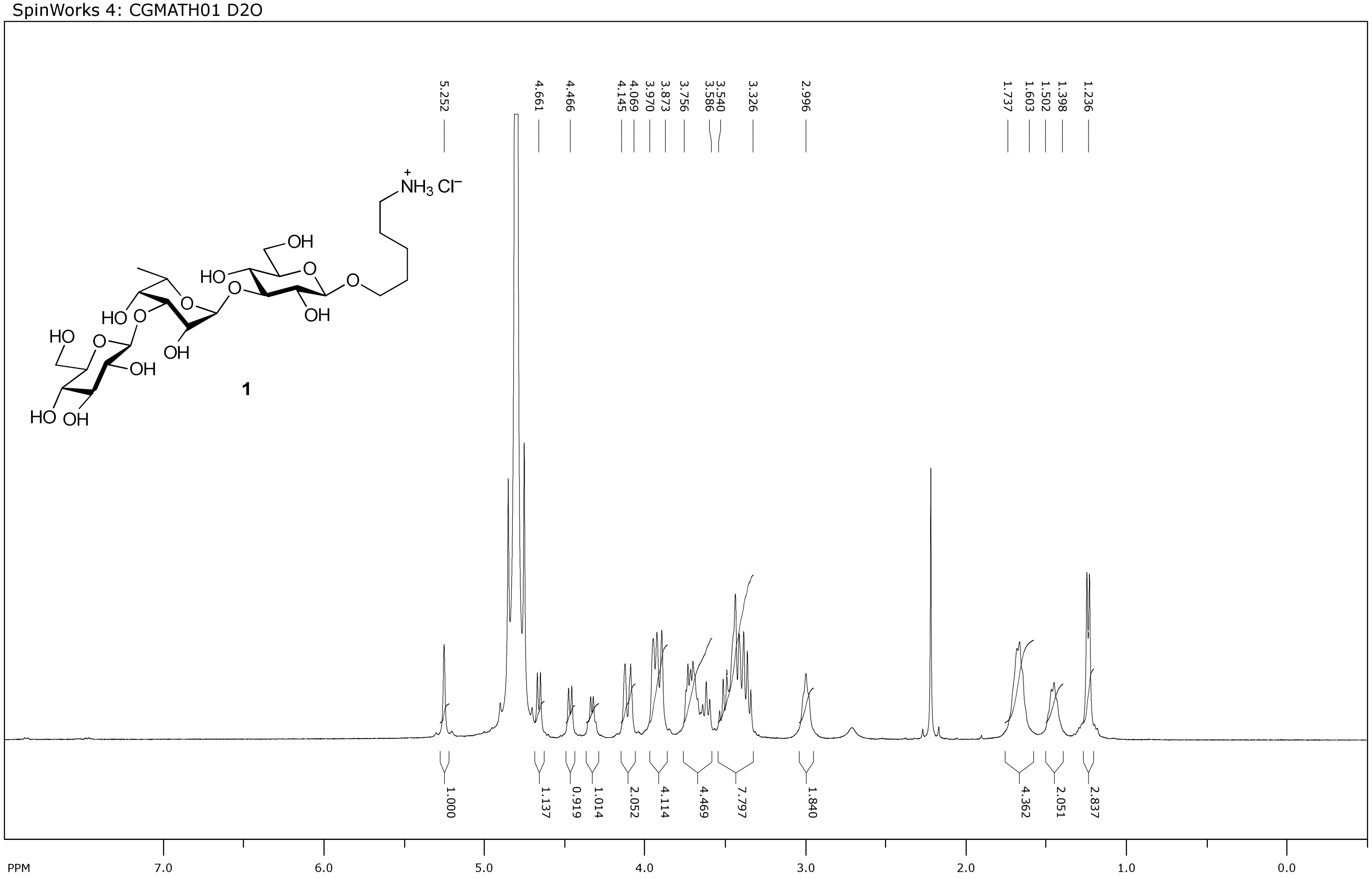

Supplementary Figure 148 │ ^{13}C NMR spectra (D_2O + acetone, 100 MHz) of compound 1.

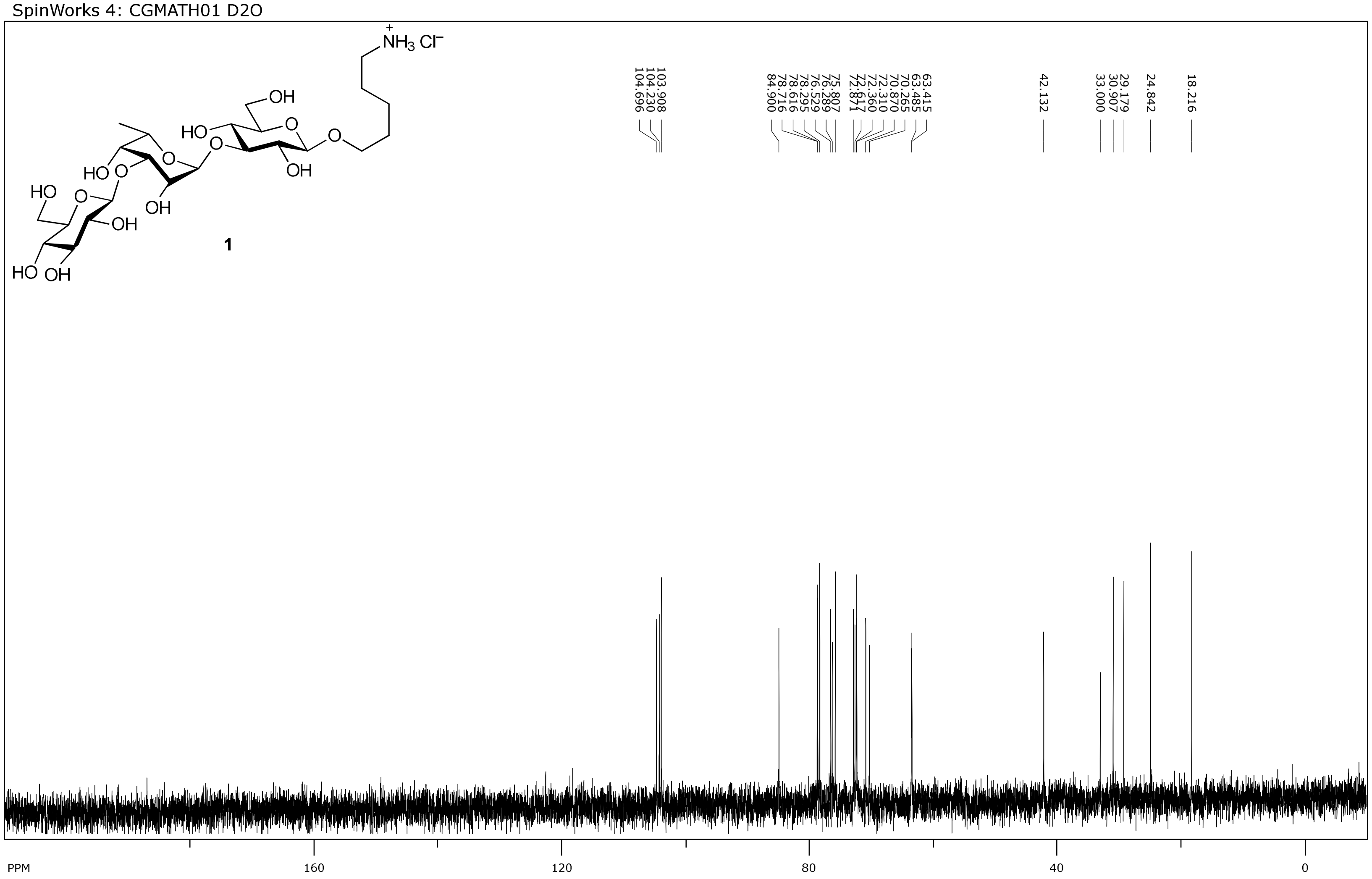

Supplementary Figure 149 | ^{1}H NMR spectra (D_2O + acetone, 400 MHz) of compound 2.

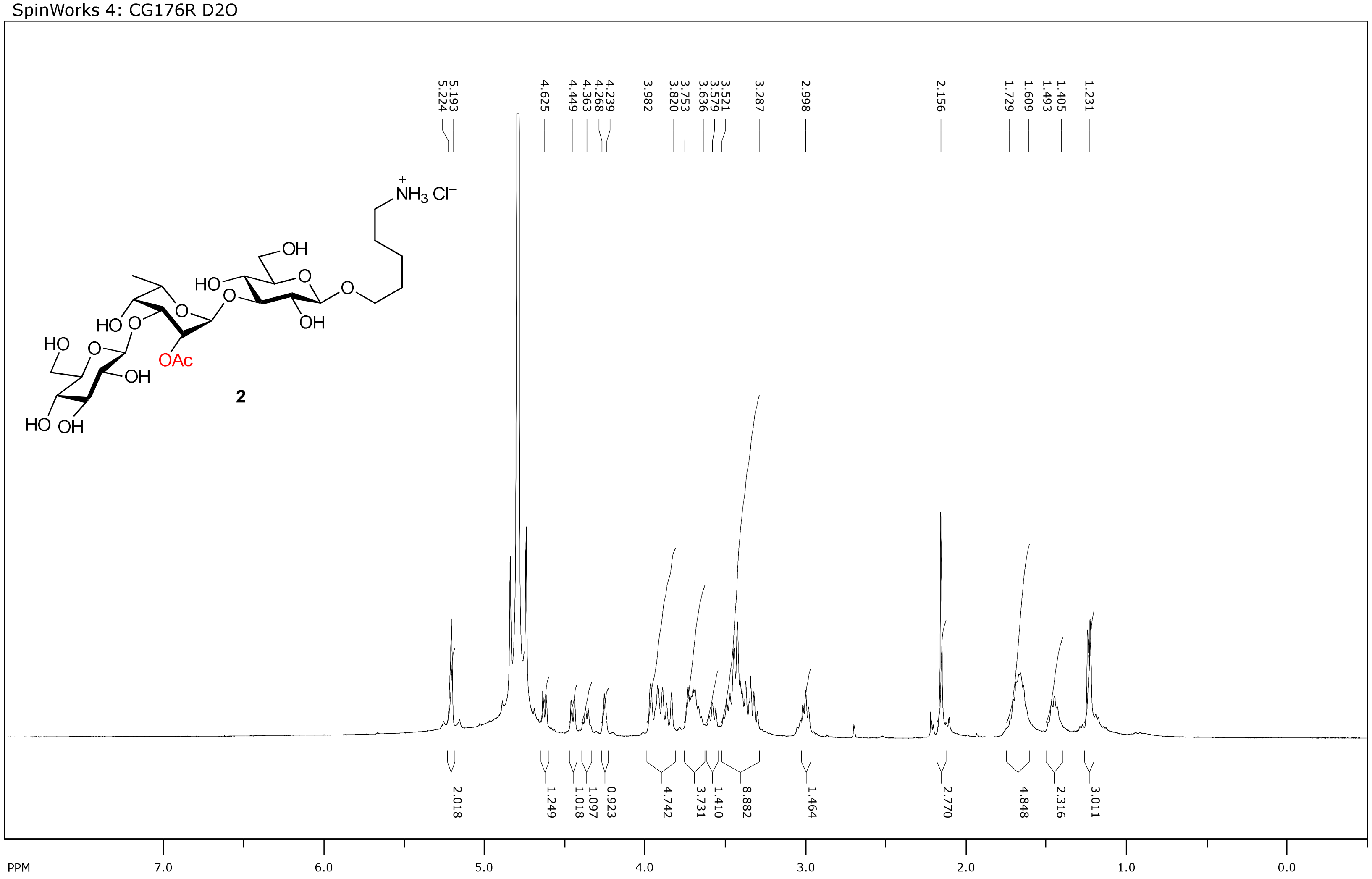

Supplementary Figure 150 | ^{13}C NMR spectra (D_2O + acetone, 100 MHz) of compound 2.

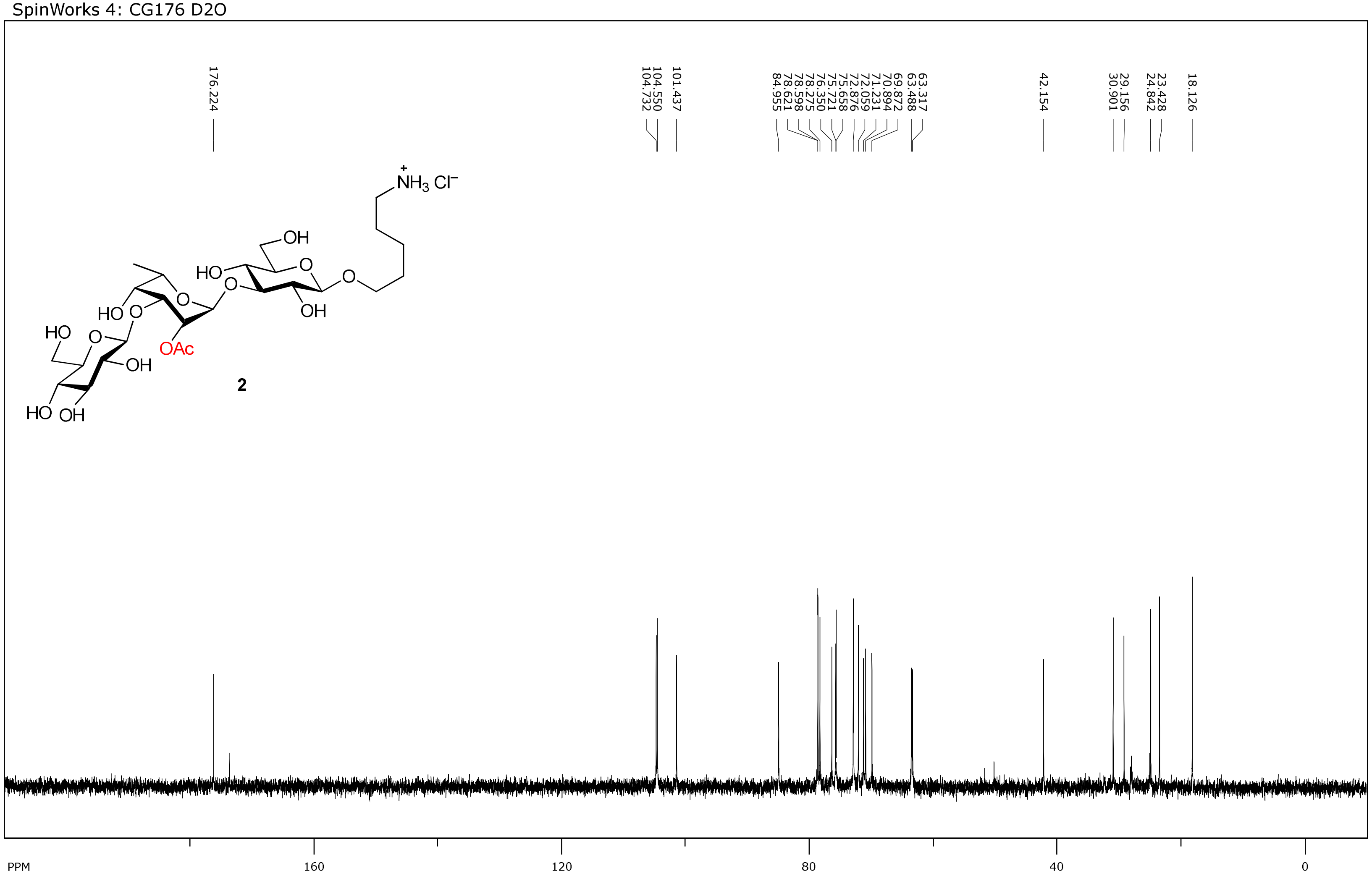

Supplementary Figure 151 | ^{1}H NMR spectra (D_2O + acetone, 400 MHz) of compound 3.

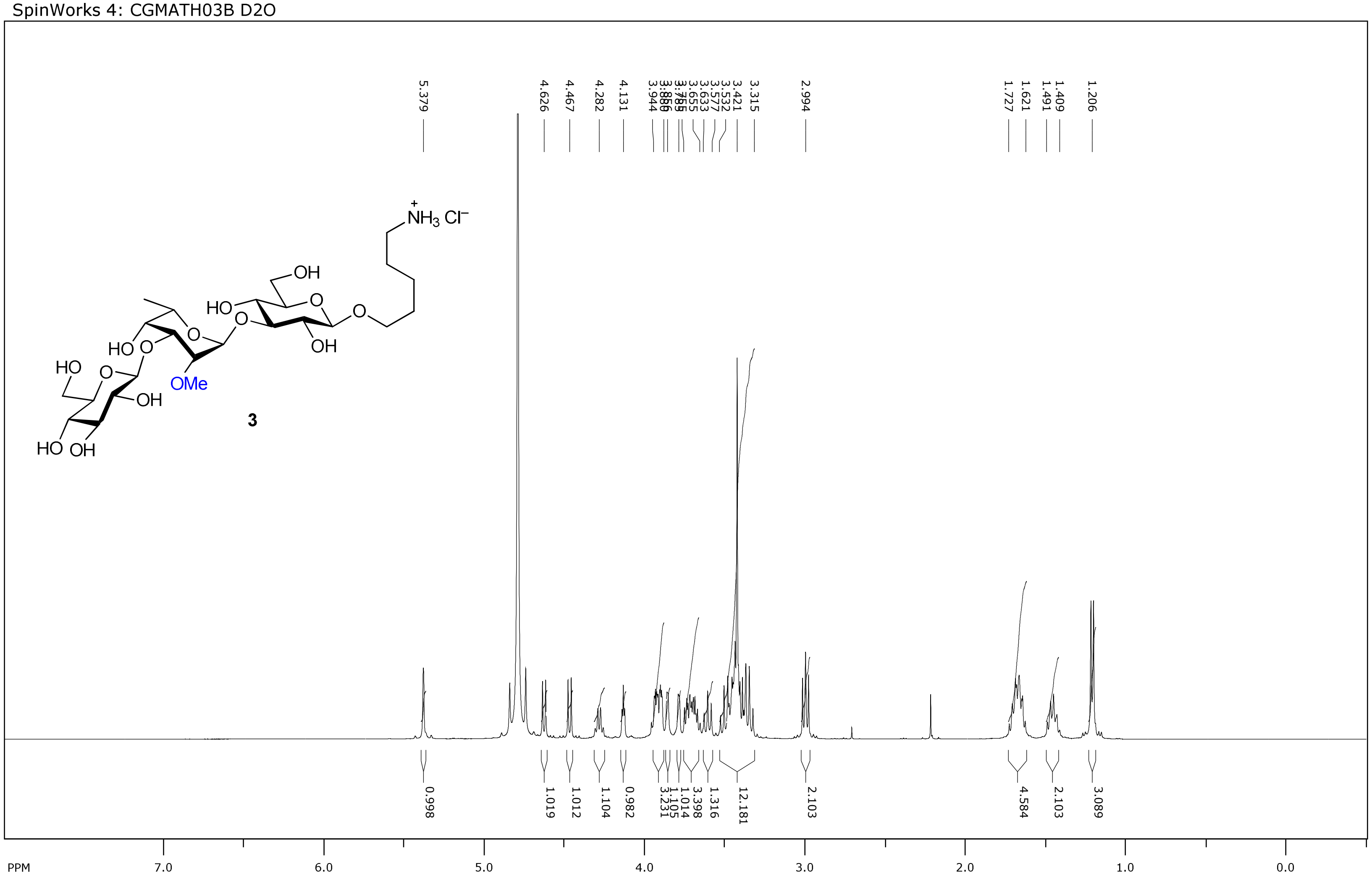

Supplementary Figure 152 | ^{13}C NMR spectra (D_2O + acetone, 100 MHz) of compound 3.

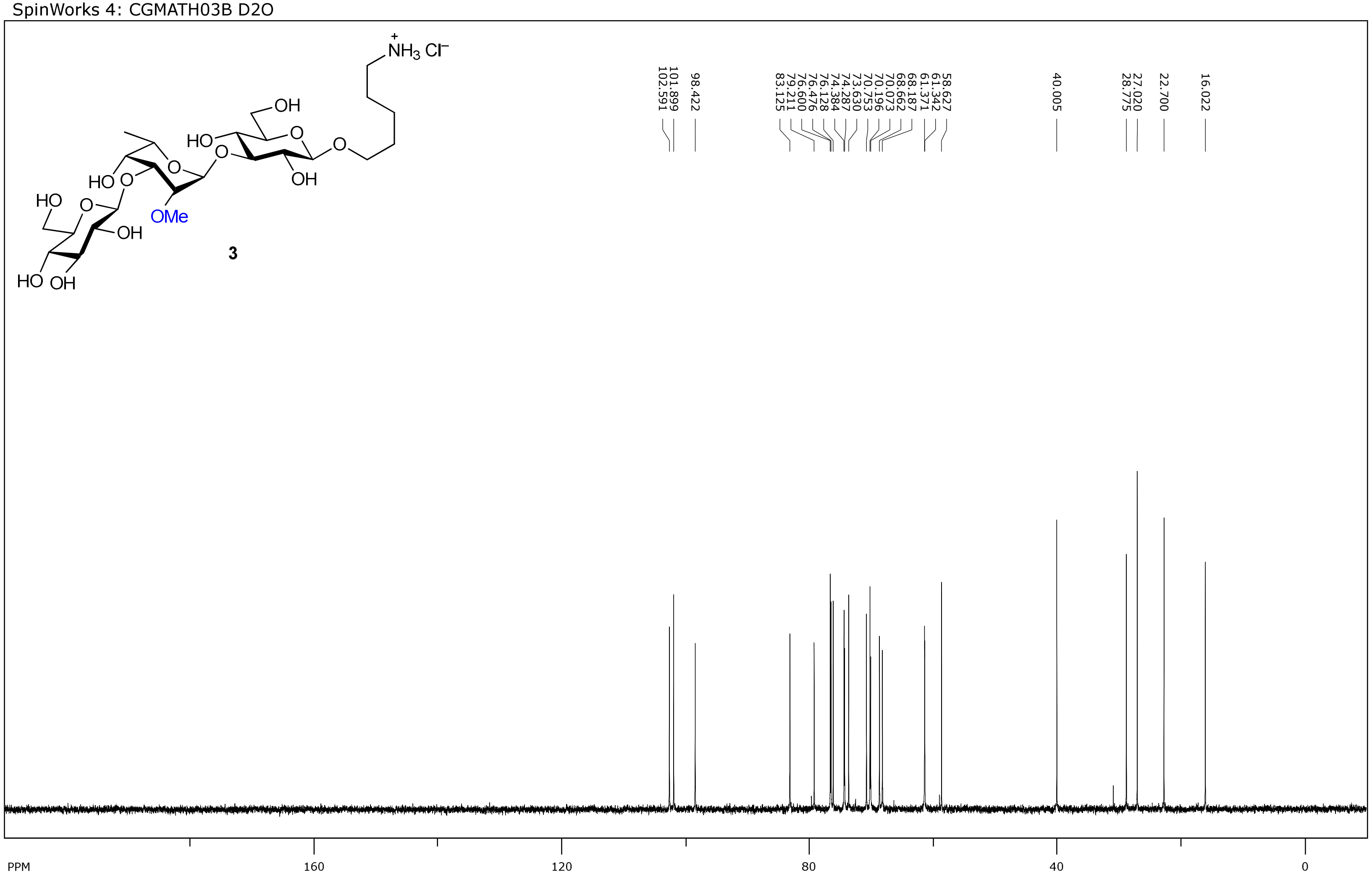

Supplementary Figure 153 │ ^{1}H NMR spectra (D_2O + acetone, 400 MHz) of compound 4.

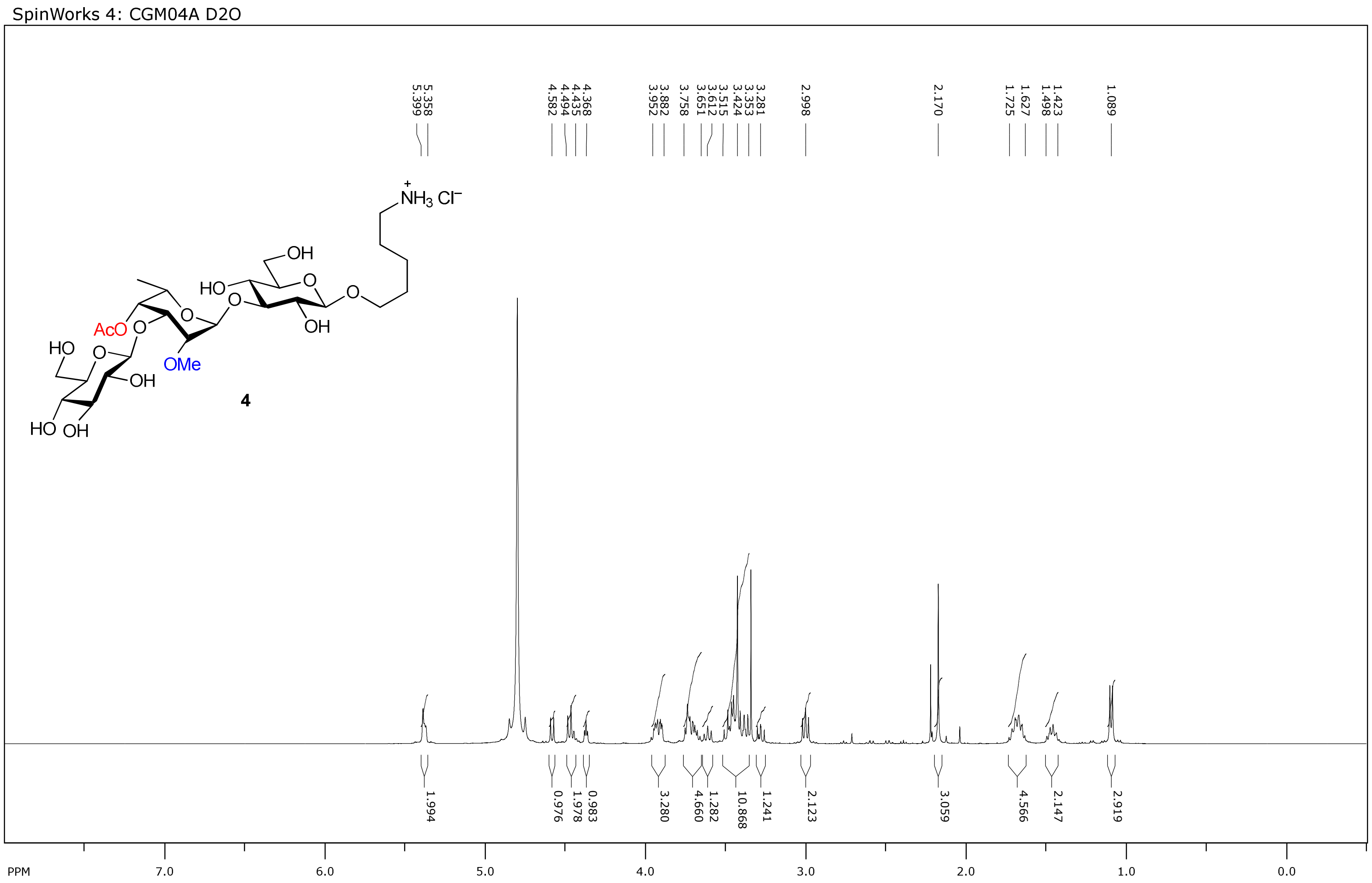

Supplementary Figure 154 | ^{13}C NMR spectra (D_2O + acetone, 100 MHz) of compound 4.

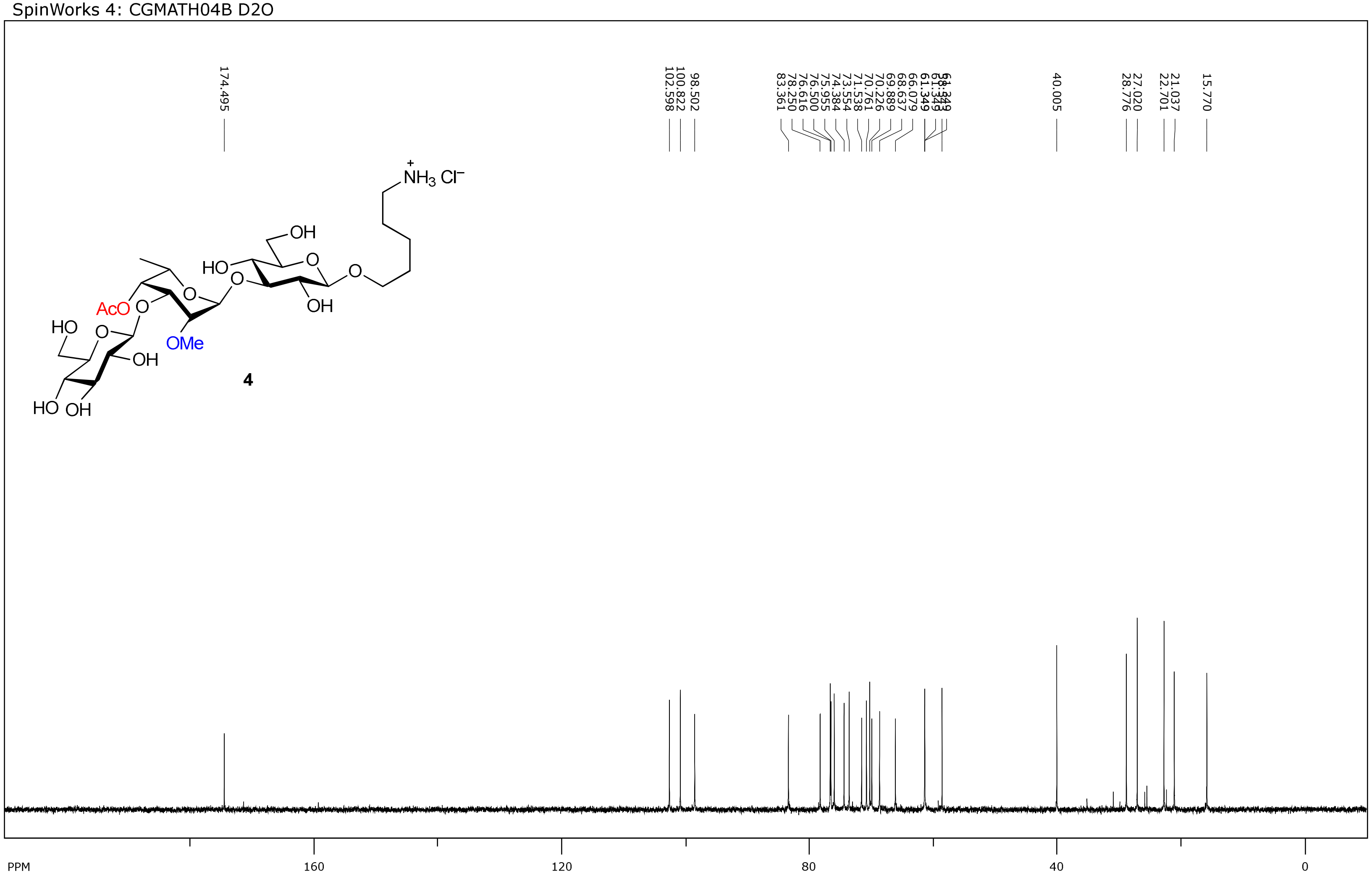

Supplementary Figure 155 | ^{1}H NMR spectra (D_2O + acetone, 400 MHz) of compound 5.

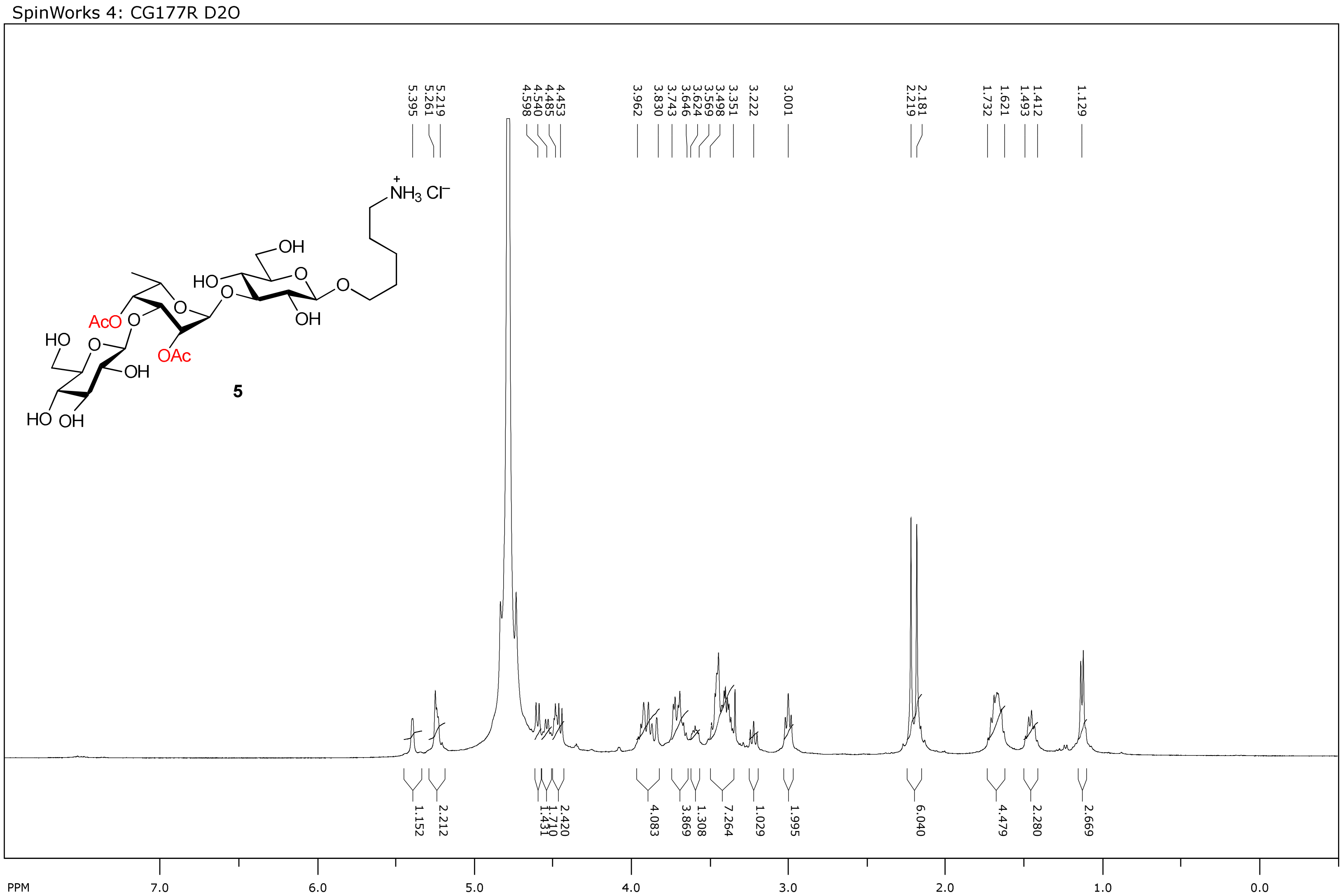

Supplementary Figure 156 | ^{13}C NMR spectra (D_2O + acetone, 100 MHz) of compound 5.

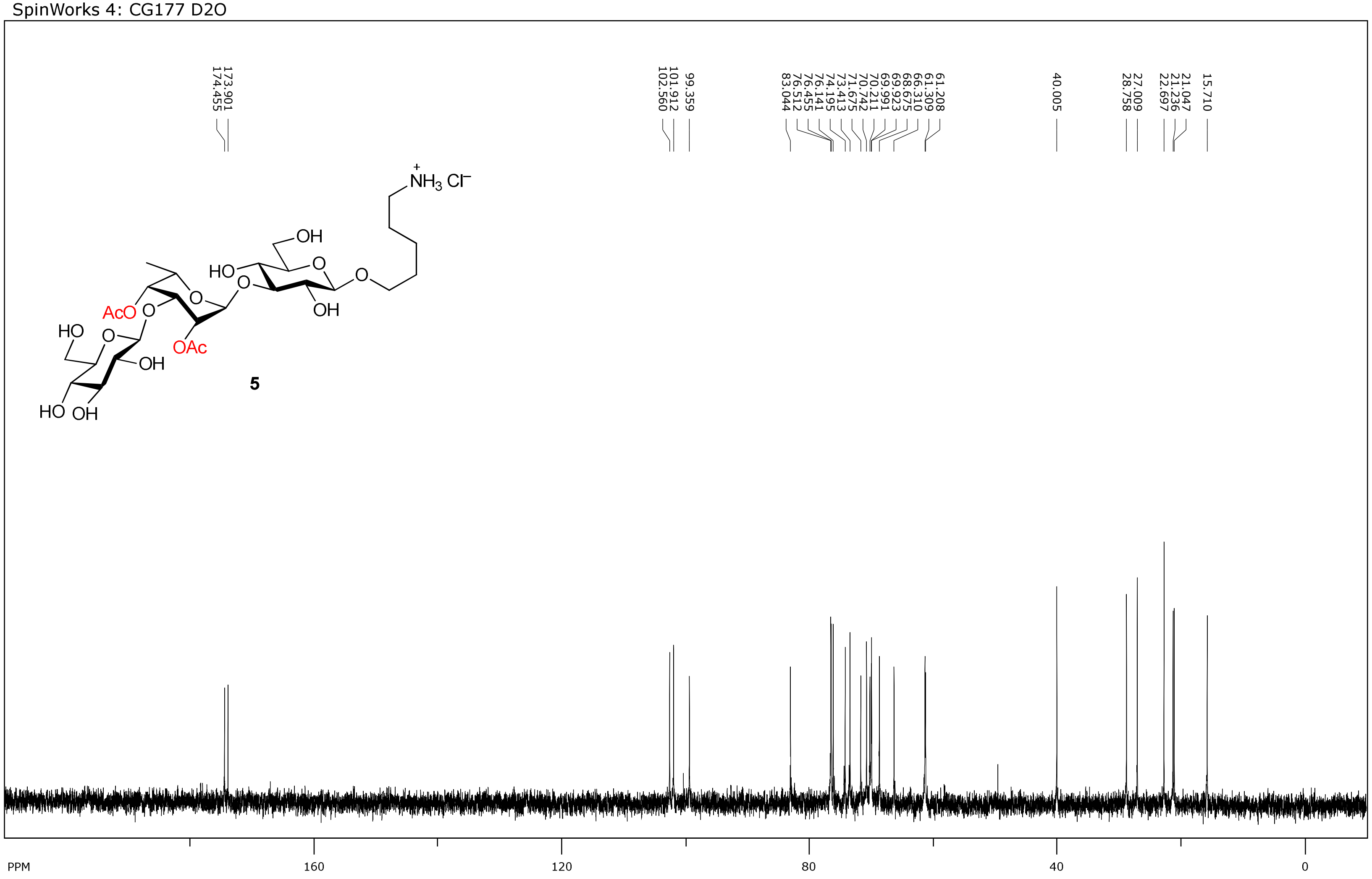

Supplementary Figure 157 │ ^{1}H NMR spectra (D_2O + acetone, 400 MHz) of compound 6.

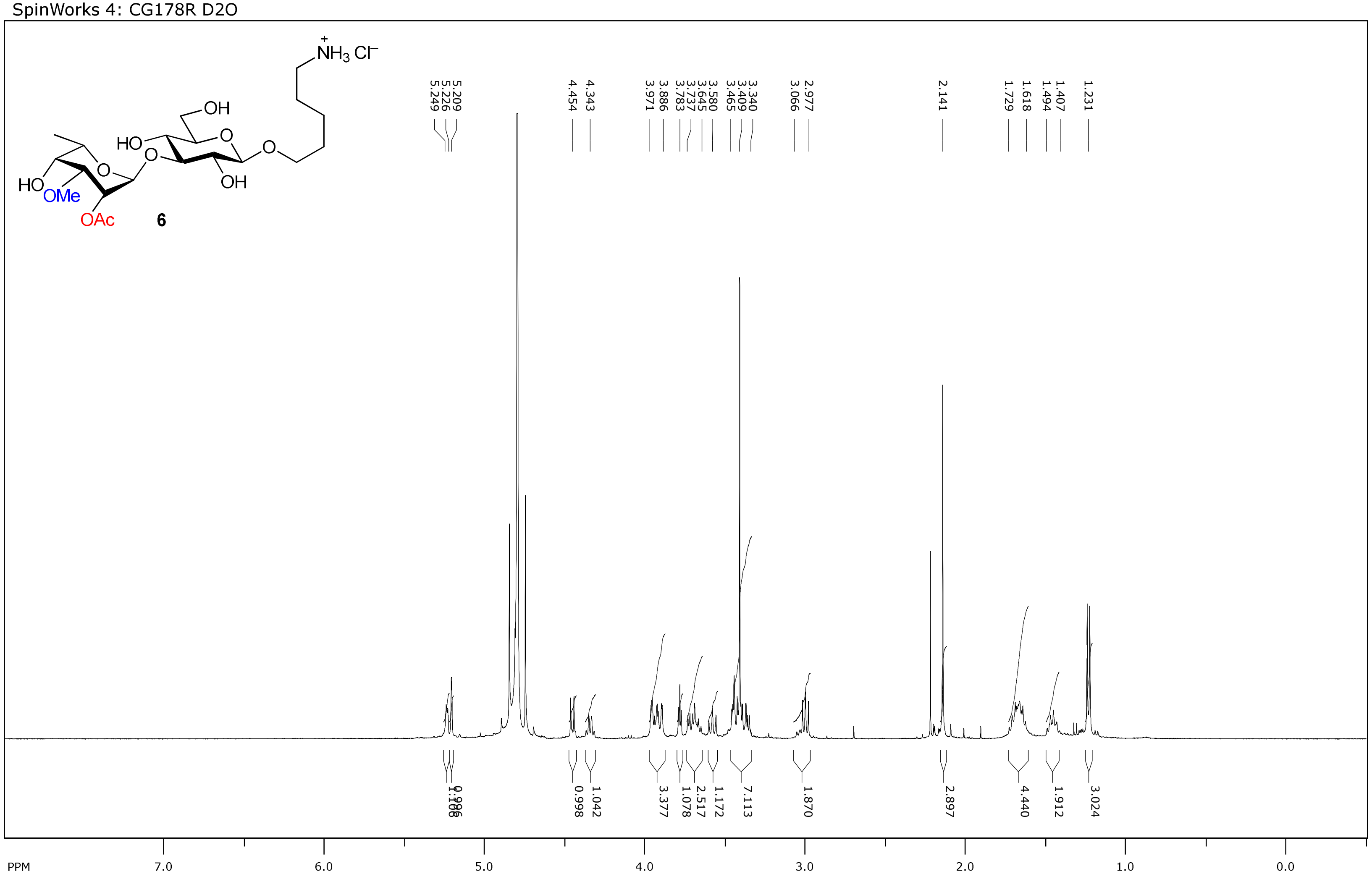

Supplementary Figure 158 | ^{13}C NMR spectra (D_2O + acetone, 100 MHz) of compound 6.

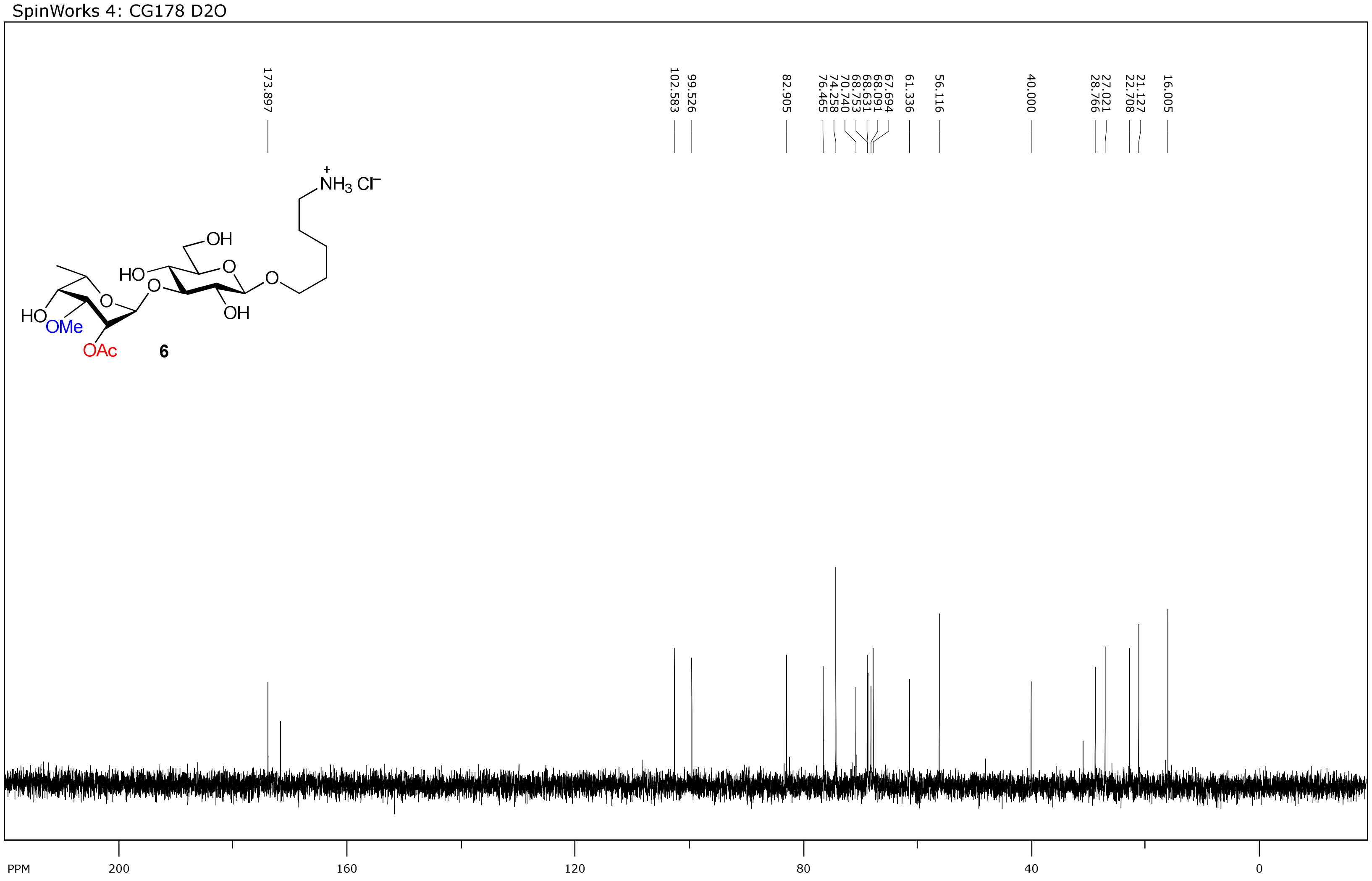

Supplementary Figure 159 │ ^{1}H NMR spectra (D_2O + acetone, 400 MHz) of compound 7.

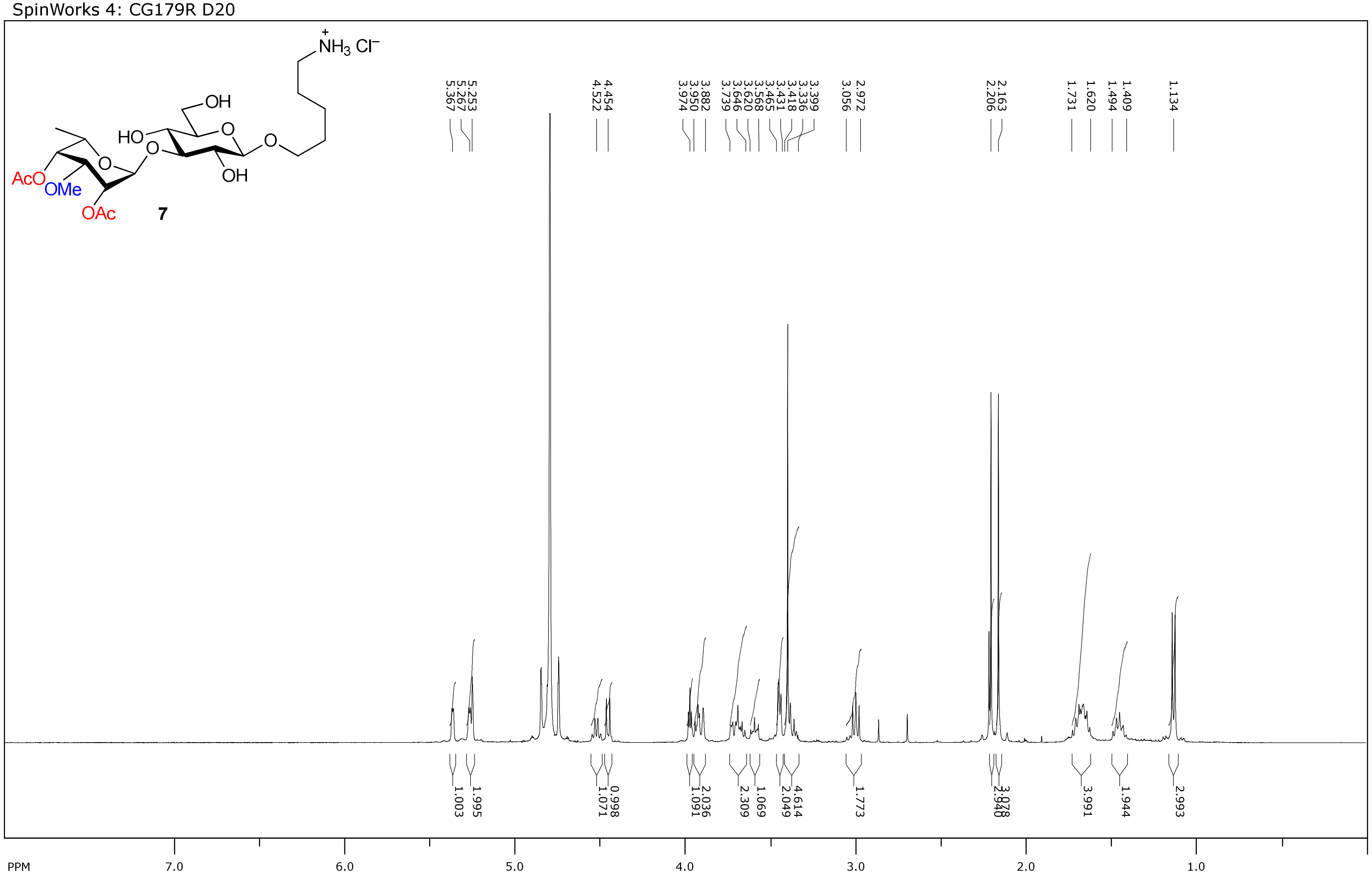

Supplementary Figure 160 | ^{13}C NMR spectra (D_2O + acetone, 100 MHz) of compound 7.

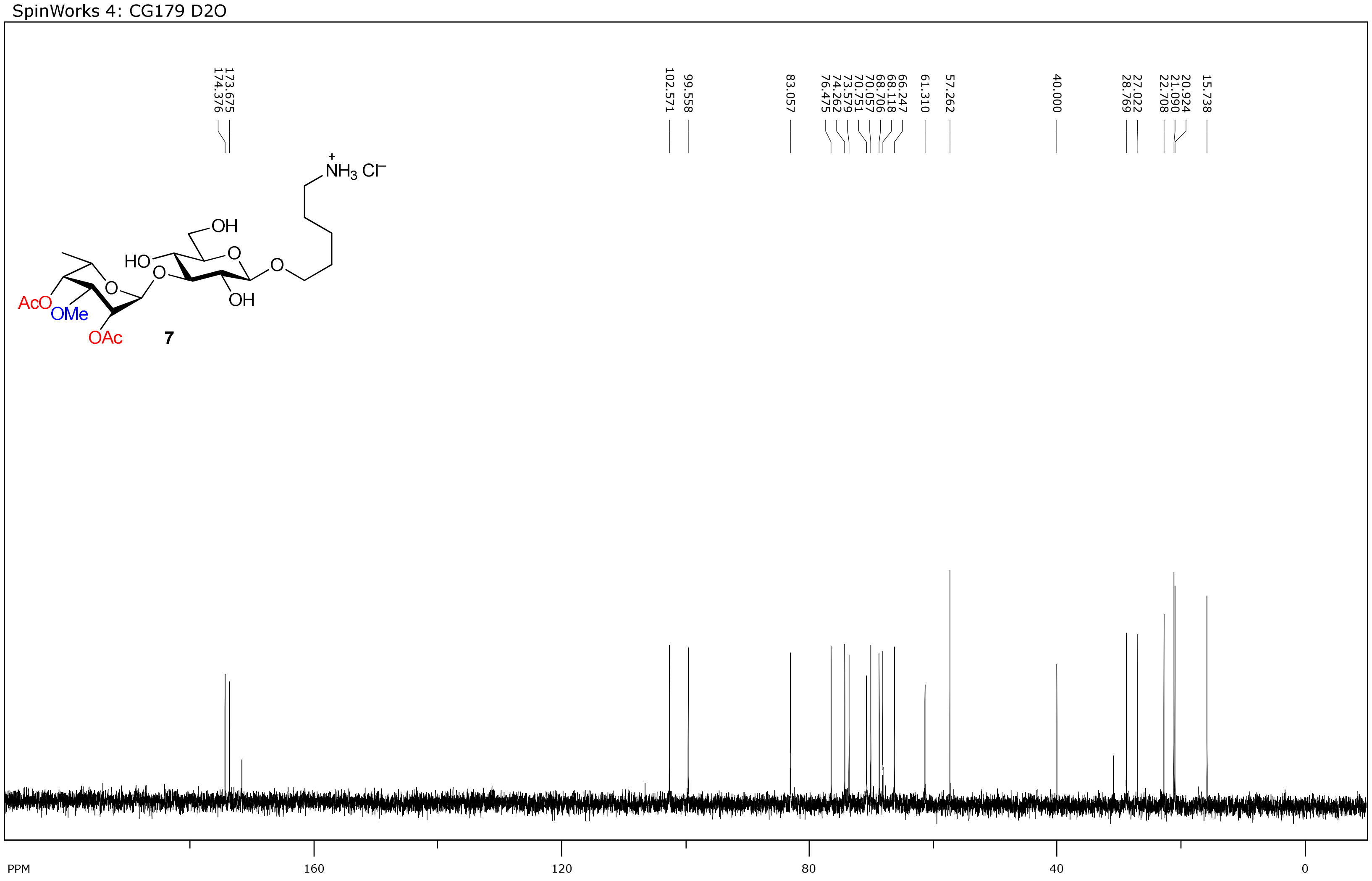

Supplementary Figure 161 │ ^{1}H NMR spectra (MeOD, 400 MHz) of compound BIO-6.

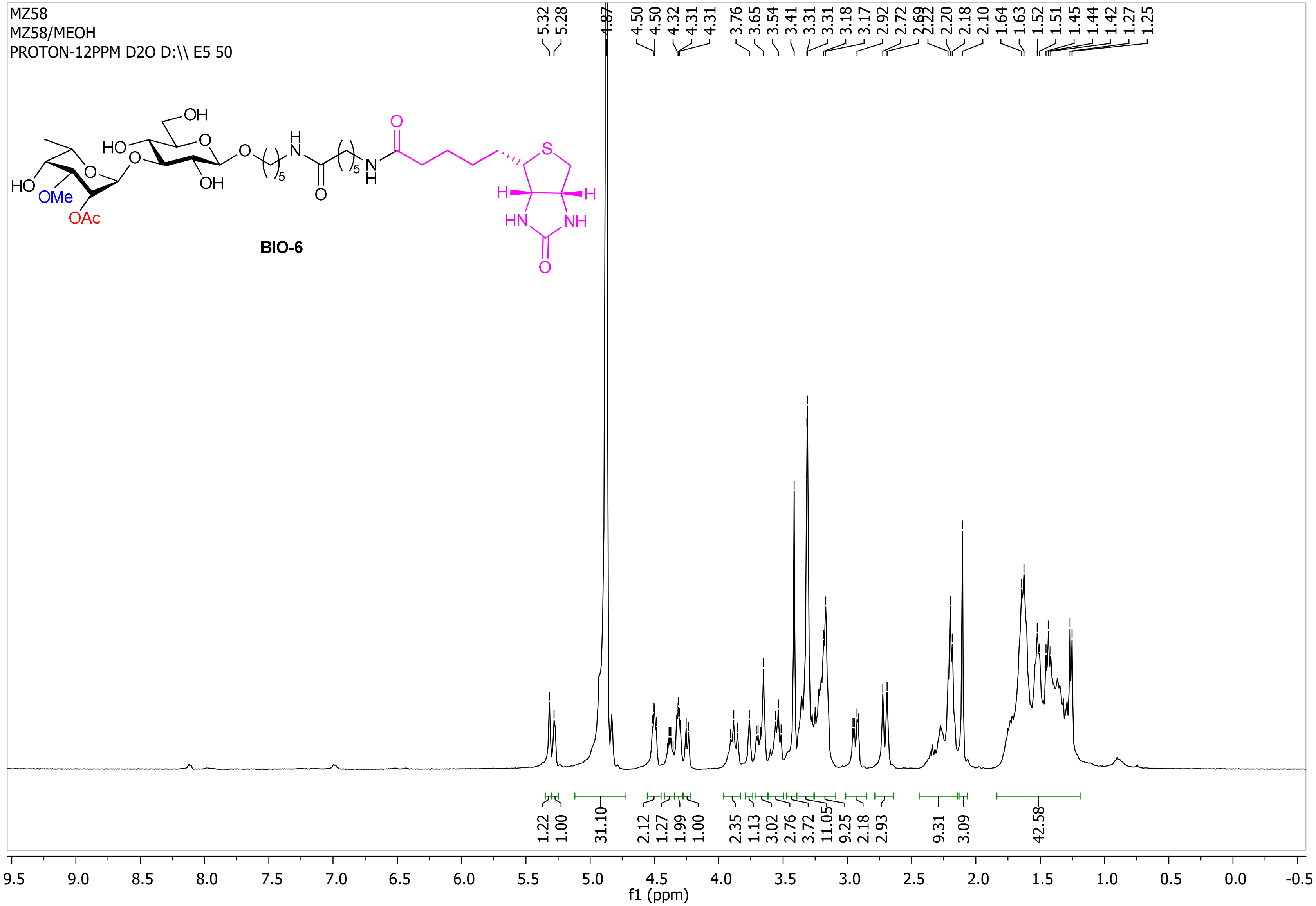

Supplementary Figure 162 | ^{13}C NMR spectra (MeOD, 100 MHz) of compound BIO-6.

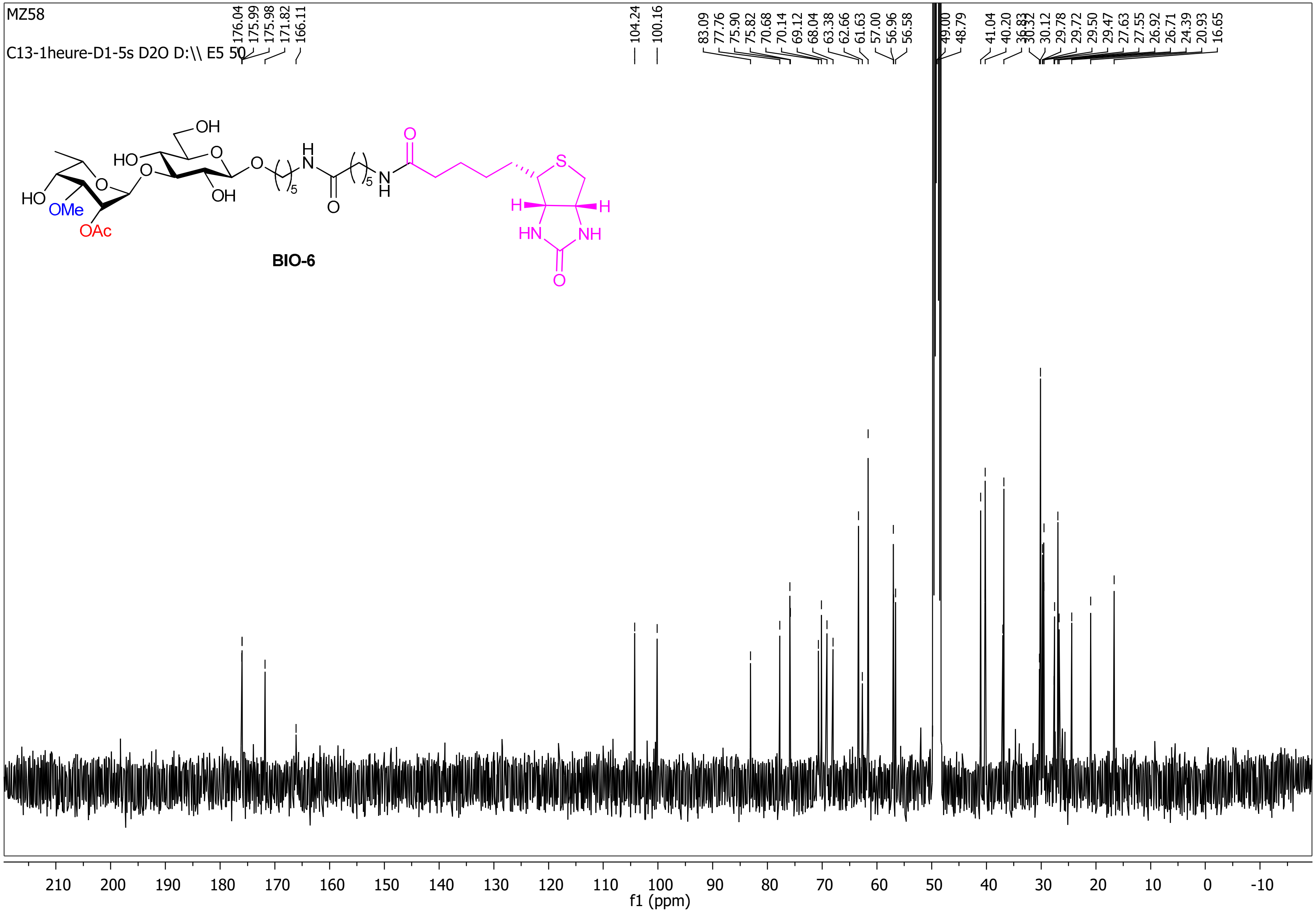

Supplementary Figure 163 │ ^{1}H NMR spectra (MeOD, 400 MHz) of compound BIO-7.

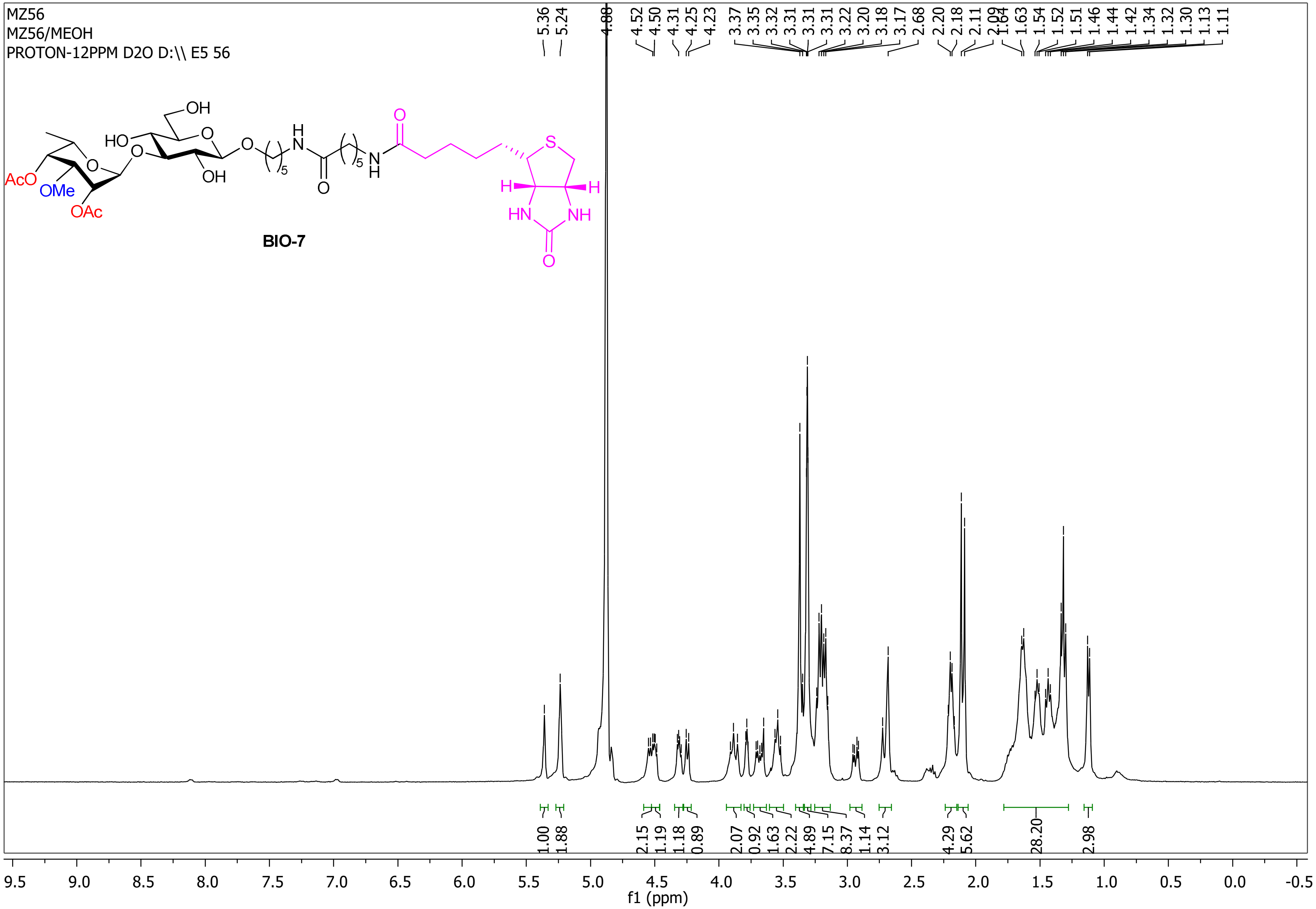

Supplementary Figure 164 | ^{13}C NMR spectra (MeOD, 100 MHz) of compound BIO-7.

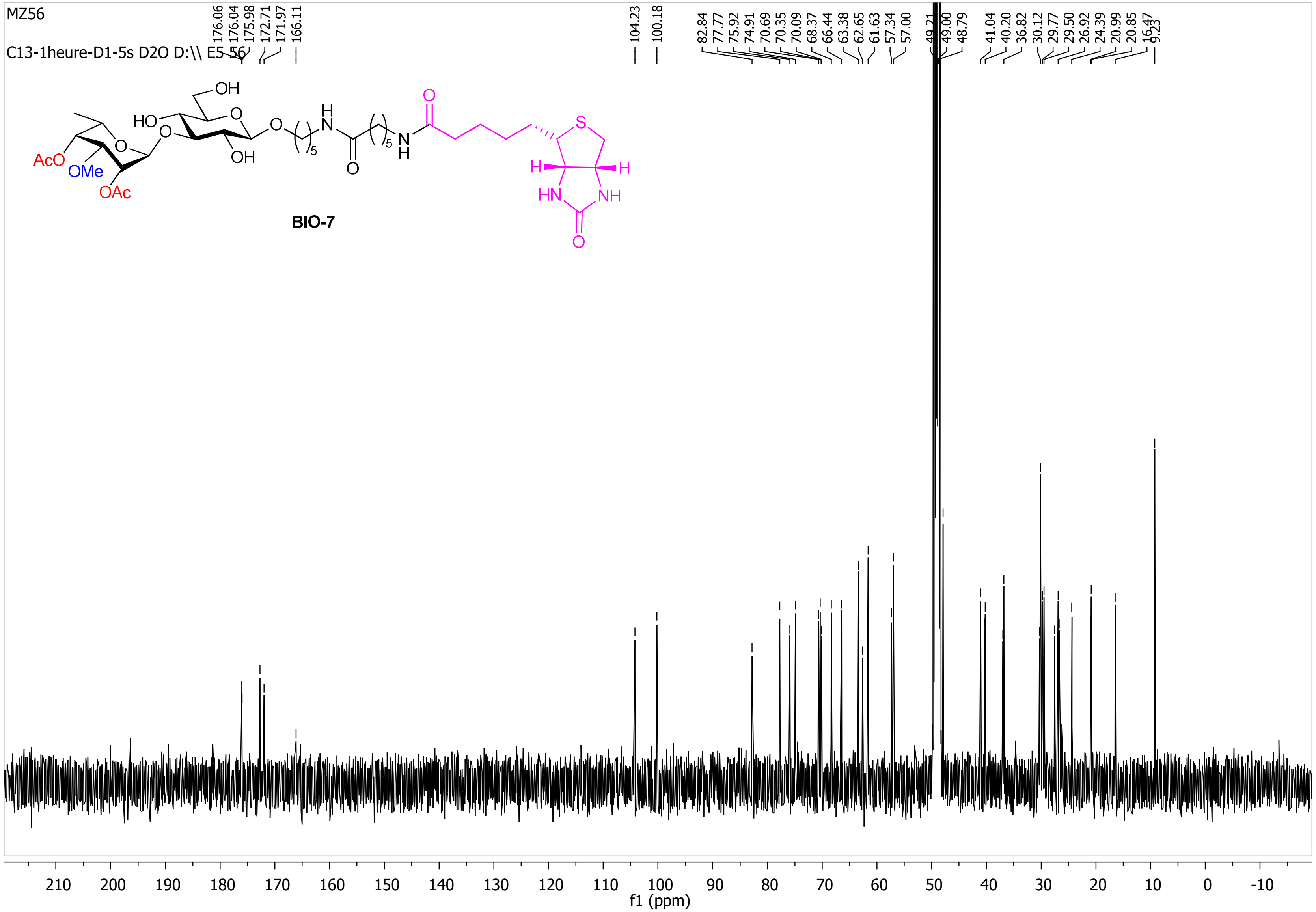

Supplementary Figure 165 | ^{1}H NMR spectra (MeOD, 400 MHz) of compound BIO-2.

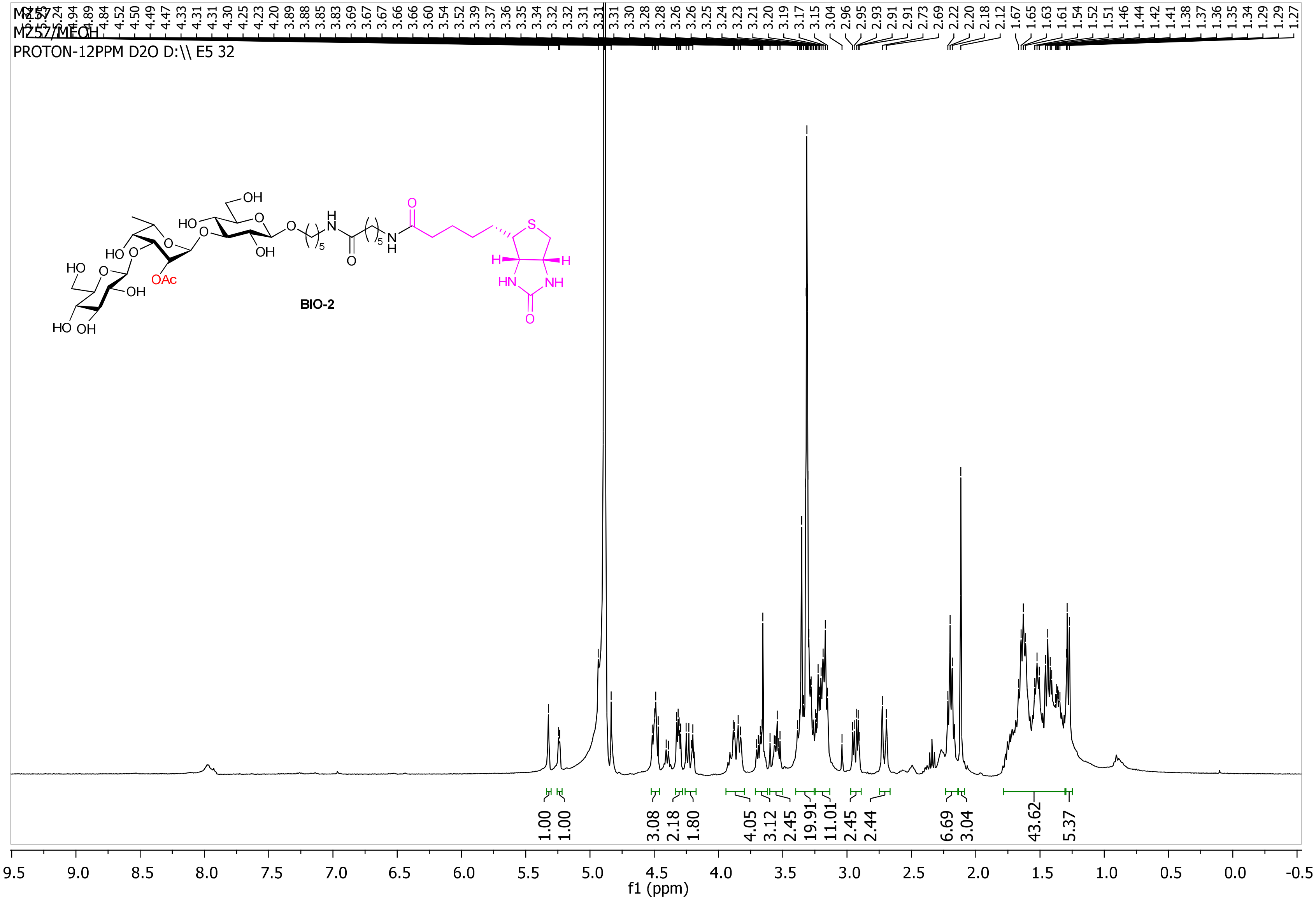

Supplementary Figure 166 | ^{13}C NMR spectra (MeOD, 100 MHz) of compound BIO-2.

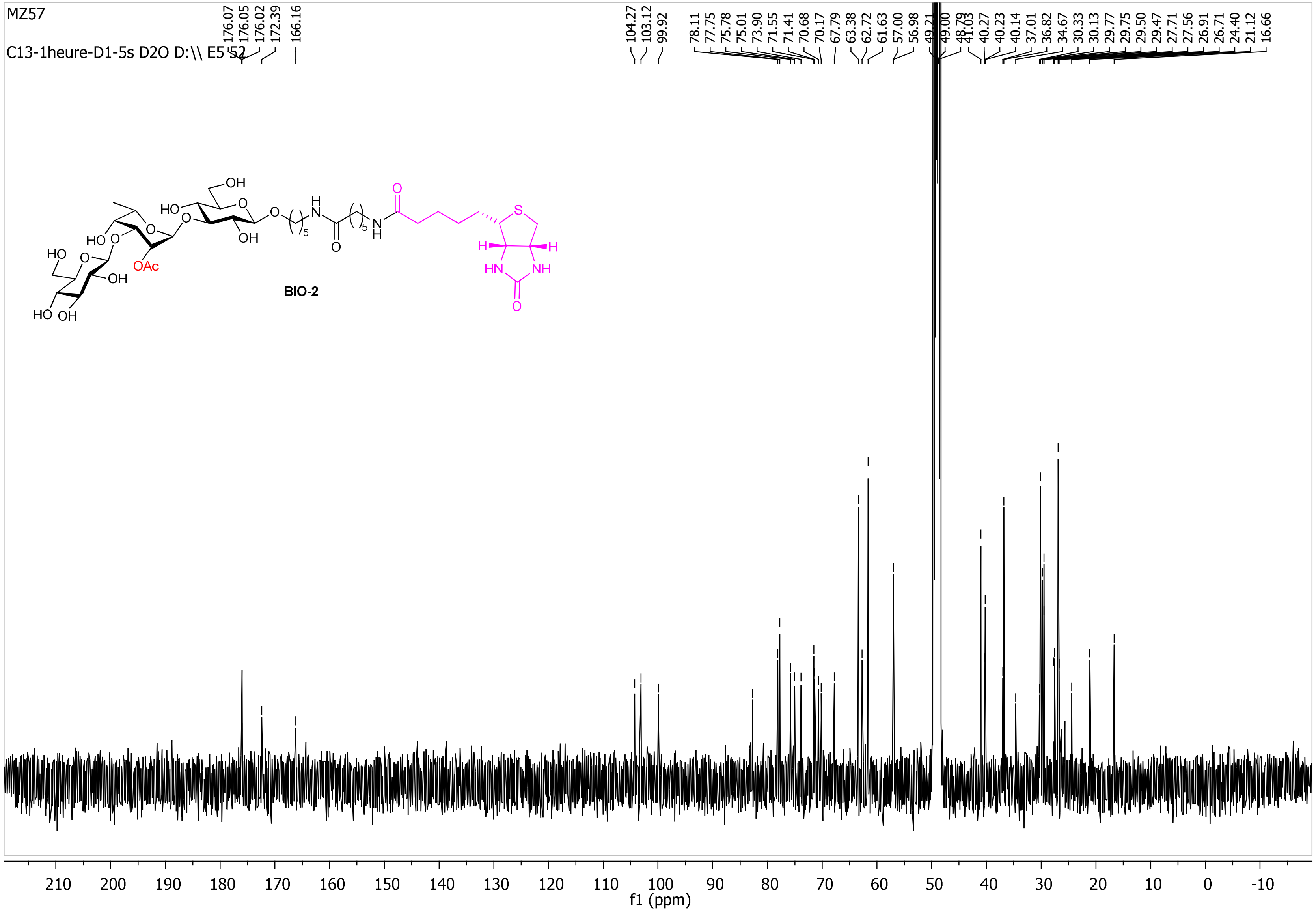

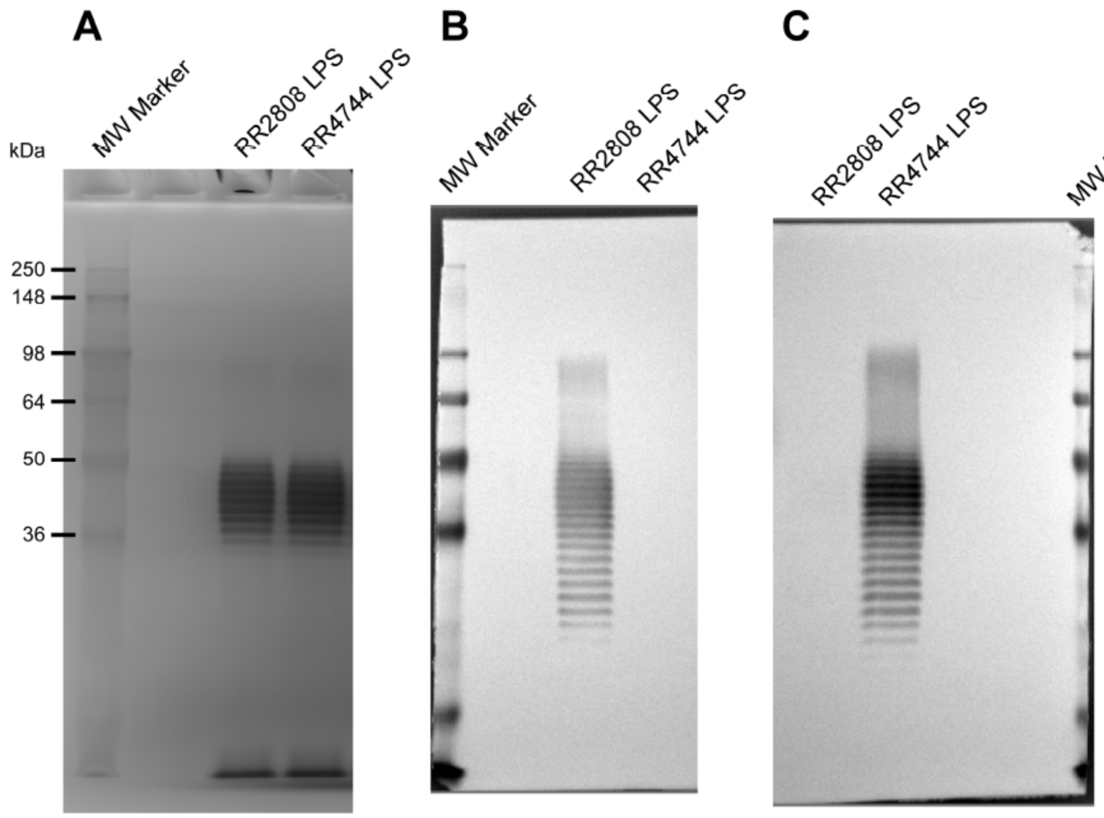

Supplementary Figure 167 │ Analysis of LPS antigens purified from wild type and mutant strains of *B. pseudomallei*. LPS antigens (2 µg/lane) were separated on 12% Tris-Glycine gels and visualized by (A) silver staining. For Western immunoblotting, LPS antigens were electrophoretically transferred to nitrocellulose membranes and probed with (B) mAb Pp-PS-W or (C) mAb 3D11. Wild type LPS was purified from *B. pseudomallei* RR2808 while OacA mutant LPS was purified from *B. pseudomallei* Bp RR4744. Data not shown: Similar to mAb 3D11, mAbs 4C7 and 9C1-2 only reacted with RR4744 LPS. Likewise, *B. mallei* LPS only reacted with mAbs 3D11, 4C7 and 9C1-2. Based on these results, Bp RR4744 OPS and *B. mallei* OPS antigens appeared to share a common epitope (see Figure 2).

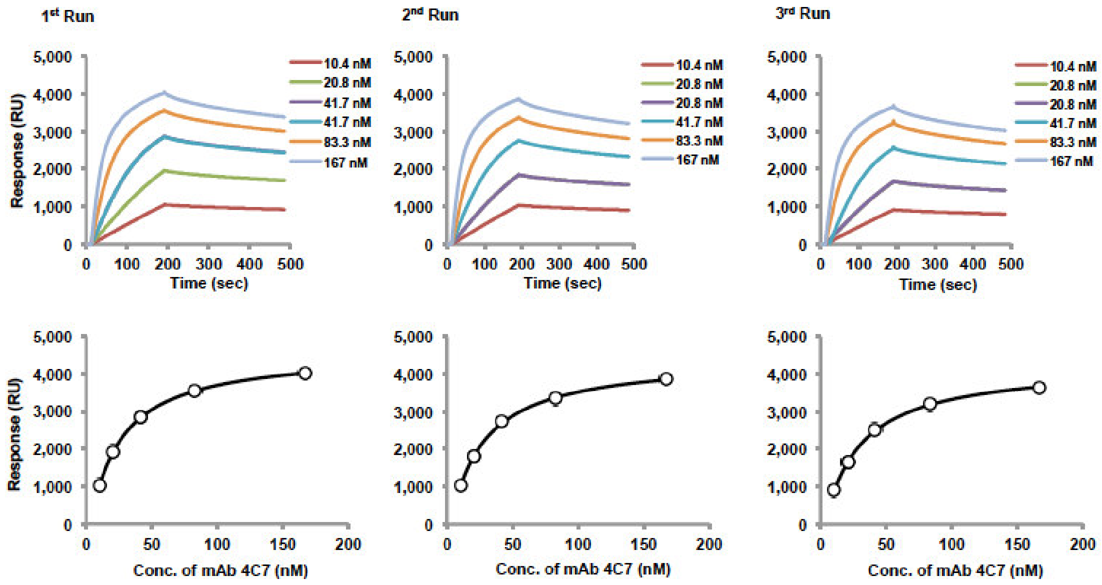

**K_D (nM)**	**20.1**	**22.2**	**24.0**
R_{max} (RU)	5421	5038	4859
χ^2 (RU^2)	113	1370	924

Supplementary Figure 168 │ Epitope mapping of disaccharide 6:mAb 4C7 interaction by STD-NMR (sensorgrams and steady-state affinity model fitting). SPR analysis was performed between mAb 4C7 and biotinylated oligosaccharide **BIO-6**. Binding affinities (K_D) were calculated using a steady-state affinity model.

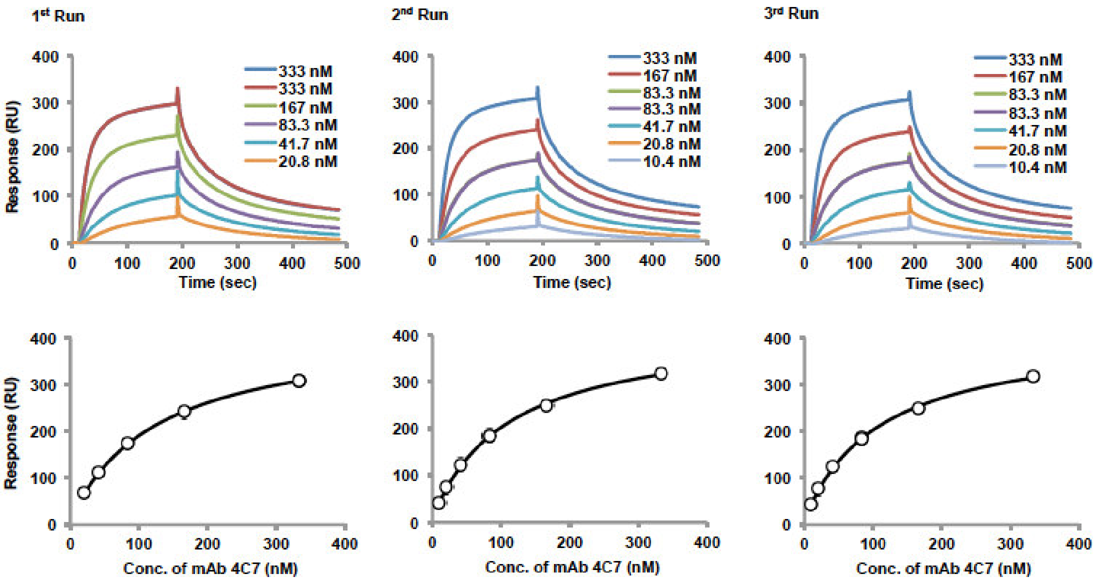

**K_D (nM)**	**128**	**110**	**110**
R_{max} (RU)	413	410	405
χ^2 (RU^2)	1.57	9.85	15.1

Supplementary Figure 169 │ Epitope mapping of disaccharide 7:mAb 4C7 interaction by STD-NMR (sensorgrams and steady-state affinity model fitting). SPR analysis was performed between mAb 4C7 and biotinylated oligosaccharide **BIO-7**. Binding affinities (K_D) were calculated using a steady-state affinity model.

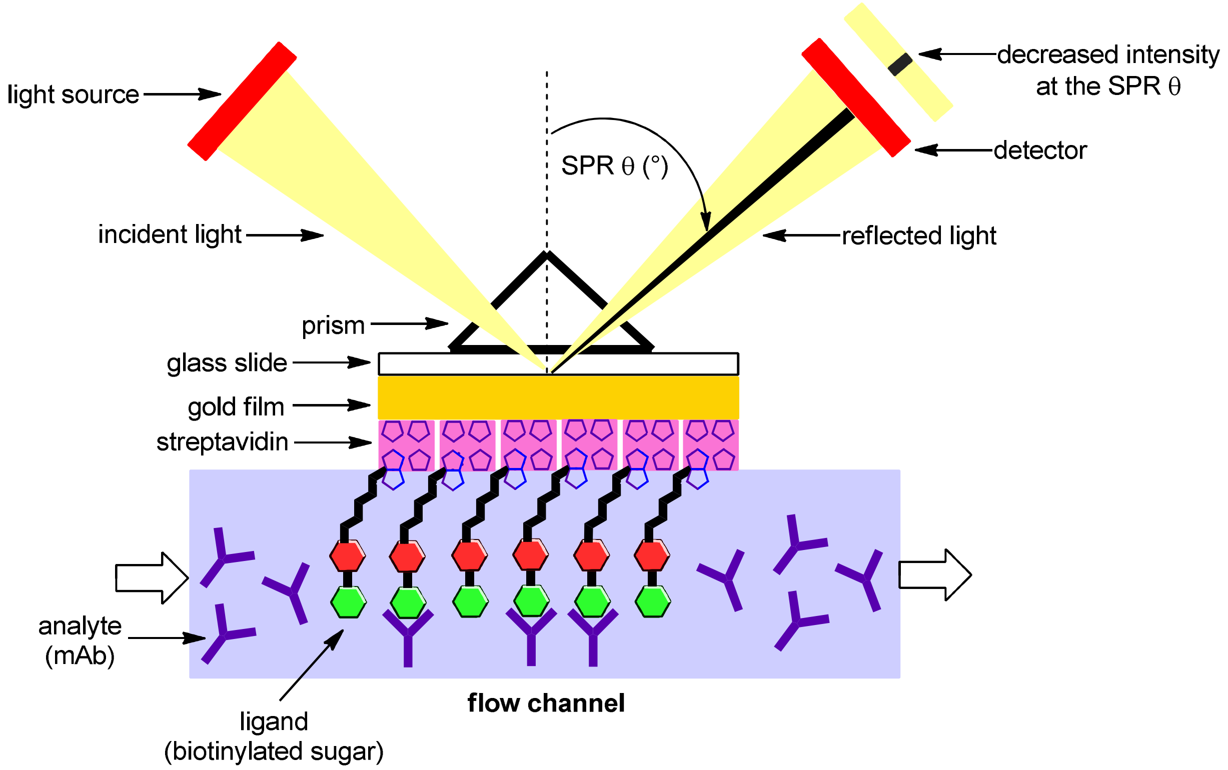

Supplementary Figure 170 │ Schematic illustration of the SPR experiments. Streptavidin-coated sensor chips were used in order to measure the K_D values of the biotinylated oligosaccharides:mAb 4C7 interactions.

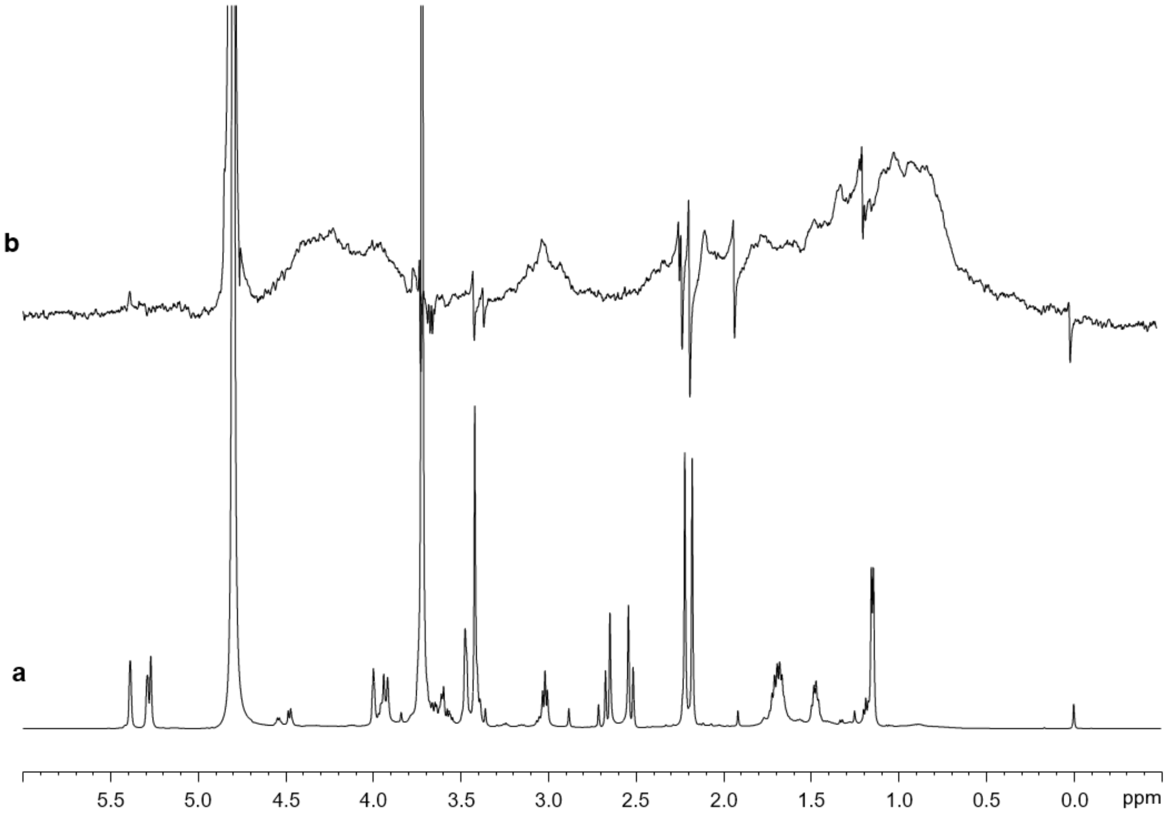

Supplementary Figure 171 │ STD-NMR spectrum of disaccharide 7 and mAb 4C7 mixture. (**a**) Reference ^{1}H NMR spectrum of disaccharide **7** at 298 K. (**b**) STD 1D NMR spectrum of a 1:100 mAb 4C7/disaccharide mixture. The irradiation frequency was set at 8 ppm and a saturation time of 2 seconds was used.

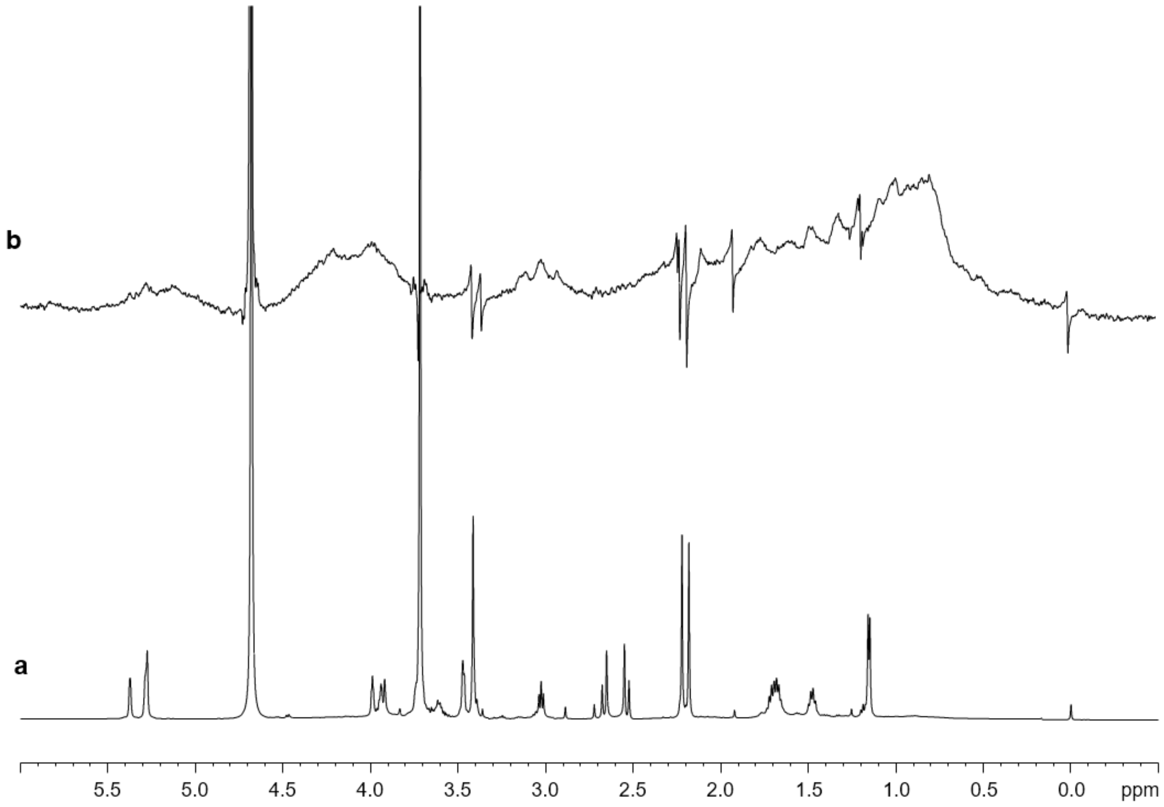

Supplementary Figure 172 │ STD-NMR spectrum of disaccharide 7 and mAb 4C7 mixture. (**a**) Reference ^{1}H NMR spectrum of disaccharide **7** at 310 K. (**b**) STD 1D NMR spectrum of a 1:100 mAb 4C7/disaccharide mixture. The irradiation frequency was set at 8 ppm and a saturation time of 2 seconds was used.

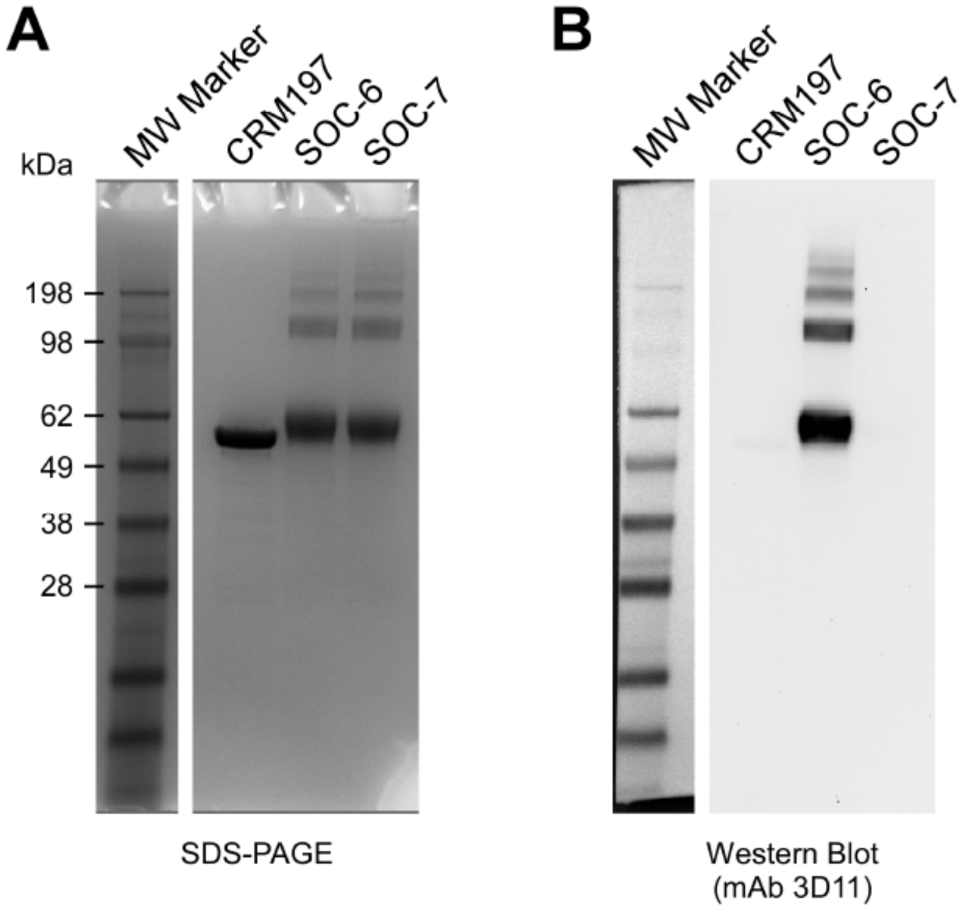

Supplementary Figure 173 │ SDS-PAGE and Western immunoblot analysis of synthetic oligosaccharide conjugates. (A) The carrier protein and conjugates (3 µg protein per lane) were separated on a 4-12% Bis-Tris Bolt gel and stained with CBB R-250; (B) The carrier protein and conjugates (1.5 µg protein per lane) were separated on 4-12% Bis-Tris Bolt gels and electrophoretically transferred to nitrocellulose. SOC-6 was detected by chemiluminescence using a 1/2000 dilution of mAb 3D11 and a 1/5000 dilution of an anti-mouse IgG-HRP conjugate. Results similar to mAb 3D11 were observed using mAbs 4C7 and 9C1-2 (data not shown). In contrast, SOC-7 was detected by chemiluminescence using a 1/400 dilution of mAb Pp-PS-W and a 1/5000 dilution of an anti-mouse IgM-HRP conjugate (data not shown).

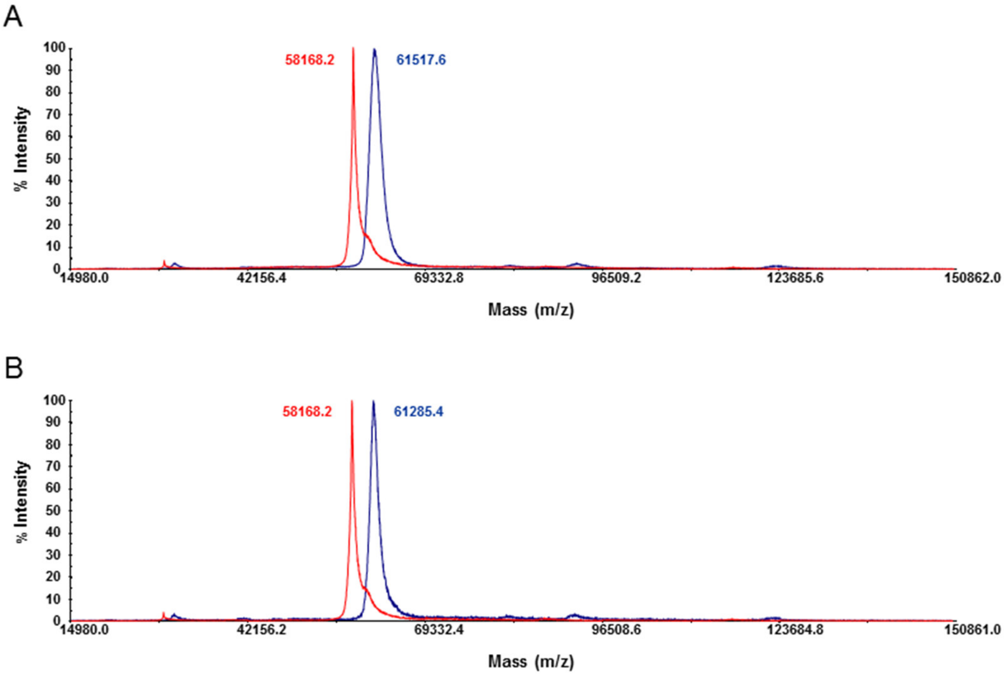

Supplementary Figure 174 │ MALDI-TOF-MS spectra of SOC-6 and SOC-7. (**a**) SOC-6 (blue line) and (**b**) SOC-7 (blue line) as well as unconjugated CRM197 (red line) were dried and reconstituted in 50 mM ammonium bicarbonate buffer (20 µL). The samples were deposited on a MALDI plate using premix method with 2,4,6-trihydroxyacetophenone (THAP) as the matrix. The MALDI analysis results were acquired on a TOF/TOFTM 5800 system (AB sCIEX) using linear positive ion mode. The data were externally calibrated using BSA. The analysis suggested that the mass of CRM197, SOC-6, and SOC-7 were 58.2 kDa, 61.5 kDa and 61.3 kDa, respectively. The mass differences indicated that SOC-6 and SOC-7 consisted of about 6 and 5 disaccharides covalently linked to CRM197, respectively.

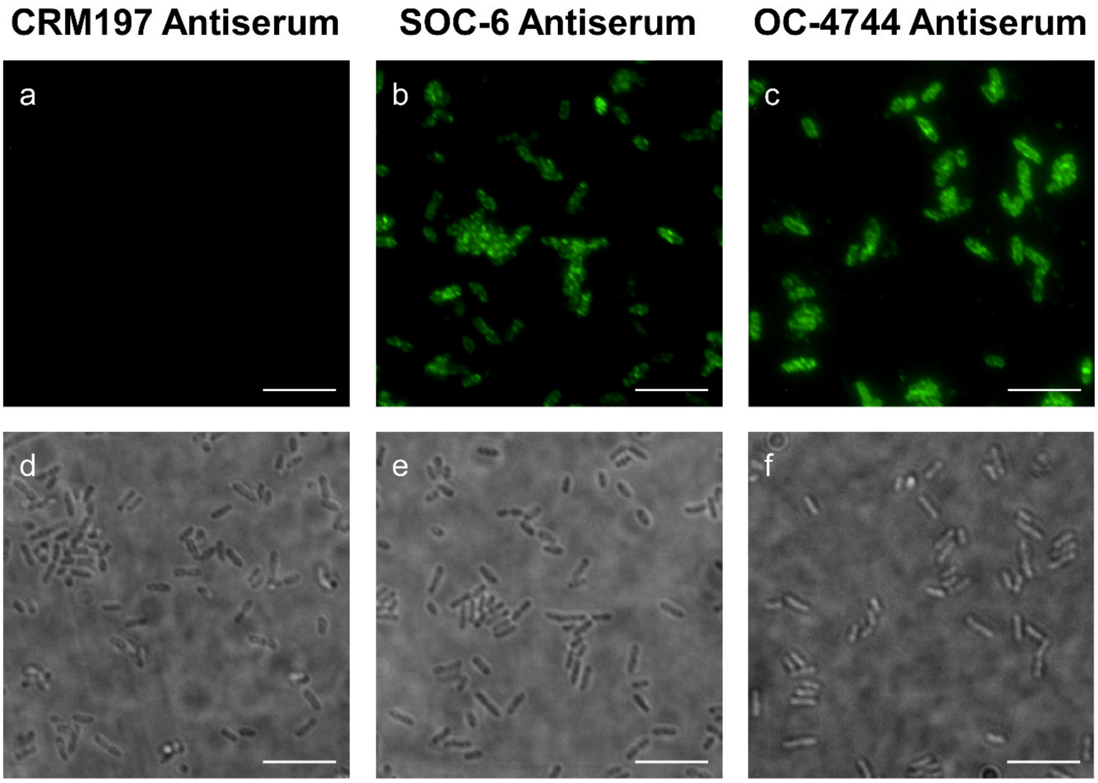

Supplementary Figure 175 │ Reactivity of CRM197, SOC-6 and OC-4744 antiserum with *B. mallei*. Paraformaldehyde-fixed *B. mallei* were labeled with CRM197 antiserum (a and d), **SOC-6** antiserum (b and e), or OC-4744 antiserum (c and f) and anti-mouse IgG-Alexa488 conjugate as described in the Supplementary Methods. Panels a-c show immunofluorescence images and panels d-f show bright field images. Scale bars are 5 µm.

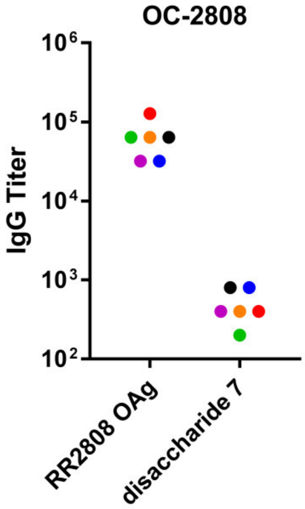

Supplementary Figure 176 | Immune responses to OC-2808. C57BL/6 mice (n = 6 per group) were immunized with OC-2808. ELISAs were used to quantitate immune serum IgG titers. Colored dots represent the mean endpoint titers for individual mice against the various target antigens.

Supplementary Methods

General methods

All starting materials and reagents were purchased from commercial sources, and used as received without further purification. Air and water sensitive reactions were performed in heat gun-dried glassware under Ar atmosphere. Moisture sensitive reagents were introduced via a dry syringe. Anhydrous solvents were supplied over molecular sieves, and used as received. Petroleum ether (PE) refers to the 40-60 °C boiling fraction. Powdered 4 Å molecular sieves were activated before use by heating with a heat gun for ~5 min under high vacuum. Reactions were monitored by thin-layer chromatography (TLC) with silica gel 60 F_{254} 0.25 mm pre-coated aluminium foil plates. Compounds were visualized by using UV_{254} and/or orcinol (1 mg·mL^{-1}) in 10% aq H_2SO_4 solution and/or Hanessian's stain [2.5 g $(NH_4)_6Mo_7O_{24}\cdot 4H_2O$, 1.0 g $Ce(NH_4)_4(SO_4)_4\cdot 2H_2O$, 90 mL H_2O, 10 mL H_2SO_4] with heating. Normal-phase flash column chromatography was performed on silica gel 60 Å (15-40 μm). Reversed-phase flash column chromatography was performed on C_{18} silica gel (fully capped, 25-40 μm). NMR spectra were recorded at 297 K in the indicated solvent ($CDCl_3$, py-d_5, D_2O or MeOD) with a 400 MHz instrument, employing standard softwares given by the manufacturer. ^1H and ^{13}C NMR spectra were referenced to tetramethylsilane (TMS, $\delta_H = \delta_C = 0.00$ ppm) as internal reference for spectra in $CDCl_3$, py-d_5, and MeOD or to internal acetone (δ_H = 2.218 ppm; δ_C = 33.0 ppm) for spectra in D_2O. Assignments were based on ^1H, ^{13}C, DEPT-135, COSY, HSQC, undecoupled HSQC and HMBC experiments. Interchangeable assignments are marked with an asterisk. High-resolution mass spectra (HRMS) were recorded on an ESI-Q-TOF mass spectrometer.

General procedures

Synthesis of trichloroacetimidate donors. 1,5-Cyclooctadiene-bis(methyldiphenylphosphine)-iridium(I) hexafluorophosphate (0.02–0.1 equiv) was dissolved in anhydrous THF (5 mL·mmol^{-1}) and the red solution was degassed under Ar. Hydrogen was bubbled through the solution for 5 min, and then the yellow solution was once again degassed under Ar. A solution of allyl taloside (1.0 equiv) in anhydrous THF (5.0 mL·mmol^{-1}) was added. The mixture was stirred for 2 h at rt under Ar. Then, a solution of iodine (2.0–2.5 equiv) in THF/H_2O (6.0 mL·mmol^{-1}, 4:1 *v/v*) was added to the mixture, which was stirred for another 1 h at rt. The excess of iodine was quenched by adding a freshly prepared 10% $Na_2S_2O_3$(aq) solution and stirred until the color turned bright yellow (~5 min). The aqueous phase was extracted with EtOAc (3 ×). The combined organic layers were washed with a saturated $NaHCO_3$(aq) solution and brine. The solvents of the dried solution ($MgSO_4$) were concentrated under reduced pressure. The residue was purified by silica gel flash chromatography to give the corresponding hemiacetal as an α/β mixture. To a cooled (0 °C) solution of the hemiacetal (1.0 equiv) in DCM/acetone (14 mL·mmol^{-1}, 8:3 *v/v*) were added DBU (0.3 equiv) or Cs_2CO_3 (0.2 equiv) followed by CCl_3CN (5.0–6.0 equiv). The mixture was stirred for 1 h at rt, then the suspension was filtered over Celite and rinsed with DCM. The solvents were concentrated under reduced pressure. The residue was purified by silica gel flash chromatography to give the trichloroacemidate donor, the α-anomer being the major compound.

Synthesis of protected disaccharides. Acceptor **13** (1.0 equiv) and donor **8-12** (2.0 equiv) were dried for 2 h under high vacuum and then dissolved in anhydrous Et_2O (20 mL·mmol^{-1}). The solution was cooled to –10 °C and TMSOTf (0.01–0.2 equiv) was added keeping rigorous anhydrous conditions. The mixture was stirred at –10 °C for 10 min under Ar, and then quenched with a few drops of Et_3N. The suspension was filtered over Celite, rinsed with DCM and the filtrate was concentrated under reduced pressure. The residue was purified by combi-flash chromatography to give the target disaccharide as a pure α-anomer.

Deprotection of PMB group. To a solution of disaccharide **15–17** (1.0 equiv) in DCM/H_2O (22 mL·mmol^{-1}, 10:1 *v/v*) was added DDQ (2.0 equiv) and the deep-green mixture was stirred for 2 h at rt. The reaction was quenched by adding a saturated $NaHCO_3$(aq) solution, stirred until the color turned bright yellow (~10 min), and diluted with EtOAc. The organic phase was washed with a saturated $NaHCO_3$(aq) solution and brine. The solvents of the dried solution ($MgSO_4$) were concentrated under reduced pressure. The residue was purified by silica gel flash chromatography to give the corresponding alcohol.

Hydrogenolysis using the H-Cube system. The oligosaccharide (1.0 equiv) was dissolved in DCE (10 mL·mmol^{-1}), then MeOH (250 mL·mmol^{-1}) followed by concentrated HCl (2.0 equiv) were added. The solution was passed without delay through a 20% $Pd(OH)_2$/C cartridge (CatCart30) using a H-Cube continuous flow system in control mode (10 bars). The temperature was set at 40 °C, and the flow rate was fixed at 1.0 mL·mmol^{-1}. After one run, the cartridge was rinsed with MeOH and the solutions were concentrated under reduced pressure keeping the bath temperature below 40 °C. The residue was subjected to C_{18} reversed-phase flash chromatography (H_2O/MeOH 10:0 to 6:4) followed by freeze-drying to give the target oligosaccharide in the form of a hydrochloride salt.

Hydrogenolysis under heterogeneous conditions. The oligosaccharide (1.0 equiv) was dissolved in anhydrous DCE (10 mL·mmol^{-1}), then anhydrous MeOH (250 mL·mmol^{-1}) followed by concentrated HCl (1.0 equiv) were added. The solution was degassed with Ar and Pd black (1 mg·mg^{-1} of compound) was added. The suspension was stirred under an atmosphere of H_2 at 40 °C for 16 h. The mixture was filtered over Celite to remove the catalyst, and the cake was rinsed with MeOH. The solutions were concentrated under reduced pressure keeping the bath temperature below 40 °C. The soluble part of the residue was dissolved in D_2O, filtered over Celite using a pipette, rinsed with D_2O and the solutions were concentrated under reduced pressure to give the target oligosaccharide in the form of a hydrochloride salt.

Biotinylation of oligosaccharides. A solution of the free oligosaccharide (1.0 equiv) and 6-biotinylamidohexanoic acid *N*-hydroxysuccinimidoyl ester (2.0 equiv) in DMF (22.5 mL·mmol^{-1}), Et_3N (2.5 mL·mmol^{-1}), and H_2O (25.0 mL·mmol^{-1}) was stirred for 1 h at rt. The solvents were concentrated under reduced pressure. The resulting residue was dissolved in EtOH and the soluble fraction was purified by silica gel flash chromatography (DCM/MeOH) to give the biotinylated oligosaccharide.

SDS-PAGE and Western immunoblotting. Glycoconjugate samples were solubilized in 1X SDS-PAGE sample buffer and heated to 100 °C for 5 min prior to electrophoresis on 4-12% Bis-Tris Bolt gels (Life Technologies). Proteins were visualized via staining with Coomassie Blue R-250. For Western immunoblot analyses, the glycoconjugate samples and CRM197 were separated on the same 4-12% gels and electrophoretically transferred to nitrocellulose membranes. The membranes were blocked with 3% skim milk in high salt Tris-buffered saline (HS-TBS; 20 mM Tris, 500 mM NaCl, pH 7.5) for 60 min at room temperature and then incubated overnight at 4 °C with 1/400 - 1/2000 dilutions of a *B. pseudomallei* (Pp-PS-W) or *B. mallei* OPS-specific mAbs (4C7, 3D11 and 9C1-2). To facilitate detection, the membranes were incubated for 1 h at room temperature with 1/5000 dilutions of an anti-mouse IgG horse radish peroxidase conjugate (SouthernBiotech). The blots were then visualized using Pierce ECL Western Blotting Substrate (Pierce).

Immunofluorescence staining and microscopy. *B. mallei* ATCC 23344 was cultured at 37 °C with aeration (200 rpm) in LB Lennox broth (Fisher Scientific) supplemented with 4% glycerol. Mid log phase bacteria were pelleted by centrifugation, fixed with 2.5% paraformaldehyde for 15 min then washed extensively with PBS and then blocked with PBS containing 10% normal goat serum (PBS-G; Invitrogen) for 20 min. Bacteria were stained with CRM197, OC-4744 or SOC-6 mouse antiserum (from mice represented by green dots in Fig 8a and 8b) diluted 1/500 in PBS-G for 30 min, washed three times with PBS and then incubated with Alexa Fluor 488 goat anti-mouse IgG (Invitrogen) diluted 1/1000 in PBS-G for 30 min. Stained bacteria were then washed three times with PBS, rinsed two times with water and mounted onto glass slides with ProLong Gold (Invitrogen) medium. Fluorescence and bright field microscopy was performed using a Nikon Eclipse 90i imaging system using a CFI Plan APO VC 100X/1.4 oil objective (Nikon Instruments Inc.). Images were acquired using NIS-Elements Advanced Research software (Nikon Instruments Inc.). All manipulations of *B. mallei* were conducted in CDC-approved and -registered biosafety level 3 facility at the University of South Alabama in accordance with standard select agent operating practices in compliance with the rules and regulations of the U.S. Federal Select Agent Program.

Immunogenicity evaluation. Groups of 6–8 week old female C57BL/6 mice (Charles River) were immunized subcutaneously on days 0, 21 and 35 with 10 μg of the OAg-CRM197 glycoconjugate OC-2808 formulated in saline plus Alhydrogel 2% (500 μg/mouse; Brenntag) and PolyI:C (PIC; 30 μg/mouse; InvivoGen). Terminal bleeds were conducted 14 days after the third immunization for the assessment of antibody responses. All procedures involving mice were performed according to protocols approved by the University of South Alabama Institutional Animal Care and Use Committee and were conducted in strict accordance with the recommendations in the Guide for the Care and Use of Laboratory Animals of the National Institutes of Health.

Synthetic methods and characterization data

Allyl 6-Deoxy-2,3-*O*-isopropylidene-*α*-L-talopyranoside (S2).

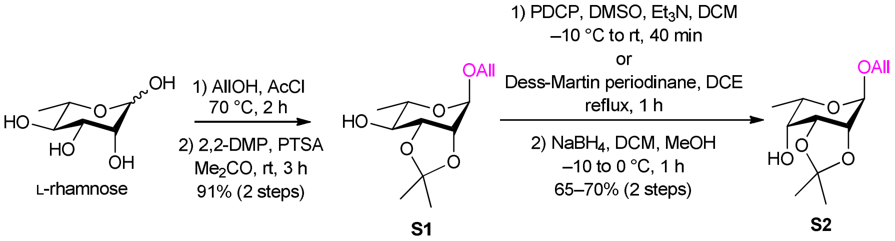

To a cooled (0 °C) solution of allylic alcohol (128 mL) was added dropwise acetyl chloride (9.9 mL, 138.8 mmol, 2.5 equiv). After 1 h, L-rhamnose (10 g, 55.5 mmol, 1.0 equiv) was added to the former solution and the reaction mixture was stirred at 70 °C. After 2 h, the reaction mixture was allowed to slowly warm up to rt and then solid $NaHCO_3$ was added. The mixture was filtered over Celite, rinsed with MeOH and the filtrate was concentrated under reduced pressure. The crude triol was dissolved in anhydrous acetone (61 mL) and 2,2-dimethoxypropane (21 mL, 166.5 mmol, 3.0 equiv) followed by a catalytic amount of PTSA (1.5 mg, 8.4 mmol, 0.15 equiv) were added. The reaction mixture was stirred for 3 h at rt under N_2 and diluted with DCM (150 mL). The organic phase was washed with water (3 × 50 mL). The solvents of the dried ($MgSO_4$) solution were concentrated under reduced pressure. The residue was purified by silica gel flash chromatography (PE/EtOAc 6:4 to 5:5) to give alcohol **S1**[1] (9.4 g, 91%, two steps) as a yellow oil: R_f 0.5 (DCM/MeOH 9:1); ^{1}H NMR (400 MHz, $CDCl_3$) δ (ppm): 5.95–5.85 (m, 1H, H-2$_{All}$), 5.31 (ddd, J = 17.2, 3.8, 1.7 Hz, 1H, H-3a$_{All}$), 5.22 (ddd, J = 10.3, 3.3, 1.4 Hz, 1H, H-3b$_{All}$), 5.01 (s, 1H, H-1), 4.19 (ddt, J = 12.5, 5.1, 1.9 Hz, 1H, H-1a$_{All}$), 4.17 (d, J = 5.6 Hz, 1H, H-2), 4.10 (t, J = 6.9 Hz, 1H, H-3), 4.01 (ddt, J = 12.6, 6.2, 2.0 Hz, 1H, H-1b$_{All}$), 3.73–3.66 (m, 1H, H-5), 3.40 (ddd, J = 9.3, 5.9, 4.6, 2.0 Hz, 1H, H-4), 2.50 (d, J = 4.6 Hz, 1H, O*H*), 1.53 (s, 3H, CH_3), 1.36 (s, 3H, CH_3), 1.30 (d, J = 6.5 Hz, 3H, CH_{3Tal}); ^{13}C NMR (100 MHz, $CDCl_3$) δ (ppm): 133.6 (C-2$_{All}$), 117.9 (C-3$_{All}$), 109.5 (*C*(CH_3)$_2$), 96.2 (C-1), 78.3 (C-3), 75.8 (C-2), 74.5 (C-4), 68.0 (C-1$_{All}$), 65.9 (C-5), 27.9, 26.1 (2 x *C*H_3), 17.5 (*C*H_{3Tal}).

Route A (Dess-Martin periodinane procedure): Alcohol **S1** (500 mg, 2.1 mmol, 1.0 equiv) was dissolved in anhydrous DCE (31 mL) at rt under Ar. Dess-Martin periodinane (1.9 g, 4.5 mmol, 2.2 equiv) was added and the mixture was refluxed for 1 h. The reaction mixture was cooled down to rt, diluted with DCM (30 mL) and washed with a 10% $Na_2S_2O_3$(aq) solution (20 mL). The organic phase was washed with brine (50 mL) and dried ($MgSO_4$). The solvents were concentrated under reduced pressure. The ketone was dissolved in MeOH/DCM (38 mL, 4:1 *v/v*), the solution was cooled to –10 °C, and $NaBH_4$ (250 mg, 6.5 mmol, 3.2 equiv) was slowly added. The mixture was stirred from –10 to 0 °C under Ar for 1 h. The reaction mixture was quenched by adding a 10% HOAc(aq) solution (2 mL) and then concentrated under reduced pressure. The residue was purified by silica gel flash chromatography (PE/EtOAc 90:10 to 85:15) to give alcohol **S2** (310 mg, 65%, two steps) as a colorless oil: R_f 0.4 (tol/EtOAc 8:2).

Route B (Pfitzner-Moffatt procedure): To a solution of DMSO (4.4 mL, 61.4 mmol, 5.0 equiv) in anhydrous DCM (123 mL) at –10 °C under Ar were added sequentially with stirring PDCP (5.5 mL, 36.8 mmol, 3.0 equiv) and Et_3N (8.6 mL, 61.4 mmol, 5.0 equiv). Then a solution of alcohol

S1 (3.0 g, 12.3 mmol, 1.0 equiv) in DCM (61 mL) was added dropwise during 1 h. The reaction mixture was stirred at –10 °C for 10 min, then allowed to slowly warm up to rt. After 30 min, water (100 mL) was added. The organic phase was separated and the aqueous phase was extracted with DCM (3 × 40 mL). The combined organic phases were washed with brine. The solvents of the dried solution ($MgSO_4$) were concentrated under reduced pressure. To a cooled (–10 °C) solution of the ketone in MeOH (123 mL) was slowly added $NaBH_4$ (558 mg, 22.1 mmol, 1.8 equiv). The mixture was stirred from –10 to 0 °C under Ar for 1 h. Then, the reaction mixture was diluted with DCM (200 mL) and the organic layer was washed with water (120 mL). The aqueous layer was back extracted with DCM (3 × 50 mL). The combined organic phases were washed with brine, dried ($MgSO_4$) and then concentrated under reduced pressure. The residue was purified by silica gel flash chromatography (PE/EtOAc 9:1 to 8:2) to give alcohol **S2** (2.1 g, 70%, two steps) as a colorless oil: R_f 0.2 (PE/EtOAc 8:2); $[\alpha]_D^{20}$ = –46 (*c* 1.3, $CHCl_3$/THF 1:1); ^{1}H NMR (400 MHz, $CDCl_3$) δ 5.97–5.87 (m, 1H, H-2_{All}), 5.31 (ddd, *J* = 17.2, 3.5, 1.6 Hz, 1H, H-$3a_{All}$), 5.22 (ddd, *J* = 10.3, 3.2, 1.6 Hz, 1H, H-$3b_{All}$), 5.09 (s, 1H, H-1), 4.25-4.18 (m, 2H, H-3, H-$1a_{All}$), 4.07 (td, *J* = 6.4, 0.6 Hz, 1H, H-2), 4.03 (ddt, *J* = 12.8, 6.3, 1.3 Hz, 1H, H-$1b_{All}$), 3.87 (dd, *J* = 13.9, 6.5 Hz, 1H, H-5), 3.56 (t, *J* = 5.8 Hz, 1H, H-4), 2.19 (d, *J* = 6.7 Hz, 1H, O*H*), 1.59 (s, 3H, CH_3), 1.38 (s, 3H, CH_3), 1.33 (d, *J* = 6.5 Hz, 3H, CH_{3Tal}); ^{13}C NMR (100 MHz, $CDCl_3$) δ 133.8 (C-2_{All}), 117.9 (C-3_{All}), 109.4 (*C*(CH_3)$_2$), 96.8 (C-1), 73.5 (C-2), 73.1 (C-3), 68.4 (C-1_{All}), 67.1 (C-4), 64.6 (C-5), 25.9, 25.4 (2 × *C*H_3), 16.8 (*C*H_{3Tal}); HRMS (ESI-TOF) *m/z* $[M + H]^+$ calcd for $C_{12}H_{21}O_5$ 245.1384; found 245.1381; *m/z* $[M + Na]^+$ calcd for $C_{12}H_{20}NaO_5$ 267.1203; found 267.1206; *m/z* $[M + K]^+$ calcd for $C_{12}H_{20}KO_5$ 283.0942; found 283.0939.

Allyl 6-Deoxy-α-L-talopyranoside (**S3**).

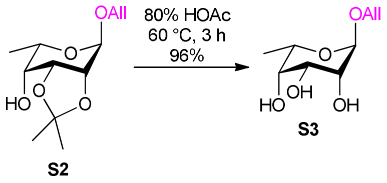

Alcohol **S2** (4.3 g, 17.5 mmol, 1.0 equiv) was dissolved in a 80% HOAc(aq) solution (220 mL). The reaction mixture was stirred at 60 °C for 3 h. Then, the mixture was concentrated under reduced pressure and co-evaporated with toluene (3 ×). Purification by silica gel flash chromatography (DCM/MeOH 98:2 to 85:15) gave triol **S3** (3.4 g, 96%) as a yellow oil: R_f 0.2 (PE/EtOAc 7:3); $[\alpha]_D^{20}$ = –97 (*c* 1.7, $CHCl_3$); ^1H NMR (400 MHz, $CDCl_3$) δ 5.94–5.84 (m, 1H, H-2_{All}), 5.28 (ddd, *J* = 17.2, 3.8, 2.4 Hz, 1H, H-$3a_{All}$), 5.19 (ddd, *J* = 10.4, 3.4, 2.1 Hz, 1H, H-$3b_{All}$), 4.91 (s, 1H, H-1), 4.16 (ddt, *J* = 13.0, 5.2, 1.5 Hz, 1H, H-$1a_{All}$), 4.00 (ddt, *J* = 12.9, 6.0, 1.3 Hz, 1H, H-$1b_{All}$), 3.92 (dd, *J* = 14.0, 6.5 Hz, 1H, H-5), 3.81–3.80 (m, 2H, H-2, H-3), 3.68 (s, 1H, H-4), 1.29 (d, *J* = 6.5 Hz, 3H, CH_{3Tal}); ^{13}C NMR (100 MHz, $CDCl_3$) δ 133.9 (C-2_{All}), 117.5 (C-3_{All}), 99.9 (C-1), 73.0 (C-4), 70.7 (C-2), 68.3 (C-1_{All}), 66.8 (C-3), 66.4 (C-5), 16.6 (CH_{3Tal}); HRMS (ESI-TOF) *m/z* $[M + Na]^+$ calcd for $C_9H_{16}NaO_5$ 227.0890; found 227.0887.

Allyl 6-Deoxy-3,4-*O*-isopropylidene-*α*-L-talopyranoside (**S4**).

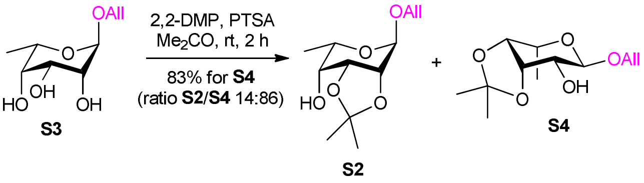

Triol **S3** (1.2 g, 6.1 mmol, 1.0 equiv) was dissolved in anhydrous acetone (7 mL). 2.2-DMP (2.2 mL, 18.4 mmol, 3.0 equiv) and PTSA (58 mg, 310 μmol, 0.05 equiv) were added sequentially. The mixture was stirred for 2 h at rt under Ar, then diluted with DCM (20 mL). The organic phase was washed with a saturated $NaHCO_3$(aq) solution (10 mL) and water (10 mL). The solvents of the dried ($MgSO_4$) solution were concentrated under reduced pressure. The residue was purified by silica gel flash chromatography (PE/EtOAc 9:1 to 7:3) to give alcohol **S4** (1.2 g, 83%) as a yellow oil: R_f 0.4 (PE/EtOAc 6:4); $[\alpha]_D^{20}$ = –43 (*c* 1.7, $CHCl_3$); ^{1}H NMR (400 MHz, $CDCl_3$) δ 5.98–5.88 (m, 1H, H-2_{All}), 5.31 (ddd, J = 17.2, 3.7, 1.6 Hz, 1H, H-$3a_{All}$), 5.19 (ddd, J = 10.4, 3.4, 0.9 Hz, 1H, H-$3b_{All}$), 4.81 (d, $J_{1,2}$ = 5.4 Hz, 1H, H-1), 4.52 (dd, J = 7.4, 3.4 Hz, 1H, H-3), 4.27 (ddt, J = 12.9, 5.3, 1.8 Hz, 1H, H-$1a_{All}$), 4.12 (dd, J = 7.4, 2.0 Hz, 1H, H-4), 4.05 (ddt, J = 12.8, 6.0, 1.7 Hz, 1H, H-$1b_{All}$), 3.85 (ddd, J = 13.8, 6.5, 2.0 Hz, 1H, H-5), 3.73 (dd, J = 5.5, 3.4 Hz, 1H, H-2), 1.53 (s, 3H, CH_3), 1.37 (s, 3H, CH_3), 1.25 (d, J = 6.5 Hz, 3H, CH_{3Tal}); ^{13}C NMR (100 MHz, $CDCl_3$) δ 134.4 (C-2_{All}), 117.3 (C-3_{All}), 110.0 (*C*(CH_3)$_2$), 100.0 (C-1), 76.2 (C-4), 73.7 (C-3), 68.8 (C-2), 68.6 (C-1_{All}), 65.2 (C-5), 26.1, 25.3 (2 × *C*H_3), 15.9 (*C*H_{3Tal}); HRMS (ESI-TOF) *m/z* $[M + Na]^+$ calcd for $C_{12}H_{20}NaO_5$ 267.1203; found 267.1209; *m/z* $[M + K]^+$ calcd for $C_{12}H_{20}KO_5$ 283.0942; found 283.0941.

Allyl 4-*O*-Benzyl-6-deoxy-*α*-L-talopyranoside (**S5**).

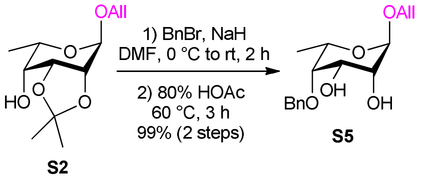

To a cooled (0 °C) solution of alcohol **S2** (6.0 g, 24.6 mmol, 1.0 equiv) in anhydrous DMF (100 mL), was slowly added NaH (60% oil dispersion, 1.5 g, 37.0 mmol, 1.5 equiv) under Ar and the reaction mixture was stirred for 1 h from 0 °C to rt. Then, the mixture was cooled again to 0 °C, BnBr (4.4 mL, 37 mmol, 1.5 equiv) was added dropwise and the reaction mixture was gradually warmed to rt. After being stirred for 2 h under Ar, the reaction was quenched with MeOH (5 mL) and diluted with EtOAc (250 mL). The organic layer was washed with water (2 × 50 mL), a 10% HCl(aq) solution (50 mL) and a saturated $NaHCO_3$(aq) solution (50 mL). Aqueous phases were back extracted with EtOAc (3 × 50 mL). Then, combined organic phases were washed with brine (100 mL) and the solvents of the dried solution ($MgSO_4$) were concentrated under reduced pressure. The residue was dried under high vacuum overnight, then dissolved in a 80% HOAc(aq) solution (308 mL). The reaction mixture was stirred at 60 °C for 3 h. Then, the mixture was concentrated under reduced pressure and co-evaporated with toluene (3 ×). Purification by silica gel flash chromatography (PE/EtOAc 9:1 to 6:4) gave diol **S5** (7.2 g, 99%, two steps) as a lite yellow foam: R_f 0.4 (PE/EtOAc 7:3); $[\alpha]_D^{20}$ = –88 (*c* 0.8, $CHCl_3$); ^1H NMR (400 MHz, $CDCl_3$) δ 7.40–7.28 (m, 5H, C*H*-Ar), 5.93–5.83 (m, 1H, H-2_{All}), 5.27 (ddd, *J* = 17.3, 3.6, 1.6 Hz, 1H, H-$3a_{All}$), 5.18 (ddd, *J* = 10.4, 3.4, 1.4 Hz, 1H, H-$3b_{All}$), 4.90 (s, 1H, H-1), 4.78 (d, *J* = 11.1 Hz, 1H, C*H*HPh), 4.71 (d, *J* = 11.1 Hz, 1H, C*H*HPh), 4.15 (ddt, *J* = 13.0, 5.1, 2.8 Hz, 1H, H-$1a_{All}$), 3.99 (ddt, *J* = 13.2, 6.0, 2.9 Hz, 1H, H-$1b_{All}$), 3.92 (dd, *J* = 13.8, 6.6 Hz, 1H, H-5), 3.87 (br s, 1H, H-4), 3.68 (d, *J* = 10.1 Hz, 1H, H-2), 3.64 (br s, 1H, H-3), 3.37 (d, *J* = 11.9 Hz, 1H, O*H*), 2.76 (s, 1H, O*H*), 1.27 (d, *J* = 6.6 Hz, 3H, CH_{3Tal}); ^{13}C NMR (100 MHz, $CDCl_3$) δ 137.3 (C-Ar), 133.8 (C-2_{All}), 128.6, 128.2, 128.0 (3 × *C*H-Ar), 117.3 (C-3_{All}), 100.1 (C-1), 81.4 (C-3), 76.7 (*C*H_2Ph), 70.8 (C-2), 68.2 (C-1_{All}), 66.8 (C-4), 66.0 (C-5), 16.9 (*C*H_{3Tal}); HRMS (ESI-TOF) *m/z* $[M + H]^+$ calcd for $C_{16}H_{23}O_5$ 295.1540; found 295.1542; *m/z* $[M + NH_4]^+$ calcd for $C_{16}H_{26}NO_5$ 312.1805; found 312.1804; *m/z* $[M + Na]^+$ calcd for $C_{16}H_{22}NaO_5$ 317.1359; found 317.1359.

Allyl 6-Deoxy-2-*O*-methyl-*α*-L-talopyranoside (**S7**).

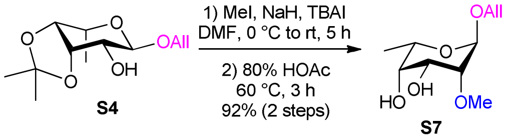

NaH (60% oil dispersion, 94 mg, 2.3 mmol, 2.5 equiv) was added dropwise to a cooled (0 °C) solution of alcohol **S4** (230 mg, 940 μmol, 1.0 equiv) in anhydrous DMF (5 mL). The mixture was stirred for 15 min at this temperature, then MeI (293 μL, 4.7 mmol, 5.0 equiv) and TBAI (35 mg, 94 μmol, 0.1 equiv) were added. The mixture was allowed to warm to rt and stirred for 5 h under Ar. The reaction mixture was diluted with EtOAc (20 mL), then poured into ice-cold brine (10 mL). The organic layer was washed again with brine (2 × 10 mL), dried over $MgSO_4$, concentrated under reduced pressure and co-evaporated with toluene (3 ×). The residue was dissolved in a 80% HOAc(aq) solution (11 mL). The reaction mixture was stirred at 60 °C for 3 h. Then, the mixture was concentrated under reduced pressure and co-evaporated with toluene (3 ×). Purification by silica gel flash chromatography (PE/EtOAc 8:2 to 7:3) gave diol **S7** (188 mg, 92%, two steps) as a yellow oil: R_f 0.2 (PE/EtOAc 7:3); $[\alpha]_D^{20}$ = –42 (*c* 1.4, $CHCl_3$); ^1H NMR (400 MHz, $CDCl_3$) δ 5.95–5.85 (m, 1H, H-2_{All}), 5.29 (ddd, J = 17.2, 3.7, 1.6 Hz, 1H, H-$3a_{All}$), 5.21 (ddd, J = 10.4, 3.3, 1.3 Hz, 1H, H-$3b_{All}$), 4.97 (d, $J_{1,2}$ = 1.2 Hz, 1H, H-1), 4.19 (ddt, J = 13.0, 5.1, 1.8 Hz, 1H, H-$1a_{All}$), 4.00 (ddt, J = 13.0, 6.0, 1.6 Hz, 1H, H-$1b_{All}$), 3.88 (ddd, J = 13.8, 6.5, 0.8 Hz, 1H, H-5), 3.82 (br s, 1H, H-3), 3.53 (d, J = 9.4 Hz, 1H, H-4), 3.49 (s, 3H, CH_{3Me}), 3.45 (dt, J = 3.5, 1.6 Hz, 1H, H-2), 3.02 (s, 1H, O*H*), 2.79 (d, J = 11.7 Hz, 1H, O*H*), 1.29 (d, J = 6.5 Hz, 3H, CH_{3Tal}); ^{13}C NMR (100 MHz, $CDCl_3$) δ 133.7 (C-2_{All}), 117.5 (C-3_{All}), 96 (C-1), 80.4 (C-2), 73.1 (C-4), 68.3 (C-1_{All}), 67.1 (C-5), 66.5 (C-3), 59.4 (CH_{3Me}), 16.5 (CH_{3Tal}); HRMS (ESI-TOF) *m/z* $[M + Na]^+$ calcd for $C_{10}H_{18}NaO_5$ 241.1046; found 241.1050.

Allyl 6-Deoxy-4-*O*-*para*-methoxybenzyl-2-*O*-methyl-*α*-L-talopyranoside (S8) and Allyl 6-Deoxy-3-*O*-*para*-methoxybenzyl-2-*O*-methyl-*α*-L-talopyranoside (S9).

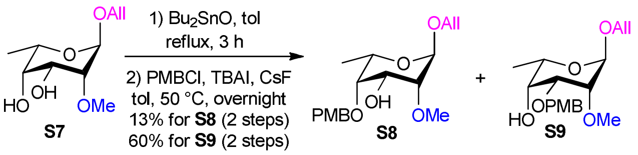

In a vessel equipped with a Dean-Stark apparatus, a suspension of Bu_2SnO (204 mg, 819 μmol, 1.05 equiv) and diol **S7** (170 mg, 780 μmol, 1.0 equiv) was refluxed in toluene (8 mL) for 5 h. The temperature was cooled to 50 °C, then TBAI (303 mg, 819 μmol, 1.05 equiv), CsF (121 mg, 796 μmol, 1.02 equiv) and PMBCl (116 μL, 858 μmol, 1.1 equiv) were successively added and the reaction was refluxed overnight. The reaction mixture was then concentrated under reduced pressure. The residue was purified by silica gel flash chromatography (PE/EtOAc 9:1 to 7:3) to give alcohol **S9** (196 mg, 60%) as a yellow amorphous solid along with its regioisomer **S8** (36 mg, 13%) as a yellow oil. Analytical data for **S9**: R_f 0.3 (PE/EtOAc 7:3); ^{1}H NMR (400 MHz, $CDCl_3$) δ 7.33–7.29 (m, 2H, C*H*-Ar), 6.89–6.86 (m, 2H, C*H*-Ar), 5.93–5.83 (m, 1H, H-2_{All}), 5.26 (ddd, *J* = 17.2, 3.8, 1.6 Hz, 1H, H-$3a_{All}$), 5.19 (ddd, *J* = 10.4, 3.4, 1.4 Hz, 1H, H-$3b_{All}$), 4.95 (d, $J_{1,2}$ = 1.5 Hz, 1H, H-1), 4.69 (d, *J* = 11.7 Hz, 1H, C*H*H_{PMB}), 4.53 (d, *J* = 11.7 Hz, 1H, CHH_{PMB}), 4.16 (ddt, *J* = 13.1, 5.0, 1.8 Hz, 1H, H-$1a_{All}$), 3.98 (ddt, *J* = 12.9, 6.0, 1.7 Hz, 1H, H-$1b_{All}$), 3.80 (s, 3H, CH_{3PMB}), 3.77 (dd, *J* = 13.0, 6.3 Hz, 1H, H-5), 3.74–3.70 (m, 1H, H-4), 3.69 (t, *J* = 3.3 Hz, 1H, H-3), 3.53 (dd, $J_{2,3}$ = 3.2 Hz, $J_{1,2}$ = 1.6 Hz, 1H, H-2), 3.53 (s, 3H, CH_{3Me}), 3.47 (d, *J* = 9.9 Hz, 1H, O*H*), 2.89 (d, *J* = 11.6 Hz, 1H, O*H*), 1.30 (d, *J* = 6.5 Hz, 3H, CH_{3Tal}); ^{13}C NMR (100 MHz, $CDCl_3$) δ 159.2 (C-Ar), 133.8 (C-2_{All}), 130.2 (C-Ar), 129.3 (*C*H-Ar), 117.3 (C-3_{All}), 113.8 (*C*H-Ar), 96.9 (C-1), 78.7 (C-2), 73.3 (C-3), 70.4 (C-4), 69.6 (*C*H_{2PMB}), 68.1 (C-1_{All}), 67.6 (C-5), 59.9 (*C*H_{3Me}), 55.3 (*C*H_{3PMB}), 16.6 (*C*H_{3Tal}); HRMS (ESI-TOF) *m/z* [M + Na]$^+$ calcd for $C_{18}H_{26}NaO_6$ 361.1622; found 361.1626. Analytical data for **S8**: R_f 0.2 (PE/EtOAc 7:3); $[\alpha]_D^{20}$ = –50 (*c* 4.3, $CHCl_3$); ^{1}H NMR (400 MHz, $CDCl_3$) δ 7.32–7.29 (m, 2H, C*H*-Ar), 6.88–6.85 (m, 2H, C*H*-Ar), 5.94–5.84 (m, 1H, H-2_{All}), 5.27 (ddd, *J* = 17.2, 3.8, 1.6 Hz, 1H, H-$3a_{All}$), 5.18 (ddd, *J* = 10.3, 3.3, 1.3 Hz, 1H, H-$3b_{All}$), 4.97 (d, $J_{1,2}$ = 1.1 Hz, 1H, H-1), 4.72 (d, *J* = 11.8 Hz, 1H, C*H*H_{PMB}), 4.57 (d, *J* = 11.8 Hz, 1H, CHH_{PMB}), 4.14 (ddt, *J* = 13.1, 5.1, 1.8 Hz, 1H, H-$1a_{All}$), 3.97 (ddt, *J* = 12.9, 6.0, 1.7 Hz, 1H, H-$1b_{All}$), 3.87–3.81 (m, 2H, H-5, H-3), 3.80 (s, 3H, CH_{3PMB}), 3.46 (s, 3H, CH_{3Me}), 3.45 (d, *J* = 1.7 Hz, 1H, H-4), 3.27 (dd, *J* = 4.5, 6.3 Hz, 1H, H-2), 2.89 (d, *J* = 11.6 Hz, 1H, O*H*), 1.19 (d, *J* = 6.5 Hz, 3H, CH_{3Tal}); ^{13}C NMR (100 MHz, $CDCl_3$) δ 159.4 (C-Ar), 134.0 (C-2_{All}), 130.7 (C-Ar), 130.1 (*C*H-Ar), 117.2 (C-3_{All}), 113.8 (*C*H-Ar), 96.3 (C-1), 78.8 (C-2), 78.4 (C-4), 75.7 (*C*H_{2PMB}), 68.0 (C-1_{All}), 66.7 (C-5), 65.9 (C-3), 59.9 (*C*H_{3Me}), 55.3 (*C*H_{3PMB}), 16.9 (*C*H_{3Tal}).

Allyl 4-*O*-Acetyl-6-deoxy-3-*O*-*para*-methoxybenzyl-2-*O*-methyl-*α*-L-talopyranoside (S10).

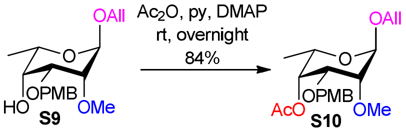

Alcohol **S9** (751 mg, 2.2 mmol, 1.0 equiv) was dissolved in anhydrous py (3 mL). Ac_2O (6 mL) and DMAP (27 mg, 222 *µ*mol, 0.1 equiv) were added. The reaction mixture was stirred overnight at rt under Ar. The mixture was then concentrated under reduced pressure and co-evaporated with toluene (3 ×). The residue was purified by silica gel flash chromatography (PE/EtOAc 9:1 to 7:3) to give derivative **S10** (714 mg, 84%) as a yellow amorphous solid: R_f 0.2 (tol/EtOAc 7:3); $[\alpha]_D^{20}$ = –107 (*c* 1.2, $CHCl_3$); ^{1}H NMR (400 MHz, $CDCl_3$) δ 7.27–7.25 (m, 2H, C*H*-Ar), 6.88–6.86 (m, 2H, C*H*-Ar), 5.93–5.83 (m, 1H, H-2_{All}), 5.31 (t, *J* = 2.1 Hz, 1H, H-4), 5.25 (ddd, *J* = 17.2, 3.8, 1.6 Hz, 1H, H-$3a_{All}$), 5.19 (ddd, *J* = 10.4, 3.3, 1.3 Hz, 1H, H-$3b_{All}$), 4.97 (d, $J_{1,2}$ = 1.2 Hz, 1H, H-1), 4.67 (d, *J* = 11.8 Hz, 1H, C*H*H_{PMB}), 4.56 (d, *J* = 11.8 Hz, 1H, CHH_{PMB}), 4.14 (ddt, *J* = 13.0, 5.1, 1.8 Hz, 1H, H-$1a_{All}$), 3.99–3.93 (m, 2H, H-$1b_{All}$, H-5), 3.80 (s, 3H, CH_{3PMB}), 3.75 (t, *J* = 3.8 Hz, 1H, H-3), 3.53 (s, 3H, CH_{3Me}), 3.41 (dt, *J* = 3.6, 1.5 Hz, 1H, H-2), 2.20 (s, 3H, CH_{3Ac}), 1.20 (d, *J* = 6.5 Hz, 3H, CH_{3Tal}); ^{13}C NMR (100 MHz, $CDCl_3$) δ 171.5, (*C*O), 159.3 (C-Ar), 133.8 (C-2_{All}), 130.2 (C-Ar), 129.3 (*C*H-Ar), 117.5 (C-3_{All}), 113.8 (*C*H-Ar), 97.8 (C-1), 77.2 (C-2), 73.0 (C-3), 70.4 (*C*H_{2PMB}), 69.1 (C-4), 68.2 (C-1_{All}), 65.1 (C-5), 60.1 (*C*H_{3Me}), 55.3 (*C*H_{3PMB}), 21.3, (*C*H_{3Ac}), 16.4 (*C*H_{3Tal}); HRMS (ESI-TOF) *m/z* $[M + Na]^+$ calcd for $C_{20}H_{28}NaO_7$ 403.1727; found 403.1738; *m/z* $[M + K]^+$ calcd for $C_{20}H_{28}KO_7$ 419.1467; found 419.1462; *m/z* $[2M + Na]^+$ calcd for $C_{40}H_{56}NaO_{14}$ 783.3562; found 783.3566.

Allyl 6-Deoxy-3-*O*-*para*-methoxybenzyl-*α*-L-talopyranoside (**S11**).

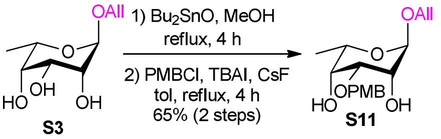

A suspension of Bu_2SnO (1.0 g, 4.0 mmol, 1.1 equiv) and triol **S3** (750 mg, 3.7 mmol, 1.0 equiv) was refluxed in MeOH (37 mL) for 4 h using a Dean-Stark apparatus. Then, the solvents were concentrated under reduced pressure and co-evaporated with toluene (3 ×). The residue was dissolved in toluene (19 mL). CsF (569 mg, 3.7 mmol, 1.02 equiv), TBAI (587 mg, 3.7 mmol, 1.05 equiv) and PMBCl (523 μL, 3.9 mmol, 1.05 equiv) were successively added and the reaction was refluxed for an additional 4 h. The mixture was then concentrated under reduced pressure. The residue was purified by silica gel flash chromatography (DCM/MeOH 97:3) to give diol **S11** (769 mg, 65%) as a yellow oil, which solidified upon standing at rt: R_f 0.6 (DCM/MeOH 95:5); $[\alpha]_D^{20}$ = –72 (*c* 1.2, $CHCl_3$); ^1H NMR (400 MHz, $CDCl_3$) δ 7.33–7.29 (m, 2H, C*H*-Ar), 6.90–6.87 (m, 2H, C*H*-Ar), 5.93–5.83 (m, 1H, H-2_{All}), 5.26 (ddd, J = 17.2, 3.7, 1.9 Hz, 1H, H-$3a_{All}$), 5.19 (ddd, J = 10.4, 3.5, 1.9 Hz, 1H, H-$3b_{All}$), 4.91 (d, $J_{1,2}$ = 1.5 Hz, 1H, H-1), 4.62 (d, J = 11.2 Hz, 1H, C*H*H_{PMB}), 4.58 (d, J = 11.2 Hz, 1H, CHH_{PMB}), 4.15 (ddt, J = 12.9, 5.2, 1.5 Hz, 1H, H-$1a_{All}$), 3.99 (ddt, J = 13.0, 6.0, 1.3 Hz, 1H, H-$1b_{All}$), 3.93–3.89 (m, 1H, H-2), 3.86 (dd, J = 13.9, 6.7 Hz, 1H, H-5), 3.80 (s, 3H, CH_{3PMB}), 3.76–3.74 (m, 1H, H-4), 3.62 (t, J = 3.3 Hz, 1H, H-3), 3.46 (d, J = 7.2 Hz, 1H, O*H*), 3.05 (d, J = 6.2 Hz, 1H, O*H*), 1.30 (d, J = 6.6 Hz, 3H, CH_{3Tal}); ^{13}C NMR (100 MHz, $CDCl_3$) δ 159.5 (C-Ar), 133.9 (C-2_{All}), 129.9 (C-Ar), 129.8 (*C*H-Ar), 117.5 (C-3_{All}), 114.0 (*C*H-Ar), 99.9 (C-1), 73.0 (C-3), 70.7 (C-4), 69.6 (*C*H_{2PMB}), 68.5 (C-2), 68.2 (C-1_{All}), 66.4 (C-5), 55.4 (*C*H_{3PMB}), 16.6 (*C*H_{3Tal}); HRMS (ESI-TOF) *m/z* $[M + Na]^+$ calcd for $C_{17}H_{24}NaO_6$ 347.1465; found 347.1472; *m/z* $[M + K]^+$ calcd for $C_{17}H_{24}KO_6$ 363.1204; found 363.1202; *m/z* $[2M + Na]^+$ calcd for $C_{34}H_{48}NaO_{12}$ 671.3038; found 671.3044.

Allyl 6-Deoxy-3-*O*-methyl-*α*-L-talopyranoside (**S12**).

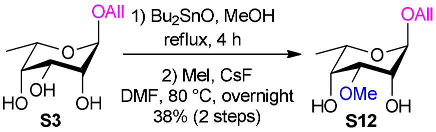

To a solution of triol **S3** (750 mg, 3.7 mmol, 1.0 equiv) in MeOH (37 mL) was added Bu_2SnO (1.0 g, 4.0 mmol, 1.1 equiv) and the mixture was refluxed for 4 h. Then, the solvents were concentrated under reduced pressure and co-evaporated with toluene (3 ×). The residue was dissolved in DMF (19 mL). CsF (569 mg, 3.7 mmol, 1.02 equiv) and MeI (23 mL, 367 mmol, 100 equiv) were sequentially added. After stirring overnight at 80 °C, the mixture was concentrated under reduced pressure and co-evaporated with toluene (3 ×). The residue was purified by silica gel flash chromatography (DCM/MeOH 98:2 to 97:3) to give diol **S12** (303 mg, 38%) as a yellow oil: R_f 0.2 (DCM/MeOH 97:3); $[\alpha]_D^{20}$ = –87 (*c* 1.4, $CHCl_3$); ^1H NMR (400 MHz, $CDCl_3$) δ 5.95–5.85 (m, 1H, H-2_{All}), 5.29 (ddd, *J* = 17.2, 3.7, 1.6 Hz, 1H, H-$3a_{All}$), 5.20 (ddd, *J* = 10.4, 3.4, 1.3 Hz, 1H, H-$3b_{All}$), 4.94 (d, $J_{1,2}$ = 0.9 Hz, 1H, H-1), 4.17 (ddt, *J* = 12.9, 5.3, 1.5 Hz, 1H, H-$1a_{All}$), 4.01 (ddt, *J* = 12.9, 6.1, 1.3 Hz, 1H, H-$1b_{All}$), 3.94–3.87 (m, 2H, H-2, H-5), 3.82 (br s, 1H, H-4), 3.53 (d, *J* = 6.0 Hz, 1H, O*H*), 3.47 (s, 3H, CH_{3Me}), 3.43 (t, *J* = 3.3 Hz, 1H, H-3), 1.32 (d, *J* = 6.5 Hz, 3H, CH_{3Tal}); ^{13}C NMR (100 MHz, $CDCl_3$) δ 133.9 (C-2_{All}), 117.7 (C-3_{All}), 99.9 (C-1), 75.3 (C-3), 70.2 (C-4), 68.3 (C-1_{All}), 68.1 (C-2), 66.4 (C-5), 55.7 (CH_{3Me}), 16.6 (CH_{3Tal}); HRMS (ESI-TOF) *m/z* $[M + Na]^+$ calcd for $C_{10}H_{18}NaO_5$ 241.1046; found 241.1054; *m/z* $[2M + Na]^+$ calcd for $C_{20}H_{36}NaO_{10}$ 459.2201; found 459.2201.

Allyl 4-*O*-Benzyl-6-deoxy-3-*O*-*para*-methoxybenzyl-*α*-L-talopyranoside (S13).

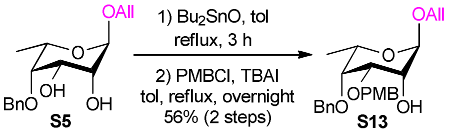

To a solution of diol **S5** (500 mg, 1.7 mmol, 1.0 equiv) in toluene (20 mL) was added Bu_2SnO (444 mg, 1.8 mmol, 1.05 equiv) and the mixture was refluxed using a Dean-Stark apparatus for 3 h. The temperature was cooled to 30 °C, then TBAI (659 mg, 1.8 mmol, 1.05 equiv) and PMBCl (253 *μ*L, 1.9 mmol, 1.1 equiv) were successively added. After refluxing overnight, the mixture was concentrated under reduced pressure. Purification by silica gel flash chromatography (PE/EtOAc 9:1 to 8:2) gave alcohol **S13** (398 mg, 56%) as a yellow oil: R_f 0.4 (PE/EtOAc 7:3); $[\alpha]_D^{20}$ = –38 (*c* 2.0, $CHCl_3$); ^{1}H NMR (400 MHz, $CDCl_3$) δ 7.38–7.24 (m, 7H, C*H*-Ar), 6.92–6.87 (m, 2H, C*H*-Ar), 5.92–5.82 (m, 1H, H-2_{All}), 5.24 (ddd, *J* = 17.2, 3.7, 1.5 Hz, 1H, H-$3a_{All}$), 5.17 (ddd, *J* = 10.4, 3.5, 1.3 Hz, 1H, H-$3b_{All}$), 4.99 (d, *J* = 11.1 Hz, 1H, CH*H*Ph), 4.92 (d, $J_{1,2}$ = 0.8 Hz, 1H, H-1), 4.76 (d, *J* = 11.4 Hz, 1H, C*H*H_{PMB}), 4.61 (d, *J* = 11.1 Hz, 1H, CH*H*Ph), 4.49 (d, *J* = 11.4 Hz, 1H, C*H*H_{PMB}), 4.23 (d, *J* = 10.3 Hz, 1H, O*H*), 4.13 (ddt, *J* = 12.9, 5.1, 1.6 Hz, 1H, H-$1a_{All}$), 4.01–3.95 (m, 2H, H-$1b_{All}$, H-2), 3.85 (dd, *J* = 13.1, 6.5 Hz, 1H, H-5), 3.82 (s, 3H, CH_{3PMB}), 3.75 (t, *J* = 3.3 Hz, 1H, H-3), 3.64 (t, *J* = 1.5 Hz, 1H, H-4), 1.20 (d, *J* = 6.5 Hz, 3H, CH_{3Tal}); ^{13}C NMR (100 MHz, $CDCl_3$) δ 159.2, 137.7 (2 × C-Ar), 133.9 (C-2_{All}), 130.4 (C-Ar), 129.2–127.9 (*C*H-Ar), 117.1 (C-3_{All}), 113.8 (*C*H-Ar), 100.8 (C-1), 78.9 (C-4), 75.5 (*C*H_2Ph), 74.1 (C-3), 69.5 (*C*H_{2PMB}), 68.1 (C-1_{All}), 68.0 (C-2), 66.5 (C-5), 55.3 (*C*H_{3PMB}), 16.8 (*C*H_{3Tal}); HRMS (ESI-TOF) *m/z* $[M + H]^+$ calcd for $C_{24}H_{31}O_6$ 415.2115; found 415.2109; *m/z* $[M + NH_4]^+$ calcd for $C_{24}H_{34}NO_6$ 432.2380; found 432.2379; *m/z* $[M + Na]^+$ calcd for $C_{24}H_{30}NaO_6$ 437.1934; found 437.1931.

Allyl 4-*O*-Benzyl-6-deoxy-3-*O*-methyl-*α*-L-talopyranoside (**S14**).

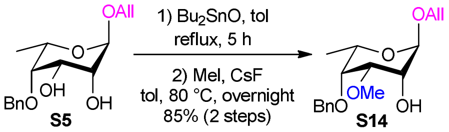

To a solution of diol **S5** (500 mg, 1.7 mmol, 1.0 equiv) in toluene (7 mL) was added Bu_2SnO (465 mg, 1.9 mmol, 1.1 equiv) and the mixture was refluxed using a Dean-Stark apparatus for 5 h. The temperature was cooled to 30 °C, then CsF (263 mg, 1.7 mmol, 1.02 equiv) and MeI (11 mL, 170 mmol, 100 equiv) were successively added. After stirring overnight at 80 °C, the mixture was concentrated under reduced pressure. Purification by silica gel flash chromatography (PE/EtOAc 9:1 to 8:2) gave alcohol **S14** (446 mg, 85%) as a yellow oil: R_f 0.4 (PE/EtOAc 7:3); $[\alpha]_D^{20}$ = –52 (*c* 1.3, $CHCl_3$); ^1H NMR (400 MHz, $CDCl_3$) δ 7.39–7.27 (m, 5H, C*H*-Ar), 5.94–5.84 (m, 1H, H-2_{All}), 5.28 (ddd, J = 17.2, 3.7, 1.6 Hz, 1H, H-$3a_{All}$), 5.19 (ddd, J = 10.4, 3.2, 1.3 Hz, 1H, H-$3b_{All}$), 4.98 (d, J = 11.1 Hz, 1H, C*H*HPh), 4.93 (d, $J_{1,2}$ = 1.7 Hz, 1H, H-1), 4.61 (d, J = 11.1 Hz, 1H, C*H*HPh), 4.15–4.11 (m, 2H, OH, H-$1a_{All}$), 4.01 (ddt, J = 13.0, 6.1, 1.3 Hz, 1H, H-$1b_{All}$), 3.96–3.92 (m, 1H, H-2), 3.87 (dd, J = 13.8, 6.5 Hz, 1H, H-5), 3.69–3.68 (m, 1H, H-4), 3.54 (t, J = 3.2 Hz, 1H, H-3), 3.48 (s, 3H, CH_{3Me}), 1.22 (d, J = 6.5 Hz, 3H, CH_{3Tal}); ^{13}C NMR (100 MHz, $CDCl_3$) δ 137.8 (C-Ar), 134.0 (C-2_{All}), 128.5-128.1 (*C*H-Ar), 117.5 (C-3_{All}), 100.9 (C-1), 78.2 (C-4), 76.6 (C-3), 75.6 (*C*H_2Ph), 68.3 (C-1_{All}), 67.9 (C-2), 66.6 (C-5), 55.9 (*C*H_{3Me}), 17.0 (*C*H_{3Tal}); HRMS (ESI-TOF) *m/z* $[M + Na]^+$ calcd for $C_{17}H_{24}NaO_5$ 331.1516; found 331.1519; *m/z* $[2M + Na]^+$ calcd for $C_{34}H_{48}NaO_{10}$ 639.3140; found 639.3138.

Allyl 2,4-*O*-Di-acetyl-6-deoxy-3-*O*-*para*-methoxybenzyl-*α*-L-talopyranoside (S15).

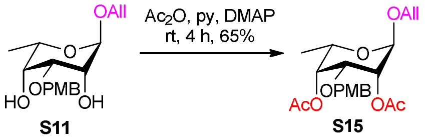

Diol **S11** (766 mg, 2.4 mmol, 1.0 equiv) was dissolved in anhydrous py (12 mL). Ac_2O (12 mL) and DMAP (29 mg, 240 *µ*mol, 0.1 equiv) were added. The reaction mixture was stirred for 4 h at rt under Ar. Then, the mixture was concentrated under reduced pressure and co-evaporated with toluene (3 ×). The residue was purified by silica gel flash chromatography (DCM/MeOH 1:0) to give derivative **S15** (632 mg, 65%) as a white amorphous solid: R_f 0.8 (DCM/MeOH 96:4); $[\alpha]_D^{20}$ = –64 (*c* 1.3, $CHCl_3$); ^1H NMR (400 MHz, $CDCl_3$) δ 7.24–7.22 (m, 2H, C*H*-Ar), 6.87–6.85 (m, 2H, C*H*-Ar), 5.91–5.81 (m, 1H, H-2_{All}), 5.28–2.18 (m, 4H, H-$3a_{All}$, H-$3b_{All}$, H-4, H-2), 5.22-5.18 (m, 2H, H-2, H-$3b_{All}$), 4.88 (d, $J_{1,2}$ = 0.9 Hz, 1H, H-1), 4.53 (d, *J* = 11.8 Hz, 1H, C*H*H_{PMB}), 4.49 (d, *J* = 11.8 Hz, 1H, CHH_{PMB}), 4.12 (ddt, *J* = 13.1, 5.2, 1.9 Hz, 1H, H-$1a_{All}$), 4.03 (dd, *J* = 6.6, 1.2 Hz, 1H, H-5), 3.97 (ddt, *J* = 12.7, 6.1, 1.8 Hz, 1H, H-$1b_{All}$), 3.80 (s, 3H, CH_{3PMB}), 3.78 (t, *J* = 3.9 Hz, 1H, H-3), 2.16 (s, 3H, CH_{3Ac}), 2.12 (s, 3H, CH_{3Ac}), 1.19 (d, *J* = 6.5 Hz, 3H, CH_{3Tal}); ^{13}C NMR (100 MHz, $CDCl_3$) δ 170.9, 170.6 (2 × CO), 159.3 (C-Ar), 133.5 (C-2_{All}), 130.0 (C-Ar), 129.2 (*C*H-Ar), 117.9 (C-3_{All}), 113.8 (*C*H-Ar), 97.8 (C-1), 70.5 (C-3), 70.3 (*C*H_{2PMB}), 69.1 (C-4), 68.4 (C-1_{All}), 67.3 (C-2), 65.1 (C-5), 55.4 (*C*H_{3PMB}), 21.3, 21.1(2 × *C*H_{3Ac}), 16.4 (*C*H_{3Tal}); HRMS (ESI-TOF) *m/z* $[M + Na]^+$ calcd for $C_{21}H_{28}NaO_8$ 431.1676; found 431.1690; *m/z* $[2M + Na]^+$ calcd for $C_{42}H_{56}NaO_{16}$ 839.3461; found 839.3468.

Allyl 2,4-*O*-Di-acetyl-6-deoxy-3-*O*-methyl-*α*-L-talopyranoside (**S16**).

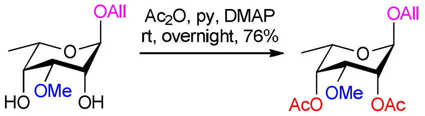

Diol **S12** (319 mg, 1.5 mmol, 1.0 equiv) was dissolved in anhydrous py (7 mL). Ac_2O (7 mL) and DMAP (18 mg, 150 μmol, 0.1 equiv) were added. The reaction mixture was stirred overnight at rt under Ar. Then, the mixture was concentrated under reduced pressure and co-evaporated with toluene (3 ×). The residue was purified by silica gel flash chromatography (PE/EtOAc 85:15 to 75:15) to give derivative **S16** (338 mg, 76%) as a yellow oil: R_f 0.8 (PE/EtOAc 7:3); $[\alpha]_D^{20}$ = –69 (*c* 1.6, $CHCl_3$); ^1H NMR (400 MHz, $CDCl_3$) δ 5.94–5.84 (m, 1H, H-2_{All}), 5.30 (ddd, *J* = 17.1, 3.5, 1.6 Hz, 1H, H-$3a_{All}$), 5.26 (d, *J* = 3.6 Hz, 1H, H-4), 5.23 (ddd, *J* = 10.4, 3.2, 1.3 Hz, 1H, H-$3b_{All}$), 5.18 (dt, *J* = 3.8, 1.5 Hz, 1H, H-2), 4.88 (d, $J_{1,2}$ = 1.1 Hz, 1H, H-1), 4.15 (ddt, *J* = 12.7, 5.3, 1.7 Hz, 1H, H-$1a_{All}$), 4.05 (ddd, *J* = 12.5, 6.6, 1.2 Hz, 1H, H-5), 4.00 (ddt, *J* = 12.4, 6.2, 1.6 Hz, 1H, H-$1b_{All}$), 3.64 (t, *J* = 3.9 Hz, 1H, H-3), 3.37 (s, 3H, CH_{3Me}), 2.17 (s, 3H, CH_{3Ac}), 2.14 (s, 3H, CH_{3Ac}), 1.20 (d, *J* = 6.5 Hz, 3H, CH_{3Tal}); ^{13}C NMR (100 MHz, $CDCl_3$) δ 170.9, 170.6 (2 × *C*O), 133.4 (C-2_{All}), 118.1 (C-3_{All}), 97.8 (C-1), 73.7 (C-3), 68.6 (C-1_{All}), 68.5 (C-4), 67.1 (C-2), 65.2 (C-5), 57.3 (CH_{3Me}), 21.3, 21.1 (2 × CH_{3Ac}), 16.4 (CH_{3Tal}); HRMS (ESI-TOF) *m/z* $[M + Na]^+$ calcd for $C_{14}H_{22}NaO_7$ 325.1258; found 325.1261.

Allyl 2-*O*-Acetyl-4-*O*-benzyl-6-deoxy-3-*O*-*para*-methoxybenzyl-*α*-L-talopyranoside (**S6**).

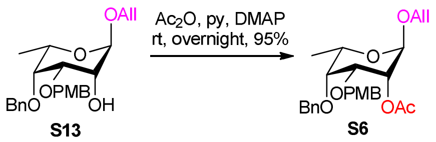

Alcohol **S13** (381 mg, 920 *μ*mol, 1.0 equiv) was dissolved in anhydrous py (3 mL). Ac_2O (3 mL) and DMAP (11 mg, 90 *μ*mol, 0.1 equiv) were added. The reaction mixture was stirred at rt overnight under Ar. Then, solvents were concentrated under reduced pressure and co-evaporated with toluene (3 ×). The residue was purified by silica gel flash chromatography (PE/EtOAc 9:1 to 8:2) to give derivative **S6** (397 mg, 95%) as a colorless oil: R_f 0.5 (PE/EtOAc 7:3); $[\alpha]_D^{20}$ = –15 (*c* 0.92, $CHCl_3$); ^{1}H NMR (400 MHz, $CDCl_3$) *δ* 7.37–7.23 (m, 7H, C*H*-Ar), 6.89–6.87 (m, 2H, C*H*-Ar), 5.92–5.82 (m, 1H, H-2_{All}), 5.34 (d, *J* = 3.5 Hz, 1H, H-2), 5.25 (ddd, *J* = 17.2, 3.8, 1.7 Hz, 1H, H-$3a_{All}$), 5.17 (ddd, *J* = 10.3, 3.4, 1.4 Hz, 1H, H-$3b_{All}$), 4.92 (d, *J* = 11.6 Hz, 1H, CH*H*Ph), 4.89 (s, 1H, H-1), 4.68 (d, *J* = 11.6 Hz, 1H, C*H*HPh), 4.67 (d, *J* = 11.5 Hz, 1H, CHH_{PMB}), 4.42 (d, *J* = 11.5 Hz, 1H, CHH_{PMB}), 4.12 (dd, *J* = 13.0, 6.4 Hz, 1H, H-$1a_{All}$), 3.97 (dd, *J* = 13.0, 6.0 Hz, 1H, H-$1b_{All}$), 3.89 (dd, *J* = 14.2, 6.5 Hz, 1H, H-5), 3.81 (s, 3H, CH_{3PMB}), 3.78 (t, *J* = 4.1 Hz, 1H, H-3), 3.52 (s, 1H, H-4), 2.08 (s, 3H, CH_{3Ac}), 1.26 (d, *J* = 6.5 Hz, 3H, CH_{3Tal}); ^{13}C NMR (100 MHz, $CDCl_3$) *δ* 171.1 (*C*O), 159.2, 139.1 (2 × C-Ar), 133.8 (C-2_{All}), 130.4 (C-Ar), 129.2–127.4 (*C*H-Ar), 117.5 (C-3_{All}), 113.8 (*C*H-Ar), 97.9 (C-1), 75.8 (C-4), 75.0 (C-3), 74.1 (*C*H_2Ph), 70.7 (*C*H_{2PMB}), 68.2 (C-1_{All}), 67.2 (C-2, C-5), 55.4 (*C*H_{3PMB}), 21.4 (*C*H_{3Ac}), 16.9 (*C*H_{3Tal}); HRMS (ESI-TOF) *m/z* $[M + H]^+$ calcd for $C_{26}H_{33}O_7$ 457.2221; found 457.2218; *m/z* $[M + NH_4]^+$ calcd for $C_{26}H_{36}NO_7$ 474.2486; found 474.2487; *m/z* $[M + Na]^+$ calcd for $C_{26}H_{32}NaO_7$ 479.2040; found 479.2041.

Allyl 2-*O*-Acetyl-4-*O*-benzyl-6-deoxy-3-*O*-methyl-*α*-L-talopyranoside (**S17**).

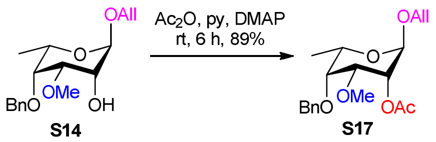

Alcohol **S14** (430 mg, 1.4 mmol, 1.0 equiv) was dissolved in anhydrous py (4 mL). Ac_2O (4 mL) and DMAP (17 mg, 139 *µ*mol, 0.1 equiv) were added. The reaction mixture was stirred for 6 h at rt under Ar. Then, solvents were concentrated under reduced pressure and co-evaporated with toluene (3 ×). The residue was purified by silica gel flash chromatography (PE/EtOAc 9:1) to give derivative **S17** (432 mg, 89%) as a yellow oil: R_f 0.5 (PE/EtOAc 8:2); $[\alpha]_D^{20}$ = –20 (*c* 1.5, $CHCl_3$); ^{1}H NMR (400 MHz, $CDCl_3$) *δ* 7.41–7.25 (m, 5H, C*H*-Ar), 5.93–5.83 (m, 1H, H-2_{All}), 5.29-5.27 (m, 1H, H-2), 5.28 (ddd, *J* = 17.2, 3.8, 1.5 Hz, 1H, H-$3a_{All}$), 5.19 (ddd, *J* = 10.4, 3.3, 1.2 Hz, 1H, H-$3b_{All}$), 4.92 (d, *J* = 11.8 Hz, 1H, CH*H*Ph), 4.88 (d, $J_{1,2}$ = 1.3 Hz, 1H, H-1), 4.65 (d, *J* = 11.8 Hz, 1H, CH*H*Ph), 4.14 (ddt, *J* = 12.8, 5.2, 1.7 Hz, 1H, H-$1a_{All}$), 3.98 (ddt, *J* = 13.1, 6.1, 1.7 Hz, 1H, H-$1b_{All}$), 3.92 (dd, *J* = 14.1, 6.3 Hz, 1H, H-5), 3.61–3.59 (m, 2H, H-3, H-4), 3.41 (s, 3H, CH_{3Me}), 2.09 (s, 3H, CH_{3Ac}), 1.28 (d, *J* = 6.5 Hz, 3H, CH_{3Tal}); ^{13}C NMR (100 MHz, $CDCl_3$) *δ* 171.1, (*C*O), 139.3 (C-Ar), 133.8 (C-2_{All}), 128.2, 128.1, 127.4 (3 × *C*H-Ar), 117.7 (C-3_{All}), 97.9 (C-1), 77.8 (C-3), 75.4 (C-4), 74.0 (*C*H_2Ph), 68.3 (C-1_{All}), 67.2 (C-5), 67.1 (C-2), 57.2 (*C*H_{3Me}), 21.3 (*C*H_{3Ac}), 16.9 (*C*H_{3Tal}); HRMS (ESI-TOF) *m/z* $[M + Na]^+$ calcd for $C_{19}H_{26}NaO_6$ 373.1622; found 373.1624; *m/z* $[M + K]^+$ calcd for $C_{19}H_{26}KO_6$ 389.1361; found 389.1360.

2-*O*-Acetyl-4-*O*-benzyl-6-deoxy-3-*O*-*para*-methoxybenzyl-*α*-L-talopyranosyl 2,2,2-Trichloroacetimidate (8).

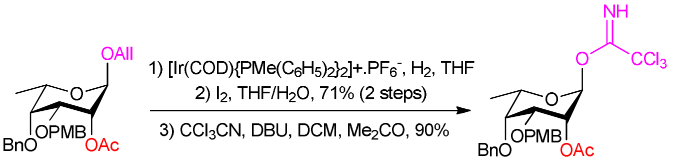

S6 **8**

Allyl taloside **S6** (397 mg, 870 μmol, 1.0 equiv) was reacted according to the general procedure for the synthesis of trichloroacetimidate donors (first part). Purification by silica gel flash chromatography (PE/EtOAc 9:1 to 7:3) gave a hemiacetal (258 mg, 71%, ratio α/β ~ 3:1) as a yellow oil: R_f 0.4 (PE/EtOAc 8:2); ^{1}H NMR (400 MHz, $CDCl_3$) δ 7.37–7.25 (m, 8H, C*H*-Ar), 6.90–6.87 (m, 2H, C*H*-Ar), 5.32–5.31 (m, 1H, H-2), 5.29 (s, 1H, H-1), 4.91 (d, *J* = 11.8 Hz, 1H, C*H*HPh), 4.69 (d, *J* = 11.8 Hz, 1H, CH*H*Ph), 4.67 (d, *J* = 11.5 Hz, 1H, C*H*H_{PMB}), 4.45 (d, *J* = 11.5 Hz, 1H, C*H*H_{PMB}), 4.14 (ddd, *J* = 13.7, 6.5, 1.3 Hz, 1H, H-5), 3.84 (t, *J* = 3.5 Hz, 1H, H-3), 3.81 (s, 3H, CH_{3PMB}), 3.54 (t, *J* = 1.6 Hz, 1H, H-4), 2.76 (s, 1H, O*H*), 2.10 (s, 3H, CH_{3Ac}), 1.27 (d, *J* = 6.6 Hz, 3H, CH_{3Tal}); ^{13}C NMR (100 MHz, $CDCl_3$) δ 171.1 (*C*O), 159.3, 139.0, 130.4 (3 × C-Ar), 129.3, 128.4, 128.2, 127.6 (4 × *C*H-Ar), 113.9 (C-Ar), 93.6 (C-1), 75.8 (C-4), 74.5 (C-3), 73.9 (*C*H_2Ph), 70.8 (*C*H_{2PMB}), 67.6 (C-2), 67.5 (C-5), 55.4 (*C*H_{3PMB}), 21.4 (*C*H_{3Ac}), 16.9 (*C*H_{3Tal}); HRMS (ESI-TOF) *m/z* [M + NH_4]$^+$ calcd for $C_{23}H_{32}NO_7$ 434.2173; found 434.2169; *m/z* [M + Na]$^+$ calcd for $C_{23}H_{28}NaO_7$ 439.1727; found 439.1725. The hemiacetal (50 mg, 120 μmol, 1.0 equiv) was reacted in the presence of DBU (4 μL, 40 μmol, 0.3 equiv) and CCl_3CN (60 μL, 600 μmol, 5.0 equiv). Purification by silica gel flash chromatography (PE/EtOAc 85:15 to 8:2 + 1% Et_3N) gave imidate **8** (61 mg, 90%) as a colorless oil: R_f 0.5 (PE/EtOAc 6:4); $[\alpha]_D^{20}$ = +3.4 (*c* 0.80, $CHCl_3$); ^{1}H NMR (400 MHz, py-d_5) δ 7.54–7.48 (m, 7H, C*H*-Ar), 7.05–7.03 (m, 2H, C*H*-Ar), 6.83 (s, 1H, H-1), 5.88 (t, *J* = 1.8 Hz, 1H, H-2), 5.22 (d, *J* = 11.3 Hz, 1H, C*H*HPh), 4.91 (d, *J* = 11.3 Hz, 1H, C*H*HPh), 4.79 (d, *J* = 11.4 Hz, 1H, C*H*H_{PMB}), 4.73 (d, *J* = 11.4 Hz, 1H, C*H*H_{PMB}), 4.41 (dd, *J* = 14.3, 6.3 Hz, 1H, H-5), 4.25 (t, *J* = 3.6 Hz, 1H, H-3), 3.88 (t, *J* = 1.5 Hz, 1H, H-4), 3.69 (s, 3H, CH_{3PMB}), 2.03 (s, 3H, CH_{3Ac}), 1.43 (d, *J* = 6.5 Hz, 3H, CH_{3Tal}); ^{13}C NMR (100 MHz, py-d_5) δ 171.8 (CO), 160.2, 159.9 (2 × C-Ar), 130.4 (*C*H-Ar), 128.9, 128.7, 128.1 (3 × *C*H-Ar), 114.7 (*C*H-Ar), 97.3 (C-1), 76.8 (C-4), 75.0 (*C*H_2Ph), 74.8 (C-3), 71.2 (CH_{2PMB}, C-5), 66.4 (C-2), 55.5 (*C*H_{3PMB}), 21.3 (*C*H_{3Ac}), 17.3 (*C*H_{3Tal}); HRMS (ESI-TOF) *m/z* [M + NH_4]$^+$ calcd for $C_{25}H_{32}Cl_3N_2O_7$ 577.1269; found 577.1264; *m/z* [M + Na]$^+$ calcd for $C_{25}H_{28}Cl_3NNaO_7$ 582.0823; found 582.0822.

4-*O*-Acetyl-6-deoxy-3-*O*-*para*-methoxybenzyl-2-*O*-methyl-*α*-L-talopyranosyl 2,2,2-Trichloroacmetimidate (9).

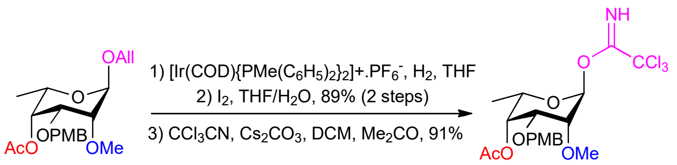

S10 **9**

Allyl taloside **S10** (829 mg, 2.2 mmol, 1.0 equiv) was reacted according to the general procedure for the synthesis of trichloroacetimidate donors (first part). Purification by silica gel flash chromatography (PE/EtOAc 85:15 to 5:5) gave a hemiacetal (658 mg, 89%, ratio α/β ~ 3:1) as a yellow amorphous solid: R_f 0.6 (DCM/MeOH 95:5); ^{1}H NMR (400 MHz, $CDCl_3$) δ 7.28–7.24 (m, 4H, C*H*-Ar), 6.90–6.87 (m, 4H, C*H*-Ar), 5.35 (dd, J = 3.4, 1.4 Hz, 1H, H-1α), 5.31 (t, J = 1.7 Hz, 1H, H-4α), 5.29–5.27 (m, 1H, H-4β), 4.70 (d, J = 11.5 Hz, 1H, C*H*HPh), 4.68 (d, J = 11.8 Hz, 1H, C*H*HPh), 4.58 (d, J = 12.6 Hz, 1H, H-1β), 4.46 (d, J = 11.8 Hz, 1H, C*H*HPh), 4.42 (d, J = 11.5 Hz, 1H, C*H*HPh), 4.22 (ddd, J = 13.6, 6.5, 1.5 Hz, 1H, H-5α), 4.09 (d, J = 12.6 Hz, 1H, O*H*), 3.81 (s, 3H, CH_{3PMB}), 3.80 (s, 3H, CH_{3PMB}), 3.79 (t, J = 3.8 Hz, 1H, H-3α), 3.66 (s, 3H, CH_{3Me}), 3.61 (ddd, J = 13.8, 6.5, 1.5 Hz, 1H, H-5β), 3.54 (s, 3H, CH_{3Me}), 3.50 (s, 1H, H-3β), 3.49 (d, J = 0.6 Hz, 1H, H-2β), 3.43–3.41 (m, 1H, H-2α), 2.94 (d, J = 3.4 Hz, 1H, O*H*), 2.21, 2.20 (2 × s, 6H, 2 × CH_{3Ac}), 1.26 (d, J = 6.5 Hz, 3H, CH_{3Tal}), 1.20 (d, J = 6.6 Hz, 3H, CH_{3Tal}); ^{13}C NMR (100 MHz, $CDCl_3$) δ 171.5, 171.3 (2 × *C*O), 159.3, 159.1 (2 × C-Ar), 130.1, 129.8 (2 × C-Ar), 129.4, 129.3 (2 × *C*H-Ar), 114.0, 113.9 (2 × *C*H-Ar), 94.1 (C-1β), 93.4 (C-1α), 77.9 (C-2β), 77.3 (C-2α), 77.0 (C-3β), 72.4 (C-3α), 70.7 (CH_{2PMB}), 70.4 (CH_{2PMB}), 69.9 (C-5β), 69.1 (C-4α), 67.6 (C-4β), 65.2 (C-5α), 61.6, 60.1 (2 × CH_{3Me}), 55.4, 55.3 (2 × CH_{3PMB}), 21.3, 21.2 (2 × CH_{3Ac}), 16.5, 16.4 (2 × CH_{3Tal}); HRMS (ESI-TOF) *m/z* $[M + Na]^+$ calcd for $C_{17}H_{24}NaO_7$ 363.1414; found 363.1427. The hemiacetal (644 mg, 1.9 mmol, 1.0 equiv) was reacted in the presence of Cs_2CO_3 (123 mg, 380 μmol, 0.2 equiv) and CCl_3CN (950 μL, 9.5 mmol, 5.0 equiv). Purification by silica gel flash chromatography (PE/EtOAc 1:0 to 5:5 + 1% Et_3N) gave imidate **9** (834 mg, 91%) as a yellow oil: R_f 0.6 (PE/EtOAc 5:5 + 1% Et_3N); $[\alpha]_D^{20}$ = –46 (*c* 1.5, $CHCl_3$); ^{1}H NMR (400 MHz, py-d_5) δ 7.58–7.48 (m, 2H, C*H*-Ar), 7.02–6.99 (m, 2H, C*H*-Ar), 6.81 (s, 1H, H-1), 5.74 (br s, 1H, H-4), 4.89 (d, J = 11.4 Hz, 1H, C*H*H_{PMB}), 4.68 (d, J = 11.4 Hz, 1H, C*H*H_{PMB}), 4.50 (dd, J = 14.0, 6.3 Hz, 1H, H-5), 4.20 (t, J = 3.8 Hz, 1H, H-3), 3.89 (t, J = 1.9 Hz, 1H, H-2), 3.68 (s, 3H, CH_{3PMB}), 3.61 (s, 3H, CH_{3Me}), 2.15 (s, 3H, CH_{3Ac}), 1.32 (d, J = 6.6 Hz, 3H, CH_{3Tal}); ^{13}C NMR (100 MHz, py-d_5) δ 171.1 (*C*O), 159.9 (C-Ar), 159.4 (C_{imine}), 130.1, 114.3 (2 × *C*H-Ar), 97.3 (C-1), 75.3 (C-2), 73.1 (C-3), 70.5 (CH_{2PMB}), 68.9 (C-4, C-5), 59.5 (CH_{3Me}), 55.2 (CH_{3PMB}), 21.0 (CH_{3Ac}), 16.6 (CH_{3Tal}); HRMS (ESI-TOF) *m/z* $[M + Na]^+$ calcd for $C_{19}H_{24}Cl_3NNaO_7$ 506.0511; found 506.0511.

2,4-Di-*O*-acetyl-6-deoxy-3-*O*-*para*-methoxybenzyl-*α*-L-talopyranosyl 2,2,2-Trichloroacetimidate (10).

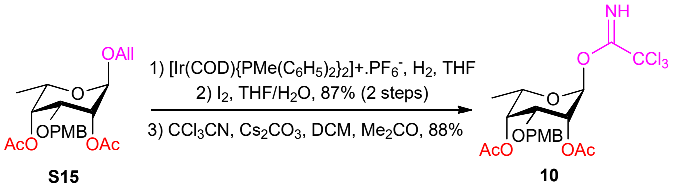

Allyl taloside **S15** (602 mg, 1.5 mmol, 1.0 equiv) was reacted according to the general procedure for the synthesis of trichloroacetimidate donors (first part). Purification by silica gel flash chromatography (DCM/MeOH 98:2 to 96:4) gave a hemiacetal (471 mg, 87%, ratio *α*/*β* ~ 3:1) as a white amorphous solid: R_f 0.5 (DCM/MeOH 95:5); ^{1}H NMR (400 MHz, $CDCl_3$) *δ* 7.25–7.22 (m, 4H, C*H*-Ar), 6.88–6.85 (m, 2H, C*H*-Ar), 5.32–5.31 (m, 1H, H-2), 5.26 (d, *J* = 2.4 Hz, 1H, H-1), 5.23 (d, *J* = 2.9 Hz, 1H, H-4), 5.17 (dt, *J* = 3.7, 1.4 Hz, 1H, H-2), 4.55 (d, *J* = 11.8 Hz, 1H, C*H*H_{PMB}), 4.49 (d, *J* = 11.8 Hz, 1H, C*H*H_{PMB}), 4.25 (ddd, *J* = 14.1, 6.6, 1.1 Hz, 1H, H-5), 3.83 (t, *J* = 3.9 Hz, 1H, H-3), 3.80 (s, 3H, CH_{3PMB}), 3.15 (d, *J* = 3.8 Hz, 1H, O*H*), 2.16, 2.13 (2 × s, 6H, 2 × CH_{3Ac}), 1.19 (d, *J* = 6.6 Hz, 3H, CH_{3Tal}); ^{13}C NMR (100 MHz, $CDCl_3$) *δ* 171.0, 170.7 (2 × *C*O), 159.3, 129.9 (2 × C-Ar), 129.2 (*C*H-Ar), 113.9 (C-Ar), 93.4 (C-1), 70.3 (*C*H_{2PMB}), 69.3 (C-3), 69.1 (C-4), 67.6 (C-2), 65.2 (C-5), 55.4 (*C*H_{3PMB}), 21.3, 21.1 (2 × *C*H_{3Ac}), 16.5 (*C*H_{3Tal}); HRMS (ESI-TOF) *m/z* [M + Na]$^+$ calcd for $C_{18}H_{24}NaO_8$ 391.1363; found 391.1376; *m/z* [2M + Na]$^+$ calcd for $C_{36}H_{48}NaO_{16}$ 759.2835; found 759.2841. The hemiacetal (455 mg, 1.2 mmol, 1.0 equiv) was reacted in the presence of Cs_2CO_3 (80 mg, 250 *µ*mol, 0.2 equiv) and CCl_3CN (620 *µ*L, 6.2 mmol, 5.0 equiv). Purification by silica gel flash chromatography (PE/EtOAc 1:0 to 7:3 + 1% Et_3N) gave imidate **10** (558 mg, 88%) as a yellow amorphous solid: R_f 0.5 (PE/EtOAc 7:3 + 1% Et_3N); $[\alpha]_D^{20}$ = –32 (*c* 1.8, $CHCl_3$); ^{1}H NMR (400 MHz, py-d_5) *δ* 7.58–7.46 (m, 2H, C*H*-Ar), 6.96–6.94 (m, 2H, C*H*-Ar), 6.83 (s, 1H, H-1), 5.81 (br s, 1H, H-2), 5.74 (t, *J* = 1.7 Hz, 1H, H-4), 4.82 (d, *J* = 11.6 Hz, 1H, C*H*H_{PMB}), 4.76 (d, *J* = 11.6 Hz, 1H, C*H*H_{PMB}), 4.55 (dd, *J* = 14.2, 6.5 Hz, 1H, H-5), 4.31 (t, *J* = 4.0 Hz, 1H, H-3), 3.64 (s, 3H, CH_{3PMB}), 2.23, 2.13 (2 × s, 6H, 2 × CH_{3Ac}), 1.33 (d, *J* = 6.6 Hz, 3H, CH_{3Tal}); ^{13}C NMR (100 MHz, py-d_5) *δ* 170.8, 170.2 (2 × CO), 159.9 (C-Ar), 159.4 (C_{imine}), 129.9, 114.3 (2 × *C*H-Ar), 96.7 (C-1), 70.7 (C-3), 70.6 (*C*H_{2PMB}), 68.9 (C-4), 68.8 (C-5), 66.0 (C-2), 55.1 (*C*H_{3PMB}), 20.9 (2 × *C*H_{3Ac}), 16.5 (*C*H_{3Tal}); HRMS (ESI-TOF) *m/z* [M + Na]$^+$ calcd for $C_{20}H_{24}Cl_3NNaO_8$ 534.0460; found 534.0467.

2-*O*-Acetyl-4-*O*-benzyl-6-deoxy-3-*O*-methyl-*α*-L-talopyranosyl 2,2,2-Trichloroacetimidate (11).

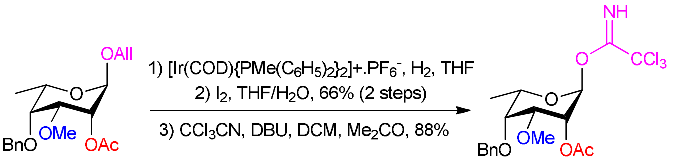

Allyl taloside **29** (413 mg, 1.2 mmol, 1.0 equiv) was reacted according to the general procedure for the synthesis of trichloroacetimidate donors (first part). Purification by silica gel flash chromatography (PE/EtOAc 85:5 to 8:2 + 1% Et_3N) gave a hemiacetal (242 mg, 66%, ratio *α*/*β* ~ 4:1) as a yellow oil: R_f 0.5 (PE/EtOAc 6:4); ^{1}H NMR (400 MHz, $CDCl_3$) *δ* 7.43–7.27 (m, 5H, C*H*-Ar), 5.29 (br s, 1H, H-1), 5.26 (dd, *J* = 3.6, 1.2 Hz, 1H, H-2), 4.90 (d, *J* = 11.8 Hz, 1H, C*H*HPh), 4.65 (d, *J* = 11.8 Hz, 1H, C*H*HPh), 4.17 (ddd, *J* = 13.8, 6.5, 1.4 Hz, 1H, H-5), 3.65 (t, *J* = 3.4 Hz, 1H, H-3), 3.62 (t, *J* = 1.6 Hz, 1H, H-4), 3.43 (s, 3H, CH_{3Me}), 2.91 (d, *J* = 3.7 Hz, 1H, O*H*), 2.11 (s, 3H, CH_{3Ac}), 1.29 (d, *J* = 6.5 Hz, 3H, CH_{3Tal}); ^{13}C NMR (100 MHz, $CDCl_3$) *δ* 171.2 (*C*O), 139.1 (C-Ar), 128.3, 128.2, 127.5 (3 × *C*H-Ar), 93.4 (C-1), 77.4 (C-3), 75.3 (C-4), 73.9 (CH_2Ph), 67.5 (C-2), 67.4 (C-5), 57.4 (CH_{3Me}), 21.4 (CH_{3Ac}), 17.0 (CH_{3Tal}); HRMS (ESI-TOF) *m/z* [M + Na]$^+$ calcd for $C_{16}H_{22}NaO_6$ 333.1309; found 333.1316; *m/z* [2M + Na]$^+$ calcd for $C_{32}H_{44}NaO_{12}$ 643.2725; found 643.2744. The hemiacetal (228 mg, 730 *μ*mol, 1.0 equiv) was reacted in the presence of Cs_2CO_3 (48 mg, 150 *μ*mol, 0.2 equiv) and CCl_3CN (370 *μ*L, 3.7 mmol, 5.0 equiv). Purification by silica gel flash chromatography (PE/EtOAc 1:0 to 4:6 + 1% Et_3N) gave imidate **11** (558 mg, 88%) as a yellow oil: R_f 0.7 (PE/EtOAc 7:3 + 1% Et_3N); $[\alpha]_D^{20}$ = –6.4 (*c* 1.4, $CHCl_3$); ^{1}H NMR (400 MHz, py-d_5) *δ* 7.61–7.16 (m, 5H, C*H*-Ar), 6.83 (s, 1H, H-1), 5.84 (t, *J* = 1.9 Hz, 1H, H-2), 5.14 (d, *J* = 11.4 Hz, 1H, C*H*HPh), 4.73 (d, *J* = 11.4 Hz, 1H, CH*H*Ph), 4.43 (dd, *J* = 14.0, 6.6 Hz, 1H, H-5), 3.97 (t, *J* = 3.7 Hz, 1H, H-3), 3.87–3.85 (m, 1H, H-4), 3.50 (s, 3H, CH_{3Me}), 2.00 (s, 3H, CH_{3Ac}), 1.43 (d, *J* = 6.5 Hz, 3H, CH_{3Tal}); ^{13}C NMR (100 MHz, py-d_5) *δ* 170.4 (*C*O), 159.2 (C-Ar), 128.9, 128.6, 127.8 (3 × *C*H-Ar), 96.9 (C-1), 77.7 (C-3), 76.0 (C-4), 74.6 (CH_2Ph), 70.7 (C-5), 65.7 (C-2), 57.2 (CH_{3Me}), 20.9 (CH_{3Ac}), 17.0 (CH_{3Tal}); HRMS (ESI-TOF) *m/z* [M + Na]$^+$ calcd for $C_{18}H_{22}Cl_3NNaO_6$ 476.0405; found 476.0417.

2,4-Di-*O*-acetyl-6-deoxy-3-*O*-methyl-*α*-L-talopyranosyl 2,2,2-Trichloroacetimidate (12).

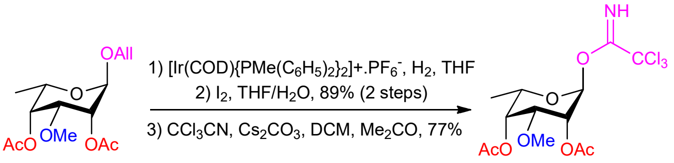

Allyl taloside **S16** (320 mg, 1.1 mmol, 1.0 equiv) was reacted according to the general procedure for the synthesis of trichloroacetimidate donors (first part). Purification by silica gel flash chromatography (PE/EtOAc 85:5 to 6:4 + 1% Et_3N) gave a hemiacetal (248 mg, 89%, ratio *α*/*β* ~ 3:1) as a yellow oil: R_f 0.2 (PE/EtOAc 6:4); ^{1}H NMR (400 MHz, $CDCl_3$) *δ* 5.28–5.27 (m, 2H, H-1, H-2), 5.18 (td, *J* = 3.8, 1.5 Hz, 1H, H-4), 4.30 (ddd, *J* = 13.7, 6.6, 1.2 Hz, 1H, H-5), 3.70 (t, *J* = 3.8 Hz, 1H, H-3), 3.38 (s, 3H, CH_{3Me}), 2.17, 2.15 (2 × s, 6H, 2 × CH_{3Ac}), 1.20 (d, *J* = 6.6 Hz, 3H, CH_{3Tal}); ^{13}C NMR (100 MHz, $CDCl_3$) *δ* 171.0, 170.8 (2 × *C*O), 93.2 (C-1), 73.3 (C-3), 68.5 (C-4), 67.4 (C-2), 65.2 (C-5), 57.3 (CH_{3Me}), 21.3, 21.1 (2 × CH_{3Ac}), 16.5 (CH_{3Tal}); HRMS (ESI-TOF) *m/z* $[M + Na]^+$ calcd for $C_{11}H_{18}NaO_7$ 285.0945; found 285.0950. The hemiacetal (234 mg, 890 *μ*mol, 1.0 equiv) was reacted in the presence of Cs_2CO_3 (58 mg, 180 *μ*mol, 0.2 equiv) and CCl_3CN (450 *μ*L, 4.5 mmol, 5.0 equiv). Purification by silica gel flash chromatography (PE/EtOAc 1:0 to 4:6 + 1% Et_3N) gave imidate **12** (281 mg, 77%) as a yellow oil: R_f 0.5 (PE/EtOAc 8:2 + 1% Et_3N); $[\alpha]_D^{20}$ = –18 (*c* 1.4, $CHCl_3$); ^{1}H NMR (400 MHz, py-d_5) *δ* 6.84 (s, 1H, H-1), 5.80 (d, *J* = 3.4 Hz, 1H, H-2), 5.68 (t, *J* = 1.7 Hz, 1H, H-4), 4.56 (dd, *J* = 14.5, 6.3 Hz, 1H, H-5), 4.06 (t, *J* = 3.9 Hz, 1H, H-3), 3.46 (s, 3H, CH_{3Me}), 2.21, 2.13 (2 × s, 6H, 2 × CH_{3Ac}), 1.32 (d, *J* = 6.6 Hz, 3H, CH_{3Tal}); ^{13}C NMR (100 MHz, py-d_5) *δ* 170.7, 170.1 (2 × CO), 159.1 (C_{imine}), 96.6 (C-1), 74.0 (C-3), 68.7 (C-5), 68.4 (C-4), 65.8 (C-2), 57.1 (CH_{3Me}), 20.9, 20.8 (2 × CH_{3Ac}), 16.5 (CH_{3Tal}).

(5-Azido-1-pentyl) 2,3,4,6-Tetra-*O*-acetyl-*β*-D-glucopyranoside (S20).

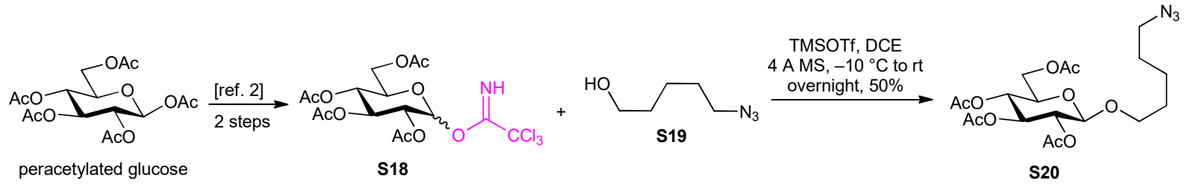

To a solution of glycosyl donor **S18**[2] (562 mg, 1.1 mmol, 1.0 equiv) and 5-azido-1-pentanol **S19**[3] (221 mg, 1.7 mmol, 1.5 equiv) in anhydrous DCE (11 mL) was added freshly activated 4 Å powdered molecular sieves (2.0 g). The mixture was stirred for 1 h at rt under Ar. Then, the reaction mixture was cooled to −10 °C and TMSOTf (60 *µ*L, 303 *µ*mol, 0.3 equiv) was added dropwise. The mixture was stirred from –10 °C to rt for 24 h under Ar. The reaction was quenched with Et_3N (100 *µ*L), filtered over Celite and rinsed with DCM. The filtrate was concentrated under reduced pressure and purified by silica gel flash chromatography (PE/EtOAc 9:1 to 7:3) to give glucoside **S20** (260 mg, 50%) as a colorless oil, which solidified upon standing at 4 °C: R_f 0.5 (PE/EtOAc 6:4); $[\alpha]_D^{20}$ = –12 (*c* 1.5, $CHCl_3$); ^1H NMR (400 MHz, $CDCl_3$) δ 5.20 (t, *J* = 9.7 Hz, 1H, H-3), 5.08 (t, *J* = 9.9 Hz, 1H, H-4), 4.98 (dd, *J* = 9.4, 8.0 Hz, 1H, H-2), 4.49 (d, *J* = 8.1 Hz, 1H, H-1), 4.26 (dd, *J* = 12.4 Hz, 4.7 Hz, 1H, H-6a), 4.14 (dd, *J* = 12.4 Hz, 2.6 Hz, 1H, H-6b), 3.88 (td, *J* = 9.6, 6.6, 6.1, 1H, H-$1a_{linker}$), 3.69 (ddd, *J* = 9.9, 4.8, 2.6 Hz, 1H, H-5), 3.49 (dt, *J* = 9.6, 7.1, 1H, H-$1b_{linker}$), 3.27 (t, *J* = 7.1 Hz, 2H, H-5_{linker}), 2.09, 2.05, 2.02, 2.01 (4 × s, 12H, 4 × CH_{3Ac}), 1.65–1.56 (m, 4H, H-2_{linker}, H-4_{linker}), 1.46–1.37 (m, 2H, H-3_{linker}); ^{13}C NMR (100 MHz, $CDCl_3$) δ 170.8, 170.4, 169.5, 169.4 (4 × *C*O), 100.9 (C-1), 72.9 (C-3), 71.9 (C-5), 71.4 (C-2), 69.8 (C-1_{linker}), 68.6 (C-4), 62.1 (C-6), 51.5 (C-5_{linker}), 29.1 (C-2_{linker}), 28.6 (C-4_{linker}), 23.3 (C-3_{linker}), 20.9, 20.8, 20.7, 20.6 (4 × CH_{3Ac}); HRMS (ESI-TOF) *m/z* $[M + H]^+$ calcd for $C_{19}H_{30}N_3O_{10}$ 460.1925; found 460.1926; *m/z* $[M + NH_4]^+$ calcd for $C_{19}H_{33}N_4O_{10}$ 477.2191; found 477.2192; *m/z* $[M + Na]^+$ calcd for $C_{19}H_{29}N_3NaO_{10}$ 482.1745; found 482.1746.

(5-Azido-1-pentyl) 4,6-*O*-Benzylidene-*β*-D-glucopyranoside (S21).

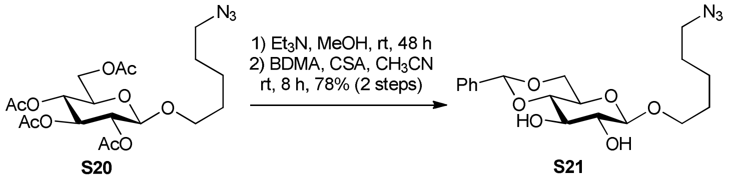

Glucoside **S20** (1.2 g, 2.6 mmol, 1.0 equiv) was dissolved in anhydrous MeOH (20 mL). Et_3N (2.1 mL, 15 mmol, 6.0 equiv) was added and the reaction mixture was stirred 48 h at rt under Ar. Then, the mixture was concentrated under reduced pressure and co-evaporated with toluene (3 ×). The residue was dissolved in anhydrous CH_3CN (10 mL) and BDMA (0.8 mL, 5.2 mmol, 2.0 equiv) followed by CSA (60 mg, 260 μmol, 0.1 equiv) were added. The mixture was stirred for 8 h at rt under Ar. Then, the reaction mixture was quenched with Et_3N (100 μL), concentrated under reduced pressure and purified by silica gel flash chromatography (PE/EtOAc 85:15 to 5:5) to give diol **S21** (776 mg, 78%, two steps) as a white amorphous solid: R_f 0.4 (DCM/MeOH 95:5); $[\alpha]_D^{20}$ = –25 (*c* 1.5, $CHCl_3$); ^{1}H NMR (400 MHz, $CDCl_3$) δ 7.50–7.48 (m, 2H, C*H*-Ar), 7.39–7.36 (m, 3H, C*H*-Ar), 5.53 (s, 1H, H-7), 4.39 (d, $J_{1,2}$ = 7.8 Hz, 1H, H-1), 4.34 (dd, *J* = 10.7, 4.9 Hz, 1H, H-6a), 3.92 (td, *J* = 8.9, 6.6 Hz, 1H, H-1a $_{linker}$), 3.84–3.76 (m, 2H, H-3, H-6b), 3.60–3.42 (m, 4H, H-1b$_{linker}$, H-4, H-2, H-5), 3.29 (t, *J* = 7.2 Hz, 2H, H-5$_{linker}$), 2.79 (s, 1H, O*H*), 2.65 (s, 1H, O*H*), 1.71–1.60 (m, 4H, H-2$_{linker}$, H-4$_{linker}$), 1.51–1.43 (m, 2H, H-3$_{linker}$); ^{13}C NMR (100 MHz, $CDCl_3$) δ 137.1 (C-Ar), 129.4, 128.5, 126.4 (3 × *C*H-Ar), 103.3 (C-1), 102.0 (C-7), 80.7 (C-4), 74.7 (C-2), 73.3 (C-3), 70.1 (C-1$_{linker}$), 68.8 (C-6), 66.5 (C-5), 51.4 (C-5$_{linker}$), 29.2 (C-2$_{linker}$), 28.6 (C-4$_{linker}$), 23.3 (C-3$_{linker}$); HRMS (ESI-TOF) *m/z* $[M + H]^+$ calcd for $C_{18}H_{26}N_3O_6$ 380.1816; found 380.1820; *m/z* $[M + NH_4]^+$ calcd for $C_{18}H_{29}N_4O_6$ 397.2082; found 397.2078; *m/z* $[M + Na]^+$ calcd for $C_{18}H_{25}N_3NaO_6$ 402.1635; found 402.1638.

(5-Azido-1-pentyl) 2-*O*-Benzyl-4,6-*O*-benzylidene-*β*-D-glucopyranoside (13).

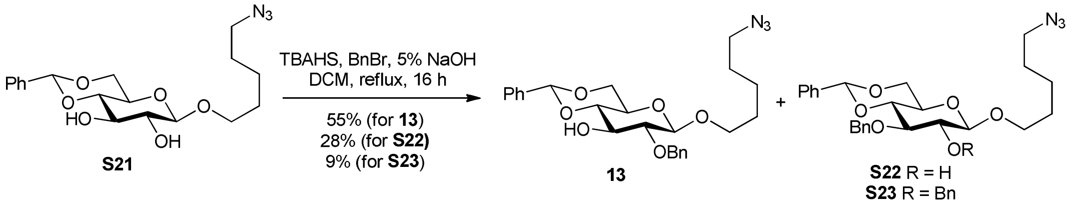

Diol **S21** (475 mg, 1.2 mmol, 1.0 equiv) was dissolved in DCM (14 mL), then a 5% NaOH(aq) solution (4 mL) was added followed by Bu_4NHSO_4 (85 mg, 250 μmol, 0.2 equiv) and benzyl bromide (261 μL, 2.2 mmol, 1.8 equiv). The emulsion was refluxed for 16 h under Ar. The reaction mixture was poured into a separatory funnel and the aqueous phase was extracted with DCM (3 × 30 mL). The organic layer was dried ($MgSO_4$) and the solvents were concentrated under reduced pressure. Purification by silica gel flash chromatography (PE/EtOAc 9:1 to 7:3) gave alcohol **13** (320 mg, 55%) as a white amorphous powder along with its regioisomer **S22** (160 mg, 28%) as a white foam, and fully benzylated **S23** (62 mg, 9%) as a colorless oil. Analytical data for **13**: R_f 0.4 (PE/EtOAc 7:3); $[\alpha]_D^{20}$ = –14 (*c* 1.2, $CHCl_3$); ^{1}H NMR (400 MHz, $CDCl_3$) δ 7.49–7.47 (m, 2H, C*H*-Ar), 7.39–7.28 (m, 8H, C*H*-Ar), 5.52 (s, 1H, H-7), 4.94 (d, *J* = 11.5 Hz, 1H, C*H*HPh), 4.74 (d, *J* = 11.5 Hz, 1H, CH*H*Ph), 4.51 (d, $J_{1.2}$ = 7.8 Hz, 1H, H-1), 4.34 (dd, *J* = 10.6, 4.9 Hz, 1H, H-6a), 3.94 (td, *J* = 8.9, 6.5 Hz, 1H, H-$1a_{linker}$), 3.83 (t, *J* = 9.5 Hz, 1H, H-3), 3.77 (t, *J* = 10.7 Hz, 1H, H-6b), 3.57 (dt, *J* = 9.6, 7.1 Hz, 1H, H-$1b_{linker}$), 3.54 (t, *J* = 9.6 Hz, 1H, H-4), 3.45–3.39 (m, 1H, H-5), 3.34 (dd, *J* = 9.3, 7.8 Hz, 1H, H-2), 3.24 (t, *J* = 7.3 Hz, 2H, H-5_{linker}), 2.49 (s, 1H, O*H*), 1.72–1.58 (m, 4H, H-2_{linker}, H-4_{linker}), 1.54–1.43 (m, 2H, H-3_{linker}); ^{13}C NMR (100 MHz, $CDCl_3$) δ 138.3, 137.1 (2 × C-Ar), 129.3–126.4 (6 × *C*H-Ar), 103.9 (C-1), 101.9 (C-7), 81.9 (C-2), 80.5 (C-4), 74.9 (*C*H_2Ph), 73.3 (C-3), 70.1 (C-1_{linker}), 68.8 (C-6), 66.2 (C-5), 51.4 (C-5_{linker}), 29.4 (C-2_{linker}), 28.7 (C-4_{linker}), 23.5 (C-3_{linker}); HRMS (ESI-TOF) *m/z* $[M + H]^+$ calcd for $C_{25}H_{32}N_3O_6$ 470.2285; found 470.2286; *m/z* $[M + NH_4]^+$ calcd for $C_{25}H_{35}N_4O_6$ 487.2551; found 487.2551; *m/z* $[M + Na]^+$ calcd for $C_{25}H_{31}N_3NaO_6$ 492.2105; found 492.2102. Analytical data for **S22**: $[\alpha]_D^{20}$ = –26 (*c* 1.5, $CHCl_3$); ^{1}H NMR (400 MHz, $CDCl_3$) δ 7.50–7.48 (m, 2H, C*H*-Ar), 7.41–7.28 (m, 8H, C*H*-Ar), 5.57 (s, 1H, H-7), 4.96 (d, *J* = 11.7 Hz, 1H, C*H*HPh), 4.80 (d, *J* = 11.7 Hz, 1H, CH*H*Ph), 4.39 (d, $J_{1,2}$ = 7.6 Hz, 1H, H-1), 4.34 (dd, *J* = 10.6, 4.9 Hz, 1H, H-6a), 3.90 (dt, *J* = 9.6, 6.7 Hz, 1H, H-$1a_{linker}$), 3.80 (t, *J* = 10.6 Hz, 1H, H-6b), 3.71 (t, *J* = 9.4 Hz, 1H, H-4), 3.66 (t, *J* = 9.2 Hz, 1H, H-3), 3.57 (dt, *J* = 9.2, 7.4 Hz, 1H, H-$1b_{linker}$), 3.47–3.41 (m, 1H, H-5), 3.28 (t, *J* = 7.1 Hz, 2H, H-5_{linker}), 2.46 (s, 1H, O*H*), 1.70–1.59 (m, 4H, H-2_{linker}, H-4_{linker}), 1.50–1.43 (m, 2H, H-3_{linker}); ^{13}C NMR (100 MHz, $CDCl_3$) δ 138.4, 137.3 (2 × C-Ar), 129.1–126.1 (6 × *C*H-Ar), 103.4 (C-1), 101.4 (C-7), 81.5 (C-4), 80.3 (C-3), 74.7 (*C*H_2Ph), 74.4 (C-2), 70.1 (C-1_{linker}), 68.8 (C-6), 66.5 (C-5), 51.4 (C-5_{linker}), 29.2 (C-2_{linker}), 28.7 (C-4_{linker}), 23.3 (C-3_{linker}); HRMS (ESI-TOF) *m/z* $[M + H]^+$ calcd for $C_{25}H_{32}N_3O_6$ 470.2285; found 470.2286; *m/z* $[M + NH_4]^+$ calcd for $C_{25}H_{35}N_4O_6$ 487.2551; found 487.2551; *m/z* $[M + Na]^+$ calcd for $C_{25}H_{31}N_3NaO_6$ 492.2105; found 492.2102. Analytical data for **S23**: $[\alpha]_D^{20}$ = –27 (*c* 5.2, $CHCl_3$); ^{1}H NMR (400 MHz, $CDCl_3$) δ 7.50–7.25 (m, 15H, C*H*-Ar), 5.56 (s, 1H, H-7), 4.91 (d, *J* = 11.7 Hz, 1H, C*H*HPh), 4.88 (d, *J* = 11.7 Hz, 1H, CH*H*Ph), 4.80 (d, *J* = 5.9 Hz, 1H, C*H*HPh), 4.78 (d, *J* = 5.9 Hz, 1H, CH*H*Ph), 4.49 (d, $J_{1,2}$ = 7.7 Hz, 1H, H-1), 4.34 (dd, *J* = 10.4, 5.1 Hz, 1H, H-6a), 3.93 (dt, *J* = 9.7, 6.8 Hz, 1H, H-$1a_{linker}$), 3.78 (t, *J* = 10.8 Hz, 1H, H-6b), 3.75 (t, *J* = 9.4 Hz, 1H, H-3), 3.68 (t, *J* = 9.5 Hz, 1H, H-4), 3.56 (dt, *J* = 9.7, 7.1 Hz, 1H, H-$1b_{linker}$), 3.45 (t, *J* = 8.6 Hz, 1H, H-2), 3.43–3.37 (m, 1H, H-5), 3.21 (t, *J* = 7.3 Hz, 2H, H-5_{linker}), 1.70–1.56 (m, 4H, H-2_{linker}, H-4_{linker}), 1.50–1.41 (m, 2H, H-3_{linker}); ^{13}C NMR (100 MHz, $CDCl_3$) δ 138.6, 138.4, 137.3 (3 × C-

Ar), 129.0–126.1 (9 × *C*H-Ar), 104.1 (C-1), 101.2 (C-7), 82.2 (C-2), 81.6 (C-4), 80.9 (C-3), 75.4, 75.2 (2 × *C*H_2Ph), 70.2 (C-1_{linker}), 68.8 (C-6), 66.1 (C-5), 51.3 (C-5_{linker}), 29.4 (C-2_{linker}), 28.7 (C-4_{linker}), 23.4 (C-3_{linker}); HRMS (ESI-TOF) *m/z* $[M + H]^+$ calcd for $C_{32}H_{38}N_3O_6$ 560.2755; found 560.2751; *m/z* $[M + NH_4]^+$ calcd for $C_{32}H_{41}N_4O_6$ 577.3020; found 577.3019; *m/z* $[M + Na]^+$ calcd for $C_{32}H_{37}N_3NaO_6$ 582.2574; found 582.2565.

Ethyl 4,6-*O*-Benzylidene-3-*O*-*tert*-butyldimethylsilyl-2-*O*-levulinoyl-1-thio-*β*-D-glucopyranoside (14).

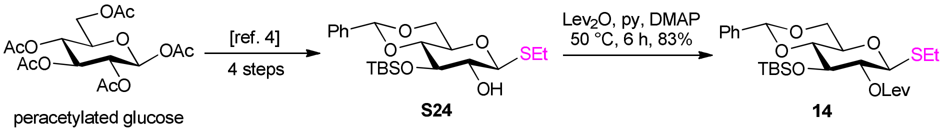

To a solution of alcohol **S24**[4] (1.8 g, 4.3 mmol, 1.0 equiv) in anhydrous py (28 mL) was added DMAP (1.3 g, 10.8 mmol, 2.5 equiv). A solution of levulinic anhydride[5] (8.3 g, 38.8 mmol, 9.0 equiv) in anhydrous py (38 mL) was added dropwise over 30 min to the former mixture. The reaction mixture was then heated to 50 °C and stirred under Ar for 6 h. The solvents were concentrated under reduced pressure and the residue was purified by silica gel flash chromatography (PE/EtOAc 95:5 to 85:15) to give **14** (1.9 g, 83%) as a yellow oil: R_f 0.3 (tol/EtOAc 95:5); $[\alpha]_D^{20}$ = –48 (*c* 1.2, $CHCl_3$); ^{1}H NMR (400 MHz, $CDCl_3$) δ 7.48–7.45 (m, 2H, C*H*-Ar), 7.37–7.35 (m, 3H, C*H*-Ar), 5.51 (s, 1H, H-7), 2.76 (dd, *J* = 10.3, 8.6 Hz, 1H, H-2), 4.45 (d, $J_{1,2}$ = 10.1 Hz, 1H, H-1), 4.32 (dd, *J* = 10.5, 4.8 Hz, 1H, H-6a), 3.88 (t, *J* = 9.0 Hz, 1H, H-3), 3.75 (t, *J* = 10.5 Hz, 1H, H-6b), 3.54 (t, *J* = 9.4 Hz, 1H, H-4), 3.50–3.44 (m, 1H, H-5), 2.83–275 (m, 2H, CH_{2SEt}), 2.73–2.59 (m, 4H, 2 × CH_{2Lev}), 2.20 (s, 3H, CH_{3Lev}), 1.25 (t, *J* = 7.4 Hz, 3H, CH_{3SEt}), 0.80 (s, 9H, $C(CH_3)_3$), 0.02, –0.02 (2 × s, 6H, 2 × CH_3); ^{13}C NMR (100 MHz, $CDCl_3$) δ 206.3 (CO), 171.7 (CO), 137.1 (C-Ar), 129.2, 128.3, 126.3 (3 × *C*H-Ar), 101.9 (C-7), 84.3 (C-1), 81.5 (C-4), 74.0 (C-3), 73.2 (C-2), 70.8 (C-5), 68.7 (C-6), 38.1 (CH_{2SEt}), 30.1 (CH_{3Lev}), 28,4 (CH_{2Lev}), 25.7 ($C(CH_3)_3$), 24.1 (CH_{2Lev}), 18.1 ($C(CH_3)_3$), 14.9 (CH_{3SEt}), –4.06, –4.80 (2 × CH_3); HRMS (ESI-TOF *m/z* $[M + Na]^+$ calcd for $C_{26}H_{40}NaO_7SSi$ 547.2156; found 547.2162; *m/z* $[2M + Na]^+$ calcd for $C_{52}H_{80}NaO_{14}S_2Si_2$ 1071.4420; found 1071.4425.

4,6-*O*-Benzylidene-3-*O*-*tert*-butyldimethylsilyl-2-*O*-levulinoyl-*α,β*-D-glucopyranose (S25).

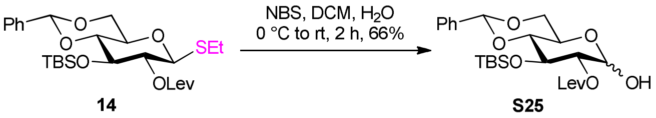

To a cooled (0 °C) solution of thioglucoside **14** (1.0 g, 1.9 mmol, 1.0 equiv) dissolved in DCM/water (22 mL, 10:1 *v/v*) was added NBS (498 mg, 2.7 mmol, 1.4 equiv). The reaction mixture was stirred from 0 °C to rt for 2 h. The mixture was then diluted with DCM (20 mL) and washed with a saturated $NaHCO_3$(aq) solution (10 mL). The aqueous phase was extracted with DCM (3 × 10 mL). The combined organic layers were washed with brine (20 mL). Solvents of the dried solution ($MgSO_4$) were concentrated under reduced pressure and the residue was purified by silica gel flash chromatography (PE/EtOAc 9:1 to 7:3) to give hemiacetal **S25** (630 mg, 66%, ratio *α*/*β* ~ 5:1) as a yellow oil: R_f 0.3 (tol/EtOAc 8:2); ^{1}H NMR (400 MHz, $CDCl_3$) *δ* 7.48–7.45 (m, 2H, C*H*-Ar), 7.36–7.33 (m, 3H, C*H*-Ar), 5.51 (s, 1H, H-7), 5.38 (d, *J* = 3.2 Hz, 1H, H-2), 4.74 (dd, *J* = 9.5, 3.6 Hz, 1H, H-3), 4.26 (dd, *J* = 10.3, 4.9 Hz, 1H, H-6a), 4.19 (t, *J* = 9.7 Hz, 1H, H-4), 4.07 (td, *J* = 10.4, 4.9 Hz, 1H, H-5), 3.71 (t, *J* = 10.8 Hz, 1H, H-6b), 3.47 (d, $J_{1,2}$ = 9.0 Hz, 1H, H-1), 2.89–272 (m, 2H, CH_{2Lev}), 2.62–2.48 (m, 2H, CH_{2Lev}), 2.19 (s, 3H, CH_{3Lev}), 0.81 (s, 9H, $C(CH_3)_3$), 0.05, 0.00 (2 × s, 6H, 2 × CH_3); ^{13}C NMR (100 MHz, $CDCl_3$) *δ* 207.4 (*C*O), 172.4 (*C*O), 137.4 (C-Ar), 129.1, 128.3, 126.3 (3 × *C*H-Ar), 101.9 (C-7), 90.9 (C-2), 82.1 (C-1), 75.3 (C-3), 69.3 (C-4), 69.1 (C-6), 62.6 (C-5), 38.3 (CH_{2Lev}), 29.9 (CH_{3Lev}), 28.2 (CH_{2Lev}), 25.8 ($C(CH_3)_3$), 18.3 ($C(CH_3)_3$), –4.14, –4.69 (2 × CH_3); HRMS (ESI-TOF) *m/z* $[M + Na]^+$ calcd for $C_{24}H_{36}NaO_8Si$ 503.2072; found 503.2082.

4,6-*O*-Benzylidene-3-*O*-*tert*-butyldimethylsilyl-2-*O*-levulinoyl-*α*-D-glucopyranosyl Fluoride (S26).

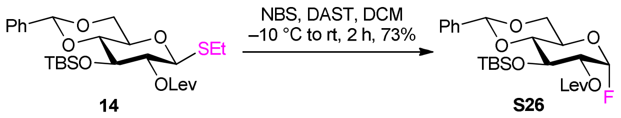

To a cooled (–10 °C) solution of thioglucoside **14** (100 mg, 191 *μ*mol, 1.0 equiv) in anhydrous DCM (2 mL) was added DAST (76 *μ*L, 572 *μ*mol, 3.0 equiv). The reaction mixture was stirred for 8 min, then NBS (47 mg, 267 *μ*mol, 1.4 equiv) was added. The mixture was stirred for 2 h from –10 °C to rt under Ar. The solution was diluted with DCM (20 mL). The organic phase was washed with a saturated $NaHCO_3$(aq) solution (2 × 10 mL) and brine (10 mL). Solvents of the dried solution ($MgSO_4$) were concentrated under reduced pressure and the residue was purified by silica gel flash chromatography (PE/EtOAc 85:15) to give fluoride **S26** (68 mg, 73%) as a yellow oil: R_f 0.2 (tol/EtOAc 8:2); $[\alpha]_D^{20}$ = –41 (*c* 0.63, $CHCl_3$); ^{1}H NMR (400 MHz, $CDCl_3$) δ 7.48–7.44 (m, 2H, C*H*-Ar), 7.39–7.34 (m, 3H, C*H*-Ar), 5.53 (s, 1H, H-7), 5.34 (dd, $J_{1,F}$ = 53.1, $J_{1,2}$ = 6.5 Hz, 1H, H-1), 5.08–5.01 (m, 1H, H-2), 4.38 (dd, *J* = 10.5, 4.8 Hz, 1H, H-6a), 3.90 (t, *J* = 8.6 Hz, 1H, H-3), 3.81 (t, *J* = 10.5 Hz, 1H, H-6b), 3.70 (t, *J* = 9.7 Hz, 1H, H-4), 3.56 (td, *J* = 10.1, 4.9 Hz, 1H, H-5), 2.83–273 (m, 2H, CH_{2Lev}), 2.72–2.58 (m, 2H, CH_{2Lev}), 2.19 (s, 3H, CH_{3Lev}), 0.81 (s, 9H, $C(CH_3)_3$), 0.04, 0.00 (2 × s, 6H, 2 × CH_3); ^{13}C NMR (100 MHz, $CDCl_3$) δ 206.2 (*C*O), 171.4 (*C*O), 136.9 (C-Ar), 129.3, 128.3, 126.3 (3 × *C*H-Ar), 108.3–106.1 (C-1), 101.9 (C-7), 80.8 (C-4), 74.9–74.7 (C-2), 72.3–72.2 (C-3), 68.6 (C-6), 66.1–66.0 (C-5), 37.9 (CH_{2Lev}), 29.9 (CH_{3Lev}), 28.0 (CH_{2Lev}), 25.7 ($C(CH_3)_3$), 18.1 ($C(CH_3)_3$), –4.20, –4.86 (2 × CH_3); HRMS (ESI-TOF) *m/z* [M + Na]$^+$ calcd for $C_{24}H_{35}FNaO_7Si$ 505.2028; found 505.2046.

4,6-*O*-Benzylidene-3-*O*-*tert*-butyldimethylsilyl-2-*O*-levulinoyl-*α,β*-D-glucopyranosyl *N*-Phenyl-2,2,2-trifluoroacetimidate (S27).

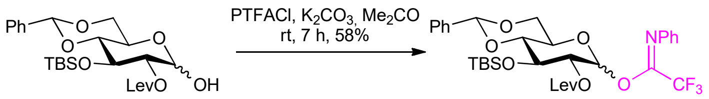

To a solution of hemiacetal **S25** (630 mg, 1.3 mmol, 1.0 equiv) in acetone (26 mL) were added K_2CO_3 (272 mg, 1.9 mmol, 1.5 equiv) followed by 2,2,2-trifluoro-*N*-phenylacetimidoyl chloride (PTFACl, 419 μL, 2.6 mmol, 2.0 equiv). The mixture was stirred for 7 h at rt under Ar, then the suspension was filtered over Celite and rinsed with DCM. The solvents were concentrated under reduced pressure. The residue was purified by silica gel flash chromatography (PE/EtOAc 9:1 + 1% Et_3N) to give imidate **S27** (499 mg, 58%, ratio *α*/*β* ~ 1:1) as a yellow amorphous solid: R_f 0.6 (PE/EtOAc 7:3); ^{1}H NMR (400 MHz, py-d_5) δ 7.50–7.34 (m, 6H, C*H*-Ar), 7.18–7.13 (m, 4H, C*H*-Ar), 5.78 (s, 1H, H-7), 5.60 (t, *J* = 9.2 Hz, 1H, H-2), 5.32 (d, *J* = 7.5 Hz, 1H, H-3), 4.58 (t, *J* = 9.9 Hz, 1H, H-4), 4.45–4.41 (m, 1H, H-6a), 4.32 (t, *J* = 9.5 Hz, 1H, H-5), 3.91–3.85 (m, 2H, H-1, H-6b), 2.97–2.75 (m, 4H, 2 × CH_{2Lev}), 2.10 (s, 3H, CH_{3Lev}), 0.96 (s, 9H, $C(CH_3)_3$), 0.21, 0.18 (2 × s, 6H, 2 × CH_3); ^{13}C NMR (100 MHz, py-d_5) δ 207.4 (*C*O), 172.4 (*C*O), 138.0, 137.4 (2 × C-Ar), 129.1–121.9 (6 × *C*H-Ar), 102.1 (C-7), 81.3 (C-1), 74.1 (C-2), 73.5 (C-3), 70.1 (C-4), 68.6 (C-6), 65.8 (C-5), 38.1 (CH_{2Lev}), 29.4 (CH_{3Lev}), 28.6 (CH_{2Lev}), 25.9 ($C(CH_3)_3$), 18.4 ($C(CH_3)_3$), –4.09, –4.69 (2 × CH_3).

(5-Azido-1-pentyl) 2-*O*-Acetyl-4-*O*-benzyl-6-deoxy-3-*O*-*para*-methoxybenzyl-*α*-L-talopyranosyl-(1→3)-2-*O*-benzyl-4,6-*O*-benzylidene-*β*-D-glucopyranoside (15).

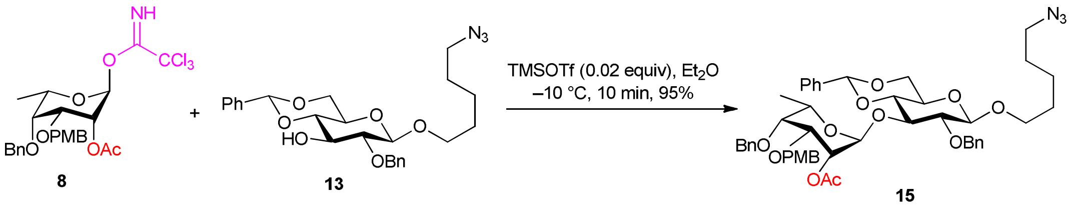

According to the general procedure for the synthesis of protected disaccharides, acceptor **13** (400 mg, 850 *µ*mol, 1.0 equiv) and donor **8** (956 mg, 1.7 mmol, 2.0 equiv) were reacted in the presence of TMSOTf (6 *µ*L, 34 *µ*mol, 0.02 equiv). Purification by combi-flash chromatography (tol/Et_2O 98:2 to 94:6) gave disaccharide **15** (698 mg, 95%) as a white amorphous solid. R_f 0.8 (tol/Et_2O 7:3); $[\alpha]_D^{20}$ = –20 (*c* 1.2, $CHCl_3$); ^{1}H NMR (400 MHz, $CDCl_3$) δ 7.41–7.20 (m, 17H, C*H*-Ar), 6.86–6.87 (m, 2H, C*H*-Ar), 5.46 (s, 1H, H-7), 5.44 (d, *J* = 3.7 Hz, 1H, H-2B), 5.34 (s, 1H, H-1B), 4.92 (d, *J* = 10.9 Hz, 1H, C*H*HPh), 4.81 (d, *J* = 11.8 Hz, 1H, CH*H*Ph), 4.69 (d, *J* = 11.2 Hz, 1H, C*H*HPh), 4.66 (d, *J* = 10.9 Hz, 1H, CH*H*Ph), 4.59 (d, *J* = 11.8 Hz, 1H, C*H*HPh), 4.50 (d, $J_{1A,2A}$ = 7.6 Hz, 1H, H-1A), 4.38 (d, *J* = 11.2 Hz, 1H, CH*H*Ph), 4.32 (dd, *J* = 10.8, 4.8 Hz, 1H, H-6aA), 4.10 (dd, *J* = 13.6, 6.3 Hz, 1H, H-5B), 3.98 (t, *J* = 9.4 Hz, 1H, H-3A), 3.92 (dt, *J* = 9.6, 6.4 Hz, 1H, H-$1a_{linker}$), 3.79 (s, 3H, CH_{3PMB}), 3.75 (t, *J* = 10.5 Hz, 1H, H-6bA), 3.70 (t, *J* = 3.7 Hz, 1H, H-3B), 3.56 (dt, *J* = 9.6, 6.9 Hz, 1H, H-$1b_{linker}$), 3.50 (t, *J* = 9.6 Hz, 1H, H-4A), 3.45 (t, *J* = 9.0 Hz, 1H, H-2A), 3.45–3.39 (m, 1H, H-5A), 3.36 (t, *J* = 1.4 Hz, 1H, H-4B), 3.19 (t, *J* = 7.3 Hz, 2H, H-5_{linker}), 1.96 (s, 3H, CH_{3Ac}), 1.69–1.55 (m, 4H, H-2_{linker}, H-4_{linker}), 1.49–1.42 (m, 2H, H-3_{linker}), 0.91 (d, *J* = 6.5 Hz, 3H, CH_{3Tal}); ^{13}C NMR (100 MHz, $CDCl_3$) δ 170.7 (*C*O), 159.1 (C-Ar), 139.2, 138.1, 137.3, 130.6 (4 × C-Ar), 129.4–126.3 (10 × *C*H-Ar), 113.8 (*C*H-Ar), 104.1 (C-1A), 101.8 (C-7), 99.2 (C-1B), 83.3 (C-2A), 79.3 (C-4A), 75.9 (C-4B), 75.5 (C-3A), 75.4 (C-3B), 74.9, 74.0, 70.7 (3 × CH_2Ph), 70.2 (C-1_{linker}), 68.9 (C-6), 67.1 (C-5B), 66.8 (C-2B), 66.4 (C-5A), 55.4 (CH_{3PMB}), 51.3 (C-5_{linker}), 29.4 (C-2_{linker}), 28.7 (C-4_{linker}), 23.4 (C-3_{linker}), 21.3 (CH_{3Ac}), 16.6 (CH_{3Tal}); HRMS (ESI-TOF) *m/z* $[M + Na]^+$ calcd for $C_{48}H_{57}N_3NaO_{12}$ 890.3834; found 890.3849.

(5-Azido-1-pentyl) 4-*O*-Acetyl-6-deoxy-3-*O*-*para*-methoxybenzyl-2-*O*-methyl-*α*-L-talopyranosyl-(1→3)-2-*O*-benzyl-4,6-*O*-benzylidene-*β*-D-glucopyranoside (16).

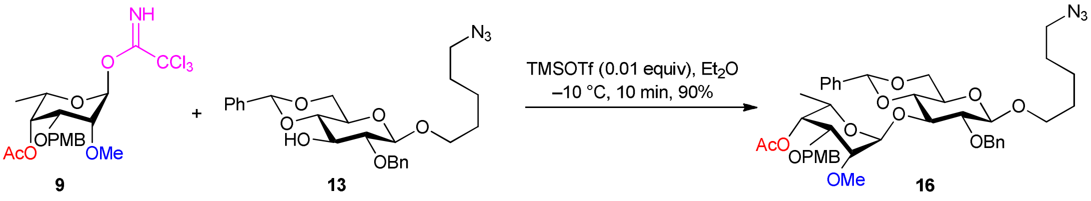

According to the general procedure for the synthesis of protected disaccharides, acceptor **13** (405 mg, 862 μmol, 1.0 equiv) and donor **9** (956 mg, 1.7 mmol, 2.0 equiv) were reacted in the presence of TMSOTf (2 μL, 9 μmol, 0.01 equiv). Purification by combi-flash chromatography (PE/EtOAc 73:27) gave disaccharide **16** (615 mg, 90%) as a white amorphous solid. R_f 0.6 (tol/EtOAc 8:2); $[\alpha]_D^{20}$ = –72 (*c* 1.4, $CHCl_3$); ^1H NMR (400 MHz, $CDCl_3$) δ 7.43–7.22 (m, 12H, C*H*-Ar), 6.87–6.85 (m, 2H, C*H*-Ar), 5.46 (s, 1H, H-7), 5.33 (d , *J* = 1.1 Hz, 1H, H-1B), 5.12 (t, *J* = 2.0 Hz, 1H, H-4B), 4.98 (d, *J* = 11.6 Hz, 1H, C*H*HPh), 4.63 (d, *J* = 11.5 Hz, 2H, CH_{2PMB}), 4.51 (d, $J_{1A,2A}$ = 7.8 Hz, 1H, H-1A), 4.39 (d, *J* = 11.6 Hz, 1H, CH*H*Ph), 4.59 (d, *J* = 11.8 Hz, 1H, C*H*HPh), 4.33 (dd, *J* = 10.6, 4.7 Hz, 1H, H-6aA), 4.16 (ddd, *J* = 13.7, 6.5, 1.4 Hz, 1H, H-5B), 3.95–3.90 (m, 2H, H-3A, H-$1a_{linker}$), 3.79 (s, 3H, CH_{3PMB}), 3.75 (t, *J* = 10.6 Hz, 1H, H-6bA), 3.67 (t, *J* = 3.7 Hz, 1H, H-3B), 3.56 (dt, *J* = 9.5, 7.0 Hz, 1H, H-$1b_{linker}$), 3.49 (t, *J* = 9.4 Hz, 1H, H-4A), 3.46–3.38 (m, 2H, H-2A, H-5A), 3.29 (dt, *J* = 3.6, 1.5 Hz, 1H, H-2B), 3.22 (s, 3H, CH_{3Me}), 3.19 (t, *J* = 7.3 Hz, 2H, H-5_{linker}), 2.11 (s, 3H, CH_{3Ac}), 1.69–1.55 (m, 4H, H-2_{linker}, H-4_{linker}), 1.50–1.39 (m, 2H, H-3_{linker}), 0.79 (d, *J* = 6.4 Hz, 3H, CH_{3Tal}); ^{13}C NMR (100 MHz, $CDCl_3$) δ 171.5 (*C*O), 159.2 (C-Ar), 138.2, 137.2, 130.5 (3 × C-Ar), 129.4–126.3 (7 × *C*H-Ar), 113.8 (*C*H-Ar), 104.2 (C-1A), 102.1 (C-7), 99.6 (C-1B), 83.3 (C-2A), 79.4 (C-4A), 77.1 (C-2B), 76.8 (C-3A), 75.0 (CH_2Ph), 73.4 (C-3B), 70.4 (CH_2Ph), 70.2 (C-1_{linker}), 69.2 (C-4B), 68.9 (C-6), 66.5 (C-5A), 65.0 (C-5B), 59.8 (CH_{3Me}), 55.4 (CH_{3PMB}), 51.4 (C-5_{linker}), 29.4 (C-2_{linker}), 28.7 (C-4_{linker}), 23.5 (C-3_{linker}), 21.2 (CH_{3Ac}), 15.8 (CH_{3Tal}); HRMS (ESI-TOF) *m/z* $[M + Na]^+$ calcd for $C_{42}H_{53}N_3NaO_{12}$ 814.3521; found 814.3515.

(5-Azido-1-pentyl) 2,4-Di-*O*-acetyl-6-deoxy-3-*O*-*para*-methoxybenzyl-*α*-L-talopyranosyl-(1→3)-2-*O*-benzyl-4,6-*O*-benzylidene-*β*-D-glucopyranoside (17).

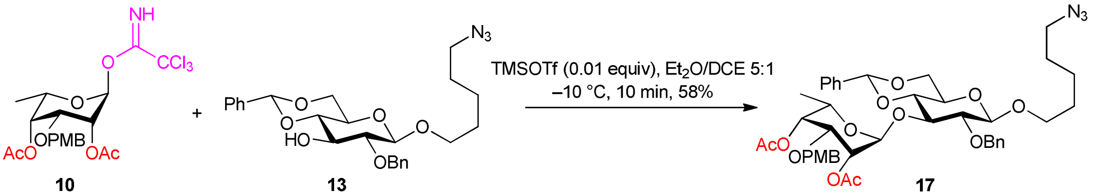

According to the general procedure for the synthesis of protected disaccharides, acceptor **13** (221 mg, 470 μmol, 1.0 equiv) and donor **10** (482 mg, 940 μmol, 2.0 equiv) were reacted in the presence of TMSOTf (1 μL, 5 μmol, 0.01 equiv). Purification by combi-flash chromatography (PE/EtOAc 75:25) gave disaccharide **17** (227 mg, 58%) as a white amorphous solid. R_f 0.5 (tol/EtOAc 8:2); $[\alpha]_D^{20}$ = –53 (*c* 3.2, $CHCl_3$); ^{1}H NMR (400 MHz, $CDCl_3$) δ 7.42–7.21 (m, 12H, C*H*-Ar), 6.86–6.84 (m, 2H, C*H*-Ar), 5.47 (s, 1H, H-7), 5.32 (d, *J* = 4.0 Hz, 1H, H-2B), 5.29 (s, 1H, H-1B), 5.05 (d, *J* = 3.2 Hz, 1H, H-4B), 4.91 (d, *J* = 10.7 Hz, 1H, C*H*HPh), 4.66 (d, *J* = 10.7 Hz, 1H, CH*H*Ph), 4.50 (d, $J_{1A,2A}$ = 8.0 Hz, 1H, H-1A), 4.49 (s, 2H, CH_{2PMB}), 4.33 (dd, *J* = 10.6, 4.8 Hz, 1H, H-6aA), 4.22 (dd, *J* = 14.1, 6.5 Hz, 1H, H-5B), 3.96–3.90 (m, 2H, H-3A, H-$1a_{linker}$), 3.79 (s, 3H, CH_{3PMB}), 3.74 (t, *J* = 10.0 Hz, 1H, H-6bA), 3.71 (t, *J* = 3.6 Hz, 1H, H-3B), 3.56 (dt, *J* = 9.5, 6.7 Hz, 1H, H-$1b_{linker}$), 3.50 (t, *J* = 9.7 Hz, 1H, H-4A), 3.46–3.39 (m, 2H, H-2A, H-5A), 3.21 (t, *J* = 7.3 Hz, 2H, H-5_{linker}), 2.07, 2.02 (2 × s, 6H, 2 × CH_{3Ac}), 1.70–1.56 (m, 4H, H-2_{linker}, H-4_{linker}), 1.49–1.41 (m, 2H, H-3_{linker}), 0.75 (d, *J* = 6.5 Hz, 3H, CH_{3Tal}); ^{13}C NMR (100 MHz, $CDCl_3$) δ 170.9, 170.1 (2 × *C*O), 159.1 (C-Ar), 138.0, 137.2, 130.3 (3 × C-Ar), 129.4–126.3 (7 × *C*H-Ar), 113.8 (*C*H-Ar), 104.2 (C-1A), 101.9 (C-7), 99.2 (C-1B), 83.1 (C-2A), 79.2 (C-4A), 76.2 (C-3A), 75.0 (*C*H_2Ph), 70.9 (C-3B), 70.3 (CH_{2PMB}), 70.2 (C-1_{linker}), 69.3 (C-4B), 68.9 (C-6), 66.7 (C-2B), 66.4 (C-5A), 64.9 (C-5B), 55.3 (CH_{3PMB}), 51.3 (C-5_{linker}), 29.4 (C-2_{linker}), 28.7 (C-4_{linker}), 23.5 (C-3_{linker}), 21.2, 21.0 (2 × CH_{3Ac}), 15.9 (CH_{3Tal}). HRMS (ESI-TOF) *m/z* [M + Na]$^+$ calcd for $C_{43}H_{53}N_3NaO_{13}$ 842.3471; found 842.3473; *m/z* [2M + Na]$^+$ calcd for $C_{86}H_{106}N_6NaO_{26}$ 1661.7049; found 10661.7047.

(5-Azido-1-pentyl) 2-*O*-Acetyl-4-*O*-benzyl-6-deoxy-3-*O*-methyl-*α*-L-talopyranosyl-(1→3)-2-*O*-benzyl-4,6-*O*-benzylidene-*β*-D-glucopyranoside (18).

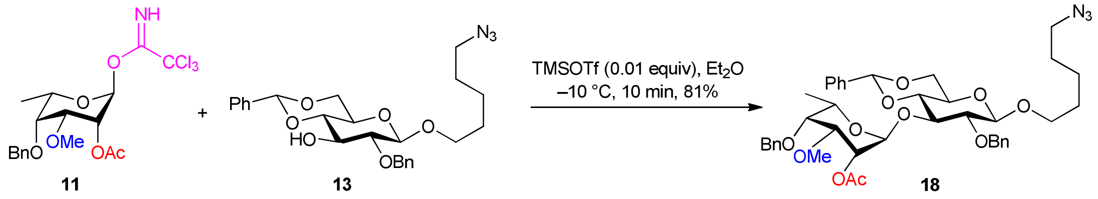

According to the general procedure for the synthesis of protected disaccharides, acceptor **13** (128 mg, 273 *μ*mol, 1.0 equiv) and donor **11** (248 mg, 546 *μ*mol, 2.0 equiv) were reacted in the presence of TMSOTf (0.5 *μ*L, 3 *μ*mol, 0.01 equiv). Purification by combi-flash chromatography (PE/EtOAc 85:15) gave disaccharide **47** (169 mg, 81%) as a white amorphous solid: R_f 0.6 (tol/EtOAc 85:15); $[\alpha]_D^{20}$ = –38 (*c* 0.13, $CHCl_3$); ^{1}H NMR (400 MHz, $CDCl_3$) *δ* 7.44–7.21 (m, 15H, C*H*-Ar), 5.48 (s, 1H, H-7), 5.37 (dt, *J* = 3.8, 1.4 Hz, 1H, H-2B), 5.33 (s, 1H, H-1B), 4.90 (d, *J* = 10.8 Hz, 1H, C*H*HPh), 4.81 (d, *J* = 11.8 Hz, 1H, CH*H*Ph), 4.66 (d, *J* = 10.8 Hz, 1H, CH*H*Ph), 4.56 (d, *J* = 11.8 Hz, 1H, CH*H*Ph), 4.50 (d, $J_{1A,2A}$ = 7.8 Hz, 1H, H-1A), 4.32 (dd, *J* = 10.6, 4.9 Hz, 1H, H-6aA), 4.12 (dd, *J* = 13.6, 6.5 Hz, 1H, H-5B), 3.99 (t, *J* = 9.5 Hz, 1H, H-3A), 3.92 (dt, *J* = 9.6, 6.5 Hz, 1H, H-$1a_{linker}$), 3.75 (t, *J* = 10.6 Hz, 1H, H-6bA), 3.57 (dt, *J* = 9.6, 6.8 Hz, 1H, H-$1b_{linker}$), 3.51 (t, *J* = 9.2 Hz, 1H, H-4A), 3.49 (t, *J* = 3.9 Hz, 1H, H-3B), 3.47–3.41 (m, 3H, H-2A, H-4A, H-5A), 3.39 (s, 3H, CH_{3Me}), 3.19 (t, *J* = 7.3 Hz, 2H, H-5_{linker}), 1.97 (s, 3H, CH_{3Ac}), 1.68–1.55 (m, 4H, H-2_{linker}, H-4_{linker}), 1.48–1.41 (m, 2H, H-3_{linker}), 0.92 (d, *J* = 6.5 Hz, 3H, CH_{3Tal}); ^{13}C NMR (100 MHz, $CDCl_3$) *δ* 170.7 (*C*O), 139.3, 138.1, 137.4 (3 × C-Ar), 129.3–126.3 (9 × *C*H-Ar), 104.2 (C-1A), 101.8 (C-7), 99.2 (C-1B), 83.3 (C-2A), 79.4 (C-4A), 77.7 (C-3B), 75.7 (C-4B), 75.4 (C-3A), 74.9 (*C*H_2Ph), 74.0 (*C*H_2Ph), 70.2 (C-1_{linker}), 68.9 (C-6), 67.1 (C-5B), 66.6 (C-2B), 66.4 (C-5A), 57.1 (*C*H_{3Me}), 51.3 (C-5_{linker}), 29.4 (C-2_{linker}), 28.7 (C-4_{linker}), 23.5 (C-3_{linker}), 21.3 (*C*H_{3Ac}), 16.7 (*C*H_{3Tal}); HRMS (ESI-TOF) *m/z* $[M + Na]^+$ calcd for $C_{41}H_{51}N_3NaO_{11}$ 784.3416; found 784.3435; *m/z* $[2M + Na]^+$ calcd for $C_{82}H_{102}N_6NaO_{22}$ 1545.6939; found 1545.6987.

(5-Azido-1-pentyl) 2,4-Di-*O*-acetyl-6-deoxy-3-*O*-methyl-*α*-L-talopyranosyl-(1→3)-2-*O*-benzyl-4,6-*O*-benzylidene-*β*-D-glucopyranoside (19).

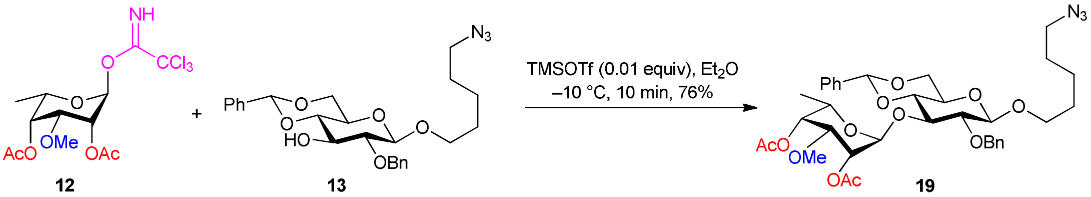

According to the general procedure for the synthesis of protected disaccharides, acceptor **13** (153 mg, 326 μmol, 1.0 equiv) and donor **12** (265 mg, 651 μmol, 2.0 equiv) were reacted in the presence of TMSOTf (0.6 μL, 3 μmol, 0.01 equiv). Purification by silica gel flash chromatography (tol/EtOAc 9:1 to 8:2) gave disaccharide **19** (176 mg, 76%) as a white amorphous solid: R_f 0.4 (tol/EtOAc 7:3); $[\alpha]_D^{20}$ = –62 (*c* 0.13, $CHCl_3$); ^{1}H NMR (400 MHz, $CDCl_3$) δ 7.44–7.26 (m, 10H, C*H*-Ar), 5.49 (s, 1H, H-7), 5.29 (s, 1H, H-1B), 5.28 (br s, 1H, H-2B), 5.04 (d, *J* = 3.0 Hz, 1H, H-4B), 4.90 (d, *J* = 10.7 Hz, 1H, C*H*HPh), 4.68 (d, *J* = 10.7 Hz, 1H, CH*H*Ph), 4.51 (d, $J_{1A,2A}$ = 7.8 Hz, 1H, H-1A), 4.34 (dd, *J* = 10.5, 4.9 Hz, 1H, H-6aA), 4.25 (ddd, *J* = 13.8, 6.5, 1.1 Hz, 1H, H-5B), 3.96–3.90 (m, 2H, H-3A , H-$1a_{linker}$), 3.77 (t, *J* = 10.6 Hz, 1H, H-6bA), 3.59–3.42 (m, 5H, H-2A, H-3B, H-4A, H-5A, H-$1b_{linker}$), 3.34 (s, 3H, CH_{3Me}), 3.21 (t, *J* = 7.3 Hz, 2H, H-5_{linker}), 2.09, 2.03 (2 × s, 6H, 2 × CH_{3Ac}), 1.69–1.56 (m, 4H, H-2_{linker}, H-4_{linker}), 1.49–1.40 (m, 2H, H-3_{linker}), 0.76 (d, *J* = 6.5 Hz, 3H, CH_{3Tal}); ^{13}C NMR (100 MHz, $CDCl_3$) δ 170.9, 170.1 (2 × *C*O), 138.0, 137.2 (2 × C-Ar), 129.5–126.3 (6 × *C*H-Ar), 104.2 (C-1A), 102.0 (C-7), 99.1 (C-1B), 83.1 (C-2A), 79.2 (C-4A), 76.1 (C-3A), 75.0 (*C*H_2Ph), 73.7 (C-3B), 70.2 (C-1_{linker}), 68.9 (C-6), 68.8 (C-4B), 66.5 (C-5A), 66.4 (C-2B), 65 (C-5B), 57.2 (*C*H_{3Me}), 51.3 (C-5_{linker}), 29.4 (C-2_{linker}), 28.7 (C-4_{linker}), 23.5 (C-3_{linker}), 21.2, 21.0 (2 × *C*H_{3Ac}), 15.6 (*C*H_{3Tal}); HRMS (ESI-TOF) *m/z* $[M + Na]^+$ calcd for $C_{36}H_{47}N_3NaO_{12}$ 736.3052; found 736.3061; *m/z* $[2M + Na]^+$ calcd for $C_{72}H_{94}N_6NaO_{24}$ 1449.6212; found 1449.6246.

(5-Azido-1-pentyl) 2-*O*-Benzyl-4,6-*O*-benzylidene-3-*O*-trimethylsilyl-*β*-D-glucopyranoside (21).

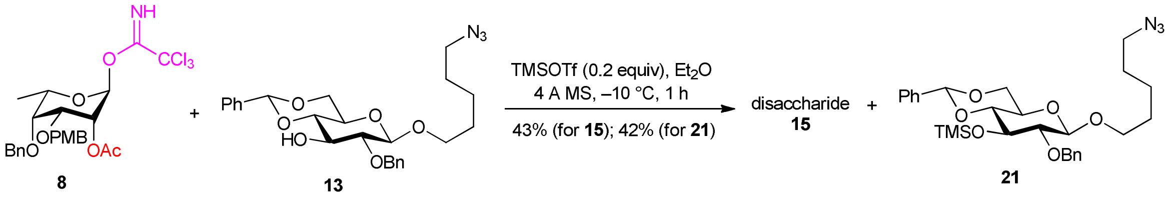

According to the general procedure for the synthesis of protected disaccharides, this derivative was obtained when the reaction was performed for only 1 h in anhydrous Et_2O with 4 Å molecular sieves. Purification by silica gel combi-flash chromatography (tol/Et_2O) gave silylated derivative **21** (42%) as a white amorphous powder along with disaccharide **15** (43%). Analytical data for **21**: $[\alpha]_D^{20}$ = –10 (*c* 1.7, $CHCl_3$); ^1H NMR (400 MHz, $CDCl_3$) δ 7.49–7.27 (m, 10H, C*H*-Ar), 5.51 (s, 1H, H-7), 4.86 (d, *J* = 11.1 Hz, 1H, C*H*HPh), 4.76 (d, *J* = 11.1 Hz, 1H, CH*H*Ph), 4.47 (d, $J_{1,2}$ = 9.8 Hz, 1H, H-1), 4.33 (dd, *J* = 10.4, 4.9 Hz, 1H, H-6aA), 3.92 (dt, *J* = 9.5, 6.6 Hz, 1H, H-$1a_{linker}$), 3.82 (t, *J* = 9.2 Hz, 1H, H-3), 3.77 (t, *J* = 10.4 Hz, 1H, H-6bA), 3.55 (dt, *J* = 9.6, 7.0 Hz, 1H, H-$1b_{linker}$), 3.48 (t, *J* = 9.6 Hz, 1H, H-4), 3.37 (dd, *J* = 9.7, 5.0 Hz, 1H, H-5), 3.32 (dd, *J* = 8.5, 8.0 Hz, 1H, H-2), 3.21 (t, *J* = 7.1 Hz, 2H, H-5_{linker}), 1.69–1.55 (m, 4H, H-2_{linker}, H-4_{linker}), 1.50–1.39 (m, 2H, H-3_{linker}), 0.09 (s, 9H, 3 × C*H*$_3$); ^{13}C NMR (100 MHz, $CDCl_3$) δ 138.6, 137.4 (2 × C-Ar), 129.1–126.3 (6 × *C*H-Ar), 104.1 (C-1), 101.6 (C-7), 83.1 (C-2), 81.4 (C-4), 75.4 (*C*H_2Ph), 74.5 (C-3), 70.2 (C-1_{linker}), 68.9 (C-6), 66.2 (C-5), 51.4 (C-5_{linker}), 29.4 (C-2_{linker}), 28.7 (C-4_{linker}), 23.4 (C-3_{linker}), 0.68 (($CH_3)_3$); HRMS (ESI-TOF) *m/z* $[M + Na]^+$ calcd for $C_{28}H_{39}N_3NaO_6Si$ 564.2500; found 564.2503.

(5-Azido-1-pentyl) 2-*O*-Acetyl-4-*O*-benzyl-6-deoxy-*α*-L-talopyranosyl-(1→3)-2-*O*-benzyl-4,6-*O*-benzylidene-*β*-D-glucopyranoside (20).

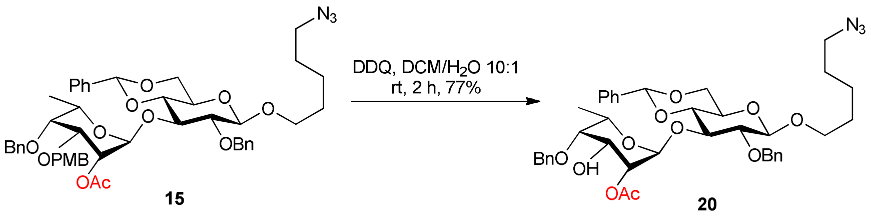

According to the general procedure for the deprotection of PMB group, disaccharide **15** (344 mg, 400 *μ*mol, 1.0 equiv) was reacted in the presence of DDQ (180 mg, 790 *μ*mol, 2.0 equiv). Purification by silica gel flash chromatography (PE/EtOAc 85:15 to 5:5) gave alcohol **20** (255 mg, 77%) as a white amorphous powder: R_f 0.4 (tol/EtOAc 8:2); $[\alpha]_D^{20}$ = –70 (*c* 1.3, $CHCl_3$); ^1H NMR (400 MHz, $CDCl_3$) *δ* 7.38–7.16 (m, 15H, C*H*-Ar), 5.42 (s, 1H, H-7), 5.24 (s, 1H, H-1B), 4.92 (d, *J* = 4.2 Hz, 1H, H-2B), 4.82 (d, *J* = 10.9 Hz, 1H, C*H*HPh), 4.64 (d, *J* = 11.6 Hz, 1H, CH*H*Ph), 4.63 (d, *J* = 10.9 Hz, 1H, C*H*HPh), 4.45 (d, *J* = 11.6 Hz, 1H, CH*H*Ph), 4.42 (d, $J_{1A,2A}$ = 7.8 Hz, 1H, H-1A), 4.26 (dd, *J* = 10.4, 5.0 Hz, 1H, H-6aA), 4.12 (dd, *J* = 14.2, 6.6 Hz, 1H, H-5B), 3.92–3.82 (m, 3H, H-3A, H-3B, H-$1a_{linker}$), 3.69 (t, *J* = 10.5 Hz, 1H, H-6bA), 3.51–3.33 (m, 4H, H-$1b_{linker}$, H-4A, H-2A, H-5A), 3.27 (d, *J* = 3.5 Hz, 1H, H-4B), 3.13 (t, *J* = 7.1 Hz, 2H, H-5_{linker}), 2.50 (d, *J* = 9.2 Hz, 1H, O*H*), 1.89 (s, 3H, CH_{3Ac}), 1.61–1.48 (m, 4H, H-2_{linker}, H-4_{linker}), 1.42–1.30 (m, 2H, H-3_{linker}), 0.87 (d, *J* = 6.6 Hz, 3H, CH_{3Tal}); ^{13}C NMR (100 MHz, $CDCl_3$) *δ* 171.4 (*C*O), 138.7, 138.3, 137.4 (3 × C-Ar), 129.3–126.3 (9 × *C*H-Ar), 104.2 (C-1A), 101.9 (C-7), 98.6 (C-1B), 83.2 (C-2A), 79.4 (C-4B), 79.3 (C-4A), 76.1 (CH_2Ph), 75.6 (C-3A), 74.9 (CH_2Ph), 70.4 (C-2B), 70.2 (C-1_{linker}), 68.9 (C-6), 66.5 (C-3B), 66.4 (C-5A), 66.3 (C-5B), 51.4 (C-5_{linker}), 29.4 (C-2_{linker}), 28.7 (C-4_{linker}), 23.5 (C-3_{linker}), 21.2 (CH_{3Ac}), 16.6 (CH_{3Tal}); HRMS (ESI-TOF) *m/z* $[M + Na]^+$ calcd for $C_{40}H_{49}N_3NaO_{11}$ 770.3259; found 770.3270.

(5-Azido-1-pentyl) 4-*O*-Acetyl-6-deoxy-2-*O*-methyl-*α*-L-talopyranosyl-(1→3)-2-*O*-benzyl-4,6-*O*-benzylidene-*β*-D-glucopyranoside (22).

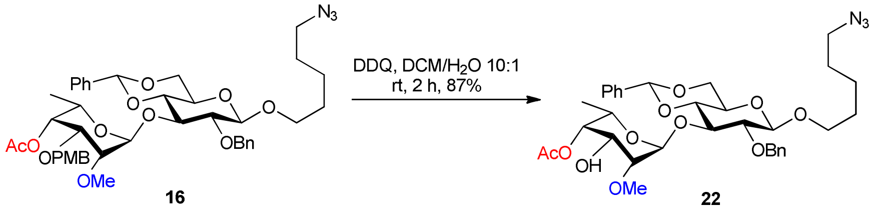

According to the general procedure for the deprotection of PMB group, disaccharide **16** (50 mg, 60 *μ*mol, 1.0 equiv) was reacted in the presence of DDQ (28 mg, 120 *μ*mol, 2.0 equiv). Purification by silica gel flash chromatography (PE/EtOAc 8:2 to 5:5) gave alcohol **22** (35 mg, 87%) as a white amorphous powder: R_f 0.3 (tol/EtOAc 8:2); ^{1}H NMR (400 MHz, $CDCl_3$) δ 7.43–7.23 (m, 10H, C*H*-Ar), 5.48 (s, 1H, H-7), 5.33 (s, 1H, H-1B), 5.06 (d, *J* = 11.6 Hz, 1H, C*H*HPh), 4.89 (d, *J* = 4.1 Hz, 1H, H-4B), 4.63 (d, *J* = 11.6 Hz, 1H, CH*H*Ph), 4.53 (d, $J_{1A,2A}$ = 7.8 Hz, 1H, H-1A), 4.35 (dd, *J* = 10.3, 4.8 Hz, 1H, H-6aA), 4.20 (dd, *J* = 14.0, 6.4 Hz, 1H, H-5B), 3.96 (t, *J* = 9.8 Hz, 1H, H-3A), 3.95–3.86 (m, 2H, H-1a_{linker}, H-3B), 3.76 (t, *J* = 10.7 Hz, 1H, H-6bA), 3.58–3.42 (m, 4H, H-1b_{linker}, H-2A, H-4A, H-5A), 3.20 (d, *J* = 4.3 Hz, 1H, H-2B), 3.16 (t, *J* = 7.2 Hz, 2H, H-5_{linker}), 3.01 (s, 3H, CH_{3Me}), 2.67 (d, *J* = 10.6 Hz, 1H, O*H*), 2.09 (s, 3H, CH_{3Ac}), 1.67–1.52 (m, 4H, H-2_{linker}, H-4_{linker}), 1.45–1.37 (m, 2H, H-3_{linker}), 0.64 (d, *J* = 6.6 Hz, 3H, CH_{3Tal}); ^{13}C NMR (100 MHz, $CDCl_3$) δ 171.5 (*C*O), 138.4, 137.1 (2 × C-Ar), 129.4–126.3 (6 × *C*H-Ar), 104.1 (C-1A), 102.1 (C-7), 97.9 (C-1B), 83.4 (C-2A), 79.4 (C-4A), 78.0 (C-2B), 76.7 (C-3A), 74.7 (*C*H_2Ph), 72.2 (C-4B), 70.1 (C-1_{linker}), 68.9 (C-6), 66.5 (C-5A), 65.1 (C-3B), 64.4 (C-5B), 59.4 (*C*H_{3Me}), 51.3 (C-5_{linker}), 29.3 (C-2_{linker}), 28.6 (C-4_{linker}), 23.4 (C-3_{linker}), 21.0 (*C*H_{3Ac}), 15.8 (*C*H_{3Tal}); HRMS (ESI-TOF) *m/z* $[M + Na]^+$ calcd for $C_{34}H_{45}N_3NaO_{11}$ 694.2946; found 694.2952; *m/z* $[2M + Na]^+$ calcd for $C_{68}H_{90}N_6NaO_{22}$ 1365.6000; found 1365.6012.

(5-Azido-1-pentyl) 2,4-Di-*O*-acetyl-6-deoxy-*α*-L-talopyranosyl-(1→3)-2-*O*-benzyl-4,6-*O*-benzylidene-*β*-D-glucopyranoside (23).

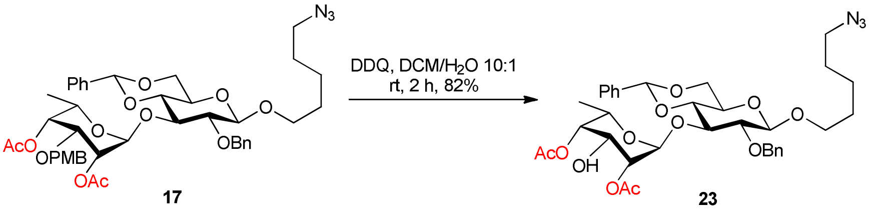

According to the general procedure for the deprotection of PMB group, disaccharide **17** (50 mg, 60 *μ*mol, 1.0 equiv) was reacted in the presence of DDQ (27 mg, 120 *μ*mol, 2.0 equiv). Purification by silica gel flash chromatography (PE/EtOAc 7:3 to 6:4) gave alcohol **23** (35 mg, 82%) as a white amorphous powder: R_f 0.2 (PE/EtOAc 7:3); ^{1}H NMR (400 MHz, $CDCl_3$) δ 7.42–7.27 (m, 10H, C*H*-Ar), 5.48 (s, 1H, H-7), 5.28 (s, 1H, H-1B), 5.04 (d, J = 3.5 Hz, 1H, H-2B), 4.90 (d, J = 10.9 Hz, 1H, C*H*HPh), 4.88 (s, 1H, H-4B), 4.68 (d, J = 10.9 Hz, 1H, CH*H*Ph), 4.50 (d, $J_{1A,2A}$ = 7.8 Hz, 1H, H-1A), 4.34 (dd, J = 10.6, 4.8 Hz, 1H, H-6aA), 4.28 (dd, J = 14.7, 6.6 Hz, 1H, H-5B), 4.11 (t, J = 4.3 Hz, 1H, H-3B), 3.95–3.90 (m, 2H, H-1a_{linker}, H-3A), 3.76 (t, J = 10.8 Hz, 1H, H-6bA), 3.58–3.40 (m, 4H, H-1b_{linker}, H-2A, H-4A, H-5A), 3.20 (t, J = 7.3 Hz, 2H, H-5_{linker}), 2.09, 2.04 (2 × s, 6H, 2 × CH_{3Ac}), 1.69–1.56 (m, 4H, H-2_{linker}, H-4_{linker}), 1.48–1.40 (m, 2H, H-3_{linker}), 0.75 (d, J = 6.6 Hz, 3H, CH_{3Tal}); ^{13}C NMR (100 MHz, $CDCl_3$) δ 171.7, 171.1 (2 × *C*O), 138.1, 137.1 (2 × C-Ar), 129.4–126.2 (6 × *C*H-Ar), 104.2 (C-1A), 101.9 (C-7), 98.7 (C-1B), 83.1 (C-2A), 79.2 (C-4A), 76.1 (C-3A), 74.9 (*C*H_2Ph), 71.8 (C-4B), 70.2 (C-1_{linker}), 69.6 (C-2B), 68.9 (C-6), 66.4 (C-5A), 65.7 (C-3B), 64.7 (C-5B), 51.3 (C-5_{linker}), 29.4 (C-2_{linker}), 28.7 (C-4_{linker}), 23.4 (C-3_{linker}), 21.1, 20.9 (2 × *C*H_{3Ac}), 15.9 (*C*H_{3Tal}); HRMS (ESI-TOF) *m/z* [M + Na]$^+$ calcd for $C_{35}H_{45}N_3NaO_{12}$ 722.2895; found 722.2886; *m/z* [2M + Na]$^+$ calcd for $C_{70}H_{90}N_6NaO_{24}$ 1421.5899; found 1421.5888.

(5-Azido-1-pentyl) 4,6-*O*-Benzylidene-3-*O*-*tert*-butyldimethylsilyl-2-*O*-levulinoyl-*β*-D-glucopyranosyl-(1→3)-4-*O*-acetyl-6-deoxy-2-*O*-methyl-*α*-L-talopyranosyl-(1→3)-2-*O*-benzyl-4,6-*O*-benzylidene-*β*-D-glucopyranoside (25).

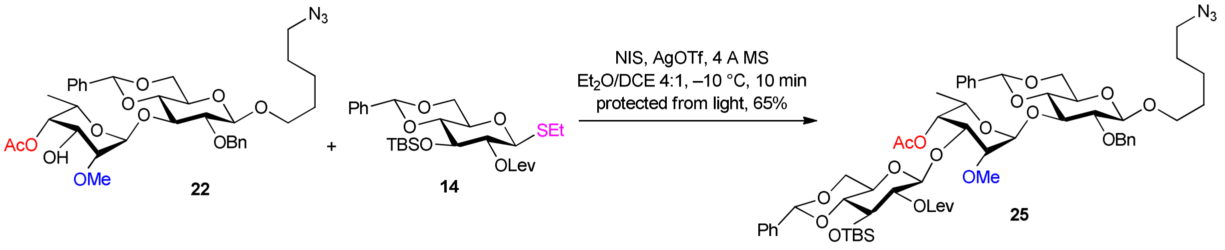

To a solution of donor **14** (457 mg, 871 *µ*mol, 1.5 equiv) and acceptor **22** **(**390 mg, 581 *µ*mol, 1.0 equiv) in anhydrous Et_2O/DCE (17 mL, 4:1 *v/v*) was added freshly activated 4 Å molecular sieves (1.5 g). The mixture was stirred at rt for 1 h under Ar. Then, the suspension was cooled to –10 °C, AgOTf (149 mg, 581 *µ*mol, 1.0 equiv) and NIS (261 mg, 1.2 mmol, 2.0 equiv) were added and the flask was protected from light. The reaction mixture was stirred for 10 min at –10 °C under Ar and then quenched with a few drops of Et_3N. The suspension was filtered over Celite, rinsed with DCM and the filtrate was concentrated under reduced pressure. The residue was purified by combi-flash chromatography (PE/EtOAc 71:29) to give trisaccharide **25** (432 mg, 65%) as a yellow amorphous solid: R_f 0.3 (tol/EtOAc 8:2); $[\alpha]_D^{20}$ = –57 (*c* 1.5, $CHCl_3$); ^{1}H NMR (400 MHz, $CDCl_3$) δ 7.48–7.28 (m, 15H, C*H*-Ar), 5.49 (s, 2H, H-7A, H-7C), 5.32 (s, 1H, H-1B), 4.98 (d, *J* = 11.4 Hz, 1H, C*H*HPh), 4.96 (s, 1H, H-4B), 4.90 (t, *J* = 9.0 Hz, 1H, H-2C), 4.62 (d, *J* = 11.4 Hz, 1H, CH*H*Ph), 4.58 (d, $J_{1C,2C}$ = 7.8 Hz, 1H, H-1C), 4.51 (d, $J_{1A,2A}$ = 7.8 Hz, 1H, H-1A), 4.31 (ddd, *J* = 19.6, 10.4, 4.9 Hz, 2H, H-6aA, H-6aC), 4.19 (dd, *J* = 15.8, 5.8 Hz, 1H, H-5B), 3.95–3.90 (m, 3H, H-$1a_{linker}$, H-3A, H-3B), 3.82 (t, *J* = 9.3 Hz, 1H, H-3C), 3.78–3.70 (m, 2H, H-6bA, H-6bC), 3.58–3.53 (m, 2H, H-$1b_{linker}$, H-4A), 3.50 (t, *J* = 8.9 Hz, 1H, H-4C), 3.46–3.40 (m, 3H, H-2A, H-5A, H-5C), 3.37 (br s, 1H, H-2B), 3.24 (s, 3H, CH_{3Me}), 3.20 (t, *J* = 7.3 Hz, 2H, H-5_{linker}), 2.77–2.74 (m, 2H, CH_{2Lev}), 2.66–2.62 (m, 2H, CH_{2Lev}), 2.16 (s, 3H, CH_{3Lev}), 2.09 (s, 3H, CH_{3Ac}), 1.69–1.56 (m, 4H, H-2_{linker}, H-4_{linker}), 1.51–1.40 (m, 2H, H-3_{linker}), 0.78 (s, 9H, C(C*H*$_3$)$_3$), 0.71 (d, *J* = 6.5 Hz, 3H, CH_{3Tal}), 0.00, –0.04 (2 × s, 6H, 2 × C*H*$_3$); ^{13}C NMR (100 MHz, $CDCl_3$) δ 206.6 (*C*O), 171.5, 171.4 (2 × *C*O), 138.1, 137.2, 137.1 (3 × C-Ar), 129.4–126.3 (9 × *C*H-Ar), 104.1 (C-1A), 102.1 (C-7A, C-7C), 100.9 (C-1C), 100.4 (C-1B), 83.2 (C-2A), 81.6 (C-4C), 79.4 (C-4A), 77.8 (C-2B), 77.0 (C-3A), 75.1 (*C*H$_2$Ph), 74.7 (C-2C), 74.4 (C-3B), 72.7 (C-3C), 70.2 (C-1_{linker}), 69.5 (C-4B), 68.9 (C-6A), 68.7 (C-6C), 66.5 (C-5A), 66.3 (C-5C), 65.0 (C-5B), 60.1 (CH_{3Me}), 51.4 (C-5_{linker}), 37.9 (CH_{2Lev}), 30.1 (CH_{3Lev}), 29.4 (C-2_{linker}), 28.7 (C-4_{linker}), 28.1 (CH_{2Lev}), 25.7 (C(*C*H$_3$)$_3$), 23.5 (C-3_{linker}), 21.1 (CH_{3Ac}), 18.1 (*C*(CH$_3$)$_3$), 15.7 (CH_{3Tal}), –4.07, –4.83 (2 × *C*H$_3$); HRMS (ESI-TOF) *m/z* [M + Na]$^+$ calcd for $C_{58}H_{79}N_3NaO_{18}Si$ 1156.5020; found 1156.5040.

(5-Azido-1-pentyl) 4,6-*O*-Benzylidene-3-*O*-*tert*-butyldimethylsilyl-2-*O*-levulinoyl-*β*-D-glucopyranosyl-(1→3)-2,4-di-*O*-acetyl-6-deoxy-*α*-L-talopyranosyl-(1→3)-2-*O*-benzyl-4,6-*O*-benzylidene-*β*-D-glucopyranoside (26).

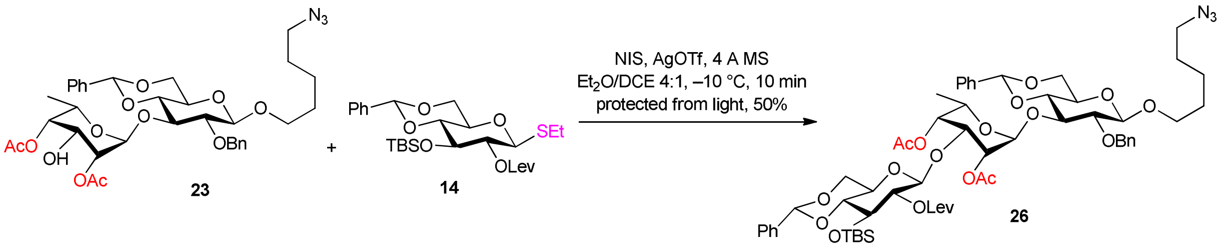

To a solution of donor **14** (105 mg, 200 *µ*mol, 1.5 equiv) and acceptor **23 (**93 mg, 133 *µ*mol, 1.0 equiv) in anhydrous Et_2O/DCE (4 mL, 4:1 *v/v*) was added freshly activated 4 Å molecular sieves (374 mg). The mixture was stirred at rt for 1 h under Ar. Then, the suspension was cooled to –10 °C, AgOTf (34 mg, 133 *µ*mol, 1.0 equiv) and NIS (60 mg, 267 *µ*mol, 2.0 equiv) were added and the flask was protected from light. The reaction mixture was stirred for 10 min at –10 °C under Ar and then quenched with a few drops of Et_3N. The suspension was filtered over Celite, rinsed with DCM and the filtrate was concentrated under reduced pressure. The residue was purified by combi-flash chromatography (PE/EtOAc 8:2) to give trisaccharide **26** (77 mg, 50%) as a colorless solid: R_f 0.5 (tol/EtOAc 8:2); $[\alpha]_D^{20}$ = –65 (*c* 1.5, $CHCl_3$); ^{1}H NMR (400 MHz, $CDCl_3$) *δ* 7.48–7.28 (m, 15H, C*H*-Ar), 5.53 (s, 1H, H-7A), 5.50 (s, 1H, H-7C), 5.31 (s, 1H, H-1B), 5.24 (d, *J* = 3.9 Hz, 1H, H-2B), 4.96 (d, *J* = 3.3 Hz, 1H, H-4B), 4.88 (d, *J* = 10.6 Hz, 1H, C*H*HPh), 4.85 (dd, *J* = 7.9, 7.7 Hz, 1H, H-2C), 4.74 (d, *J* = 10.6 Hz, 1H, CH*H*Ph), 4.57 (d, $J_{1C,2C}$ = 7.8 Hz, 1H, H-1C), 4.52 (d, $J_{1A,2A}$ = 7.7 Hz, 1H, H-1A), 4.34 (dt, *J* = 10.3, 4.9 Hz, 2H, H-6aA, H-6aC), 4.19 (dd, *J* = 13.9, 6.2 Hz, 1H, H-5B), 3.99–3.90 (m, 3H, H-1a_{linker}, H-3A, H-3B), 3.82–3.72 (m, 3H, H-6bA, H-6bC, H-3C), 3.61–3.37 (m, 6H, H-1b_{linker}, H-4A, H-4C, H-2A, H-5A, H-5C), 3.21 (t, *J* = 7.2 Hz, 2H, H-5_{linker}), 2.80–2.71 (m, 2H, CH_{2Lev}), 2.67–2.57 (m, 2H, CH_{2Lev}), 2.17 (s, 3H, CH_{3Lev}), 2.10 (s, 3H, CH_{3Ac}), 2.04 (s, 3H, CH_{3Ac}), 1.69–1.56 (m, 4H, H-2_{linker}, H-4_{linker}), 1.50–1.40 (m, 2H, H-3_{linker}), 0.80 (d, *J* = 6.5 Hz, 3H, CH_{3Tal}), 0.77 (s, 9H, C(CH_3)$_3$), 0.00, –0.04 (2 × s, 6H, 2 × CH_3); ^{13}C NMR (100 MHz, $CDCl_3$) *δ* 206.6 (*C*O), 171.3 (*C*O), 170.9, 169.9 (2 × *C*O), 138.2, 137.2, 137.1 (3 × C-Ar), 129.4–126.3 (9 × *C*H-Ar), 104.2 (C-1A), 102.0 (C-7A), 101.9 (C-7C), 100.6 (C-1C), 98.9 (C-1B), 83.0 (C-2A), 81.3 (C-4C), 79.3 (C-4A), 76.1 (C-3A), 74.9 (*C*H_2Ph), 74.7 (C-2C), 72.8 (C-3C), 71.3 (C-3B), 70.2 (C-1_{linker}), 68.9 (C-6), 68.8 (C-4B), 68.7 (C-6C), 68.4 (C-2B), 66.3 (C-5A), 66.2 (C-5C), 64.7 (C-5B), 51.3 (C-5_{linker}), 37.9 (*C*H_{2Lev}), 30.0 (*C*H_{3Lev}), 29.4 (C-2_{linker}), 28.7 (C-4_{linker}), 28.1 (*C*H_{2Lev}), 25.7 (C(*C*H_3)$_3$), 23.4 (C-3_{linker}), 21.1, 20.9 (2 × *C*H_{3Ac}), 18.1 (*C*(CH$_3$)$_3$), 15.9 (*C*H_{3Tal}), –4.06, –4.87 (2 × *C*H_3); HRMS (ESI-TOF) *m/z* $[M + Na]^+$ calcd for $C_{59}H_{79}N_3NaO_{19}Si$ 1184.4969; found 1184.4986.

(5-Azido-1-pentyl) 3-*O*-Acetyl-4-*O*-benzyl-6-deoxy-*α*-L-talopyranosyl-(1→3)-2-*O*-benzyl-4,6-*O*-benzylidene-*β*-D-glucopyranoside (27).

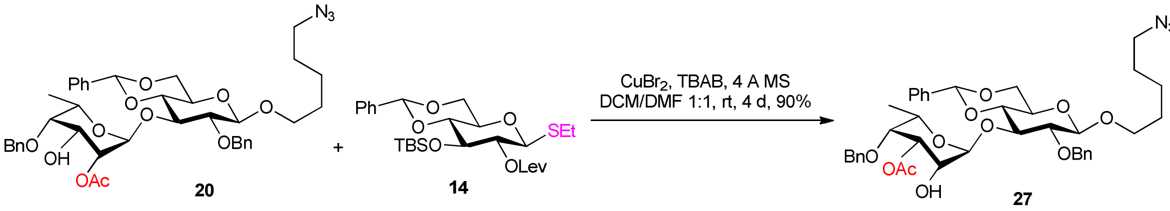

The acceptor **20** (15 mg, 20 *μ*mol, 1.0 equiv) and the donor **14** (21 mg, 40 *μ*mol, 2.0 equiv) were dissolved in anhydrous DCM/DMF (800 *μ*L, 1:1 *v/v*). Freshly activated powdered molecular sieves (4 Å, 60 mg) were added and the mixture was stirred for 1 h at rt under Ar. Then, Bu_4NBr (14 mg, 42 *μ*mol, 2.1 equiv) followed by $CuBr_2$ (9 mg, 40 *μ*mol, 2.0 equiv) were added and the mixture was stirred for 4 d at rt under Ar. The solution was filtered over Celite, rinsed and diluted with DCM (20 mL). The organic phase was washed with a saturated $NaHCO_3$(aq) solution (3 × 10 mL). The aqueous phase was back extracted with DCM (10 mL). The combined organic phases were washed with brine (15 mL) and the solvents of the dried ($MgSO_4$) solution were concentrated under reduced pressure. The residue was purified by silica gel flash chromatography (PE/EtOAc 85:15 to 8:2) to give alcohol **27** (14 mg, 90%) as a yellow oil: R_f 0.5 (tol/EtOAc 85:15); $[\alpha]_D^{20}$ = –58 (*c* 1.4, $CHCl_3$); ^1H NMR (400 MHz, $CDCl_3$) δ 7.47–7.13 (m, 15H, C*H*-Ar), 5.48 (s, 1H, H-7), 5.23 (d, *J* = 1.6 Hz, 1H, H-1B), 5.02 (t, *J* = 3.4 Hz, 1H, H-3B), 4.85 (d, *J* = 10.8 Hz, 1H, C*H*HPh), 4.66 (d, *J* = 10.8 Hz, 2H, CH_2Ph), 4.51 (d, *J* = 10.8 Hz, 1H, CH*H*Ph), 4.48 (d, $J_{1A,2A}$ = 7.8 Hz, 1H, H-1A), 4.32 (dd, *J* = 10.4, 5.0 Hz, 1H, H-6aA), 4.16 (dd, *J* = 13.8, 6.4 Hz, 1H, H-5B), 4.03 (d, *J* = 11.2 Hz, 1H, O*H*), 3.92 (dt, *J* = 9.8, 6.3 Hz, 1H, H-$1a_{linker}$), 3.89 (t, *J* = 9.5 Hz, 1H, H-3A), 3.76 (t, *J* = 10.4 Hz, 1H, H-6bA), 3.71 (br s, 1H, H-2B), 3.58–3.53 (m, 2H, H-4B, H-$1b_{linker}$), 3.51 (t, *J* = 9.8 Hz, 1H, H-4A), 3.45–3.39 (m, 2H, H-5A, H-2A), 3.22 (t, *J* = 7.3 Hz, 2H, H-5_{linker}), 2.09 (s, 3H, CH_{3Ac}), 1.70–1.58 (m, 4H, H-2_{linker}, H-4_{linker}), 1.52–1.42 (m, 2H, H-3_{linker}), 0.77 (d, *J* = 6.5 Hz, 3H, CH_{3Tal}); ^{13}C NMR (100 MHz, $CDCl_3$) δ 170.6 (*C*O), 137.8, 137.4, 137.3 (3 × C-Ar), 129.3–125.4 (9 × *C*H-Ar), 104.2 (C-1A), 102.2 (C-1B), 102.1 (C-7), 82.7 (C-2A), 79.4 (C-4A), 79.3 (C-4B), 77.6 (C-3A), 76.1, 75.2 (2 × *C*H_2Ph), 70.2 (C-1_{linker}), 70.1 (C-3B), 69.2 (C-2B), 68.9 (C-6), 66.5 (C-5A), 66.3 (C-5B), 51.4 (C-5_{linker}), 29.4 (C-2_{linker}), 28.7 (C-4_{linker}), 23.5 (C-3_{linker}), 21.3 (*C*H_{3Ac}), 16.2 (*C*H_{3Tal}); HRMS (ESI-TOF) *m/z* $[M + Na]^+$ calcd for $C_{40}H_{49}N_3NaO_{11}$ 770.3259; found 770.3266; *m/z* $[2M + Na]^+$ calcd for $C_{80}H_{98}N_6NaO_{22}$ 1517.6626; found 1517.6666.

(5-Azido-1-pentyl) 2-*O*-Acetyl-4-*O*-benzyl-3-*O*-*tert*-butyldimethylsilyl-6-deoxy-*α*-L-talopyranosyl-(1→3)-2-*O*-benzyl-4,6-*O*-benzylidene-*β*-D-glucopyranoside (28).

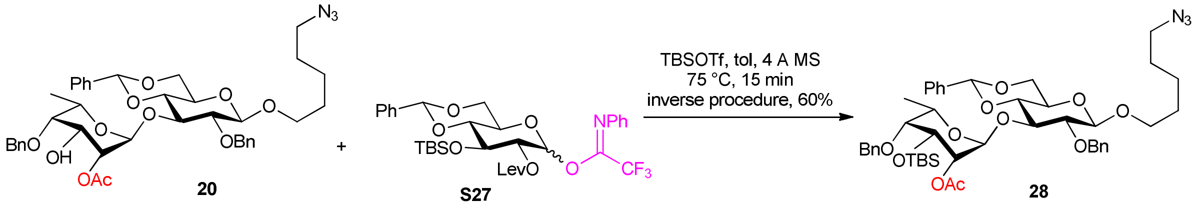

To a solution of acceptor **20** (15 mg, 20 *μ*mol, 1.0 equiv) in anhydrous toluene (1 mL) was added freshly activated powdered molecular sieves (4 Å, 171 mg) and the mixture was stirred for 40 min at rt under Ar. TBSOTf (2 *μ*L, 9 *μ*mol, 0.3 equiv) was injected keeping rigorous anhydrous conditions and the mixture was heated at 75 °C for 15 min. A solution of donor **S27** (20 mg, 30 *μ*mol, 1.5 equiv) in anhydrous toluene (1 mL) was added dropwise at the same temperature over 10 min to the former mixture. After stirring for 1 h at 75 °C, the reaction mixture was allowed to slowly warm up to rt and then quenched with few drops of Et_3N. The suspension was filtered over Celite, rinsed with DCM and the filtrate was concentrated under reduced pressure. The residue was purified by silica gel flash chromatography (tol/EtOAc 95:5) to give silylated derivative **28** (10 mg, 60%) as a yellow oil: R_f 0.5 (tol/EtOAc 85:15); $[\alpha]_D^{20}$ = –32 (*c* 0.9, $CHCl_3$); ^{1}H NMR (400 MHz, $CDCl_3$) δ 7.46–7.20 (m, 15H, C*H*-Ar), 5.52 (s, 1H, H-7), 5.28 (s, 1H, H-1B), 5.16 (d, *J* = 4.1 Hz, 1H, H-2B), 4.92 (d, *J* = 11.6 Hz, 1H, C*H*HPh), 4.88 (d, *J* = 10.7 Hz, 1H, CH*H*Ph), 4.68 (d, *J* = 10.7 Hz, 1H, C*H*HPh), 4.56 (d, *J* = 11.6 Hz, 1H, CH*H*Ph), 4.50 (d, $J_{1A,2A}$ = 7.8 Hz, 1H, H-1A), 4.33 (dd, *J* = 10.7, 4.8 Hz, 1H, H-6aA), 4.13 (dd, *J* = 13.9, 6.4 Hz, 1H, H-5B), 4.00–3.96 (m, 2H, H-3A, H-3B), 3.91 (dt, *J* = 9.6, 6.4 Hz, 1H, H-$1a_{linker}$), 3.77 (t, *J* = 10.7 Hz, 1H, H-6bA), 3.58–3.52 (m, 2H, H-$1b_{linker}$, H-4A), 3.48–3.39 (m, 2H, H-2A, H-5A), 3.23 (t, *J* = 1.6 Hz, 1H, H-4B), 3.19 (t, *J* = 7.2 Hz, 2H, H-5_{linker}), 1.96 (s, 3H, CH_{3Ac}), 1.68–1.54 (m, 4H, H-2_{linker}, H-4_{linker}), 1.47–1.40 (m, 2H, H-3_{linker}), 0.92 (d, *J* = 6.4 Hz, 3H, CH_{3Tal}), 0.89 (s, 9H, $C(CH_3)_3$), 0.12, 0.11 (2 × s, 6H, 2 × CH_3); ^{13}C NMR (100 MHz, $CDCl_3$) δ 170.7 (*C*O), 139.5, 138.2, 137.5 (3 × C-Ar), 129.3–126.3 (9 × *C*H-Ar), 104.2 (C-1A), 101.8 (C-7), 99.1 (C-1B), 83.2 (C-2A), 79.3 (C-4A), 78.8 (C-4B), 75.5 (C-3B), 74.8 (2 × CH_2Ph), 70.2 (C-1_{linker}), 70.0 (C-2B), 69.2 (C-3A), 68.9 (C-6), 66.8 (C-5B), 66.4 (C-5A), 51.4 (C-5_{linker}), 29.4 (C-2_{linker}), 28.7 (C-4_{linker}), 25.9 ($C(CH_3)_3$), 23.5 (C-3_{linker}), 21.2 (CH_{3Ac}), 18.1 ($C(CH_3)_3$), 16.7 (CH_{3Tal}), –4.71, –4.82 (2 × CH_3); HRMS (ESI-TOF) *m/z* $[M + Na]^+$ calcd for $C_{46}H_{63}N_3NaO_{11}Si$ 884.4124; found 884.4151.

4,6-*O*-Benzylidene-3-*O*-*tert*-butyldimethylsilyl-2-*O*-levulinoyl-*β*-D-glucopyranosyl-(1↔1)-4,6-*O*-benzylidene-3-*O*-*tert*-butyldimethylsilyl-2-*O*-levulinoyl-*α*-D-glucopyranoside (29).

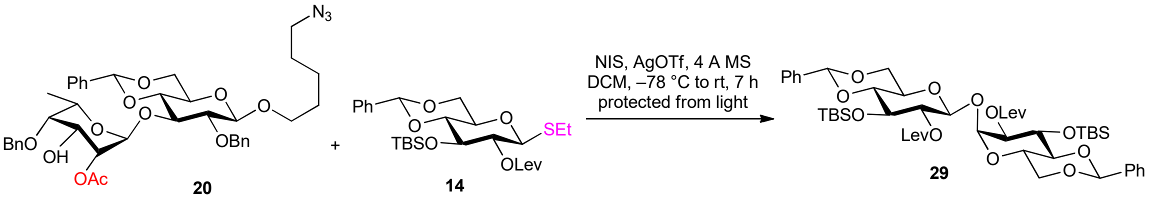

To a solution of donor **14** (28 mg, 53 μmol, 2.0 equiv) and acceptor **20** (20 mg, 27 μmol, 1.0 equiv) in anhydrous DCM (500 μL) was added freshly activated 4 Å molecular sieves (80 mg). The mixture was stirred at rt for 30 min under Ar. Then, the suspension was cooled to –78 °C and AgOTf (7 mg, 27 μmol, 1.0 equiv) followed by NIS (12 mg, 53 μmol, 2.0 equiv) were added. The flask was protected from light and the reaction mixture was stirred from –78 °C to rt for 7 h under Ar and then quenched with a few drops of Et_3N. The suspension was filtered over Celite, rinsed with DCM and the filtrate was concentrated under reduced pressure. The residue was purified by silica gel flash chromatography (tol/EtOAc 9:1) to give dimer **29** (major compound, variable yields) as a colorless oil: R_f 0.7 (tol/EtOAc 85:15); $[\alpha]_D^{20}$ = –47 (*c* 0.77, $CHCl_3$); ^{1}H NMR (400 MHz, $CDCl_3$) δ 7.49–7.46 (m, 4H, C*H*-Ar), 7.37–7.30 (m, 6H, C*H*-Ar), 6.15 (d, *J* = 4.0 Hz, 1H, H-1A), 5.51 (s, 1H, H-7A), 5.45 (s, 1H, H-7B), 4.90 (d, *J* = 7.7 Hz, 1H, H-1B), 4.83 (t, *J* = 8.5 Hz, 1H, H-2B), 4.33 (dd, *J* = 10.7, 4.9 Hz, 1H, H-6aA), 4.28 (dd, *J* = 10.4, 4.8 Hz, 1H, H-6aB), 4.12 (t, *J* = 9.2 Hz, 1H, H-3A), 3.93 (dd, *J* = 8.4, 3.9 Hz, 1H, H-2A), 3.88 (dd, *J* = 9.8, 4.7 Hz, 1H, H-5A), 3.80 (t, *J* = 9.0 Hz, 1H, H-3B), 3.78 (t, *J* = 10.1 Hz, 1H, H-6bB), 3.66 (t, *J* = 10.7 Hz, 1H, H-6bA), 3.56 (t, *J* = 9.4 Hz, 1H, H-4B), 3.47 (t, *J* = 9.7 Hz, 1H, H-4A), 3.37 (dd, *J* = 9.7, 4.7 Hz, 1H, H-5), 2.93–2.49 (m, 8H, 4 × CH_{2Lev}), 2.22, 2.21 (2 × s, 6H, 2 × CH_{3Lev}), 0.85, 0.79 (2 × s, 18H, 2 × C(CH_3)$_3$), –0.01, –0.00 (2 × s, 12H, 4 × CH_3); ^{13}C NMR (100 MHz, $CDCl_3$) δ 206.8, 205.9 (2 × *C*O), 171.6, 171.4 (2 × *C*O), 137.1, 137.0 (2 × C-Ar), 129.4–126.3 (6 × *C*H-Ar), 102.6 (C-7B), 101.9 (C-7A), 100.5 (C-1B), 92.2 (C-1A), 81.8 (C-4A), 81.6 (C-4B), 75.6 (C-2A), 75.1 (C-2B), 72.6 (C-3B), 72.3 (C-3A), 68.9 (C-6B), 68.7 (C-6A), 66.5 (C-5B), 64.5 (C-5A), 38.1, 37.8 (2 × *C*H_{2Lev}), 30.0 (2 × *C*H_{3Lev}), 28.1, 28.0 (2 × *C*H_{2Lev}), 26.1, 25.7 (2 × C(*C*H_3)$_3$), 18.4, 18.1 (2 × *C*(CH_3)$_3$), –3.74, –4.10, –4.45, –4.84 (4 × *C*H_3); HRMS (ESI-TOF) *m/z* [M + Na]$^+$ calcd for $C_{48}H_{70}NaO_{15}Si_2$ 965.4145; found 965.4165.

Allyl 6-Deoxy-2,3-*O*-isopropylidene-4-*O*-levulinoyl-*α*-L-talopyranoside (**S28**).

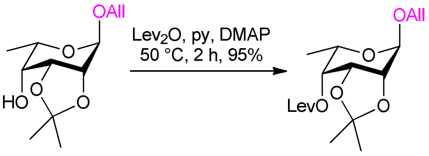

S2 **S28**

Alcohol **S2** (3.5 g, 14.9 mmol, 1.0 equiv) was dissolved in anhydrous py (95 mL) and DMAP (4.4 g, 35.9 mmol, 2.5 equiv) was added. A solution of levulinic anhydride[5] (24.6 g, 115 mmol, 8.0 equiv) in anhydrous py (127 mL) was added dropwise over 50 min to the former mixture. The reaction mixture was then heated to 50 °C and stirred under Ar for an additional 2 h. The solvents were concentrated under reduced pressure and the residue was purified by silica gel flash chromatography (PE/EtOAc, 8:2 to 7:3) to give derivative **S28** (4.7 g, 95%) as a yellow oil: R_f 0.5 (PE/EtOAc, 6:4); $[\alpha]_D^{20}$ = –11 (*c* 2.1, $CHCl_3$); ^{1}H NMR (400 MHz, $CDCl_3$) δ 5.96–5.86 (m, 1H, H-2_{All}), 5.31 (ddd, *J* = 17.2, 3.5, 1.5 Hz, 1H, H-$3a_{All}$), 5.22 (ddd, *J* = 10.3, 3.3, 1.2 Hz, 1H, H-$3b_{All}$), 5.10 (dd, *J* = 5.5, 2.2 Hz, 1H, H-4), 5.08 (s, 1H, H-1), 4.39 (t, *J* = 6.4 Hz, 1H, H-3), 4.20 (ddt , *J* = 12.8, 5.3, 1.4 Hz, 1H, H-1aAll), 4.09 (dd, *J* = 6.5, 09 Hz, 1H, H-2), 4.03–3.98 (m, 2H, H-$1b_{All}$, H-5), 2.87–2.61 (m, 4H, 2 × CH_{2Lev}), 2.19 (s, 3H, CH_{3Lev}), 1.52, 1.34 (2 × s, 6H, 2 × CH_3), 1.23 (d, *J* = 6.5 Hz, 3H, CH_{3Tal}); ^{13}C NMR (100 MHz, $CDCl_3$) δ 206.4 (*C*O), 172.7 (*C*O), 133.7 (C-2_{All}), 118.0 (C-3_{All}), 109.8 (*C*(CH_3)$_2$), 96.9 (C-1), 73.2 (C-2), 71.1 (C-3), 68.5 (C-1_{All}), 67.6 (C-4), 63.6 (C-5), 38.0 (*C*H_{2Lev}), 30.0 (*C*H_{3Lev}), 28.3 (*C*H_{2Lev}), 26.3, 25.8 (2 × *C*H_3), 16.6 (*C*H_{3Tal}); HRMS (ESI-TOF) *m/z* $[M + Na]^+$ calcd for $C_{17}H_{26}NaO_7$ 365.1571; found 365.1580.

Allyl 2-*O*-Acetyl-3-*O*-chloroacetyl-6-deoxy-4-*O*-levulinoyl-*α*-L-talopyranoside (S30).

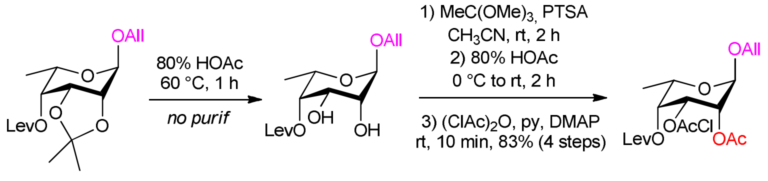

Compound **S28** (4.6 g, 13.6 mmol, 1.0 equiv) was dissolved in a 80% HOAc(aq) solution (170 mL). The reaction mixture was stirred at 60 °C for 1 h. Then, the mixture was concentrated under reduced pressure and co-evaporated with toluene (3 ×). Crude diol **S29** was obtained as a yellow amorphous solid [R_f 0.3 (DCM/MeOH 98:2)], which was used directly for the next step without purification in order to avoid migration of the levulinoyl group. Diol **S29** (250 mg, 730 μmol, 1.0 equiv) was dissolved in anhydrous acetonitrile (3 mL). Trimethyl orthoactetate (186 μL, 1.5 mmol, 2.0 equiv) and PTSA (7 mg, 37 μmol, 0.05 equiv) were added sequentially. The reaction mixture was stirred for 2 h at rt under Ar. The suspension was then cooled to 0 °C and a 80% HOAc(aq) solution (3 mL) was added. The mixture was stirred at 0 °C for 10 min, then allowed to slowly warm up to rt. After 2 h, cooled water (20 mL) was added and the mixture was diluted with DCM (30 mL). The aqueous layer was extracted with DCM (2 × 10 mL). The combined organic phases were washed with brine (30 mL). The solvents of the dried solution ($MgSO_4$) were concentrated under reduced pressure. The residue was dissolved in anhydrous py (6 mL), then chloroacetyl anhydride (437 mg, 2.5 mmol, 3.5 equiv) and DMAP (9 mg, 73 μmol, 0.1 equiv) were added. The reaction mixture was stirred at rt for 10 min under Ar. Then, the suspension was diluted with EtOAc (30 mL) and the organic phase was washed with a saturated NH_4Cl(aq) solution (3 × 15 mL) and brine (20 mL). The solvents of the dried solution ($MgSO_4$) were concentrated under reduced pressure. The residue was purified by silica gel flash chromatography (PE/EtOAc 8:2 to 6:4) to give derivative **S30** (254 mg, 83%, four steps) as a yellow oil: R_f 0.3 (tol/EtOAc 8:2); $[\alpha]_D^{20}$ = –57 (*c* 1.3, $CHCl_3$); ^{1}H NMR (400 MHz, $CDCl_3$) δ 5.93–5.83 (m, 1H, H-2_{All}), 5.35 (t, J = 4.0 Hz, 1H, H-3), 5.30 (ddd, J = 17.2, 3.6, 1.6 Hz, 1H, H-$3a_{All}$), 5.23 (ddd, J = 10.4, 3.1, 2.0 Hz, 1H, H-$3b_{All}$), 5.19 (d, J = 3.7 Hz, 1H, H-4), 5.17 (dt, J = 3.9, 1.5 Hz, 1H, H-2), 4.91 (d, $J_{1,2}$ = 1.1 Hz, 1H, H-1), 4.20–4.13 (m, 2H, H-1aAll, H-5), 4.02 (ddt, J = 13.3, 6.1, 1.6 Hz, 1H, H-$1b_{All}$), 4.01 (d, J = 2.9 Hz, 2H, CH_{2Cl}), 2.91–2.82 (m, 1H, CHH$_{Lev}$), 2.75–2.58 (m, 3H, CH_{2Lev}), 2.20 (CH_{3Lev}), 2.19 (s, 3H, CH_{3Ac}), 1.23 (d, J = 6.4 Hz, 3H, CH_{3Tal}); ^{13}C NMR (100 MHz, $CDCl_3$) δ 206.4 (CO), 172.8, 170.4, 166.4 (3 × CO), 133.2 (C-2_{All}), 118.3 (C-3_{All}), 97.5 (C-1), 68.8 (C-4), 68.6 (C-1_{All}), 67.9 (C-3), 66.8 (C-2), 64.7 (C-5), 40.7 (CH$_{2Cl}$), 37.7 (CH$_{2Lev}$), 30.0 (CH$_{3Lev}$), 27.8 (CH$_{2Lev}$), 21.1 (CH$_{3Ac}$), 16.2 (CH$_{3Tal}$); HRMS (ESI-TOF) *m/z* $[M + Na]^+$ calcd for $C_{18}H_{25}ClNaO_9$ 443.1079; found 443.1097.

Allyl 4,6-*O*-Benzylidene-3-*O*-*tert*-butyldimethylsilyl-2-*O*-levulinoyl-*β*-D-glucopyranosyl-(1→2)-6-deoxy-4-*O*-levulinoyl-*α*-L-talopyranoside (**S32**).

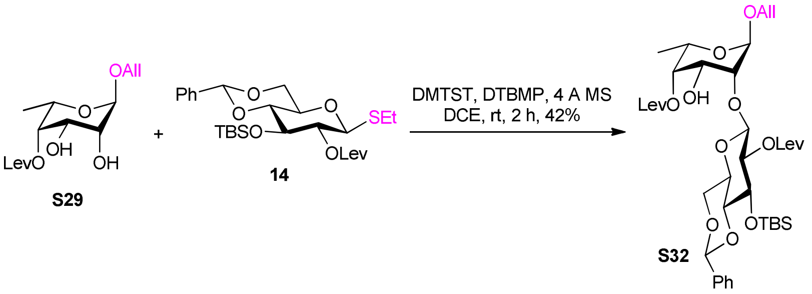

To a solution of donor **14** (56 mg, 108 *μ*mol, 1.3 equiv), acceptor **S29** (25 mg, 83 *μ*mol, 1.0 equiv) and DTBMP (51 mg, 248 *μ*mol, 3.0 equiv) in anhydrous DCE (1.5 mL) was added freshly activated 4 Å powdered molecular sieves (100 mg). The mixture was stirred for 30 min at rt under Ar. Then, Me_2S_2 (22 *μ*L, 248 *μ*mol, 3.0 equiv) and MeOTf (28 *μ*L, 248 *μ*mol, 3.0 equiv) were added. The solution was stirred for an additional 2 h at rt. Then, the reaction mixture was quenched with few drops of Et_3N, filtered over Celite and rinsed with DCM. The filtrate was concentrated under reduced pressure and purified by silica gel flash chromatography (PE/EtOAc 7:3 to 4:6) to give disaccharide **S32** (26 mg, 42%, major regioisomer) as a yellow oil: R_f 0.5 (tol/EtOAc 5:5); $[\alpha]_D^{20}$ = –5.6 (*c* 2.5, $CHCl_3$); ^{1}H NMR (400 MHz, $CDCl_3$) δ 7.42–7.40 (m, 2H, C*H*-Ar), 7.31–7.28 (m, 3H, C*H*-Ar), 5.88–5.78 (m, 1H, H-2_{All}), 5.64 (d, $J_{1C,2C}$ = 5.7 Hz, 1H, H-1C), 5.47 (s, 1H, H-7), 5.22 (ddd, *J* = 17.2, 3.7, 1.8 Hz, 1H, H-$3a_{All}$), 5.15 (ddd, *J* = 10.4, 3.3, 1.2 Hz, 1H, H-$3b_{All}$), 5.04 (d, *J* = 3.5 Hz, 1H, H-4B), 4.84 (d, $J_{1A,2A}$ = 1.4 Hz, 1H, H-1B), 4.30 (dd, *J* = 10.6, 3.8 Hz, 1H, H-6aC), 4.24 (dd, *J* = 5.6, 3.9 Hz, 1H, H-2C), 4.10 (ddt, *J* = 13.0, 5.1, 1.4 Hz, 1H, H-$1a_{All}$), 3.97–3.93 (m, 3H, H-$1b_{All}$, H-3B, H-5B), 3.86 (dd, *J* = 8.8, 3.8 Hz, 1H, H-3C), 3.65–3.58 (m, 3H, H-2B, H-5C, H-6bC), 3.46 (t, *J* = 9.3 Hz, 1H, H-4C), 2.79–2.67 (m, 4H, 2 × CH_{2Lev}), 2.64–2.54 (m, 4H, 2 × CH_{2Lev}), 2.13 (s, 3H, CH_{3Lev}), 2.11 (s, 3H, CH_{3Lev}), 1.09 (d, *J* = 6.6 Hz, 3H, CH_{3Tal}), 0.82 (s, 9H, $C(CH_3)_3$), 0.45, 0.00 (2 × s, 6H, 2 × CH_3); ^{13}C NMR (100 MHz, $CDCl_3$) δ 207.4, 206.2 (2 × *C*O), 172.2, 172.1 (2 × *C*O), 137.2 (C-Ar), 133.7 (C-2_{All}), 129.8, 128.3, 126.1 (3 × *C*H-Ar), 117.7 (C-3_{All}), 101.4 (C-7), 99.8 (C-1B), 98.8 (C-1C), 80.5 (C-2C), 80.1 (C-4C), 74.4 (C-3C), 73.1 (C-4B), 69.7 (C-2B), 68.7 (C-6C), 68.4 (C-1_{All}), 66.8 (C-3B), 65.1 (C-5B), 63.3 (C-5C), 38.2, 38.0 (2 × CH_{2Lev}), 30.2, 30.1 (2 × CH_{3Lev}), 28.0 (2 × CH_{2Lev}), 25.8 ($C(CH_3)_3$), 18.2 ($C(CH_3)_3$), 16.3 (CH_{3Tal}), –4.40, –4.82 (2 × CH_3); HRMS (ESI-TOF) *m/z* $[M + Na]^+$ calcd for $C_{38}H_{56}NaO_{14}Si$ 787.3332; found 787.3347.

Allyl 2,3,4,6-Tetra-*O*-benzyl-*β*-D-glucopyranosyl-(1→3)-6-deoxy-*α*-L-talopyranoside (**S34**).

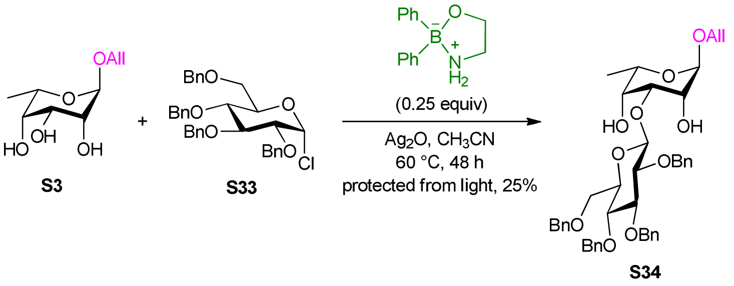

To a solution of donor **S33**[6] (75 mg, 134 *μ*mol, 1.0 equiv) and acceptor **S3** (30 mg, 147 *μ*mol, 1.1 equiv) in anhydrous acetonitrile (1.5 mL) were added silver(I) oxide (62 mg, 267 *μ*mol, 2.0 equiv) and 2-aminoethyl diphenylborinate (8 mg, 33 *μ*mol, 0.25 equiv). The flask was purged with a stream of Ar for 5 min, then protected from light. The reaction mixture was stirred at 60 °C under Ar for 48 h. The solution was then quenched with a few drops of MeOH, diluted with DCM and filtered through a plug of Celite. The filtrate was concentrated under reduced pressure. The residue was purified by silica gel flash chromatography (tol/EtOAc 95:5 to 85:15) to give disaccharide **S34** (25 mg, 25%, major regioisomer) as a yellow amorphous solid: R_f 0.5 (tol/EtOAc 7:3); $[\alpha]_D^{20}$ = –14 (*c* 2.4, $CHCl_3$); ^1H NMR (400 MHz, $CDCl_3$) δ 7.38–7.25 (m, 18H, C*H*-Ar), 7.19–7.15 (m, 2H, C*H*-Ar), 5.92–5.82 (m, 1H, H-2_{All}), 5.27 (ddd, *J* = 17.2, 3.8, 1.5 Hz, 1H, H-$3a_{All}$), 5.19 (ddd, *J* = 10.4, 3.4, 1.2 Hz, 1H, H-$3b_{All}$), 4.97 (d, *J* = 11.3 Hz, 1H, C*H*HPh), 4.92 (d, $J_{1B,2B}$ = 1.6 Hz, 1H, H-1B), 4.91 (d, *J* = 10.8 Hz, 1H, CH*H*Ph), 4.82 (d, *J* = 10.8 Hz, 1H, C*H*HPh), 4.81 (d, *J* = 11.3 Hz, 1H, CH*H*Ph), 4.80 (d, *J* = 10.8 Hz, 1H, C*H*HPh), 4.60 (d, $J_{1B,2B}$ = 7.7 Hz, 1H, H-1C), 4.53 (d, *J* = 11.8 Hz, 1H, CH*H*Ph), 4.52 (d, *J* = 10.8 Hz, 1H, C*H*HPh), 4.48 (d, *J* = 11.8 Hz, 1H, CH*H*Ph), 4.16 (ddt, *J* = 13.0, 5.2, 1.4 Hz, 1H, H-$1a_{All}$), 4.01–3.95 (m, 2H, H-$1b_{All}$, H-2B), 3.89–3.83 (m, 2H, H-3B, H-5B), 3.69–3.63 (m, 3H, H-4B, H-4C, H-6aC), 3.61–3.49 (m, 5H, H-2C, H-3C, H-5C, H-6bC, O*H*), 3.29 (d, *J* = 8.8 Hz, 1H, O*H*), 1.30 (d, *J* = 6.6 Hz, 3H, CH_{3Tal}); ^{13}C NMR (100 MHz, $CDCl_3$) δ 138.4, 138.3, 137.9, 137.8 (4 × C-Ar), 133.8 (C-2_{All}), 128.4–127.5 (12 × *C*H-Ar), 117.6 (C-3_{All}), 101.8 (C-1C), 99.3 (C-1B), 84.7 (C-4B), 81.9 (C-2C), 77.7 (C-3C), 75.7 (C-3B), 75.8 (*C*H_2Ph), 75.1 (*C*H_2Ph), 74.9 (*C*H_2Ph), 74.6 (C-5B), 73.5 (*C*H_2Ph), 70.8 (C-4C), 69.4 (C-2B), 69.0 (C-6C), 68.6 (C-1_{All}), 66.8 (C-5B), 16.5 (*C*H_{3Tal}); HRMS (ESI-TOF) *m/z* $[M + Na]^+$ calcd for $C_{43}H_{57}NaO_{10}$ 749.3296; found 749.3303; *m/z* $[2M + Na]^+$ calcd for $C_{86}H_{100}NaO_{20}$ 1475.6700; found 1475.6737.

Allyl 2,3,4,6-Tetra-*O*-benzyl-*β*-D-glucopyranosyl-(1→2)-3-*O*-acetyl-4-*O*-benzyl-6-deoxy-*α*-L-talopyranoside (S35).

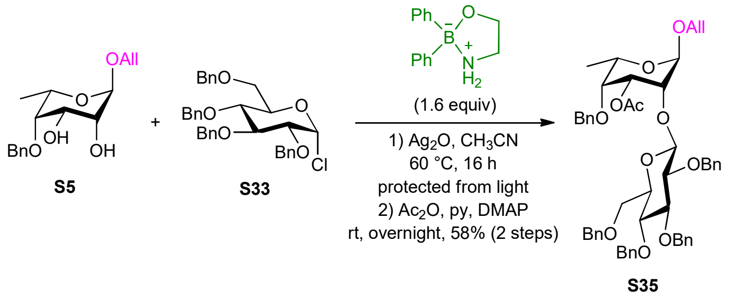

To a solution of donor **S33**[6] (52 mg, 93 *µ*mol, 1.0 equiv) and acceptor **S5** (30 mg, 102 *µ*mol, 1.1 equiv) in anhydrous acetonitrile (3 mL) were added silver(I) oxide (42 mg, 186 *µ*mol, 2.0 equiv) and 2-aminoethyl diphenylborinate (34 mg, 149 *µ*mol, 1.6 equiv). The round bottom flask was purged with a stream of Ar for 5 min, then protected from light and the reaction mixture was stirred at 60 °C under Ar. After 16 h, the reaction was quenched with a few drops of MeOH, diluted with DCM and filtered through a plug of Celite. The filtrate was concentrated under reduced pressure. The residue was dissolved in anhydrous py (0.4 mL), then Ac_2O (0.4 mL) and DMAP (1.1 mg, 9 *µ*mol, 0.1 equiv) were added. The suspension was stirred at rt overnight under Ar. Then, the mixture was concentrated under reduced pressure and co-evaporated with toluene (3 ×). The residue was purified by silica gel flash chromatography (tol/EtOAc 98:2 to 96:4) to give disaccharide **S35** (45 mg, 58%, two steps) as a yellow oil: R_f 0.5 (tol/EtOAc 9:1); $[\alpha]_D^{20}$ = –1.3 (*c* 1.1, $CHCl_3$); ^1H NMR (400 MHz, $CDCl_3$) δ 7.38–7.14 (m, 25H, C*H*-Ar), 5.88–5.78 (m, 1H, H-2_{All}), 5.29 (s, 1H, H-1B), 5.24 (d, *J* = 12.2 Hz, 1H, C*H*HPh), 5.23–5.18 (m, 1H, H-$3a_{All}$), 5.13 (t, *J* = 4.0 Hz, 1H, H-3B), 5.10 (ddd, *J* = 10.4, 3.3, 1.2 Hz, 1H, H-$3b_{All}$), 4.85 (d, *J* = 10.2 Hz, 1H, CH*H*Ph), 4.81 (d, *J* = 10.8 Hz, 1H, C*H*HPh), 4.72 (d, *J* = 12.1 Hz, 1H, CH*H*Ph), 4.71 (d, *J* = 10.8 Hz, 1H, C*H*HPh), 4.62 (d, *J* = 12.1 Hz, 1H, CH*H*Ph), 4.55–4.49 (m, 4H, 2 × CH_2Ph), 4.46 (d, $J_{1C,2C}$ = 7.5 Hz, 1H, H-1C), 4.07 (ddt, *J* = 12.7, 5.2, 1.5 Hz, 1H, H-$1a_{All}$), 3.96 (dd, *J* = 13.7, 6.8 Hz, 1H, H-5B), 3.91–3.89 (m, 1H, H-2B), 3.86 (ddt, *J* = 13.0, 5.9, 1.7 Hz, 1H, H-$1b_{All}$), 3.67–3.65 (m, 2H, H-6aC, H-6bC), 3.62–3.59 (m, 3H, H-2C, H-3C, H-4B), 3.52 (dd, *J* = 9.5, 7.7 Hz, 1H, H-4C), 3.47–3.39 (m, 1H, H-5C), 1.87 (s, 3H, CH_{3Ac}), 1.19 (d, *J* = 6.6 Hz, 3H, CH_{3Tal}); ^{13}C NMR (100 MHz, $CDCl_3$) δ 170.7 (*C*O), 139.5, 138.7, 138.3, 138.2, 138.1 (5 × C-Ar), 134.2 (C-2_{All}), 129.2–127.5 (15 × *C*H-Ar), 117.1 (C-3_{All}), 106.4 (C-1C), 99.8 (C-1B), 84.7 (C-3C), 82.1 (C-4B), 77.8 (C-4C), 76.7 (C-2B), 75.8 (*C*H_2Ph), 75.4 (C-2C), 75.1 (*C*H_2Ph), 74.9 (*C*H_2Ph), 74.8 (C-5C), 74.1 (*C*H_2Ph), 73.6 (*C*H_2Ph), 70.6 (C-3B), 69.5 (C-6C), 68.2 (C-1_{All}), 66.2 (C-5B), 21.1 (*C*H_{3Ac}), 16.9 (*C*H_{3Tal}); HRMS (ESI-TOF) *m/z* $[M + Na]^+$ calcd for $C_{52}H_{58}N_3NaO_{11}$ 881.3871; found 881.3885.

Allyl 2,3-*O*-Isopropylidene-4-*O*-levulinoyl-*α*-L-rhamnopyranoside (**30**).

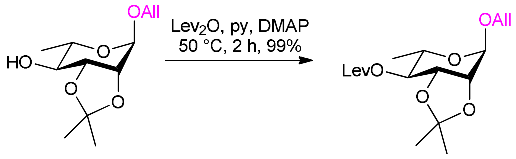

S1 **30**

Alcohol **S1** (9.8 g, 40 mmol, 1.0 equiv) was dissolved in anhydrous py (240 mL) and DMAP (9.8 g, 80 mmol, 2.0 equiv) was added. A solution of levulinic anhydride[5] (25.7 g, 120 mmol, 3.0 equiv) in anhydrous py (200 mL) was added dropwise over 1 h to the former mixture. The reaction mixture was then heated to 50 °C and stirred under Ar for an additional 2 h. The solvents were concentrated under reduced pressure and the residue was purified by silica gel flash chromatography (PE/EtOAc 9:1 to 75:25) to give derivative **30** (13.6 g, 99%) as a yellow oil: R_f 0.5 (tol/EtOAc 8:2); $[\alpha]_D^{20}$ = –10 (*c* 1.7, $CHCl_3$); ^{1}H NMR (400 MHz, $CDCl_3$) δ 5.96–5.86 (m, 1H, H-2_{All}), 5.32 (ddd, *J* = 17.2, 3.5, 1.6 Hz, 1H, H-$3a_{All}$), 5.23 (ddd, *J* = 10.4, 3.0, 1.2 Hz, 1H, H-$3b_{All}$), 5.05 (s, 1H, H-1), 4.85 (dd, *J* = 10.3, 7.1 Hz, 1H, H-4), 4.21–4.15 (m, 3H, H-$1a_{All}$, H-2, H-3), 4.01 (ddt, *J* = 12.8, 6.2, 1.3 Hz, 1H, H-$1b_{All}$), 3.80–3.72 (m, 1H, H-5), 2.91–2.84 (m, 1H, CH_{2Lev}), 2.72–2.52 (m, 3H, CH_{2Lev}), 2.19 (s, 3H, CH_{3Lev}), 1.55 (s, 3H, CH_3), 1.34 (s, 3H, CH_3), 1.19 (d, *J* = 6.3 Hz, 3H, CH_{3Tal}); ^{13}C NMR (100 MHz, $CDCl_3$) δ 206.5 (*C*O), 172.2 (*C*O), 133.7 (C-2_{All}), 118.0 (C-3_{All}), 109.9 ($C(CH_3)_2$), 96.2 (C-1), 76.1 (C-2), 75.8 (C-3), 74.9 (C-4), 68.2 (C-1_{All}), 64.2 (C-5), 38.0 (CH_{2Lev}), 29.9 (CH_{3Lev}), 28.1 (CH_{2Lev}), 27.8, 26.5 (2 × CH_3), 17.0 (CH_{3Tal}); HRMS (ESI-TOF) *m/z* $[M + Na]^+$ calcd for $C_{17}H_{26}NaO_7$ 365.1571; found 365.1585.

Allyl 4-*O*-Levulinoyl-*α*-L-rhamnopyranoside (70).

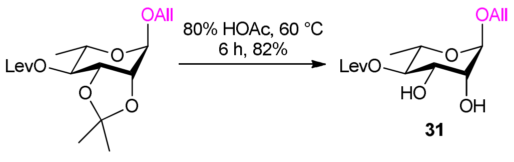

Compound **30** (13.6 g, 39.8 mmol, 1.0 equiv) was dissolved in a 80% HOAc(aq) solution (500 mL). The reaction mixture was stirred at 60 °C for 6 h. Then, the mixture was concentrated under reduced pressure and co-evaporated with toluene (3 ×). Purification by silica gel flash chromatography (DCM/MeOH 98:2 to 96:4) gave diol **31** (9.9 g, 82%) as a white amorphous solid: R_f 0.3 (DCM/MeOH 95:5); $[\alpha]_D^{20}$ = –71 (*c* 1.2, $CHCl_3$); ^{1}H NMR (400 MHz, $CDCl_3$) δ 5.95–5.85 (m, 1H, H-2_{All}), 5.30 (ddd, *J* = 17.2, 3.7, 1.5 Hz, 1H, H-$3a_{All}$), 5.21 (ddd, *J* = 10.4, 3.4, 1.3 Hz, 1H, H-$3b_{All}$), 4.91 (t, *J* = 9.7 Hz, 1H, H-4), 4.86 (s, 1H, H-1), 4.18 (ddt, *J* = 12.9, 5.1, 1.4 Hz, 1H, H-$1a_{All}$), 4.03–3.97 (m, 2H, H-$1b_{All}$, H-2), 3.94 (dd, *J* = 9.4, 3.5 Hz, 1H, H-3), 3.84–3.77 (m, 1H, H-5), 2.82 (t, *J* = 6.7 Hz, 1H, C*H*H_{Lev}), 2.60–2.56 (m, 3H, CH_{2Lev}), 2.20 (s, 3H, CH_{3Lev}), 1.21 (d, *J* = 6.2 Hz, 3H, CH_{3Tal}); ^{13}C NMR (100 MHz, $CDCl_3$) δ 207.6 (*C*O), 173.5 (*C*O), 133.8 (C-2_{All}), 117.6 (C-3_{All}), 98.6 (C-1), 75.6 (C-4), 71.0 (C-2), 70.2 (C-3), 68.2 (C-1_{All}), 65.8 (C-5), 38.4 (CH_{2Lev}), 29.9 (CH_{3Lev}), 28.3 (CH_{2Lev}), 17.4 (CH_{3Tal}); HRMS (ESI-TOF) *m/z* $[M + Na]^+$ calcd for $C_{14}H_{22}NaO_7$ 325.1258; found 325.1258; *m/z* $[2M + Na]^+$ calcd for $C_{28}H_{44}NaO_{14}$ 627.2623; found 627.2654.

Allyl 2,3,4,6-Tetra-*O*-benzyl-*β*-D-glucopyranosyl-(1→3)-2-*O*-acetyl-4-*O*-levulinoyl-*α*-L-rhamnopyranoside (32).

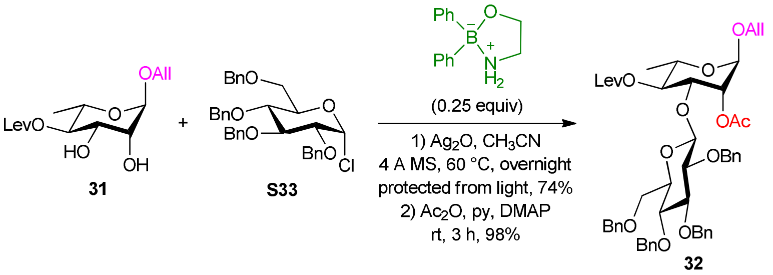

To a solution of donor **S33** (13.4 mg, 2.4 mmol, 1.5 equiv) and acceptor **31** (482 mg, 1.6 mmol, 1.0 equiv) in anhydrous acetonitrile (32 mL) was added freshly activated 4 Å powdered molecular sieves (2.0 g) and the suspension was stirred for 1 h at rt under Ar. Then, silver(I) oxide (738 mg, 3.2 mmol, 2.0 equiv) and 2-aminoethyl diphenylborinate (89.6 mg, 398 *µ*mol, 0.25 equiv) were added and the round bottom flask was protected from light. The reaction mixture was stirred overnight at 60 °C under Ar, quenched with a few drops of MeOH, diluted with DCM (10 mL) and filtered through a plug of Celite. The filtrate was concentrated under reduced pressure. The residue was purified by silica gel flash chromatography (PE/EtOAc 8:2 to 6:4) to give allyl 2,3,4,6-tetra-*O*-benzyl-*β*-D-glucopyranosyl-(1→3)-4-*O*-levulinoyl-*α*-L-rhamnopyranoside (978 mg, 74%) as a white amorphous solid: R_f 0.3 (tol/EtOAc 9:1); $[\alpha]_D^{20}$ = –13 (*c* 1.2, $CHCl_3$); ^1H NMR (400 MHz, $CDCl_3$) δ 7.33–7.16 (m, 20H, C*H*-Ar), 5.93–5.83 (m, 1H, H-2_{All}), 5.29 (ddd, *J* = 17.2, 3.6, 1.6 Hz, 1H, H-$3a_{All}$), 5.21 (ddd, *J* = 10.4, 3.3, 1.2 Hz, 1H, H-$3b_{All}$), 5.18 (t, *J* = 9.9 Hz, 1H, H-4B), 4.86 (d, $J_{1B,2B}$ = 1.5 Hz, 1H, H-1B), 4.85 (d, *J* = 10.9 Hz, 1H, C*H*HPh), 4.84 (d, *J* = 11.5 Hz, 1H, CH*H*Ph), 4.80 (d, *J* = 10.9 Hz, 1H, C*H*HPh), 4.75 (d, *J* = 10.9 Hz, 1H, CH*H*Ph), 4.63 (d, *J* = 11.5 Hz, 1H, C*H*HPh), 4.54 (d, $J_{1C,2C}$ = 7.7 Hz, 1H, H-1C), 4.52 (d, *J* = 10.9 Hz, 1H, CH*H*Ph), 4.49 (s, 2H, CH_2Ph), 4.16–4.13 (m, 2H, H-$1a_{All}$, H-2B), 4.05 (dd, *J* = 9.6, 3.4 Hz, 1H, H-3B), 3.97 (ddt, *J* = 12.9, 6.2, 1.2 Hz, 1H, H-$1b_{All}$), 3.85–3.78 (m, 1H, H-5B), 3.68 (dd, *J* = 10.4, 1.7 Hz, 1H, H-6aC), 3.62 (t, *J* = 9.1 Hz, 1H, H-3C), 3.57 (dd, *J* = 10.9, 5.2 Hz, 1H, H-6bC), 3.53 (t, *J* = 9.4 Hz, 1H, H-4C), 3.51–3.43 (m, 2H, H-2C, H-5C), 2.57–2.49 (m, 1H, CH_{2Lev}), 2.44–2.36 (m, 1H, CH_{2Lev}), 2.28–2.20 (m, 1H, CH_{2Lev}), 2.13–2.06 (m, 1H, CH_{2Lev}), 2.04 (s, 3H, CH_{3Lev}), 1.32 (d, *J* = 6.3 Hz, 3H, CH_{3Tal}); ^{13}C NMR (100 MHz, $CDCl_3$) δ 206.5 (*C*O), 172.1 (*C*O), 138.5, 138.3, 138.1, 138.0 (4 × C-Ar), 133.8 (C-2_{All}), 128.6–127.7 (12 × *C*H-Ar), 117.9 (C-3_{All}), 103.6 (C-1C), 98.5 (C-1B), 84.7 (C-3C), 81.8 (C-2C), 78.9 (C-3B), 77.7 (C-4C), 75.8 (*C*H_2Ph), 75.1 (*C*H_2Ph), 74.7 (C-5C), 74.5 (*C*H_2Ph), 73.7 (*C*H_2Ph), 72.7 (C-4B), 70.1 (C-2B), 69.0 (C-6C), 68.2 (C-1_{All}), 66.5 (C-5B), 37.7 (*C*H_{2Lev}), 29.8 (*C*H_{3Lev}), 27.8 (*C*H_{2Lev}), 17.5 (*C*H_{3Tal}); HRMS (ESI-TOF) *m/z* $[M + Na]^+$ calcd for $C_{48}H_{56}NaO_{12}$ 847.3664; found 847.3661.

The latter compound (2.9 g, 3.6 mmol, 1.0 equiv) was dissolved in anhydrous py (15 mL), then Ac_2O (15 mL) and DMAP (44 mg, 360 *µ*mol, 0.1 equiv) were added. The reaction mixture was stirred at rt for 3 h under Ar. The mixture was then concentrated under reduced pressure and the residue was purified by silica gel flash chromatography (PE/EtOAc 9:1 to 75:25) to give disaccharide **32** (3.0 g, 98%) as a colorless oil: R_f 0.5 (tol/EtOAc 8:2); $[\alpha]_D^{20}$ = +6.6 (*c* 1.4, $CHCl_3$); ^1H NMR (400 MHz, $CDCl_3$) δ 7.37–7.15 (m, 20H, C*H*-Ar), 5.89–5.79 (m, 1H, H-2_{All}), 5.29–5.24 (m, 2H, H-2B, H-$3a_{All}$), 5.18 (t, *J* = 9.9 Hz, 1H, H-4B), 5.17 (ddd, *J* = 10.4, 3.1, 1.3 Hz, 1H, H-$3b_{All}$), 4.86 (d, *J* = 10.9 Hz, 1H, C*H*HPh), 4.84 (d, $J_{1B,2B}$ = 1.6 Hz, 1H, H-1B), 4.81 (d, *J* = 11.6 Hz,

1H, CH*H*Ph), 4.77 (d, *J* = 10.8 Hz, 1H, C*H*HPh), 4.76 (d, *J* = 10.9 Hz, 1H, CH*H*Ph), 4.63 (d, *J* = 12.2 Hz, 1H, C*H*HPh), 4.61 (d, *J* = 11.6 Hz, 1H, CH*H*Ph), 4.55 (d, *J* = 12.2 Hz, 1H, C*H*HPh), 4.54 (d, *J* = 10.8 Hz, 1H, C*H*HPh), 4.50 (d, $J_{1C,2C}$ = 7.7 Hz, 1H, H-1C), 4.22 (dd, *J* = 9.9, 3.5 Hz, 1H, H-3B), 4.13 (ddt, *J* = 12.9, 5.3, 1.4 Hz, 1H, H-1a$_{All}$), 3.98 (ddt, *J* = 12.9, 6.1, 1.3 Hz, 1H, H-1b$_{All}$), 3.85–3.78 (m, 1H, H-5B), 3.73–3.66 (m, 2H, H-6aC, H-6bC), 3.61–3.56 (m, 2H, H-3C, H-4C), 3.46–3.42 (m, 1H, H-5C), 3.37 (td, *J* = 7.6, 2.4 Hz, 1H, H-2C), 2.55–2.42 (m, 2H, CH_{2Lev}), 2.27–2.17 (m, 2H, CH_{2Lev}), 2.10 (s, 3H, CH_{3Ac}), 2.04 (s, 3H, CH_{3Lev}), 1.21 (d, *J* = 6.3 Hz, 3H, CH_{3Tal}); ^{13}C NMR (100 MHz, $CDCl_3$) δ 206.6 (*C*O), 172.2, 170.5 (2 × *C*O), 138.8, 138.7, 138.6, 138.3 (4 × C-Ar), 133.6 (C-2$_{All}$), 128.5–127.5 (12 × *C*H-Ar), 117.9 (C-3$_{All}$), 104.5 (C-1C), 96.6 (C-1B), 84.7 (C-4C), 82.0 (C-2C), 77.8 (C-3C), 75.7 (*C*H_2Ph), 75.3 (C-5C), 75.1 (*C*H_2Ph), 74.6 (C-3B), 74.5 (*C*H_2Ph), 73.7 (*C*H_2Ph), 73.1 (C-4B), 72.7 (C-2B), 68.9 (C-6C), 68.5 (C-1$_{All}$), 66.8 (C-5B), 37.7 (*C*H_{2Lev}), 29.7 (*C*H_{3Lev}), 27.9 (*C*H_{2Lev}), 21.2 (*C*H_{3Ac}), 17.5 (*C*H_{3Tal}); HRMS (ESI-TOF) *m/z* $[M + Na]^+$ calcd for $C_{50}H_{58}NaO_{13}$ 889.3770; found 889.3750.

Allyl 2,3,4,6-Tetra-*O*-benzyl-β-D-glucopyranosyl-(1→3)-2-*O*-acetyl-α-L-rhamnopyranoside (33).

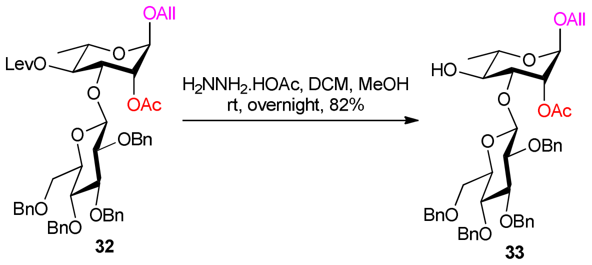

To a solution of disaccharide **32** (983 mg, 1.1 mmol, 1.0 equiv) in anhydrous DCM (5 mL) were added MeOH (11 mL) and hydrazine acetate (209 mg, 2.3 mmol, 2.0 equiv). After stirring at rt overnight, the reaction mixture was concentrated under reduced pressure and co-evaporated with toluene (3 ×). The residue was purified by silica gel flash chromatography (PE/EtOAc 9:1 to 75:25) to give alcohol **33** (715 mg, 82%) as a yellow oil: R_f 0.5 (tol/EtOAc 8:2); $[\alpha]_D^{20}$ = –72 (*c* 0.90, $CHCl_3$); ^{1}H NMR (400 MHz, $CDCl_3$) δ 7.35–7.15 (m, 20H, C*H*-Ar), 5.92–5.82 (m, 1H, H-2_{All}), 5.28 (ddd, *J* = 17.2, 3.8, 1.6 Hz, 1H, H-$3a_{All}$), 5.19–5.17 (m, 2H, H-2B, H-$3b_{All}$), 4.88 (d, *J* = 11.1 Hz, 1H, C*H*HPh), 4.84 (d, *J* = 10.5 Hz, 1H, CH*H*Ph), 4.81 (d, *J* = 11.1 Hz, 1H, C*H*HPh), 4.79 (d, $J_{1B,2B}$ = 1.8 Hz, 1H, H-1B), 4.78 (d, *J* = 10.5 Hz, 1H, CH*H*Ph), 4.65 (d, $J_{1C,2C}$ = 7.8 Hz, 1H, H-1C), 4.61 (d, *J* = 12.1 Hz, 1H, C*H*HPh), 4.59 (d, *J* = 11.8 Hz, 1H, CH*H*Ph), 4.58 (d, *J* = 11.8 Hz, 1H, C*H*HPh), 4.52 (d, *J* = 12.1 Hz, 1H, CH*H*Ph), 4.13 (ddt, *J* = 12.9, 5.3, 1.6 Hz, 1H, H-$1a_{All}$), 4.00–3.95 (m, 2H, H-$1b_{All}$, H-3B), 3.73–3.60 (m, 5H, H-3C, H-4C, H-5B, H-6aC, H-6bC), 3.53 (t, *J* = 9.6 Hz, 1H, H-4B), 3.48 (t, *J* = 8.5 Hz, 1H, H-2C), 3.42 (dt, *J* = 9.3, 3.2 Hz, 1H, H-5C), 2.97 (d, *J* = 2.4 Hz, 1H, O*H*), 2.07 (s, 3H, CH_{3Ac}), 1.25 (d, *J* = 6.3 Hz, 3H, CH_{3Tal}); ^{13}C NMR (100 MHz, $CDCl_3$) δ 170.4 (*C*O), 138.5, 138.3, 138.2, 137.9 (4 × C-Ar), 133.6 (C-2_{All}), 128.6–127.7 (12 × *C*H-Ar), 117.9 (C-3_{All}), 103.9 (C-1C), 96.7 (C-1B), 85.2 (C-3C), 82.3 (C-2C), 79.1 (C-3B), 77.9 (C-4C), 75.7 (*C*H_2Ph), 75.6 (*C*H_2Ph), 75.2 (*C*H_2Ph), 75.1 (C-5C), 73.6 (*C*H_2Ph), 72.5 (C-4B), 72.3 (C-2B), 68.7 (C-6C), 68.4 (C-1_{All}), 68.0 (C-5B), 21.2 (*C*H_{3Ac}), 17.7 (*C*H_{3Tal}); HRMS (ESI-TOF) *m/z* $[M + Na]^+$ calcd for $C_{45}H_{52}NaO_{11}$ 791.3402; found 791.3399.

Allyl 2,3,4,6-Tetra-*O*-benzyl-*β*-D-glucopyranosyl-(1→3)-2-*O*-acetyl-6-deoxy-*α*-L-talopyranoside (34).

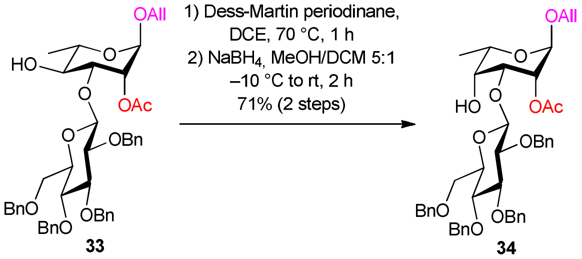

Dess-Martin periodinane (857 mg, 2.0 mmol, 2.2 equiv) was added to a solution of alcohol **33** (706 mg, 918 *μ*mol, 1.0 equiv) in anhydrous DCE (18 mL) and the combined mixture was heated at 70 °C under Ar for 1 h. The mixture was cooled to rt, then diluted with DCM (25 mL) and quenched with a 10% $Na_2S_2O_3$(aq) solution (25 mL). The organic phase was washed with brine (30 mL) and dried ($MgSO_4$). The solvents were concentrated under reduced pressure to give a ketone. To a cooled (–10 °C) solution of the ketone in MeOH/DCM (22 mL, 5:1 *v*/*v*), $NaBH_4$ (69 mg, 1.8 mmol, 2.0 equiv) was slowly added and the mixture was stirred from –10 °C to rt under Ar for 2 h. The reaction mixture was treated with a 10% HOAc(aq) solution (2 mL) and then concentrated under reduced pressure. The residue was purified by silica gel flash chromatography (PE/EtOAc 85:15 to 8:2) to give alcohol **34** (504 mg, 71%, two steps) as a colorless oil: R_f 0.4 (tol/EtOAc 8:2); $[\alpha]_D^{20}$ = –64 (*c* 1.0, $CHCl_3$); ^{1}H NMR (400 MHz, $CDCl_3$) δ 7.37–7.14 (m, 20H, C*H*-Ar), 5.93–5.83 (m, 1H, H-2_{All}), 5.29 (ddd, *J* = 17.2, 3.6, 1.6 Hz, 1H, H-$3a_{All}$), 5.21–5.19 (m, 2H, H-2B, H-$3b_{All}$), 4.98 (d, *J* = 11.0 Hz, 1H, C*H*HPh), 4.89 (d, *J* = 11.0 Hz, 1H, CH*H*Ph), 4.86 (d, $J_{1B,2B}$ = 1.4 Hz, 1H, H-1B), 4.80 (d, *J* = 10.8 Hz, 1H, C*H*HPh), 4.77 (d, *J* = 11.0 Hz, 1H, CH*H*Ph), 4.73 (d, *J* = 11.0 Hz, 1H, C*H*HPh), 4.64 (d, $J_{1C,2C}$ = 7.6 Hz, 1H, H-1C), 4.60 (d, *J* = 12.1 Hz, 1H, CH*H*Ph), 4.53 (d, *J* = 10.8 Hz, 1H, C*H*HPh), 4.51 (d, *J* = 12.1 Hz, 1H, CH*H*Ph), 4.21 (t, *J* = 3.8 Hz, 1H, H-3B), 4.15 (ddt, *J* = 12.9, 5.3, 1.4 Hz, 1H, H-$1a_{All}$), 4.01 (ddt, *J* = 12.9, 6.0, 1.6 Hz, 1H, H-$1b_{All}$), 3.89 (dd, *J* = 13.3, 6.6 Hz, 1H, H-5B), 3.76 (br s, 1H, H-4B), 3.69–3.68 (m, 2H, H-6aC, H-6bC), 3.66–3.60 (m, 2H, H-3C, H-4C), 3.45–3.40 (m, 1H, H-5C), 1.99 (s, 3H, CH_{3Ac}), 1.32 (d, *J* = 6.6 Hz, 3H, CH_{3Tal}); ^{13}C NMR (100 MHz, $CDCl_3$) δ 169.7 (*C*O), 138.7, 138.6, 138.3, 138.2 (4 × C-Ar), 133.6 (C-2_{All}), 128.5–127.7 (12 × *C*H-Ar), 117.9 (C-3_{All}), 101.2 (C-1C), 97.2 (C-1B), 84.6 (C-3C), 82.1 (C-2C), 77.6 (C-4C), 75.7 (*C*H_2Ph), 75.2 (*C*H_2Ph), 75.1 (C-5C), 74.7 (*C*H_2Ph), 73.5 (*C*H_2Ph), 71.9 (C-3B), 70.9 (C-2B), 69.1 (C-4B), 68.7 (C-6C), 68.6 (C-1_{All}), 66.8 (C-5B), 21.2 (*C*H_{3Ac}), 16.5 (*C*H_{3Tal}); HRMS (ESI-TOF) *m/z* $[M + Na]^+$ calcd for $C_{45}H_{52}NaO_{11}$ 791.3402; found 791.3418.

2,3,4,6-Tetra-*O*-benzyl-*β*-D-glucopyranosyl-(1→3)-2-*O*-acetyl-6-deoxy-*α*-L-talopyranosyl 2,2,2-Trichloroacetimidate (**35**).

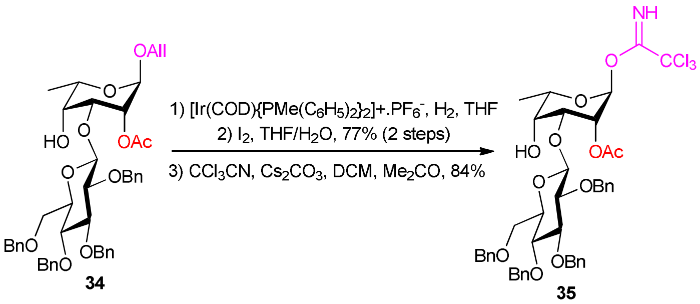

Allyl taloside **34** (458 mg, 600 μmol, 1.0 equiv) was reacted according to the general procedure for the synthesis of trichloroacetimidate donors (first part). Purification by silica gel flash chromatography (PE/EtOAc 9:1 to 5:5) gave a hemiacetal (292 mg, 77%, ratio α/β ~ 3:1) as a yellow amorphous solid: R_f 0.2 (tol/EtOAc 8:2); ^{1}H NMR (400 MHz, $CDCl_3$) δ 7.37–7.14 (m, 20H, C*H*-Ar), 5.24–5.19 (m, 2H, H-1B, H-2B), 4.98 (d, *J* = 11.1 Hz, 1H, C*H*HPh), 4.90 (d, *J* = 11.1 Hz, 1H, CH*H*Ph), 4.80 (d, *J* = 10.8 Hz, 1H, C*H*HPh), 4.77 (d, *J* = 11.7 Hz, 1H, CH*H*Ph), 4.74 (d, *J* = 11.7 Hz, 1H, C*H*HPh), 4.65 (d, $J_{1C,2C}$ = 7.7 Hz, 1H, H-1C), 4.60 (d, *J* = 12.1 Hz, 1H, CH*H*Ph), 4.52 (d, *J* = 10.8 Hz, 1H, C*H*HPh), 4.50 (d, *J* = 12.1 Hz, 1H, CH*H*Ph), 4.26 (t, *J* = 3.7 Hz, 1H, H-3B), 4.16–4.11 (m, 1H, H-5B), 3.78–3.75 (m, 1H, H-4B), 3.70–3.67 (m, 2H, H-6aC, H-6bC), 3.65–3.3.62 (m, 2H, H-3C, H-4C), 3.52 (dd, *J* = 7.5, 1.8 Hz, 1H, H-2C), 3.45–3.41 (m, 1H, H-5C), 2.92 (d, *J* = 3.8 Hz, 1H, O*H*), 2.59 (d, *J* = 9.1 Hz, 1H, O*H*), 2.00 (s, 3H, CH_{3Ac}), 1.32 (d, *J* = 6.6 Hz, 3H, CH_{3Tal}); ^{13}C NMR (100 MHz, $CDCl_3$) δ 169.8 (*C*O), 138.7, 138.6, 138.3, 138.2 (4 × C-Ar), 128.1–127.5 (12 × *C*H-Ar), 101.3 (C-1C), 92.7 (C-1B), 84.6 (C-3C), 82.1 (C-2C), 77.6 (C-4C), 75.7 (*C*H_2Ph), 75.2 (*C*H_2Ph), 75.0 (C-5C), 74.7 (*C*H_2Ph), 73.5 (*C*H_2Ph), 71.5 (C-3B), 71.2 (C-2B), 69.2 (C-4B), 68.8 (C-6C), 66.9 (C-5B), 21.2 (CH_{3Ac}), 16.6 (CH_{3Tal}); HRMS (ESI-TOF) *m/z* [M + Na]$^+$ calcd for $C_{42}H_{48}NaO_{11}$ 751.3089; found 751.3100; *m/z* [2M + Na]$^+$ calcd for $C_{84}H_{96}NaO_{22}$ 1479.6285; found 1479.6306. Then, the hemiacetal (282 mg, 390 μmol, 1.0 equiv) was reacted in the presence of Cs_2CO_3 (25 mg, 80 μmol, 0.2 equiv) and CCl_3CN (190 μL, 1.9 mmol, 5.0 equiv). Purification by silica gel flash chromatography (PE/EtOAc 8:2 to 6:4 + 1% Et_3N) gave imidate **35** (285 mg, 84%) as a yellow oil: R_f 0.4 (tol/EtOAc 8:2); $[\alpha]_D^{20}$ = +32 (*c* 0.82, $CHCl_3$); ^{1}H NMR (400 MHz, py-d_5) δ 7.65–7.29 (m, 20H, C*H*-Ar), 6.94 (s, 1H, H-1B), 5.93 (br s, 1H, H-2B), 5.42 (d, *J* = 11.1 Hz, 1H, C*H*HPh), 5.21 (d, $J_{1C,2C}$ = 7.7 Hz, 1H, H-1C), 5.13 (d, *J* = 11.1 Hz, 1H, CH*H*Ph), 5.00 (d, *J* = 10.9 Hz, 1H, C*H*HPh), 4.97–4.91 (m, 2H, 2 × CH_2Ph), 4.80 (t, *J* = 4.3 Hz, 1H, H-3B), 4.76 (d, *J* = 11.1 Hz, 1H, CH*H*Ph), 4.73 (d, *J* = 12.1 Hz, 1H, C*H*HPh), 4.65 (d, *J* = 12.1 Hz, 1H, CH*H*Ph), 4.48 (dd, *J* = 14.2, 6.3 Hz, 1H, H-5B), 3.33 (br s, 1H, H-4B), 3.96–3.88 (m, 4H, H-3C, H-4C, H-6aC, H-6bC), 3.80–3.75 (m, 2H, H-2C, H-5C), 1.96 (s, 3H, CH_{3Ac}), 1.53 (d, *J* = 6.6 Hz, 3H, CH_{3Tal}); ^{13}C NMR (100 MHz, py-d_5) δ 169.7 (*C*O), 139.6, 139.1, 138.3, 138.2 (4 × C-Ar), 128.5–127.7 (12 × *C*H-Ar), 101.9 (C-1C), 96.3 (C-1B), 84.8 (C-4C), 82.6 (C-2C), 78.2 (C-3C), 75.6 (*C*H_2Ph), 75.5 (C-5C), 74.9 (*C*H_2Ph), 74.6 (*C*H_2Ph), 73.7 (*C*H_2Ph), 72.9 (C-3B), 70.7 (C-5B), 69.5 (C-6C), 69.2 (C-2B), 68.4 (C-4B), 20.9 (CH_{3Ac}), 16.9 (CH_{3Tal}).

2,3,4,6-Tetra-*O*-benzyl-*β*-D-glucopyranosyl-(1→3)-6-deoxy-1,2,4-*O*-orthoacetyl-*β*-L-talopyranose (36).

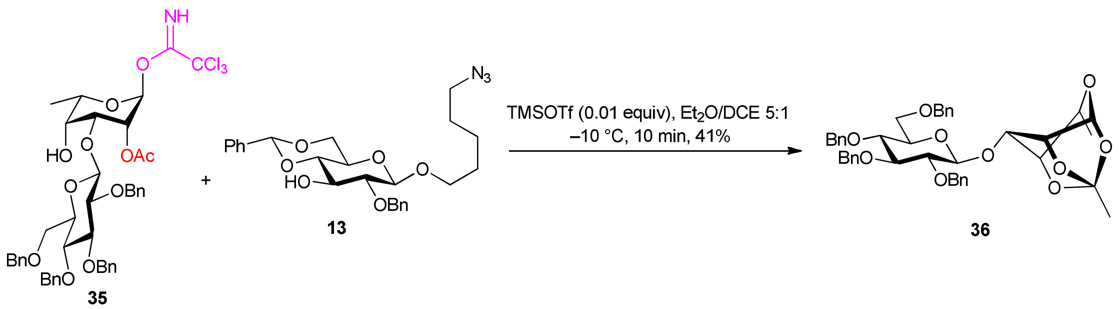

A mixture of acceptor **13** (223 mg, 476 *µ*mol, 1.5 equiv) and donor **35** (277 mg, 317 *µ*mol, 1.0 equiv) in anhydrous Et_2O/DCE (8 mL, 5:1 *v/v*) was cooled to –10 °C and TMSOTf (0.6 *µ*L, 3 *µ*mol, 0.01 equiv) was added keeping rigorous anhydrous conditions. The mixture was stirred for 10 min at –10 °C under Ar and then quenched with a few drops of Et_3N. The suspension was filtered over Celite, rinsed with DCM and the filtrate was concentrated under reduced pressure. The residue was purified by combi-flash chromatography (PE/acetone 92:8) to give tricyclic orthoester **36** (92 mg, 41%) as a white amorphous powder, which was recrystallized in EtOAc: R_f 0.6 (tol/EtOAc 8:2); $[\alpha]_D^{20}$ = +17 (*c* 1.0, $CHCl_3$); ^{1}H NMR (400 MHz, $CDCl_3$) δ 7.41–7.25 (m, 18H, C*H*-Ar), 7.18–7.15 (m, 2H, C*H*-Ar), 5.68 (d, $J_{1,2}$ = 5.1 Hz, 1H, H-1B), 5.08 (d, *J* = 10.8 Hz, 1H, C*H*HPh), 4.95 (d, *J* = 11.0 Hz, 1H, C*H*HPh), 4.82 (d, *J* = 11.1 Hz, 1H, C*H*HPh), 4.79 (d, *J* = 11.1 Hz, 1H, C*H*HPh), 4.78 (d, *J* = 10.9 Hz, 1H, C*H*HPh), 4.74–4.70 (m, 1H, H-2B), 4.58–4.49 (m, 4H, H-1C, CH_2Ph, C*H*HPh), 4.06–4.00 (m, 2H, H-4B, H-5B), 3.75–3.69 (m, 2H, H-6aC, H-3B), 3.67–3.59 (m, 3H, H-2C, H-3C, H-6bC), 3.56 (t, $J_{3,4} \approx J_{4,5} \approx$ 9.6 Hz, 1H, H-4C), 3.47 (ddd, $J_{4,5}$ = 9.6 Hz, $J_{5,6a}$ = 5.4 Hz, $J_{5,6b}$ = 1.8 Hz, 1H, H-5C), 1.69 (s, 3H, C$H_{3orthoester}$), 1.39 (d, $J_{5,6}$ = 6.4 Hz, 3H, H-6B); ^{13}C NMR (100 MHz, $CDCl_3$) δ 138.7, 138.6, 138.2, 138.1 (4 × C-Ar), 128.6–127.8 (*C*H-Ar), 118.2 ($C_{orthoester}$), 103.1 (C-1C), 99.1 (C-1B), 84.7 (C-2C), 81.9 (C-3C), 77.8 (C-4C), 76.7 (C-5B), 75.8 (*C*H_2Ph), 75.5 (C-2B), 75.2 (*C*H_2Ph), 75.1 (C-5C), 75.0 (*C*H_2Ph), 73.6 (*C*H_2Ph), 73.5 (C-4B), 70.8 (C-3B), 69.3 (C-6C), 20.9 (*C*$H_{3orthoester}$), 19.4 (C-6B); HRMS (ESI-TOF) *m/z* [M + Na]$^+$ calcd for $C_{42}H_{46}NaO_{10}$ 733.2983; found 733.2995.

2,3,4,6-Tetra-*O*-benzyl-*β*-D-glucopyranosyl-(1→3)-2-*O*-acetyl-4-*O*-levulinoyl-*α*-L-rhamnopyranosyl 2,2,2-Trichloroacetimidate (37).

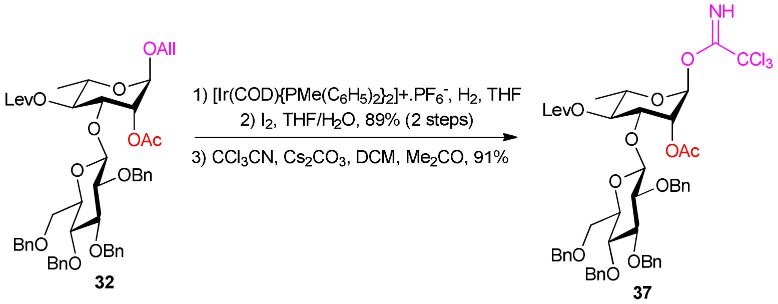

Taloside **32** (1.0 g, 1.2 mmol, 1.0 equiv) was reacted according to the general procedure for the synthesis of trichloroacetimidate donors (first part). Purification by silica gel flash chromatography (PE/EtOAc 85:15 to 65:35) gave a hemiacetal (881 mg, 89%, ratio *α*/*β* ~ 10:1) as a white foam: R_f 0.2 (tol/EtOAc 8:2); ^{1}H NMR (400 MHz, $CDCl_3$) *δ* 7.38–7.15 (m, 20H, C*H*-Ar), 5.30 (dd, *J* = 3.5, 1.8 Hz, 1H, H-2B), 5.19 (d, $J_{1B,2B}$ = 1.7 Hz, 1H, H-1B), 5.18 (t, *J* = 10.0 Hz, 1H, H-4B), 4.87 (d, *J* = 10.9 Hz, 1H, C*H*HPh), 4.81 (d, *J* = 11.7 Hz, 1H, CH*H*Ph), 4.77 (d, *J* = 10.9 Hz, 1H, C*H*HPh), 4.76 (d, *J* = 10.9 Hz, 1H, CH*H*Ph), 4.62 (d, *J* = 12.1 Hz, 1H, C*H*HPh), 4.61 (d, *J* = 11.7 Hz, 1H, CH*H*Ph), 4.55 (d, *J* = 12.1 Hz, 1H, C*H*HPh), 4.54 (d, *J* = 10.9 Hz, 1H, CH*H*Ph), 4.51 (d, $J_{1C,2C}$ = 7.8 Hz, 1H, H-1C), 4.27 (dd, *J* = 10.0, 3.4 Hz, 1H, H-3B), 4.07–4.00 (m, 1H, H-5B), 3.73 (dd, *J* = 10.9, 1.9 Hz, 1H, H-6aC), 3.66 (dd, *J* = 10.6, 4.7 Hz, 1H, H-6bC), 3.60 (t, *J* = 9.0 Hz, 1H, H-3C), 3.56 (t, *J* = 8.8 Hz, 1H, H-4C), 3.48–3.43 (m, 1H, H-5C), 3.37 (dd, *J* = 8.4, 7.9 Hz, 1H, H-2C), 3.01 (d, *J* = 3.9 Hz, 1H, O*H*), 2.53–2.43 (m, 2H, CH_{2Lev}), 2.26–2.23 (m, 2H, CH_{2Lev}), 2.19 (s, 3H, CH_{3Ac}), 2.04 (s, 3H, CH_{3Lev}), 1.20 (d, *J* = 6.3 Hz, 3H, CH_{3Tal}); ^{13}C NMR (100 MHz, $CDCl_3$) *δ* 206.7 (*C*O), 172.2, 170.5 (2 × *C*O), 138.8, 138.7, 138.5, 138.2 (4 × C-Ar), 128.5–127.5 (12 × *C*H-Ar), 104.6 (C-1C), 92.1 (C-1B), 84.7 (C-3C), 81.9 (C-2C), 77.9 (C-4C), 75.7 (*C*H$_2$Ph), 75.1 (*C*H$_2$Ph), 75.0 (C-5C), 74.5 (*C*H$_2$Ph), 74.2 (C-3B), 73.6 (*C*H$_2$Ph), 73.1 (C-2B, C-4B), 69.1 (C-6C), 66.8 (C-5B), 37.7 (*C*H_{2Lev}), 29.8 (*C*H_{3Lev}), 27.9 (*C*H_{2Lev}), 21.2 (*C*H_{3Ac}), 17.6 (*C*H_{3Tal}); HRMS (ESI-TOF) *m/z* [M + Na]$^+$ calcd for $C_{47}H_{54}NaO_{13}$ 849.3457; found 849.3489. Then, the hemiacetal (861 mg, 1.0 mmol, 1.0 equiv) was reacted in the presence of Cs_2CO_3 (68 mg, 210 *μ*mol, 0.2 equiv) and CCl_3CN (630 *μ*L, 6.5 mmol, 6.0 equiv). Purification by silica gel flash chromatography (PE/EtOAc 8:2 to 6:4 + 1% Et_3N) gave imidate **37** (921 mg, 91%) as a white foam: R_f 0.4 (tol/EtOAc 8:2); $[\alpha]_D^{20}$ = +44 (*c* 0.82, $CHCl_3$/THF 1:1); ^{1}H NMR (400 MHz, $CDCl_3$) *δ* 7.33–7.14 (m, 20H, C*H*-Ar), 6.26 (d, $J_{1B,2B}$ = 1.9 Hz, 1H, H-1B), 5.46 (dd, *J* = 3.5, 1.9 Hz, 1H, H-2B), 5.28 (t, *J* = 10.2 Hz, 1H, H-4B), 4.88 (d, *J* = 11.0 Hz, 1H, C*H*HPh), 4.81 (d, *J* = 11.0 Hz, 1H, CH*H*Ph), 4.78 (d, *J* = 10.8 Hz, 1H, C*H*HPh), 4.77 (d, *J* = 10.8 Hz, 1H, CH*H*Ph), 4.62 (d, *J* = 12.3 Hz, 1H, C*H*HPh), 4.59 (d, *J* = 11.9 Hz, 1H, CH*H*Ph), 4.56 (d, *J* = 11.9 Hz, 1H, C*H*HPh), 4.52 (d, $J_{1C,2C}$ = 7.8 Hz, 1H, H-1C), 4.51 (d, *J* = 12.3 Hz, 1H, C*H*HPh), 4.25 (dd, *J* = 10.1, 3.5 Hz, 1H, H-3B), 4.03–3.95 (m, 1H, H-5B), 3.72 (dd, *J* = 11.0, 3.9 Hz, 1H, H-6aC), 3.66 (t, *J* = 9.4 Hz, 1H, H-3C), 3.62–3.57 (m, 2H, H-4C, H-6bC), 3.43–3.37 (m, 2H, H-2C, H-5C), 2.55–2.46 (m, 2H, CH_{2Lev}), 2.26–2.18 (m, 2H, CH_{2Lev}), 2.16 (s, 3H, CH_{3Ac}), 2.04 (s, 3H, CH_{3Lev}), 1.25 (d, *J* = 6.3 Hz, 3H, CH_{3Tal}); ^{13}C NMR (100 MHz, $CDCl_3$) *δ* 206.6 (*C*O), 172.1, 170.0 (2 × *C*O), 159.9 (C-Ar), 138.7, 138.4, 138.3 (3 × C-Ar), 128.5–127.5 (12 × *C*H-Ar), 104.7 (C-1C), 94.6 (C-1B), 84.7 (C-4C), 81.9 (C-2C), 77.7 (C-3C), 75.7 (*C*H$_2$Ph), 75.2 (C-5C), 75.1 (*C*H$_2$Ph), 74.6 (*C*H$_2$Ph), 74.2 (C-3B), 73.6 (*C*H$_2$Ph), 72.3 (C-4B), 71.1 (C-2B), 69.7 (C-5B), 68.6 (C-6C), 37.7 (*C*H_{2Lev}), 29.8 (*C*H_{3Lev}), 27.8 (*C*H_{2Lev}), 21.1 (*C*H_{3Ac}), 17.6 (*C*H_{3Tal}); HRMS (ESI-TOF) *m/z* [M + Na]$^+$ calcd for $C_{49}H_{54}Cl_3NNaO_{13}$ 992.2553; found 992.2552.

(5-Azido-1-pentyl) 2,3,4,6-Tetra-*O*-benzyl-*β*-D-glucopyranosyl-(1→3)-2-*O*-acetyl-*α*-L-rhamnopyranosyl-(1→3)-2-*O*-benzyl-4,6-*O*-benzylidene-*β*-D-glucopyranoside (38).

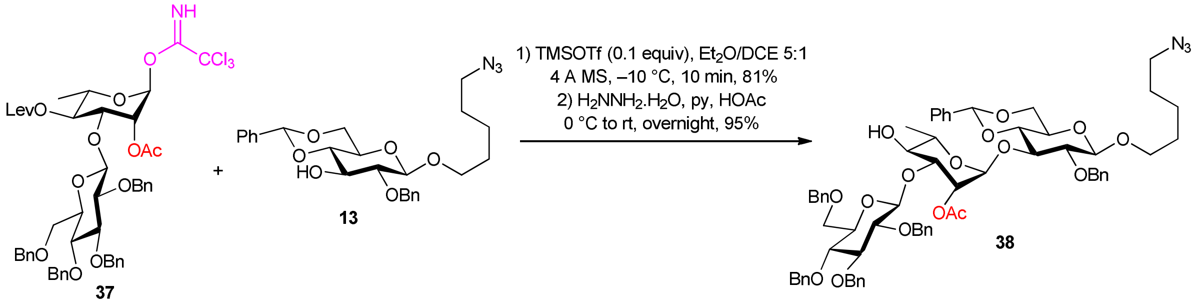

Acceptor **13** (350 mg, 745 μmol, 1.0 equiv) and donor **37** (869 mg, 895 μmol, 1.2 equiv) were dried for 4 h under high vacuum and then dissolved in anhydrous Et_2O/DCE (18 mL, 5:1 *v*/*v*). Freshly activated 4 Å powdered molecular sieves (1.4 g) were added and the suspension was stirred for 40 min at rt under Ar. Then, the reaction mixture was cooled to −10 °C and TMSOTf (14 μL, 75 μmol, 0.1 equiv) was injected. The mixture was stirred at –10 °C for 10 min under Ar. The reaction was then quenched with Et_3N (100 μL), filtered over Celite and rinsed with DCM. The filtrate was concentrated under reduced pressure and purified by combi-flash chromatography (PE/EtOAc 7:3) to give (5-azido-1-pentyl) 2,3,4,6-tetra-*O*-benzyl-*β*-D-glucopyranosyl-(1→3)-2-*O*-acetyl-4-*O*-levulinoyl-*α*-L-rhamnopyranosyl-(1→3)-2-*O*-benzyl-4,6-*O*-benzylidene-*β*-D-glucopyranoside (768 mg, 81%) as a white foam: R_f 0.6 (tol/EtOAc 8:2); $[\alpha]_D^{20}$ = –17 (*c* 1.2, $CHCl_3$); ^{1}H NMR (400 MHz, $CDCl_3$) δ 7.48–7.15 (m, 30H, C*H*-Ar), 5.48 (s, 1H, H-7A), 5.43 (dd, *J* = 3.6, 1.5 Hz, 1H, H-2B), 5.24 (d, *J* = 1.3 Hz, 1H, H-1B), 5.08 (t, *J* = 10.2 Hz, 1H, H-4B), 4.85 (d, *J* = 11.0 Hz, 1H, C*H*HPh), 4.78 (d, *J* = 11.7 Hz, 1H, CH*H*Ph), 4.77 (d, *J* = 10.8 Hz, 1H, C*H*HPh), 4.76 (d, *J* = 10.8 Hz, 1H, CH*H*Ph), 4.75 (d, *J* = 11.0 Hz, 1H, C*H*HPh), 4.73 (d, *J* = 10.8 Hz, 1H, CH*H*Ph), 4.63 (d, *J* = 12.1 Hz, 1H, C*H*HPh), 4.57 (d, *J* = 12.1 Hz, 1H, CH*H*Ph), 4.56 (d, *J* = 11.7 Hz, 1H, C*H*HPh), 4.53 (d, *J* = 10.9 Hz, 1H, CH*H*Ph), 4.49 (d, $J_{1C,2C}$ = 7.6 Hz, 1H, H-1C), 4.45 (d, $J_{1A,2A}$ = 7.7 Hz, 1H, H-1A), 4.32 (dd, *J* = 10.5, 4.8 Hz, 1H, H-6aA), 4.18 (dd, *J* = 10.0, 3.6 Hz, 1H, H-3B), 4.15–4.09 (m, 1H, H-5B), 3.92 (t, *J* = 9.5 Hz, 1H, H-3A), 3.90–3.86 (m, 1H, H-$1a_{linker}$), 3.77–3.68 (m, 3H, H-6bA, H-6aC, H-6bC), 3.59–3.49 (m, 3H, H-$1b_{linker}$, H-3C, H-4A), 3.44 (t, *J* = 9.5 Hz, 1H, H-4C), 3.40–3.32 (m, 4H, H-2A, H-2C, H-5A, H-5C), 3.20 (t, *J* = 7.2 Hz, 2H, H-5_{linker}), 2.50–2.33 (m, 2H, CH_{2Lev}), 2.25–2.16 (m, 2H, CH_{2Lev}), 2.04 (s, 3H, CH_{3Ac}), 2.02 (s, 3H, CH_{3Lev}), 1.66–1.55 (m, 4H, H-2_{linker}, H-4_{linker}), 1.46–1.37 (m, 2H, H-3_{linker}), 0.82 (d, *J* = 6.3 Hz, 3H, CH_{3Tal}); ^{13}C NMR (100 MHz, $CDCl_3$) δ 206.6 (*C*O), 171.9, 169.7 (2 × *C*O), 138.7, 138.6, 138.4, 138.2, 138.1, 137.1 (6 × C-Ar), 129.2–126.2 (18 × *C*H-Ar), 104.1 (C-1A), 103.9 (C-1C), 101.6 (C-7A), 97.7 (C-1B), 84.6 (C-3C), 82.9 (C-2A), 82.1 (C-2C), 79.0 (C-4C*), 77.8 (C-4A*), 75.9 (C-3A), 75.5 (*C*H_2Ph), 75.3 (C-5C), 74.9 (*C*H_2Ph), 74.8 (*C*H_2Ph), 74.4 (*C*H_2Ph), 74.2 (C-3B), 73.7 (*C*H_2Ph), 73.1 (C-4B), 71.9 (C-2B), 70.0 (C-1_{linker}), 69.2 (C-6A*), 68.8 (C-6C*), 66.3 (C-5A*), 66.2 (C-5B*), 51.3 (C-5_{linker}), 37.5 (*C*H_{2Lev}), 29.7 (*C*H_{3Lev}), 29.3 (C-2_{linker}), 28.6 (C-4_{linker}), 27.7 (*C*H_{2Lev}), 23.3 (C-3_{linker}), 20.9 (*C*H_{3Ac}), 16.8 (*C*H_{3Tal}); HRMS (ESI-TOF) *m/z* $[M + Na]^+$ calcd for $C_{72}H_{83}N_3NaO_{18}$ 1300.5564; found 1300.5586.

Acetic acid (2.6 mL) and hydrazine monohydrate (151 μL, 3.1 mmol, 5.0 equiv) were slowly added to a stirred solution of the latter compound (796 mg, 620 μmol, 1.0 equiv) in anhydrous py (4 mL) at 0 °C under Ar. Then, the reaction mixture was stirred from 0 °C to rt overnight. After this time,

solvents were concentrated and co-evaporated with toluene (3 ×). The residue was purified by silica gel flash chromatography (PE/EtOAc 9:1 to 8:2) to give alcohol **38** (696 mg, 95%) as a colorless oil: R_f 0.6 (tol/EtOAc 8:2); $[\alpha]_D^{20}$ = –20 (*c* 0.79, $CHCl_3$); ^1H NMR (400 MHz, $CDCl_3$) δ 7.47–7.15 (m, 30H, C*H*-Ar), 5.49 (s, 1H, H-7A), 5.34 (dd, *J* = 3.7, 1.5 Hz, 1H, H-2B), 5.21 (d, *J* = 1.4 Hz, 1H, H-1B), 4.87 (d, *J* = 11.1 Hz, 1H, C*H*HPh), 4.83–4.76 (m, 6H, 3 × CH_2Ph), 4.67 (d, $J_{1C,2C}$ = 7.8 Hz, 1H, H-1C), 4.60 (d, *J* = 12.1 Hz, 1H, CH*H*Ph), 4.54 (d, *J* = 10.8 Hz, 1H, C*H*HPh), 4.52 (d, *J* = 12.1 Hz, 1H, CH*H*Ph), 4.46 (d, $J_{1A,2A}$ = 7.8 Hz, 1H, H-1A), 4.32 (dd, *J* = 10.4, 4.9 Hz, 1H, H-6aA), 4.04–4.00 (m, 1H, H-5B), 3.98 (dd, *J* = 9.6, 3.5 Hz, 1H, H-3B), 3.92 (t, *J* = 9.5 Hz, 1H, H-3A), 3.90–3.86 (m, 1H, H-1a_{linker}), 3.74 (t, *J* = 10.7 Hz, 1H, H-6bA), 3.70–3.67 (m, 2H, H-6aC, H-6bC), 3.63–3.57 (m, 2H, H-3C, H-4C), 3.54 (dd, *J* = 6.9, 2.9 Hz, 1H, H-4A), 3.51–3.40 (m, 5H, H-1b_{linker}, H-2A, H-2C, H-4B, H-5C), 3.37 (dd, *J* = 9.8, 4.8 Hz, 1H, H-5A), 3.19 (t, *J* = 7.3 Hz, 2H, H-5_{linker}), 2.75 (s, 1H, O*H*), 2.02 (s, 3H, CH_{3Ac}), 1.66–1.55 (m, 4H, H-2_{linker}, H-4_{linker}), 1.46–1.39 (m, 2H, H-3_{linker}), 0.89 (d, *J* = 6.2 Hz, 3H, CH_{3Tal}); ^{13}C NMR (100 MHz, $CDCl_3$) δ 169.9 (*C*O), 138.6, 138.4, 138.3, 138.2, 137.9, 137.2 (6 × C-Ar), 129.1–126.3 (18 × *C*H-Ar), 104.2 (C-1A), 103.5 (C-1C), 101.6 (C-7A), 98.1 (C-1B), 85.1 (C-3C), 83.0 (C-2A), 82.2 (C-2C), 79.2 (C-4A), 78.8 (C-3B), 77.9 (C-4C), 76.4 (C-3A), 75.7 (*C*H_2Ph), 75.5 (*C*H_2Ph), 75.2 (C-5C), 75.1 (*C*H_2Ph), 74.9 (*C*H_2Ph), 73.6 (*C*H_2Ph), 72.3 (C-4B), 71.9 (C-2B), 70.1 (C-1_{linker}), 68.9 (C-6A*), 68.8 (C-6C*), 67.9 (C-5B), 66.4 (C-5A), 51.4 (C-5_{linker}), 29.4 (C-2_{linker}), 28.7 (C-4_{linker}), 23.4 (C-3_{linker}), 21.1 (*C*H_{3Ac}), 17.2 (*C*H_{3Tal}); HRMS (ESI-TOF) *m/z* $[M + Na]^+$ calcd for $C_{67}H_{77}N_3NaO_{16}$ 1202.5196; found 1202.5220.

(5-Azido-1-pentyl) 2,3,4,6-Tetra-*O*-benzyl-*β*-D-glucopyranosyl-(1→3)-2-*O*-acetyl-6-deoxy-*α*-L-talopyranosyl-(1→3)-2-*O*-benzyl-4,6-*O*-benzylidene-*β*-D-glucopyranoside (39).

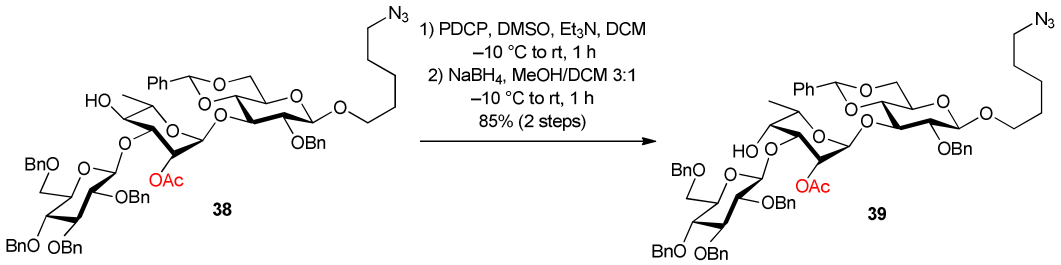

To a solution of DMSO (20 μL, 216 μmol, 10 equiv) in anhydrous DCM (0.4 mL) at –10 °C under Ar were sequentially added PDCP (20 μL, 130 μmol, 6.0 equiv) and Et_3N (40 μL, 216 μmol, 10 equiv). Then, a solution of trisaccharide **38** (26 mg, 22 μmol, 1.0 equiv) in DCM (0.1 mL) was added dropwise. The reaction mixture was stirred at –10 °C for 10 min, then allowed to slowly warm up to rt. After 1 h, DCM (6 mL) was added. The organic phase was washed with water (3 × 3 mL). The aqueous layer was back extracted with DCM (6 mL). The combined organic phases were washed with brine (5 mL). Then, the solvents of the dried solution ($MgSO_4$) were concentrated under reduced pressure to give a ketone. To a cooled (–10 °C) solution of the ketone in MeOH/DCM (0.4 mL, 3:1 *v/v*), $NaBH_4$ (3.2 mg, 86 μmol, 4.0 equiv) was slowly added. The mixture was stirred from –10 °C to rt under Ar for 1 h. Then, the reaction mixture was diluted with DCM (6 mL) and the organic phase was washed with water (3 × 4 mL). The aqueous layer was extracted with DCM (2 × 3 mL). The combined organic phases were washed with brine, dried ($MgSO_4$) and then concentrated under reduced pressure. The residue was purified by silica gel flash chromatography (PE/EtOAc 8:2 to 75:25) to give alcohol **39** (22 mg, 85%, two steps) as a colorless solid: R_f 0.5 (tol/EtOAc 8:2); $[\alpha]_D^{20}$ = –45 (*c* 0.34, $CHCl_3$); ^{1}H NMR (400 MHz, $CDCl_3$) δ 7.41–7.13 (m, 30H, C*H*-Ar), 5.37 (s, 1H, H-7A), 5.32 (dd, *J* = 3.7, 1.2 Hz, 1H, H-2B), 5.30 (d, *J* = 1.2 Hz, 1H, H-1B), 4.92 (d, *J* = 11.0 Hz, 1H, C*H*HPh), 4.88 (d, *J* = 11.0 Hz, 1H, CH*H*Ph), 4.85 (d, *J* = 10.2 Hz, 1H, C*H*HPh), 4.79 (d, *J* = 10.8 Hz, 1H, CH*H*Ph), 4.77 (d, *J* = 10.2 Hz, 1H, C*H*HPh), 4.75 (d, *J* = 10.8 Hz, 1H, CH*H*Ph), 4.69 (d, *J* = 11.1 Hz, 1H, C*H*HPh), 4.63 (d, $J_{1C,2C}$ = 7.6 Hz, 1H, H-1C), 4.56 (d, *J* = 12.2 Hz, 1H, CH*H*Ph), 4.54 (d, *J* = 11.1 Hz, 1H, C*H*HPh), 4.49 (d, $J_{1A,2A}$ = 7.8 Hz, 1H, H-1A), 4.46 (d, *J* = 12.2 Hz, 1H, CH*H*Ph), 4.31 (dd, *J* = 10.5, 4.8 Hz, 1H, H-6aA), 4.19–4.14 (m, 2H, H-3B, H-5B), 3.97 (t, *J* = 9.4 Hz, 1H, H-3A), 3.91 (dt, *J* = 9.5, 6.8 Hz, 1H, H-$1a_{linker}$), 3.72 (t, *J* = 10.4 Hz, 1H, H-6bA), 3.67 (d, *J* = 2.9 Hz, 2H, H-6aC, H-6bC), 3.63–3.57 (m, 3H, H-3C, H-4B, H-4C), 3.53 (dt, *J* = 9.5, 6.9 Hz, 1H, H-$1b_{linker}$), 3.49–3.42 (m, 4H, H-2A, H-2C, H-4A, H-5C), 3.37 (dd, *J* = 9.6, 4.7 Hz, 1H, H-5A), 3.19 (t, *J* = 7.3 Hz, 2H, H-5_{linker}), 1.88 (s, 3H, CH_{3Ac}), 1.67–1.54 (m, 4H, H-2_{linker}, H-4_{linker}), 1.47–1.37 (m, 2H, H-3_{linker}), 0.96 (d, *J* = 6.4 Hz, 3H, CH_{3Tal}); ^{13}C NMR (100 MHz, $CDCl_3$) δ 169.2 (*C*O), 138.7, 138.6, 138.4, 138.3, 138.2, 137.3 (6 × C-Ar), 129.1–126.3 (18 × *C*H-Ar), 104.2 (C-1A), 101.7 (C-7A), 100.6 (C-1C), 98.3 (C-1B), 84.6 (C-3C), 83.1 (C-2A), 82.1 (C-2C), 79.2 (C-4A), 77.6 (C-4C), 75.8 (C-3A), 75.7 (*C*H_2Ph), 75.2 (C-5C), 75.1 (*C*H_2Ph), 74.8 (*C*H_2Ph), 74.7 (*C*H_2Ph), 73.4 (*C*H_2Ph), 71.6 (C-3B), 70.2 (C-1_{linker}), 70.1 (C-2B), 68.9 (C-4B, C-6A*), 68.8 (C-6C*), 66.6 (C-5B), 66.4 (C-5A), 51.4 (C-5_{linker}), 29.4 (C-2_{linker}), 28.7 (C-4_{linker}), 23.4 (C-3_{linker}), 21.0 (*C*H_{3Ac}), 16.1 (*C*H_{3Tal}); HRMS (ESI-TOF) *m/z* $[M + Na]^+$ calcd for $C_{67}H_{77}N_3NaO_{16}$ 1202.5196; found 1202.5225.

(5-Azido-1-pentyl) 2,3,4,6-Tetra-*O*-benzyl-*β*-D-glucopyranosyl-(1→3)-6-deoxy-*α*-L-talopyranosyl-(1→3)-2-*O*-benzyl-4,6-*O*-benzylidene-*β*-D-glucopyranoside (40).

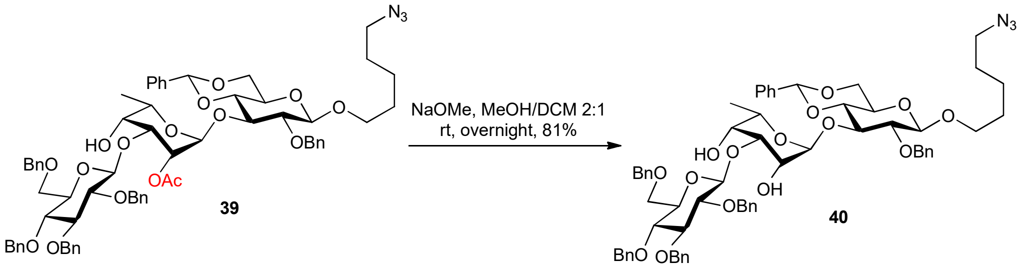

To a solution of compound **39** (29 mg, 25 *µ*mol, 1.0 equiv) in anhydrous MeOH/DCM (0.8 mL, 2:1 *v/v*) was added NaOMe (25% in MeOH, 2.4 *µ*L, 10 *µ*mol, 0.4 equiv). The reaction mixture was stirred overnight at rt under Ar. Dowex H^+ was added until neutralization, then the solution was filtered off and the solvents were concentrated under reduced pressure. The residue was purified by silica gel flash chromatography (PE/EtOAc 85:15 to 7:3) to give diol **40** (23 mg, 81%) as a colorless solid: R_f 0.6 (tol/EtOAc 8:2); $[\alpha]_D^{20}$ = –34 (*c* 0.20, $CHCl_3$); ^1H NMR (400 MHz, $CDCl_3$) δ 7.45–7.13 (m, 30H, C*H*-Ar), 5.43 (s, 1H, H-7A), 5.30 (br s, 1H, H-1B), 4.93 (d, *J* = 11.1 Hz, 1H, C*H*HPh), 4.91 (d, *J* = 10.9 Hz, 1H, CH*H*Ph), 4.85–4.78 (m, 4H, 2 × CH_2Ph), 4.65 (d, *J* = 10.9 Hz, 1H, C*H*HPh), 4.59 (d, $J_{1C,2C}$ = 7.7 Hz, 1H, H-1C), 4.49 (d, *J* = 10.8 Hz, 1H, CH*H*Ph), 4.48 (d, $J_{1A,2A}$ = 7.8 Hz, 1H, H-1A), 4.45 (d, *J* = 12.2 Hz, 1H, C*H*HPh), 4.40 (d, *J* = 12.2 Hz, 1H, CH*H*Ph), 4.32 (dd, *J* = 10.7, 4.6 Hz, 1H, H-6aA), 4.09 (dd, *J* = 13.9, 6.2 Hz, H-5B), 3.96 (t, *J* = 9.4 Hz, 1H, H-3A), 3.94–3.89 (m, 2H, H-2B, H-$1a_{linker}$), 3.83 (t, *J* = 3.6 Hz, 1H, H-3B), 3.74 (t, *J* = 10.4 Hz, 1H, H-6bA), 3.64 (t, *J* = 9.3 Hz, 1H, H-3C), 3.60–3.37 (m, 10H, H-$1b_{linker}$, H-2A, H-2C, H-4A, H-4B, H-4C, H-5A, H-5C, H-6aC, H-6bC), 3.22 (t, *J* = 7.3 Hz, 2H, H-5_{linker}), 3.14 (d, *J* = 8.5 Hz, O*H*), 1.69–1.57 (m, 4H, H-2_{linker}, H-4_{linker}), 1.50–1.43 (m, 2H, H-3_{linker}), 0.89 (d, *J* = 6.5 Hz, 3H, CH_{3Tal}); ^{13}C NMR (100 MHz, $CDCl_3$) δ 138.5, 138.4, 138.0, 137.9, 137.8, 137.3 (6 × C-Ar), 129.1–126.3 (18 × *C*H-Ar), 104.2 (C-1A), 101.9 (C-7A), 101.4 (C-1C), 100.8 (C-1B), 84.7 (C-3C), 82.7 (C-2A), 81.9 (C-2C), 79.4 (C-4A), 77.8 (C-4C), 76.3 (C-3A), 75.8 (*C*H_2Ph), 75.3 (C-3B), 75.2 (*C*H_2Ph), 75.1 (*C*H_2Ph), 75.0 (*C*H_2Ph), 74.9 (C-5C), 73.6 (*C*H_2Ph), 70.7 (C-4B), 70.2 (C-1_{linker}), 69.7 (C-2B), 69.0 (C-6A*), 68.9 (C-6C*), 66.7 (C-5B), 66.4 (C-5A), 51.4 (C-5_{linker}), 29.4 (C-2_{linker}), 28.7 (C-4_{linker}), 23.4 (C-3_{linker}), 16.1 (*C*H_{3Tal}); HRMS (ESI-TOF) *m/z* $[M + Na]^+$ calcd for $C_{65}H_{75}N_3NaO_{15}$ 1160.5090; found 1160.5083.

(5-Azido-1-pentyl) 2,3,4,6-Tetra-*O*-benzyl-*β*-D-glucopyranosyl-(1→3)-2,4-di-*O*-acetyl-6-deoxy-*α*-L-talopyranosyl-(1→3)-2-*O*-benzyl-4,6-*O*-benzylidene-*β*-D-glucopyranoside (41).

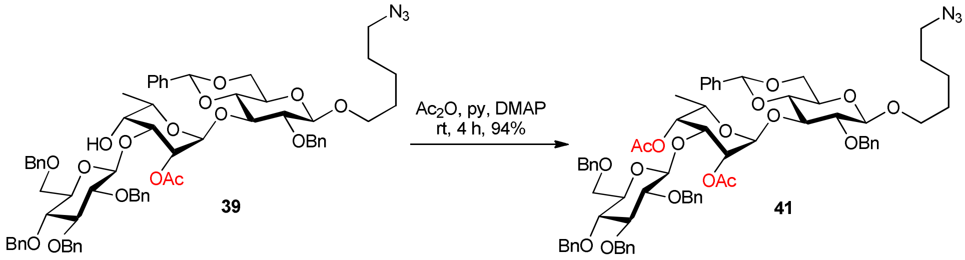

Ac_2O (0.4 mL) and DMAP (500 μg, 4.2 μmol, 0.1 equiv) were added to a solution of alcohol **39** (50 mg, 40 μmol, 1.0 equiv) in anhydrous py (0.4 mL). The reaction mixture was stirred at rt for 4 h under Ar. The solution was then concentrated under reduced pressure and co-evaporated with toluene (3 ×). The residue was purified by silica gel flash chromatography (PE/EtOAc 9:1 to 7:3) to give diacetylated trisaccharide **41** (49 mg, 94%) as a colorless oil: $[\alpha]_D^{20}$ = –46 (*c* 0.34, $CHCl_3$); ^{1}H NMR (400 MHz, $CDCl_3$) δ 7.39–7.12 (m, 30H, C*H*-Ar), 5.34–5.32 (m, 3H, H-1B, H-2B, H-7A), 5.13 (d, *J* = 3.1 Hz, 1H, H-4B), 4.86 (d, *J* = 10.8 Hz, 1H, C*H*HPh), 4.83 (d, *J* = 10.9 Hz, 1H, CH*H*Ph), 4.82 (d, *J* = 11.6 Hz, 1H, C*H*HPh), 4.78 (d, *J* = 10.7 Hz, 1H, CH*H*Ph), 4.75 (d, *J* = 10.7 Hz, 1H, C*H*HPh), 4.71 (d, *J* = 10.9 Hz, 1H, CH*H*Ph), 4.61 (d, *J* = 11.6 Hz, 1H, C*H*HPh), 4.59 (d, *J* = 12.4 Hz, 1H, CH*H*Ph), 4.57 (d, $J_{1C,2C}$ = 7.4 Hz, 1H, H-1C), 4.50 (d, *J* = 10.8 Hz, 1H, C*H*HPh), 4.49 (d, *J* = 12.4 Hz, 1H, CH*H*Ph), 4.48 (d, $J_{1A,2A}$ = 7.4 Hz, 1H, H-1A), 4.33–4.26 (m, 3H, H-3B, H-5B, H-6aA), 3.94 (t, *J* = 9.3 Hz, 1H, H-3A), 3.90 (dt, *J* = 9.5, 6.9 Hz, 1H, H-1a_{linker}), 3.72– 3.65 (m, 3H, H-6bA, H-6aC, H-6bC), 3.64–3.58 (m, 2H, H-3C, H-4C), 3.54 (dt, *J* = 9.5, 6.9 Hz, 1H, H-1b_{linker}), 3.49–3.36 (m, 5H, H-2A, H-2C, H-4A, H-5A, H-5C), 3.19 (t, *J* = 7.4 Hz, 2H, H-5_{linker}), 2.00 (s, 3H, CH_{3Ac}), 1.95 (s, 3H, CH_{3Ac}), 1.67–1.55 (m, 4H, H-2_{linker}, H-4_{linker}), 1.47–1.40 (m, 2H, H-3_{linker}), 0.77 (d, *J* = 6.5 Hz, 3H, CH_{3Tal}); ^{13}C NMR (100 MHz, $CDCl_3$) δ 170.8, 169.2 (2 × *C*O), 138.7, 138.6, 138.4, 138.3, 138.2, 137.2 (6 × C-Ar), 129.4–126.2 (18 × *C*H-Ar), 104.2 (C-1A), 101.8 (C-7A), 100.2 (C-1C), 98.8 (C-1B), 84.5 (C-3C), 83.2 (C-2A), 81.8 (C-2C), 79.1 (C-5C), 77.5 (C-4C), 75.9 (C-3A), 75.5 (*C*H_2Ph), 75.1 (C-4A), 75.0 (*C*H_2Ph), 74.9 (*C*H_2Ph), 74.1 (*C*H_2Ph), 73.4 (*C*H_2Ph), 70.2 (C-1_{linker}), 69.7 (C-3B), 68.9 (C-6A*), 68.8 (C-6C*), 68.3 (C-2B), 68.0 (C-4B), 66.3 (C-5A), 64.9 (C-5B), 51.3 (C-5_{linker}), 29.4 (C-2_{linker}), 28.7 (C-4_{linker}), 23.4 (C-3_{linker}), 21.2, 21.0 (2 × *C*H_{3Ac}), 15.9 (*C*H_{3Tal}); HRMS (ESI-TOF) *m/z* $[M + Na]^+$ calcd for $C_{69}H_{79}N_3NaO_{17}$ 1244.5302; found 1244.5309.

Allyl 4-*O*-Levulinoyl-3-*O*-methyl-*α*-L-rhamnopyranoside (**S36**).

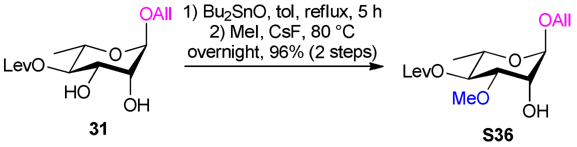

To a solution of diol **31** (500 mg, 1.7 mmol, 1.0 equiv) in toluene (7 mL) was added Bu_2SnO (465 mg, 1.9 mmol, 1.1 equiv) and the mixture was refluxed using a Dean-Stark apparatus for 5 h. The temperature was cooled to 30 °C, then CsF (263 mg, 1.7 mmol, 1.02 equiv) and MeI (5.2 mL, 85 mmol, 50 equiv) were successively added. After stirring overnight at 80 °C, the mixture was concentrated under reduced pressure. Purification by silica gel flash chromatography (PE/EtOAc 8:2 to 5:5) gave alcohol **S36** (503 mg, 96%) as a yellow oil: $[\alpha]_D^{20} = -48$ (*c*, 1.0, $CHCl_3$); ^1H NMR (400 MHz, $CDCl_3$) δ 5.89 (dddd, *J* = 16.6, 10.4, 6.1, 5.3 Hz, 1H, H-2_{All}), 5.29 (ddd, *J* = 17.2, 3.1, 1.6 Hz, 1H, H-$3a_{All}$), 5.20 (ddd, *J* = 11.6, 2.6, 1.2 Hz, 1H, H-$3b_{All}$), 4.97 (t, *J* = 9.7 Hz, 1H, H-4), 4.87 (d, *J* = 1.5 Hz, 1H, H-1), 4.15 (ddt, *J* = 12.9, 5.2, 1.4 Hz, 1H, H-$1a_{All}$), 4.07 (dd, *J* = 3.3, 1.7 Hz, 1H, H-2), 3.98 (ddt, *J* = 12.9, 6.2, 1.3 Hz, 1H, H-$1b_{All}$), 3.77 (dq, *J* = 10.1, 6.3 Hz, 1H, H-5), 3.52 (dd, *J* = 9.6, 3.4 Hz, 1H, H-3), 3.41 (s, 3H, -OCH_3), 2.85–2.54 (m, 4H, $(CH_2)_{2Lev}$), 2.17 (s, 3H, CH_{3Lev}), 1.17 (d, 3H, *J* = 6.3 Hz, CH_{3Rha}); ^{13}C NMR (100 MHz, $CDCl_3$) δ 206.6 (*C*O), 172.3 (*C*O), 133.8 (C-2_{All}), 117.8 (C-3_{All}), 98.3 (C-1), 78.9 (C-3), 73.0 (C-4), 68.2 (C-1_{All}), 67.8 (C-2), 66.2 (C-5), 57.7 (-OCH_3), 38.0 (CH_{2Lev}), 29.9 (CH_{3Lev}), 28.1 (CH_{2Lev}), 17.5 (CH_{3Rha}); HRMS (ESI-TOF) *m/z* $[M + Na]^+$ calcd for $C_{15}H_{24}NaO_7$ 339.1414; found 339.1407.

Allyl 2-*O*-Acetyl-4-*O*-levulinoyl-3-*O*-methyl-*α*-L-rhamnopyranoside (**S37**).

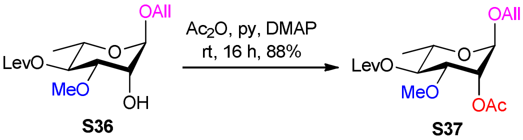

Alcohol **S36** (473 mg, 1.5 mmol, 1.0 equiv) was dissolved in anhydrous py (10 mL). Ac_2O (10 mL) and DMAP (2 mg, 15 µmol, 0.01 equiv) were added. The reaction mixture was stirred for 16 h at rt under Ar. Then, solvents were concentrated under reduced pressure and co-evaporated with toluene (3 ×). The residue was purified by silica gel flash chromatography (PE/EtOAc 8:2 to 65:35) to give derivative **S37** (520 mg, 88%) as a colorless oil: $[\alpha]_D^{20}$ = –39 (*c*, 1.8, $CHCl_3$); ^{1}H NMR (400 MHz, $CDCl_3$) δ 5.89 (ddd, *J* = 22.3, 10.9, 5.9 Hz, 1H, H-2_{All}) 5.32 (m, 2H, H-2, H-$3a_{All}$), 5.22 (dd, *J* = 10.4, 1.3 Hz, 1H, H-$3b_{All}$), 4.96 (t, *J* = 9.8 Hz, 1H, H-4), 4.78 (d, *J* = 1.6 Hz, 1H, H-1), 4.16 (ddt, *J* = 12.8, 5.2, 1.3 Hz, 1H, H-$1a_{All}$), 3.98 (ddt, *J* = 12.8, 6.2, 1.1 Hz, 1H, H-$1b_{All}$), 3.80 (dq, *J* = 9.8, 6.3 Hz, 1H, H-5), 3.61 (dd, *J* = 9.9, 3.4 Hz, 1H, H-3), 3.33 (s, 3H, -OCH_3), 2.88 – 2.48 (m, 4H, $(CH_2)_{2Lev}$), 2.18 (s, 3H, CH_{3Lev}), 2.11 (s, 3H, CH_{3Ac}), 1.20 (d, 3H, *J* = 6.3 Hz, CH_{3Rha}); ^{13}C NMR (100 MHz, $CDCl_3$) δ 206.6 (CO_{Lev}), 172.2 (CO_{Lev}), 170.5 (CO_{Ac}), 133.5 (C-2_{All}), 118.1 (C-3_{All}), 96.9 (C-1), 77.0 (C-3), 72.9 (C-4), 68.4 (C-1_{All}), 68.2 (C-2), 66.7 (C-5), 57.8 (-OCH_3), 38.0 (CH_{2Lev}), 30.0 (CH_{3Lev}), 28.1 (CH_{2Lev}), 21.1 (CH_{3Ac}), 17.5 (CH_{3Rha}); HRMS (ESI-TOF) *m/z* $[M + Na]^+$ calcd for $C_{17}H_{26}NaO_8$ 381.1520; found 381.1514.

2-*O*-Acetyl-4-*O*-levulinoyl-3-*O*-methyl-*α*-L-rhamnopyranosyl 2,2,2-Trichloroacetimidate (S38).

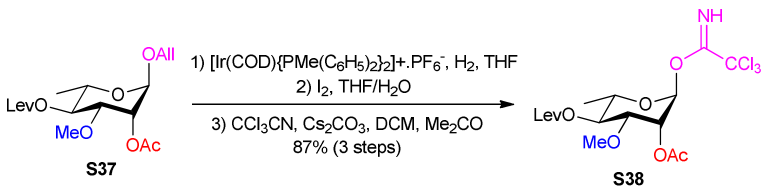

Derivative **S37** (500 mg, 1.4 mmol, 1.0 equiv) was reacted according to the general procedure for the synthesis of trichloroacetimidate donors. Purification by silica gel flash chromatography (PE/EtOAc 7:3 to 4:6 + 1% Et_3N) gave trichloroacetimidate donor **S38** (560 mg, 87%, over three steps) as a yellow oil: $[\alpha]_D^{20}$ = –28 (*c*, 2.4, $CHCl_3$); ^{1}H NMR (400 MHz, $CDCl_3$) δ 8.72 (s, 1H, N*H*), 6.20 (d, *J* = 1.9 Hz, 1H, H-1), 5.49 (dd, *J* = 3.3, 2.0, 1H, H-2), 5.06 (t, *J* = 9.9 Hz, 1H, H-4), 3.99 (dq, *J* = 10.0, 6.3 Hz, 1H, H-5), 3.66 (dd, *J* = 9.9, 3.4 Hz, 1H, H-3), 3.37 (s, 3H, CH_{3Me}), 2.90 – 2.48 (m, 4H, $(CH_2)_{2Lev}$), 2.18 (s, 3H, CH_{3Lev}), 2.15 (s, 3H, CH_{3Ac}), 1.24 (d, 3H, *J* = 6.4 Hz, CH_{3Rha}); ^{13}C NMR (100 MHz, $CDCl_3$) δ 206.5 (CO_{Lev}), 172.2 (CO_{Lev}), 170.1 (CO_{Ac}), 160.0 (C_{imine}), 95.1(C-1), 91.0 (CCl_3), 77.0 (C-3), 72.2 (C-4), 69.5 (C-5), 66.6 (C-2), 58.1 (CH_{3Me}), 38.0 (CH_{2Lev}), 29.9 (CH_{3Lev}), 28.0 (CH_{2Lev}), 21.0 (CH_{3Ac}), 17.5 (CH_{3Rha}); HRMS (ESI-TOF) *m/z* $[M + Na]^+$ calcd for $C_{16}H_{22}Cl_3NNaO_8$ 484.0303; found 484.0288.

(5-Azido-1-pentyl) 2-*O*-Acetyl-4-*O*-levulinoyl-3-*O*-methyl-*α*-L-rhamnopyranosyl-(1→3)-2-*O*-benzyl-4,6-*O*-benzylidene-*β*-D-glucopyranoside (S39).

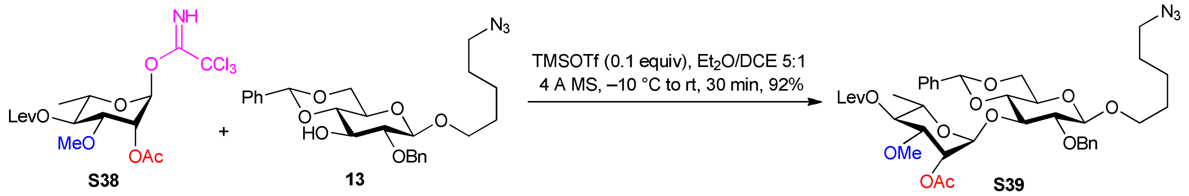

S38 **13** **S39**

Acceptor **13** (222 mg, 473 *μ*mol, 1.0 equiv) and donor **S38** (263 mg, 567 *μ*mol, 1.2 equiv) were dried for 4 h under high vacuum and then dissolved in anhydrous Et_2O/DCE (11 mL, 5:1 *v/v*). Freshly activated 4 Å powdered molecular sieves (890 mg) were added and the suspension was stirred for 40 min at rt under Ar. Then, the reaction mixture was cooled to −10 °C and TMSOTf (8.6 *μ*L, 47 *μ*mol, 0.1 equiv) was injected. The mixture was stirred at –10 °C for 10 min under Ar and after that time 20 min at rt. The reaction was then quenched with Et_3N (63 *μ*L), filtered over Celite and rinsed with DCM. The filtrate was concentrated under reduced pressure and purified by flash chromatography (PE/EtOAc 8:2 to 65:35) to give disaccharide **S39** (336 mg, 92%) as a white amorphous solid: $[\alpha]_D^{20}$ = –41 (*c*, 1.3, $CHCl_3$); ^{1}H NMR (400 MHz, $CDCl_3$) *δ* 7.50-7.20 (m, 10H, C*H*-Ar), 5.54 (s, 1H, H-7), 5.41 (dd, *J* = 3.3, 1.7 Hz, 1H, H-2B), 5.18 (d, *J* = 1.3 Hz, 1H, H-1B), 4.89 (d, *J* = 10.8 Hz, 1H, C*H*HPh), 4.83 (t, *J* = 9.9 Hz, 1H, H-4B), 4.70 (d, *J* = 10.8 Hz, 1H, CH*H*Ph), 4.50 (d, *J* = 7.8 Hz, 1H, H-1), 4.36 (dd, *J* = 10.5, 4.9 Hz, 1H, H-6aA), 4.12 (dq, *J* = 12.6, 6.3 Hz, 1H, H-5B), 3.98 – 3.88 (m, 2H, H-3, H-$1a_{linker}$), 3.78 (t, *J* = 10.2 Hz, 1H, H-6bA), 3.61 – 3.52 (m, 3H, H-4, H-3B, H-$1b_{linker}$), 3.49 – 3.38 (m, 2H, H-2, H-5), 3.34 (s, 3H, CH_{3Me}), 3.23 (t, *J* = 6.8 Hz, 2H, H-5_{linker}), 2.81 – 2.60 (m, 2H, CH_2-3_{Lev}), 2.50 (t, *J* = 6.7 Hz, 2H, CH_2-2_{Lev}), 2.17 (s, 3H, CH_{3Lev}), 2.06 (s, 3H, CH_{3Ac}), 1.72 – 1.57 (m, 4H, H-2_{linker}, H-4_{linker}), 1.52- 1.41 (m, 2H, H-3_{linker}), 0.80 (d, *J* = 6.2 Hz, 3H, CH_3-6B); ^{13}C NMR (100 MHz, $CDCl_3$) *δ* 206.4 (*C*O_{Lev}), 172.1 (*C*O_{Lev}), 170.1 (*C*O_{Ac}), 137.9, 137.2 (2 × C-Ar), 129.3 – 126.3 (6 × C-Ar), 104.3 (C-1), 101.8 (C-7), 98.2 (C-1B), 82.7 (C-2), 79.3 (C-4), 77.1 (C-3B), 76.1 (C-3), 75.0 (*C*H_2Ph), 72.9 (C-4B), 70.2 (C-1_{linker}), 68.9 (C-6), 67.9 (C-2B), 66.5 (C-5), 66.3 (C-5B), 57.8 (*C*H_{3Me}), 51.4 (C-5_{Linker}), 38.0 (C-3_{Lev}), 30.0 (*C*H_{3Lev}), 29.4 (C-2_{linker}), 28.8 (C-4_{linker}), 28.1 (C-2_{Lev}), 23.5 (C-3_{linker}), 21.1 (*C*H_{3Ac}), 16.9 (C-6B); HRMS (ESI-TOF) *m/z* $[M + Na]^+$ calcd for $C_{39}H_{51}N_3NaO_{13}$ 792.3314; found 792.3296.

(5-Azido-1-pentyl) 2-*O*-Acetyl-3-*O*-methyl-*α*-L-rhamnopyranosyl-(1→3)-2-*O*-benzyl-4,6-*O*-benzylidene-*β*-D-glucopyranoside (S40).

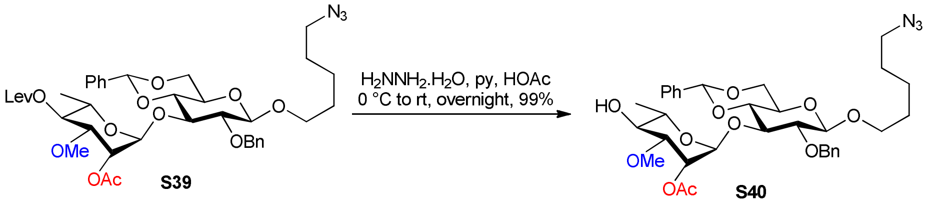

Acetic acid (2.1 mL) and hydrazine monohydrate (124 μL, 2.6 mmol, 5.0 equiv) were slowly added to a stirred solution of disaccharide **S39** (394 mg, 511 μmol, 1.0 equiv) in anhydrous Py (3.3 mL) at 0 °C under Ar. Then, the reaction mixture was stirred from 0 °C to rt overnight. After this time, solvents were concentrated and co-evaporated with toluene (3 ×). The residue was purified by silica gel flash chromatography (PE/EtOAc 85:15 to 8:2) to give alcohol **S40** (340 mg, 99%) as a white amorphous solid: $[\alpha]_D^{20}$ = –36 (*c*, 1.5, $CHCl_3$); ^{1}H NMR (400 MHz, $CDCl_3$) δ 7.50 – 7.23 (m, 10H, C*H*-Ar), 5.52 (s, 1H, H-7), 5.39 (dd, *J* = 3.0, 1.7 Hz, 1H, H-2B), 5.16 (d, *J* = 1.4 Hz, 1H, H-1B), 4.90 (d, *J* = 10.8 Hz, 1H, C*H*HPh), 4.70 (d, *J* = 10.8 Hz, 1H, CH*H*Ph), 4.50 (d, *J* = 7.8 Hz, 1H, H-1), 4.35 (dd, *J* = 10.5, 4.9 Hz, 1H, H-6aA), 4.02 (dq, *J* = 9.0, 6.2 Hz, 1H, H-5B), 3.97 – 3.89 (m, 2H, H-3, H-$1a_{linker}$), 3.77 (t, *J* = 10.2 Hz, 1H, H-6bA), 3.61 – 3.51 (m, 2H, H-4, H-$1b_{linker}$), 3.48 – 3.36 (m, 4H, H-2, H-3B, H-4B, H-5), 3.40 (s, 3H, CH_{3Me}), 3.22 (t, *J* = 6.8 Hz, 2H, H-5_{linker}), 2.04 (s, 3H, CH_{3Ac}), 1.72 – 1.56 (m, 4H, H-2_{linker}, H-4_{linker}), 1.52- 1.38 (m, 2H, H-3_{linker}), 0.93 (d, *J* = 6.2 Hz, 3H, CH_3-6B); ^{13}C NMR (100 MHz, $CDCl_3$) δ 170.1 (*C*O_{Ac}), 138.0, 137.2 (2 × C-Ar), 129.2 – 126.3 (6 × C-Ar), 104.3 (C-1), 101.8 (C-7), 98.7 (C-1B), 82.8 (C-2), 79.7 (C-3B), 79.3 (C-4), 76.5 (C-3), 75.0 (*C*H_2Ph), 71.8 (C-4B), 70.2 (C-1_{Linker}), 68.9 (C-6), 68.1 (C-5B), 67.5 (C-2B), 66.5 (C-5), 57.5 (*C*H_{3Me}), 51.4 (C-5_{Linker}), 29.4 (C-2_{Linker}), 28.8 (C-4_{Linker}), 23.5 (C-3_{Linker}), 21.0 (*C*H_{3Ac}), 17.2 (C-6B); HRMS (ESI-TOF) *m/z* $[M + Na]^+$ calcd for $C_{34}H_{45}N_3NaO_{11}$ 694.2946; found 694.2934.

(5-Azido-1-pentyl) 2-*O*-Acetyl-6-deoxy-3-*O*-methyl-*α*-L-talopyranosyl-(1→3)-2-*O*-benzyl-4,6-*O*-benzylidene-*β*-D-glucopyranoside (S41).

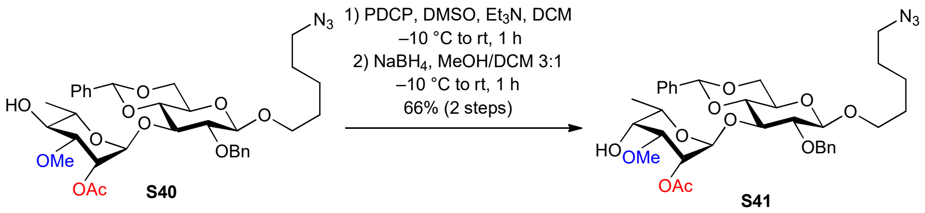

To a solution of DMSO (172 μL, 5 equiv) in anhydrous DCM (7 mL) at –10 °C under Ar were sequentially added phenyl dichlorophosphate (PDCP, 216 μL, 1.45 mmol, 3 equiv) and Et_3N (337 μL, 5 equiv). Then, a solution of disaccharide **S40** (324 mg, 483 μmol, 1.0 equiv) in DCM (4 mL) was added dropwise. The reaction mixture was stirred at –10 °C for 10 min, then allowed to slowly warm up to rt. After 1 h, DCM (20 mL) was added. The organic phase was washed with water (3 × 20 mL). The aqueous layer was back extracted with DCM (20 mL). The combined organic phases were washed with brine (20 mL). Then, the solvents of the dried solution ($MgSO_4$) were concentrated under reduced pressure to give a ketone. To a cooled (–10 °C) solution of the ketone in MeOH/DCM (10 mL, 3:1 *v/v*), $NaBH_4$ (55 mg, 1.4 mmol, 3.0 equiv) was slowly added. The mixture was stirred from –10 °C to rt under Ar for 1 h. Then, the reaction mixture was diluted with DCM (10 mL) and the organic phase was washed with water (3 × 10 mL). The aqueous layer was extracted with DCM (2 × 5 mL). The combined organic phases were washed with brine, dried ($MgSO_4$) and then concentrated under reduced pressure. The residue was purified by silica gel flash chromatography (PE/EtOAc 9:1 to 8:2) to give alcohol **S41** (213 mg, 66%, two steps) as a white solid: $[\alpha]_D^{20}$ = –57 (*c*, 1.1, $CHCl_3$); ^{1}H NMR (400 MHz, $CDCl_3$) δ 7.46 – 7.27 (m, 10H, C*H*-Ar), 5.50 (s, 1H, H-7), 5.33 (m, 1H, H-2B), 5.27 (s, 1H, H-1B), 4.90 (d, *J* = 10.9 Hz, 1H, C*H*HPh), 4.71 (d, *J* = 10.9 Hz, 1H, CH*H*Ph), 4.51 (d, *J* = 7.8 Hz, 1H, H-1), 4.34 (dd, *J* = 10.5, 4.8 Hz, 1H, H-6aA), 4.15 (q, *J* = 6.3 Hz, 1H, H-5B), 3.97 (t, *J* = 9.1, 1H, H-3), 3.93 (dt, *J* = 9.5, 6.4 Hz, 1H, H-$1a_{linker}$), 3.77 (t, *J* = 10.2 Hz, 1H, H-6bA), 3.60 – 3.42 (m, 6H, H-$1b_{linker}$, H-2, H-3B, H-4, H-4B, H-5), 3.40 (s, 3H, CH_{3Me}), 3.21 (t, *J* = 6.9 Hz, 2H, H-5_{linker}), 2.04 (s, 3H, CH_{3Ac}), 1.70 – 1.55 (m, 4H, H-2_{linker}, H-4_{linker}), 1.50- 1.40 (m, 2H, H-3_{linker}), 0.94 (d, *J* = 6.6 Hz, 3H, CH_3-6B); ^{13}C NMR (100 MHz, $CDCl_3$) δ 169.43 (*C*O_{Ac}), 138.1, 137.3 (2 × C-Ar), 129.4 – 126.3 (6 × C-Ar), 104.3 (C-1), 101.7(C-7), 98.7 (C-1B), 83.0 (C-2), 79.37 (C-4), 75.9 (C-3), 74.9 (*C*H_2Ph), 74.3 (C-3B), 70.3 (C-1_{Linker}), 69.6 (C-4B), 69.0 (C-6), 67.8 (C-5B), 66.9 (C-2B), 66.4 (C-5), 56.4 (*C*H_{3Me}), 51.4 (C-5_{Linker}), 29.4 (C-2_{Linker}), 28.7 (C-4_{Linker}), 23.5 (C-3_{Linker}), 21.2 (*C*H_{3Ac}), 16.1 (C-6B); HRMS (ESI-TOF) *m/z* $[M + Na]^+$ calcd for $C_{34}H_{45}N_3NaO_{11}$ 694.2946; found 694.2945.

(5-Amino-1-pentyl) *β*-D-Glucopyranosyl-(1→3)-4-*O*-acetyl-6-deoxy-2-*O*-methyl-*α*-L-talopyranosyl-(1→3)-*β*-D-glucopyranoside Hydrochloride (4).

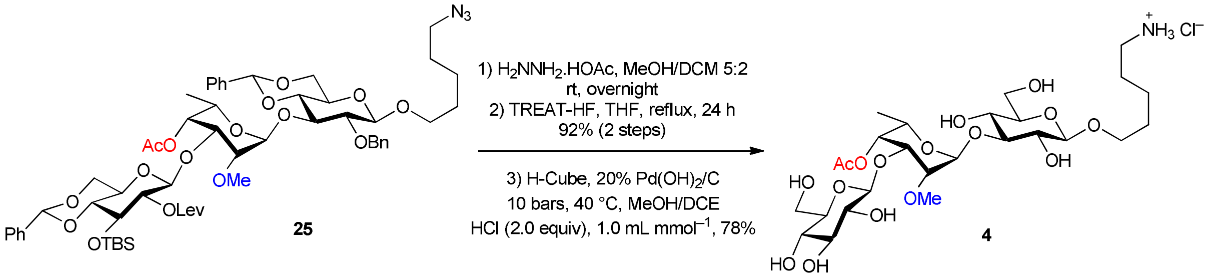

To a solution of trisaccharide **25** (983 mg, 1.1 mmol, 1.0 equiv) in anhydrous MeOH/DCM (0.3 mL, 5:2 *v/v*) was added hydrazine acetate (209 mg, 2.3 mmol, 2.0 equiv). After being stirred overnight at rt under Ar, the reaction mixture was concentrated under reduced pressure and co-evaporated with toluene (3 ×) to give an alcohol. For analytical data, a small sample was purified by silica gel flash chromatography (DCM/MeOH 98:2): $[\alpha]_D^{20}$ = –52 (*c* 1.2, $CHCl_3$); ^{1}H NMR (400 MHz, $CDCl_3$) δ 7.50–7.27 (m, 15H, C*H*-Ar), 5.51 (s, 1H, H-7A*), 5.49 (s, 1H, H-7C*), 5.35 (s, 1H, H-1B), 4.99 (d, *J* = 11.2 Hz, 1H, C*H*HPh), 4.93 (br s, 1H, H-4B), 4.66 (d, *J* = 11.2 Hz, 1H, CH*H*Ph), 4.52 (d, $J_{1A,2A}$ = 7.7 Hz, 1H, H-1A), 4.42 (d, $J_{1C,2C}$ = 7.6 Hz, 1H, H-1C), 4.33 (td, *J* = 11.0, 4.9 Hz, 2H, H-6aA, H-6aC), 4.22 (dd, *J* = 13.9, 6.4 Hz, 1H, H-5B), 3.92–3.88 (m, 1H, H-$1a_{linker}$), 3.91 (t, *J* = 9.7 Hz, 1H, H-3A), 3.86 (t, *J* = 4.1 Hz, 1H, H-3B), 3.79–3.71 (m, 3H, H-3C, H-6bA, H-6bC), 3.55–3.51 (m, 1H, H-$1b_{linker}$), 3.52 (t, *J* = 9.8 Hz, 1H, H-4A), 3.44–3.34 (m, 4H, H-2A, H-4C, H-5A, H-5C), 3.35–3.31 (m, 2H, H-2B, H-2C), 3.21 (s, 3H, CH_{3Me}), 3.20 (t, *J* = 7.0 Hz, 2H, H-5_{linker}), 1.69–1.56 (m, 4H, H-2_{linker}, H-4_{linker}), 1.50–1.42 (m, 2H, H-3_{linker}), 0.86 (s, 9H, $C(CH_3)_3$), 0.70 (d, *J* = 6.5 Hz, 3H, CH_{3Tal}), 0.10, 0.04 (2 × s, 6H, 2 × CH_3); ^{13}C NMR (100 MHz, $CDCl_3$) δ 173.0 (*C*O), 138.2, 137.4, 137.2 (3 × C-Ar), 129.5–126.3 (9 × *C*H-Ar), 105.7 (C-1C), 104.2 (C-1A), 102.2 (C-7A*), 101.7 (C-7C*), 99.7 (C-1B), 83.1 (C-2A), 81.5 (C-4C), 79.4 (C-4A), 78.1 (C-2B), 76.9 (C-3B), 76.8 (C-3A), 75.4 (C-2C), 74.9 (CH_2Ph), 74.2 (C-3C), 71.1 (C-4B), 70.2 (C-1_{linker}), 68.9 (C-6A), 68.8 (C-6C), 66.6 (C-5A), 66.5 (C-5C), 64.7 (C-5B), 60.0 (CH_{3Me}), 51.4 (C-5_{linker}), 29.4 (C-2_{linker}), 28.7 (C-4_{linker}), 25.9 ($C(CH_3)_3$), 23.5 (C-3_{linker}), 21.1 (CH_{3Ac}), 18.5 ($C(CH_3)_3$), 15.7 (CH_{3Tal}), –4.41, –4.44 (2 × CH_3); HRMS (ESI-TOF) *m/z* $[M + Na]^+$ calcd for $C_{53}H_{73}N_3NaO_{16}Si$ 1058.4652; found 1058.4690.

To a solution of the latter alcohol in anhydrous THF (5 mL) was added TREAT-HF (372 μL, 2.3 mmol, 15 equiv). The mixture was refluxed for 24 h under Ar, then additional TREAT-HF (124 μL, 760 μmol, 5.0 equiv) was added and the reaction was refluxed for another 24 h under Ar. The solution was cooled to rt and diluted with EtOAc (10 mL). The organic phase was washed with a saturated $NaHCO_3$(aq) solution (2 × 5 mL) and brine (10 mL). The solvents of the dried ($MgSO_4$) solution were concentrated under reduced pressure and the residue was purified by silica gel flash chromatography (PE/EtOAc 3:7 to 1:9) to give (5-azido-1-pentyl) 4,6-*O*-benzylidene-*β*-D-glucopyranosyl-(1→3)-4-*O*-acetyl-6-deoxy-2-*O*-methyl-*α*-L-talopyranosyl-(1→3)-2-*O*-benzyl-4,6-*O*-benzylidene-*β*-D-glucopyranoside (128 mg, 92%, two steps) as a white amorphous solid: R_f 0.4 (DCM/MeOH 98:2); $[\alpha]_D^{20}$ = –67 (*c* 1.2, $CHCl_3$); ^{1}H NMR (400 MHz, $CDCl_3$) δ 7.51–7.27 (m, 15H, C*H*-Ar), 5.54 (s, 1H, H-7A*), 5.49 (s, 1H, H-7C*), 5.35 (s, 1H, H-1B), 5.00 (d, *J* = 11.4 Hz, 1H, C*H*HPh), 4.92 (br s, 1H, H-4B), 4.65 (d, *J* = 11.4 Hz, 1H, CH*H*Ph), 4.53 (d, $J_{1A,2A}$ = 7.8 Hz, 1H, H-1A), 4.47 (d, $J_{1C,2C}$ = 7.5 Hz, 1H, H-1C), 4.35 (dd, *J* = 10.5, 4.8 Hz, 2H, H-6aA, H-6aC),

4.24 (dd, J = 13.9, 6.5 Hz, 1H, H-5B), 3.96 (t, J = 9.7 Hz, 1H, H-3A), 3.95–3.91 (m, 2H, H-1a$_{linker}$, H-3B), 3.82 (t, J = 9.6 Hz, 1H, H-3C), 3.77 (td, J = 10.8, 6.2 Hz, 2H, H-6bA, H-6bC), 3.59–3.52 (m, 3H, H-1b$_{linker}$, H-4A, H-4C), 3.50–3.44 (m, 3H, H-2A, H-5A, H-5C), 3.40 (dd, J = 9.1, 7.8 Hz, 1H, H-2C), 3.32 (d, J = 3.5 Hz, 1H, H-2B), 3.20 (t, J = 7.8 Hz, 2H, H-5$_{linker}$), 3.19 (s, 3H, CH_{3Me}), 2.12 (s, 3H, CH_{3Ac}), 1.69–1.56 (m, 4H, H-2$_{linker}$, H-4$_{linker}$), 1.50–1.40 (m, 2H, H-3$_{linker}$), 0.72 (d, J = 6.5 Hz, 3H, CH_{3Tal}); ^{13}C NMR (100 MHz, $CDCl_3$) δ 173.3 (*C*O), 138.2, 137.2, 137.1 (3 × C-Ar), 129.5–126.4 (9 × *C*H-Ar), 105.3 (C-1C), 104.2 (C-1A), 102.2 (C-7A*), 102.0 (C-7C*), 99.5 (C-1B), 83.1 (C-2A), 80.4 (C-4C), 79.4 (C-4A), 78.1 (C-2B), 77.4 (C-3B), 76.9 (C-3A), 74.9 (*C*H_2Ph), 74.6 (C-2C), 73.1 (C-3C), 71.1 (C-4B), 70.2 (C-1$_{linker}$), 68.9 (C-6A), 68.8 (C-6C), 66.6 (C-5A), 66.5 (C-5C), 64.5 (C-5B), 59.9 (*C*H_{3Me}), 51.3 (C-5$_{linker}$), 29.4 (C-2$_{linker}$), 28.7 (C-4$_{linker}$), 23.5 (C-3$_{linker}$), 21.4 (*C*H_{3Ac}), 15.7 (*C*H_{3Tal}); HRMS (ESI-TOF) *m/z* $[M + H]^+$ calcd for $C_{47}H_{60}N_3O_{16}$ 922.3968; found 922.3986; *m/z* $[M + Na]^+$ calcd for $C_{47}H_{59}N_3NaO_{16}$ 944.3788; found 944.3807; *m/z* $[2M + Na]^+$ calcd for $C_{94}H_{118}N_6NaO_{32}$ 1865.7683; found 1865.7707.

The latter compound (42.4 mg, 46.0 μmol) was reacted according to the general procedure for hydrogenolysis using the H-Cube system giving target trisaccharide **4** (24 mg, 78%) as a white amorphous powder: $[\alpha]_D^{20}$ = –56 (*c* 0.11, H_2O); ^{1}H NMR (400 MHz, D_2O) δ 5.40–5.36 (m, 2H, H-1B, H-4B), 4.58 (d, $J_{1,2}$ = 7.8 Hz, 1H, H-1A), 4.49–4.44 (m, 1H, H-5B), 4.47 (d, $J_{1,2}$ = 8.2 Hz, 1H, H-1C), 4.37 (t, $J_{2,3} \approx J_{3,4} \approx$ 3.8 Hz, 1H, H-3B), 3.95–3.88 (m, 3H, H-6aA, H-1a$_{linker}$, H-6aC), 3.76–3.65 (m, 4H, H-6bA, H-1b$_{linker}$, H-6bC, H-2B), 3.61 (t, $J_{2,3} \approx J_{3,4} \approx$ 8.8 Hz, 1H, H-3A), 3.52–3.35 (m, 6H, H-3C, H-5A, H-5C, H-4A, H-4C, H-2A), 3.42 (s, 3H, OCH_3), 3.28 (dd, $J_{2,3}$ = 9.1 Hz, $J_{1,2}$ = 7.9 Hz, 1H, H-2C), 3.00 (t, J = 7.5 Hz, 2H, H-5ab$_{linker}$), 2.17 (s, 3H, COCH_3), 1.73–1.63 (m, 4H, H-4ab$_{linker}$, H-2ab$_{linker}$), 1.50–1.42 (m, 2H, H-3ab$_{linker}$), 1.09 (d, $J_{5,6}$ = 6.6 Hz, 3H, H-6B); ^{13}C NMR (100 MHz, D_2O) δ 174.5 (*C*OCH_3), 102.6 (C-1A), 100.8 (C-1C), 95.5 (C-1B), 83.4 (C-3A), 78.3 (C-2B), 76.6, 76.5 (C-5A, C-5C), 74.4 (C-2C), 73.6 (C-2A), 71.5 (C-3B), 70.8 (C-6C), 70.2, 69.9 (C-4B, C-4A*), 68.6 (C-4C*), 66.1 (C-5B), 61.3 (C-6A, C-1$_{linker}$), 58.5 (O*C*H_3), 40.0 (C-5$_{linker}$), 28.8 (C-2$_{linker}$), 27.0 (C-4$_{linker}$), 22.7 (C-3$_{linker}$), 21.0 (CO*C*H_3), 15.8 (C-6B); HRMS (ESI-TOF) *m/z* $[M + H]^+$ calcd for $C_{26}H_{48}NO_{16}$ 630.2973; found 630.3018.

(5-Amino-1-pentyl) *β*-D-Glucopyranosyl-(1→3)-6-deoxy-2-*O*-methyl-*α*-L-talopyranosyl-(1→3)-*β*-D-glucopyranoside Hydrochloride (3).

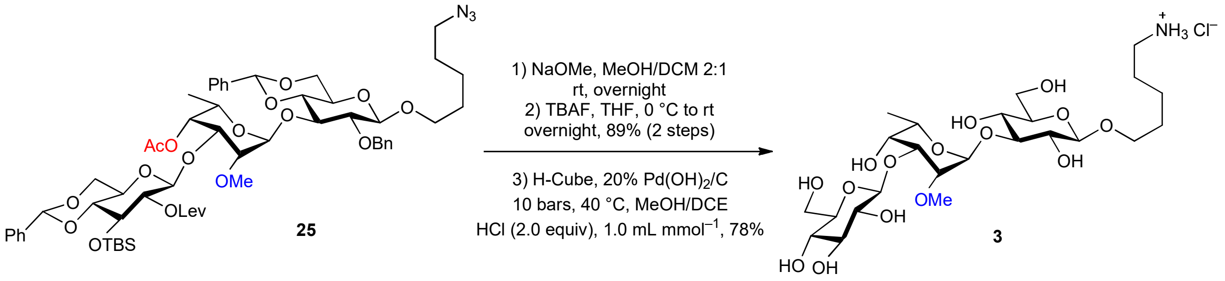

NaOMe (25% in MeOH, 40 μL, 168 μmol, 1.1 equiv) was added to a solution of trisaccharide **25** (173 mg, 153 μmol, 1.0 equiv) in anhydrous MeOH/DCM (5 mL, 2:1 *v/v*). The reaction mixture was stirred overnight at rt under Ar. Dowex H^+ resin was added to neutralize the reaction, then the suspension was filtered off and the solvents were concentrated under reduced pressure and co-evaporated with toluene (3 ×). The residue was dissolved in THF (8 mL). The solution was cooled to 0 °C and TBAF (1.5 mL, 1.0 M solution in THF, 1.5 mmol, 10 equiv) was added. After being stirred under Ar from 0 °C to rt overnight, the mixture was concentrated under reduced pressure. Purification by silica gel flash chromatography (DCM/MeOH 99:1 to 98:2) gave (5-azido-1-pentyl) 4,6-*O*-benzylidene-*β*-D-glucopyranosyl-(1→3)-6-deoxy-2-*O*-methyl-*α*-L-talopyranosyl-(1→3)-2-*O*-benzyl-4,6-*O*-benzylidene-*β*-D-glucopyranoside (120 mg, 89%, two steps) as a white foam: R_f 0.5 (DCM/MeOH 95:5); $[\alpha]_D^{20}$ = –58 (*c* 1.3, $CHCl_3$); ^1H NMR (400 MHz, $CDCl_3$) δ 7.50–7.32 (m, 15H, C*H*-Ar), 5.54 (s, 1H, H-7A*), 5.49 (s, 1H, H-7C*), 5.27 (d, *J* = 1.5 Hz, 1H, H-1B), 5.05 (d, *J* = 11.5 Hz, 1H, C*H*HPh), 4.58 (d, *J* = 11.5 Hz, 1H, CH*H*Ph), 4.52 (d, $J_{1A,2A}$ = 7.8 Hz, 1H, H-1A), 4.51 (d, $J_{1C,2C}$ = 7.6 Hz, 1H, H-1C), 4.34 (td, *J* = 10.3, 5.2, 4.8 Hz, 2H, H-6aA, H-6aC), 4.05 (dd, *J* = 13.7, 6.4 Hz, 1H, H-5B), 3.97–3.90 (m, 3H, H-$1a_{linker}$, H-3A, H-3B), 3.84–3.74 (m, 3H, H-3C, H-6bA, H-6bC), 3.61–3.45 (m, 8H, H-$1b_{linker}$, H-2B, H-2C, H-4A, H-4B, H-4C, H-5A, H-5C), 3.42 (dd, *J* = 9.2, 7.8 Hz, 1H, H-2A), 3.19 (t, *J* = 7.2 Hz, 2H, H-5_{linker}), 3.10 (s, 3H, CH_{3Me}), 1.69–1.56 (m, 4H, H-2_{linker}, H-4_{linker}), 1.48–1.40 (m, 2H, H-3_{linker}), 0.87 (d, *J* = 6.5 Hz, 3H, CH_{3Tal}); ^{13}C NMR (100 MHz, $CDCl_3$) δ 138.3, 137.2, 137.1 (3 × C-Ar), 129.5–126.4 (9 × *C*H-Ar), 104.2 (C-1A), 102.1 (C-7A*), 102.0 (C-7C*), 101.6 (C-1C), 98.4 (C-1B), 83.5 (C-2A), 80.4 (C-4C), 79.4 (C-4A), 79.3 (C-2B), 77.2 (C-3A), 75.1 (*C*H_2Ph), 74.9 (C-2C), 74.2 (C-3B), 73.3 (C-3C), 70.7 (C-4B), 70.2 (C-1_{linker}), 68.9 (C-6A), 68.8 (C-6C), 66.9 (C-5A), 66.8 (C-5C), 66.5 (C-5B), 59.5 (*C*H_{3Me}), 51.4 (C-5_{linker}), 29.4 (C-2_{linker}), 28.7 (C-4_{linker}), 23.5 (C-3_{linker}), 16.0 (*C*H_{3Tal}); HRMS (ESI-TOF) *m/z* $[M + Na]^+$ calcd for $C_{45}H_{57}N_3NaO_{15}$ 902.3682; found 902.3659.

The latter compound (62.8 mg, 71.4 μmol) was reacted according to the general procedure for hydrogenolysis using the H-Cube system giving target trisaccharide **3** (35 mg, 78%) as a white amorphous powder: $[\alpha]_D^{20}$ = –53 (*c* 0.18, H_2O); ^1H NMR (400 MHz, D_2O) δ 5.38 (s, 1H, H-1B), 4.63 (d, $J_{1,2}$ = 7.8 Hz, 1H, H-1C), 4.47 (d, $J_{1,2}$ = 8.1 Hz, 1H, H-1A), 4.28 (q, $J_{5,6}$ = 6.5 Hz, 1H, H-5B), 4.13 (t, $J_{2,3} \approx J_{3,4} \approx$ 3.3 Hz, 1H, H-3B), 3.94–3.88 (m, 3H, H-6aC, H-6aA, H-$1a_{linker}$), 3.86 (br s, 1H, H-4_B), 3.79 (br s, 1H, H-2_B), 3.76–3.66 (m, 3H, H-6bA, H-$1b_{linker}$, H-6bC), 3.61 (t, $J_{2,3} \approx J_{3,4} \approx$ 8.9 Hz, 1H, H-3A), 3.53–3.32 (m, 7H, H-3C, H-5A, H-5C, H-4A, H-4C, H-2A, H-2C), 3.42 (s, 3H, OCH_3), 2.99 (t, *J* = 7.5 Hz, 2H, H-$5ab_{linker}$), 1.73–1.62 (m, 4H, H-$4ab_{linker}$, H-$2ab_{linker}$), 1.49–1.41 (m, 2H, H-$3ab_{linker}$), 1.21 (d, $J_{5,6}$ = 6.5 Hz, 3H, H-6B); ^{13}C NMR (100 MHz, D_2O) δ 102.6 (C-

1A), 101.9 (C-1C), 98.4 (C-1B), 83.1 (C-3A), 79.2 (C-2B), 76.6, 76.5 (C-5A, C-5C), 76.1 (C-3C), 74.4, 74.3, 73.6 (C-2A, C-2B, C-2C), 70.6 (C-6C), 70.2, 70.1 (C-4B, C-4A*), 68.7 (C-4C*), 68.2 (C-5B), 61.4, 61.3 (C-6A, C-1_{linker}), 58.6 (O*C*H_3), 40.0 (C-5_{linker}), 28.8 (C-2_{linker}), 27.0 (C-4_{linker}), 22.7 (C-3_{linker}), 16.0 (C-6B); HRMS (ESI-TOF) *m/z* $[M + H]^+$ calcd for $C_{24}H_{46}NO_{15}$ 588.2867; found 588.2849.

(5-Amino-1-pentyl) *β*-D-Glucopyranosyl-(1→3)-2-*O*-acetyl-6-deoxy-*α*-L-talopyranosyl-(1→3)-*β*-D-glucopyranoside Hydrochloride (2).

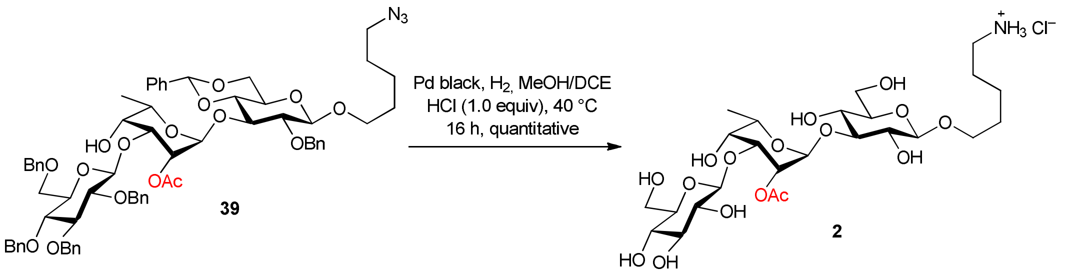

Protected trisaccharide **39** (50 mg, 42 *μ*mol, 1.0 equiv) was reacted according to the general procedure for hydrogenolysis under heterogeneous conditions to give target trisaccharide **2** (28 mg, quant.) as a white foam: $[\alpha]_D^{20}$ = –37 (*c* 0.27, H_2O); ^{1}H NMR (400 MHz, D_2O) *δ* 5.22–5.19 (m, 2H, H-1B, H-2B), 4.63 (d, $J_{1,2}$ = 7.9 Hz, 1H, H-1C), 4.45 (d, $J_{1,2}$ = 8.1 Hz, 1H, H-1A), 4.36 (q, $J_{5,6}$ = 6.6 Hz, 1H, H-5B), 4.27–4.24 (m, 1H, H-3B), 3.98–3.82 (m, 4H, H-4B, H-6aC, H-6aA, H-$1a_{linker}$), 3.75–3.64 (m, 3H, H-6bA, H-$1b_{linker}$, H-6bC), 3.58 (t, $J_{2,3} \approx J_{3,4} \approx$ 8.4 Hz, 1H, H-3A), 3.52–3.29 (m, 7H, H-3C, H-5A, H-5C, H-4A, H-4C, H-2A, H-2C), 3.06–2.97 (m, 2H, H-$5ab_{linker}$), 2.16 (s, 3H, COC*H*$_3$), 1.73–1.61 (m, 4H, H-$4ab_{linker}$, H-$2ab_{linker}$), 1.49–1.41 (m, 2H, H-$3ab_{linker}$), 1.23 (d, $J_{5,6}$ = 6.4 Hz, 3H, H-6B); ^{13}C NMR (100 MHz, D_2O) *δ* 176.2 (*C*OCH$_3$), 104.7 (C-1A), 104.6 (C-1C), 101.4 (C-1B), 85.0 (C-3A), 78.62, 78.60 (C-5A, C-5C), 78.3 (C-3C), 76.4 (C-2C), 75.72, 75.66 (C-2A, C-3B), 72.9 (C-2B, C-6C), 72.1 (C-4A*), 71.2 (C-4B), 70.9 (C-4C*), 69.9 (C-5B), 63.5, 63.3 (C-6A, C-1_{linker}), 42.2 (C-5_{linker}), 30.9 (C-2_{linker}), 29.2 (C-4_{linker}), 24.8 (C-3_{linker}), 23.4 (CO*C*H$_3$), 18.1 (C-6B); HRMS (ESI-TOF) *m/z* $[M + H]^+$ calcd for $C_{25}H_{46}NO_{16}$ 616.2817; found 616.2775.

(5-Amino-1-pentyl) β-D-Glucopyranosyl-(1→3)-6-deoxy-α-L-talopyranosyl-(1→3)-β-D-glucopyranoside Hydrochloride (1).

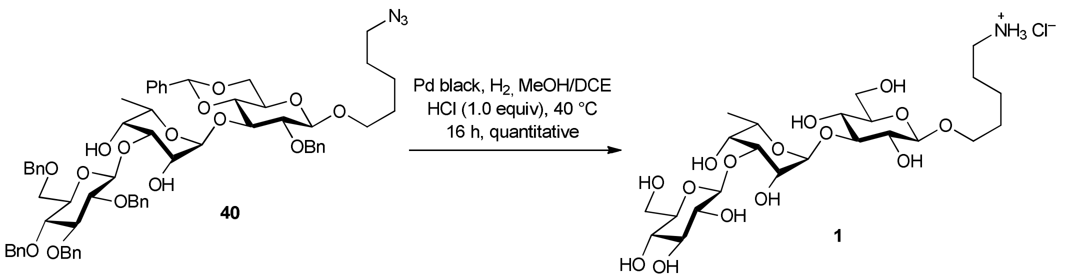

Trisaccharide **40** (20.2 mg, 17.7 μmol, 1.0 equiv) was reacted according to the general procedure for hydrogenolysis under heterogeneous conditions to give target trisaccharide **1** (10.8 mg, quant.) as a white amorphous powder: $[\alpha]_D^{20}$ = –42 (*c* 0.48, H_2O); ^1H NMR (400 MHz, D_2O) δ 5.25 (br s, 1H, H-1B), 4.66 (d, $J_{1,2}$ = 7.7 Hz, 1H, H-1C), 4.47 (d, $J_{1,2}$ = 8.0 Hz, 1H, H-1A), 4.33 (dt, $J_{4,5}$ = 12.9 Hz, $J_{5,6a} \approx J_{5,6b} \approx$ 6.4 Hz, 1H, H-5B), 4.15–4.07 (m, 2H, H-2B, H-3B), 3.97–3.87 (m, 4H, H-4B, H-6aA, H-6aC, H-1a_{linker}), 3.76–3.59 (m, 4H, H-1b_{linker}, H-6bA, H-6bC, H-3A), 3.54–3.33 (m, 7H, H-3C, H-5A, H-5C, H-4A, H-4C, H-2A, H-2C), 3.00 (t, *J* = 6.9 Hz, 2H, H-5ab_{linker}), 1.73–1.60 (m, 4H, H-4ab_{linker}, H-2ab_{linker}), 1.50–1.40 (m, 2H, H-3ab_{linker}), 1.24 (d, $J_{5,6}$ = 6.4 Hz, 3H, H-6B); ^{13}C NMR (100 MHz, D_2O) δ 104.7 (C-1A), 104.2 (C-1B), 103.9 (C-1C), 84.9 (C-3A), 78.7 (C-5C), 78.6 (C-5A), 78.3 (C-3C), 76.5 (C-2C), 76.3 (C-3B), 75.8 (C-2A), 72.9 (C-6C), 72.6 (C-4B), 72.4 (C-2B), 72.3 (C-4C), 70.9 (C-4A), 70.3 (C-5B), 63.5, 63.4 (C-6A, C-1_{linker}), 42.1 (C-5_{linker}), 30.9 (C-2_{linker}), 29.2 (C-4_{linker}), 24.8 (C-3_{linker}), 18.2 (C-6B); HRMS (ESI-TOF) *m/z* $[M + H]^+$ calcd for $C_{23}H_{44}NO_{15}$ 574.2711; found 574.2762.

(5-Amino-1-pentyl) *β*-D-Glucopyranosyl-(1→3)-2,4-di-*O*-acetyl-6-deoxy-*α*-L-talopyranosyl-(1→3)-*β*-D-glucopyranoside Hydrochloride (5).

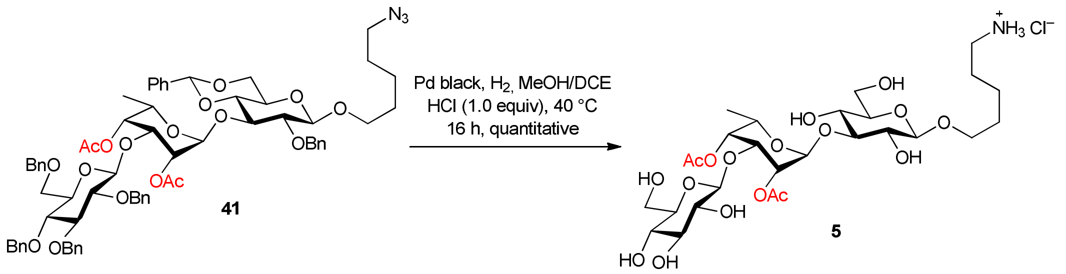

Trisaccharide **41** (45.0 mg, 36.8 *μ*mol) was reacted according to the general procedure for hydrogenolysis under heterogeneous conditions giving deprotected trisaccharide **5** (26 mg, quant.) as a white foam: $[\alpha]_D^{20}$ = –40 (*c* 0.17, H_2O); ^1H NMR (400 MHz, D_2O) *δ* 5.40 (br s, 1H, H-4B), 5.26–5.22 (m, 2H, H-1B, H-2B), 4.60 (d, $J_{1,2}$ = 7.9 Hz, 1H, H-1C), 4.54 (q, $J_{5,6}$ = 6.5 Hz, 1H, H-5B), 4.49 (t, $J_{2,3} \approx J_{3,4} \approx$ 3.5 Hz, 1H, H-3B), 4.45 (d, $J_{1,2}$ = 8.1 Hz, 1H, H-1A), 3.96–3.83 (m, 3H, H-6aC, H-6aA, H-1a_{linker}), 3.74–3.65 (m, 3H, H-6bA, H-1b_{linker}, H-6bC), 3.62–3.60 (m, 1H, H-3A), 3.50–3.35 (m, 6H, H-3C, H-5A, H-5C, H-4A, H-4C, H-2A), 3.22 (t, $J_{1,2} \approx J_{2,3} \approx$ 8.3 Hz, 1H, H-2C), 3.00 (t, *J* = 7.4 Hz, 2H, H-5ab_{linker}), 2.22 (s, 3H, COCH_3), 2.18 (s, 3H, COCH_3), 1.73–1.62 (m, 4H, H-4ab_{linker}, H-2ab_{linker}), 1.46–1.41 (m, 2H, H-3ab_{linker}), 1.13 (d, $J_{5,6}$ = 6.5 Hz, 3H, H-6B); ^{13}C NMR (100 MHz, D_2O) *δ* 174.5, 173.9 (2 × *C*OCH3), 102.6 (C-1A), 101.9 (C-1C), 99.4 (C-1B), 83.0 (C-3A), 76.51, 76.46 (C-5A, C-5C), 76.1 (C-3C), 74.2 (C-2A), 73.4 (C-2C), 71.7 (C-3B), 70.7 (C-6C), 70.2 (C-4B), 70.0, 69.9 (C-2B, C-4A*), 68.7 (C-4C*), 66.3 (C-5B), 61.3, 61.2 (C-6B, C-1_{linker}), 40.0 (C-5_{linker}), 28.8 (C-2_{linker}), 27.0 (C-4_{linker}), 22.7 (C-3_{linker}), 21.2, 21.0 (2 × CO*C*H3), 15.7 (C-6B); HRMS (ESI-TOF) *m/z* $[M + H]^+$ calcd for $C_{27}H_{48}NO_{17}$ 658.2922; found 658.2925.

(5-Amino-1-pentyl) 2-*O*-Acetyl-6-deoxy-3-*O*-methyl-*α*-L-talopyranosyl-(1→3)-*β*-D-glucopyranoside Hydrochloride (6).

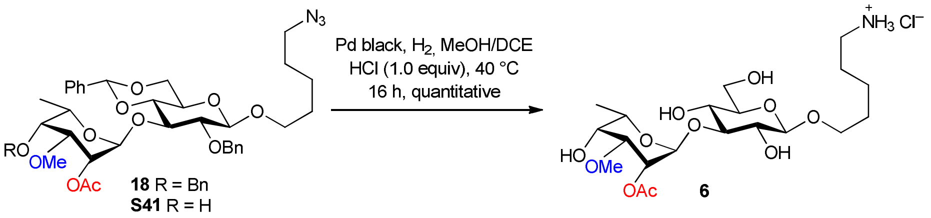

Disaccharide **18** (15.4 mg, 20.2 *μ*mol) or disaccharide **S41** (50 mg, 74 *μ*mol) was reacted according to the representative procedure for hydrogenolysis under heterogeneous conditions giving target disaccharide **6** (10.2 mg or 38 mg, quant.) as a white amorphous powder: $[\alpha]_D^{20}$ = –16 (*c* 0.12, H_2O); ^1H NMR (400 MHz, D_2O) *δ* 5.25–5.23 (m, 1H, H-2B), 5.21 (s, 1H, H-1B), 4.45 (d, $J_{1,2}$ = 8.1 Hz, 1H, H-1A), 4.34 (q, $J_{5,6}$ = 6.5 Hz, 1H, H-5B), 3.97–3.89 (m, 3H, H-4B, H-6aA, H-1a_{linker}), 3.78 (t, $J_{2,3} \approx J_{3,4} \approx$ 3.6 Hz, 1H, H-3B), 3.74–3.65 (m, 2H, H-1b_{linker}, H-6bA), 3.58 (t, $J_{2,3} \approx J_{3,4} \approx$ 8.9 Hz, 1H, H-3A), 3.47–3.34 (m, 3H, H-5A, H-4A, H-2A), 3.41 (s, 3H, OC*H*$_3$), 3.07–2.98 (m, 2H, H-5ab_{linker}), 2.14 (s, 3H, COC*H*$_3$), 1.73–1.62 (m, 4H, H-4ab_{linker}, H-2ab_{linker}), 1.49–1.41 (m, 2H, H-3ab_{linker}), 1.23 (d, $J_{5,6}$ = 6.6 Hz, 3H, H-6B); ^{13}C NMR (100 MHz, D_2O) *δ* 173.9 (*C*OCH$_3$), 102.6 (C-1A), 99.5 (C-1B), 82.9 (C-3A), 76.5 (C-5A); 74.3 (C-3B, C-2A), 70.7 (C-6A), 68.8 (C-4A), 68.6 (C-2B), 68.1 (C-4B), 67.7 (C-5B), 61.3 (C-1_{linker}), 56.1 (O*C*H$_3$), 40.0 (C-5_{linker}), 28.8 (C-2_{linker}), 27.0 (C-4_{linker}), 22.7 (C-3_{linker}), 21.1 (CO*C*H$_3$), 16.0 (C-6B); HRMS (ESI-TOF) *m/z* $[M + H]^+$ calcd for $C_{20}H_{38}NO_{11}$ 468.2445; found 468.2449.

(5-Amino-1-pentyl) 2,4-Di-*O*-acetyl-6-deoxy-3-*O*-methyl-*α*-L-talopyranosyl-(1→3)-*β*-D-glucopyranoside Hydrochloride (7).

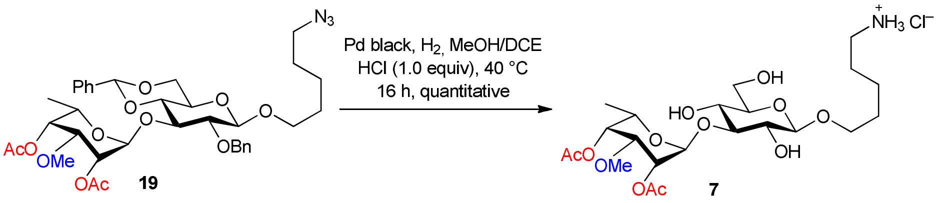

Disaccharide **19** (99 mg, 14 *μ*mol) was reacted according to the representative procedure for hydrogenolysis under heterogeneous conditions giving target disaccharide **7** (76 mg, quant.) as a white amorphous powder: $[\alpha]_D^{20}$ = –24 (*c* 0.08, H_2O); ^1H NMR (400 MHz, D_2O) δ 5.37 (br d, $J_{3,4}$ = 3.2 Hz, 1H, H-4B), 5.27 (br d, $J_{2,3}$ = 3.8 Hz, 1H, H-2B), 5.25 (s, 1H, H-1B), 4.52 (q, $J_{5,6}$ = 6.6 Hz, 1H, H-5B), 4.45 (d, $J_{1,2}$ = 8.1 Hz, 1H, H-1A), 3.97 (t, $J_{2,3} \approx J_{3,4} \approx$ 3.6 Hz, 1H, H-3B), 3.95–3.88 (m, 2H, H-6aA, H-1a_{linker}), 3.74–3.65 (m, 2H, H-1b_{linker}, H-6bA), 3.62–3.57 (m, 1H, H-3A), 3.47–3.34 (m, 3H, H-5A, H-4A, H-2A), 3.40 (s, 3H, OCH_3), 3.06–2.97 (m, 2H, H-5ab_{linker}), 2.21 (s, 3H, COCH_3), 2.16 (s, 3H, COCH_3), 1.73–1.62 (m, 4H, H-4ab_{linker}, H-2ab_{linker}), 1.49–1.41 (m, 2H, H-3ab_{linker}), 1.13 (d, $J_{5,6}$ = 6.6 Hz, 3H, H-6B); ^{13}C NMR (100 MHz, D_2O) δ 174.4, 173.7 (2 × *C*OCH$_3$), 102.6 (C-1A), 99.6 (C-1B), 83.1 (C-3A), 76.5 (C-5A), 74.3 (C-2A), 73.6 (C-3B), 70.8 (C-6A), 70.1 (C-4B), 68.7 (C-4A), 68.1 (C-2B), 66.2 (C-5B), 61.3 (C-1_{linker}), 57.3 (O*C*H$_3$), 40.0 (C-5_{linker}), 28.8 (C-2_{linker}), 27.0 (C-4_{linker}), 22.7 (C-3_{linker}), 21.1, 20.9 (2 × CO*C*H$_3$), 15.7 (C-6B); HRMS (ESI-TOF) *m/z* $[M + H]^+$ calcd for $C_{22}H_{40}NO_{12}$ 510.2551; found 510.2541.

Biotinylated Disaccharide 6 (BIO-6).

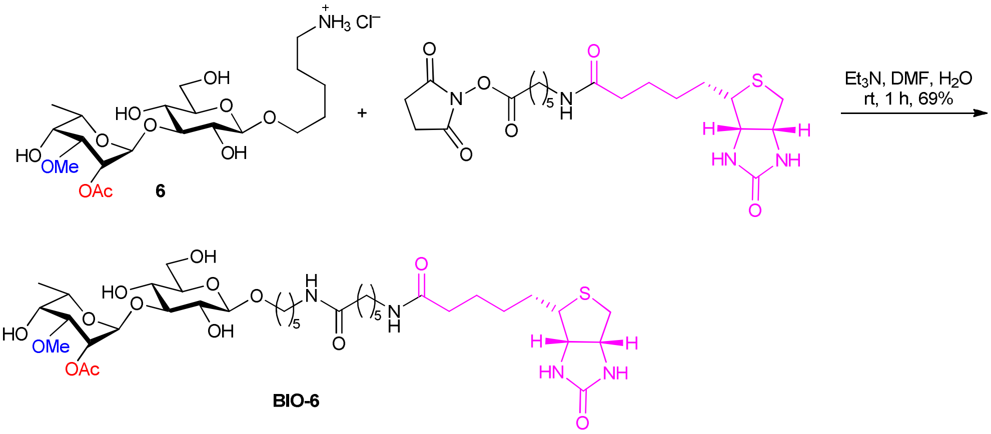

Disaccharide **6** (10 mg, 20 μmol, 1.0 equiv) was reacted according to the general procedure for the synthesis of biotinylated oligosaccharides to give derivative **BIO-6** (11 mg, 69%) as a white amorphous powder: $[\alpha]_D^{20}$ = 12 (*c*, 1.0, MeOH); ^{1}H NMR (400 MHz, $CDCl_3$) δ 5.32 (s, 1H, H-1B), 5.28 (s, 1H, H-2B), 4.50 (dd, *J* = 7.1, 4.7 Hz, 1H, H-8_{biotin}), 4.42-4.35 (m, 1H, H-5B), 4.34-4.28 (m, 1H, H-7_{biotin}), 4.28-4.22 (m, 1H, H-1A), 3.96-3.83 (m, 2H, H-6aA, H-1_{linker}), 3.76 (s, 1H, H-4B), 3.73-3.62 (m, 2H, H-3B, H-1_{linker}), 3.60-3.50 (m, 2H, H-3A, H-6bA), 3.41 (s, 3H, CH_{3Me}), 3.38-3.27 (m, 2H, H-2A, H-5H), 3.26-3.27 (m, 5H, H-5_{linker}, H-6_{biotin}, H-1'$_{biotin}$), 2.93 (dd, *J* = 12.7, 4.7 Hz, 1H, H-$9a_{biotin}$), 2.71 (d, *J* = 13.0 Hz, 1H, H-$9b_{biotin}$), 2.24-2.14 (m, 4H, H-2_{biotin}, H-5'$_{biotin}$), 2.10 (s, 3H, CH_{3Ac}), 1.80-1.28 (m, 18H, H-2_{linker}, H-3_{linker}, H-4_{linker}, H-3_{biotin}, H-4_{biotin}, H-5_{biotin}, H-2'$_{biotin}$, H-3'$_{biotin}$, H-4'$_{biotin}$), 1.26 (d, *J* = 6.5 Hz, 3H, CH_3-6B); ^{13}C NMR (100 MHz, $CDCl_3$) δ 176.0-171.8 (3 × *C*O), 166.1 (C-10_{biotin}), 104.2 (C-1A), 100.2 (C-1B), 83.1 (C-3A), 77.8 (C-5A), 75.9, 75.8 (C-2A, C-3B), 70.7 (C-6A), 70.1 (C-4A), 70.1 (C-4B), 69.1 (C-2B), 68.0 (C-5B), 63.4 (C-7_{biotin}), 62.7 (C-1_{linker}), 61.6 (C-8_{biotin}), 57.0 (C-6_{biotin}), 56.6 (*C*H_{3Me}), 41.04 (C-9_{biotin}), 40.3, 40.2 (C-1'$_{biotin}$, C-5_{linker}), 37.0, 36.8 (C-2_{biotin}, C-5'$_{biotin}$), 30.3-24.4 (9 × CH_2), 20.9 (*C*H_{3Ac}), 16.7 (C-6B); HRMS (ESI-TOF) *m/z* $[M + Na]^+$ calcd for $C_{36}H_{62}N_4NaO_{14}S$ 829.3875; found 829.3881.

Biotinylated Disaccharide 7 (BIO-7).

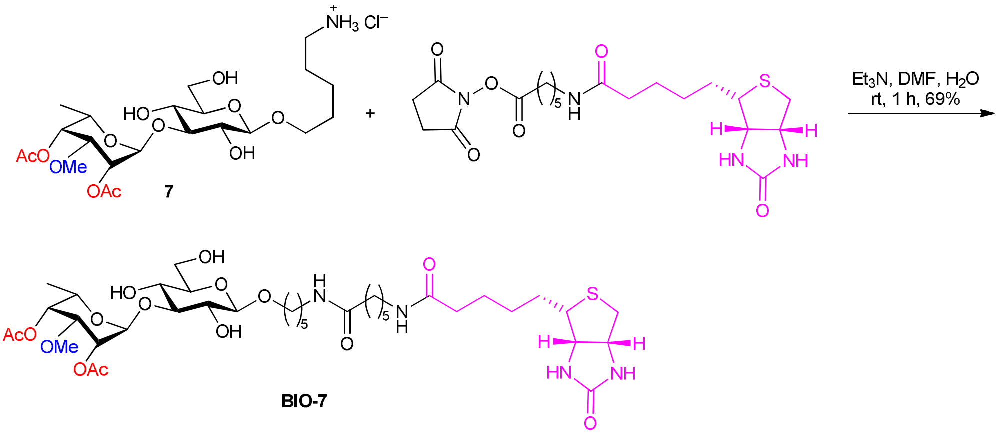

Disaccharide **7** (10 mg, 18 μmol, 1.0 equiv) was reacted according to the general procedure for the synthesis of biotinylated oligosaccharides to give derivative **BIO-7** (11 mg, 69%) as a white amorphous powder: $[\alpha]_D^{20}$ = -8 (*c*, 0.9, MeOH); ^{1}H NMR (400 MHz, $CDCl_3$) δ 5.36 (s, 1H, H-1B), 5.24 (m, 2H, H-2B and H-4B), 4.52 (m, 2H, H-5B, H-8_{biotin}), 4.31 (dd, *J* = 7.6, 4.3 Hz, 1H, H-7_{biotin}), 4.24 (m, 1H, H-1A), 3.89 (m, 2H, H-6aA, H-$1a_{linker}$), 3.78 (t, *J* = 3.4 Hz, 1H, H-3B), 3.68 (m, 1H, H-$1b_{linker}$), 3.54 (m, 2H, H-6aA and H-3A), 3.37 (s, 3H, CH_{3Me}), 3.36 – 3.29 (m, 3H, H-2A, H-4A, H-5A), 3.26 – 3.12 (m, 5H, H-5_{linker}, H-1'$_{biotin}$, H-6_{biotin}), 2.93 (dd, *J* = 12.7, 4.8, 1H, H-$9a_{biotin}$), 2.71 (d, *J* = 13.2 Hz, 1H, H-$9b_{biotin}$), 2.19 (m, 4H, H-2_{biotin}, H-5'$_{biotin}$), 2.11, 2.09 (2 × s, 6H, 2 × CH_{3Ac}), 1.79 – 1.26 (m, 18H, 9 × CH_2), 1.12 (d, *J* = 6.4 Hz, 3H, CH_3-6B); ^{13}C NMR (100 MHz, $CDCl_3$) δ 176.0 - 172.0 (4 × *C*O), 166.1 (C-10_{biotin}), 104.2 (C-1A), 100.2 (C-1B), 82.8 (C-3A), 77.8 (C-5A), 75.9 (C-2A), 74.9 (C-3B), 70.7 (C-6A), 70.4 (C-4B), 70.1 (C-4A), 68.4 (C-2B), 66.4 (C-5B), 63.4 (C-7_{biotin}), 62.7 (C-1_{linker}), 61.6 (C-8_{biotin}), 57.3 (*C*H_{3Me}), 57.0 (C-6_{biotin}), 47.9 (C-1_{Et3N}), 41.1 (C-9_{biotin}), 40.3, 40.2 (C-5_{linker}, C-1'$_{biotin}$), 30.3 – 24.4 (9 × *C*H_2), 21.0, 20.9 (2 × *C*H_{3Ac}), 16.5 (C-6B), 9.2 (C-2_{Et3N}); HRMS (ESI-TOF) *m/z* $[M + Na]^+$ calcd for $C_{38}H_{64}N_4NaO_{15}S$ 871.3981; found 871.3992.

Biotinylated Trisaccharide 2 (BIO-2).

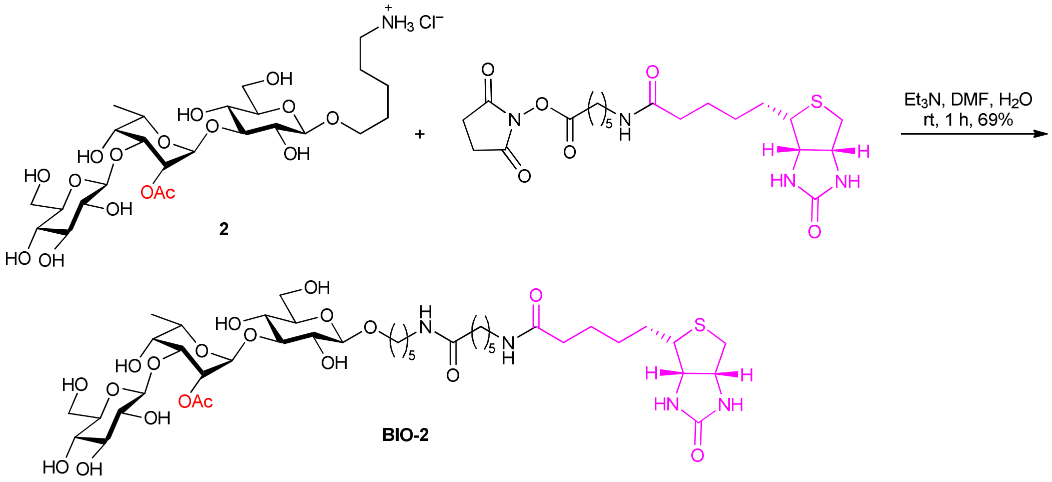

Trisaccharide **2** (10 mg, 15 μmol, 1.0 equiv) was reacted according to the general procedure for the synthesis of biotinylated oligosaccharides to give derivative **BIO-2** (8 mg, 55%) as a white amorphous powder: $[\alpha]_D^{20}$ = 11 (*c*, 0.7, MeOH); ^{1}H NMR (400 MHz, $CDCl_3$) δ 5.32 (s, 1H, H-1B), 5.24 (d, *J* = 3.7 Hz, H-2B), 4.52 – 4.46 (m, 2H, H-1C, H-8_{biotin}), 4.43-4.37 (m, 1H, H-5B), 4.34-4.28 (m, 1H, H-7_{biotin}), 4.24 (d, *J* = 7.9 Hz, 1H, H-1A), 4.20 (t, *J* = 3.6 Hz, 1H, H-3B), 3.93-3.81 (m, 4H, H-6aA, H-4B, H-6aC, H-$1a_{linker}$), 3.72-3.62 (m, 2H, H-6bC, H-$1b_{linker}$), 3.58-3.51 (m, 2H, H-3A, H-6bA), 3.40-3.14 (m, 12H, H-2A, H-4A, H-5A, H-2C, H-3C, H-4C, H-5C, H-5_{linker}, H-6_{biotin}, H-$1'_{biotin}$), 2.94 (dd, *J* = 12.8, 4.9 Hz, 1H, H-$9a_{biotin}$), 2.71 (d, *J* = 12.8 Hz, 1H, H-$9b_{biotin}$), 2.25-2.14 (m, 4H, H-2_{biotin}, H-$5'_{biotin}$), 2.12 (s, 3H, CH_{3Ac}), 1.80-1.31 (m, 18H, H-2_{linker}, H-3_{linker}, H-4_{linker}, H-3_{biotin}, H-4_{biotin}, H-5_{biotin}, H-$2'_{biotin}$, H-$3'_{biotin}$, H-$4'_{biotin}$), 1.28 (d, *J* = 6.6 Hz, 3H, CH_3-6B); ^{13}C NMR (100 MHz, $CDCl_3$) δ 176.1-172.4 (3 × *C*O), 166.2 (C-10_{biotin}), 104.3 (C-1A), 103.1 (C-1C), 99.9 (C-1B), 82.8 (C-3A), 78.1-77.8 (C-5A, C-3C, C-5C), 75.8, 75.0 (C-2A, C-2C), 73.9 (C-3B), 71.6 (C-2B), 71.4 (C-4A), 70.7 (C-6A), 70.2, 70.1 (C-4B, C-4C), 67.8 (C-5B), 63.4 (C-7_{biotin}), 62.7, 62.7 (C-6C, C-1_{linker}), 61.6 (C-8_{biotin}), 57.0 (C-6_{biotin}), 41.0 (C-9_{biotin}), 40.3, 40.2 (C-5_{linker}, C-$1'_{biotin}$), 36.8 (C-2_{biotin}, C-$5'_{biotin}$), 30.3-24.4 (9 × CH_2), 21.1 (CH_{3Ac}), 16.7 (C-6B); HRMS (ESI-TOF) *m/z* $[M + Na]^+$ calcd for $C_{41}H_{70}N_4NaO_{19}S$ 977.4247; found 977.4261.

Crystal data and structure refinement of compound 36

Identification code	bl7_a	
Empirical formula	$C_{42} H_{46} O_{10}$	
Formula weight	710.79	
Temperature	200(2) K	
Wavelength	0.71073 Å	
Crystal system	Monoclinic	
Space group	$P2_1$	
Unit cell dimensions	a = 13.068(2) Å	α= 90°.
	b = 8.6099(13) Å	β= 102.761(7)°.
	c = 16.359(2) Å	γ = 90°.
Volume	1795.1(5) Å^3	
Z	2	
Density (calculated)	1.315 Mg/m^3	
Absorption coefficient	0.093 mm^{-1}	
F(000)	756	
Crystal size	0.300 x 0.080 x 0.040 mm^3	
Theta range for data collection	2.255 to 30.062°.	
Index ranges	-18<=h<=18, -12<=k<=12, -23<=l<=22	
Reflections collected	122253	
Independent reflections	10457 [R(int) = 0.0505]	
Completeness to theta = 25.242°	99.4 %	
Absorption correction	Semi-empirical from equivalents	
Refinement method	Full-matrix least-squares on F^2	
Data / restraints / parameters	10457 / 1 / 471	
Goodness-of-fit on F^2	1.147	
Final R indices [I>2sigma(I)]	R1 = 0.0465, wR2 = 0.1322	
R indices (all data)	R1 = 0.0637, wR2 = 0.1548	
Absolute structure parameter	0.06(13)	
Extinction coefficient	n/a	
Largest diff. peak and hole	0.777 and -0.912 e.Å^{-3}	

Supplementary Table 2 │ Atomic coordinates (x 10^4) and equivalent isotropic displacement parameters (Å^{2}x 10^3). U(eq) is defined as one third of the trace of the orthogonalized U^{ij} tensor.

	x	y	z	U(eq)
C(1)	4627(2)	4249(3)	5691(1)	22(1)
C(2)	4984(2)	4696(3)	4897(1)	22(1)
C(3)	4194(2)	4168(2)	4118(1)	21(1)
C(4)	3102(2)	4762(3)	4148(1)	24(1)
O(5)	2833(1)	4293(2)	4914(1)	26(1)
C(6)	3527(2)	4892(3)	5637(1)	23(1)
O(7)	3172(2)	4378(2)	6332(1)	30(1)
C(8)	2541(2)	5447(3)	6667(1)	25(1)
C(9)	1460(2)	4781(4)	6651(2)	33(1)
C(10)	936(2)	5755(4)	7253(2)	37(1)
O(11)	1372(2)	7231(3)	7435(1)	37(1)
C(12)	2484(2)	7234(3)	7812(2)	30(1)
C(13)	3002(2)	5755(3)	7591(1)	24(1)
O(14)	1611(2)	3300(2)	7062(1)	36(1)
C(15)	1866(2)	3731(3)	7911(2)	32(1)
O(16)	2872(1)	4427(2)	8106(1)	27(1)
O(17)	1083(2)	4824(3)	7986(1)	38(1)
C(18)	2691(3)	7591(4)	8740(2)	41(1)
C(19)	1850(3)	2376(4)	8479(2)	47(1)
O(20)	5343(1)	4887(2)	6396(1)	27(1)
C(21)	5594(3)	3825(3)	7082(2)	43(1)
C(22)	6424(2)	4515(3)	7773(2)	37(1)
C(23)	6273(3)	4452(4)	8584(2)	47(1)
C(24)	7035(4)	5024(5)	9246(2)	56(1)
C(25)	7939(3)	5651(5)	9111(2)	58(1)
C(26)	8091(3)	5740(6)	8298(3)	62(1)
C(27)	7334(3)	5164(5)	7635(2)	50(1)
O(28)	5989(1)	4054(2)	4892(1)	26(1)
C(29)	6770(2)	5238(4)	4935(3)	51(1)
C(30)	7783(2)	4512(3)	4842(2)	31(1)

C(31)	8281(3)	3381(4)	5367(2)	46(1)
C(32)	9199(3)	2750(5)	5275(3)	66(1)
C(33)	9648(3)	3225(6)	4671(3)	66(1)
C(34)	9194(3)	4381(6)	4109(3)	70(2)
C(35)	8226(3)	5051(4)	4196(2)	45(1)
O(36)	4435(1)	4826(2)	3381(1)	27(1)
C(37)	5079(3)	3876(4)	2982(2)	42(1)
C(38)	4941(2)	4423(3)	2089(1)	28(1)
C(39)	5534(2)	5654(4)	1899(2)	36(1)
C(40)	5416(3)	6172(5)	1089(3)	58(1)
C(41)	4715(4)	5446(7)	450(2)	73(2)
C(42)	4129(3)	4235(8)	628(2)	73(2)
C(43)	4234(3)	3699(5)	1458(2)	49(1)
C(44)	2259(2)	4127(4)	3441(2)	34(1)
O(45)	1346(2)	5070(3)	3291(1)	47(1)
C(46)	1313(3)	6161(5)	2651(2)	55(1)
C(47)	1041(2)	5446(4)	1782(2)	37(1)
C(48)	1578(3)	5886(6)	1181(4)	70(1)
C(49)	1282(5)	5223(7)	364(3)	84(2)
C(50)	480(4)	4178(7)	186(2)	70(1)
C(51)	-28(3)	3773(6)	780(2)	59(1)
C(52)	252(2)	4378(4)	1578(2)	41(1)

Supplementary Table 3 │ Bond lengths [Å] and angles [°].

C(1)-O(20)	1.425(3)
C(1)-C(2)	1.522(3)
C(1)-C(6)	1.525(3)
C(1)-H(1)	1.0000
C(2)-O(28)	1.427(3)
C(2)-C(3)	1.522(3)
C(2)-H(2)	1.0000
C(3)-O(36)	1.428(2)
C(3)-C(4)	1.527(3)
C(3)-H(3)	1.0000
C(4)-O(5)	1.433(2)
C(4)-C(44)	1.512(3)
C(4)-H(4)	1.0000
O(5)-C(6)	1.419(3)
C(6)-O(7)	1.391(3)
C(6)-H(6)	1.0000
O(7)-C(8)	1.424(3)
C(8)-C(9)	1.520(3)
C(8)-C(13)	1.522(3)
C(8)-H(8)	1.0000
C(9)-O(14)	1.434(4)
C(9)-C(10)	1.562(4)
C(9)-H(9)	1.0000
C(10)-O(11)	1.398(4)
C(10)-O(17)	1.420(4)
C(10)-H(10)	1.0000
O(11)-C(12)	1.447(4)
C(12)-C(18)	1.512(4)
C(12)-C(13)	1.523(3)
C(12)-H(12)	1.0000
C(13)-O(16)	1.453(3)
C(13)-H(13)	1.0000
O(14)-C(15)	1.404(3)
C(15)-O(17)	1.415(3)

C(15)-O(16)	1.416(3)
C(15)-C(19)	1.495(4)
C(18)-H(18A)	0.9800
C(18)-H(18B)	0.9800
C(18)-H(18C)	0.9800
C(19)-H(19A)	0.9800
C(19)-H(19B)	0.9800
C(19)-H(19C)	0.9800
O(20)-C(21)	1.429(3)
C(21)-C(22)	1.505(4)
C(21)-H(21A)	0.9900
C(21)-H(21B)	0.9900
C(22)-C(27)	1.376(5)
C(22)-C(23)	1.385(4)
C(23)-C(24)	1.389(5)
C(23)-H(23)	0.9500
C(24)-C(25)	1.360(6)
C(24)-H(24)	0.9500
C(25)-C(26)	1.389(7)
C(25)-H(25)	0.9500
C(26)-C(27)	1.388(5)
C(26)-H(26)	0.9500
C(27)-H(27)	0.9500
O(28)-C(29)	1.433(3)
C(29)-C(30)	1.502(4)
C(29)-H(29A)	0.9900
C(29)-H(29B)	0.9900
C(30)-C(31)	1.364(4)
C(30)-C(35)	1.392(4)
C(31)-C(32)	1.355(6)
C(31)-H(31)	0.9500
C(32)-C(33)	1.321(7)
C(32)-H(32)	0.9500
C(33)-C(34)	1.396(8)
C(33)-H(33)	0.9500
C(34)-C(35)	1.426(6)

C(34)-H(34)	0.9500
C(35)-H(35)	0.9500
O(36)-C(37)	1.431(3)
C(37)-C(38)	1.508(3)
C(37)-H(37A)	0.9900
C(37)-H(37B)	0.9900
C(38)-C(43)	1.373(4)
C(38)-C(39)	1.388(4)
C(39)-C(40)	1.374(4)
C(39)-H(39)	0.9500
C(40)-C(41)	1.378(8)
C(40)-H(40)	0.9500
C(41)-C(42)	1.363(9)
C(41)-H(41)	0.9500
C(42)-C(43)	1.411(6)
C(42)-H(42)	0.9500
C(43)-H(43)	0.9500
C(44)-O(45)	1.419(3)
C(44)-H(44A)	0.9900
C(44)-H(44B)	0.9900
O(45)-C(46)	1.400(5)
C(46)-C(47)	1.517(5)
C(46)-H(46A)	0.9900
C(46)-H(46B)	0.9900
C(47)-C(52)	1.368(4)
C(47)-C(48)	1.381(5)
C(48)-C(49)	1.425(9)
C(48)-H(48)	0.9500
C(49)-C(50)	1.363(9)
C(49)-H(49)	0.9500
C(50)-C(51)	1.339(7)
C(50)-H(50)	0.9500
C(51)-C(52)	1.377(5)
C(51)-H(51)	0.9500
C(52)-H(52)	0.9500

O(20)-C(1)-C(2)	108.85(17)
O(20)-C(1)-C(6)	110.41(17)
C(2)-C(1)-C(6)	108.80(17)
O(20)-C(1)-H(1)	109.6
C(2)-C(1)-H(1)	109.6
C(6)-C(1)-H(1)	109.6
O(28)-C(2)-C(3)	109.55(17)
O(28)-C(2)-C(1)	111.30(18)
C(3)-C(2)-C(1)	111.09(17)
O(28)-C(2)-H(2)	108.3
C(3)-C(2)-H(2)	108.3
C(1)-C(2)-H(2)	108.3
O(36)-C(3)-C(2)	110.71(17)
O(36)-C(3)-C(4)	106.00(17)
C(2)-C(3)-C(4)	109.86(16)
O(36)-C(3)-H(3)	110.1
C(2)-C(3)-H(3)	110.1
C(4)-C(3)-H(3)	110.1
O(5)-C(4)-C(44)	106.78(19)
O(5)-C(4)-C(3)	110.22(17)
C(44)-C(4)-C(3)	112.54(18)
O(5)-C(4)-H(4)	109.1
C(44)-C(4)-H(4)	109.1
C(3)-C(4)-H(4)	109.1
C(6)-O(5)-C(4)	113.06(17)
O(7)-C(6)-O(5)	107.38(18)
O(7)-C(6)-C(1)	108.56(18)
O(5)-C(6)-C(1)	109.72(17)
O(7)-C(6)-H(6)	110.4
O(5)-C(6)-H(6)	110.4
C(1)-C(6)-H(6)	110.4
C(6)-O(7)-C(8)	115.81(18)
O(7)-C(8)-C(9)	111.4(2)
O(7)-C(8)-C(13)	111.06(19)
C(9)-C(8)-C(13)	104.20(18)
O(7)-C(8)-H(8)	110.0

C(9)-C(8)-H(8)	110.0
C(13)-C(8)-H(8)	110.0
O(14)-C(9)-C(8)	107.2(2)
O(14)-C(9)-C(10)	102.5(2)
C(8)-C(9)-C(10)	108.8(2)
O(14)-C(9)-H(9)	112.6
C(8)-C(9)-H(9)	112.6
C(10)-C(9)-H(9)	112.6
O(11)-C(10)-O(17)	111.2(2)
O(11)-C(10)-C(9)	114.0(2)
O(17)-C(10)-C(9)	103.5(2)
O(11)-C(10)-H(10)	109.3
O(17)-C(10)-H(10)	109.3
C(9)-C(10)-H(10)	109.3
C(10)-O(11)-C(12)	114.6(2)
O(11)-C(12)-C(18)	111.7(2)
O(11)-C(12)-C(13)	110.8(2)
C(18)-C(12)-C(13)	114.7(2)
O(11)-C(12)-H(12)	106.4
C(18)-C(12)-H(12)	106.4
C(13)-C(12)-H(12)	106.4
O(16)-C(13)-C(8)	111.54(19)
O(16)-C(13)-C(12)	113.90(18)
C(8)-C(13)-C(12)	106.43(19)
O(16)-C(13)-H(13)	108.3
C(8)-C(13)-H(13)	108.3
C(12)-C(13)-H(13)	108.3
C(15)-O(14)-C(9)	101.9(2)
O(14)-C(15)-O(17)	104.1(2)
O(14)-C(15)-O(16)	109.93(19)
O(17)-C(15)-O(16)	110.6(2)
O(14)-C(15)-C(19)	112.1(3)
O(17)-C(15)-C(19)	110.8(2)
O(16)-C(15)-C(19)	109.2(2)
C(15)-O(16)-C(13)	115.10(18)
C(15)-O(17)-C(10)	105.47(19)

C(12)-C(18)-H(18A)	109.5
C(12)-C(18)-H(18B)	109.5
H(18A)-C(18)-H(18B)	109.5
C(12)-C(18)-H(18C)	109.5
H(18A)-C(18)-H(18C)	109.5
H(18B)-C(18)-H(18C)	109.5
C(15)-C(19)-H(19A)	109.5
C(15)-C(19)-H(19B)	109.5
H(19A)-C(19)-H(19B)	109.5
C(15)-C(19)-H(19C)	109.5
H(19A)-C(19)-H(19C)	109.5
H(19B)-C(19)-H(19C)	109.5
C(1)-O(20)-C(21)	112.67(19)
O(20)-C(21)-C(22)	109.9(2)
O(20)-C(21)-H(21A)	109.7
C(22)-C(21)-H(21A)	109.7
O(20)-C(21)-H(21B)	109.7
C(22)-C(21)-H(21B)	109.7
H(21A)-C(21)-H(21B)	108.2
C(27)-C(22)-C(23)	118.9(3)
C(27)-C(22)-C(21)	122.9(3)
C(23)-C(22)-C(21)	118.2(3)
C(22)-C(23)-C(24)	120.3(3)
C(22)-C(23)-H(23)	119.9
C(24)-C(23)-H(23)	119.9
C(25)-C(24)-C(23)	120.8(3)
C(25)-C(24)-H(24)	119.6
C(23)-C(24)-H(24)	119.6
C(24)-C(25)-C(26)	119.4(3)
C(24)-C(25)-H(25)	120.3
C(26)-C(25)-H(25)	120.3
C(27)-C(26)-C(25)	120.0(4)
C(27)-C(26)-H(26)	120.0
C(25)-C(26)-H(26)	120.0
C(22)-C(27)-C(26)	120.6(3)
C(22)-C(27)-H(27)	119.7

C(26)-C(27)-H(27)	119.7
C(2)-O(28)-C(29)	111.74(19)
O(28)-C(29)-C(30)	109.5(2)
O(28)-C(29)-H(29A)	109.8
C(30)-C(29)-H(29A)	109.8
O(28)-C(29)-H(29B)	109.8
C(30)-C(29)-H(29B)	109.8
H(29A)-C(29)-H(29B)	108.2
C(31)-C(30)-C(35)	119.7(3)
C(31)-C(30)-C(29)	122.8(3)
C(35)-C(30)-C(29)	117.5(3)
C(32)-C(31)-C(30)	121.8(4)
C(32)-C(31)-H(31)	119.1
C(30)-C(31)-H(31)	119.1
C(33)-C(32)-C(31)	120.6(4)
C(33)-C(32)-H(32)	119.7
C(31)-C(32)-H(32)	119.7
C(32)-C(33)-C(34)	121.3(3)
C(32)-C(33)-H(33)	119.3
C(34)-C(33)-H(33)	119.3
C(33)-C(34)-C(35)	118.6(3)
C(33)-C(34)-H(34)	120.7
C(35)-C(34)-H(34)	120.7
C(30)-C(35)-C(34)	117.9(3)
C(30)-C(35)-H(35)	121.0
C(34)-C(35)-H(35)	121.0
C(3)-O(36)-C(37)	114.84(18)
O(36)-C(37)-C(38)	107.9(2)
O(36)-C(37)-H(37A)	110.1
C(38)-C(37)-H(37A)	110.1
O(36)-C(37)-H(37B)	110.1
C(38)-C(37)-H(37B)	110.1
H(37A)-C(37)-H(37B)	108.4
C(43)-C(38)-C(39)	119.6(3)
C(43)-C(38)-C(37)	120.2(3)
C(39)-C(38)-C(37)	120.2(3)

C(40)-C(39)-C(38)	121.0(3)
C(40)-C(39)-H(39)	119.5
C(38)-C(39)-H(39)	119.5
C(39)-C(40)-C(41)	119.9(4)
C(39)-C(40)-H(40)	120.1
C(41)-C(40)-H(40)	120.1
C(42)-C(41)-C(40)	119.7(3)
C(42)-C(41)-H(41)	120.1
C(40)-C(41)-H(41)	120.1
C(41)-C(42)-C(43)	121.1(4)
C(41)-C(42)-H(42)	119.5
C(43)-C(42)-H(42)	119.5
C(38)-C(43)-C(42)	118.7(4)
C(38)-C(43)-H(43)	120.6
C(42)-C(43)-H(43)	120.6
O(45)-C(44)-C(4)	111.6(2)
O(45)-C(44)-H(44A)	109.3
C(4)-C(44)-H(44A)	109.3
O(45)-C(44)-H(44B)	109.3
C(4)-C(44)-H(44B)	109.3
H(44A)-C(44)-H(44B)	108.0
C(46)-O(45)-C(44)	113.5(3)
O(45)-C(46)-C(47)	113.0(3)
O(45)-C(46)-H(46A)	109.0
C(47)-C(46)-H(46A)	109.0
O(45)-C(46)-H(46B)	109.0
C(47)-C(46)-H(46B)	109.0
H(46A)-C(46)-H(46B)	107.8
C(52)-C(47)-C(48)	118.9(3)
C(52)-C(47)-C(46)	120.6(3)
C(48)-C(47)-C(46)	120.4(4)
C(47)-C(48)-C(49)	118.9(4)
C(47)-C(48)-H(48)	120.6
C(49)-C(48)-H(48)	120.6
C(50)-C(49)-C(48)	120.1(4)
C(50)-C(49)-H(49)	120.0

C(48)-C(49)-H(49)	120.0
C(51)-C(50)-C(49)	119.8(4)
C(51)-C(50)-H(50)	120.1
C(49)-C(50)-H(50)	120.1
C(50)-C(51)-C(52)	121.3(4)
C(50)-C(51)-H(51)	119.3
C(52)-C(51)-H(51)	119.3
C(47)-C(52)-C(51)	120.9(3)
C(47)-C(52)-H(52)	119.5
C(51)-C(52)-H(52)	119.5

Symmetry transformations used to generate equivalent atoms.

Supplementary Table 4 │ Anisotropic displacement parameters ($Å^2$ x 10^3). The anisotropic displacement factor exponent takes the form: $-2\pi^2[h^2 a^{*2}U^{11} + ... + 2 h k a^* b^* U^{12}]$

	U^{11}	U^{22}	U^{33}	U^{23}	U^{13}	U^{12}
C(1)	26(1)	19(1)	20(1)	-4(1)	5(1)	-2(1)
C(2)	22(1)	20(1)	25(1)	-1(1)	8(1)	-2(1)
C(3)	24(1)	22(1)	19(1)	1(1)	9(1)	0(1)
C(4)	25(1)	29(1)	20(1)	-1(1)	8(1)	2(1)
O(5)	23(1)	34(1)	21(1)	-3(1)	8(1)	-2(1)
C(6)	27(1)	24(1)	19(1)	-2(1)	9(1)	0(1)
O(7)	42(1)	28(1)	25(1)	2(1)	20(1)	7(1)
C(8)	30(1)	29(1)	20(1)	-1(1)	11(1)	3(1)
C(9)	27(1)	44(1)	28(1)	-5(1)	3(1)	0(1)
C(10)	24(1)	51(2)	37(1)	1(1)	10(1)	4(1)
O(11)	37(1)	38(1)	40(1)	2(1)	15(1)	13(1)
C(12)	39(1)	28(1)	27(1)	-2(1)	15(1)	1(1)
C(13)	26(1)	26(1)	22(1)	-1(1)	9(1)	-2(1)
O(14)	37(1)	36(1)	35(1)	-10(1)	10(1)	-12(1)
C(15)	32(1)	32(1)	34(1)	-3(1)	14(1)	-4(1)
O(16)	30(1)	27(1)	24(1)	2(1)	7(1)	-2(1)
O(17)	34(1)	45(1)	40(1)	2(1)	21(1)	1(1)
C(18)	60(2)	36(1)	30(1)	-9(1)	18(1)	-4(1)
C(19)	57(2)	38(2)	55(2)	8(1)	28(2)	-7(1)
O(20)	35(1)	20(1)	22(1)	-2(1)	0(1)	-4(1)
C(21)	56(2)	28(1)	36(1)	6(1)	-10(1)	-6(1)
C(22)	44(1)	25(1)	33(1)	3(1)	-7(1)	1(1)
C(23)	55(2)	41(2)	40(1)	8(1)	1(1)	-4(1)
C(24)	82(3)	50(2)	28(1)	7(1)	-5(1)	0(2)
C(25)	60(2)	49(2)	50(2)	-2(2)	-23(2)	2(2)
C(26)	44(2)	63(2)	75(3)	-8(2)	4(2)	-11(2)
C(27)	54(2)	53(2)	42(2)	-2(1)	8(1)	-8(2)
O(28)	20(1)	26(1)	35(1)	-1(1)	8(1)	0(1)
C(29)	31(1)	29(1)	98(3)	-9(2)	24(2)	-4(1)
C(30)	23(1)	28(1)	44(1)	-4(1)	9(1)	-4(1)
C(31)	46(2)	42(2)	49(2)	12(1)	5(1)	-3(1)

C(32)	43(2)	53(2)	91(3)	-3(2)	-7(2)	13(2)
C(33)	29(1)	71(3)	95(3)	-41(3)	7(2)	6(2)
C(34)	69(2)	98(3)	59(2)	-44(2)	44(2)	-59(3)
C(35)	50(2)	41(2)	39(1)	3(1)	2(1)	-18(1)
O(36)	36(1)	25(1)	23(1)	4(1)	16(1)	6(1)
C(37)	59(2)	45(2)	31(1)	12(1)	29(1)	25(1)
C(38)	34(1)	30(1)	25(1)	-1(1)	15(1)	5(1)
C(39)	37(1)	36(1)	38(1)	2(1)	14(1)	1(1)
C(40)	63(2)	63(2)	60(2)	33(2)	38(2)	23(2)
C(41)	74(3)	119(4)	32(2)	27(2)	25(2)	55(3)
C(42)	55(2)	119(4)	37(2)	-30(2)	-5(2)	21(3)
C(43)	43(2)	56(2)	52(2)	-22(2)	16(1)	-9(1)
C(44)	28(1)	45(2)	25(1)	-9(1)	3(1)	1(1)
O(45)	26(1)	80(2)	33(1)	-8(1)	4(1)	12(1)
C(46)	48(2)	49(2)	57(2)	-8(2)	-13(2)	6(2)
C(47)	31(1)	40(1)	39(1)	9(1)	5(1)	6(1)
C(48)	53(2)	66(3)	97(3)	32(2)	28(2)	-3(2)
C(49)	99(4)	106(4)	66(3)	45(3)	57(3)	44(3)
C(50)	70(2)	102(4)	34(2)	-2(2)	6(2)	43(3)
C(51)	43(2)	74(3)	54(2)	-18(2)	0(1)	10(2)
C(52)	35(1)	49(2)	39(1)	1(1)	8(1)	0(1)

Supplementary Table 5 │ Hydrogen coordinates (x 10^4) and isotropic displacement parameters ($Å^2$x 10^3).

	x	y	z	U(eq)
H(1)	4616	3093	5742	26
H(2)	5040	5853	4880	26
H(3)	4190	3009	4080	25
H(4)	3098	5922	4116	29
H(6)	3536	6052	5620	28
H(8)	2475	6443	6346	30
H(9)	1013	4714	6071	40
H(10)	168	5859	7004	44
H(12)	2795	8109	7547	36
H(13)	3770	5959	7663	29
H(18A)	2307	8530	8829	61
H(18B)	3445	7757	8955	61
H(18C)	2457	6717	9036	61
H(19A)	2021	2728	9063	71
H(19B)	2368	1606	8392	71
H(19C)	1150	1905	8354	71
H(21A)	5851	2837	6891	52
H(21B)	4957	3599	7295	52
H(23)	5647	4016	8688	56
H(24)	6924	4976	9800	68
H(25)	8461	6025	9568	70
H(26)	8713	6195	8196	74
H(27)	7445	5218	7081	60
H(29A)	6875	5784	5480	61
H(29B)	6535	6007	4483	61
H(31)	7975	3025	5808	56
H(32)	9522	1959	5649	79
H(33)	10293	2772	4619	80
H(34)	9523	4715	3676	85
H(35)	7893	5841	3825	54

H(37A)	4868	2773	2994	50
H(37B)	5824	3968	3279	50
H(39)	6028	6146	2335	44
H(40)	5817	7029	969	70
H(41)	4641	5788	-112	88
H(42)	3642	3743	187	87
H(43)	3824	2853	1577	59
H(44A)	2534	4070	2924	40
H(44B)	2074	3061	3583	40
H(46A)	2004	6679	2732	66
H(46B)	785	6965	2692	66
H(48)	2134	6617	1309	84
H(49)	1645	5509	-56	101
H(50)	281	3738	-359	84
H(51)	-592	3055	650	70
H(52)	-109	4049	1992	49

Supplementary References

1. Valente, L., Olesker, A., Rabanal, R., Barata, L. E. S. & Lukacs, G. Synthesis of nogalose, a component of the antitumor antibiotic nogalamycin. *Tetrahedron Lett.* **13**, 1153-1156 (1979).
2. Schmidt, R. R. & Michel, J. Facile synthesis of α- and β-O-glycosyl imidates; preparation of glycosides and disaccharides. *Angew. Chem. Int. Ed. Engl.* **19**, 731-732 (1980).
3. Hsu, C. H. et al. Highly α-selective sialyl phosphate donors for efficient preparation of natural sialosides. *Chem. – Eur. J.* **16**, 1754-1760, (2010).
4. Mo, K.-F., Li, H., Mague, J. T. & Ensley, H. E. Synthesis of the β-1,3-glucan, laminarahexaose: NMR and conformational studies. *Carbohydr. Res.* **344**, 439-447 (2009).
5. Meldgaard, M., Hansen, F. G. & Wengel, J. 3 '-C-branched LNA-type nucleosides locked in an N-type furanose ring conformation: Synthesis, incorporation into oligodeoxynucleotides, and hybridization studies. *J. Org. Chem.* **69**, 6310-6322 (2004).
6. Grob, V. D., Squires, T. G. & Vercellotti, J. R. Glycosyl chlorides through reaction with zinc chloride-thionyl chloride. *Carbohydr. Res.* **10**, 595-597 (1969).
